# Supplementary material for: Bayesian-optimization-assisted discovery of stereoselective aluminum complexes for ring-opening polymerization of racemic lactide
Source: Nat Commun. 2023 Jun 20;14:3647. doi: 10.1038/s41467-023-39405-5 (PMC10282063; doi:10.1038/s41467-023-39405-5)
Supplement: Supplementary file 1 — Supplementary information [file 41467_2023_39405_MOESM1_ESM.pdf]

Bayesian-Optimization-Assisted Discovery of Stereoselective Aluminum Complexes for Ring-Opening Polymerization of Racemic Lactide

Xiaoqian Wang[1],* Yang Huang[1],* Xiaoyu Xie[1],* Yan Liu[1], Ziyu Huo[1], Maverick Lin[1], Hongliang Xin[1,†] & Rong Tong[1,†]

[1]Department of Chemical Engineering, Virginia Polytechnic Institute and State University, 635 Prices Fork Road, Blacksburg, Virginia, 24061, United States

[†] To whom correspondence should be addressed. E-mail: hxin@vt.edu (H.X.); rtong@vt.edu (R.T.)
* These authors contributed equally to this work.

Supplementary Information

Table of contents

S1. General	S-3
S2. Ligand and Aluminum Complex Synthesis	S-5
S3. Polymerization Procedures	S-39
S4. Computation Methods	S-40
S5. Supplementary Tables	S-52
S6. Supplementary Figures	S-72
S7. NMR Spectra of ligands	S-100
S8. NMR Spectra of Al complexes	S-138
S9. Supplementary References	S-169

S1. General

S1.1 Materials

rac-lactide (*rac*-LA) and L-LA (Sigma-Aldrich, St. Louis, MO) were recrystallized three times from toluene, dried and stored in the glove box freezer (−30 ºC). Anhydrous tetrahydrofuran (THF) was dried by alumina columns and stored with 4Å molecular sieve in the dark bottle in the glove box (MBraun, Labstar Pro, < 1 ppm oxygen and moisture). Anhydrous THF-d_8, benzyl alcohol, hexane, toluene, diethyl ether, triethylamine and dichloromethane were dried and stored by 4Å molecular sieves in the glove box. All other chemicals were purchased from Sigma-Aldrich (St. Louis, MO) unless otherwise noted. All metal catalysts were synthesized in the glove box, characterized by NMR, and stored in the glove box freezer (−30 ºC).

S1.2 NMR spectroscopy

All room temperature NMR and homodecoupling ^{1}H NMR spectra were recorded on Agilent U4-DD2 (400 MHz) or Bruker Avance II (500 MHz). All ^{13}C NMR spectra are proton decoupled. All of the high temperature ^{1}H NMR spectra were recorded at 85 degree on Bruker Avance III (600 MHz) using toluene-d_8 as the solvent.

Determination of LA conversion: the conversion of LA in the polymerization was determined by integrating the methine region of PLA (δ = 5.17 ppm in $CDCl_3$) versus that of the lactide monomer (δ = 5.04 ppm in $CDCl_3$).

Determination of PLA stereochemistry: the microstructure analysis of PLA was examined of the methine region in the homodecoupled ^{1}H NMR (δ = 5.14-5.24 ppm) and ^{13}C NMR (δ = 69.0-69.5 ppm), following the literature assignment.[1-3] See Supplementary Figs. 6-11.

S1.3 Size-exclusion chromatography (SEC)

SEC experiments were performed on a system equipped with an isocratic pump with degasser (Agilent 1260 series, Agilent Technologies, Santa Clara, CA, USA), Wyatt DAWN HELEOS multiangle laser light scattering (MALS) detector (GaAs 30 mW laser at λ=690nm), and an Wyatt Optilab rEX differential refractive index (DRI) detector with a 690 nm light source (Wyatt Technology, Santa Barbara, CA, USA). Separations were performed using serially connected size-exclusion columns (100 Å, 500 Å, 10^3 Å, and 10^4 Å Phenogel columns, 5 μm, 300 × 4.6 mm, Phenomenex, Torrance, CA, USA) at 35 °C using THF or chloroform (for stereoblock PLA having low solubility in THF) as the mobile

phase with a flow rate of 0.35 mL/min. The polymer molecular weight (MW) and molecular weight distribution (*Đ*) were determined using Zimm model fit of MALS-DRI data by ASTRA software (Version 6.1, Wyatt Technology). Data collection interval: 0.5 sec.

The refractive index increment dn/dc value was determined by the Wyatt Optilab rEX refractive index detector using ASTRA software dn/dc template (Version 6.1, Wyatt Technology). Five polymer / THF solutions with different concentrations were sequentially injected into the refractive index detector and the refractive index values were plotted versus concentration in ASTRA software. The slope of the linear fitting data is the dn/dc value.

The dn/dc values: poly(L-LA), 0.042.

S1.4 Differential scanning calorimetry (DSC)

DSC measurements were performed on TA Instruments DSC Q2000 instrument equipped with photocalorimeter accessory and RCS90 cooling system. Polymer samples in crimped aluminum pans were analyzed under nitrogen at a heating rate of 10 °C/min from 0 to 230 °C. Glass transition temperature (T_g) and melting temperature (T_m) were obtained and reported from the second heating run.

S1.5 Mechanical testing of polymers

Solvent-cast polyester sheets were prepared by pouring dichloromethane (DCM) solutions of PLA (~200 mg/mL) into a polydimethylsiloxane mold. The solvent was slowly evaporated in the fume hood over 1-2 weeks, and DCM peak was no longer observed in ^{1}H NMR spectroscopy, confirming that the polyester sheets were sufficiently dry for mechanical tests. Tensile stress-strain testing was performed by an Instron 5966 universal testing system (10 kN load cell) or dynamic mechanical analyzer (DMA, Q800, TA instrument) on test specimens (10 mm × 4 mm × 0.2 mm, length × width × thickness) at 10 mm/min or 5 N/min extension rate, using the ASTM D882-18 standard, at ambient temperature (22 ºC) and humidity (~ 16%). All values are reported as the average of three to five samples.

S2. Ligand and Aluminum Complex Synthesis

S2.0 General ligand synthetic routes

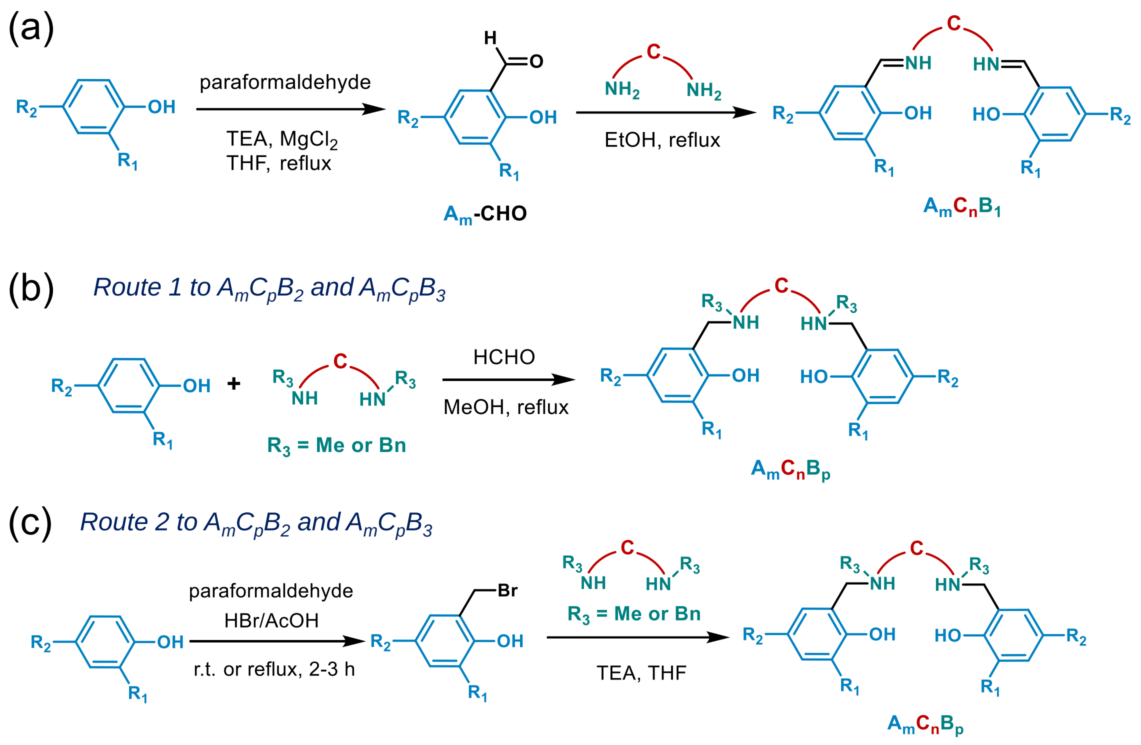

Supplementary Figure 1 (a) Scheme of the synthesis of salicylaldehydes and the salen ligands $A_mC_pB_1$. (b) Scheme of the synthesis of the ligands $A_mC_pB_2$ and $A_mC_pB_3$ (route 1). (c) Scheme of the synthesis of the ligands $A_mC_pB_2$ and $A_mC_pB_3$ (route 2).

The salicylaldehyde forms of A_1, A_2, A_3, A_4, A_6, A_7 and A_{16} are commercially available (Sigma-Aldrich), and these salicylaldehydes can directly react with diamines to form the ligand, as shown in Supplementary Fig. 1a. The rest salicylaldehyde can be prepared from the phenol form. We note that the phenols of A_{10}, A_{12} and A_{15} are not commercially available, and request multi-step reactions to prepare.[4, 5]

The synthesis of $A_mC_pB_2$ and $A_mC_pB_3$ have two routes (Supplementary Fig. 1b-c). The first route usually can be applicable to prepare $A_mC_1B_2$ and $A_mC_1B_3$ by the one-step reaction between phenol and *N,N'*-dimethyl-diamine or *N,N'*-dibenzyl-diamine (Supplementary Fig. 1b).[6, 7]

The second route is to prepare *o*-bromomethylphenol from phenol first. The obtained *g*-bromomethylphenol then reacts with *N,N'*-dimethyl-diamine or *N,N'*-dibenzyl-diamine to synthesize the ligand (Supplementary Fig. 1c).

S2.1 Synthesis of Salicylaldehydes (A_m-CHO)

Salicylaldehydes (A_8-CHO, A_{11}-CHO, A_{14}-CHO)were synthesized from phenol based on the reported procedures.[8] In brief, in a glove box, the phenol (10 mmol) was dissolved in anhydrous THF (50 mL) in a flask with a stirrer bar, followed by the addition of dry triethylamine (37.5 mmol) and anhydrous $MgCl_2$ (15 mmol). The solution was stirred for 15 min, and dry paraformaldehyde (67.5 mmol) was added into the mixture. The flask was sealed and taken out of the box, and was heated at reflux temperature for about 4 hours. The solution was allowed to cool to room temperature, followed by the addition of 5% aqueous HCL (80 mL) under stirring. The mixture was stirred for 30 minutes, and was extracted by diethyl ether (3 × 100 mL). The ether fraction was combined, washed by saturated NaCl solution, and dried by $MgSO_4$. The ether fraction was dried, and was purified by flash chromatography using diethyl ether/hexane as eluent.

S2.2 Synthesis of *N*,*N*'-diphenyl-diamine (C_nB_3)

***N*1,*N*3-dibenzyl-2,2-dimethylpropane-1,3-diamine** was synthesized from 2,2-dimethyl-1,3-propanediamine based on the reported procedures.[9] In brief, 2,2-dimethyl-1,3-propanediamine (4.7 mmol, 481.4 mg) was dissolved in dry methanol (15 mL) in a flask with a stirrer bar, followed by the addition of benzaldehyde (9.4 mmol, 0.958 mL). The solution was refluxed for 10 h and then cooled via an ice bath. Addition of $NaBH_4$ (20.2 mmol, 766.4 mg) was performed slowly and in small portions to prevent boiling, and the reaction mixture was stirred for 4 h until completion. The solvent was evaporated in vacuo, and then saturated $NaHCO_3$ (20 mL), water (20 mL) and DCM (80 mL) was added. The aqueous layer was extracted twice more with DCM. The combined organic layers were dried by $MgSO_4$, filtered, and concentrated in vacuo to afford a yellow oil.

***N*1,*N*2-dibenzylbenzene-1,2-diamine** (C_3B_3, C_6B_3) were synthesized according to literature.[10] In a round-bottom flask, triethylamine (2.0 equiv, 2.019 g) was added to a solution of 1,2-phenylenediamine (1.0 equiv, 1.08 g) and benzyl bromide (2.0 equiv, 3.413 g) in dry MeCN (15 mL). The resulting solution was stirred at room temperature overnight.

Then, after evaporation of the solvent, the reaction mixture was extracted with DCM (100 mL × 2), washed with water (100 mL × 2) and brine (100 mL × 1), dried over $MgSO_4$, and filtered. The solvent was evaporated under reduced pressure, and the residue was purified by flash chromatography using AcOEt/hexane (1:9) as eluent.

S2.3 Synthesis of *ortho*-bromomethylphenol (A_m-CH_2Br)

The *o*-bromomethylphenol that was used in route 2 in Scheme S3 was synthesized according to the literature.[11, 12] Attempts to prepare A_4-CH_2Br and A_9-CH_2Br were not successful.

For A_3-CH_2Br and A_{14}-CH_2Br,[11] the phenol (1 equiv.) and paraformaldehyde (1.1 equiv.) was added into 20 mL acetic acid, followed by the addition of HBr solution (33% in acetic acid). After stirring for 3 hours, the solution was evaporated, and the crude product was purified by column chromatography using DCM/hexane as eluent.

For A_5-CH_2Br and A_6-CH_2Br,[12] a HBr solution (30 mL, 33 % in acetic acid) was cooled in ice, and then the phenol (12.3 mmol, 1 equiv.) and paraformaldehyde (3.1 equiv.) were successively added. The mixture was heated up to 80 ºC for 2 hours. A white precipitate formed after cooling the mixture to room temperature. The solid was filtered off, washed with excess water and petroleum ether, and dried.

S2.4 Synthesis of $A_mB_1C_p$ ligands

The $A_mB_1C_p$ ligands ($A_1Y_6B_1$, $A_3Y_8B_1$, $A_4C_2B_1$, $A_4C_6B_1$, $A_4C_8B_1$, $A_7C_2B_1$, $A_8C_2B_1$, $A_8C_5B_1$, $A_8C_9B_1$, $A_8C_{11}B_1$, $A_{11}C_2B_1$, $A_{11}C_3B_1$, $A_{11}C_5B_1$, $A_{11}C_8B_1$, $A_{14}C_3B_1$, $A_{14}C_5B_1$, $A_{14}C_{11}B_1$, $A_{16}C_7B_1$, $A_{16}C_{11}B_1$) were prepared according to the reported procedures.[4, 13] In brief, the salicylaldehyde (2 equiv., 5 mmol) and the appropriate diamine (1 equiv., 2.5 mmol) was dissolved in Ethanol (10 mL) at room temperature. The mixture was heated at reflux for overnight. A precipitate generally formed during the reaction. The solution was allowed to cool down to room temperature, and the solid was collected by filtration. The obtained solid was washed with cold methanol and dried in vacuum.

S2.5 Synthesis of $A_mB_2C_p$ and $A_mB_3C_p$ ligands

Route 1 (Supplementary Fig. 1b):

The ligand was prepared according to the reported procedures.[6, 7] To a stirred solution of *N,N'*-dimethyl-diamine or *N,N'*-dibenzyl-diamine (1 equiv., 40.0 mmol) and diphenol (2

equiv., 80.0 mmol) in methanol (10 mL) was added aqueous formaldehyde solution (37 wt%, 10 equiv.). After stirring at 80 ºC overnight, the reaction was cooled to room temperature. The precipitate was filtered and washed repetitively by methanol to remove residue starting materials. In some cases, the precipitates were oil, and it could be purified by flash chromatography using dichloromethane/hexane as eluent. NMR spectra of ligands $A_2C_1B_3$, $A_4C_1B_2$, $A_4C_1B_3$, $A_5C_1B_2$, $A_5C_1B_3$, $A_{16}C_1B_2$, $A_{16}C_1B_3$ are in section S7.

Route 2 (Supplementary Fig. 1c):

The ligand was prepared according to the reported procedures.[11] In brief, To a solution of *N,N'*-dimethyl-diamine or *N,N'*-dibenzyl-diamine (5 mmol) and TEA (10 mmol) in THF (10 mL), the *o*-bromomethylphenol (10 mmol) in THF (10 mL) was added dropwise, and the reaction mixture was stirred at room temperature for overnight. Precipitate was removed by filtration and the solvent was evaporated. The crude product was purified by flash chromatography using dichloromethane / methanol as eluent. NMR spectra of ligands $A_3C_6B_2$, $A_5C_2B_2$, $A_5C_2B_3$, $A_5C_3B_3$, $A_5C_5B_2$, $A_5C_6B_2$, $A_5C_6B_3$, $A_6C_2B_2$, $A_6C_6B_2$ are in section S7.

Round 1 ligands in Table S3:

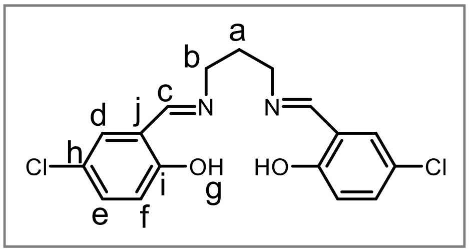

$A_7C_2B_1$. Followed the general procedure of $A_mB_1C_p$ (S2.4). The crude solid was washed with cold methanol to afford the ligand as a yellow powder (72% yield). ^{1}H NMR (400 MHz, $CDCl_3$) δ 13.34 (s, 2H, g), 8.31 (s, 2H, c), 7.25 (m, 4H, d, e), 6.91 (d, 2H, f), 3.72 (t, 4H, b), 2.13 (m, 2H, a). ^{13}C NMR (125 MHz, $CDCl_3$) δ 164.50, 159.79, 132.31, 130.52, 123.37, 119.54, 118.69, 56.99, 31.63.

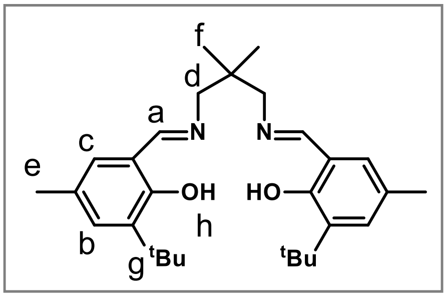

$A_{11}C_3B_1$. Followed the general procedure of $A_mB_1C_p$ (S2.4). The crude solid was purified by flash chromatography on silica gel using hexane/ether (5/1) to afford the ligand as a yellow powder (47% yield). ^{1}H NMR (400 MHz, $CDCl_3$) δ 13.78 (s, 2H, h), 8.30 (s, 2H, a), 7.15 (s, 2H, b), 6.92 (s, 2H, c), 3.49 (s, 4H, d), 2.30 (s, 6H, e), 1.46 (s, 18H, g), 1.10 (s, 6H, f). ^{13}C NMR (125 MHz, $CDCl_3$) δ 166.56, 158.35, 137.25, 130.77, 129.74, 126.64, 118.48, 68.56, 36.43, 29.53, 24.68, 20.80.

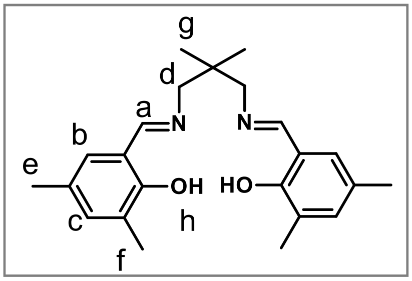

$A_{14}C_3B_1$. Followed the general procedure of $A_mB_1C_p$ (S2.4). The crude solid was washed with cold methanol to afford the ligand as a yellow-green powder (65% yield). ^{1}H NMR (400 MHz, $CDCl_3$) δ 13.51 (s, 2H, h), 8.27 (s, 2H, a), 7.02 (s, 2H, b), 6.90 (s, 2H, c), 3.48 (s, 4H, d), 2.27 (s, 12H, e, f), 1.08(s, 6H, g). ^{13}C NMR (125 MHz, $CDCl_3$) δ 166.00, 157.32, 134.66, 129.11, 127.22, 125.71, 117.77, 68.38, 26.34, 24.53, 20.45, 15.59.

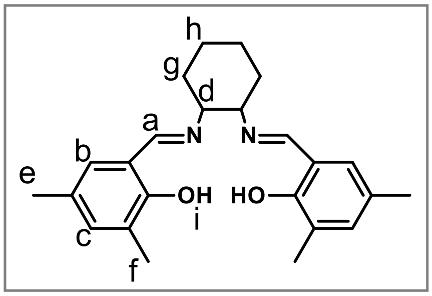

$A_{14}C_5B_1$. Followed the general procedure of $A_mB_1C_p$ (S2.4). The crude solid was washed with cold methanol and hexane to afford the ligand as a yellow powder (60% yield). ^{1}H NMR (400 MHz, $CDCl_3$) δ 13.37 (s, 2H, i), 8.19 (s, 2H, a), 6.94 (s, 2H, b), 6.80 (s, 2H, c), 3.29-3.27 (m, 2H, d), 2.21, 2.79 (s, 12H, e, f), 1.93-1.43 (m, 8H, g, h). ^{13}C NMR (125 MHz, $CDCl_3$) δ 164.88, 157.10, 134.26, 129.23, 127.14, 125.43, 117.71, 72.83, 33.35, 24.32, 20.38, 15.50.

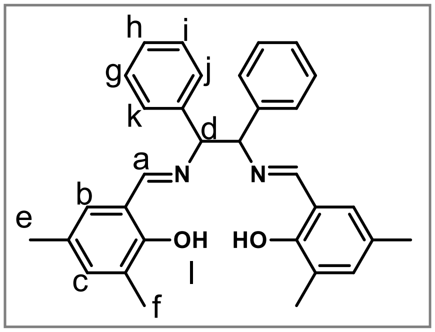

$A_{14}C_{11}B_1$. Followed the general procedure of $A_mB_1C_p$ (S2.4). The crude solid was washed with cold methanol to afford the ligand as a pale, off-white powder (65% yield). ^{1}H NMR (400 MHz, $CDCl_3$) δ 13.24 (s, 2H, l), 8.31 (s, 2H, a), 7.2-7.1(m, 10H, k, g, h, i, j), 6.95 (s, 2H, b), 6.78 (s, 2H, c), 4.69 (s, 2H, d), 2.24, 2.18 (s, 12H, e, f). ^{13}C NMR (125 MHz, $CDCl_3$)

δ 166.41, 157.03, 139.74, 134.71, 129.56, 128.44, 128.04, 127.61, 127.32, 125.48, 117.71, 80.62, 20.36, 15.56.

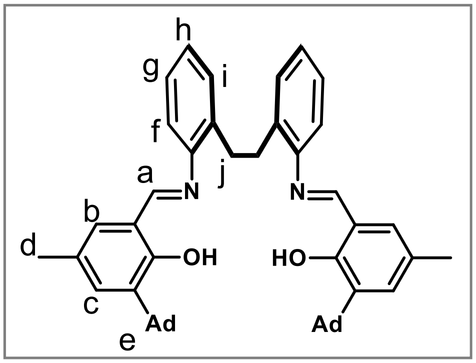

$A_8C_9B_1$. Followed the general procedure of $A_mB_1C_p$ (S2.4). The crude solid was washed with cold methanol to afford the ligand as a yellow powder (55% yield). ^{1}H NMR (400 MHz, $CDCl_3$) δ 8.49 (s, 2H, a), 7.54(dd, 2H, b), 7.25-7.02 (m, 10H, f, g, c, h, i), 4.19 (s, 4H, j), 2.33 (s, 6H, d), 2.28 (d, 12H, e), 2.14 (s, 6H, e), 1.85 (q, 12H, e). ^{13}C NMR (125 MHz, $CDCl_3$) δ 163.25, 158.77, 137.80, 136.25, 131.60, 130.99, 130.42, 127.61, 127.27, 127.00, 119.08, 117.73, 77.41, 77.16, 76.91, 40.46, 37.34, 37.18, 34.36, 31.10, 29.33, 20.90.

Round 1 ligands in Table S4

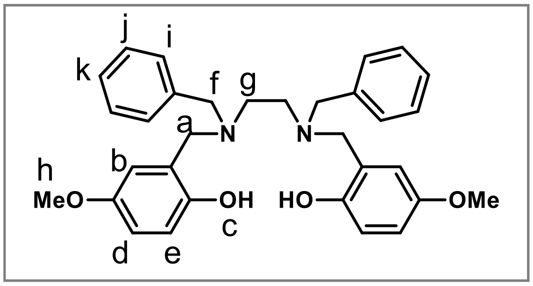

$A_4C_1B_3$. Followed the general route 1 procedures in $A_mB_3C_p$ (S2.5). The product was purified by flash chromatography on silica gel using DCM/hexane (5/1) to afford the ligand as a white powder (45% yield). ^{1}H NMR (400 MHz, $CDCl_3$) δ 10.01 (s, 2H, c), 7.32-7.12 (m, 10H, i, j, k), 6.76-6.49 (m, 6H, b, d, e), 3.73 (s, 6H, h), 3.62 (s, 4H, a), 3.51 (s, 4H, f), 2.68 (s, 4H, g). ^{13}C NMR (125 MHz, $CDCl_3$) δ 152.74, 151.33, 136.32, 129.64, 128.78, 127.90, 122.45, 116.70, 114.72, 113.88, 58.59, 58.37, 55.84, 50.09.

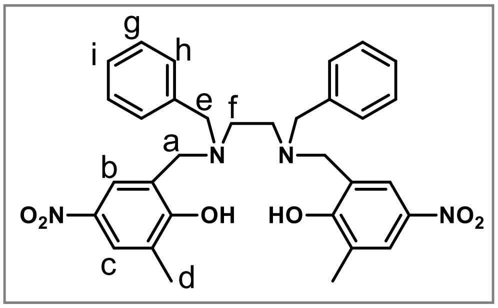

$A_5C_1B_3$. Followed the general route 1 procedures in $A_mB_3C_p$ (S2.5). The crude solid was washed with cold methanol to afford the ligand as an off-white powder (60% yield). ^{1}H NMR (400 MHz, CDCl3) δ 7.95 (d, 2H, b), 7.72 (d, 2H, c), 7.37-7.28 (m, 6H, g, h, i), 7.17-7.15 (m, 4H, j, k), 3.72 (s, 4H, a), 3.57 (s, 4H, e), 2.69 (s, 4H, f), 2.21 (s, 6H, d). ^{13}C NMR (125 MHz, CDCl3) δ 162.19, 139.90, 135.37, 129.51, 129.09, 128.46, 126.39, 126.14, 122.59, 120.57, 77.41, 77.16, 76.91, 58.97, 57.95, 51.09, 49.86, 15.84.

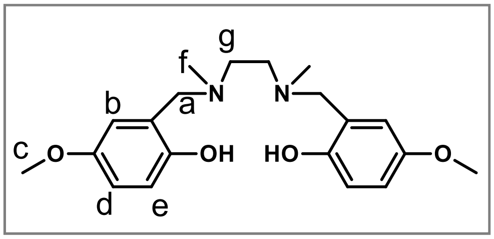

$A_4C_1B_2$. Followed the general route 1 procedures in $A_mB_2C_p$ (S2.5). The crude solid was washed with cold methanol to afford the ligand as a white powder (60% yield). ^{1}H NMR (400 MHz, $CDCl_3$) δ 6.80-6.69 (m, 4H, d, e), 6.53 (s, 2H, b), 3.73 (s, 6H, c), 3.65 (s, 4H, a), 2.69 (s, 4H, g), 2.27 (s, 6H, f). ^{13}C NMR (125 MHz, $CDCl_3$) δ 152.62. 151.65, 122.44, 116.71, 114.54, 113.79, 61.99, 55.86, 54.24, 41.88.

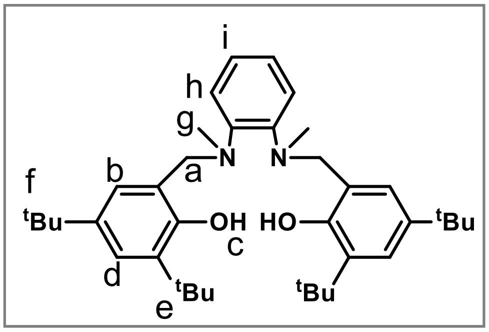

$A_3C_6B_2$. Followed the general route 2 procedures in $A_mB_2C_p$ (S2.5). The product was purified by flash chromatography on silica gel using DCM/hexane (5/1) to afford the ligand as an off-white powder (45% yield). ^{1}H NMR (400 MHz, $CDCl_3$) δ 9.73 (s, 2H, c), 7.41-7.35 (m, 2H, d), 7.22-7.14 (m, 4H, b, i), 6.85 (d, 2H, h), 4.04 (s, 4H, a), 2.71 (s, 6H, g), 1.29 (d, 36H, e, f). ^{13}C NMR (125 MHz, $CDCl_3$) δ 153.71, 148.14, 140.71, 136.07, 126.25, 124.69, 123.20, 122.89, 121.63, 77.41, 77.16, 76.91, 61.04, 44.35, 35.01, 34.23, 31.79, 31.74, 29.77.

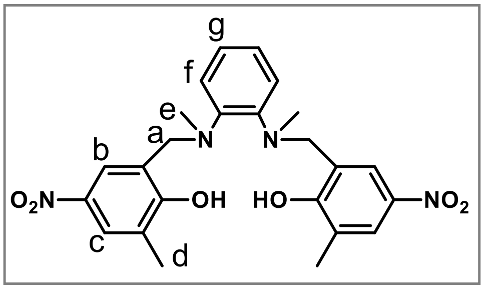

$A_5C_6B_2$. Followed the general route 2 procedures in $A_mB_2C_p$ (S2.5). The crude solid was washed with cold methanol and hexane to afford the ligand as a brown powder (55% yield). ^{1}H NMR (400 MHz, $CDCl_3$) δ 8.01 (d, 2H, b), 7.89 (d, 2H, c), 7.42-7.26 (m, 4H, g, f), 4.06 (s, 4H, a), 2.77 (s, 6H, e), 2.14 (s, 6H, d). ^{13}C NMR (125 MHz, $CDCl_3$) δ 161.70, 146.82, 139.98, 127.56, 126.92, 126.46, 123.97, 122.74, 121.18, 76.91, 61.26, 43.12, 16.00.

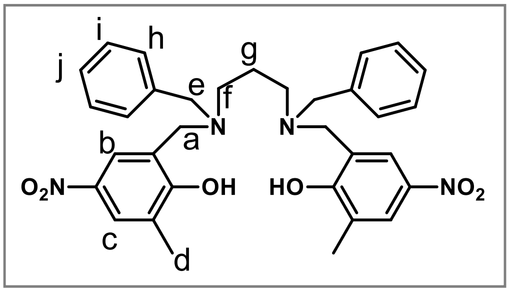

$A_5C_2B_3$. Followed the general route 2 procedures in $A_mB_3C_p$ (S2.5). The crude solid was purified by flash chromatography on silica gel using DCM/methanol (10/1) to afford the ligand as a yellow-green powder (48 % yield). ^{1}H NMR (400 MHz, $CDCl_3$) δ 7.97 (s, 2H, b), 7.78 (s, 2H, c), 7.38-7.17 (m, 10H, h, i, j, k, l), 3.77 (s, 4H, a), 3.60 (s, 4H, e), 2.48 (t,

4H, f), 1.86 (m, 2H, g). ^{13}C NMR (125 MHz, $CDCl_3$) δ 162.48, 139.81, 135.59, 129.60, 129.00, 128.31, 126.28, 126.00, 122.61, 120.77, 58.34, 57.27, 50.94, 23.27, 15.86.

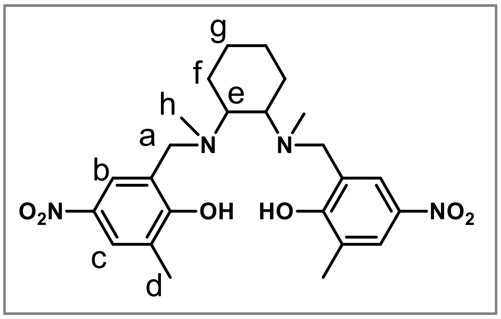

$A_5C_5B_2$. Followed the general route 2 procedures in $A_mB_2C_p$ (S2.5). The crude solid was washed with cold methanol and hexane to afford the ligand as a yellow powder (35% yield). ^{1}H NMR (400 MHz, THF-*d*$_8$) δ 7.95 (dd, 4H, c, b), 3.99 (d, 2H, a), 3.72 (s, 2H, a), 3.43 (s, 2H, e), 2.40 (m, 12H, d, h), 1.82 (m, 2H, f), 1.59 (m, 2H, f), 1.44-1.23 (m, 4H, g). ^{13}C NMR was not able to acquire due to the low solubility of the ligand. ESI-MS confirmed the complex: exact mass calculated for $[M+H]^+$ ($C_{24}H_{33}N_4O_6$): 473.2395; found 473.2397.

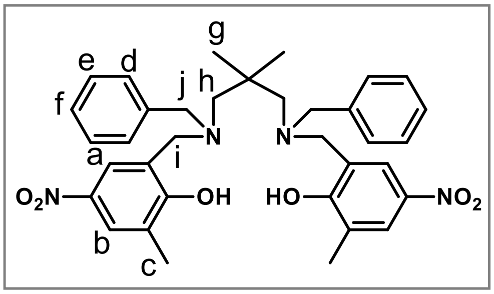

$A_5C_3B_3$. Followed the general route 2 procedures in $A_mB_3C_p$ (S2.5). The product was purified by flash chromatography on silica gel using DCM/methanol (10/1) to afford the ligand as a yellow wax (30% yield). ^{1}H NMR (400 MHz, $CDCl_3$) δ 8.01 (d, 2H, a), 7.81 (d, 2H, b), 7.43-7.13 (m, 10H, d, e, f), 3.80 (s, 4H, i), 3.45 (s, 4H, j), 2.41-2.28 (s, 6H, c), 2.22 (s, 4H, h), 0.80 (s, 6H, g). ^{13}C NMR (125 MHz, $CDCl_3$) δ 162.25, 139.73, 135.79, 130.61, 128.68, 128.24, 127.69, 127.01, 126.28, 125.89, 122.60, 121.18, 77.27, 77.06, 76.85, 63.94, 61.01, 60.19, 36.69, 24.98, 15.91.

Round 2 ligands in Table S5

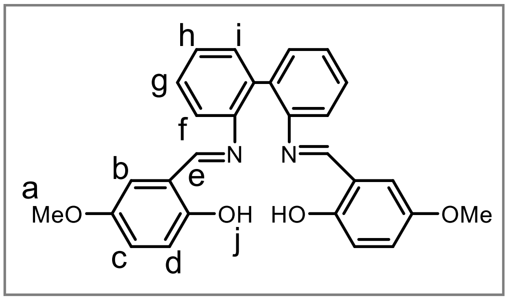

$A_4C_8B_1$. Followed the general procedure of $A_mB_1C_p$ (S2.4). The crude solid was washed with cold methanol to afford the ligand as an orange powder (70% yield). ^{1}H NMR (400 MHz, $CDCl_3$) δ 11.99 (s, 2H, j), 8.32 (s, 2H, e), 7.48-7.34 (m, 6H, g, f, h), 7.19 (d, 2H, i), 6.88-6.77 (m, 4H, c, b), 6.42 (d, 2H, d), 3.67 (s, 6H, a). ^{13}C NMR (125 MHz, $CDCl_3$) δ 162.73, 155.11, 152.13, 147.71, 134.87, 130.90, 129.44, 127.06, 120.60, 119.13, 118.07, 117.91, 114.67, 55.75.

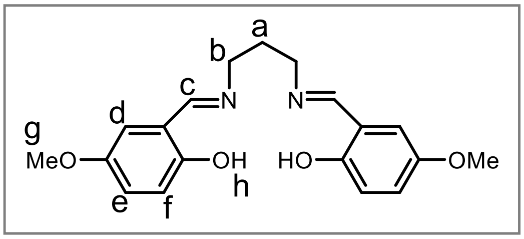

$A_4C_2B_1$. Followed the general procedure of $A_mB_1C_p$ (S2.4). The crude solid was washed with cold methanol to afford the ligand as a yellow powder (75% yield). ^{1}H NMR (400 MHz, $CDCl_3$): δ 12.90 (s, 2H, h), 8.33 (d, 2H, c), 7.07-6.82 (m, 4H, e, d), 6.76 (dd, 2H, f), 3.78 (s, 6H, g), 3.72 (td, 4H, b), 2.12 (p, 2H, a). ^{13}C NMR (125 MHz, $CDCl_3$) δ 165.30, 155.31, 152.15, 119.42, 118.50, 117.82, 114.93, 77.41, 77.16, 76.91, 57.08, 56.07, 31.79.

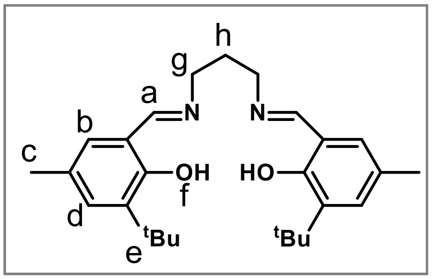

$A_{11}C_2B_1$ Followed the general procedure of $A_mB_1C_p$ (S2.4). The crude solid was washed with cold methanol to afford the ligand as a yellow-green powder (72% yield). ^{1}H NMR (400 MHz, $CDCl_3$) δ 13.76 (s, 2H, f), 8.33 (s, 2H, a), 7.14 (s, 2H, b), 6.91 (s, 2H, d), 3.71 (t, 4H, g), 2.29 (s, 6H, c), 2.13 (m, 2H, h), 1.44 (s, 18H, e). ^{13}C NMR (125 MHz, $CDCl_3$) δ 166.23, 158.27, 137.26, 130.57, 129.63, 126.69, 118.46, 57.03, 34.88, 31.83, 31.09, 29.53, 20.81.

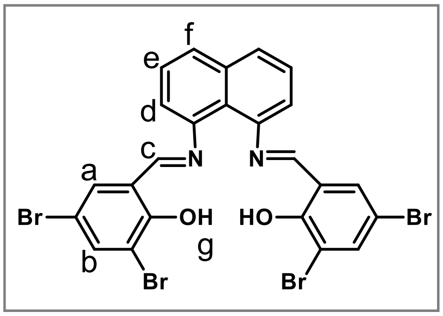

$A_{16}C_7B_1$. Followed the general procedure of $A_mB_1C_p$ (S2.4). The product was purified by flash chromatography on silica gel using ether/hexane (1/1) to afford the ligand as a brown powder (65% yield). ^{1}H NMR (400 MHz, DMSO-d_6) δ 7.77, 7.58 (d, 2H, c), 7.22-7.07 (m, 6H, d, e, f), 6.83 (s, 2H, g), 6.58 (d, 4H, a, b). ^{13}C NMR (125 MHz, DMSO-d_6) δ 152.06, 142.06, 134.20, 134.18, 131.00, 130.62, 126.86, 116.41, 112.64, 111.99, 110.83, 105.50, 62.66.

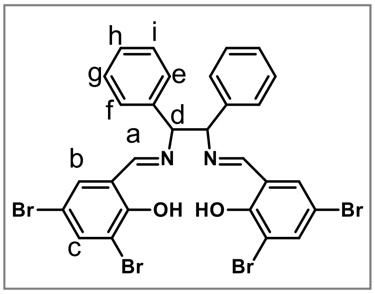

$A_{16}C_{11}B_1$. Followed the general procedure of $A_mB_1C_p$ (S2.4). The crude solid was washed with cold ethanol and methanol to afford the ligand as a light-yellow powder (46% yield). ^{1}H NMR (400 MHz, $CDCl_3$) δ 8.24 (s, 2H, a), 7.67 (s, 2H, c), 7.29-7.11 (m, 12H, b, e, f, g, h, i), 4.75 (s, 2H, d). ^{13}C NMR (125 MHz, $CDCl_3$) δ 164.63, 157.17, 138.17, 138.12, 133.32, 128.83, 128.32, 127.85, 120.05, 111.97, 110.45, 80.08.

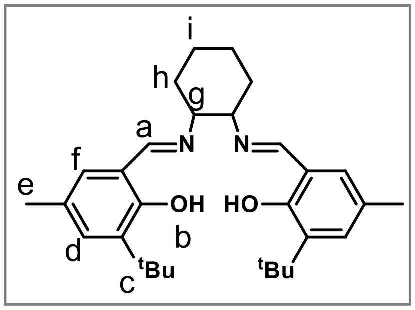

$A_{11}C_5B_1$ Followed the general procedure of $A_mB_1C_p$ (S2.4). The crude solid was washed with cold methanol and hexane to afford the ligand as a yellow-green powder (63% yield). ^{1}H NMR (400 MHz, $CDCl_3$) δ 13.62 (s, 2H, b), 8.23 (s, 2H, a), 7.04 (s, 2H, f), 6.78 (s, 2H, d), 3.29 (d, 2H, g), 2.19 (s, 6H, e), 1.98-1.68 (m, 8H, h, i), 1.39 (s, 18H, c). ^{13}C NMR (125 MHz, $CDCl_3$) δ 165.66, 158.10, 136.86, 130.36, 129.85, 126.55, 118.43, 72.55, 34.78, 33.25, 29.51, 24.47, 20.68.

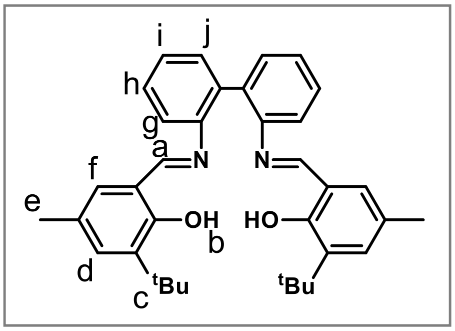

$A_{11}C_8B_1$. Followed the general procedure of $A_mB_1C_p$ (S2.4). The crude solid was washed with methanol and hexane to afford the ligand as an orange yellow powder (60% yield). ^{1}H NMR (400 MHz, $CDCl_3$) δ 12.89 (s, 2H, b), 8.24 (s, 2H, a), 7.44-7.34 (m, 6H, g, h, j), 7.12-7.02 (m, 4H, f, i), 6.49 (s, 2H, d), 2.15 (s, 6H, e), 1.35(s, 18H, c). ^{13}C NMR (125 MHz, $CDCl_3$) δ 163.92, 157.96, 148.08, 136.92, 134.68, 131.02, 130.87, 130.43, 129.17, 126.60, 119.15, 118.28, 34.80, 29.44, 20.63.

<u>Round 2 ligands in Table S6</u>

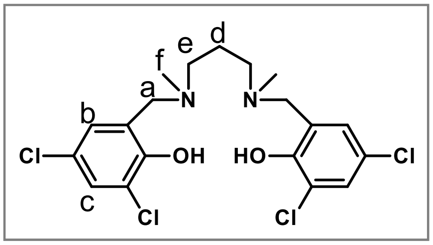

$A_6C_2B_2$ Followed the general route 2 procedures in $A_mB_2C_p$ (S2.5). The product was purified by flash chromatography on silica gel using DCM/methanol (20/1) to afford the ligand as a light-yellow solid (35% yield). ^{1}H NMR (400 MHz, $CDCl_3$) δ 7.27 (s, 2H, c), 6.87 (s, 2H, b), 3.69 (s, 4H, a), 2.54 (t, 4H, e), 2.30 (s, 6H, f), 1.84 (m, 2H, d). ^{13}C NMR (125 MHz, $CDCl_3$) δ 152.76, 128.88, 126.74, 123.76, 123.62, 121.62, 61.09, 54.78, 41.37, 24.65.

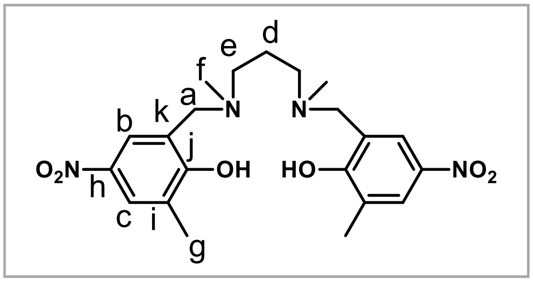

$A_5C_2B_2$ Followed the general route 2 procedures in $A_mB_2C_p$ (S2.5). The product was washed with cold methanol to afford the ligand as a yellow powder (43% yield). ^{1}H NMR (400 MHz, $CDCl_3$) δ 7.97 (s, 2H, b), 7.79 (s, 2H, c), 3.77 (s, 4H, a), 2.58, (t, 4H, e), 2.33 (s, 6H, f), 2.22 (s, 6H, g), 1.88 (m, 2H, d). ^{13}C NMR (125 MHz, $CDCl_3$) δ 162.83, 139.69, 126.25, 125.99, 122.45, 120.52, 60.97, 54.53, 41.35, 24.43, 15.77.

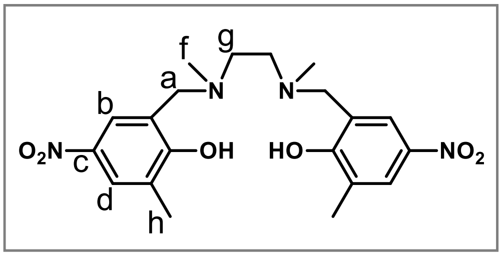

$A_5C_1B_2$ Followed the general route 1 procedures in $A_mB_2C_p$ (S2.5). The crude solid was washed with cold methanol to afford the ligand as a yellow powder (55% yield). ^{1}H NMR

(400 MHz, $CDCl_3$) δ 8.00 (s, 2H, b), 7.79 (s, 2H, d), 3.78 (s, 4H, a), 2.72 (s, 4H, g), 2.33 (s, 6H, h), 2.25 (s, 6H, f). ^{13}C NMR (125 MHz, $CDCl_3$) δ 162.58, 139.81, 126.47, 126.17, 122.59, 120.50, 61.31, 53.71, 41.68, 15.84.

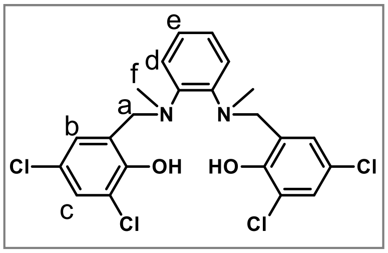

$A_6C_6B_2$. Followed the general route 2 procedures in $A_mB_2C_p$ (S2.5). The crude solid was washed with cold methanol and hexane to afford the ligand as a brown wax (50% yield). ^{1}H NMR (400 MHz, $CDCl_3$) δ 7.34-7.17 (m, 6H, c, b, e), 6.99-6.85 (d, 2H, d), 3.98 (s, 4H, a), 2.68 (s, 6H, f). ^{13}C NMR (125 MHz, $CDCl_3$) δ 151.16, 151.05, 150.90, 147.40, 128.85, 128.76, 126.50, 125.02, 123.88, 122.61, 121.92, 77.30, 77.05, 76.79, 59.56, 42.14.

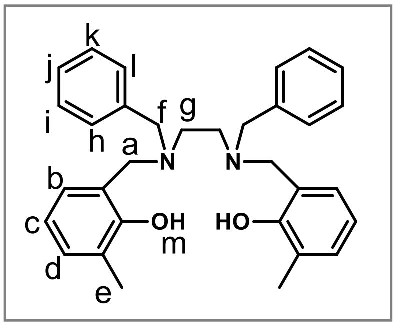

$A_2C_1B_3$. Followed the general route 1 procedures in $A_mB_3C_p$ (S2.5). The product was purified by flash chromatography on silica gel using DCM/hexane (1/5) and washed with cold methanol to afford the ligand as a white powder (47% yield). ^{1}H NMR (400 MHz, $CDCl_3$) δ 10.58 (s, 2H, m), 7.33-7.27 (m, 4H, h, l), 7.21-7.06 (m, 6H, i, k, j), 7.02 (dt, 2H, b), 6.79-6.62 (m, 4H, c, d), 3.65 (s, 4H, a), 3.52 (s, 4H, f), 2.69 (s, 4H, g), 2.20 (s, 6H, e). ^{13}C NMR (125 MHz, $CDCl_3$) δ 155.69, 136.40, 130.26, 129.59, 128.78, 128.49, 128.28, 127.86, 126.53, 125.10, 120.99, 119.03, 77.41, 77.16, 76.91, 66.01, 58.33, 49.80, 15.88, 15.83.

Round 3 ligands in Table S7

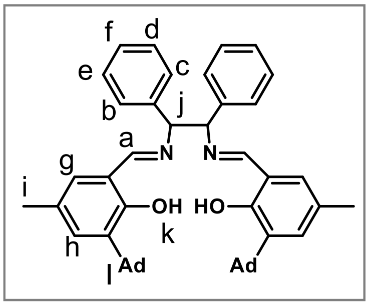

$A_8C_{11}B_1$ Followed the general route procedures in $A_mB_1C_p$ (S2.4). The crude solid was repetitively washed with cold methanol to afford the ligand as a yellow powder (35% yield). ^{1}H NMR (400 MHz, $CDCl_3$) δ 13.46 (s, 2H, k), 8.30 (s, 2H, a), 7.25-7.15 (m, 10H, b, c, d, e, f), 6.99 (d, 2H, g), 6.76 (d, 2H, h), 4.69 (s, 2H, j), 2.18 (s, 6H, i), 2.15 (m, 12H, l), 2.09 (m, 6H, l), 1.88-1.78 (m, 12H, l). ^{13}C NMR (125 MHz, $CDCl_3$) δ 167.29, 158.34, 139.71, 137.17, 130.76, 130.04, 128.76, 128.41, 128.25, 127.57, 126.74, 118.44, 80.24, 77.41, 77.16, 76.91, 41.05, 40.39, 37.33, 37.13, 37.00, 36.75, 29.27, 29.13, 20.73.

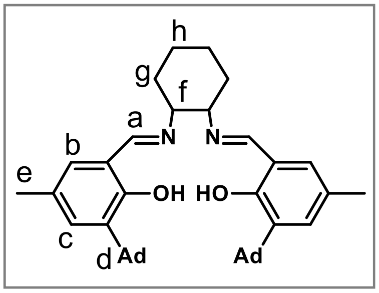

$A_8C_5B_1$ Followed the general procedure of $A_mB_1C_p$ (S2.4). The crude solid was washed with methanol to afford the ligand as a light-yellow powder (55% yield). ^{1}H NMR (400 MHz, toluene-d_8) δ 7.86 (s, 2H, a), 7.00 (m, 2H, c), 6.57 (s, 2H, b), 2.85 (m, 2H, f), 2.12 (s, 6H, e), 2.3-1.2 (m, 38H, d, g, h). ^{13}C NMR (125 MHz, toluene-d_8) δ 166.26, 158.91, 137.44, 130.66, 129.98, 129.02, 126.51, 118.73, 72.35, 41.07, 40.65, 37.61, 37.34, 37.21, 37.00, 33.04, 32.87, 29.69, 29.53, 24.48.

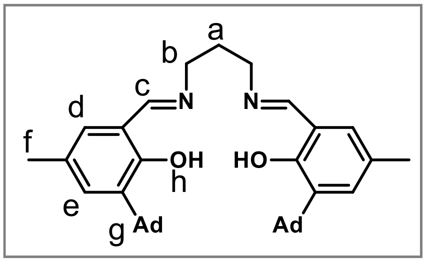

$A_8C_2B_1$. Followed the general procedure of $A_mB_1C_p$ (S2.4). The crude solid was washed with methanol to afford the ligand as a yellow powder (yield: 45%). ^{1}H NMR (400 MHz, toluene-d_8) δ 13.83 (s, 2H, h), 8.33 (s, 2H, c), 7.08 (d, 2H, d), 6.90 (d, 2H, e), 3.80-3.61 (m, 4H, b), 2.29 (s, 6H, f), 2.19 (d, 12H, g), 2.10 (d, 8H, a, g), 1.81 (m, 12H, g). ^{13}C NMR (125 MHz, $CDCl_3$) δ 166.38, 165.92, 158.56, 137.54, 130.55, 129.49, 126.79, 118.45, 77.41, 77.16, 76.91, 56.99, 41.04, 40.64, 40.41, 37.31, 37.13, 37.06, 31.77, 29.23, 29.13, 20.84.

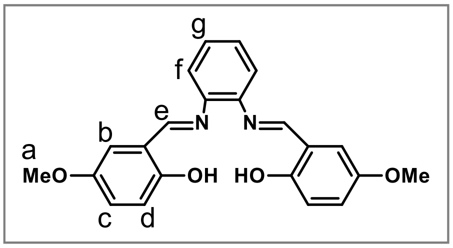

$A_4C_6B_1$ Followed the general procedure of $A_mB_1C_p$ (S2.4). The crude solid was washed with cold methanol to afford the ligand as an orange-yellow powder (54% yield). ^{1}H NMR (400 MHz, DMSO-d_6) δ 7.64 (s, 2H, e), 7.31-7.25 (m, 6H, b, c, d), 7.00-6.94 (m, 4H, f, g), 3.80 (s, 6H, a). ^{13}C NMR (125 MHz, DMSO-d_6) δ 152.17, 152.13, 151.74, 122.79, 118.91, 118.17, 112.45, 109.93, 55.82.

<u>Round 3 ligands in Table S8</u>

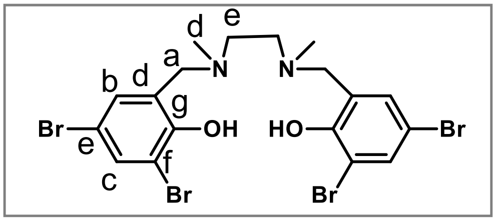

$A_{16}C_1B_2$. Followed the general route 1 procedures in $A_mB_2C_p$ (S2.5). The crude solid was washed with cold methanol to afford the ligand as an off-white powder (yield 60%). ^{1}H NMR (400 MHz, $CDCl_3$) δ 7.57 (s, 2H, c), 7.04 (s, 2H, b), 3.69 (s, 4H, a), 2.70 (s, 4H, e). 2.31 (s, 6H, d). ^{13}C NMR (125 MHz, $CDCl_3$) δ 154.04, 134.54, 130.37, 124.00, 111.23, 110.96, 61.21, 54.11, 41.89.

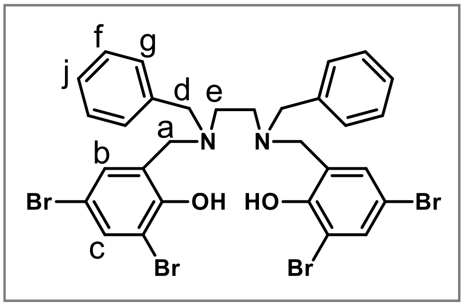

$A_{16}C_1B_3$. Followed the general route 1 procedures in $A_mB_2C_p$ (S2.5). The crude solid was washed with cold methanol to afford the ligand as an off-white powder (52% yield). ^{1}H NMR (400 MHz, $CDCl_3$) δ 7.55 (s, 2H, c), 7.35-7.10 (m, 10H, f, g, b), 6.97 (s, 2H, j), 3.63 (s, 4H, a), 3.53 (s, 4H, d), 2.62 (s, 4H, e). ^{13}C NMR (125 MHz, $CDCl_3$) δ 153.77, 135.28, 134.58, 130.48, 129.53, 129.08, 128.40, 124.17, 111.17, 111.12, 59.08, 58.11, 50.19.

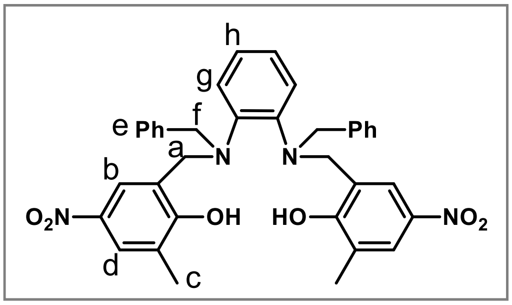

$A_5C_6B_3$. Followed the general route 2 procedures in $A_mB_2C_p$ (S2.5). The crude solid was washed with cold methanol and hexane to afford the ligand as a light-yellow wax (60% yield). ^{1}H NMR (400 MHz, $CDCl_3$) δ 7.98 (m, 2H, b), 7.90-7.69 (m, 2H, d), 7.35-7.27 (m, 4H, e), 7.25 (m, 6H, e), 6.93 (m, 2H, g), 6.61 (m, 2H, h), 4.01 (d, 8H, a, f), 2.25 (s, 6H, c). ^{13}C NMR (125 MHz, $CDCl_3$) δ 162.08, 144.40, 139.59, 137.95, 130.11, 129.34, 128.55, 127.65, 127.40, 123.47, 122.10, 121.70, 121.60, 117.82, 115.10, 112.28, 77.41, 77.16, 76.91, 59.93, 58.59, 15.92.

S2.6 Synthesis of Aluminum complexes

The Al complexes were prepared according to the literature. In brief, to a stirred solution of the ligand (1 mmol) in toluene (5 mL) was added $AlMe_3$ (0.5 mL of a 2.0 M solution in toluene, 1 mmol). The reaction mixture was stirred at 110 ºC overnight. The solvent was evaporated to dryness to leave a white solid. The solid was washed by hexane and ether to the unreacted ligand and $AlMe_3$, and the overall yields for Al complexes were around 50-70%. Note that some complexes had low solubility and formed aggregation in the solution, leading to broad peaks in NMR spectra, which was also found in the literature.[13, 14] We also attempted the synthesis of Al complexes using $Al(O^iPr)_3$ or by reaction of the ligand-AlMe complex with alkoxide but many of the obtained products had more severe aggregation problems based on NMR spectra. Al complexes having ligands including $A_6C_6B_2$, $A_8C_{11}B_1$, $A_8C_2B_1$, $A_4C_6B_1$, $A_5C_6B_3$ had low solubility at room temperature, and we acquired their ^1H NMR spectra at 85 °C. It is also difficult to acquire ^{13}C NMR spectra for the Al complexes having low solubilities as the compound would precipitate when leaving for ^{13}C NMR acquisition over the time.

Nevertheless, the successful synthesis of Al complexes could also be confirmed by ESI-MS. We note that in our ESI-MS measurements, Al complexes constantly lose labile CH_3 regardless of the ionization modes (positive or negative) or intensities. However, the existence of Al-CH_3 group in all Al complexes could be confirmed in NMR spectra.

<u>Round 1 catalysts in Table S3:</u>

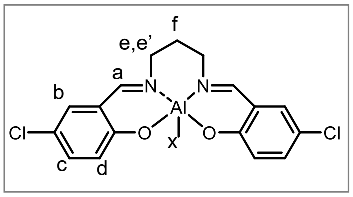

($A_7C_2B_1$)AlMe. Followed the procedure in S2.6. The crude solid was washed by hexane to afford the product as a yellow solid. ^1H NMR (400 MHz, $CDCl_3$) δ 8.10 (s, 2H, a), 7.27-6.91 (m, 6H, b, c, d), 4.08, 3.67 (m, 4H, e, e'), 2.04 (m, 2H, f), -0.77 (m, 3H, x). ^{13}C NMR was not acquired due to the low solubility of the complex. ^1H NMR spectra suggested the aggregation status of the complex in solution. ESI-MS. Exact mass calculated for $[M-CH_3]^+$ ($C_{17}H_{14}AlCl_2N_2O_2$): 375.0248, found: 375.0253.

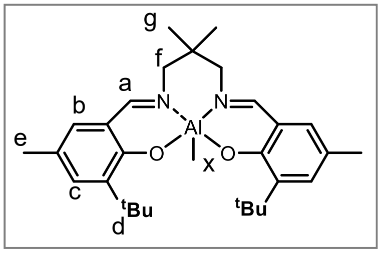

($A_{11}C_3B_1$)AlMe. Followed the procedure in S2.6. The crude solid was washed by hexane to afford the product as a yellow solid. ^{1}H NMR (400 MHz, $CDCl_3$) δ 8.05 (s, 2H, a), 7.23 (d, 2H, b), 6.89 (d, 2H, c), 3.33 (q, 4H, f), 2.27 (s, 6H, e), 1.48 (s, 18H, d), 1.13, 1.03 (s, 6H, g), -1.09 (s, 3H, x). ^{13}C NMR (125 MHz, $CDCl_3$) δ 169.73, 163.35, 141.62, 133.81, 130.79, 124.22, 119.08, 77.41, 77.16, 76.91, 67.30, 36.50, 35.38, 29.69, 26.09, 25.64, 20.71. ESI-MS. Exact mass calculated for $[M-CH_3]^+$ ($C_{29}H_{40}AlN_2O_2$): 475.2905, found: 475.2912.

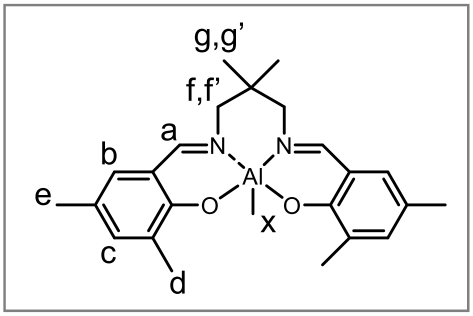

($A_{14}C_3B_1$)AlMe. Followed the procedure in S2.6. The crude solid was washed by hexane to afford the product as a yellow solid. ^{1}H NMR (400 MHz, $CDCl_3$) δ 7.94 (s, 2H, a), 7.06 (d, 2H, b), 6.77 (d, 2H, c), 4.12 ,3.15 (d, 4H, f, f'), 2.18 (d, 12H, d, e), 1.12, 0.88 (s, 6H, g, g'), -0.71 (s, 3H, x). ^{13}C NMR (125 MHz, $CDCl_3$) δ 168.49, 162.84, 137.01, 130.44, 129.56, 124.45, 117.63, 71.81, 36.48, 26.19, 22.38, 20.38, 16.21.

ESI-MS. Exact mass calculated for $[M-CH_3]^+$ ($C_{23}H_{28}AlN_2O_2$): 391.1966, found: 391.1961.

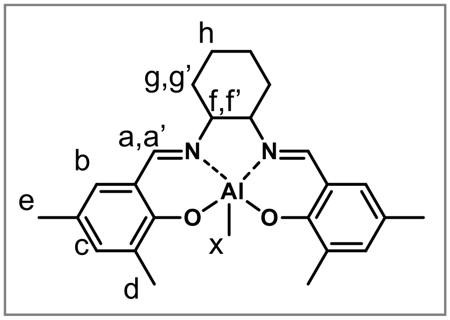

$(A_{14}C_5B_1)AlMe$. Followed the procedure in S2.6. The crude solid was washed by hexane to afford the product as a yellow solid. ^1H NMR (400 MHz, $CDCl_3$) δ 8.20 8.03 (d, 2H, a,a' enantiomer), 7.12 (s, 2H, b), 6.86 (d, 2H, c), 3.59, 3.03 (t, 2H, f,f' enantiomer), 2.31(d, 6H, e, enantiomer), 2.23 (d, 6H, d, enantiomer), 2.55-2.05 (m, 4H, g, g'), 1.45 (m, 4H, h), -1.15 (s, 3H, x). ^{13}C NMR (125 MHz, $CDCl_3$) δ 166.72, 163.41, 162.08, 161.65, 137.50, 136.90, 130.71, 130.41, 130.15, 124.60, 124.07, 117.68, 117.50, 65.83, 62.53, 31.39, 31.10, 29.86, 29.01, 27.18, 24.38, 24.00, 20.43, 16.13.

ESI-MS. Exact mass calculated for $[M-CH_3]^+$ ($C_{24}H_{28}AlN_2O_2$): 403.1966, found: 403.1976.

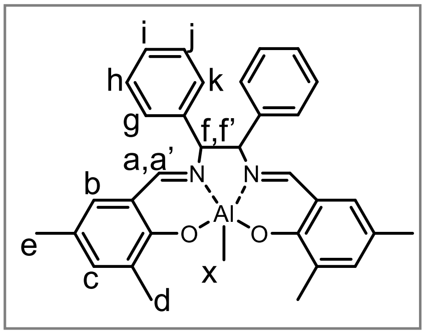

$(A_{14}C_{11}B_1)AlMe$. Followed the procedure in S2.6. The crude solid was washed by hexane to afford the product as a yellow solid. ^1H NMR (400 MHz, $CDCl_3$) δ 7.95, 7.67 (s, 2H, a, a' enantiomer), 7.39-7.01 (m, 12H, b, g, h, i, j, k), 6.57 (d, 2H, c), 5.18, 4.77 (q, 2H, f, f' enantiomer), 3.03 (t, 6H, e), 2.31(s, 6H, d), -0.85 (s, 3H, x). ^{13}C NMR (125 MHz, $CDCl_3$) δ 166.72, 163.41, 162.08, 161.65, 137.50, 136.90, 130.71, 130.41, 130.15, 124.60, 124.07, 117.68, 117.50, 65.83, 62.53, 31.39, 31.10, 29.86, 29.01, 27.18, 24.38, 24.00, 20.43, 16.13.

ESI-MS. Exact mass calculated for $[M-CH_3]^+$ ($C_{32}H_{30}AlN_2O_2$): 501.2123, found: 501.2133; exact mass calculated for $[M+H]^+$ ($C_{33}H_{34}AlN_2O_2$): 517.2436, found 517.2436.

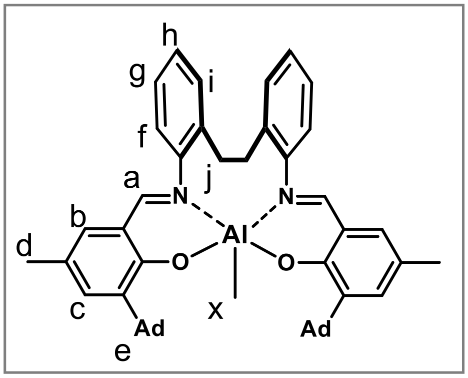

($A_8C_9B_1$)AlMe. Followed the procedure in S2.6. The crude solid was washed by hexane to afford the product as a yellow solid. ^1H NMR (400 MHz, $CDCl_3$) δ 8.45-6.15 (br, 14H, a, b, c, f, g, h, i), 3.52-2.73 (br, 4H, j), 2.4 – 1.25 (m, br, 36H, d, e), -0.62 – -0.85 (s, 3H, x). ^{13}C NMR was not acquired due to the low solubility of the complex. ESI-MS. Exact mass calculated for $[M-CH_3]^+$ ($C_{50}H_{54}AlN_2O_2$): 741.4001, found: 741.4003; exact mass calculated for $[M+H]^+$ ($C_{51}H_{58}AlN_2O_2$): 757.4314, found 757.3952.

<u>Round 1 catalysts in Table S4:</u>

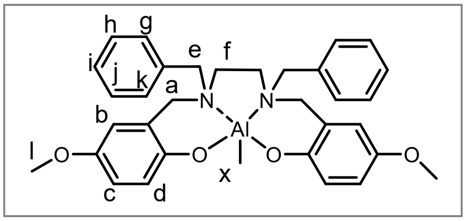

($A_4C_1B_3$)AlMe. Followed the procedure in S2.6. The crude solid was washed by hexane to afford the product as an orange solid. ^1H NMR (400 MHz, $CDCl_3$) δ 7.37-7.24 (m, 10H, g, h, i, j, k), 6.82 (d, 4H, c, d), 6.50 (s, 2H, b), 3.77 (s, 14H, a, e, l), 2.34 (br s, 4H, f), -0.57 (s, 3H, x). ^{13}C NMR (400 MHz, $CDCl_3$) δ 138.03, 132.15, 129.18, 128.37, 125.44, 120.75, 55.93, 21.62, -3.93. NMR suggested the complex might have isomers or aggregations in solution. ESI-MS. Exact mass calculated for $[M-CH_3]^+$ ($C_{32}H_{34}AlN_2O_4$): 537.2334, found: 537.1756.

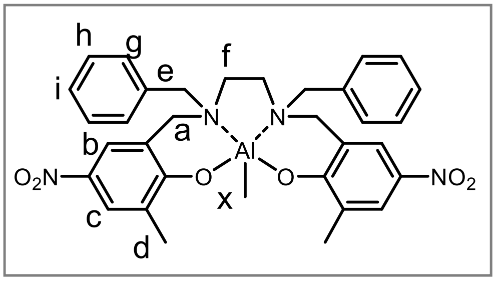

($A_5C_1B_3$)AlMe. Followed the procedure in S2.6. The crude solid was washed by hexane to afford the product as a yellow solid. ^{1}H NMR (400 MHz, toluene-d_8) δ 7.83 (s, 2H, b), 7.49 (s, 2H, c), 7.19-6.90 (m, 10H, g, h, i), 3.54 (m, 4H, a), 3.04 (d, 4H, e), 2.22 (m, 4H, f), 2.05 (s, 6H, d), 0.14 (s, 3H, $AlCH_3$). ^{13}C NMR was not acquired due to the low solubility of the complex.

ESI-MS. Exact mass calculated for $[M-H]^-$ ($C_{33}H_{34}AlN_4O_6$): 609.2299; found 609.3357.

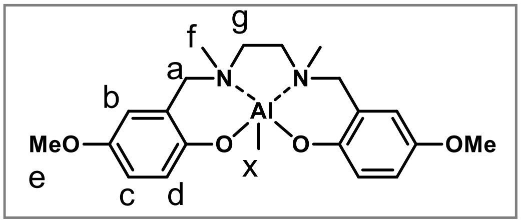

($A_4C_1B_2$)AlMe. Followed the procedure in S2.6. The crude solid was washed by hexane to afford the product as a white solid. ^{1}H NMR (400 MHz, $CDCl_3$) δ 6.93-6.41 (m, 6H, b, c, d), 4.32,3.17 (d, 4H, a), 3.73 (s, 6H, e), 3.45-2.62 (m, 4H, g), 2.32 (s, 6H, f), -0.84 (s, 3H, x). ^{13}C NMR (125 MHz, $CDCl_3$) δ 153.79, 150.80, 121.84, 121.15, 120.26, 114.88, 114.78, 61.65, 55.97, 55.12, 44.42.

ESI-MS. Exact mass calculated for $[M-CH_3]^+$ ($C_{20}H_{26}AlN_2O_4$): 385.1708, found: 385.1661.

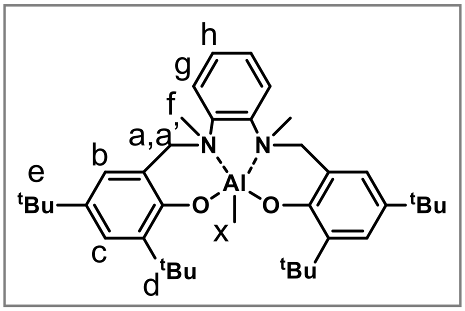

($A_3C_6B_2$)AlMe. Followed the procedure in S2.6. The crude solid was washed by hexane to afford the product as an off-white solid. ^{1}H NMR (400 MHz, $CDCl_3$) δ 7.83-7.44 (m, 4H, b, c), 7.37 (d, 2H, g), 6.85 (d, 2H, h), 4.49,3.86 (m, 4H, a, a'), 3.01(s, 6H, f), 1.55(s, 18H, e), 1.31(s, 18H, d), -0.90 (d, 3H, x). ^{13}C NMR (125 MHz, $CDCl_3$) δ 156.96, 145.92, 137.71, 137.30, 129.13, 124.27, 123.84, 123.30, 119.80, 77.41, 77.36, 77.16, 76.91, 46.63, 45.92, 35.19, 34.15, 31.98, 30.02, 29.79.

ESI-MS. Exact mass calculated for $[M-CH_3]^+$ ($C_{38}H_{54}AlN_2O_2$): 597.4001, found: 597.4006.

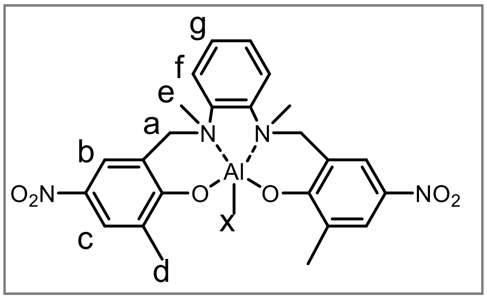

($A_5C_6B_2$)AlMe. Followed the procedure in S2.6. The crude solid was washed by hexane to afford the product as a yellow-pink solid. ^{1}H NMR (400 MHz, $CDCl_3$) δ 8.11-7.42 (m, 8H, b, c, f, g), 4.75-3.95 (m, 4H, a), 2.76 (s, 6H, e), 2.19 (s, 6H, d), 0.06, -0.43, -0.88 (s, 3H, x). ^{13}C NMR (125 MHz, $CDCl_3$) δ 165.11, 143.62, 137.45, 130.25, 129.19, 128.92, 128.38, 127.22, 125.44, 123.97, 123.20, 119.17, 62.70, 47.78, 31.12, 21.62, 16.34, 16.30, 15.50, -3.94. NMR suggested isomer structures in $CDCl_3$.

ESI-MS. Exact mass calculated for $[M-CH_3]^+$ ($C_{24}H_{24}AlN_4O_6$): 491.1511, found: 491.1513.

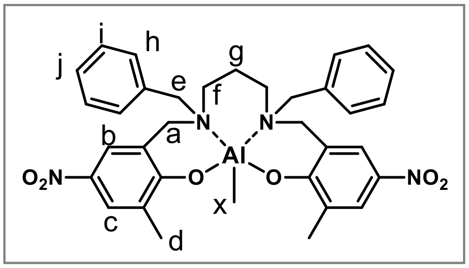

($A_5C_2B_3$)AlMe. Followed the procedure in S2.6. The crude solid was washed by hexane to afford the product as a yellow solid. ^{1}H NMR (600 MHz, toluene-d_8, 85 °C) δ 7.86 (s, 2H, b), 7.59 (s, 2H, c), 7.12-6.95 (m, 10H, h, i, j), 3.01, 3.08 (s, 8H, a, e), 2.10-2.00 (m, 10H,

d, f), 1.62 (s, 3H, x), 1.35 (m, 2H, g). ^{13}C NMR was not acquired due to the low solubility of the complex.

ESI-MS. Exact mass calculated for $[M-CH_3]^-$ ($C_{33}H_{33}AlN_4O_6$): 609.2294, found: 609.1463.

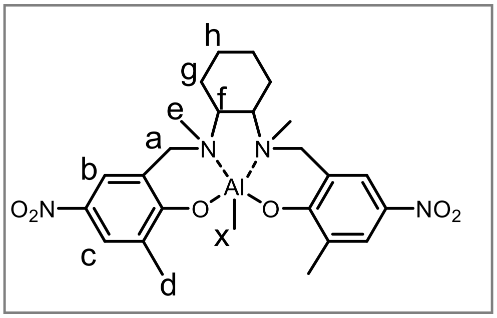

($A_5C_5B_2$)AlMe. Followed the procedure in S2.6. The crude solid was washed by hexane to afford the product as a yellow solid. ^{1}H NMR (400 MHz, $CDCl_3$) δ 8.05(m, 2H, b), 7.82(q, 2H, c), 3.94 (m, 4H, a, enantiomers), 2.94 (m, 2H, f), 2.31-2.27 (m, 12H, d, e), 1.65-1.38 (m, 4H, g, h), -0.71,-0.78 (s, 3H, x, enantiomers). ^{13}C NMR was not acquired due to the low solubility of the complex.

ESI-MS. Exact mass calculated for $[M-CH_3]^+$ ($C_{24}H_{30}AlN_4O_6$): 497.1981, found: 497.1977.

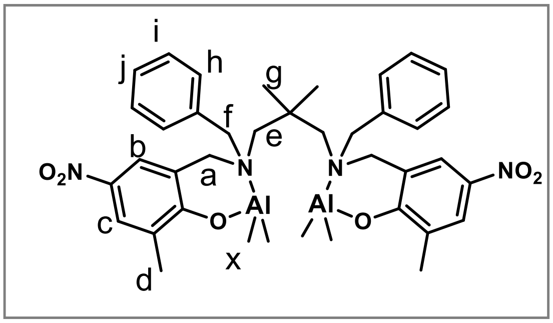

($A_5C_3B_3$)Al_2Me_4: Followed the procedure in S2.6 and used 2 equiv. $AlMe_3$ (1 equiv. gave mixture). The crude solid was washed by hexane to afford the product as a brown solid. ^{1}H NMR (400 MHz, $CDCl_3$) δ 8.07-7.97 (d, 2H, b), 7.81 (d, 2H, c), 7.42-7.16 (m, 10H, h, i, j), 3.78 (d, 4H, a), 3.47 (d, 4H, f), 2.33 (s, 4H, e), 2.30-2.15 (m, 6H, d), 1.28 (m, 12H, x), 0.80 (s, 6H, g). ^{13}C NMR was not acquired due to the low solubility of the complex.

ESI-MS. Exact mass calculated for $[M+Na]^+$ ($C_{39}H_{50}Al_2N_4O_6Na$) 747.3253; found 747.3569

Round 2 catalysts in Table S5:

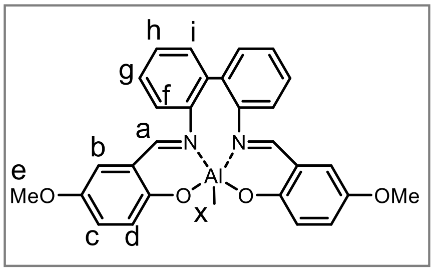

$(A_4C_8B_1)AlMe$. Followed the procedure in S2.6. The crude solid was washed by hexane to afford the product as a yellow solid. ^1H NMR (400 MHz, $CDCl_3$) δ 8.00 (d, 2H, a, enantiomer), 7.47-6.95 (m, 12H, c, d, f, g, h, i), 6.58 (s, 2H, b), 3.69 (d, 6H, e), -1.11 (s, 3H, x). ^{13}C NMR (125 MHz, $CDCl_3$) δ 172.68, 168.60, 163.41, 159.63, 150.84, 150.19, 147.98, 147.72, 137.99, 132.98, 132.85, 130.63, 129.40, 129.38, 129.15, 128.34, 128.09, 127.66, 126.62, 125.41, 124.80, 124.08, 123.89, 118.59, 118.09, 114.99, 112.97, 77.37, 77.16, 76.95, 55.96, 55.81, 21.59.

ESI-MS. Exact mass calculated for $[M-CH_3]^+$ ($C_{28}H_{22}AlN_2O_4$): 477.1395, found: 477.1386.

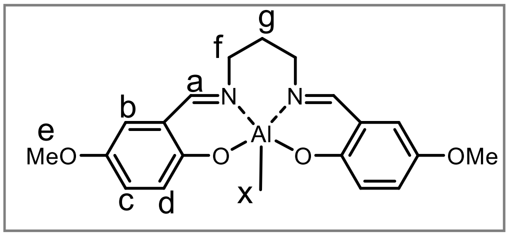

$(A_4C_2B_1)AlMe$. Followed the procedure in S2.6. The crude solid was washed by hexane and cold toluene to afford the product as a yellow solid. ^1H NMR (400 MHz, $CDCl_3$) δ 8.13 (s, 2H, a), 7.02-6.61 (m, 6H, b, c, d), 3.74 (s, 6H, e), 3.72 (br, 4H, f), 2.36 (s, 2H, g), -0.78 (s, 3H, x). ^{13}C NMR (125 MHz, $CDCl_3$) δ 168.53, 160.92, 150.28, 124.56, 123.40, 117.79, 114.07, 58.28, 56.09, 31.64, 29.54.

ESI-MS. Exact mass calculated for $[M-CH_3]^+$ ($C_{19}H_{20}AlN_2O_4$): 367.1238, found: 367.1216.

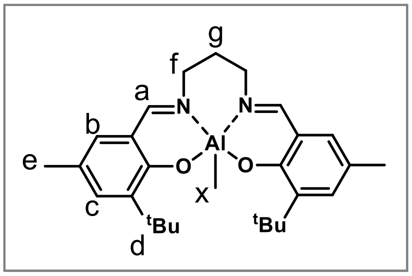

($A_{11}C_2B_1$)AlMe. Followed the procedure in S2.6. The crude solid was washed by hexane to afford a yellow-green solid. ^{1}H NMR (400 MHz, toluene-d_8) δ 7.37 (s, 2H, a), 7.33 (d, 2H, c), 6.57 (d, 2H, b), 3.09, 2.79 (m, 4H, f), 2.30, 2.11 (s, 2H, g), 2.23 (m, 6H, e), 1.72 (s, 18H, d), -0.50 (s, 3H, x). ^{13}C NMR (125 MHz, toluene-d_8) δ 169.77, 163.99, 141.54, 137.44, 134.00, 131.10, 123.81, 119.29, 54.95, 35.58, 35.45, 30.10, 27.32.

ESI-MS. Exact mass calculated for $[M\text{-}CH_3]^+$ ($C_{27}H_{36}AlN_2O_2$): 447.2592, found: 447.2593.

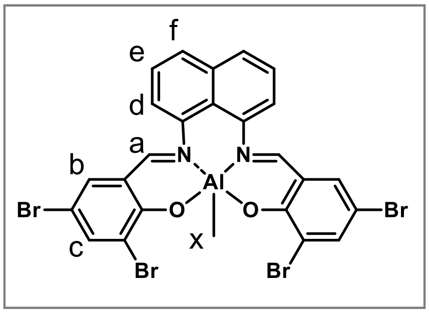

($A_{16}C_7B_1$)AlMe. Followed the procedure in S2.6. The crude solid was washed by hexane and toluene. ^{1}H NMR (600 MHz, toluene-d_8, at 85 °C) δ 8.79 (s, 2H, a), 7.59 – 6.66 (m, 8H, b, d, e, f), 6.16 (s, 2H, c), 0.40 (s, 3H, x).). ^{13}C NMR was not acquired due to the low solubility of the complex.

ESI-MS. Exact mass calculated for $[M\text{-}CH_3]^+$ ($C_{24}H_{12}AlBr_4N_2O_2$): 702.7448, found: 702.2150.

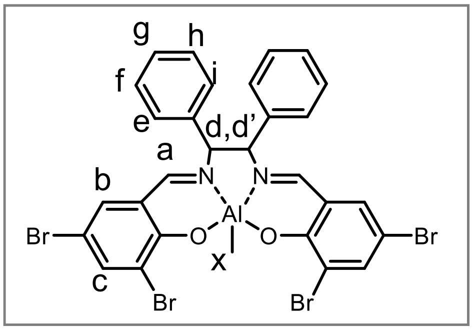

($A_{16}C_{11}B_1$)AlMe. Followed the procedure in S2.6. The crude solid was washed by hexane and cold toluene to afford the product as a light-yellow solid. ^{1}H NMR (400 MHz, toluene-*d*8) δ 7.67 (d, 2H, a), 7.47-6.84 (m, 14H, b, c, e, f, g, h, i), 5.05,4.06 (d, 2H, d, d' enantiomer), -0.29 (s, 3H, x). ^{13}C NMR was not acquired due to the low solubility of the complex.
ESI-MS. Exact mass calculated for $[M-CH_3]^+$ ($C_{28}H_{18}AlBr_4N_2O_2$): 760.7876, found: 760.7877.

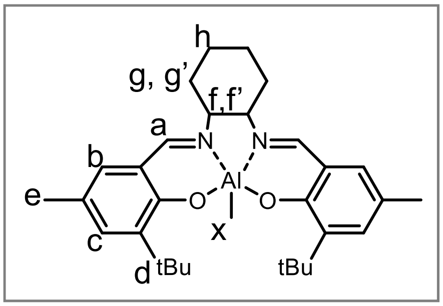

($A_{11}C_5B_1$)AlMe. Followed the procedure in S2.6. The crude solid was washed by hexane to afford the product as a yellow-green solid. ^{1}H NMR (400 MHz, $CDCl_3$) δ 8.21 (dd, 2H, a), 7.22 (t, 2H, b), 6.86 (dd, 2H, c), 3.49,3.08 (t, 2H, f, f' enantiomer), 2.51,2.05 (m, 4H, g, g' enantiomer), 2.24 (d, 6H, e, enantiomer), 1.49 (d, 18H, d, enantiomer), 1.42 (m, 4H, h), -1.18 (s, 3H, x). ^{13}C NMR (125 MHz, $CDCl_3$) δ 167.23, 164.14, 162.65, 162.03, 141.65, 141.64, 134.16, 133.34, 131.44, 131.12, 124.39, 123.92, 119.13, 119.07, 77.37, 77.16, 76.95, 65.66, 62.58, 35.50, 35.37, 29.84, 29.80, 28.81, 27.32, 24.33, 23.90, 20.69.
ESI-MS. Exact mass calculated for $[M-CH_3]^+$ ($C_{30}H_{40}AlN_2O_2$): 487.2905, found: 487.2906.

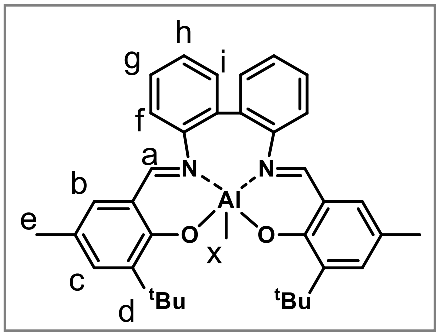

($A_{11}C_8B_1$)AlMe. Followed the procedure in S2.6. The crude solid was washed by hexane and diethyl ether to afford the product with a yellow solid. ^{1}H NMR (400 MHz, toluene-*d*8) δ 7.51 (d, 2H, a, enantiomer), 7.37-6.21 (m, 12H, i, g, f, h, b, c), 2.07 (m, 18H, d), 1.75

(m, 6H, e, enantiomer), -0.64 (s, 3H, x). ^{13}C NMR (125 MHz, toluene-d_8) δ (125 MHz, Toluene-d_8) 169.44, 166.19, 149.01, 131.88, 130.83, 128.53, 128.48, 127.59, 126.49, 124.73, 119.59, 118.40, 35.79, 34.95, 32.04, 30.94, 29.86, 11.63.

ESI-MS. Exact mass calculated for $[M-CH_3]^+$ ($C_{36}H_{38}AlN_2O_2$): 557.2749, found: 557.2763.

Round 2 catalysts in Table S6:

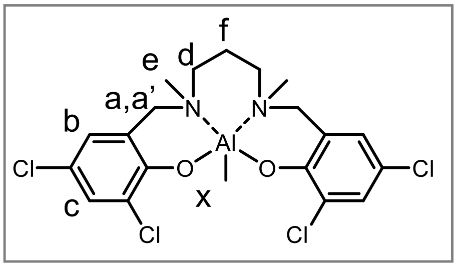

($A_6C_2B_2$)AlMe. Followed the procedure in S2.6. The crude solid was washed by hexane to afford a brown solid. ^1H NMR (400 MHz, THF-d_8) δ 7.32-6.84 (m, 4H, b, c), 5.41-4.20 (m, 4H, a, a'), 3.81-2.50 (m, 4H, d), 2.29 (d, 6H, e), 2.17-1.88 (m, 2H, f), 0.21 (s, 3H, x). ^{13}C NMR (125 MHz, THF-d_8) δ 153.22, 128.06, 126.62, 124.86, 122.64, 121.04, 60.14, 54.47, 40.30, 34.08, 22.23.

ESI-MS. Exact mass calculated for $[M+Na]^+$ ($C_{20}H_{23}AlCl_4N_2O_2Na$): 513.0221, found: 513.2747.

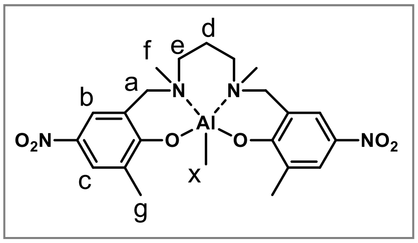

($A_5C_2B_2$)AlMe. Followed the procedure in S2.6. The crude solid was washed by hexane to afford the product as an orange solid. ^1H NMR (400 MHz, THF-d_8): 7.23-7.03 (m, 4H, b, c), 3.62 (m, 4H, a), 2.73-1.94 (m br,16H, e, f, g), 1.76 (m, 2H, d), 0.19, -0.81 (br, 3H, x). The complex formed aggregation in the solution. ^{13}C NMR was not acquired due to the low solubility of the complex.

ESI-MS. Exact mass calculated for $[M-CH_3]^+$ ($C_{21}H_{26}AlN_4O_6$): 457.1668, found: 457.2748.

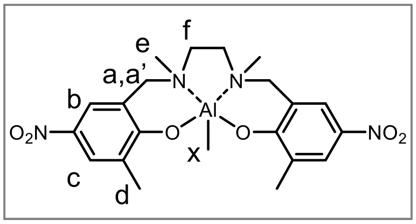

$(A_5C_1B_2)AlMe$. Followed the procedure in S2.6. The crude solid was washed by hexane to afford the product as a yellow solid. ^1H NMR (400 MHz, THF-d_8) δ 8.01 (d, 2H, b), 7.86 (d, 2H, c), 4.36, 3.54 (dd, 4H, a, a'), 3.12-2.60 (m, 4H, f), 2.38-2.17 (m, 12H, d, e), -0.76 (s, 3H, x). ^{13}C NMR (125 MHz, THF-d_8) δ 163.59, 135.59, 126.82, 126.06, 123.20, 118.31, 58.39, 47.57, 44.68, 18.64, 11.53.

ESI-MS. Exact mass calculated for $[M-CH_3]^+$ ($C_{20}H_{24}AlN_4O_6$): 443.1511, found: 443.1473.

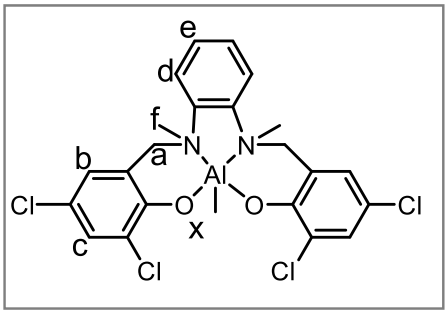

$(A_6C_6B_2)AlMe$. The crude solid was washed by hexane to afford the product as a yellow-pink solid. ^1H NMR (600 MHz, toluene-d_8, 85 °C) δ 7.45-6.28 (m, 8H, c, b, d, e), 4.29-3.42 (m, 4H, a), 3.24-2.23 (m, 6H, f), 0.14, -0.28,-0.85 (s, 3H, x). The complex has multiple isomers or aggregation states in the solution.

ESI-MS. Exact mass calculated for $[M-CH_3]^+$ ($C_{23}H_{21}AlCl_4N_2O_2$): 510.9908, found: 510.9904.

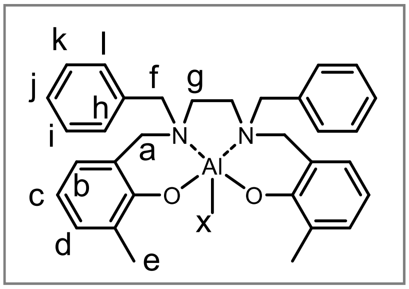

($A_2C_1B_3$)AlMe. Followed the procedure in S2.6. The crude solid was washed by hexane, to afford the product as a white solid. ^{1}H NMR (400 MHz, toluene-d_8) δ 7.33-6.95 (m, 12H, h, i, j, k, l), 6.76-6.67 (m, 6H, b, c, d), 3.59 (s, 8H, a, f), 2.60-2.32 (m, 10H, g, e), -0.26 (s, 3H, x). ^{13}C NMR was not acquired due to the low solubility of the complex.
ESI-MS. Exact mass calculated for $[M-CH_3]^+$ ($C_{32}H_{34}AlN_2O_2$): 505.2436, found: 505.2852.

<u>Round 3 catalysts in Table S7:</u>

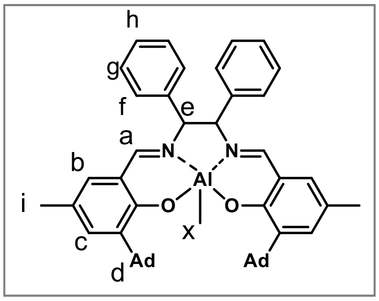

($A_8C_{11}B_1$)AlMe. Followed the procedure in S2.6. The crude solid was washed by hexane, and diethyl ether to afford a yellow solid. ^{1}H NMR (600 MHz, toluene-d_8, 85 °C) δ 9.13 (s, 2H, a), 8.51-5.99 (m br, 14H, b, c, f, g, h), 4.24 (m, 2H, e), 2.74-1.22 (m br, 36H, d, i), -0.01, -1.21 (s, 3H, x). The complex has isomer and aggregation states in the solution.
ESI-MS. Exact mass calculated for $[M-CH_3]^+$ ($C_{50}H_{54}AlN_2O_2$): 741.4001, found: 741.3984; exact mass calculated for $[M+H]^+$ ($C_{51}H_{58}AlN_2O_2$): 757.4314; found: 757.3987.

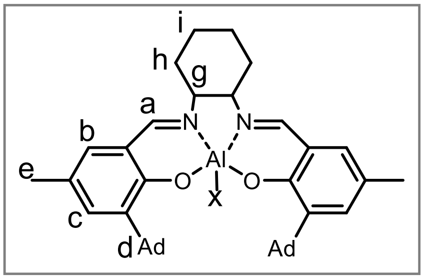

($A_8C_5B_1$)AlMe. Followed the procedure in S2.6. The crude solid was washed by hexane to afford a yellow solid. ^{1}H NMR (400 MHz, THF-d_8) δ 8.43, 7.01 (dd, 2H, a, enantiomer), 8.05 –6.57 (m, 4H, b, c, enantiomer), 4.04-3.75 (m, 2H, g, enantiomer), 2.37–0.52 (m, 44H, d, e, h, i), -1.01 (s, 3H, x). ^{13}C NMR (125 MHz, THF-d_8) δ 172.80, 172.32, 167.37, 167.31, 167.10, 163.15, 162.78, 159.97, 159.40, 159.14, 157.51, 156.74, 152.02, 142.56, 142.36, 142.07, 141.70, 138.10, 137.96, 136.32, 135.58, 133.46, 132.12, 131.88, 131.43, 131.33,

130.60, 129.00, 127.25, 126.63, 126.44, 126.04, 126.00, 125.83, 124.90, 124.48, 122.25, 122.01, 120.53, 119.93, 119.62, 73.35, 68.84, 68.50, 68.39, 64.33, 62.96, 42.22, 41.87, 41.42, 41.27, 41.24, 41.21, 41.15, 38.28, 38.26, 38.16, 38.13, 38.11, 38.09, 38.05, 38.02, 37.99, 37.92, 37.85, 37.71, 30.75, 30.73, 30.56, 30.49, 30.46, 30.36, 30.34, 30.33, 30.27, 26.56, 21.72, 20.88, 20.86, 20.83, 20.77, 20.68. The NMR spectra suggested that the complex has enantiomers and might have aggregation states in the solution. ESI-MS. Exact mass calculated for $[M-CH_3]^+$ ($C_{42}H_{52}AlN_2O_2$): 643.3844, found: 643.3830.

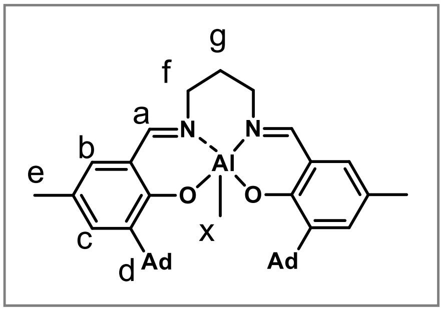

($A_8C_2B_1$)AlMe. Followed the procedure in S2.6. The crude solid was washed by hexane, and to a yellow solid. ^{1}H NMR (600 MHz, toluene-d_8, 85 °C) δ 8.37-7.78 (br, 2H, a), 7.38-6.26 (br, 4H, b,c), 4.88 (m, 4H, f), 2.46-1.04 (br m, 38H, d, e, g), 0.17 (s, 3H, x). The complex has aggregation states in the solution.

ESI-MS. Exact mass calculated for $[M-CH_3]^+$ ($C_{39}H_{48}AlN_2O_2$): 603.3531, found: 603.3536.

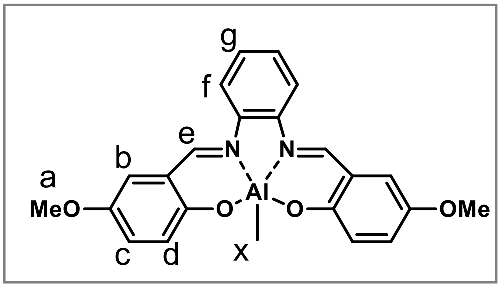

($A_4C_6B_1$)AlMe. Followed the procedure in S2.6. The crude solid was washed by hexane, and diethyl ether to afford a yellow solid. ^{1}H NMR (600 MHz, toluene-d_8, 85 °C) δ 7.21-6.82 (m br, 12H, b, c, d, e, f, g), 3.55 (m br, 6H, a), 0.40, 0.15 (s, 3H, x). ^{1}H NMR suggested that the complex has aggregation states in the solution.

ESI-MS. Exact mass calculated for $[M-CH_3]^+$ ($C_{22}H_{18}AlN_2O_4$): 401.1082, found: 401.0976.

Round 3 catalysts in Table S8:

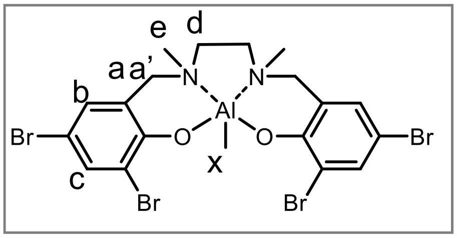

$(A_{16}C_1B_2)AlMe$. Followed the procedure in S2.6. The crude solid was washed by hexane and cold toluene to afford the product as an off-white solid. ^{1}H NMR (400 MHz, THF-d_8) δ 7.51 (s, 2H, b), 7.04 (s, 2H, c) 4.19-3.38 (m, 4H, a, a'), 3.10-2.80 (m, 4H, d), 2.32 (s, 6H, e), -0.75 – -0.98 (m, 3H, x). ^{13}C NMR (125 MHz, THF-d_8) δ 157.17, 135.38, 131.48, 125.91, 115.88, 107.57, 63.16, 61.15, 55.17, 44.56, 14.63. ^{13}C NMR suggested that the complex has aggregation states in THF solution.

ESI-MS. Exact mass calculated for $[M-CH_3]^+$ ($C_{19}H_{21}AlBr_4N_2O_2$): 640.7876, found: 640.7876.

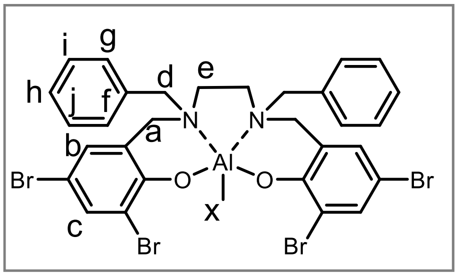

$(A_{16}C_1B_3)AlMe$. Followed the procedure in S2.6. The crude solid was washed by hexane, to afford the product as an orange solid. ^{1}H NMR (400 MHz, toluene-d_8) δ 7.64 (s, 2H, c), 7.17-6.71 (m, 12H, b, g, i, j, f, h), 3.20 (m, 8H, a, d), 2.30 (br, 4H, e), -0.31 (s, 3H, x). ^{13}C NMR (125 MHz, toluene-d_8) δ 155.90, 136.91, 134.92, 131.94, 130.42, 128.94, 128.83, 127.25, 125.28, 123.14, 116.44, 107.47, 56.96, 56.26, 46.38, 21.02.

ESI-MS. Exact mass calculated for $[M-CH_3]^-$ ($C_{30}H_{26}AlBr_4N_2O_2$): 792.8502, found: 792.8582.

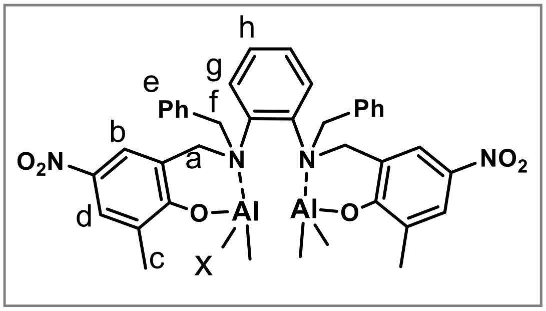

$(A_5C_6B_3)Al_2Me_4$. Followed the procedure in S2.6 using 2 equiv. $AlMe_3$ as 1 equiv. $AlMe_3$ got mixed products. The crude solid was washed by hexane to afford the product as a brown solid. ^{1}H NMR (600 MHz, toluene-d_8, 85 ºC) δ 8.04 (d, 2H, b), 7.73 (m, 2H, d), 7.23-6.41 (m, 14H, e, g, h), 4.01-3.62 (m, 4H, a, f), 0.96 (d, 6H, c), 0.09 (s, 3H, x). ^{13}C NMR was not acquired due to the low solubility of the complex.

ESI-MS. Exact mass calculated for $[M+Na]^+$ ($C_{40}H_{44}Al_2N_4O_6Na$): 753.2784, found: 753.3102.

S3. Polymerization Procedures

In a glove box, *rac*-lactide (200 mg, 1.39 mmol) in the toluene (1 mL) was mixed with benzyl alcohol (1.5 mg, 0.014 mmol) and Al catalyst (0.014 mmol) in a 15 mL thick-wall glass vessel equipped with a stirrer bar ([*rac*-LA]/[Al]/[BnOH] = 100/1/1). The reaction was stirred at 70 ºC for overnight. The reaction was cooled to room temperature, and an aliquot of the solution was dried for NMR analysis to determine the conversion and stereochemistry (see S1.2). The remaining solution was dried, and the obtained solid was washed by excess methanol to remove the residue monomers for SEC analysis (see S1.3).

S4. Computation Studies

S4.1 DFT computation

DFT calculations were performed using the Gaussian 16 software package.[15] All geometries were optimized using the B3LYP-D3 functional with 6-31g(d) basis set,[16-19] with an SMD solvation model for toluene.[20] Single-point energies of the optimized structures were calculated by ORCA[21, 22] using ωB97M-V functional[23] with def2-TZVP basis set[24] and a SMD solvation model for toluene. For the ligands studied where X-ray crystal structures were available as input starting structures, this level of theory replicated geometries and bond metrics well.[25] Vibrational frequencies were computed at the same level of theory, and all stationary points were verified to be minima (zero imaginary frequencies). Free energies of species involved in the heated reactions were corrected to 70 or 110 °C to match the experimental protocol using the Shermo program.[26] The Gaussian 16 output files for descriptor generation can be found in https://github.com/hlxin.

S4.2 Descriptor generation for machine learning

Descriptors for each of the two fragments of each whole molecule are calculated before they are concatenated to form a single fixed-length feature vector to represent the whole molecule. Source code and descriptor computation scripts can be found in https://github.com/hlxin.

DFT descriptors

We modified *auto-qchem* package[27] (https://github.com/PrincetonUniversity/auto-qchem) to generate below descriptors from Gaussian output files. The DFT descriptors include: the number of atoms, charge, spin multiplicity, dipole moment, electronic spatial extent, self-consistent field energy, values and corrections of *E*, *H*, *G*, ZPE, stoichiometry, HOMO, LUMO, electronegativity, hardness, element labels, atomic buried volume, atomic Mulliken charge, atomic polar tensor charge, vibrational frequencies, reduced mass, force constants, IR intensity and steric descriptors. Steric descriptors (e.g., sterimol[28]) for each molecule were measured by MORFEUS (github.com/kjelljorner/morfeus) using on the Gaussian 16 output results. All DFT descriptor sets used in this work can be found in https://github.com/hlxin.

One-hot-encoding.

One-hot-encoding (OHE) is one of the simplest ways to represent categorical reaction data. Here the presence or absence of a given component (e.g., functional groups) is represented by a 1 or a 0, respectively. All OHE descriptor sets used in this work can be found in https://github.com/hlxin.

Mordred descriptors

The open-source molecular descriptor-calculation software Mordred (https://github.com/mordred-descriptor/mordred) can be used to calculate two- and three-dimensional descriptors for molecules.[29] Mordred contains diverse cheminformatics properties of each molecule such as connectivity and topological information. All Mordred descriptor sets used in this work can be found in https://github.com/hlxin.

EI descriptors

Electrotopological-state index (EI)[30] are a group of atomic level descriptors that are calculated for each atom (such as C, N, O) or hydride group (such as -CH3, -NH, -OH) to characterize their chemical environment in the molecule. The focus of the method is on the individual atoms and hydride groups of the molecular skeleton. We used *rdkit.Chem.Estate* package (https://www.rdkit.org/docs/source/rdkit.Chem.EState.html) to generate EI descriptors, and all EI descriptor sets used in this work can be found in https://github.com/hlxin.

CM descriptors

Coulomb matrix (CM)[31] is a simple global descriptor which mimics the electrostatic interaction between nuclei. We used *smi2cm* package (https://github.com/avanteijlingen/smi2cm) to generate CM descriptors which are sorted eigenvalues of the Coulomb matrix, and all CM descriptor sets used in this work can be found in https://github.com/hlxin.

S4.3 Principles of Gaussian process regression

The principles of Gaussian Process Regression model[32] are as follow:

The observed target value (observed P_m P_r value in our case) can be written as:

$$y = f(\mathbf{x}) + \varepsilon \tag{1}$$

where ε is the noise that follows an independently, identically distributed Gaussian distribution as prior:

$$\varepsilon \sim \mathcal{N}(0, \sigma_n^2) \tag{2}$$

$f(\mathbf{x})$ is the predicted value of the function we try to fit and can be regarded as a linear regression model in projected feature space:

$$f(x) = \phi(\mathbf{x})^{\mathrm{T}}\mathbf{w} \tag{3}$$

where $\mathbf{w}$ is the vector of weights that follow an Gaussian distributions as prior:

$$\mathbf{w} \sim \mathcal{N}(\mathbf{0}, \Sigma_p) \tag{4}$$

$\phi(\mathbf{x})$ is a function that projects original feature $\mathbf{x}$ into a new feature space for better fitting and is eigenfunction of variance kernel:

$$k(\mathbf{x}, \mathbf{x}') = \phi(\mathbf{x})^{\mathrm{T}}\Sigma_p\phi(\mathbf{x}') \tag{5}$$

Using all priors above and likelihood $p(\mathbf{y}|X, \mathbf{w}) = \mathcal{N}(X^{\mathrm{T}}\mathbf{w}, \sigma_n^2 I)$, the posterior predictive distributions of new points can be computed as Gaussian distributions based on Bayes' rule:

$$\mathbf{f}_*|X, \mathbf{y}, X_* \sim \mathcal{N}\left(\bar{\mathbf{f}}_*, \mathrm{cov}(\mathbf{f}_*)\right) \tag{6}$$

$$\mathbf{f}_* = \mathbf{k}_*^{\mathrm{T}}[K + \sigma_n^2 I]^{-1}\mathbf{y} \tag{7}$$

$$\mathrm{cov}(\mathbf{f}_*) = k(\mathbf{x}_*, \mathbf{x}_*) - \mathbf{k}_*^{\mathrm{T}}(K + \sigma_n^2 I)^{-1}\mathbf{k}_* \tag{8}$$

The covariance kernel as a function of the distance $\boldsymbol{r}$ between two points we use is $Matérn$ class:

$$k(\boldsymbol{r}) = \alpha\frac{2^{1-\upsilon}}{\Gamma(\upsilon)}\left(\frac{\sqrt{2\upsilon}\boldsymbol{r}}{l}\right)^{\upsilon} K_\upsilon\left(\frac{\sqrt{2\upsilon}\boldsymbol{r}}{l}\right) \tag{9}$$

where Γ is the gamma function and K_υ is the modified Bessel function. There are three free parameters: α is the output scale parameter, υ is the shape parameter, and l is the length scale parameter. These three free parameters together with noise variance σ_n^2 are called hyperparameters of Gaussian process regression and need to be optimized. We use the priors same as the defaults in *edbo* package (https://github.com/b-shields/edbo) because we found that the Gaussian process regression performance is not sensitive to these settings. υ is discrete and it is set to 2.5. Gamma distribution priors are added to the rest three in

order to avoid overfitting by considering the outcome irrelevance of many of the high dimensional features. Automatic relevance determination (ARD) that assign different length scales to different feature dimensions is utilized. The three continuous hyperparameters α, l and σ_n^2 are optimized through maximizing their joint posterior distribution:

$$p(\alpha, l, \sigma_n^2 | \mathbf{y}, X) = \frac{p(\mathbf{y}|X, \alpha, l, \sigma_n^2) p(\alpha, l, \sigma_n^2)}{p(\mathbf{y}|X)} \sim p(\mathbf{y}|X, \alpha, l, \sigma_n^2) p(\alpha, l, \sigma_n^2) \quad (10)$$

where the left part is the marginal likelihood that incorporates a trade-off between model fit and model complexity, and the right part is the multiplication of all the hyperparameter priors. Gradient-based optimizer Adam is utilized to optimize the log form of the expression above:

$$\log\left[p(\mathbf{y}|X, \alpha, l, \sigma_n^2) p(\alpha, l, \sigma_n^2)\right] = -\frac{1}{2}\mathbf{y}^{\mathrm{T}} K_y^{-1} \mathbf{y} - \frac{1}{2}\log|K_y| - \frac{n}{2}\log 2\pi + \log p(\alpha, l, \sigma_n^2) \quad (11)$$

where the first term is data-fit term, the second term is model complexity penalty, the third term is a normalization constant, and the last term is the hyperparameters priors.

S4.4 Principles of Bayesian optimization

We modified codes in *edbo* package to fulfill our need for Bayesian optimization. A key component of Bayesian optimization is the acquisition function that determines which new points to select based on the current training dataset. The acquisition function we utilize is expected improvement (EI) which is most frequently used for Bayesian optimization. The definition of EI is as follows: first, let's define the utility function:

$$u(x) = \max(0, f(x) - f') \quad (12)$$

$f(x)$ is the target random variable (P_{m}, P_{r} in our case) at point x sampled from the Gaussian distribution (mean and variance are the Gaussian process regression outputs at that point). f' is the target maximum in the current training set. Utility function captures the improvement of each point x within one iteration. Second, the acquisition function is defined to be the expected value of utility function:

$$a_{EI}(x) = \mathbb{E}\,[u(x)] = \int_{f'}^{+\infty} (f - f')\mathcal{N}\big(f; \mu(x), K(x,x)\big)df$$
$$= (\mu(x) - f')\big[1 - \Phi\big(f'; \mu(x), K(x,x)\big)\big]$$
$$+ K(x,x)\mathcal{N}\big(f'; \mu(x), K(x,x)\big) \quad (13)$$

where $\Phi\big(f'; \mu(x), K(x,x)\big)$ is the cumulative distribution function (CDF) of the Gaussian distribution. In the form of the final expansion, in order to maximize the whole function, we can either maximize the predicted mean value $\mu(x)$ in the first term or maximize the predicted covariance $K(x,x)$ in the second term, which can be interpreted as the trade-off between exploitation and exploration, respectively.

S4.5 Evaluation of descriptor performance and Bayesian optimization searching efficiency

The initial dataset for machine learning (ML) were 56 data points (i.e. 56 unique Al complexes) collected from literature (see Table S1).[4, 5, 7, 13, 33-37] As shown in Figure 1, the Al complexes were fragmented into substituents A_m and B_nC_p. Descriptors corresponding to each substituent in these 56 data points were generated by various methods (see section S4.2), and combined together for Bayesian optimization using Gaussian process regression model using re-coded *edbo* package (source code see https://github.com/hlxin). For Gaussian process regression (GPR), we use the Matern covariance kernel function. In the descriptor evaluation based on the 56 literature data points among one-hot encoding, density functional theory, electrotopological-state index and coulomb matrix descriptors, for each representation, feature decorrelation by removing features with Pearson correlation coefficient over 0.95 was carried out before training the ML model in order to reduce the chance of overfitting. We applied 5-fold cross validation for the training, i.e. separating the randomly shuffled dataset into 45 points for training and 11 points for test in each fold. Both the mean and standard deviation values of prediction mean absolute errors based on 5-cross validation of these 5 representations were shown in Figure 2a.

We did not use the cross-validation method to tune the GPR hyperparameters, therefore there is no validation set. Instead, due to the probabilistic nature of GPR, the hyperparameters are optimized by maximizing (with gradient-based optimizer Adam) the

posterior likelihood function directly on the training set during training (see mathematical details in S4.3 in the Supplementary Information). In the 5-fold cross validation test for descriptor comparison, there are also only training and test sets with the ratio of 4:1.

For Bayesian optimization, we use the Expected Improvement (EI) acquisition function. To evaluate the search efficiency of Gaussian process regression in BO, we compared our algorithm using DFT descriptors with random search using the initial dataset. 3 initial points are selected randomly, and 3 points are proposed in each iteration. The comparison of search performance between BO and random search (RS) both averaged over 10 independent runs (each run having 12 iterations) is shown in Figure 2c-d. Each of the 10 independent BO runs will propose 3 points in each iteration. Up to the i-th iteration, each BO run has proposed 3×i points in total, and the maximum of these points is the highest observed P_m or P_r up to the i-th iteration. For the exploration over the overall chemical space, in each iteration, we further select the model proposed new catalysts which are empirically estimated to be able to be synthesized within a small number of steps.

Considering the complexity of new molecule synthesis, a metric called synthetic scale is proposed, based on the sum of evaluated number of synthesis steps of each of the two split small molecules of each whole Al-complex. Values from low to high correspond to the increase in synthesis difficulty, and we only synthesize the model-suggested catalysts with low synthetic scale values (see Table S2). This can be regarded as an extra component of the acquisition function in addition to mean and variance.

S4.6 Chemical space analysis

Starting from these 56 literature data points as the initial training set, we search the entire chemical space. Considering the large numbers of descriptors for each molecule, we use dimensionality reduction (e.g., principle component analysis (PCA)) to visualize the feature space. The distribution of the initial training set over the entire chemical space is projected onto 2-dimension using t-SNE algorithm[38] under *Sklearn* library (https://scikit-learn.org/stable/modules/generated/sklearn.manifold.TSNE.html), as shown in Supplementary Figure 5. Most of these data points are concentrated in a small local area,

so in order to find more points close to the global optima, it is necessary to run multiple iterations and sample more broadly to obtain a training set that is more representative.

S4.7 SHAP analysis

We used the SHAP (SHapley Additive exPlanations) package (https://github.com/slundberg/shap), a game theoretic approach to explain the output of a machine learning model, to calculate the Shapley value, the magnitude of contribution of each feature in DFT representation for the determination of P_m or P_r referenced to output averages.

As its name implies, there are two key components of SHAP analysis approach. The first one is the additive feature attribution methods in which an effect is attributed to each feature and the sum of effects of all the features approximates the output of the original model:

$$g(z') = \phi_0 + \sum_{i=1}^{M} \phi_i z_i' \quad (14)$$

where ϕ_0 is the mean value of model output, M is the number of input features, ϕ_i is the effect or contribution of each feature and $z_i' \in \{0,1\}^M$ for the unstructured data. The second component of SHAP is to use Shapley value to calculate ϕ_i. For a machine learning model with multiple features, the Shapley value for a certain feature is defined as the average expected marginal contribution of that feature, i.e. the difference between model output with and without that feature, after all possible sequential combinations have been considered:

$$\phi_i = \sum_{S \subseteq F \setminus \{i\}} \frac{|S|!(|F|-|S|-1)!}{|F|!} [f_{S\cup\{i\}}(x_{S\cup\{i\}}) - f_S(x_S)] \quad (15)$$

where i is the feature index, F is the full feature set, S is a subset from F without feature i. The features not included in S are marginalized and sometimes approximated as the corresponding feature values from randomly selected samples from the training dataset. The SHAP value is close to the classic Shapley value, but with model output f being replaced by a conditional expectation function of the original model $E[f(x)|x_S]$, considering that most models cannot handle arbitrary patterns of missing input values.

S4.8. Multivariate linear regression

Multivariate linear regression analysis was performed using R 4.1.2. The descriptors for the model were selected based on the SHAP analysis, selecting most impactful descriptors that were mechanistically meaningful for analysis.

S4.9. Comparing Bayesian optimization with other optimization algorithms

In this section, we evaluated random forest regression (RFR)[39] as the ML surrogate model and sequential model-based algorithm configuration (SMAC)[40] as the optimization method, and compared them to our current method.

(1) We found that for regression, RFR and GPR exhibited similar prediction error (in terms of both mean and standard deviation values) in a 5-fold cross validation test, as shown in Supplementary Table 13.

(2) For searching performance, BO is more efficient than SMAC, in the search for the catalyst with highest P_m or P_r value in the 56 literature data points. We found that BO could achieve convergence within 5 iterations while SMAC required 11 iterations for searching highest P_m values. For searching highest P_r values BO is as efficient as SMAC as both converged at the 7th iteration. The optimization curves of BO and SMAC are shown below in Supplementary Figure 2.

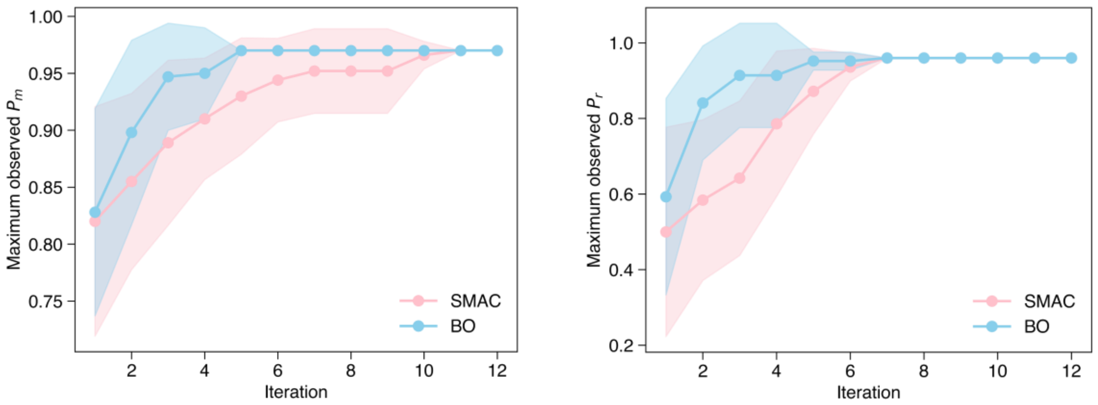

Supplementary Figure 2. The optimization curves for 12-round search of the maximum P_m (left) and P_r values (right) (P_m, probability of *meso* linkages; P_r, probability of *racemic* linkages) using sequential model-based algorithm configuration (SMAC, red) or Bayesian optimization (blue) methods. Each optimization process was independently repeated for 10

runs (12 iterations per run). Data are shown as the mean value with the standard deviation (band width) of the highest P_m (left) or P_r (right) observed up to each iteration.

(3) The SHAP analysis based on RFR and our GPR is shown below (Supplementary Figure 3). Both indicate the importance of E_HOMO and $\mathrm{V_{Bur}}$ in determining P_m (or P_r) values. However, in terms of the range of SHAP value for each descriptor, the SHAP analysis based on RFR is only able to clearly quantify the correlation for the top-ranked descriptors, while the one based on our GPR can quantify more descriptors, which allows for potential deep understanding of the nuance difference among structurally similar ligands.

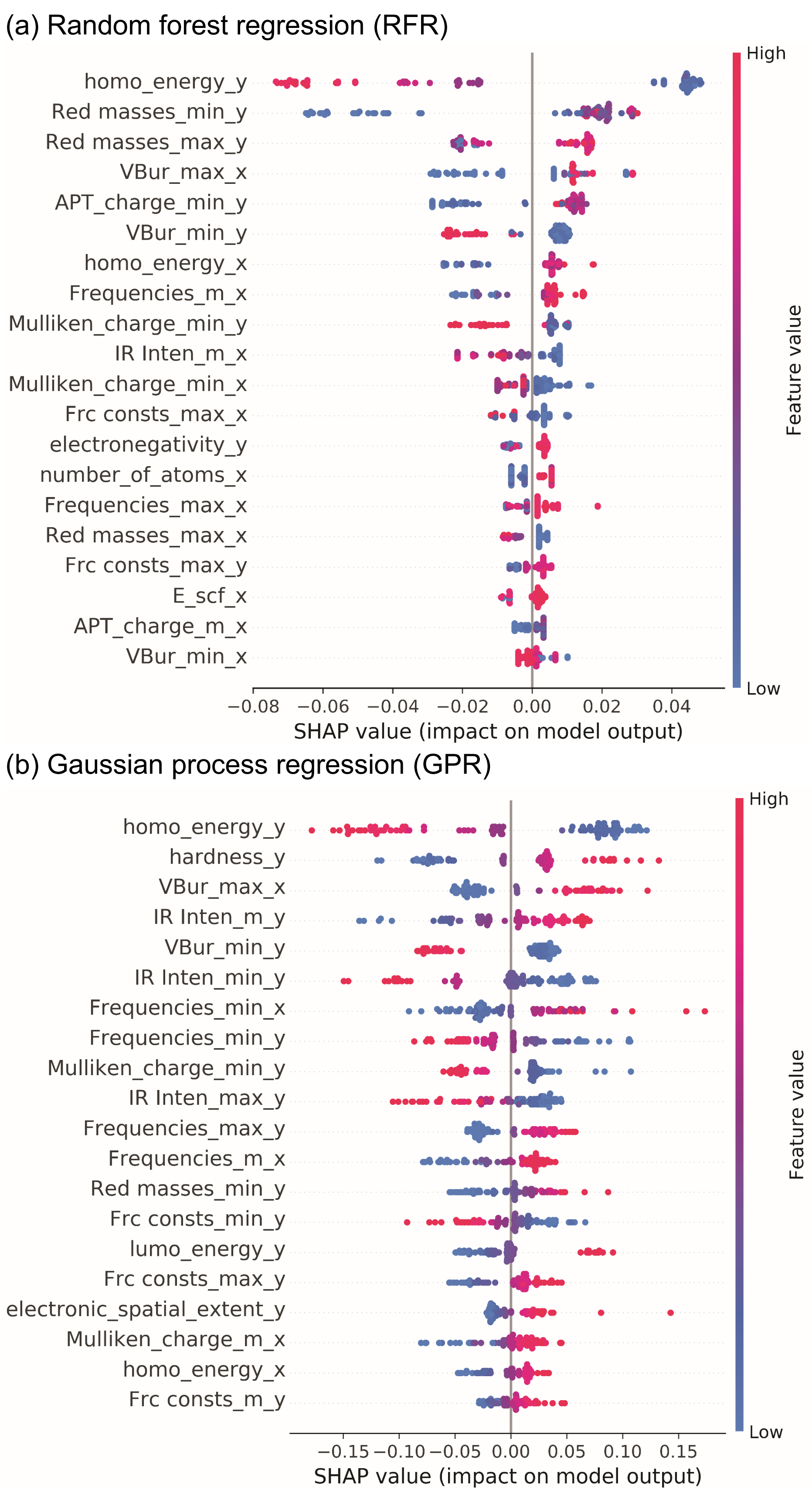

(a) Random forest regression (RFR)
homo_energy_y
Red masses_min_y
Red masses_max_y
VBur_max_x
APT_charge_min_y
VBur_min_y
homo_energy_x
Frequencies_m_x
Mulliken_charge_min_y
IR Inten_m_x
Mulliken_charge_min_x
Frc consts_max_x
electronegativity_y
number_of_atoms_x
Frequencies_max_x
Red masses_max_x
Frc consts_max_y
E_scf_x
APT_charge_m_x
VBur_min_x
−0.08 −0.06 −0.04 −0.02 0.00 0.02 0.04
SHAP value (impact on model output)
High
Low
Feature value
(b) Gaussian process regression (GPR)
homo_energy_y
hardness_y
VBur_max_x
IR Inten_m_y
VBur_min_y
IR Inten_min_y
Frequencies_min_x
Frequencies_min_y
Mulliken_charge_min_y
IR Inten_max_y
Frequencies_max_y
Frequencies_m_x
Red masses_min_y
Frc consts_min_y
lumo_energy_y
Frc consts_max_y
electronic_spatial_extent_y
Mulliken_charge_m_x
homo_energy_x
Frc consts_m_y
−0.15 −0.10 −0.05 0.00 0.05 0.10 0.15
SHAP value (impact on model output)
High
Low
Feature value

Supplementary Figure 3. Descriptor ranking by SHAP (SHapley Additive exPlanations) values based on (a) random forest regression (RFR), and (b) Gaussian process regression (GPR) for optimizing P_m values (P_m, probability of *meso* linkages) of the entire dataset of Al complexes.

S4.10. The Interdependencies of DFT Descriptors

With DFT descriptors, from the Pearson correlation matrix, the highly correlated feature pairs are extracted and tabulated in Supplementary Table 14 (x: A_m fragment, y: B_nC_p fragment). Below are the relationships with high correlation: (1) For both A_m and B_nC_p, the number of atoms is positively correlated with all the four thermodynamic energy corrections. This is obvious because more atoms mean higher degrees of freedom and free energy correction per degree of freedom is a constant. (2) For both A_m and B_nC_p, the four thermodynamic energy corrections are correlated to each other. The reason is the same as the first one, i.e. all the correction terms are linearly dependent on the number of atoms so they themselves are also linearly correlated to each other. (3) For both A_m and B_nC_p, the four thermodynamic energies, i.e. self-consistent field energy (DFT energy), ZPE (zero point energy), E, H, G are almost the same. This is because the thermodynamic energy corrections (~10^{-1}) added to the self-consistent field energies are so tiny compared to the self-consistent field energies (~10^{3}) themselves. (4) For A_m, the LUMO energy is negatively correlated with electronegativity. This is because the electronegativity here is defined as the negative value of the average of HOMO and LUMO energies and these two energies are kind of positively correlated to each other. (5) For B_nC_p, the number of atoms is negatively correlated with all the four energies. The reason is that various B_nC_p mainly differ in the number of carbon atoms and more carbon atoms lead to more C-C bonds and lower formation energies. (6) For B_nC_p, the electronic spatial extension is negatively correlated with all the four energies. The reason is that the more atoms included, the larger the molecular fragment can be, and further out the electronic density can extend with significant probability, i.e. higher electronic spatial extension. And the number of atoms is negatively correlated with the total energy. (7) For B_nC_p, the mean reduced mass is negatively correlated to the mean frequency. This can be understood in a simple harmonic

oscillation model where the vibrational frequency is expressed as: $\nu = \frac{1}{2\pi}\sqrt{\frac{k}{\mu}}$, where μ is the reduced mass of the system.

S5. Supplementary Tables

Supplementary Table 1. Literature data of stereoselective ROP of *rac*-lactide by aluminum complexes with salen-type ligands.[a]

Ligand	P_m	Ref.	Ligand	P_m	Ref.
$A_1C_1B_1$	0.72	4	$A_6C_3B_1$	0.59	13
$A_1C_1B_2$	0.68	7	$A_6C_5B_1$	0.57	41
$A_1C_1B_3$	0.79	7	$A_6C_5B_2$	0.30	34
$A_1C_2B_1$	0.77	4	$A_6C_8B_1$	0.37	13
$A_1C_3B_1$	0.63	13	$A_6C_9B_1$	0.60	13
$A_1C_5B_2$	0.64	34	$A_7C_1B_1$	0.70	13
$A_1C_8B_1$	0.84	13	$A_8C_1B_1$	0.90	33
$A_1C_9B_1$	0.50	13	$A_8C_3B_1$	0.93	33
$A_1C_{11}B_1$	0.72	36	$A_8C_6B_1$	0.45	33
$A_2C_{11}B_1$	0.86	36	A_9C1B1	0.78	4
$A_3C_1B_1$	0.83	13	$A_9C_2B_1$	0.82	4
$A_3C_1B_2$	0.58	7	$A_{10}C_1$B1	0.82	4
$A_3C_1B_3$	0.39	7	$A_{10}C_2B_1$	0.83	4
$A_3C_2B_1$	0.92	4	$A_{10}C_3B_1$	0.85	4
$A_3C_3B_1$	0.93	4	$A_{10}C_6B_1$	0.68	35
$A_3C_4B_1$	0.92	4	$A_{11}C_{10}B_1$	0.65	5
$A_3C_5B_1$	0.93	37	$A_{12}C_{10}B_1$	0.65	5
$A_3C_6B_1$	0.79	35	$A_{13}C_2B_1$	0.90	4
$A_3C_7B_1$	0.72	13	$A_{13}C_5B_1$	0.88	41
$A_3C_8B_1$	0.63	13	$A_{14}C_{10}B_1$	0.73	5
$A_3C_9B_1$	0.55	13	$A_{14}C_1B_1$	0.69	4
$A_3C_{12}B_1$	0.88	4	$A_{14}C_1B_2$	0.20	7
$A_4C_{11}B_1$	0.83	36	$A_{14}C_1B_3$	0.17	7
$A_5C_{11}B_1$	0.76	36	$A_{14}C_2B_1$	0.78	4
$A_6C_1B_1$	0.56	13	$A_{14}C_5B_2$	0.44	34
$A_6C_1B_2$	0.12	7	$A_{15}C_3B_1$	0.97	4
$A_6C_1B_3$	0.04	7	$A_{16}C_2B_1$	0.76	4
$A_6C_2B_1$	0.60	13	$A_{16}C_3B_1$	0.64	13

[a] P_m, the probability of *meso* linkages between monomer units.

Discussion:

About chiral salan ligands:

We are fully aware that chiral centers could be introduced to both salen and salan ligands. Al complexes bearing enantiopure binaphthyl [14, 42] bipyrrolidine ligands[43-46] have been shown to mediate stereoselective ROP via enantiomorphic site control. A few reasons listed below led us not to adapt these interesting ligands in our initial establishment of adopting Bayesian optimization for stereoselective catalyst development:

(1) When introducing these chiral ligands, different stereoisomers of ligands have to be studied, namely the (*R*,*R*), (*S*,*S*), the meso (*R*,*S*) forms, and the racemic form ((*R*,*R*) + (*S*,*S*)). It remains difficult now to establish machine learning models using DFT descriptors to reflect the nuance between the ligand isomers, and even isomer mixtures. Even for the current machine learning models in organic chemistry, the skeletons of chiral ligands are often fixed without directly manipulating the chiral centers[47, 48] (in the references only one chirality of the ligands are considered). In addition, when considering chirality, the configuration of M(salan) or M(salen) can be complicated: trans, *cis*-α and *cis*-β, and the *cis* metal complex can have two chiral configurations (*Δ* and *Λ,* see Supplementary Fig. 4a). Therefore, we may not use the fragmentation strategy to separate the ligands into two parts. Furthermore, it is also computationally difficult to predict the metal complex configuration even we perform the whole metal complex DFT computation. In brief, from a computation point of view, it remains challenging to incorporate such complicated ligands into the machine learning model at the beginning of the development stage.

(2) Such problem might be resolved to use problem-specific descriptors[49] to cluster chiral bipyrrolidine-based ligands separately from other ligands. Nevertheless, only a small number of bipyrrolidine-based salan ligands (not considering isomer, 5 symmetric ligands and 1 asymmetric ligand, see Supplementary Fig. 4b) reported in the literature could be available for the training set. Such number is definitely not enough to perform a cluster-based machine-learning training.

(3) Experimentally, the mechanism of such stereoselectivity control using chiral bipyrrolidine-based salan ligands is enantiomorphic site control (or dual-stereocontrol mechanism reported by Kol and Tolman), which is different from our current chain-end control mechanism. For this type of stereoselective ROP, not only the measurement of P_m / P_r using NMR spectra is necessary, the kinetic studies are often required to determine the chirality preference of the chiral catalyst. Currently, the DFT-descriptor dataset only corresponds to the P_m/P_r value output; whereas for chiral-salan catalyst, we definitely need to incorporate the kinetic selectivity factor s ($k_{L\text{-}LA}/k_{D\text{-}LA}$) and even the rates towards *meso*-LA and *rac*-LA into the experimental output sets (polymerization of *meso*-LA by racemic binaphthyl-Al leads to heterotactic PLA instead of syndiotactic PLA[14]). Therefore, even from the experiment point of view, the chiral salan ligands should be separated from our current training sets for machine learning as these ligands need additional experiment results for model training and verification.

About salalen and other ligands:

For the salalen ligand, here are a few reasons that we did not select:

(1) The salalen ligand requests at least three steps to prepare, assuming the salicylaldehyde and one-side protected diamine are commercially available (often not; two synthetic routes

were provided according to the literature[50, 51]). This runs counter to our ligand selection rule that the ligand should be synthesized within 3 steps to accelerate the discovery process and for future scale-up (Supplementary Fig. 4c-d).
(2) One challenge for asymmetric ligand is that this increases the computational work for generating DFT descriptors. Using our current fragment method for symmetric salan and salen ligands, we only need to calculate A_m and B_nC_p two parts. While for asymmetric such as salalen, we are expected to calculate A_mB_1 (n = 1 for C=N bond), $B_1C_pB_n$, A_mB_n ($n \neq 1$ for *N*-alkyl) three parts (not considering chiral factors). This would dramatically expand the chemical space; however, only less than 20 training data points from the literature could be added to the training library. This will make the model training, optimization, and search processes more difficult, as the uncertainty gets increased without a large amount of initial training data to be input into the model.
(3) Studies have shown that salan Al complexes appear to be more active than the salalen Al complexes.[52] This is supported by the fact that many salalen-Al-mediated ROP of *rac*-LA produced polymers with M_ns often less than 15 kDa [50, 51, 53]. Notably, ethylene-bridged salalen-Al complexes showed slight isoselectivities, whereas cyclohexane-bridged salalen-Al complexes showed moderate heteroselectivity. [50, 53]
(4) Moreover, some chiral salalen complexes involved somewhat complicated stereoselectivity mechanisms. Kol and coworkers found that the ROP using one salalen-Al complex had a selectivity factor of 10, indicating enantiomorphic site control mechanism.[51] However, the k_{app} for *rac*-LA was similar to that of D-LA and slower than L-LA. The chain-end control seemed to be the prevailing mechanism during enchainment at high monomer conversion, whereas the enantiomorphic site control occurred at low monomer conversion. For such complex systems, as we discussed above for the chiral salan ligands, we could not simply incorporate them with other ligands as more experimental measurements will be needed besides NMR studies. Again, at the beginning stage, we could not introduce such complicated systems with laborious synthetic demands into the machine-learning based workflow.

The emphasis of this work is on whether the algorithm can help identify new ligands with high P_m and ligands with high P_r, without going through high-throughput synthesis approach to prepare large amounts of ligands and explore the whole chemical space. We do find a handful of ligands to achieve our goal, especially our new heteroselective Al complexes that outperformed the literature data. Therefore even the ligand structural variation is subtle, our algorithm could still help identify new high-performance catalysts through Bayesian optimization. That actually confirms that our framework is really powerful and has significant potential to recognize subtle structural variations.

We also note many other interesting ligand skeletons, such as enolic Schiff-bases ligands,[54] NNO tridentate ligands,[55] catam ligands,[56] etc., were not involved in the initial training dataset. Reasons for exclusion are similar to the above discussions on chiral salan ligands, and salalen ligands, especially lack of sufficient literature data points, having too complicated structures to start with, or requiring more kinetic experiments for identification PLA microstructures. We hope that more data-science tools, more problem-

specific descriptors, and more efficient algorithms could be added to our current system to include all above chemical knowledge into one holistic model in the near future.

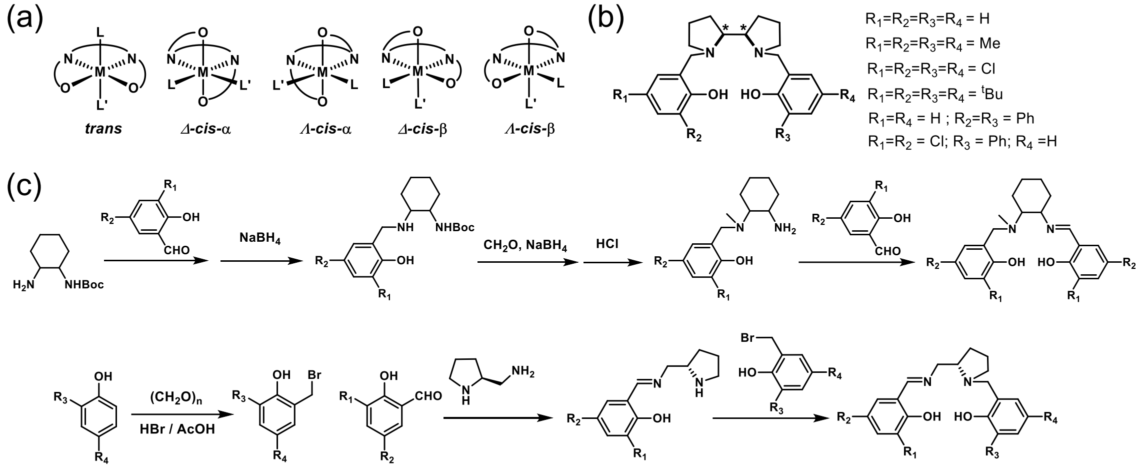

Supplementary Figure 4. (a) Possible chiral configurations of salen-metal complex. (b) The reported chiral salan ligands for PLA polymerization. (c) Two synthetic routes to prepare asymmetrical salanen ligands, which involve more than 3 synthetic steps and can be both synthetically and computationally challenge for initial BO studies without prior knowledge.

Supplementary Table 2. Synthetic scale assignments for A_m and B_nC_p fragments [a]

A_m	A_1	A_2	A_3	A_4	A_5	A_6	A_7	A_8	A_9	A_{10}	A_{11}	A_{12}	A_{13}	A_{14}	A_{15}	A_{16}
For B_1	0	0	0	0	2	0	0	2	2	6	2	4	2	2	10	0
For B_2 & B_3	0	0	0	2	2	2	2	2	2	6	2	6	2	2	10	2

B_nC_p	C_1	C_2	C_3	C_4	C_5	C_6	C_7	C_8	C_9	C_{10}	C_{11}	C_{12}
For B_1	0	0	0	4	0	0	0	2	0	6	0	4
For B_2	0	0	2	6	2	0	8	4	2	8	2	6
For B_3	0	2	2	6	2	4	8	4	2	8	2	6

[a] We assigned the value 2 for each reaction step to prepare the targeted compound (using 2 is to magnify the difference). The value 0 means commercially available. Detailed synthetic steps for individual fragment are described in S2.

Supplementary Table 3. First round Bayesian-optimization-model-proposed isoselective Al ligands, and corresponding experimental results [a]

Entry	Ligand	Predicted mean P_m	Predicted variance	Measured P_m [b]	Temp. (°C)	Conv. % [c]	M_n (kDa) [d]	MW_{cal} (kDa)	$Đ$ [d]
1	$A_7C_2B_1$	0.87	0.023	0.81	70	87.7	9.8	12.7	1.23
2	$A_{11}C_3B_1$	0.89	0.007	0.95	70	95.2	14.0	13.8	1.03
3	$A_{14}C_3B_1$	0.98	0.018	0.72	70	94.3	17.3	13.7	1.20
4	$A_{14}C_5B_1$	0.93	0.007	0.77	70	21.9	n.d.	3.2	n.d.
5	$A_{14}C_{11}B_1$	0.87	0.011	0.77	70	84.7	20.6	12.3	1.04
6	$A_8C_9B_1$	0.87	0.009	0.52	70	35.3	8.4	5.1	1.06
7 [e]	$A_3C_3B_1$	-	-	0.94	70	96.2	11.9	13.9	1.16

[a] Abbreviation: P_m, the probability of *meso* linkages between monomer units; Temp., temperature, Conv.%, LA conversion %; M_n, number-average molecular weight; MW_{cal}, molecular weight calculated from the feed ratio and monomer conversion; $Đ$, molecular weight distribution; n.d., not determined because of low monomer conversions. Polymerization conditions: [*rac*-LA]/[Al]/[BnOH] = 100/1/1 at 70 °C for 12 hours; [*rac*-LA] = 1.39 M in toluene.

[b] Determined by homodecoupled ^{1}H and ^{13}C NMR.

[c] Determined by ^{1}H NMR.

[d] Determined by size-exclusion chromatography.

[e] The ligand was synthesized according to the literature[4] as the benchmark for comparison.

Supplementary Table 4. First round Bayesian-optimization-model-proposed heteroselective Al ligands, and corresponding experimental results [a]

Entry	Ligand	Predicted mean P_r	Predicted variance	Measured P_r [b]	Temp. (°C)	Conv. % [c]	M_n (kDa) [d]	MW_{cal} (kDa)	$Đ$ [d]
1	$A_4C_1B_3$	0.87	0.008	0.11	70	95.2	18.9	13.8	1.03
2	$A_5C_1B_3$	0.97	0.002	0.93	70	81.3	8.3	11.7	1.06
3	$A_4C_1B_2$	0.86	0.008	0.15	70	94.3	15.0	13.7	1.31
4	$A_3C_6B_2$	0.77	0.029	0.54	110	55.5	12.1	8.0	1.12
5	$A_5C_6B_2$	0.91	0.016	0.83	110	88.5	20.2	12.8	1.08
6	$A_5C_2B_3$	0.96	0.007	0.52	110	87.0	20.7	12.5	1.13
7	$A_5C_5B_2$	0.88	0.006	0.74	70	90.9	12.4	13.1	1.03
8	$A_5C_3B_3$	0.92	0.019	0.45	110	40.3	24.4	5.8	1.20
9 [e]	$A_6C_1B_3$	-	-	0.89	70	75.8	12.5	11.0	1.09

[a] Abbreviation: P_r, the probability of *racemic* linkages between monomer units; Temp., temperature, Conv.%, LA conversion %; M_n, number-average molecular weight; MW_{cal}, molecular weight calculated from the feed ratio and monomer conversion; $Đ$, molecular weight distribution. Polymerization conditions: [*rac*-LA]/[Al]/[BnOH] = 100/1/1 at 70 or 110 °C for 12 hours; [*rac*-LA] = 1.39 M in toluene. We note that the selection of 110 °C for polymerization is due to the low reactivity of the Al complex at 70 °C.

[b] Determined by homodecoupled ^1H and ^{13}C NMR.

[c] Determined by ^1H NMR.

[d] Determined by size-exclusion chromatography.

[e] The ligand was synthesized according to the literature[7] as the benchmark for comparison.

Discussion:

There are 47 catalysts with P_m over 0.5, and only 9 catalysts having P_r over 0.5 in our initial training dataset. Therefore, optimization of P_r was considerably difficult comparing to optimizing P_m in our first round BO prediction. Given the limited information of heteroselective catalysts in the first round, the errors can be large in the prediction. In addition, in our first-round prediction, the descriptors of temperature and monomer feed ratios were included in the model, which unnecessarily enlarged the chemical space for prediction, thereby further increased the uncertainty and led to high prediction errors. After realizing the low relevance of these descriptors to the P_m / P_r value prediction, we removed them in the subsequent iterations, which could also contribute to the decreased errors in later P_r value prediction.

Our data shows that Gibson's Al complex (entry 9) does have hetereoselectivity but the P_r becoming lower when increasing [LA]/[Al] ratio (also the LA conversion rate of 75.8% is moderate) and cannot be used to prepare high-MW poly(*ht*-LA). In our case, our newly identified ligand $A_{16}C_1B_2$ could eventually prepare poly(*ht*-LA) with relatively higher MW (64.7 kDa) and a decent P_r value of 0.87 (Supplementary Table 9 entry 2).

Supplementary Table 5. Second round Bayesian-optimization-model-preposed isoselective Al ligands, and corresponding experimental results [a]

Entry	Ligand	Predicted mean P_m	Predicted variance	Measured P_m [b]	Temp. (°C)	Conv. % [c]	M_n (kDa) [d]	MW_{cal} (kDa)	$Đ$ [d]
1	$A_4C_8B_1$	0.90	0.009	0.86	70	97.0	27.9	14.0	1.09
2	$A_4C_2B_1$	0.83	0.013	0.52	70	96.2	25.8	13.9	1.06
3	$A_{11}C_2B_1$	0.94	0.002	0.92	70	97.0	32.2	14.0	1.05
4	$A_{16}C_7B_1$	0.72	0.026	0.41	70	73.5	13.9	10.6	1.07
5	$A_{16}C_{11}B_1$	0.85	0.010	0.79	70	93.5	21.4	13.5	1.02
6	$A_{11}C_5B_1$	0.90	0.010	n.d.	70	12.5	n.d.	1.8	n.d.
7	$A_{11}C_8B_1$	0.88	0.010	n.d.	70	19.1	n.d.	2.8	n.d.

[a] Abbreviation: P_m, the probability of *meso* linkages between monomer units; Temp., temperature, Conv.%, LA conversion %; M_n, number-average molecular weight; MW_{cal}, molecular weight calculated from the feed ratio and monomer conversion; $Đ$, molecular weight distribution; n.d., not determined because of low monomer conversions. Polymerization conditions: [*rac*-LA]/[Al]/[BnOH] = 100/1/1 at 70 °C for 12 hours; [*rac*-LA] = 1.39 M in toluene.

[b] Determined by homodecoupled ^1H and ^{13}C NMR.

[c] Determined by ^1H NMR.

[d] Determined by size-exclusion chromatography.

Supplementary Table 6. Second round Bayesian-optimization-model-proposed heteroselective Al ligands, and corresponding experimental results [a]

Entry	Ligand	Predicted mean P_r	Predicted variance	Measured P_r [b]	Temp. (°C)	Conv. % [c]	M_n (kDa) [d]	MW_{cal} (kDa)	$Đ$ [d]
1	$A_6C_2B_2$	0.89	0.003	0.51	110	82.6	13.8	11.9	1.09
2	$A_5C_2B_2$	0.87	0.007	0.31	110	90.9	14.4	13.1	1.07
3	$A_5C_1B_2$	0.87	0.007	0.94	70	78.1	11.1	11.3	1.01
4	$A_6C_6B_2$	0.82	0.025	0.75	110	82.6	13.8	11.9	1.09
5	$A_2C_1B_3$	0.76	0.010	0.61	110	87.7	12.2	12.6	1.04

[a] Abbreviation: P_r, the probability of *racemic* linkages between monomer units; Temp., temperature, Conv.%, LA conversion %; M_n, number-average molecular weight; MW_{cal}, molecular weight calculated from the feed ratio and monomer conversion; $Đ$, molecular weight distribution. Polymerization conditions: [*rac*-LA]/[Al]/[BnOH] = 100/1/1 at 70 or 110 °C for 12 hours; [*rac*-LA] = 1.39 M in toluene. We note that the selection of 110 °C for polymerization is due to the low reactivity of the Al complex at 70 °C.

[b] Determined by homodecoupled ^{1}H and ^{13}C NMR.

[c] Determined by ^{1}H NMR.

[d] Determined by size-exclusion chromatography.

Supplementary Table 7. Third round Bayesian-optimization-model-proposed isoselective Al ligands, and corresponding experimental results [a]

Entry	Ligand	Predicted mean P_m	Predicted variance	Measured P_m [b]	Temp. (°C)	Conv. % [c]	M_n (kDa) [d]	MW_{cal} (kDa)	$Đ$ [d]
1	$A_8C_{11}B_1$	0.92	0.015	0.85	70	37.5	6.5	5.4	1.05
2	$A_8C_5B_1$	0.94	0.005	0.82	110	79.4	9.8	11.4	1.02
3	$A_8C_2B_1$	0.92	0.004	0.69	70	42.4	7.3	6.1	1.11
4	$A_4C_6B_1$	0.89	0.017	0.51	70	50.3	13.4	7.3	1.06

[a] Abbreviation: P_m, the probability of *meso* linkages between monomer units; Temp., temperature, Conv.%, LA conversion %; M_n, number-average molecular weight; MW_{cal}, molecular weight calculated from the feed ratio and monomer conversion; $Đ$, molecular weight distribution; n.d., not determined because of low monomer conversions. Polymerization conditions: [*rac*-LA]/[Al]/[BnOH] = 100/1/1 at 70 °C for 12 hours; [*rac*-LA] = 1.39 M in toluene.

[b] Determined by homodecoupled ^{1}H and ^{13}C NMR.

[c] Determined by ^{1}H NMR.

[d] Determined by size-exclusion chromatography.

Discussion:

For the P_m optimization, the sufficient number of reasonable P_m-value catalysts in the initial training dataset (47 catalysts having $P_m > 0.5$, and 18 of them having $P_m \geq 0.8$) made the uncertainty of P_m optimization process much lower compared to that in the P_r optimization. In other words, there may not be enough room to decrease the prediction errors in the P_m-value optimization process. Additionally, many factors—such as experimental measurements (errors in P_r /P_m measurements) and errors in literature data (e.g., some literature data was found reportedly higher when we tried to repeat, and we could not repeat all reported catalysts) — would bring the uncertainty into the system. This uncertainty could account for a large portion in the prediction error after just three iterations.

Because of many realistic factors affecting experimental measurement, P_r /P_m values have uncertainty which may be difficult to shrink down further, even after a large number of iterations of Bayesian optimization. Ultimately the reason why we perform multiple iterations is not only trying to refine the model to make it more accurate, but mainly aiming to efficiently find more high-performance catalysts (P_m or P_r > 0.8). We believe that we have already achieved our goal of finding more high-performance catalysts guided by Bayesian optimization.

Supplementary Table 8. Third round Bayesian-optimization-model-proposed heteroselective Al ligands, and corresponding experimental results [a]

Entry	Ligand	Predicted mean P_r	Predicted variance	Measured P_r [b]	Temp. (ºC)	Conv. % [c]	M_n (kDa) [d]	MW_{cal} (kDa)	$Đ$ [d]
1	$A_{16}C_1B_2$	0.80	0.016	0.93	70	87.7	12.9	12.6	1.01
2	$A_{16}C_1B_3$	0.86	0.014	0.93	70	58.8	15.9	8.5	1.08
3	$A_5C_6B_3$	0.84	0.059	n.d.	70	16.1	n.d.	2.3	n.d.

[a] Abbreviation: P_r, the probability of *racemic* linkages between monomer units; Temp., temperature, Conv.%, LA conversion %; M_n, number-average molecular weight; MW_{cal}, molecular weight calculated from the feed ratio and monomer conversion; $Đ$, molecular weight distribution. Polymerization conditions: [*rac*-LA]/[Al]/[BnOH] = 100/1/1 at 70 °C for 12 hours; [*rac*-LA] = 1.39 M in toluene.

[b] Determined by homodecoupled ^1H and ^{13}C NMR.

[c] Determined by ^1H NMR.

[d] Determined by size-exclusion chromatography.

Discussion:

For entry 2 (also entry 6 in Table 1), the obtained polymer's M_n is higher than the calculated molecular weight. We doubt that at 70 ºC reaction temperature, moderate chain transfer might occur. Nevertheless, the molecular weight distribution was 1.08, suggesting no significant transesterification happening that often causes large distribution.

Supplementary Table 9. Thermal and mechanical properties of PLAs with various microstructures [a]

Entry	polymer	P_m / P_r	M_n (kDa) [b]	$Đ$ [b]	T_g / T_m (°C) [c]	E_y (MPa) [d]	σ (MPa) [d]	ε (%) [d]
1	poly(*sb*-LA)	0.96/0.04	44.5	1.04	- / 192	2430.6 ± 392.6	48.5 ± 1.8	5.0 ± 0.8
2	poly(*ht*-LA)	0.13/0.87	67.4	1.03	50 / -	1239.5 ± 177.9	11.2 ± 1.2	533.1 ± 44.1
3	poly(L-LA)	1/0	28.7	1.03	- / 176	1645.8 ± 281.5	30.5 ± 1.7	4.7 ± 0.9
4	poly(*r*-LA)	0.40/0.60	55.6	1.03	40 / -	1021.4 ± 22.2	8.6 ± 1.2	469.1 ± 60.9
5	poly(*sb*-LA) + poly(*ht*-LA) (1/1) [e]	-	-	-	53 / 182	2165.2 ± 214.6	23.0 ± 3.1	404.2 ± 40.2

[a] Abbreviations: M_n, number-average molecular weight; $Đ$, molecular weight distribution; T_g, glass transition temperature; T_m, melting temperature; E_y, Young's modulus; σ, fracture stress; ε, fracture strain; *sb*, stereoblock; *ht*, heterotactic, *r*, random of L- and D-LA. For all mechanical tests, n = 4-5, and data are presented as mean ± standard deviation.

[b] Determined by size-exclusion chromatography.

[c] Determined by differential scanning calorimetry. See Supplementary Fig.13.

[d] Determined by stress-strain tests. See Fig. 3d.

[e] The PLAs in entries 1 and 2 were mixed at 1/1 mass ratio in DCM solution, and dried to prepare the polymer film for mechanical testing.

Supplementary Table 10. Buried volume (V_{bur}) of the Al complex ligands [a]

Ligand	V_{bur}	P_m	Ligand	V_{bur}	P_m
$A_1C_1B_1$	0.592498	0.72	$A_6C_2B_2$	0.673325	0.49
$A_1C_1B_2$	0.650828	0.68	$A_6C_3B_1$	0.62977	0.59
$A_1C_1B_3$	0.64713	0.79	$A_6C_5B_1$	0.602264	0.57
$A_1C_2B_1$	0.62083	0.77	$A_6C_5B_2$	0.681927	0.3
$A_1C_3B_1$	0.623002	0.63	$A_6C_6B_2$	0.6758	0.25
$A_1C_5B_2$	0.666249	0.64	$A_6C_8B_1$	0.652051	0.37
$A_1C_8B_1$	0.644737	0.84	$A_6C_9B_1$	0.672382	0.60
$A_1C_9B_1$	0.664933	0.50	$A_7C_1B_1$	0.592504	0.70
$A_1C_{11}B_1$	0.605025	0.72	$A_7C_2B_1$	0.620638	0.81
$A_2C_1B_3$	0.671363	0.39	$A_8C_1B_1$	0.61563	0.90
$A_2C_{11}B_1$	0.611297	0.86	$A_8C_2B_1$	0.644393	0.69
$A_3C_1B_1$	0.63175	0.83	$A_8C_3B_1$	0.645779	0.93
$A_3C_1B_2$	0.709735	0.58	$A_8C_5B_1$	0.616905	0.82
$A_3C_1B_3$	0.711447	0.39	$A_8C_6B_1$	0.610621	0.45
$A_3C_2B_1$	0.657322	0.92	$A_8C_9B_1$	0.681175	0.52
$A_3C_3B_1$	0.658772	0.93	$A_8C_{11}B_1$	0.624126	0.85
$A_3C_4B_1$	0.660851	0.92	$A_9C_1B_1$	0.607663	0.78
$A_3C_5B_1$	0.629047	0.93	$A_9C_2B_1$	0.631016	0.82
$A_3C_6B_1$	0.623276	0.79	$A_{10}C_1B_1$	0.616666	0.82
$A_3C_6B_2$	0.709828	0.46	$A_{10}C_2B_1$	0.648749	0.83
$A_3C_7B_1$	0.64865	0.72	$A_{10}C_3B_1$	0.651049	0.85
$A_3C_8B_1$	0.680372	0.63	$A_{10}C_6B_1$	0.608519	0.68
$A_3C_9B_1$	0.702921	0.55	$A_{11}C_2B_1$	0.65749	0.92
$A_3C_{12}B_1$	0.657368	0.88	$A_{11}C_3B_1$	0.659127	0.95
$A_4C_1B_2$	0.645727	0.85	$A_{13}C_2B_1$	0.657368	0.90
$A_4C_1B_3$	0.649314	0.89	$A_{13}C_5B_1$	0.602823	0.88
$A_4C_2B_1$	0.620591	0.52	$A_{14}C_1B_1$	0.599015	0.69
$A_4C_6B_1$	0.586004	0.51	$A_{14}C_1B_2$	0.666942	0.20
$A_4C_8B_1$	0.644719	0.86	$A_{14}C_1B_3$	0.667041	0.17

$A_4C_{11}B_1$	0.605089	0.83	$A_{14}C_2B_1$	0.627341	0.78
$A_5C_1B_2$	0.675206	0.06	$A_{14}C_3B_1$	0.629548	0.72
$A_5C_1B_3$	0.679958	0.08	$A_{14}C_5B_1$	0.602829	0.77
$A_5C_2B_2$	0.692392	0.69	$A_{14}C_5B_2$	0.680156	0.44
$A_5C_2B_3$	0.677646	0.48	$A_{14}C_{11}B_1$	0.611437	0.77
$A_5C_3B_3$	0.711715	0.55	$A_{15}C_3B_1$	0.665009	0.97
$A_5C_5B_2$	0.690709	0.26	$A_{16}C_1B_2$	0.679207	0.07
$A_5C_6B_2$	0.675713	0.17	$A_{16}C_1B_3$	0.684035	0.07
$A_5C_{11}B_1$	0.609416	0.76	$A_{16}C_2B_1$	0.62963	0.76
$A_6C_1B_1$	0.599009	0.56	$A_{16}C_3B_1$	0.632012	0.64
$A_6C_1B_2$	0.671467	0.12	$A_{16}C_7B_1$	0.621244	0.41
$A_6C_1B_3$	0.673977	0.04	$A_{16}C_{11}B_1$	0.614383	0.79
$A_6C_2B_1$	0.627376	0.60			

[a] The geometries of ligands were optimized using the B3LYP-D3 functional with 6-31g(d) basis set,[16-19] with an SMD solvation model of toluene.[20] The V_{bur} values were obtained using Morfeus package in the github (kjelljorner.github.io/morfeus/index.html).[28] The data are used to plot Figure 4b and included in the source dataset.

Supplementary Table 11. The energy barrier differences in **TS1** of D- and L-LA insertion into ligand-Al-(L-LA)$_2$ and the observed P_m values.

Al Catalyst Ligand	**TS1** Δ*E* (kcal/mol)	P_m
$A_{11}C_3B_1$	4.30	0.95
$A_{11}C_2B_1$	4.13	0.92
$A_4C_8B_1$	5.98	0.86
$A_7C_2B_1$	2.77	0.81
$A_4C_1B_3$	5.80	0.89
$A_4C_1B_2$	3.30	0.85
$A_8C_5B_1$	2.38	0.82
$A_8C_{11}B_1$	6.93	0.85
$A_3C_3B_1$	3.71	0.93
$A_5C_1B_2$	1.69	0.06
$A_6C_1B_3$	1.01	0.11

Discussion:

Though the **TS1** energy differences for highly isoselective Al catalysts were usually larger than 3 kcal/mol, it remains difficult to draw any quantitative relationship between P_m and ΔE, which was different from linear free energy relationships often observed in the organic reactions.[57]

Supplementary Table 12. The energy barrier differences in **TS2** of D- and L-LA insertion into Al-(L-LA)$_2$ and the observed P_m values.

Al Catalyst Ligand	TS2 ΔE (kcal/mol)	P_m
$A_3C_3B_1$	1.04	0.93
$A_4C_1B_3$	6.79	0.89
$A_6C_1B_3$	20.58	0.11
$A_5C_1B_3$	14.70	0.06

Discussion:

Though the **TS2** energy differences for highly heteroselective Al catalysts were usually larger than 10 kcal/mol, it remains difficult to draw any quantitative relationship between P_m and ΔE.

Supplementary Table 13. Comparison of the prediction errors (in terms of both mean and standard deviation values) for different descriptors using random forest regression (RFR) and Gaussian process regression (GPR) in 5-fold cross validation tests (see **S4.9**).

Descriptor	Model	Mean	Standard deviation
DFT	GPR	0.104	0.012
	RFR	0.107	0.014
Mordred	GPR	0.100	0.013
	RFR	0.097	0.034
CM	GPR	0.109	0.010
	RFR	0.119	0.033
EI	GPR	0.102	0.020
	RFR	0.091	0.019
OHE	GPR	0.097	0.012
	RFR	0.095	0.022

[a] Abbreviations: DFT, density function theory; CM, coulomb matrix; OHE, one-hot encoding; EI, electrotopological-state index; RFR, random forest regression; GPR, Gaussian process regression.

Supplementary Table 14. Pearson correlation coefficients of various DFT descriptors that are the highly correlated feature pairs (see **S4.10**).

Feature_1	Feature_2	Pearson correlation coefficient
number_of_atoms_x	zero_point_correction_x	0.9970
number_of_atoms_x	E_thermal_correction_x	0.9976
number_of_atoms_x	H_thermal_correction_x	0.9976
number_of_atoms_x	G_thermal_correction_x	0.9954
E_scf_x	E_zpe_x	1.0000
E_scf_x	E_x	1.0000
E_scf_x	H_x	1.0000
E_scf_x	G_x	1.0000
zero_point_correction_x	E_thermal_correction_x	0.9999
zero_point_correction_x	H_thermal_correction_x	0.9999
zero_point_correction_x	G_thermal_correction_x	0.9998
E_thermal_correction_x	H_thermal_correction_x	1.0000
E_thermal_correction_x	G_thermal_correction_x	0.9995
H_thermal_correction_x	G_thermal_correction_x	0.9995
E_zpe_x	E_x	1.0000
E_zpe_x	H_x	1.0000
E_zpe_x	G_x	1.0000
E_x	H_x	1.0000
E_x	G_x	1.0000
H_x	G_x	1.0000
lumo_energy_x	electronegativity_x	0.9773
number_of_atoms_y	E_scf_y	0.9632
number_of_atoms_y	zero_point_correction_y	0.9954
number_of_atoms_y	E_thermal_correction_y	0.9962
number_of_atoms_y	H_thermal_correction_y	0.9962
number_of_atoms_y	G_thermal_correction_y	0.9936
number_of_atoms_y	E_zpe_y	0.9632
number_of_atoms_y	E_y	0.9632
number_of_atoms_y	H_y	0.9632
number_of_atoms_y	G_y	0.9632
electronic_spatial_extent_y	E_scf_y	0.9598

electronic_spatial_extent_y	E_zpe_y	0.9597
electronic_spatial_extent_y	E_y	0.9597
electronic_spatial_extent_y	H_y	0.9597
electronic_spatial_extent_y	G_y	0.9597
E_scf_y	E_zpe_y	1.0000
E_scf_y	E_y	1.0000
E_scf_y	H_y	1.0000
E_scf_y	G_y	1.0000
zero_point_correction_y	E_thermal_correction_y	1.0000
zero_point_correction_y	H_thermal_correction_y	1.0000
zero_point_correction_y	G_thermal_correction_y	0.9998
E_thermal_correction_y	H_thermal_correction_y	1.0000
E_thermal_correction_y	G_thermal_correction_y	0.9996
H_thermal_correction_y	G_thermal_correction_y	0.9996
E_zpe_y	E_y	1.0000
E_zpe_y	H_y	1.0000
E_zpe_y	G_y	1.0000
E_y	H_y	1.0000
E_y	G_y	1.0000
H_y	G_y	1.0000
Frequencies_m_y	Red masses_m_y	0.9689

The above data was also included in the source data set.

S6. Supplementary Figures (Supplementary Figs. 1-4 in S1, S4 and Table S1)

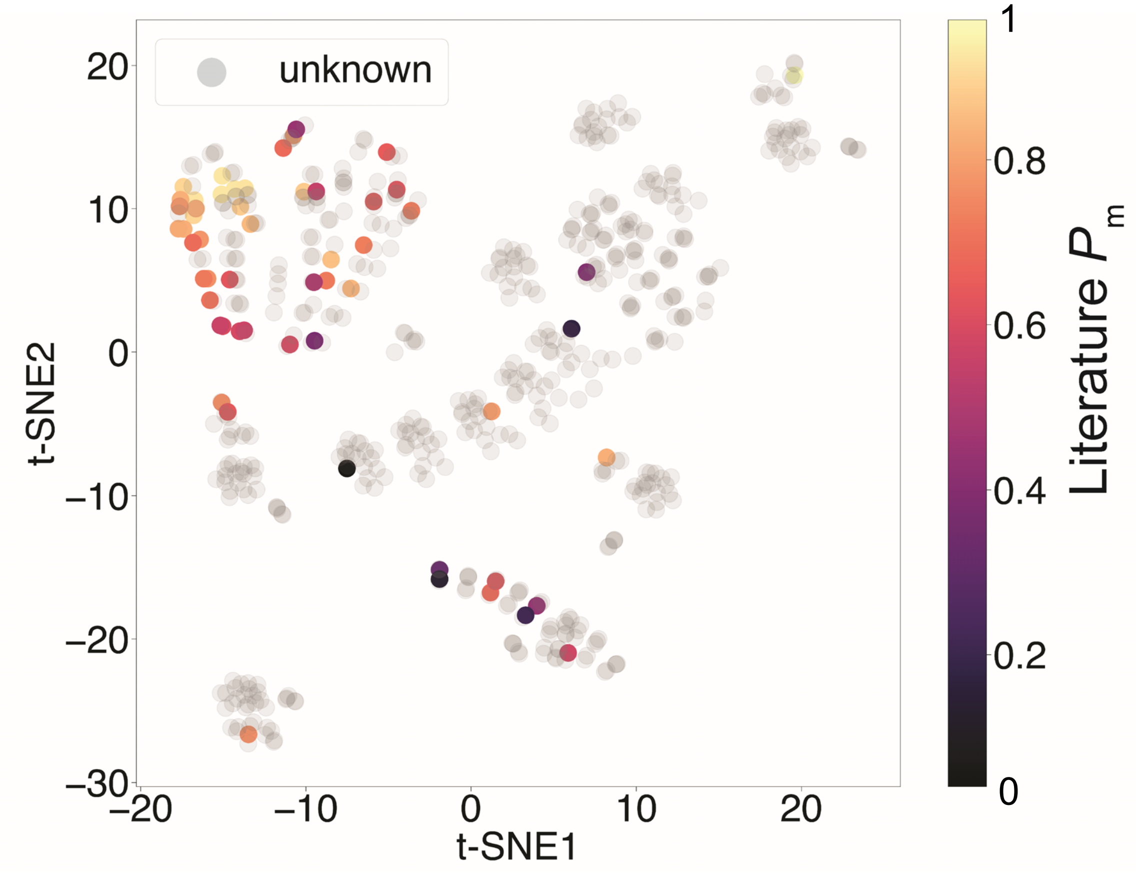

Supplementary Figure 5. The projection of the distribution of the initial training set (56 data points) over the entire chemical space (576 data points) onto 2-dimension using t-SNE algorithm (the analysis method in S4.6).

The colored points are the data points in the initial training set, and the gray points are the rest of Al complexes in the overall chemical space. This distribution indicates two things: (1) the initial data points distribution is kind of localized, and the uncertainties in the regions without known P_m values nearby should be high and worth investigating; (2) reasonable isoselective catalysts (P_m > 0.5) dominate in the initial training set, so uncertainties in the high P_r (low P_m) catalysts prediction would be high at the beginning of the optimization.

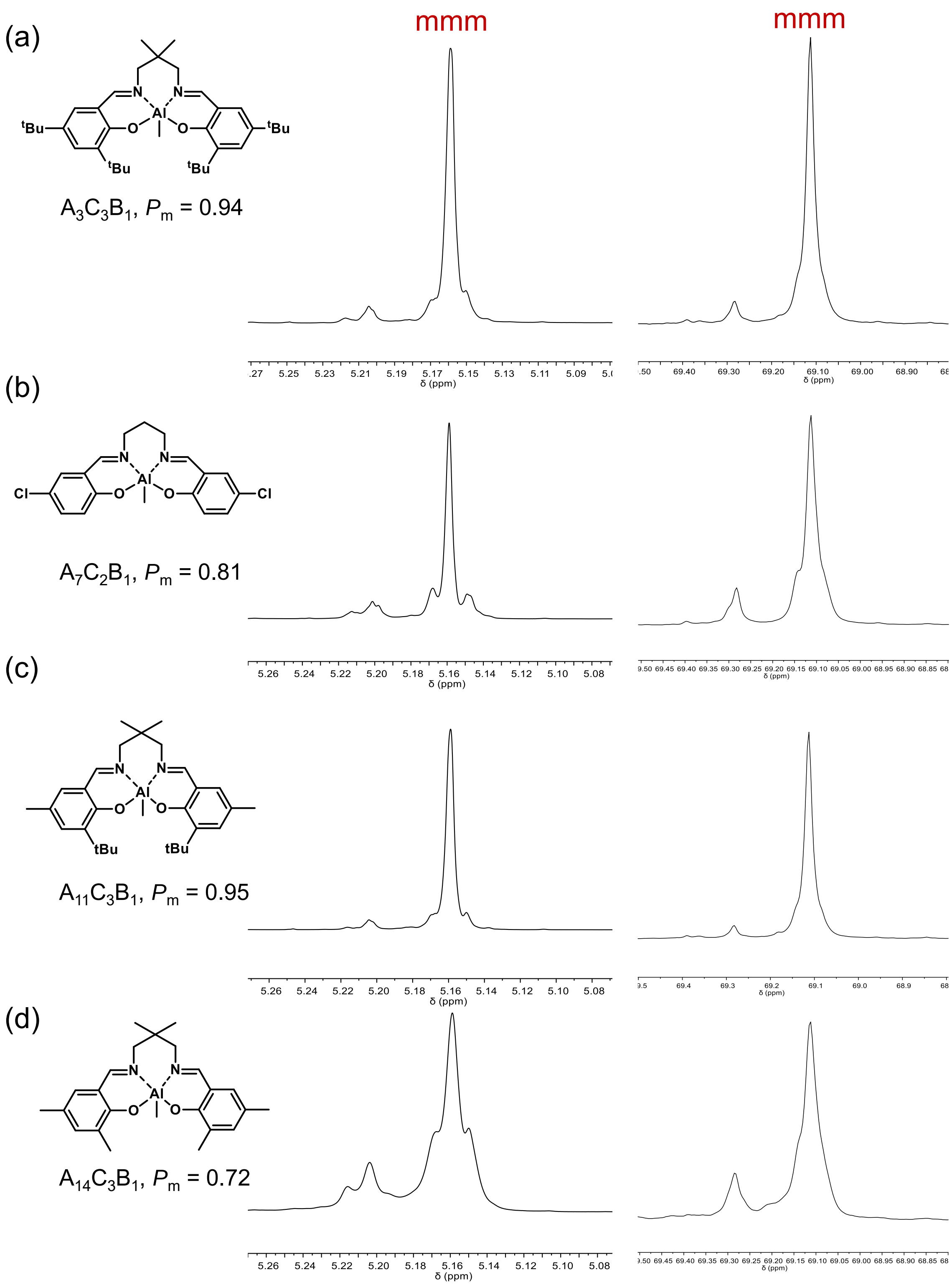

(Data continue on the next page)

(e)

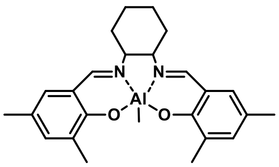

$A_{14}C_5B_1$, P_m = 0.77

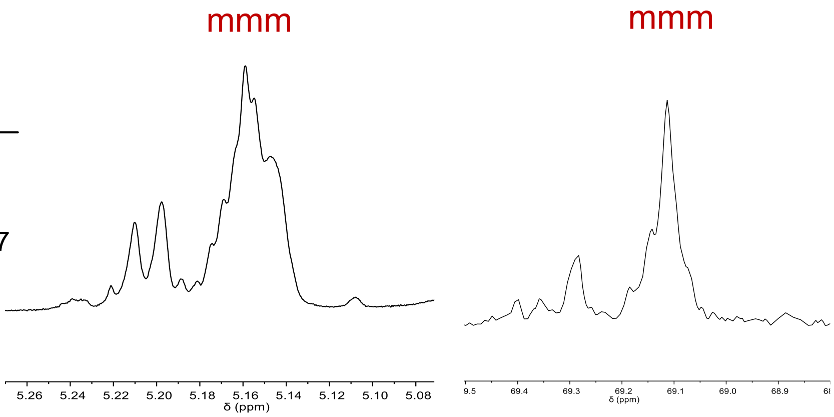

(f)

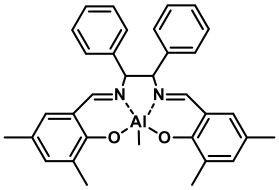

$A_{14}C_{11}B_1$, P_m = 0.77

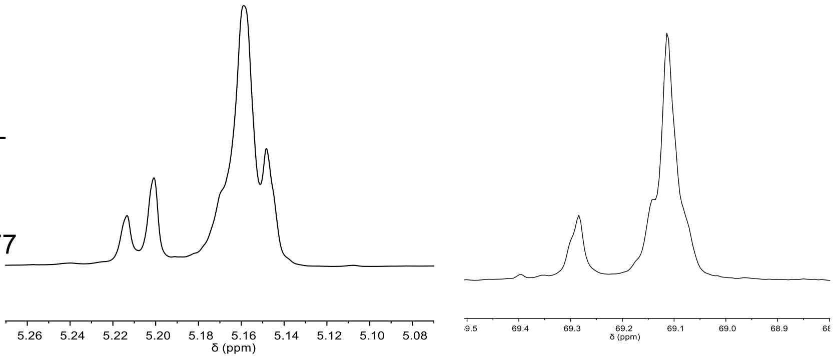

(g)

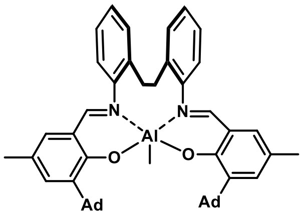

$A_8C_9B_1$, P_m = 0.52

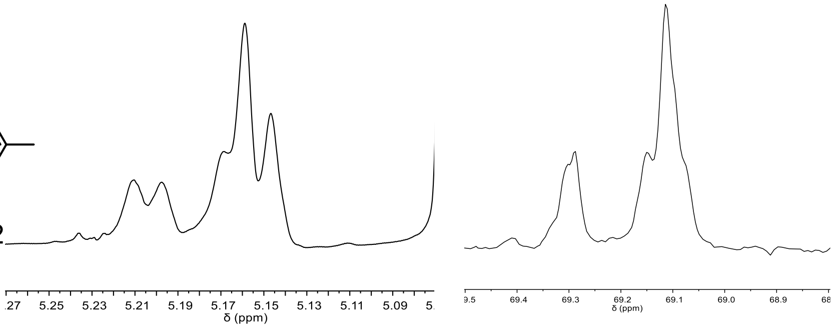

(Data continue on the next page)

(h)

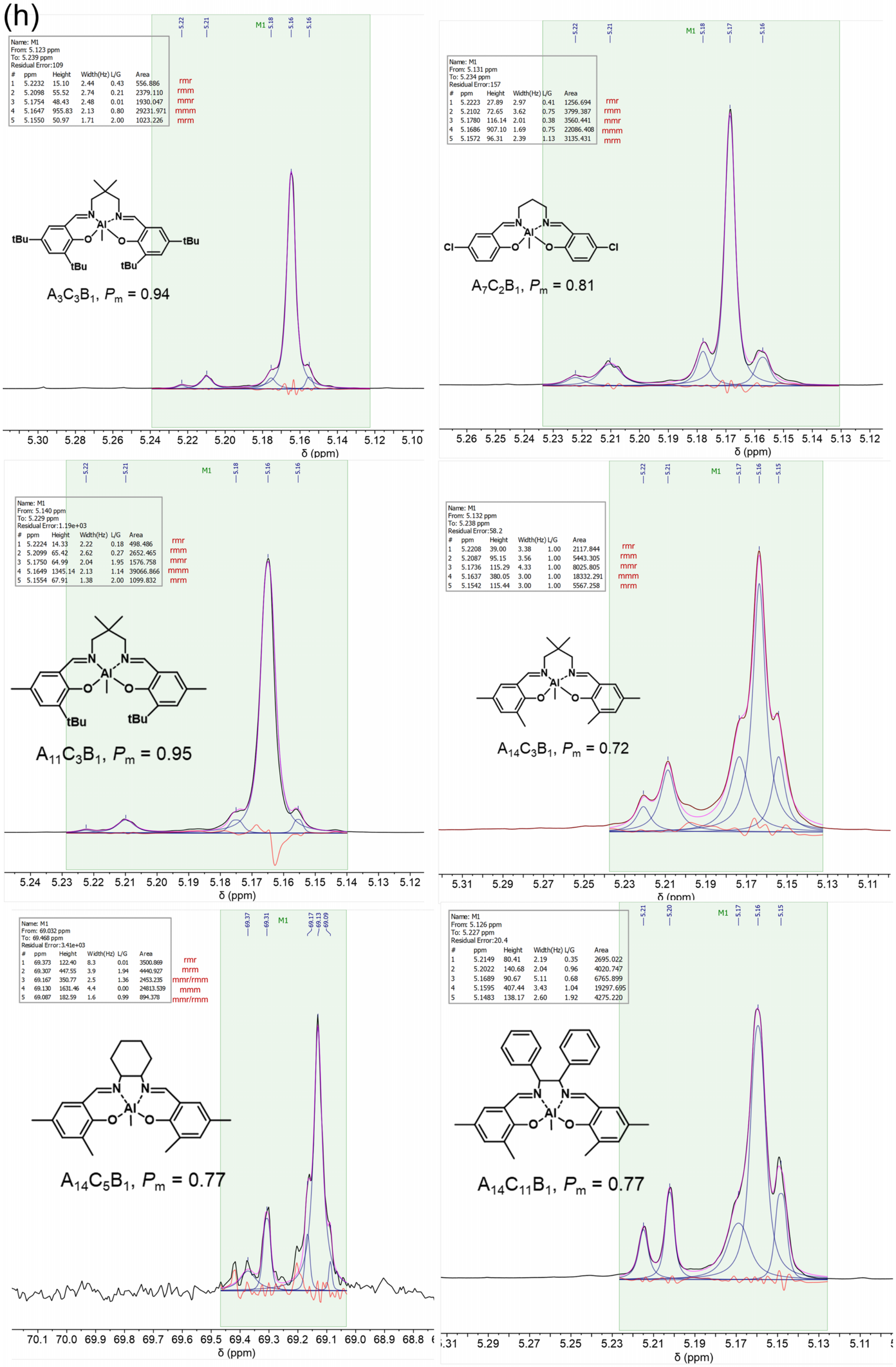

(Data continue on the next page)

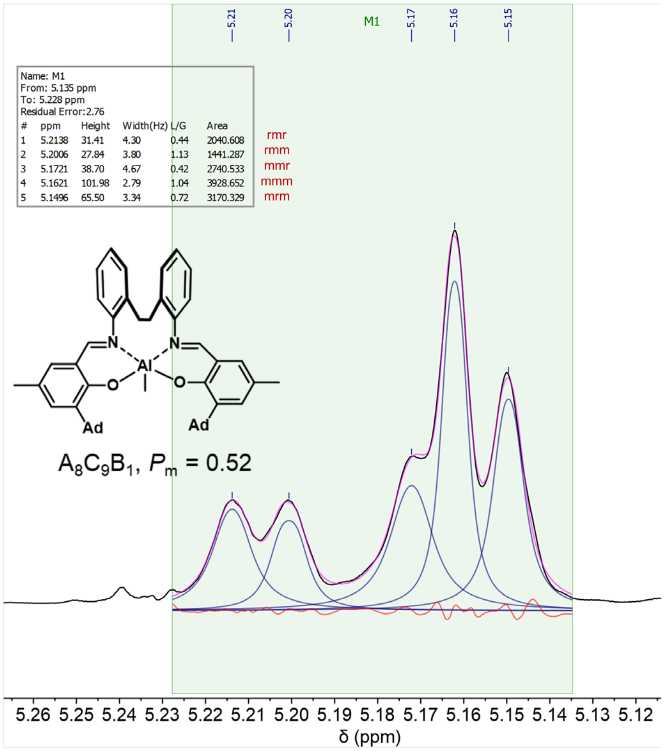

Supplementary Figure 6. (a-g) Homodecoupling ^{1}H NMR (left, 400 MHz, $CDCl_3$) and ^{13}C NMR (right, 125 MHz, $CDCl_3$) of PLA that were prepared by the first-round proposed isotactic Al complexes in Supplementary Table 3. (h) Peak fitting and area integration of the homodecoupling ^{1}H NMR spectra in (a-g). We note that the calculation of the P_m value of the polymerization mediated by ($A_{14}C_5B_1$)AlMe used the ^{13}C spectra, as the conversion of *rac*-LA was low (21.9 %), thereby, the attempt of homodecoupling of the protons in the methine region for this specific sample was not successful. We thus used the calculation of the methine region in ^{13}C NMR spectrum instead.

The calculation of the P_m value in the homodecoupling ^{1}H NMR spectra of the methine region was based on the equations of tetrad probabilities following the Bernoullian Statistics.[58] We compared the results of different calculation methods, and found that using the peak area of *mrm*, whose probability equals to $P_r/2$ (in some references written as $(P_r^2+P_rP_m)/2$, which are the same), could give results close to the literature reports (e.g., we tested $A_3C_3B_1$ and our P_m result (0.94) is close to the literature results (0.93)[4]). This is because that (1) the *mrm* peak (the lowest chemical shift values) is only affected by the *mmm* peak, while other peaks often overlap with multiple tetrad peaks in the homodecoupling ^{1}H NMR, (2) the mathematical calculation is straightforward, calculations of other peaks often giving two-root solutions (as solving quadratic equations).

Note that for enantiomorphic-site control enchainment, the NMR tetrad peaks would be different from chain-end control mechanism, as the stereoerror would be corrected by the chiral catalyst (ideal enantiomorphic control statistics: *mmr*/*rmm*/*rmr*/*mrm* = 1/1/1/2); and we did not observe such NMR difference.

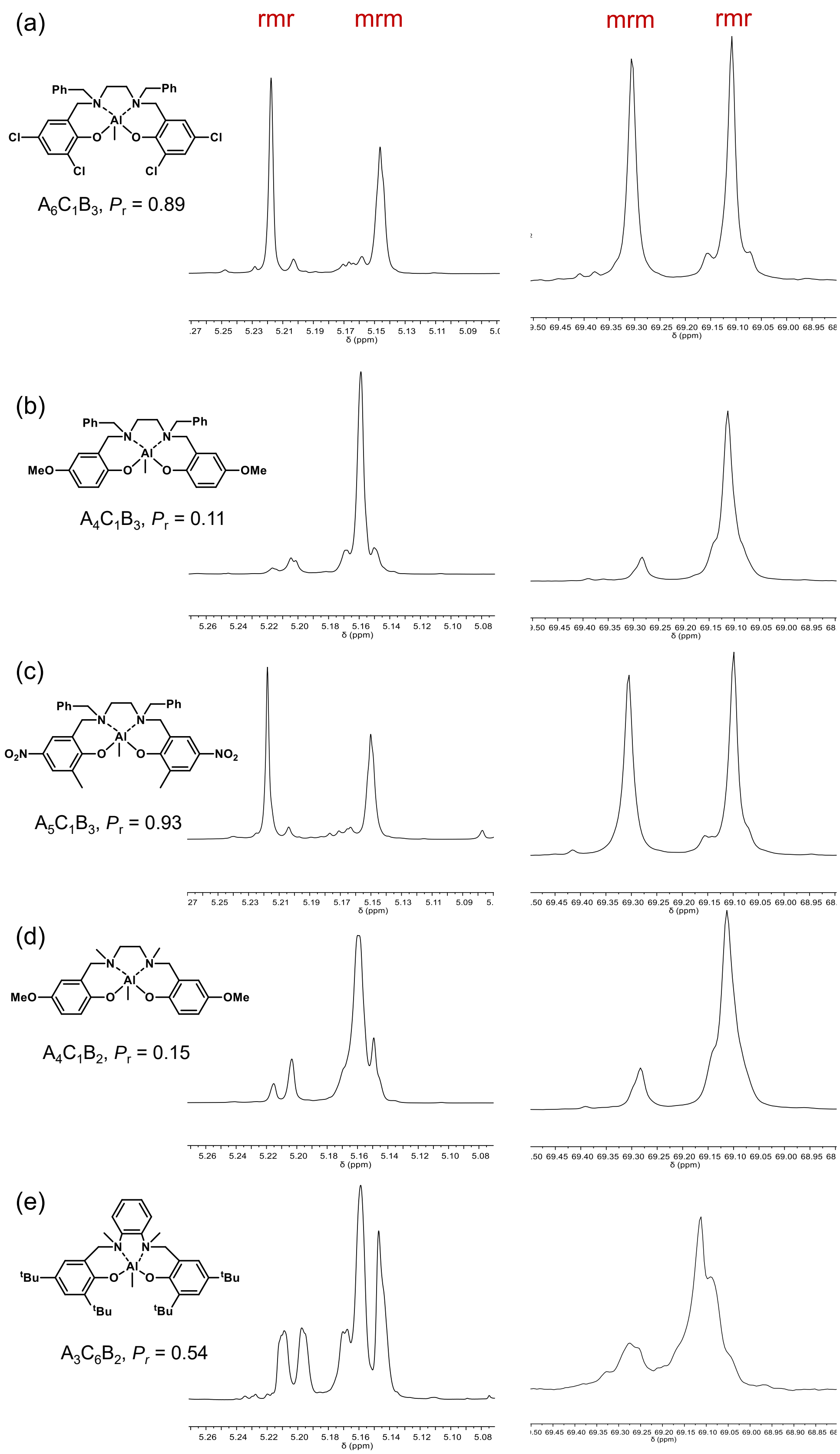

(Data continue on the next page)

(f)

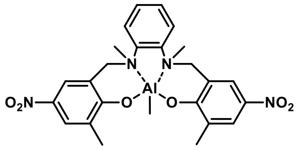

$A_5C_6B_2$, P_r = 0.83

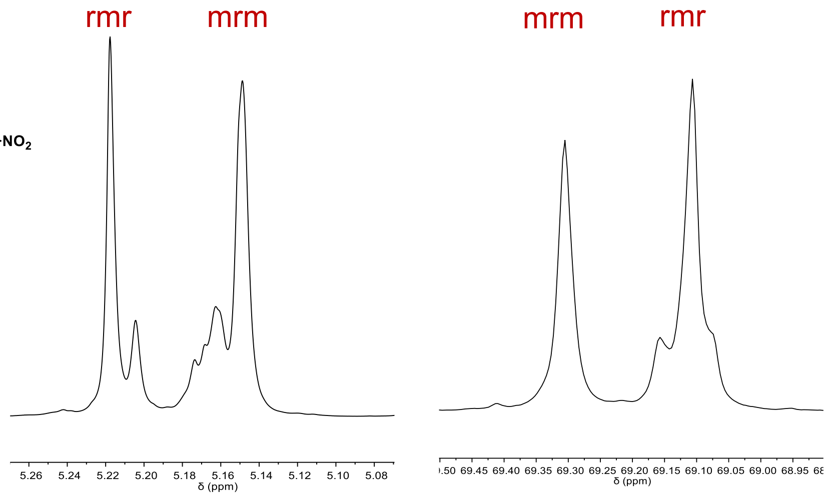

(g)

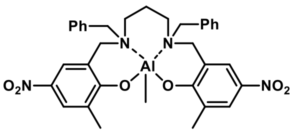

$A_5C_2B_3$, P_r = 0.52

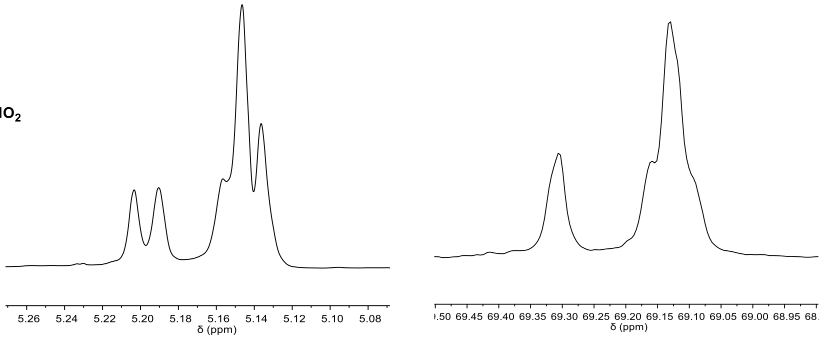

(h)

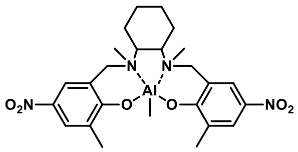

$A_5C_5B_2$, P_r = 0.74

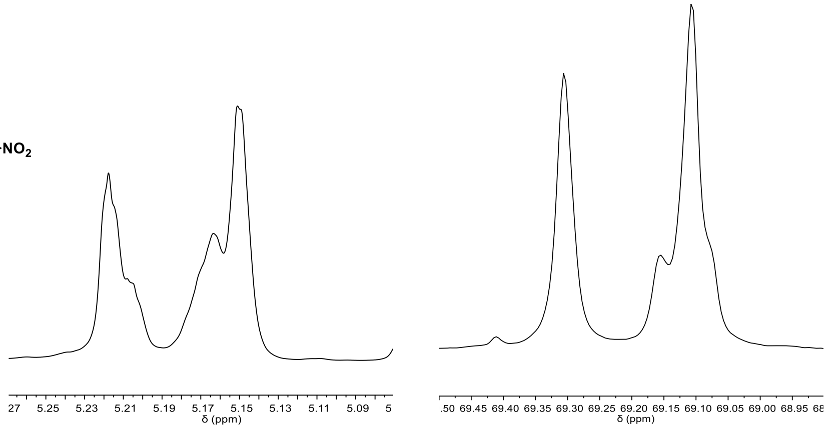

(i)

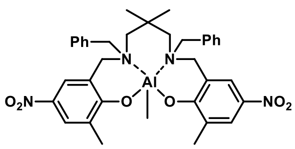

$A_5C_3B_3$, P_r = 0.45

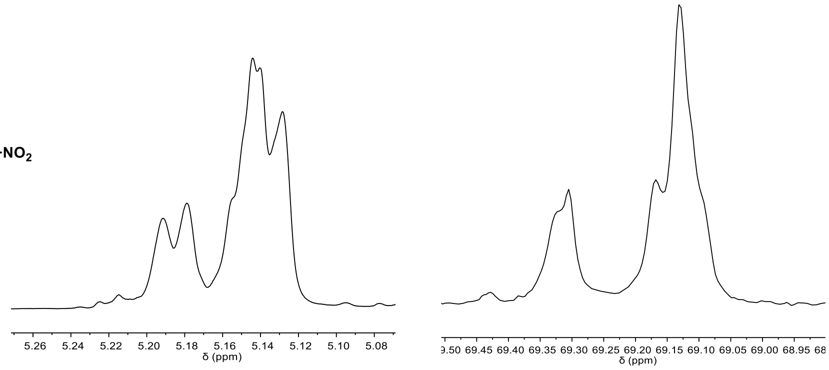

(Data continue on the next page)

(j)

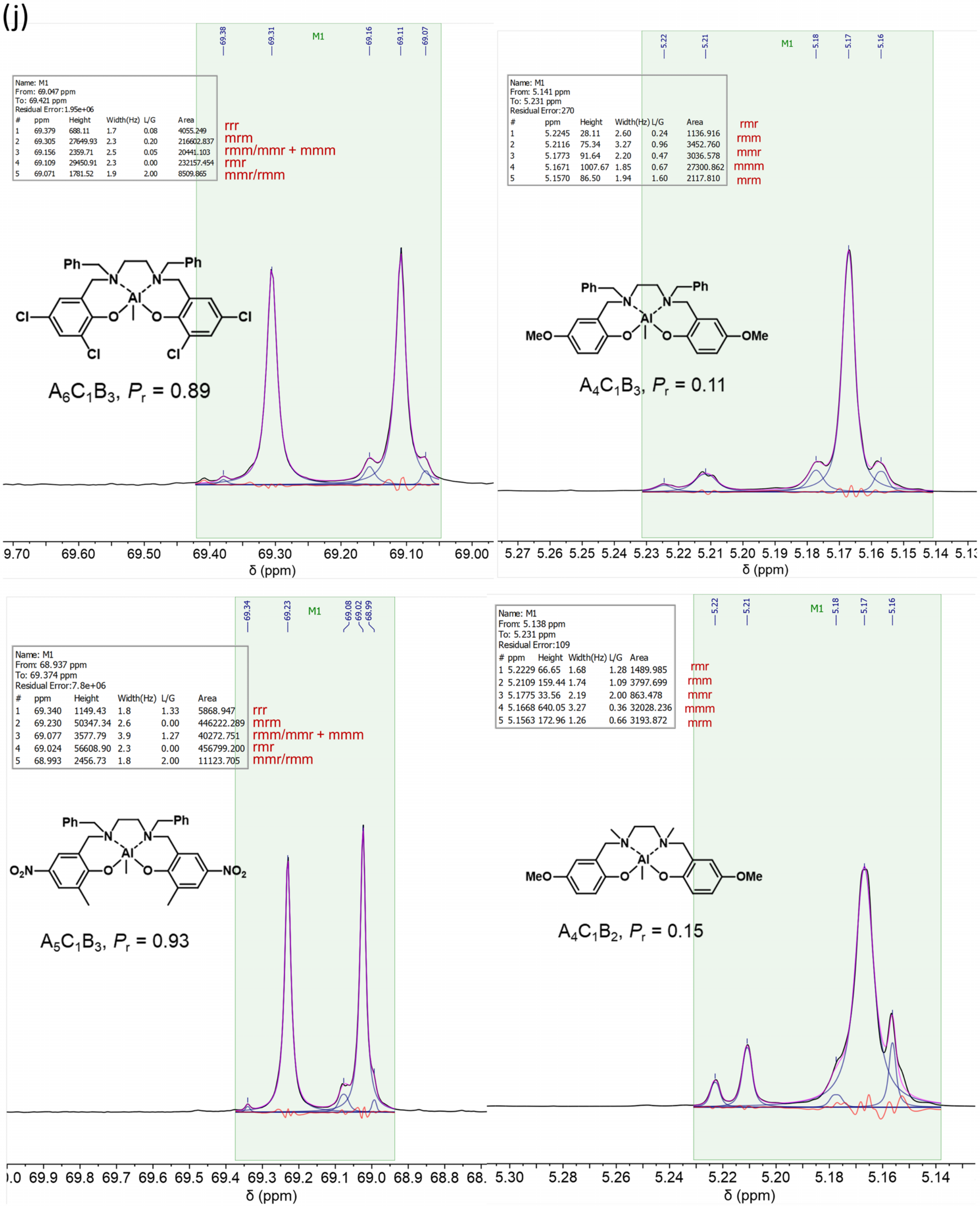

(Data continue on the next page)

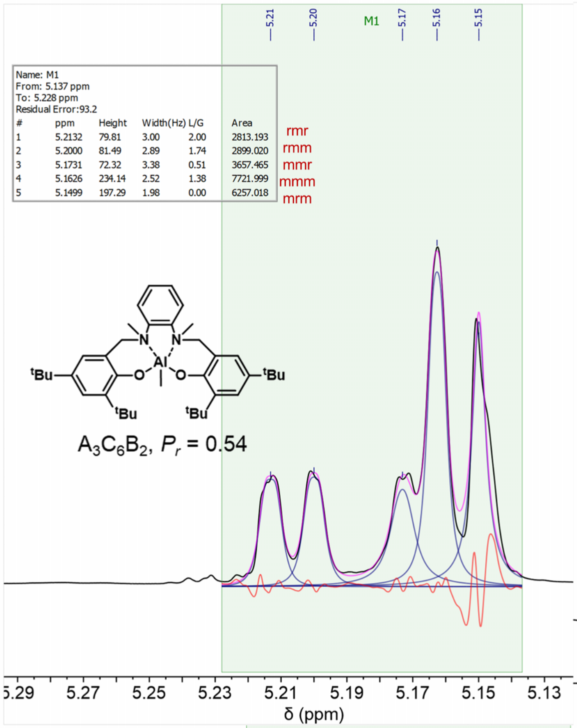

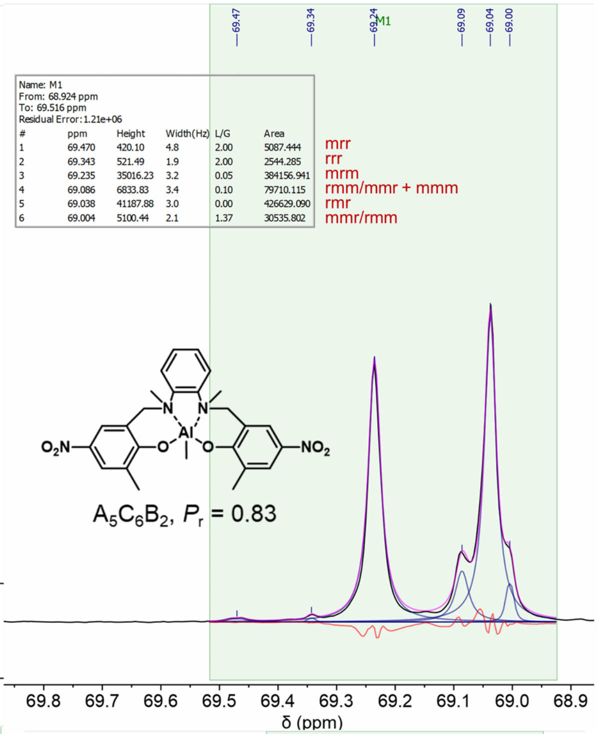

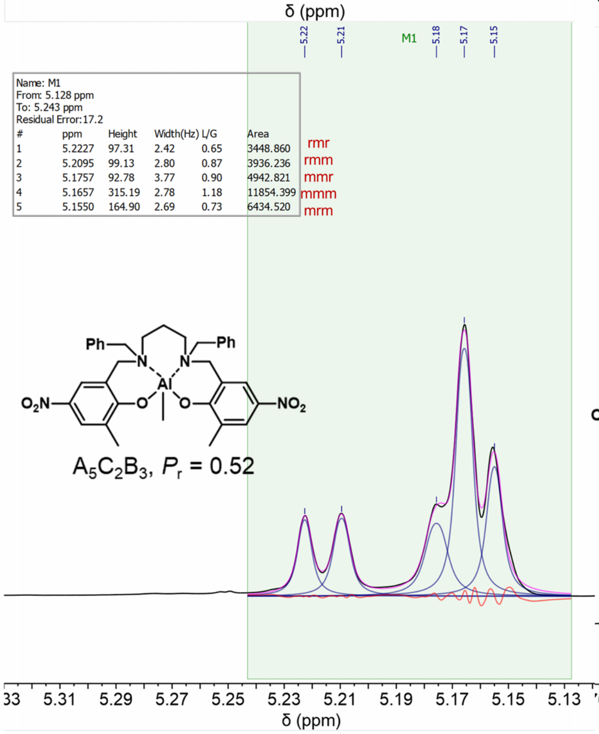

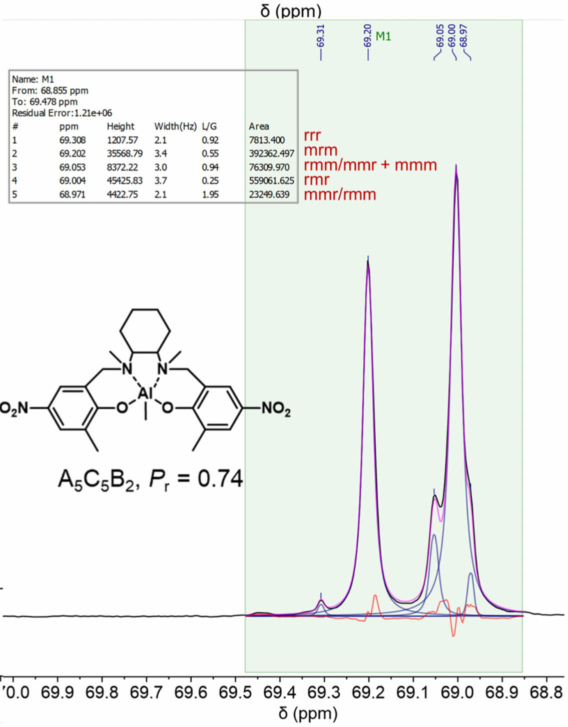

(Data continue on the next page)

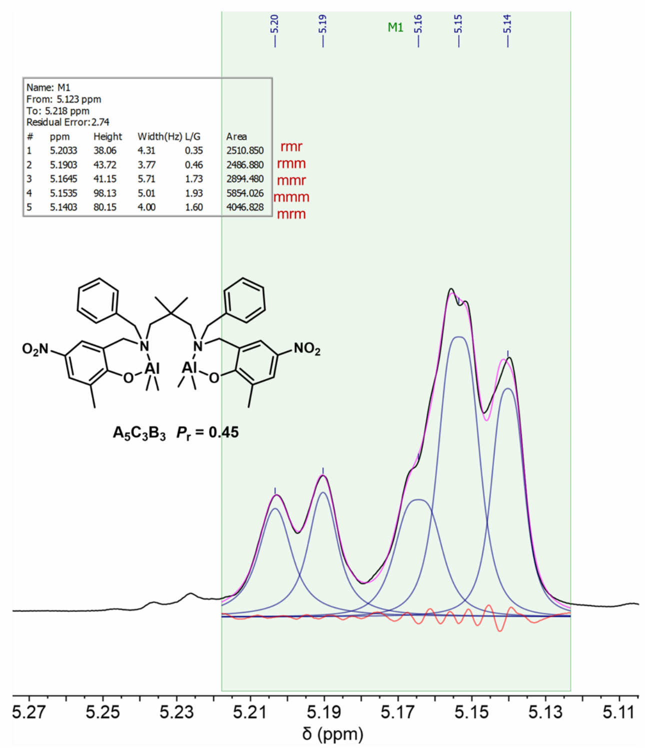

Supplementary Figure 7. (a-i) Homodecoupling ^{1}H NMR (left, 400 MHz, $CDCl_3$) and ^{13}C NMR (right, 125 MHz, $CDCl_3$) of PLA that were prepared by the first-round proposed heterotactic Al complexes in Supplementary Table 4. (j) Peak fitting and area integration of the homodecoupling ^{1}H NMR spectra in (a-i) to calculate P_r values. We note that the calculation of the P_r values of the polymerization mediated by Al complexes with the ligands $A_4C_1B_3$, $A_4C_1B_2$, $A_3C_6B_2$, $A_5C_2B_3$, and $A_5C_2B_3$ used the homodecoupling ^{1}H spectra, because the P_r values were low. Typically, poly(*rac*-LA) with a high P_r value would show two major tetrad peaks (*rmr* and *mrm*) in homodecoupling ^{1}H and ^{13}C NMR spectra, and above ligands exhibited strong *mmm* tetrad peak in their NMR spectra. Thus we used the homodecoupling ^{1}H spectra to calculate the P_m value and then derived the P_r value.

The calculation of the P_r value in the ^{13}C NMR spectrum of the methine region was based on the equations of tetrad probabilities following the Bernoullian Statistics.[58] We did not used the homodecoupling ^{1}H NMR spectra for the calculation because the tetrad *mrm* peak in heterotactic PLA was mixed with *mmm* and *mmr* peaks; on the other hand the *mrm* peak in ^{13}C NMR spectra methine region was dominant and only affected by trace *rrr* peak. Using $(A_6Y_1B_3)$Al complex as an example, we found that the P_r value calculated based on the *mrm* peak in ^{13}C NMR was 0.89, which was close to the reported value P_r (0.96, although we could not repeat that high value); while the P_r value calculated based on the *mrm* peak in ^{1}H NMR spectra was 0.86 (and the calculation became more difficult when P_r value got lower). The reason that we did not calculate the P_r value based on the *rmr* peak was because the peak was influenced by *mmm*, *mmr*, *rmm* peaks, and often overestimated the P_r values.

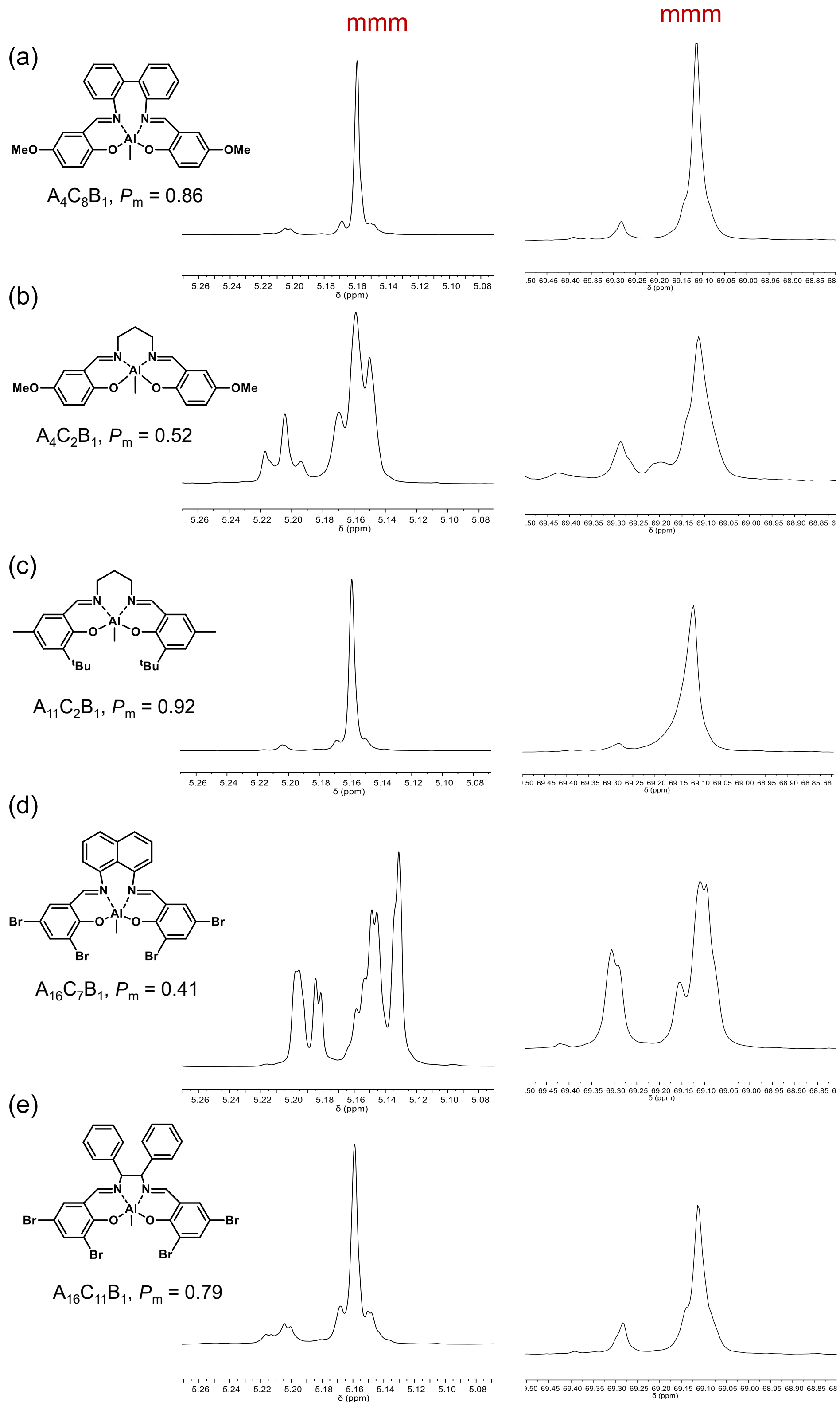

(Data continue on the next page)

(f)

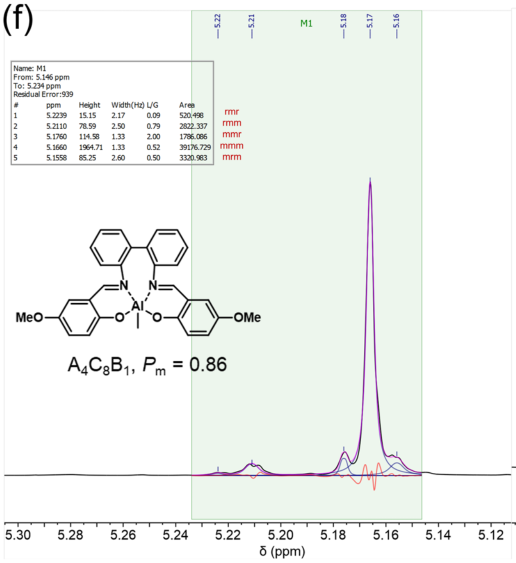
Name: M1
From: 5.146 ppm
To: 5.234 ppm
Residual Error:939
ppm Height Width(Hz) L/G Area
1 5.2239 15.15 2.17 0.09 520.498 rmr
2 5.2110 78.59 2.50 0.79 2822.337 rmm
3 5.1760 114.58 1.33 2.00 1786.086 mmr
4 5.1660 1964.71 1.33 0.52 39176.729 mmm
5 5.1558 85.25 2.60 0.50 3320.983 mrm
M1
MeO
OMe
N
Al
O
A4C8B1, Pm = 0.86
δ (ppm)

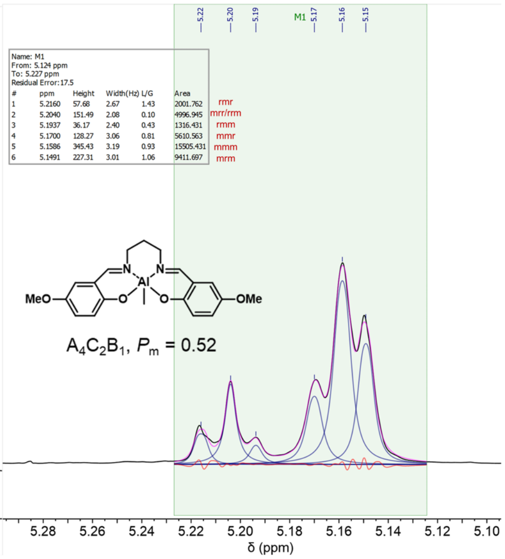
Name: M1
From: 5.124 ppm
To: 5.227 ppm
Residual Error:17.5
ppm Height Width(Hz) L/G Area
1 5.2160 57.68 2.67 1.43 2001.762 rmr
2 5.2040 151.49 2.08 0.10 4996.945 mrr/rrm
3 5.1937 36.17 2.40 0.43 1316.431 rmm
4 5.1700 128.27 3.06 0.81 5610.563 mmr
5 5.1586 345.43 3.19 0.93 15505.431 mmm
6 5.1491 227.31 3.01 1.06 9411.697 mrm
M1
MeO
OMe
N
Al
O
A4C2B1, Pm = 0.52
δ (ppm)

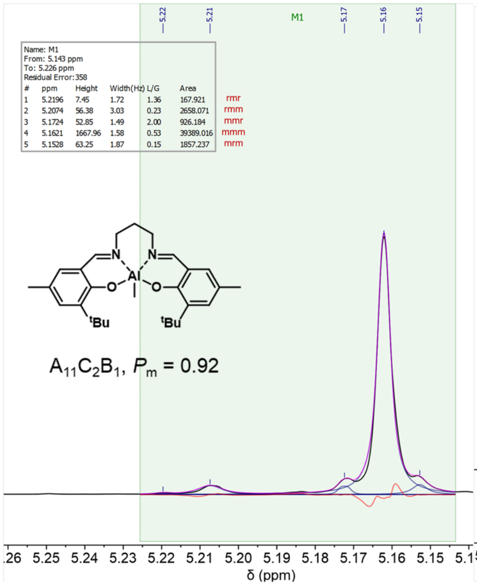
Name: M1
From: 5.143 ppm
To: 5.226 ppm
Residual Error:358
ppm Height Width(Hz) L/G Area
1 5.2196 7.45 1.72 1.36 167.921 rmr
2 5.2074 56.38 3.03 0.23 2658.071 rmm
3 5.1724 52.85 1.49 2.00 926.184 mmr
4 5.1621 1667.96 1.58 0.53 39389.016 mmm
5 5.1528 63.25 1.87 0.15 1857.237 mrm
M1
N
Al
O
tBu
A11C2B1, Pm = 0.92
δ (ppm)

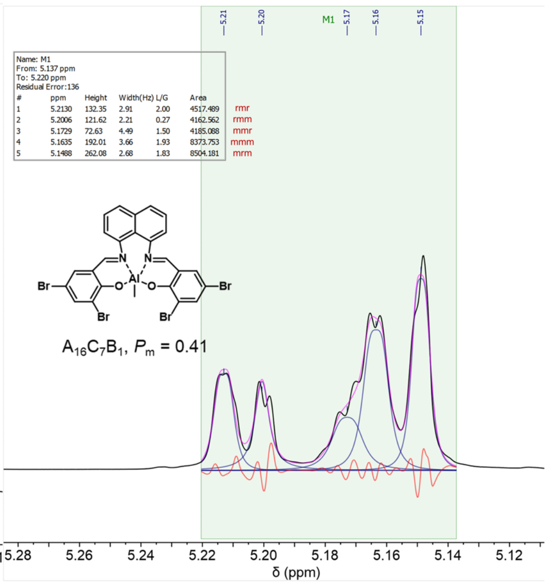
Name: M1
From: 5.137 ppm
To: 5.220 ppm
Residual Error:136
ppm Height Width(Hz) L/G Area
1 5.2130 132.35 2.91 2.00 4517.489 rmr
2 5.2006 121.62 2.21 0.27 4162.562 rmm
3 5.1729 72.63 4.49 1.50 4185.088 mmr
4 5.1635 192.01 3.66 1.93 8373.753 mmm
5 5.1488 262.08 2.68 1.83 8504.181 mrm
M1
Br
N
Al
O
A16C7B1, Pm = 0.41
δ (ppm)

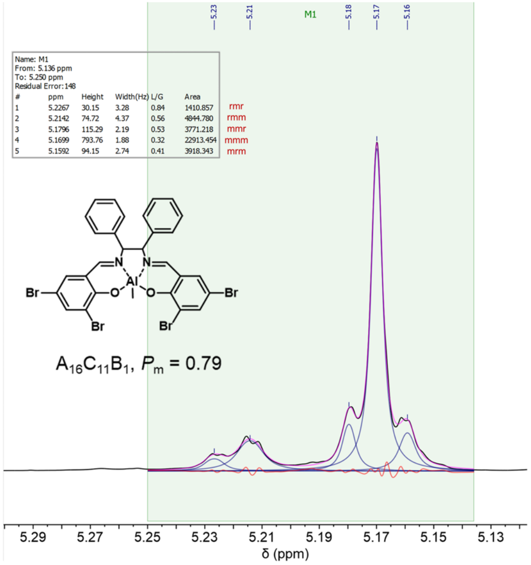
Name: M1
From: 5.136 ppm
To: 5.250 ppm
Residual Error:148
ppm Height Width(Hz) L/G Area
1 5.2267 30.15 3.28 0.84 1410.857 rmr
2 5.2142 74.72 4.37 0.56 4844.780 rmm
3 5.1796 115.29 2.19 0.53 3771.218 mmr
4 5.1699 793.76 1.88 0.32 22913.454 mmm
5 5.1592 94.15 2.74 0.41 3918.343 mrm
M1
Br
N
Al
O
A16C11B1, Pm = 0.79
δ (ppm)

Supplementary Figure 8. (a-e) Homodecoupling ^{1}H NMR (left, 400 MHz, $CDCl_3$) and ^{13}C NMR (right, 125 MHz, $CDCl_3$) of PLA that were prepared by the second-round proposed isotactic Al complexes in Supplementary Table 5. (f) Peak fitting and area integration of the homodecoupling ^{1}H NMR spectra in (a-e) to calculate P_m values.

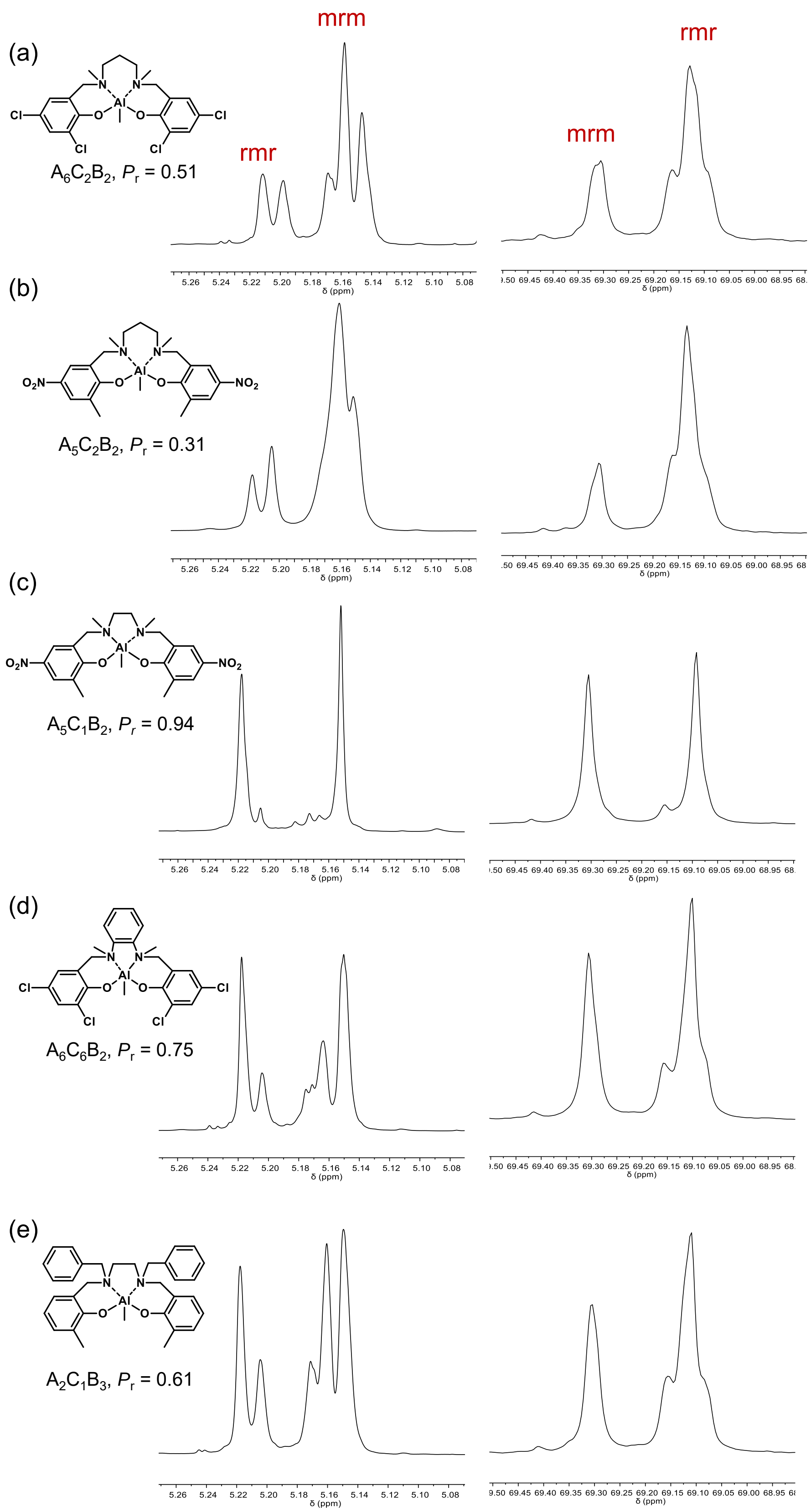

(Data continue on the next page)

(f)

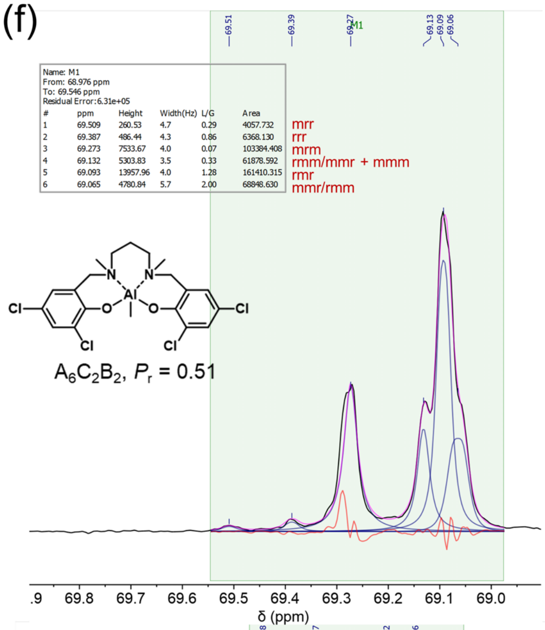
69.51
69.39
69.27
69.13
69.09
69.06
M1
Name: M1
From: 68.976 ppm
To: 69.546 ppm
Residual Error: 6.31e+05
ppm Height Width(Hz) L/G Area
1 69.509 260.53 4.7 0.29 4057.732
2 69.387 486.44 4.3 0.86 6368.130
3 69.273 7533.67 4.0 0.07 103384.408
4 69.132 5303.83 3.5 0.33 61878.592
5 69.093 13957.96 4.0 1.28 161410.315
6 69.065 4780.84 5.7 2.00 68848.630
mrr
rrr
mrm
rmm/mmr + mmm
rmr
mmr/rmm
Cl
N
Al
O
$A_6C_2B_2$, P_r = 0.51
.9 69.8 69.7 69.6 69.5 69.4 69.3 69.2 69.1 69.0
δ (ppm)

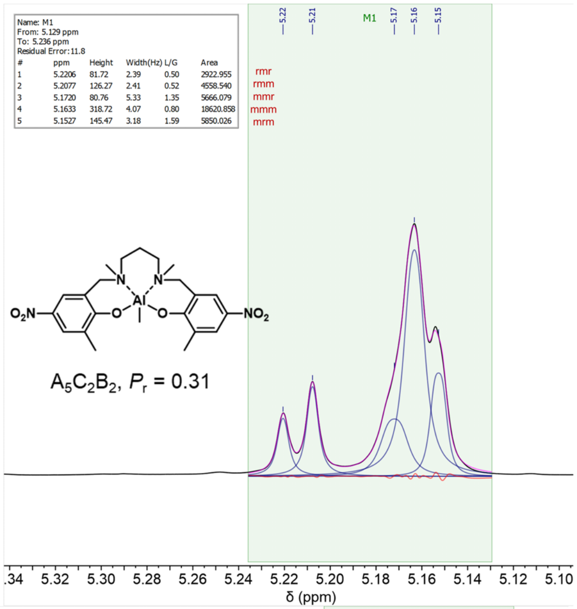
5.22
5.21
5.17
5.16
5.15
M1
Name: M1
From: 5.129 ppm
To: 5.236 ppm
Residual Error: 11.8
ppm Height Width(Hz) L/G Area
1 5.2206 81.72 2.39 0.50 2922.955
2 5.2077 126.27 2.41 0.52 4558.540
3 5.1720 80.76 5.33 1.35 5666.079
4 5.1633 318.72 4.07 0.80 18620.858
5 5.1527 145.47 3.18 1.59 5850.026
rmr
rmm
mmr
mmm
mrm
O_2N
NO_2
N
Al
O
$A_5C_2B_2$, P_r = 0.31
.34 5.32 5.30 5.28 5.26 5.24 5.22 5.20 5.18 5.16 5.14 5.12 5.10
δ (ppm)

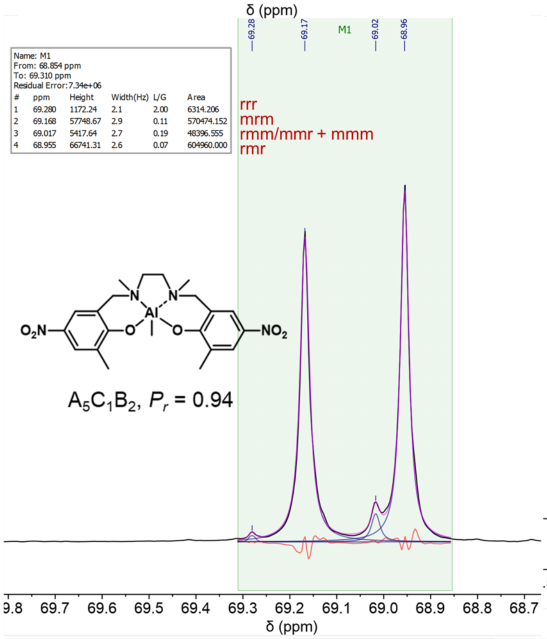
69.28
69.17
M1
69.02
68.96
Name: M1
From: 68.854 ppm
To: 69.310 ppm
Residual Error: 7.34e+06
ppm Height Width(Hz) L/G Area
1 69.280 1172.24 2.1 2.00 6314.206
2 69.168 57748.67 2.9 0.11 570474.152
3 69.017 5417.64 2.7 0.19 48396.555
4 68.955 66741.31 2.6 0.07 604960.000
rrr
mrm
rmm/mmr + mmm
rmr
O_2N
NO_2
N
Al
O
$A_5C_1B_2$, P_r = 0.94
9.8 69.7 69.6 69.5 69.4 69.3 69.2 69.1 69.0 68.9 68.8 68.7
δ (ppm)

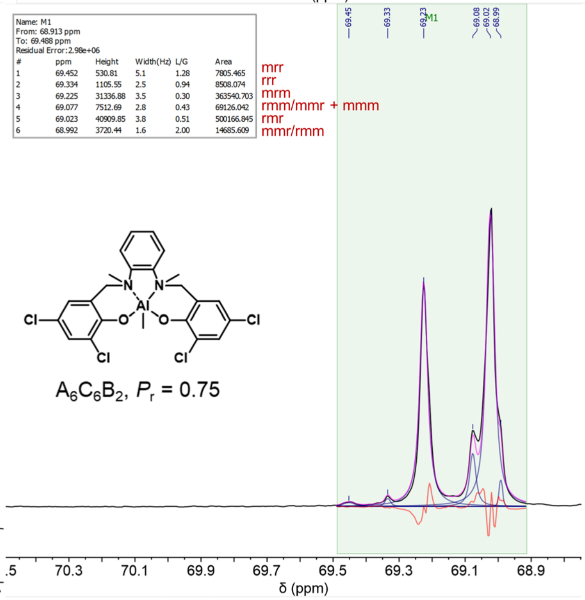
69.45
69.33
69.23
M1
69.08
69.02
68.99
Name: M1
From: 68.913 ppm
To: 69.488 ppm
Residual Error: 2.98e+06
ppm Height Width(Hz) L/G Area
1 69.452 530.81 5.1 1.28 7805.465
2 69.334 1105.55 2.5 0.94 8508.074
3 69.225 31336.88 3.5 0.30 363540.703
4 69.077 7512.69 2.8 0.43 69126.042
5 69.023 40909.85 3.8 0.51 500166.845
6 68.992 3720.44 1.6 2.00 14685.609
mrr
rrr
mrm
rmm/mmr + mmm
rmr
mmr/rmm
Cl
N
Al
O
$A_6C_6B_2$, P_r = 0.75
.5 70.3 70.1 69.9 69.7 69.5 69.3 69.1 68.9
δ (ppm)

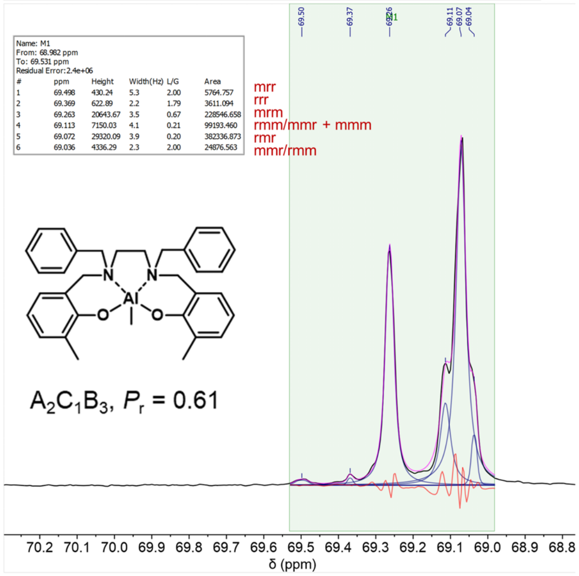
69.50
69.37
69.26
M1
69.11
69.07
69.04
Name: M1
From: 68.982 ppm
To: 69.531 ppm
Residual Error: 2.4e+06
ppm Height Width(Hz) L/G Area
1 69.498 430.24 5.3 2.00 5764.757
2 69.369 622.89 2.2 1.79 3611.094
3 69.263 20643.67 3.5 0.67 228546.658
4 69.113 7150.03 4.1 0.21 99193.460
5 69.072 29320.09 3.9 0.20 382336.873
6 69.036 4336.29 2.3 2.00 24876.563
mrr
rrr
mrm
rmm/mmr + mmm
rmr
mmr/rmm
N
Al
O
$A_2C_1B_3$, P_r = 0.61
70.2 70.1 70.0 69.9 69.8 69.7 69.6 69.5 69.4 69.3 69.2 69.1 69.0 68.9 68.8
δ (ppm)

Supplementary Figure 9. (a-e) Homodecoupling ^{1}H NMR (left, 400 MHz, $CDCl_3$) and ^{13}C NMR (right, 125 MHz, $CDCl_3$) of PLA that were prepared by the second-round proposed heterotactic Al complexes in Supplementary Table 6. (f) Peak fitting and area integration of the homodecoupling ^{1}H NMR spectra in (a-e) to calculate P_r values. We note that for $(A_5C_2B_2)$Al complex, the P_r value seemed to be low based on ^{13}C NMR spectra. Instead, we used homodecoupling ^{1}H NMR to calculate the P_m value that could derive the P_r value.

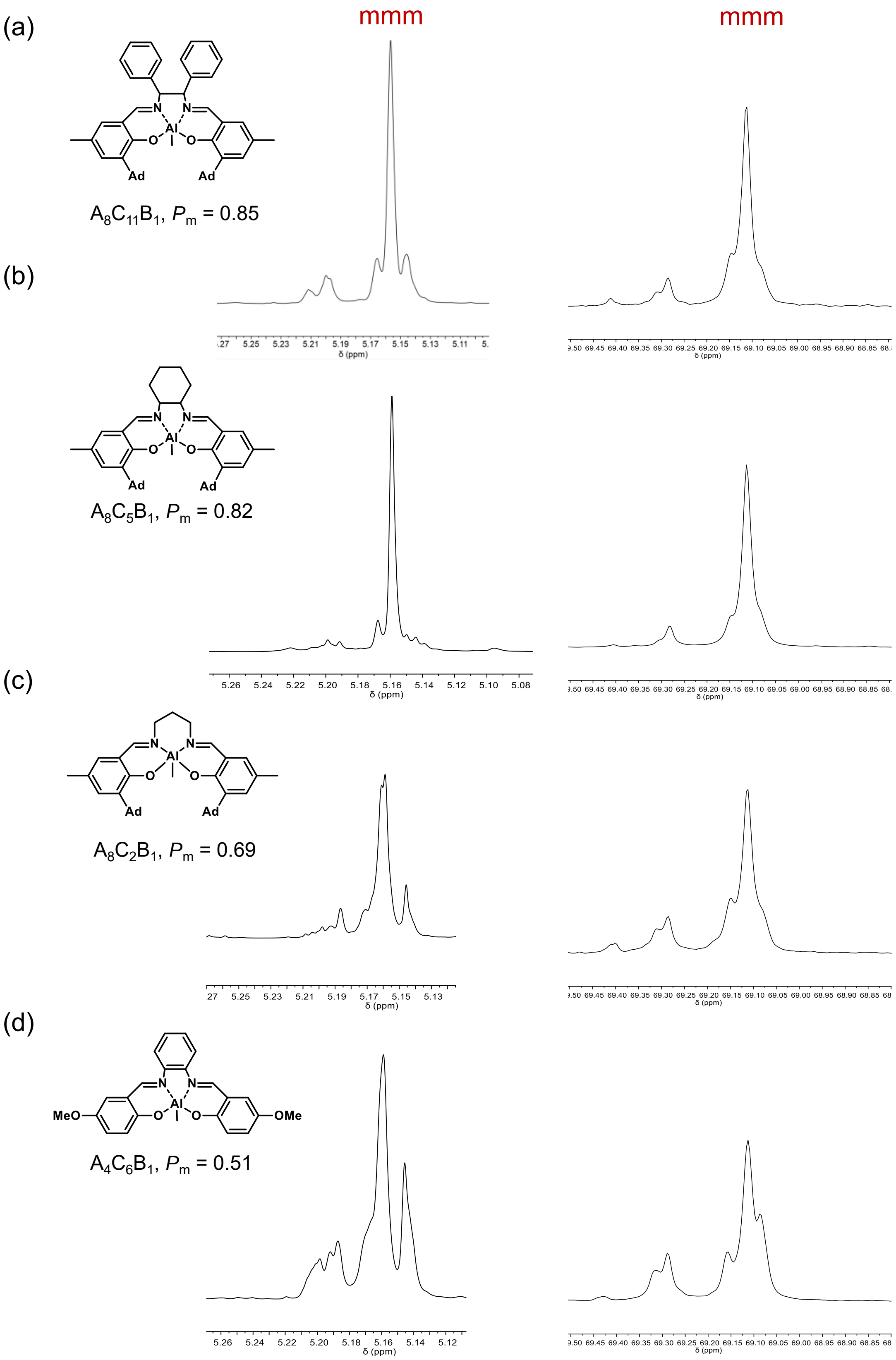

(Data continue on the next page)

(e)

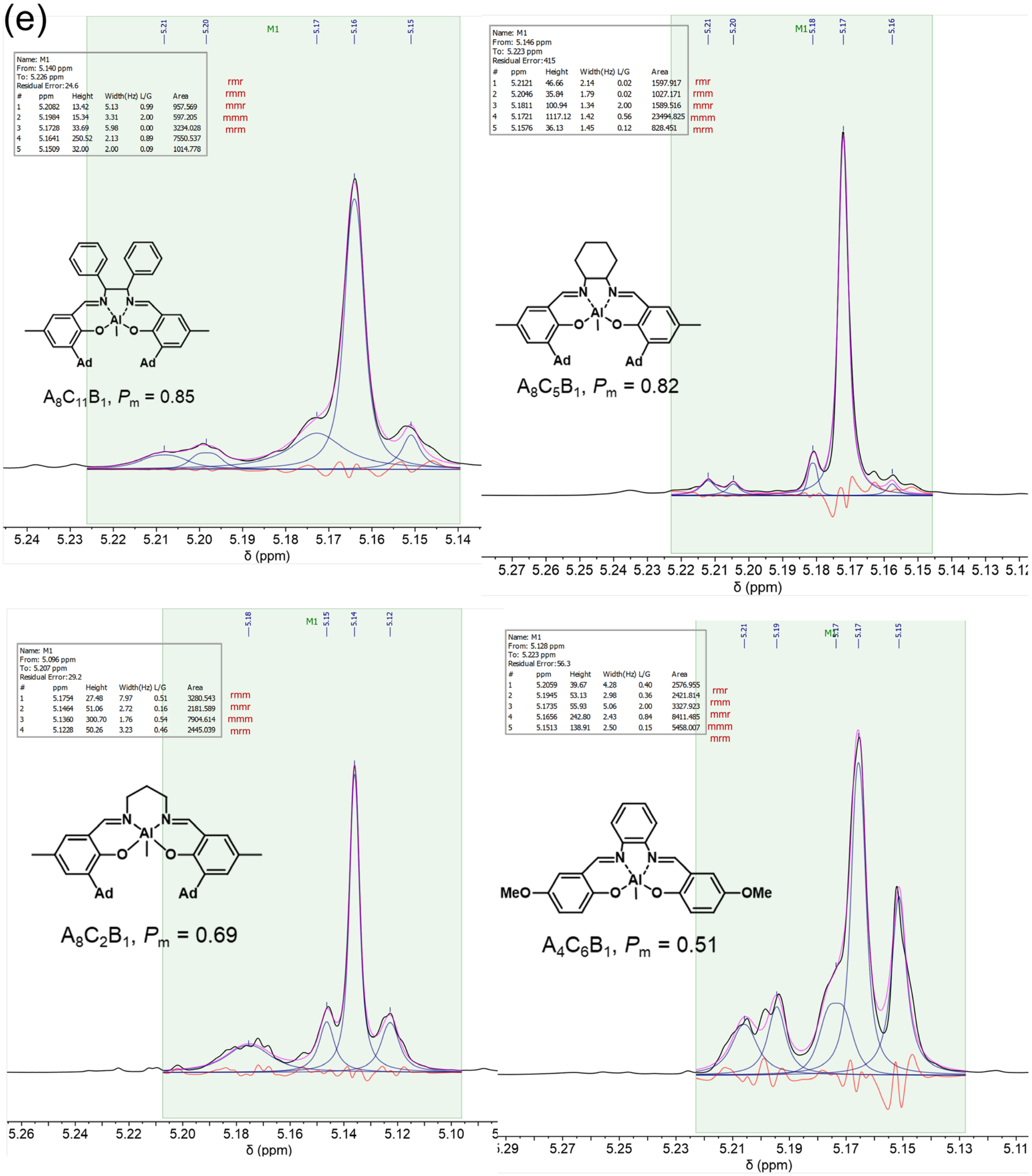

Supplementary Figure 10. (a-d) Homodecoupling ^{1}H NMR (left, 400 MHz, $CDCl_3$) and ^{13}C NMR (right, 125 MHz, $CDCl_3$) of PLA that were prepared by the third-round proposed isotactic Al complexes in Supplementary Table 7. (e) Peak fitting and area integration of the homodecoupling ^{1}H NMR spectra in (a-d) to calculate P_m values.

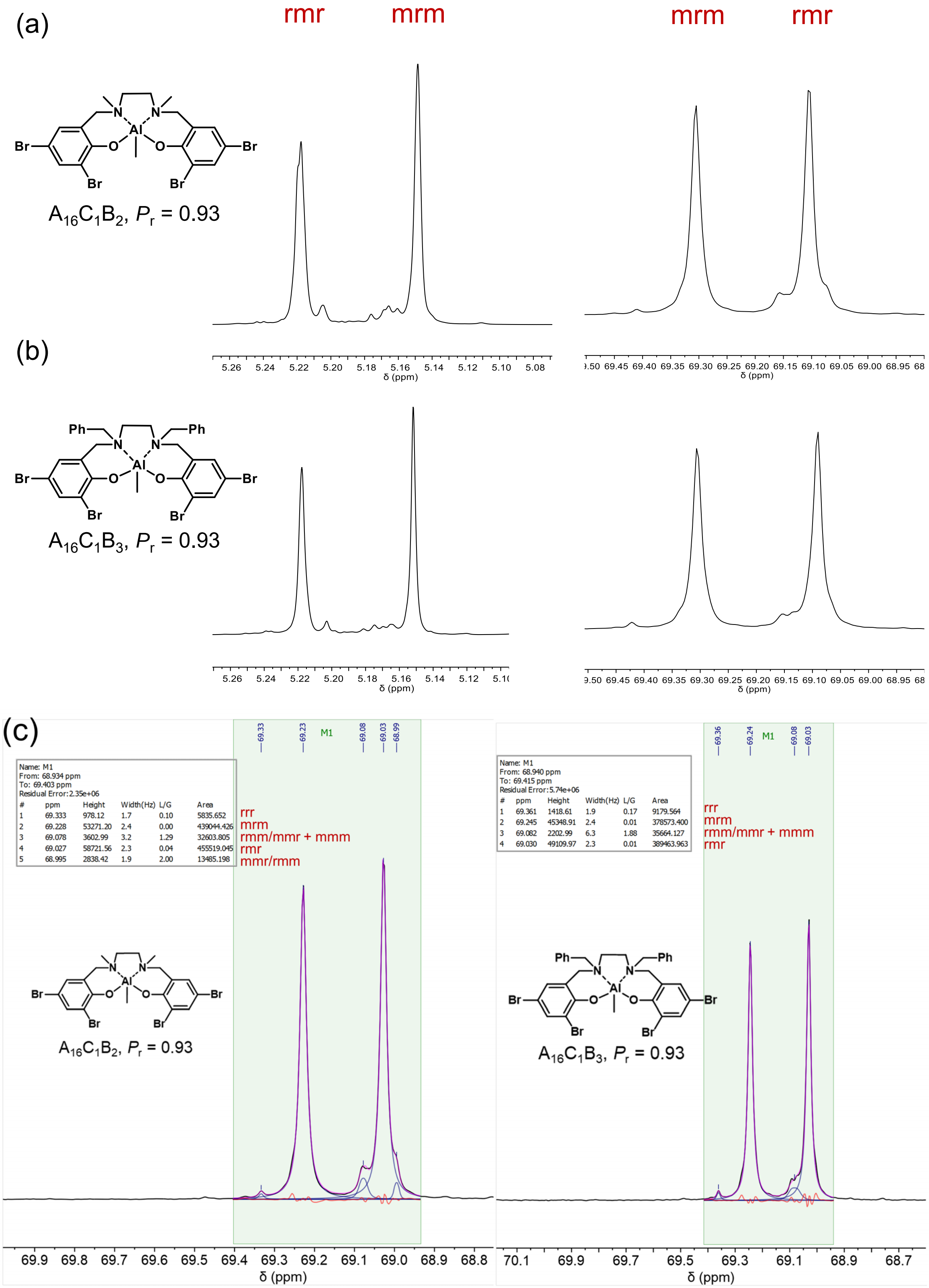

Supplementary Figure 11. (a-b) Homodecoupling ¹H NMR (left, 400 MHz, CDCl3) and ¹³C NMR (right, 125 MHz, CDCl3) of PLA that were prepared by the third-round proposed

heterotactic Al complexes in Supplementary Table 8. (c) Peak fitting and area integration of the methine region in ^{13}C NMR spectra in (a-b) to calculate P_r values.

(a)

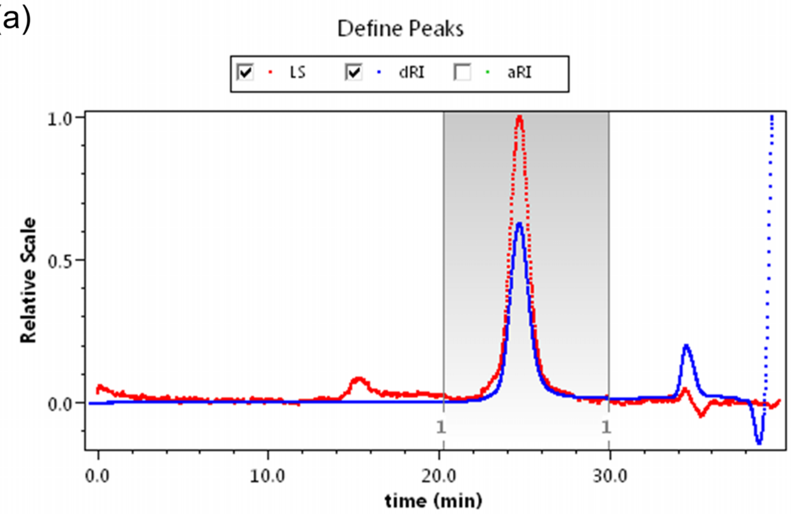

Results

Peak Results

	Peak 1
**Masses**	
Injected Mass (µg)	0.00
Calculated Mass (µg)	1423.86
Mass Recovery (%)	n/a
Mass Fraction (%)	100.0
**Molar mass moments (g/mol)**	
Mn	1.401×10^4 (±5.140%)
Mw	1.443×10^4 (±4.350%)
**Polydispersity**	
Mw/Mn	1.030 (±6.734%)
Mz/Mn	1.502 (±49.823%)

(b)

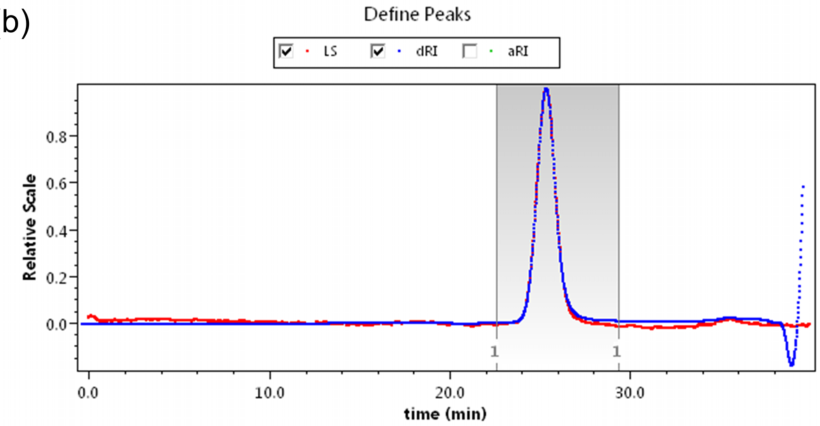

Results

Peak Results

	Peak 1
**Masses**	
Injected Mass (µg)	0.00
Calculated Mass (µg)	1499.37
Mass Recovery (%)	n/a
Mass Fraction (%)	100.0
**Molar mass moments (g/mol)**	
Mn	1.287×10^4 (±3.529%)
Mw	1.301×10^4 (±2.333%)
**Polydispersity**	
Mw/Mn	1.011 (±4.230%)
Mz/Mn	1.017 (±5.895%)

Supplementary Figure 12. The SEC signals in the light scattering (LS) and refractive index (dRI) detectors versus time of (a) stereoblock PLA (Supplementary Table 3, entry 2), and (b) heterotactic PLA (Supplementary Table 8, entry 1). The peak results clearly indicate the unimodal narrow MW distribution of the obtained polymers.

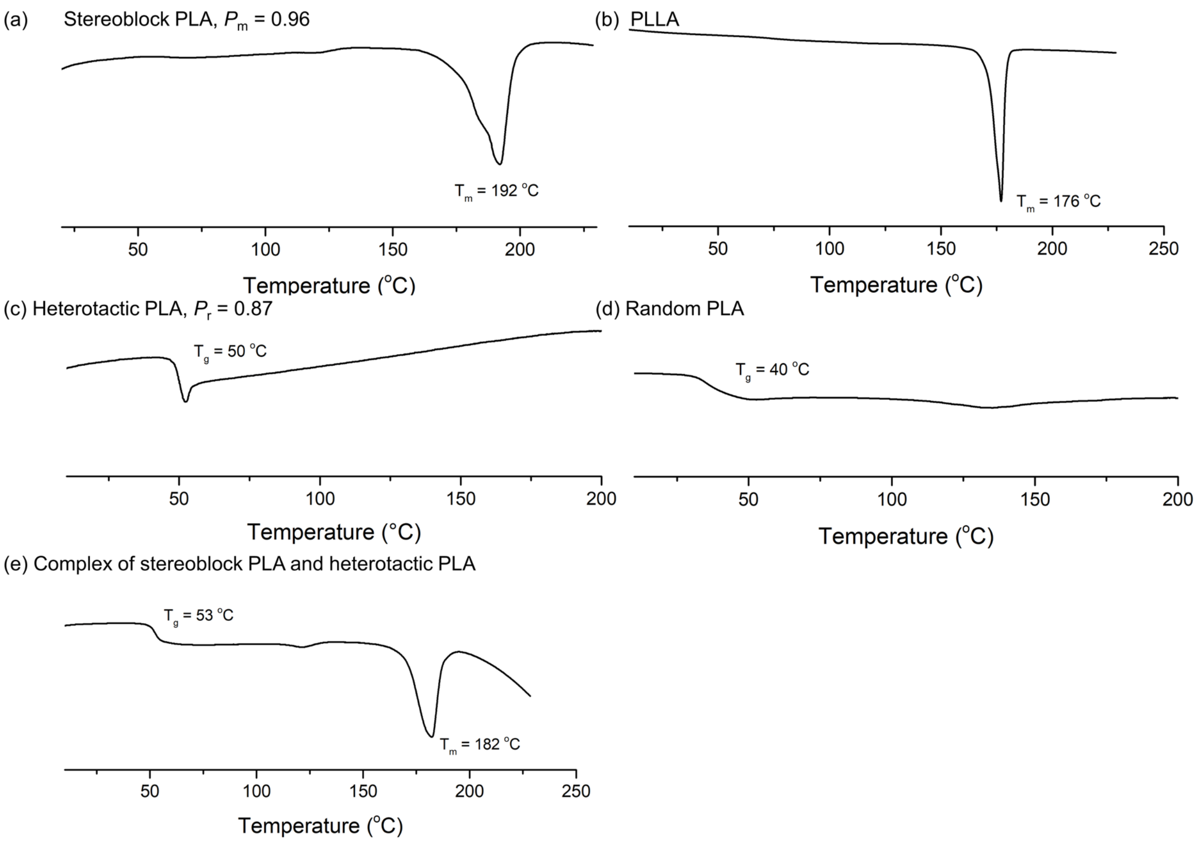

Supplementary Figure 13. DSC measurements of T_gs and T_ms of PLAs with various microstructures as shown in Supplementary Table 9: (a) stereoblock PLA (Supplementary Table 9, entry 1), (b) poly(L-LA) (Supplementary Table 9, entry 3), (c) poly(*ht*-LA) (Supplementary Table 9, entry 2), (d) poly(*r*-LA) (Supplementary Table 9, entry 4), (e) poly(*sb*-LA) + poly(*ht*-LA) (1/1) (Supplementary Table 9, entry 5).

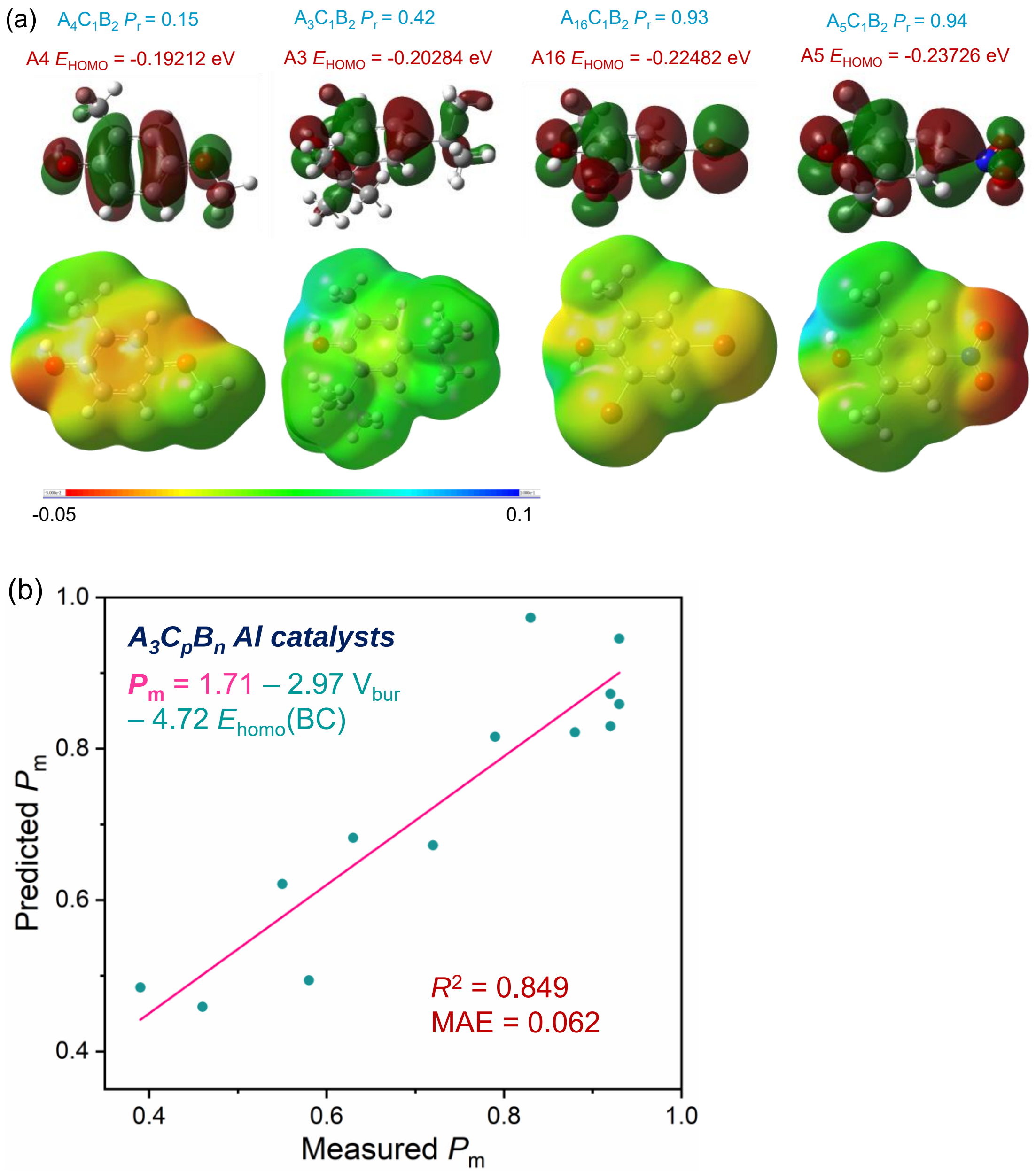

Supplementary Figure 14. (a) Computed E_{HOMO}s and electrostatic potential maps of various fragment A_m. The electronic effects in A_m fragments significantly impact the stereoselectivity of the Al complexes. (b) Multivariate linear regression model correlating V_{bur} of the ligand and E_{HOMO} of B_nC_p fragments impacting the P_m values of Al catalysts with $A_3B_nC_p$ ligands. Though more comprehensive model was given in Fig. 4e, the two-descriptor model featured the importance of HOMO energies of B_nC_p fragments, as highlighted in SHAP analysis in Fig. 4a.

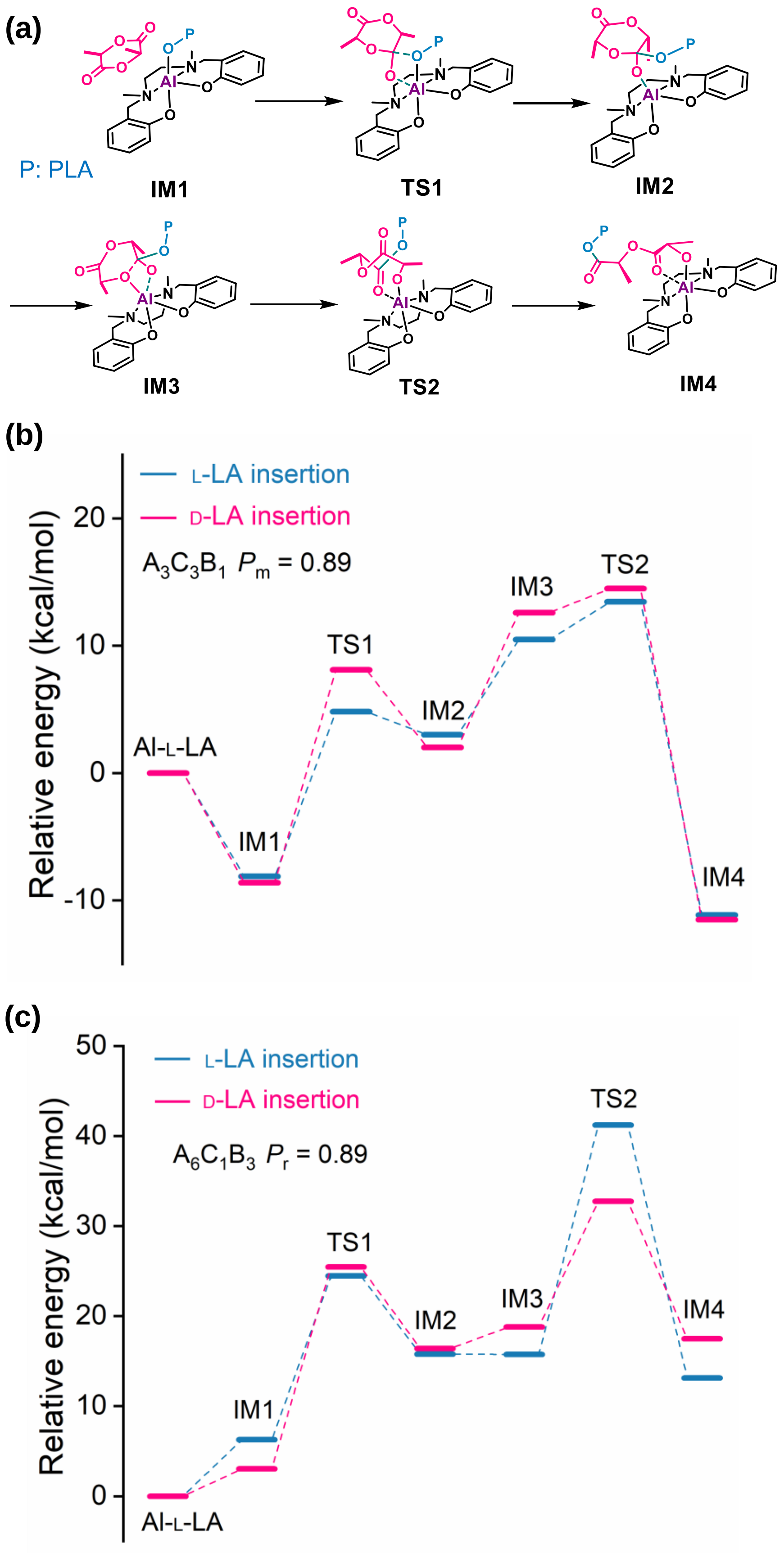

(a)
P: PLA
IM1
TS1
IM2
IM3
TS2
IM4
(b)
L-LA insertion
D-LA insertion
$A_3C_3B_1$ P_m = 0.89
Relative energy (kcal/mol)
Al-L-LA
IM1
TS1
IM2
IM3
TS2
IM4
(c)
L-LA insertion
D-LA insertion
$A_6C_1B_3$ P_r = 0.89
Relative energy (kcal/mol)
Al-L-LA
IM1
TS1
IM2
IM3
TS2
IM4

Supplementary Figure 15. (a) The reaction pathways of coordination-insertion ring-opening of LA by Al-alkoxide.[59] (b) DFT-calculated ring-opening reaction pathways of LA isomers by isoselective ($A_3C_3B_1$)Al-(L-LA)$_2$. (c) DFT-calculated ring-opening reaction pathways of LA isomers by heteroselective ($A_6C_1B_3$)Al-(L-LA)$_2$. Note that based on the energy diagrams and literature studies,[59] **TS1** may be critical for isoselective ROP whereas **TS2** important for heteroselective ROP (also see Supplementary Table 11).

Discussion:

Recently, Tolman, Kol and coworkers discussed the possible rapid exchange of lactidyl units between the Al catalysts with different enantiomers in the ROP of *rac*-LA.[46] In our DFT computation, such stereoisomer interconversion was not considered, as well as possible dimeric-monomeric Al complex equilibrium.

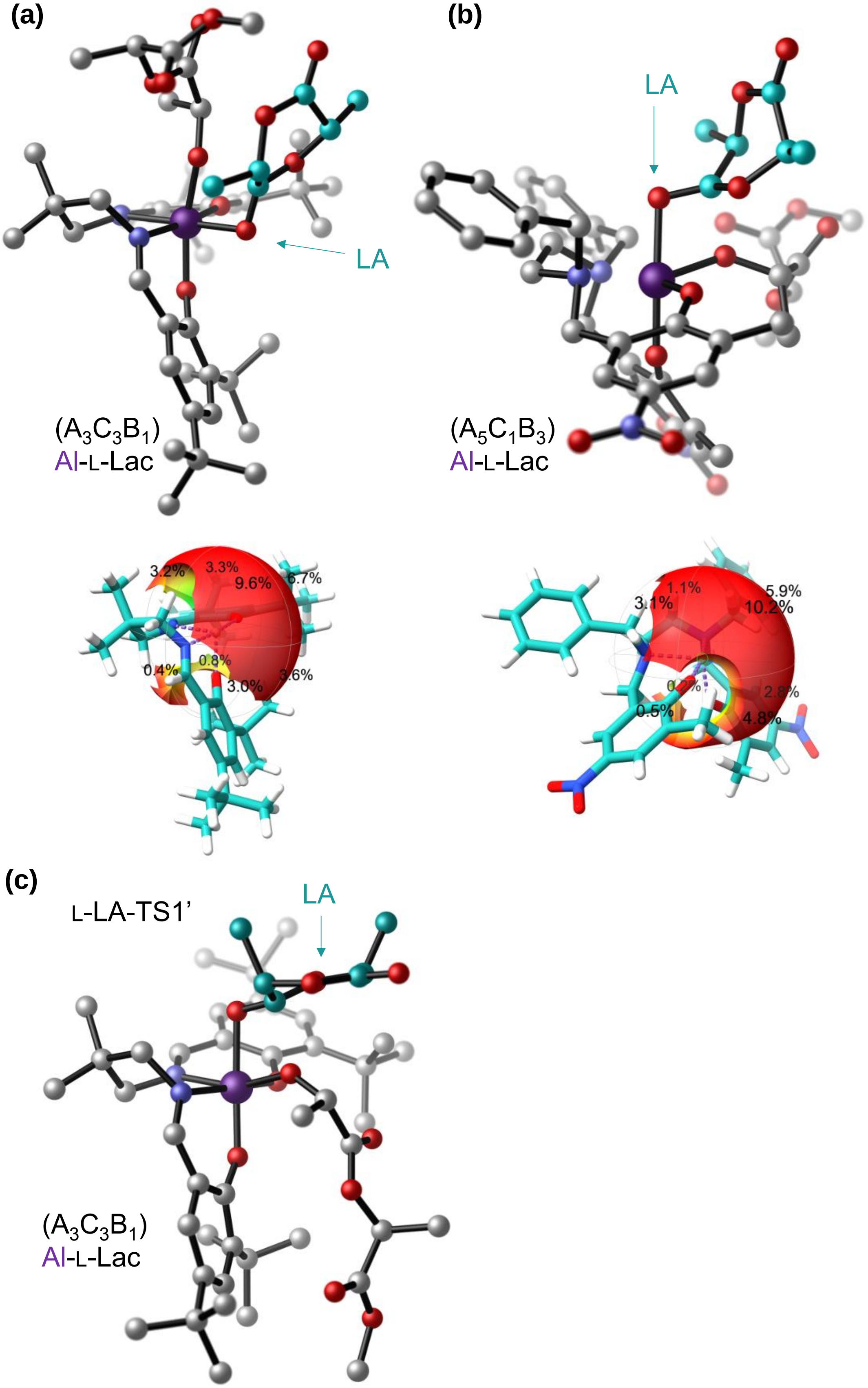
(a)
LA
(A3C3B1)
Al-L-Lac
(b)
LA
(A5C1B3)
Al-L-Lac
(c)
L-LA-TS1’
LA
(A3C3B1)
Al-L-Lac

Supplementary Figure 16. The V_{bur} of Al complex might impact the monomer docking position in the **TS1** state in the ring-opening reaction. The N-N-O-Al atoms (counter closkwise) of the ligand were positioned in the *xy* plane. (a) Isoselective ($A_3C_3B_1$)Al complex reacting with LA, whose carbonyl group approach Al along *xy* plane. (b) Heteroselective ($A_5C_1B_3$)Al complex reacting with LA, whose carbonyl group approaching Al from the top *z* axis. It is likely that the top hemisphere of Al complex in ($A_5C_1B_3$)Al has less free volume compared with that of ($A_3C_3B_1$)Al, which could not allow for the alkoxide polymer docking on the top of the catalyst. (c) DFT computation on possible **TS1** docking structures of LA from the top *z* axis to ($A_3C_3B_1$)Al.

Discussion:

We note that we also performed DFT computation on possible **TS1** docking structures of LA from the top *z* axis to ($A_3C_3B_1$)Al, as shown in (c). Such conformers exhibited 2.29 kcal/mol higher energy than the current **TS1** that had LA approaching from the xy plane to the Al center.

For the buried volume calculation of the ligand, we followed the literature method to calculate the whole catalyst including metal. The Al complex was DFT computed first to obtain the optimized geometry without imaginary frequencies (the lowest energy). As shown in Gibson's paper (Figure 1 in reference 45) and confirmed by our computation studies, there is no isomer found for such achiral salan based Al complexes. Thus, in our case there is only one stable conformation of our Al complex, and buried volume calculation is not affected by the nitrogen atom chirality.

S7. NMR Spectra of Ligands

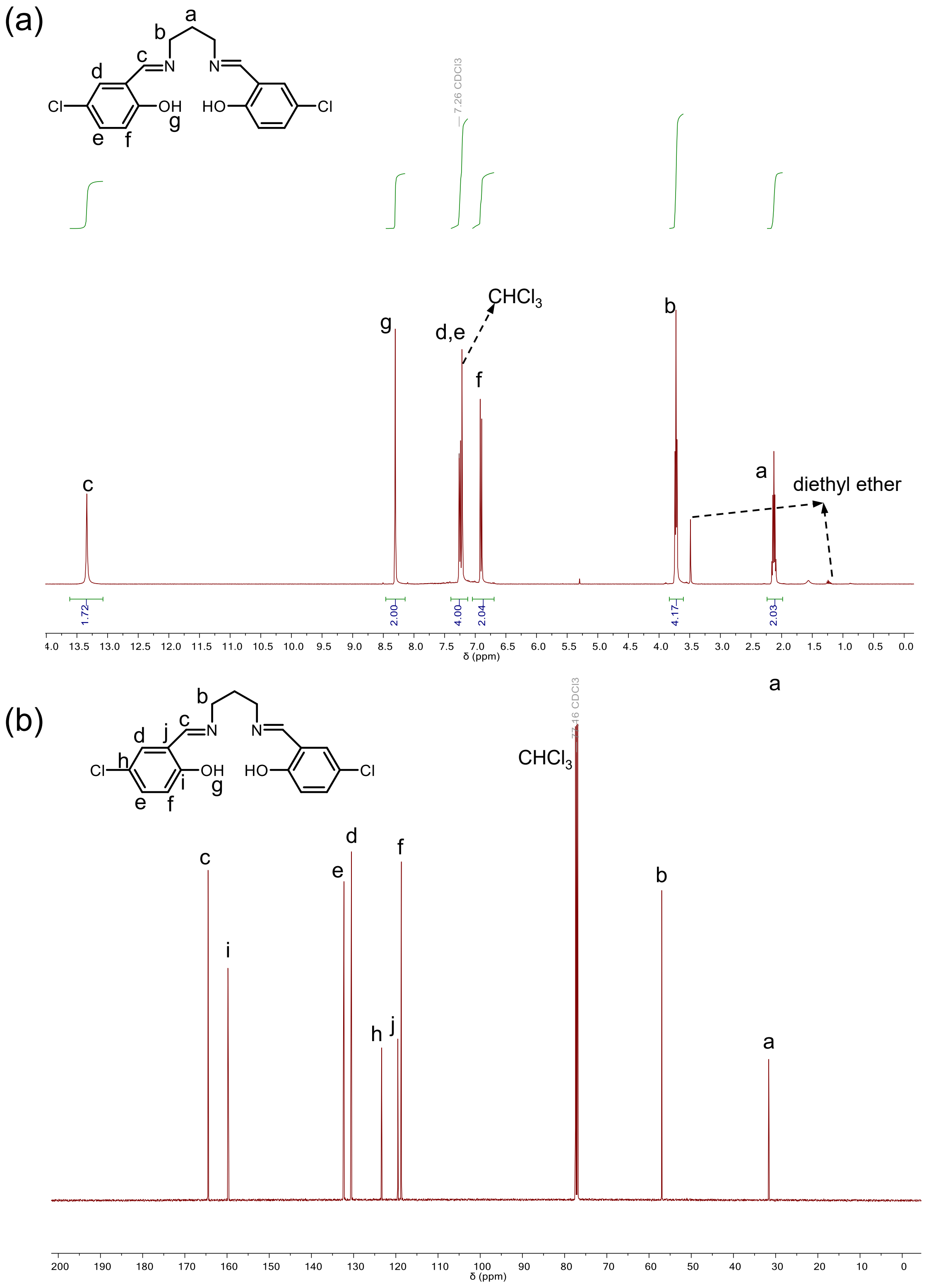

Supplementary Figure 17. (a) ^{1}H NMR (400 MHz, $CDCl_3$) and (b) ^{13}C NMR (125 MHz, $CDCl_3$) spectra of $A_7C_2B_1$.

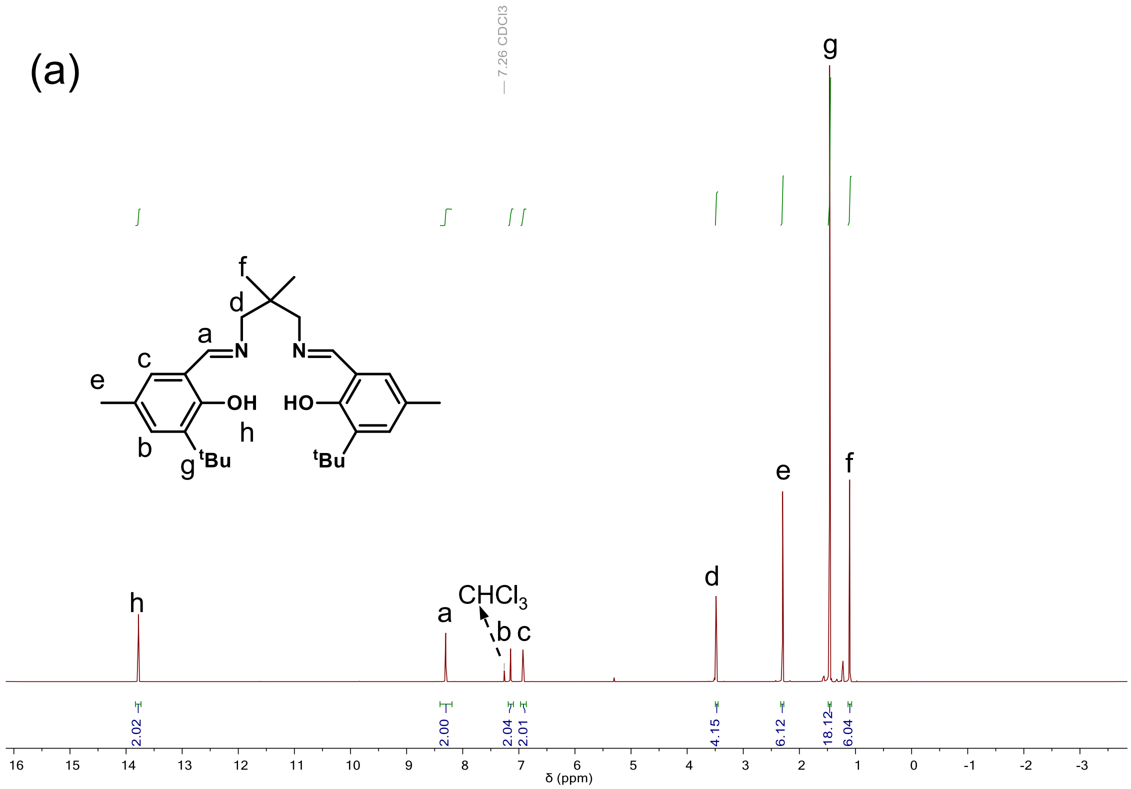

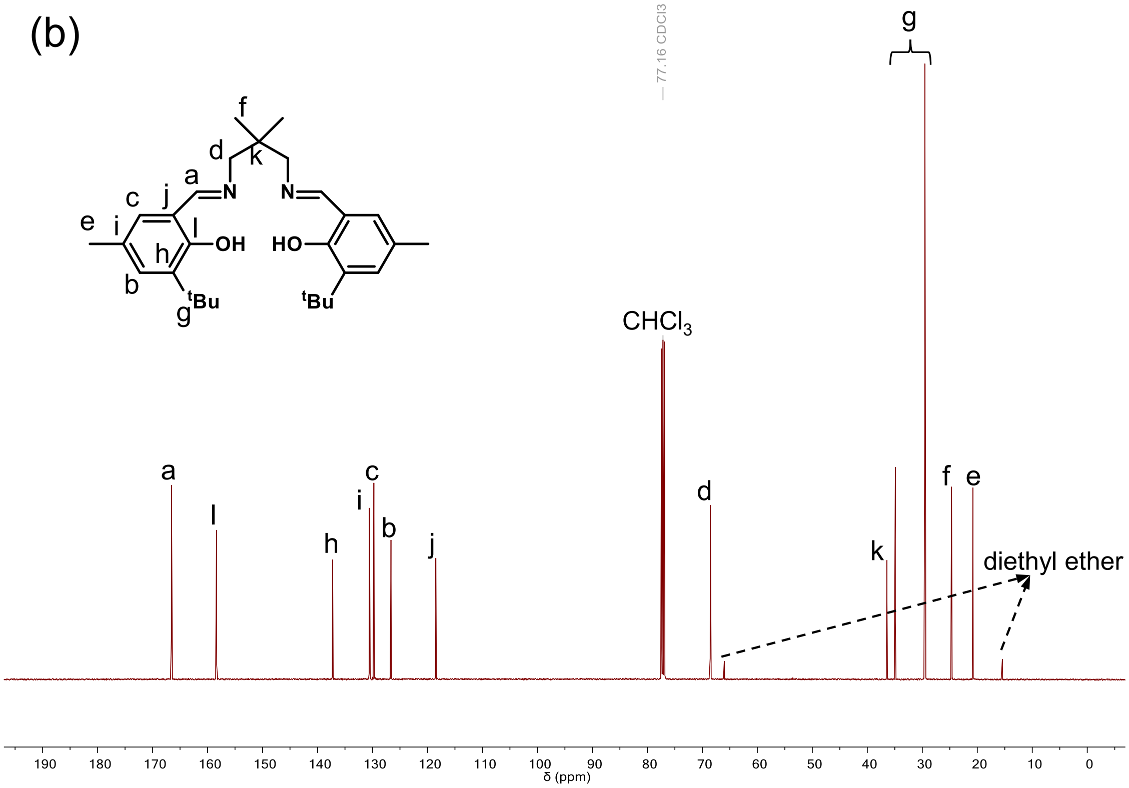

Supplementary Figure 18. (a) ^{1}H NMR (400 MHz, $CDCl_3$) and (b) ^{13}C NMR (125 MHz, $CDCl_3$) spectra of $A_{11}C_3B_1$.

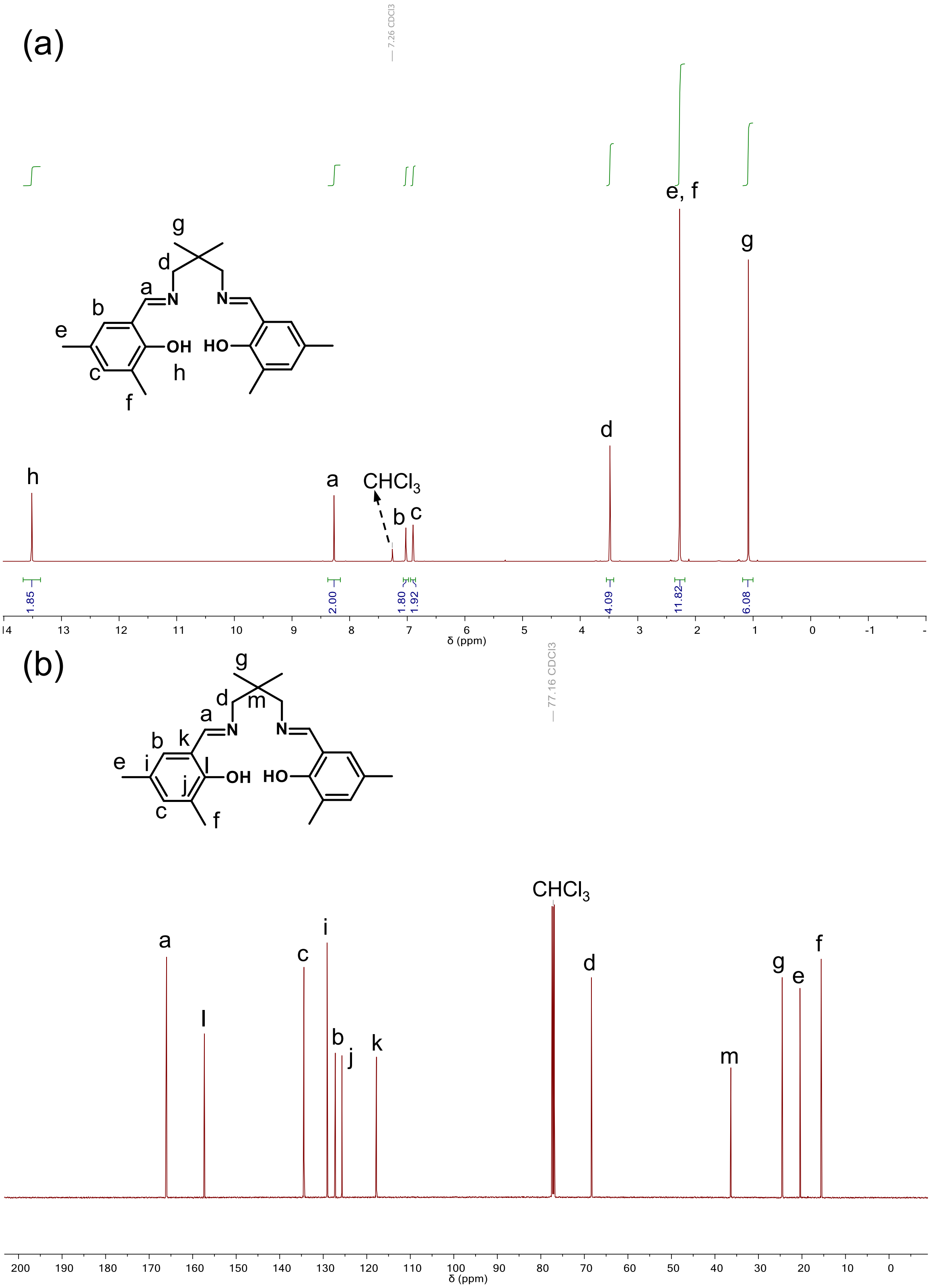

Supplementary Figure 19. (a) ^{1}H NMR (400 MHz, $CDCl_3$) and (b) ^{13}C NMR (125 MHz, $CDCl_3$) spectra of $A_{14}C_3B_1$.

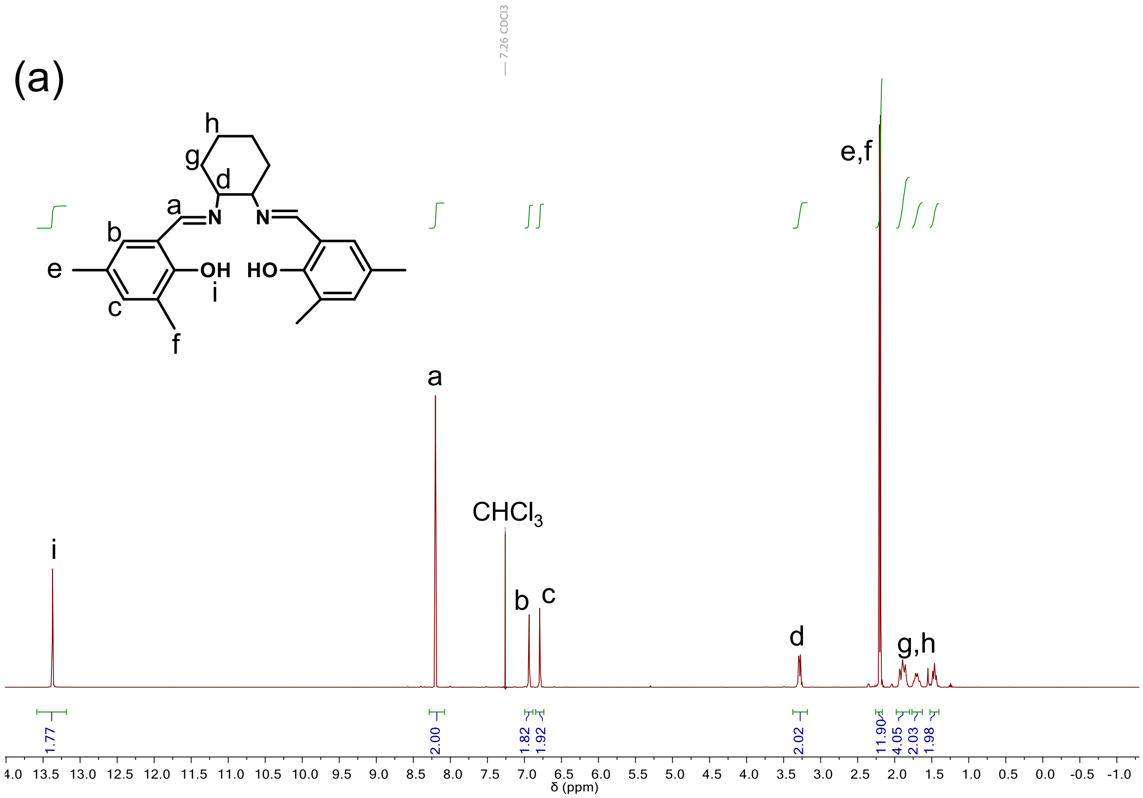

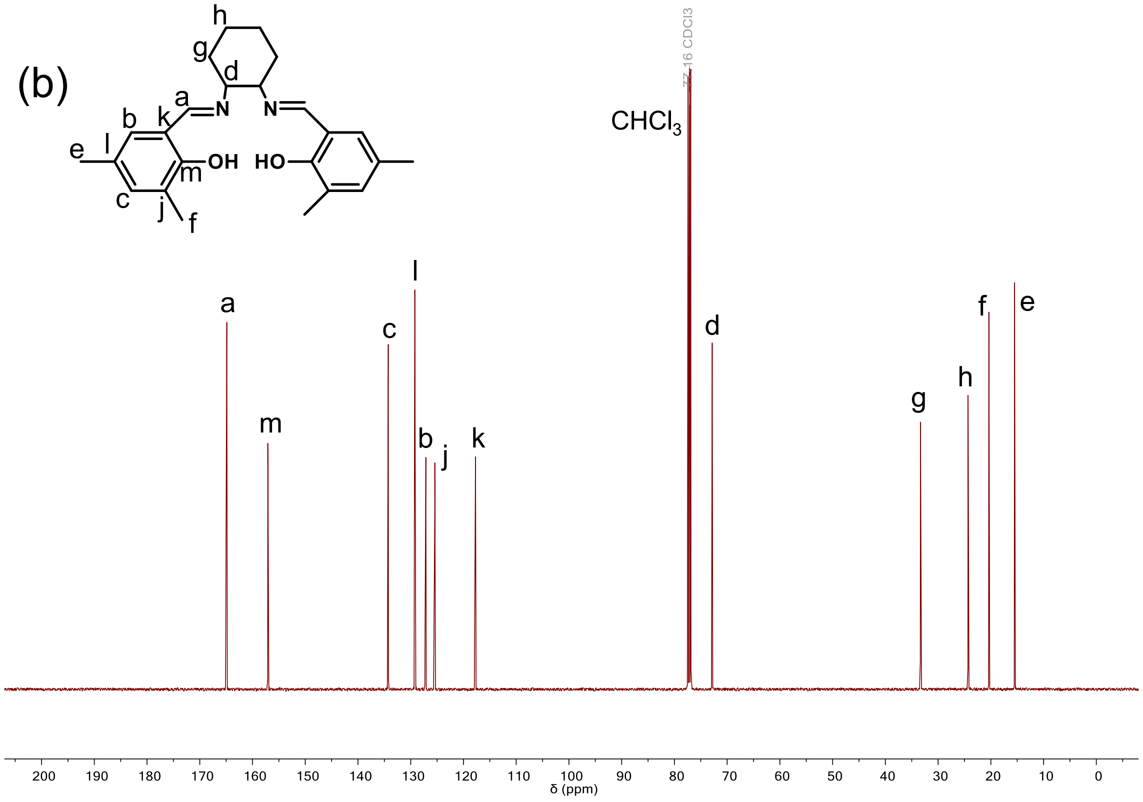

Supplementary Figure 20. (a) ^{1}H NMR (400 MHz, $CDCl_3$) and (b) ^{13}C NMR (125 MHz, $CDCl_3$) spectra of $A_{14}C_5B_1$.

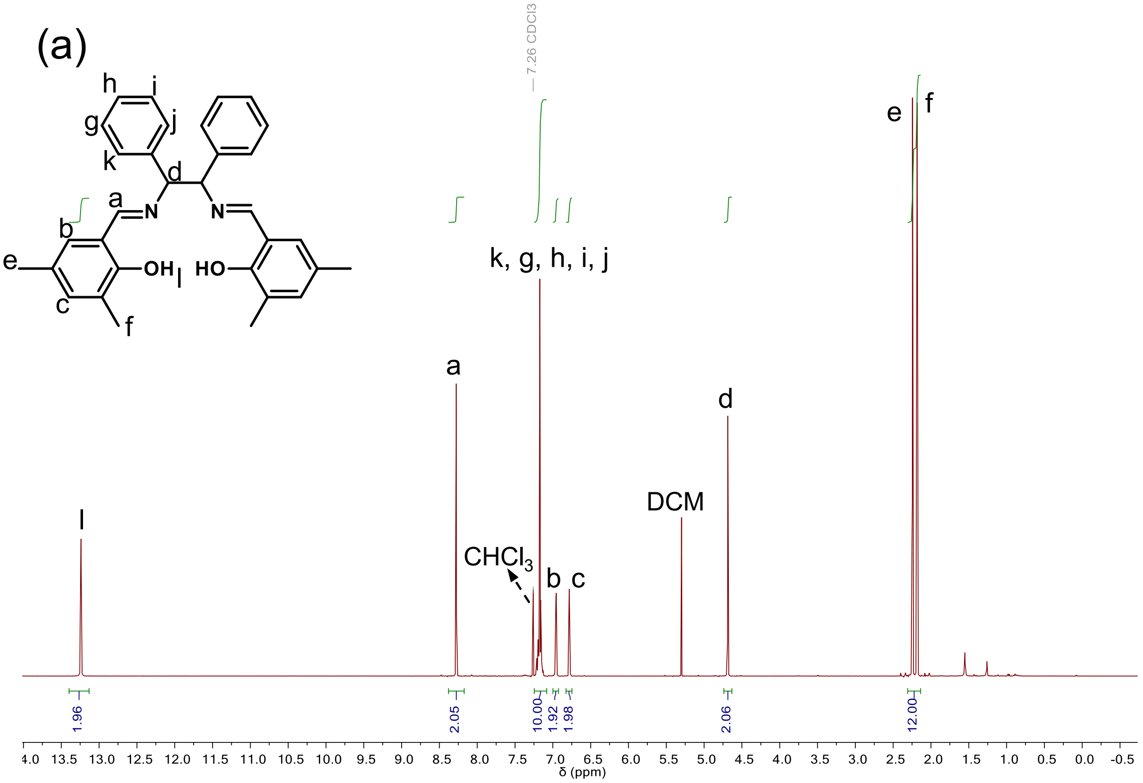

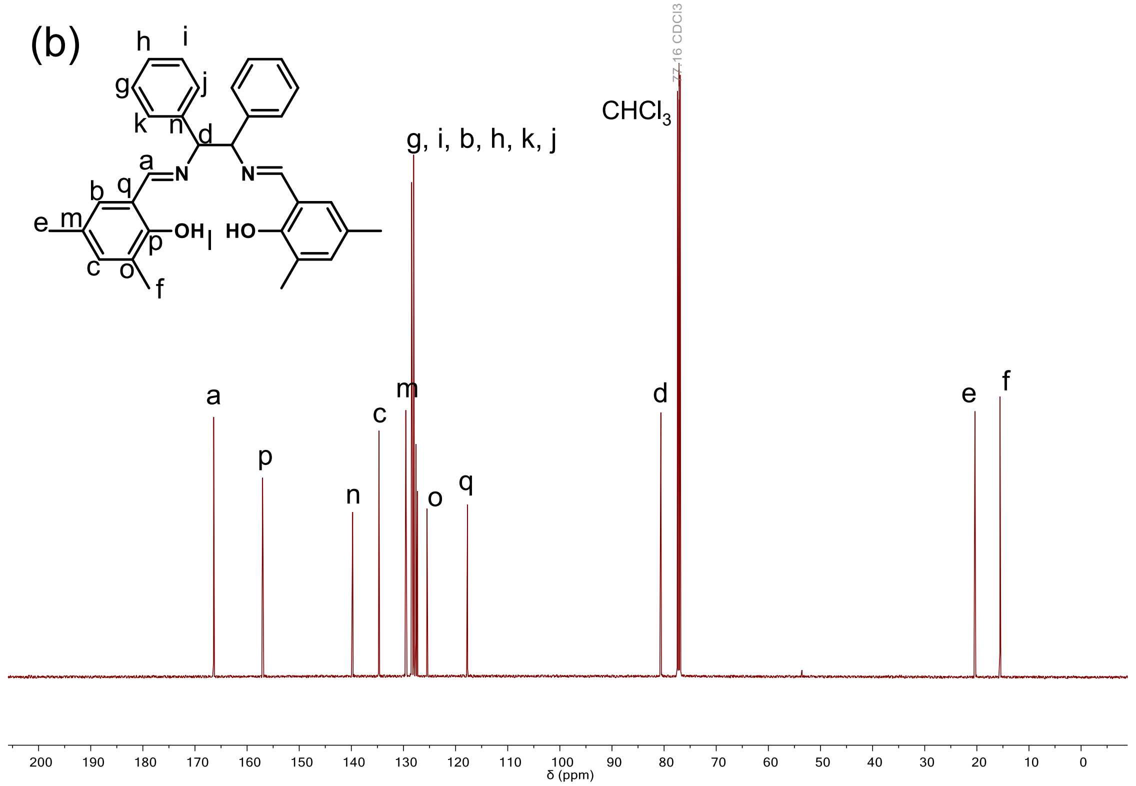

Supplementary Figure 21. (a) ^{1}H NMR (400 MHz, $CDCl_3$) and (b) ^{13}C NMR (125 MHz, $CDCl_3$) spectra of $A_{14}C_{11}B_1$.

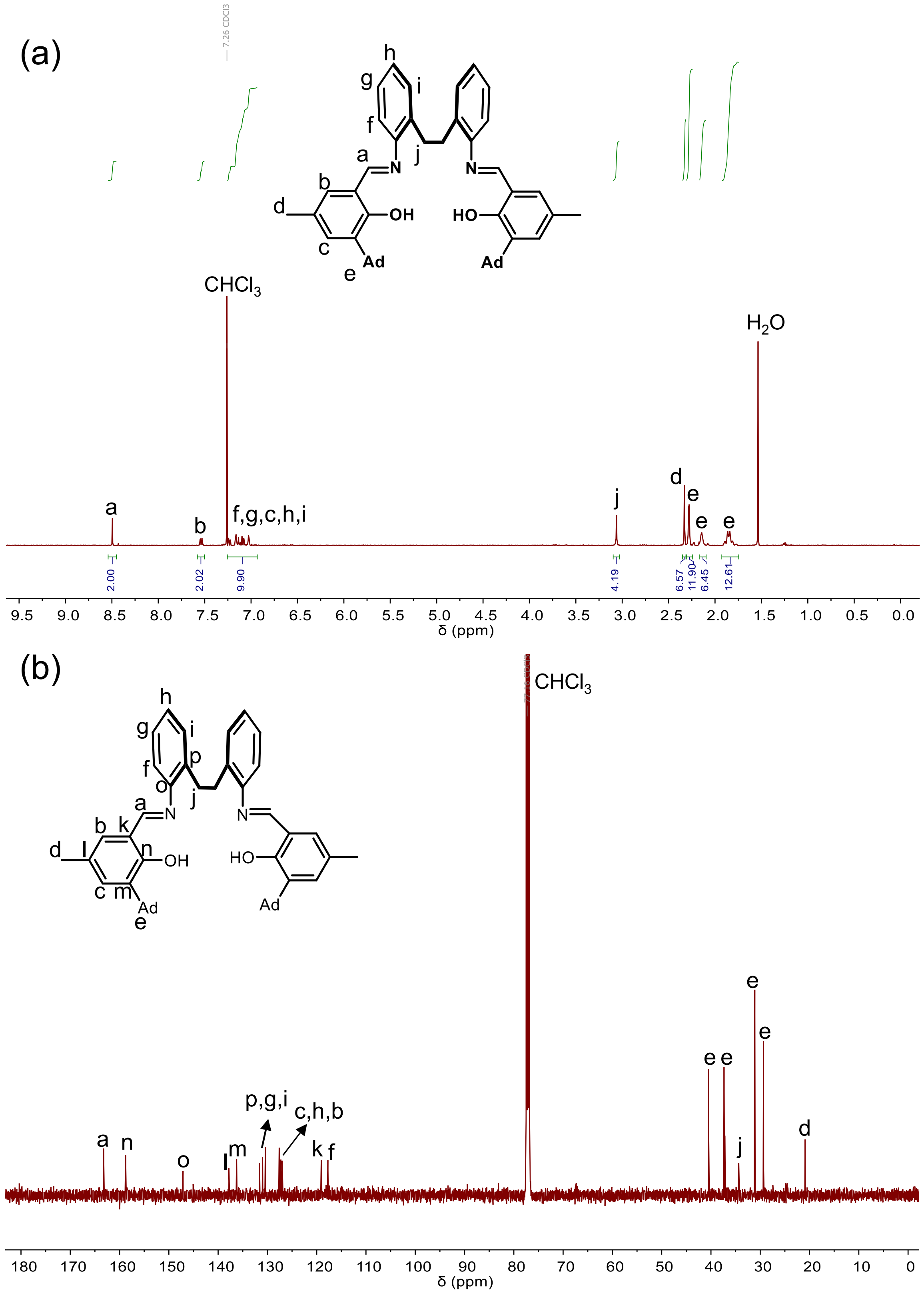

Supplementary Figure 22. (a) ^{1}H NMR (400 MHz, $CDCl_3$) and (b) ^{13}C NMR (125 MHz, $CDCl_3$) spectra of $A_8C_9B_1$.

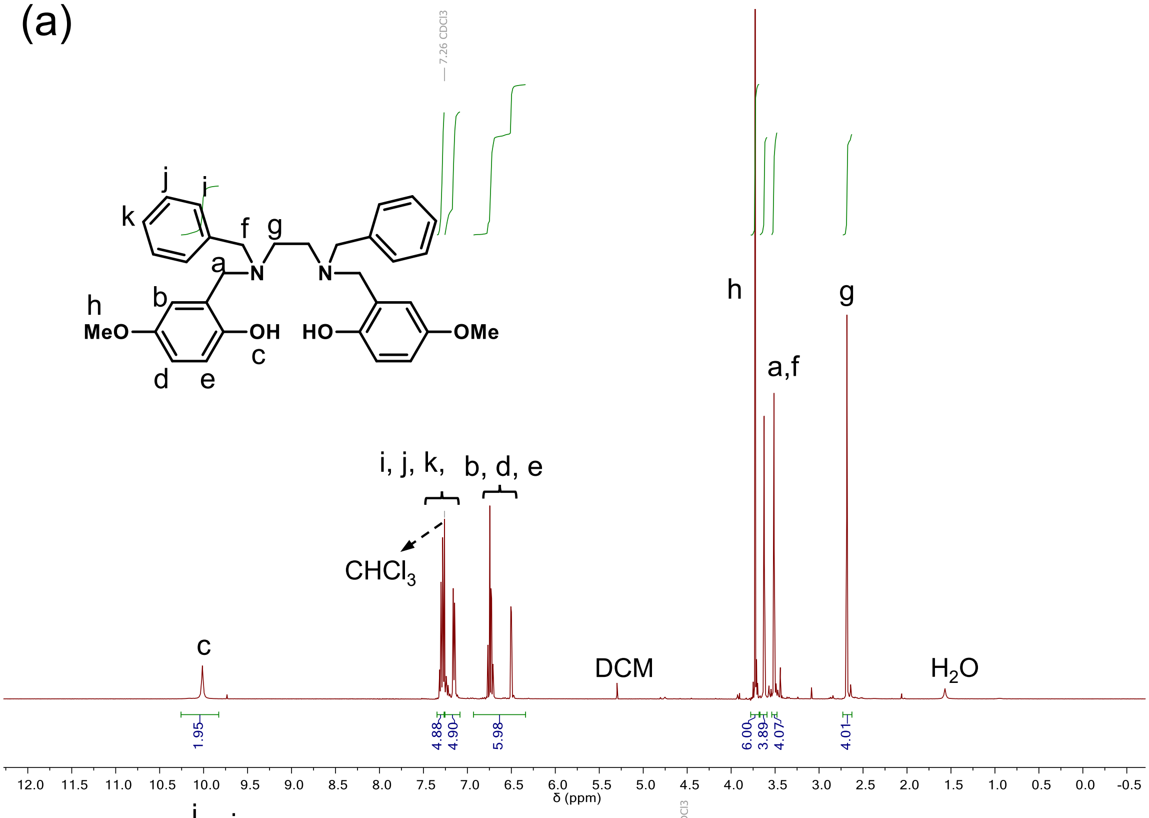

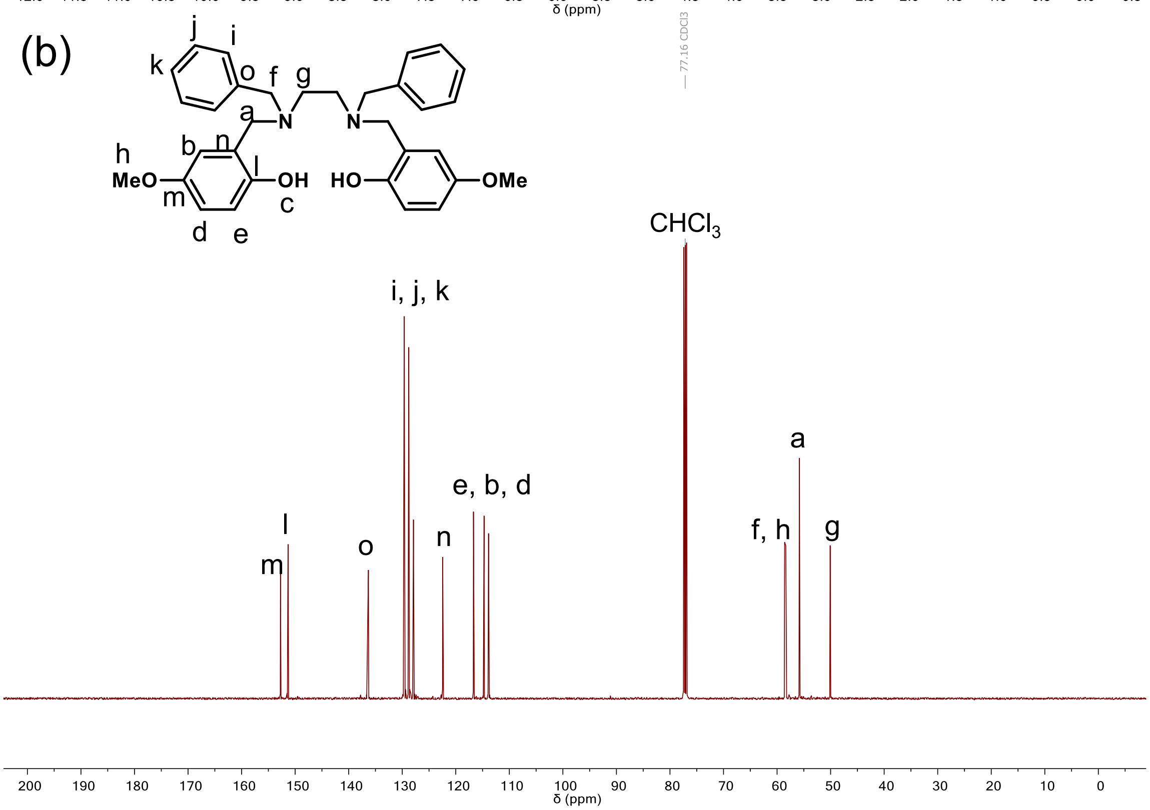

Supplementary Figure 23. (a) ^{1}H NMR (400 MHz, $CDCl_3$) and (b) ^{13}C NMR (125 MHz, $CDCl_3$) spectra of $A_4C_1B_3$.

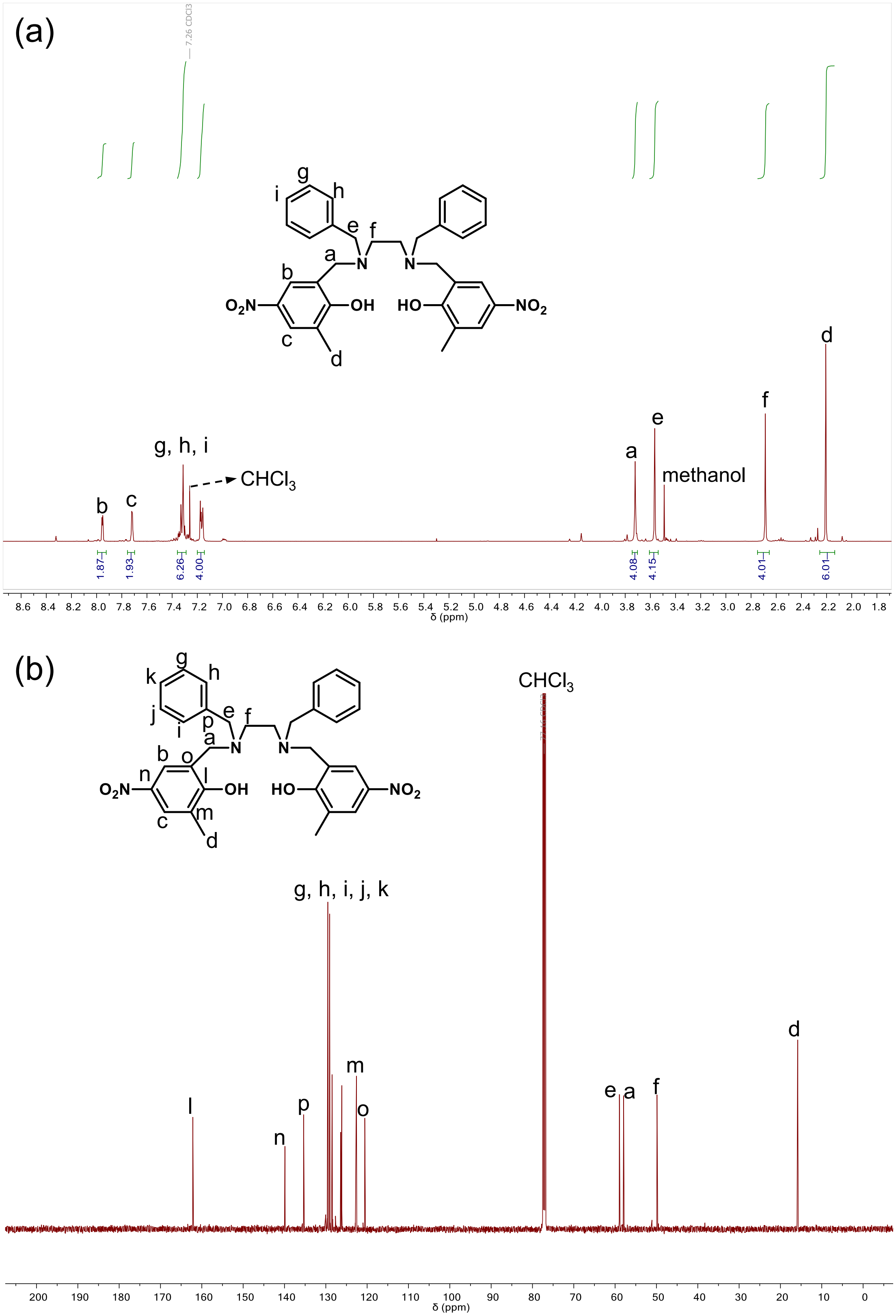

Supplementary Figure 24. (a) ^{1}H NMR (400 MHz, $CDCl_3$) and (b) ^{13}C NMR (125 MHz, $CDCl_3$) spectra of $A_5C_1B_3$.

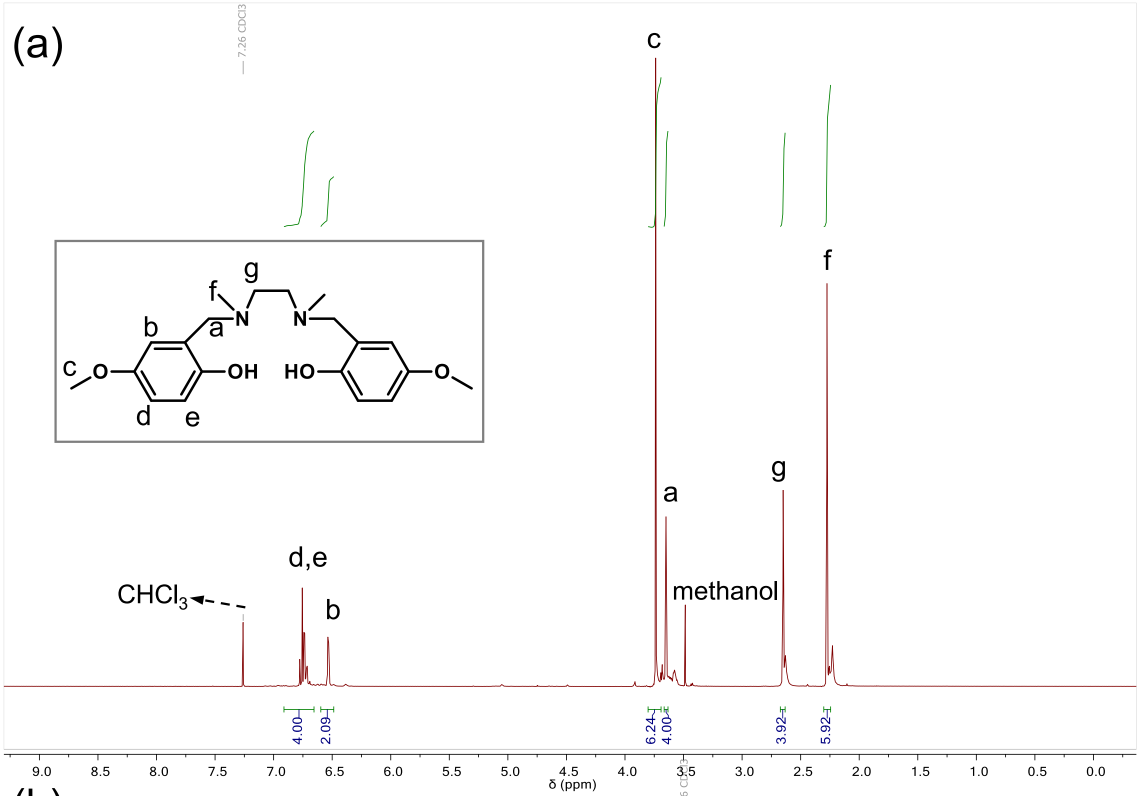

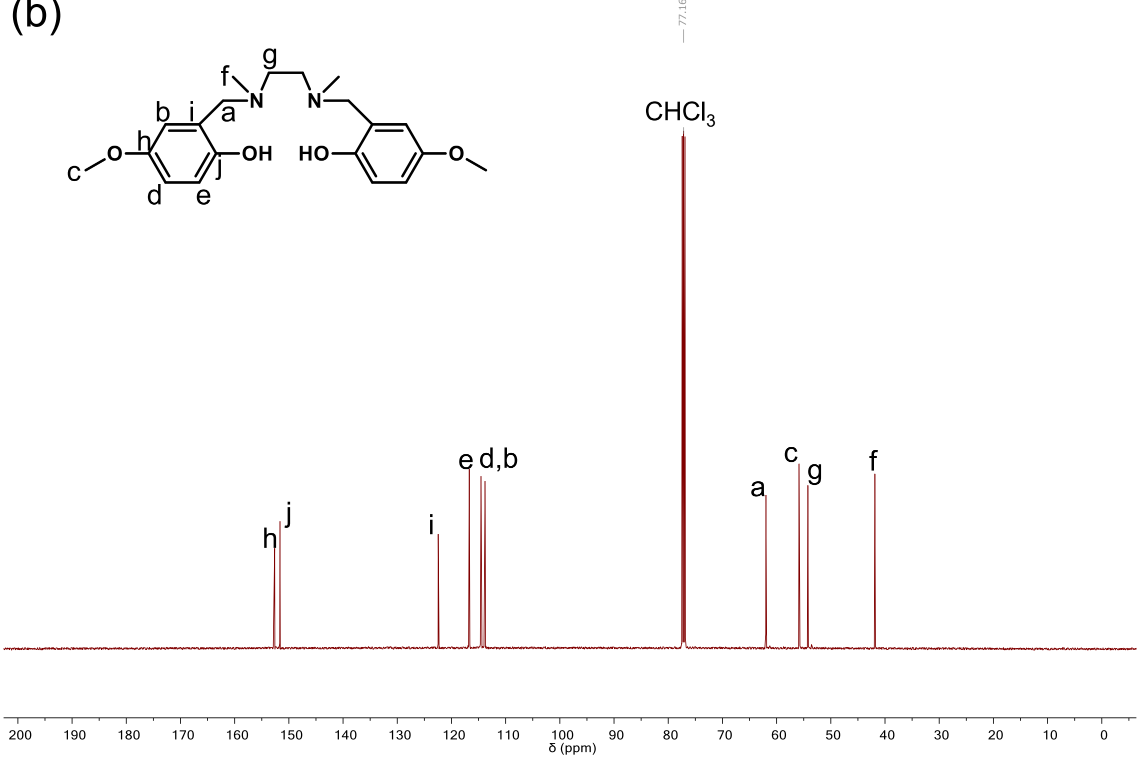

Supplementary Figure 25. (a) ^{1}H NMR (400 MHz, $CDCl_3$) and (b) ^{13}C NMR (125 MHz, $CDCl_3$) spectra of $A_4C_1B_2$.

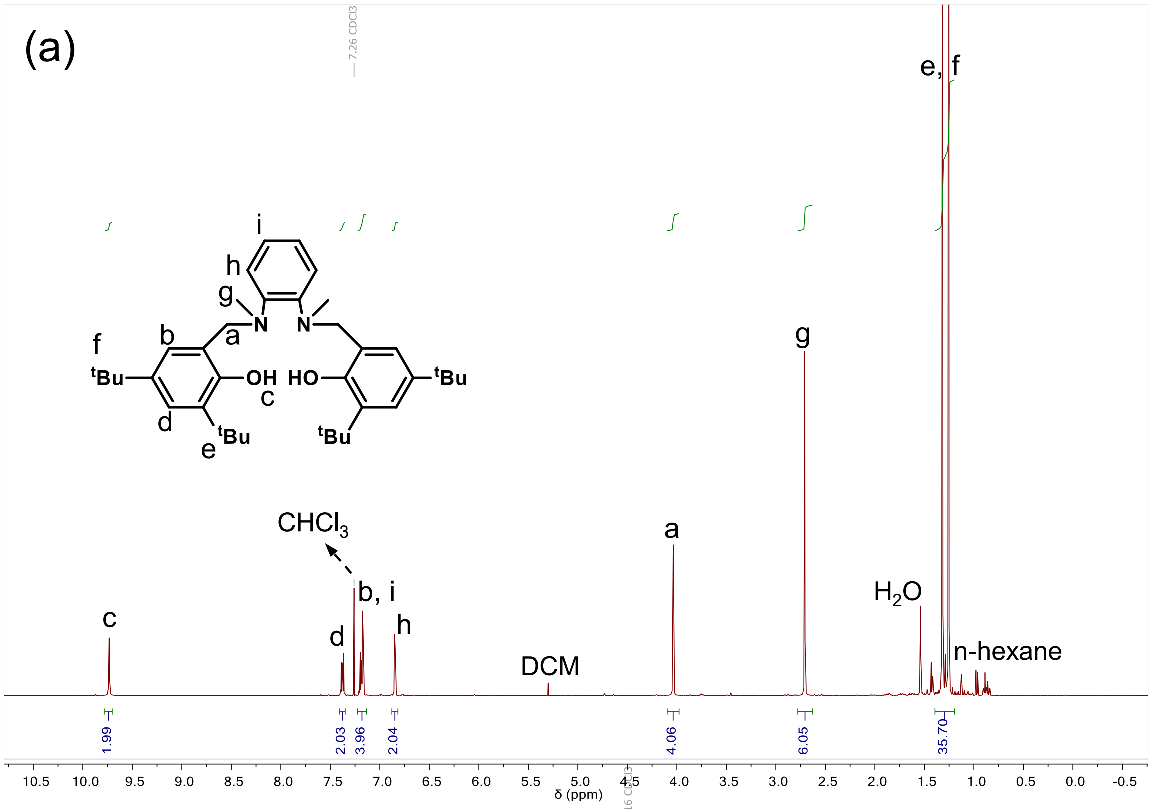

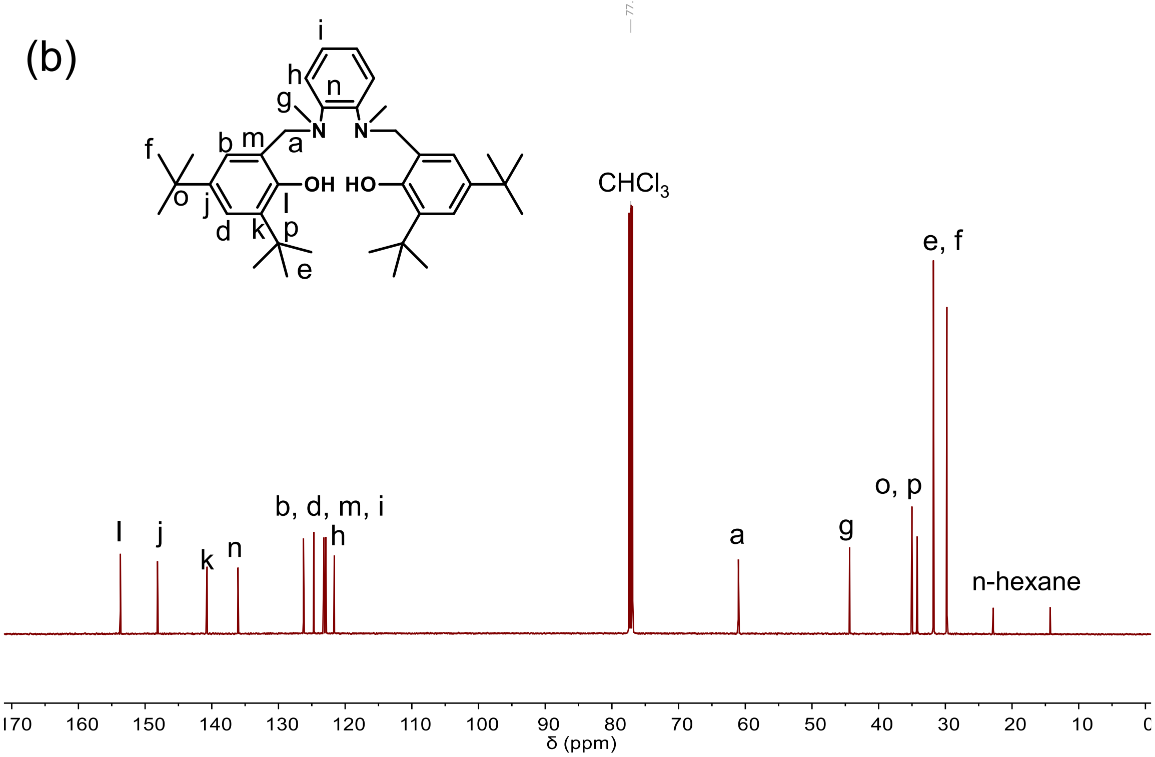

Supplementary Figure 26. (a) ^{1}H NMR (400 MHz, $CDCl_3$) and (b) ^{13}C NMR (125 MHz, $CDCl_3$) spectra of $A_3C_6B_2$.

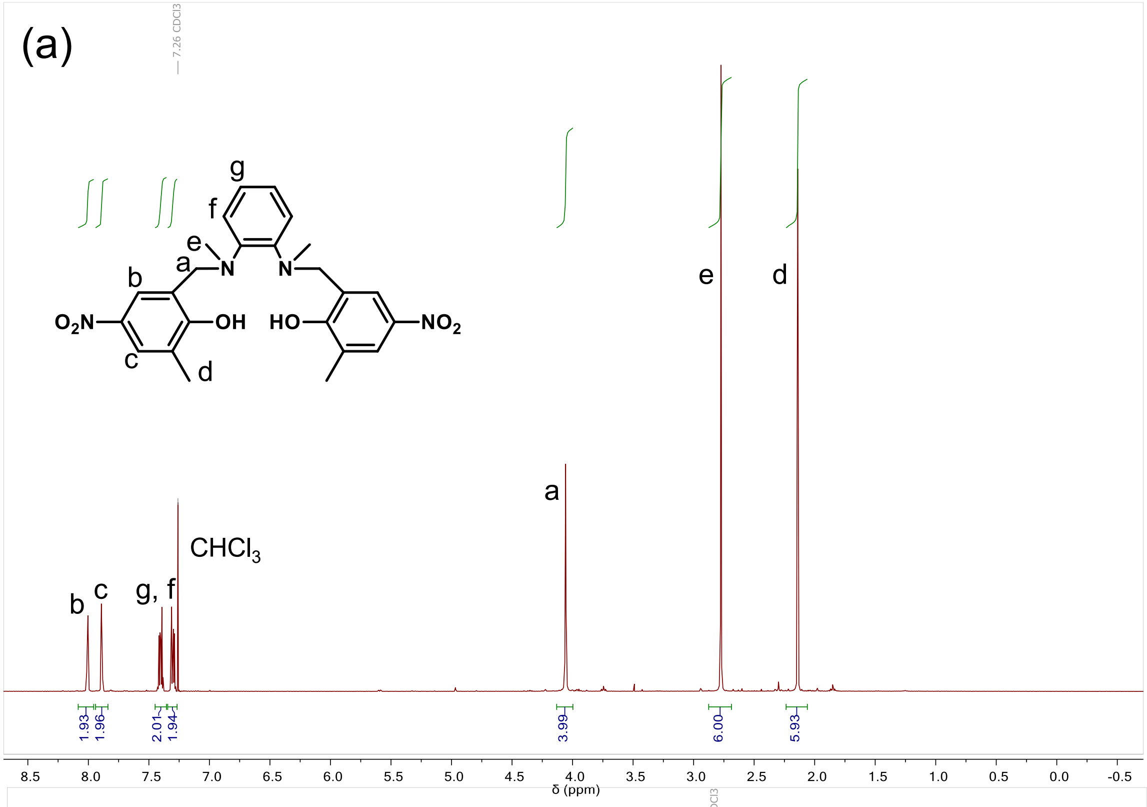

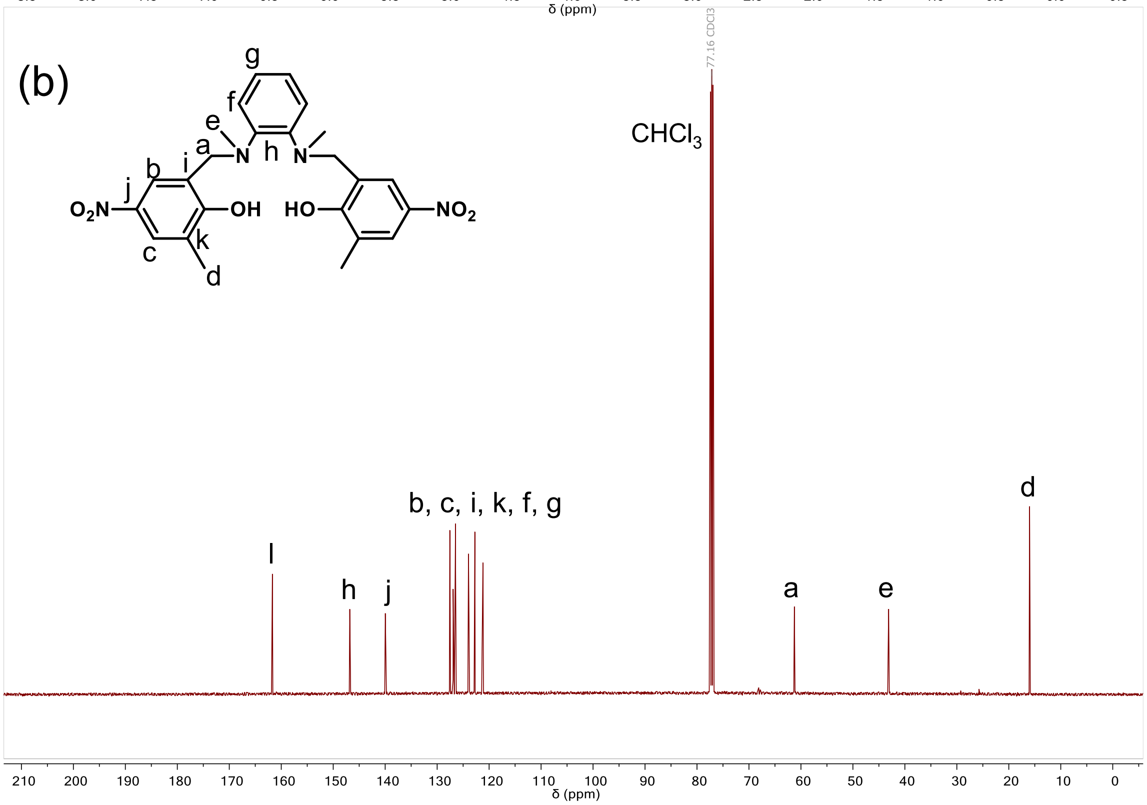

Supplementary Figure 27. (a) ^1H NMR (400 MHz, $CDCl_3$) and (b) ^{13}C NMR (125 MHz, $CDCl_3$) spectra of $A_5C_6B_2$.

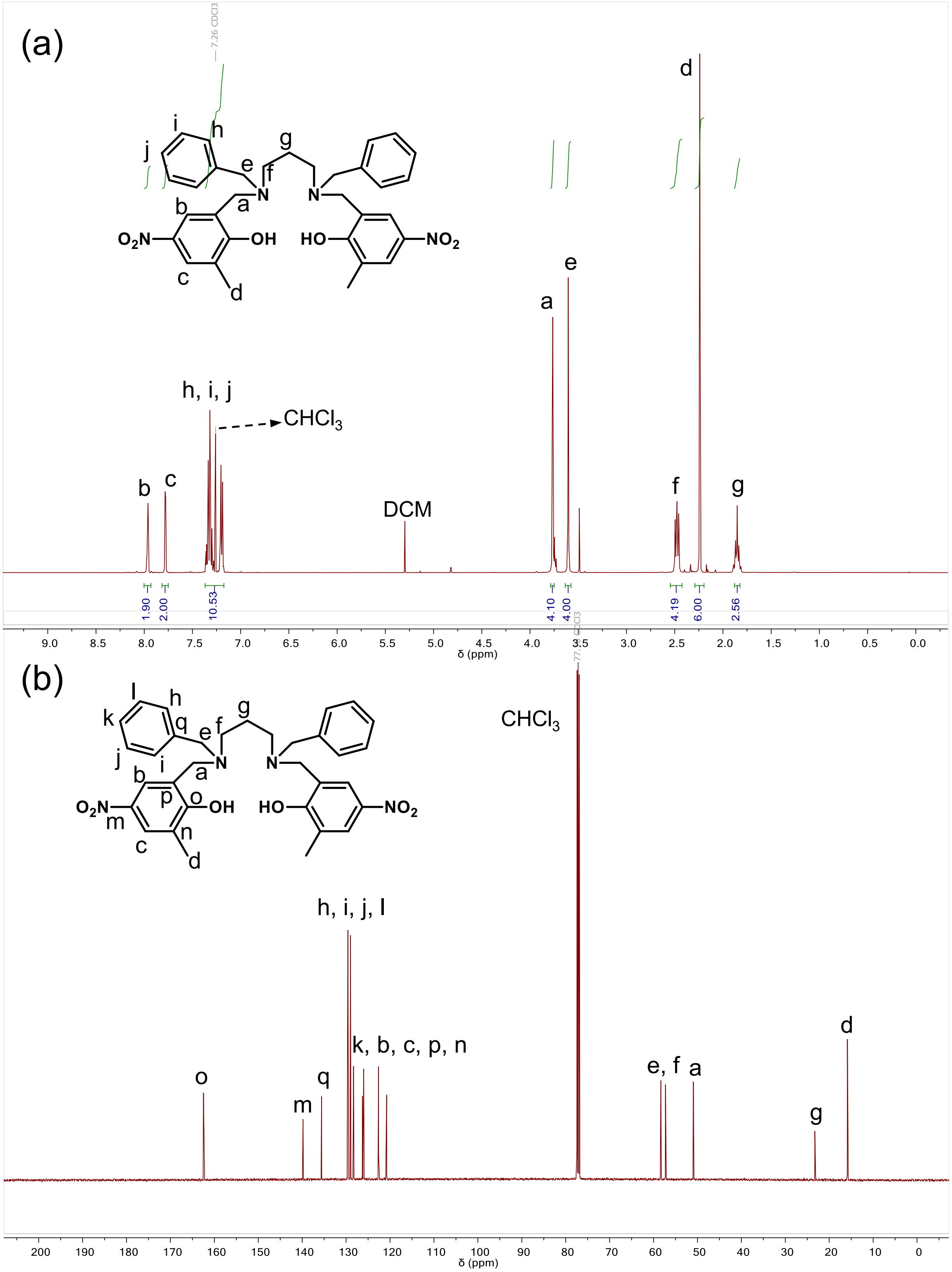

Supplementary Figure 28. (a) ^{1}H NMR (400 MHz, CDCl$_3$) and (b) ^{13}C NMR (125 MHz, CDCl$_3$) spectra of $A_5C_2B_3$.

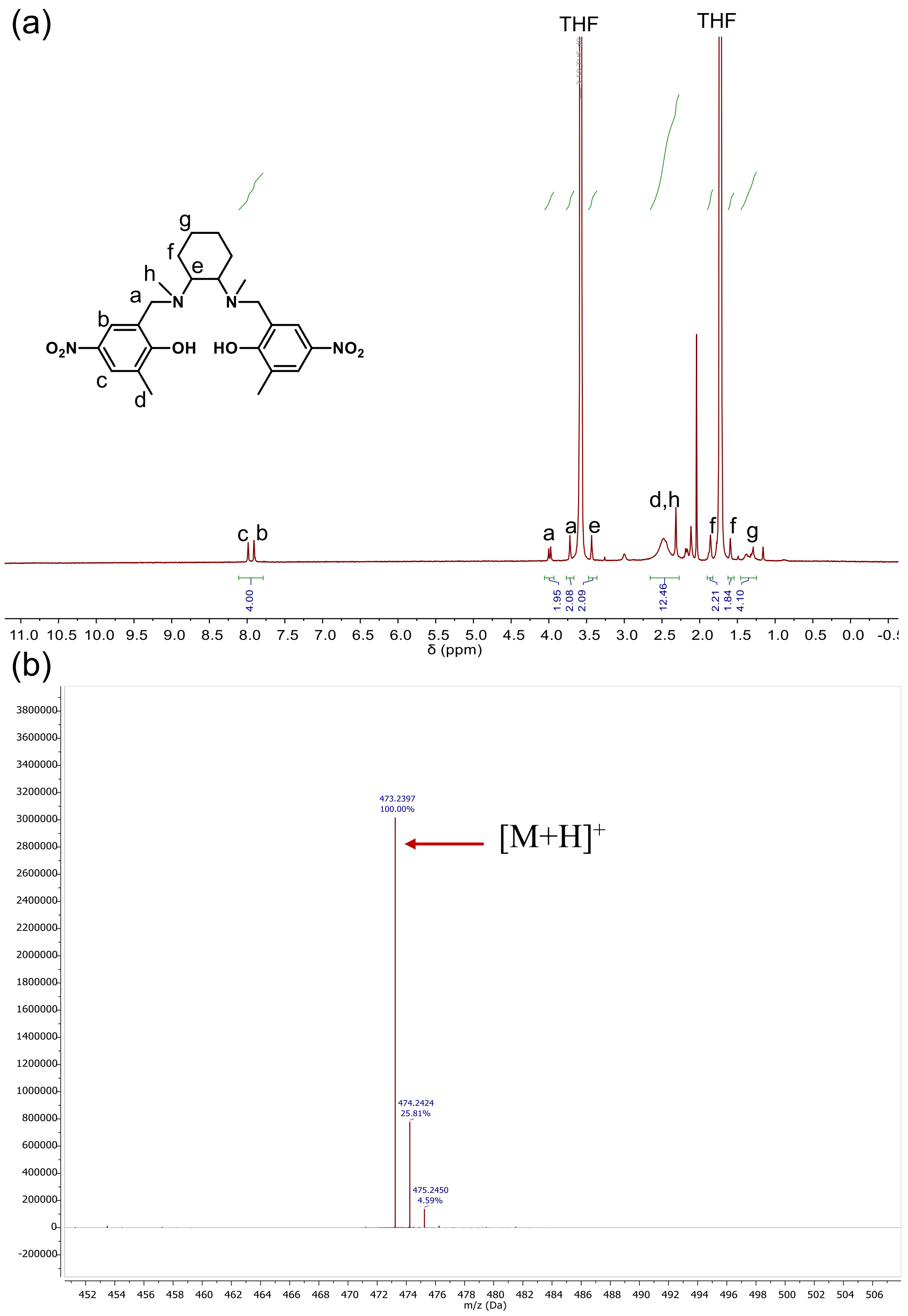

Supplementary Figure 29. (a) ^{1}H NMR (400 MHz, THF-d_8) spectra and (b) High resolution ESI-MS of $A_5C_5B_2$. We note that $A_5C_5B_2$ had low solubility in $CHCl_3$, THF and toluene. For ^{13}C NMR, the acquisition of NMR spectrum was not successful due to the precipitation over the time. Nevertheless, the purity of the product could be confirmed by ^{1}H NMR and ESI-MS.

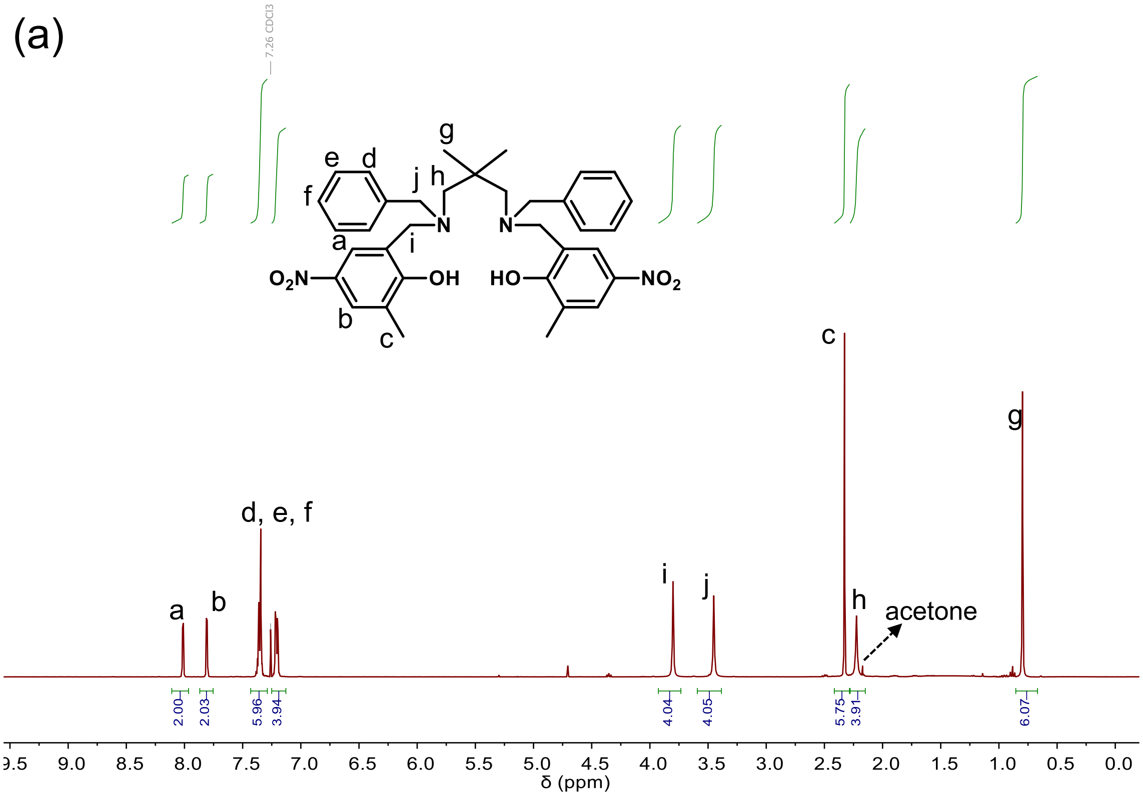

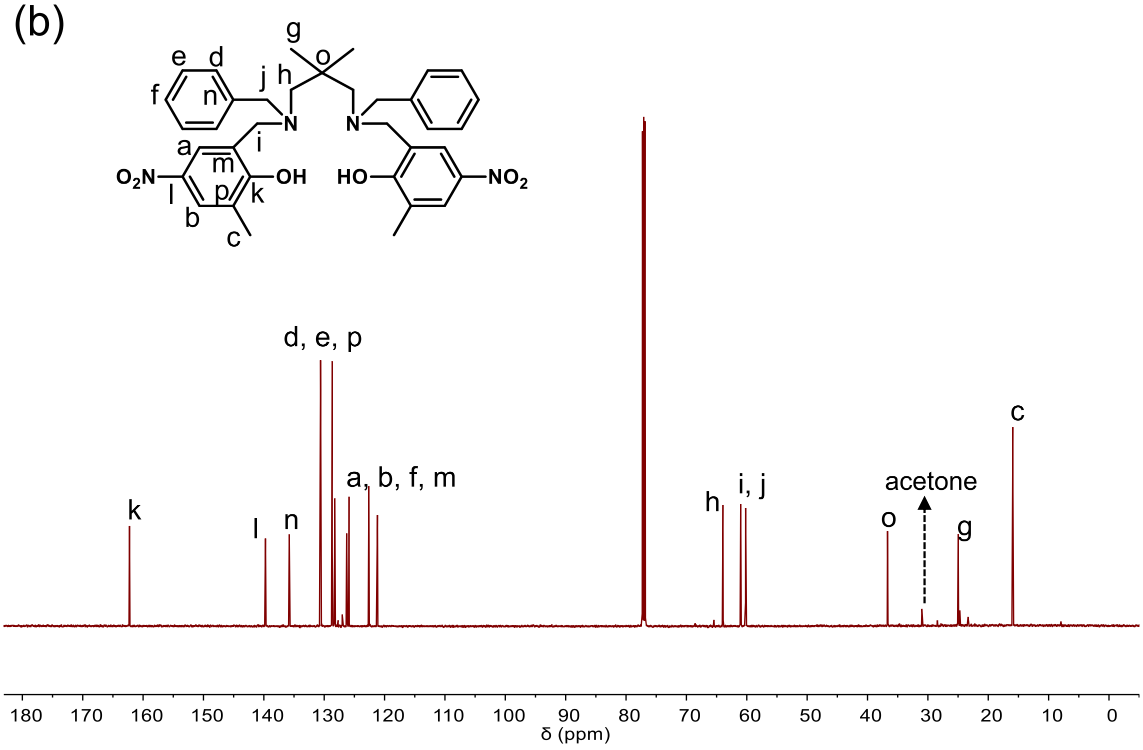

Supplementary Figure 30. (a) ^{1}H NMR (400 MHz, $CDCl_3$) and (b) ^{13}C NMR (125 MHz, $CDCl_3$) spectra of $A_5C_3B_3$.

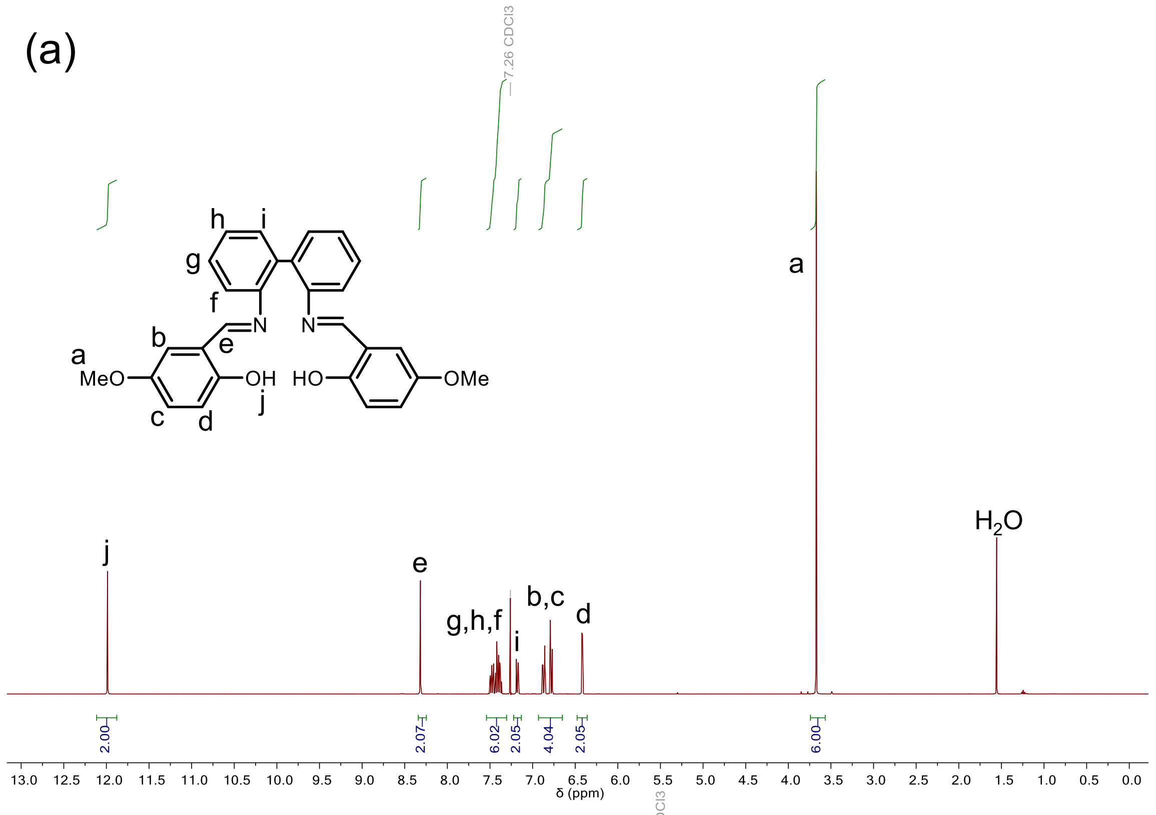

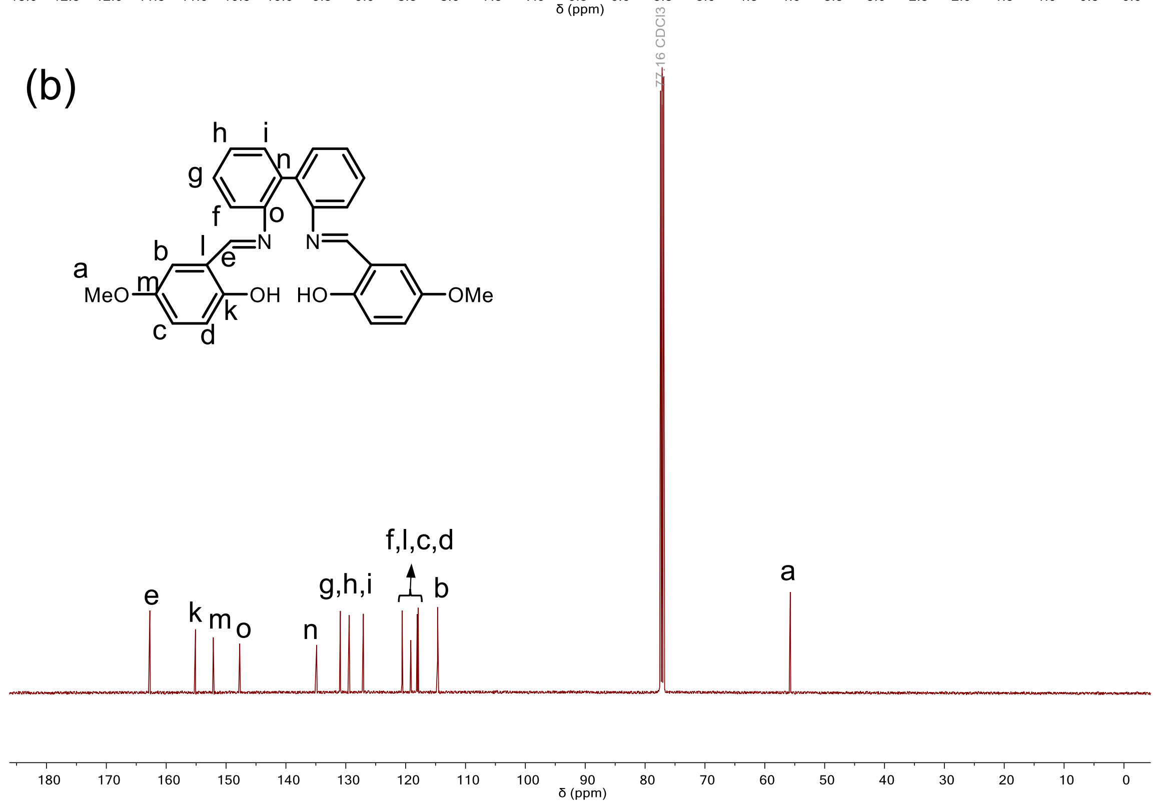

Supplementary Figure 31. (a) ^{1}H NMR (400 MHz, $CDCl_3$) and (b) ^{13}C NMR (125 MHz, $CDCl_3$) spectra of $A_4C_8B_1$.

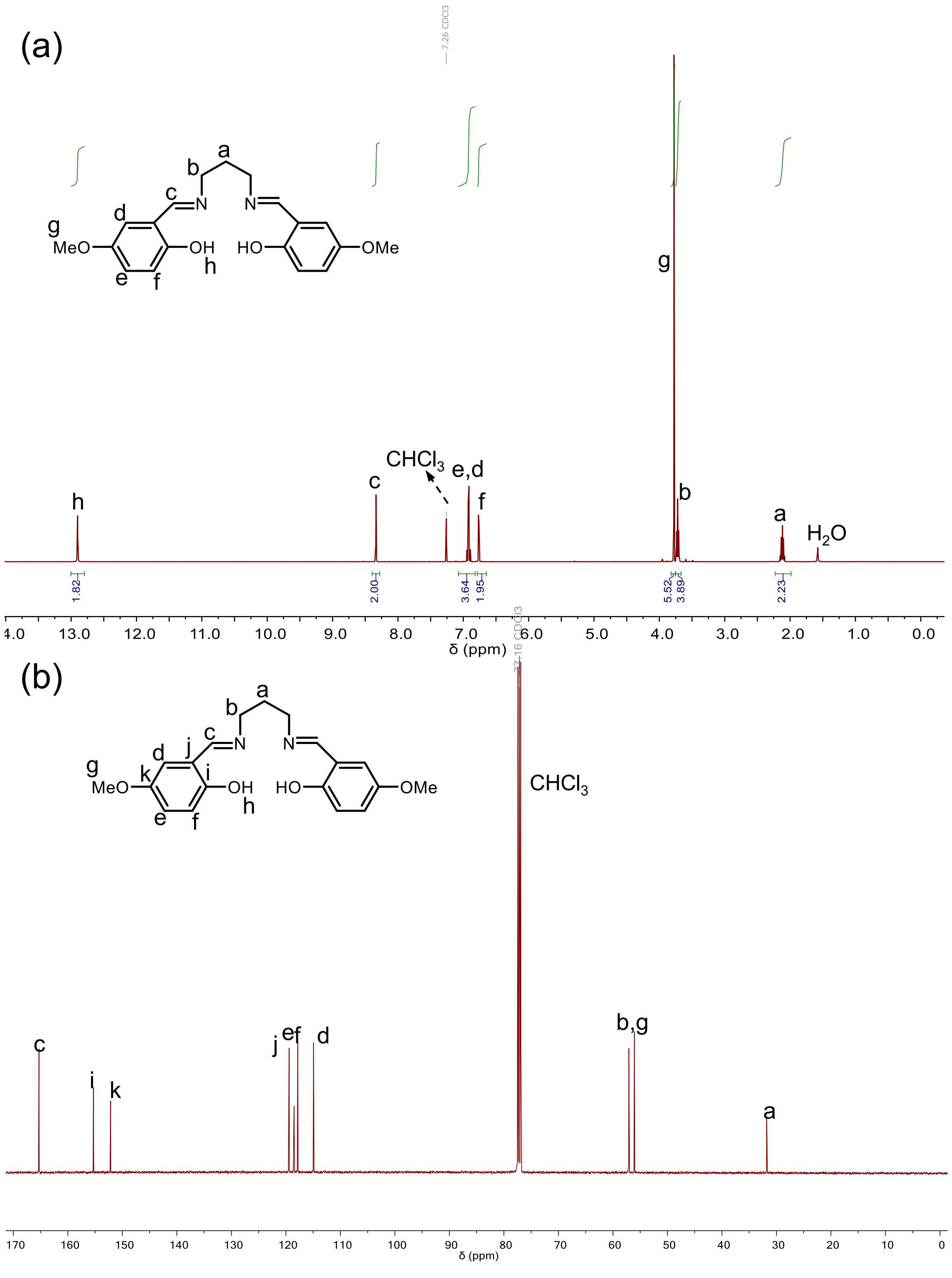

Supplementary Figure 32. (a) ^{1}H NMR (400 MHz, $CDCl_3$) and (b) ^{13}C NMR (125 MHz, $CDCl_3$) spectra of $A_4C_2B_1$.

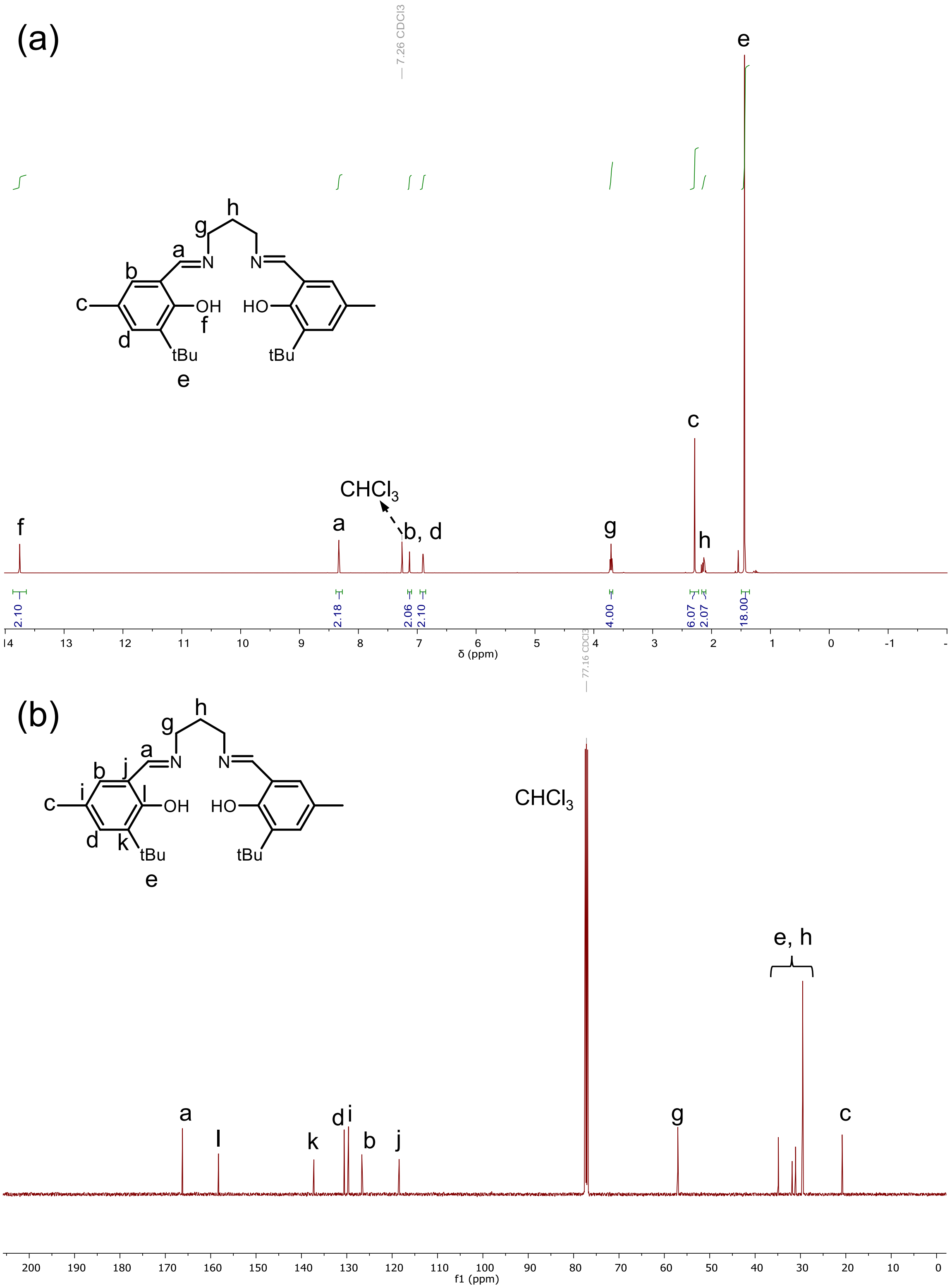

Supplementary Figure 33. (a) ^{1}H NMR (400 MHz, $CDCl_3$) and (b) ^{13}C NMR (125 MHz, $CDCl_3$) spectra of $A_{11}C_2B_1$.

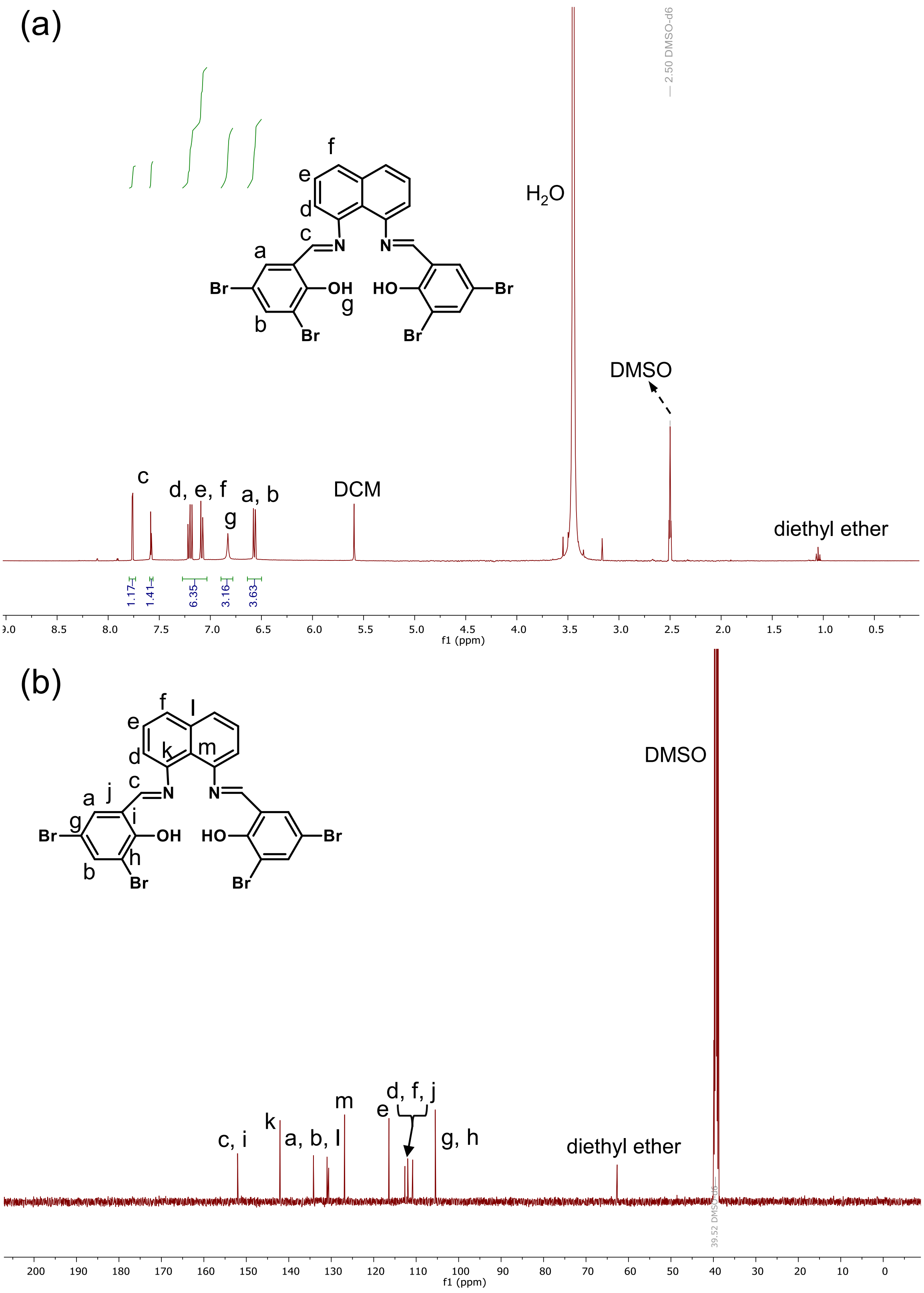

Supplementary Figure 34. (a) ^{1}H NMR (400 MHz, DMSO-d_6) and (b) ^{13}C NMR (125 MHz, DMSO-d_6) spectra of $A_{16}C_7B_1$.

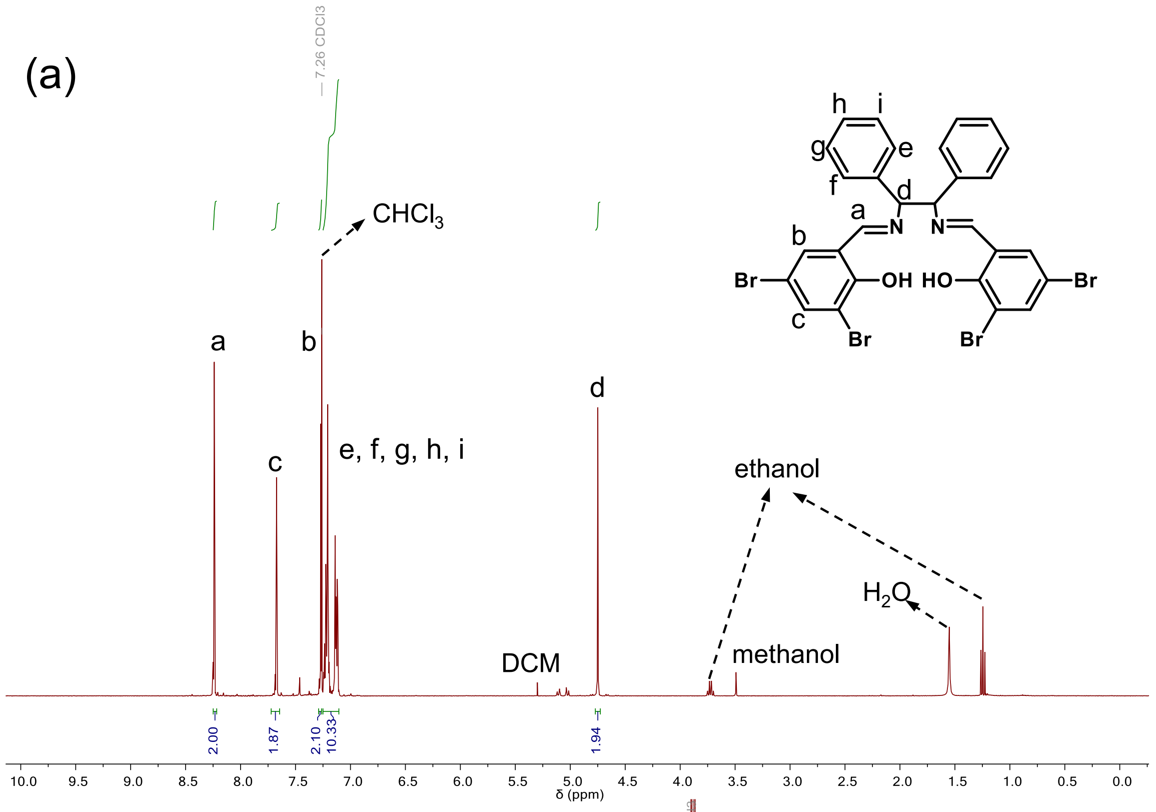

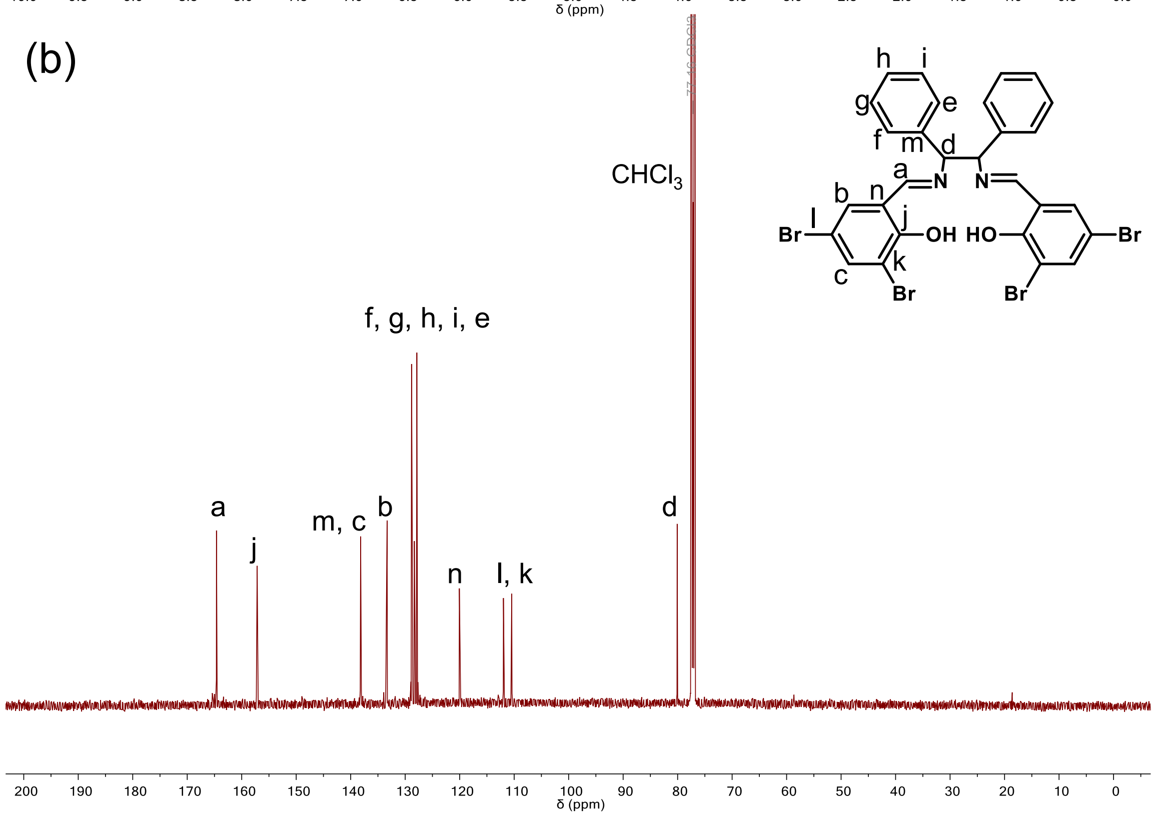

Supplementary Figure 35. (a) ^{1}H NMR (400 MHz, $CDCl_3$) and (b) ^{13}C NMR (125 MHz, $CDCl_3$) spectra of $A_{16}C_{11}B_1$.

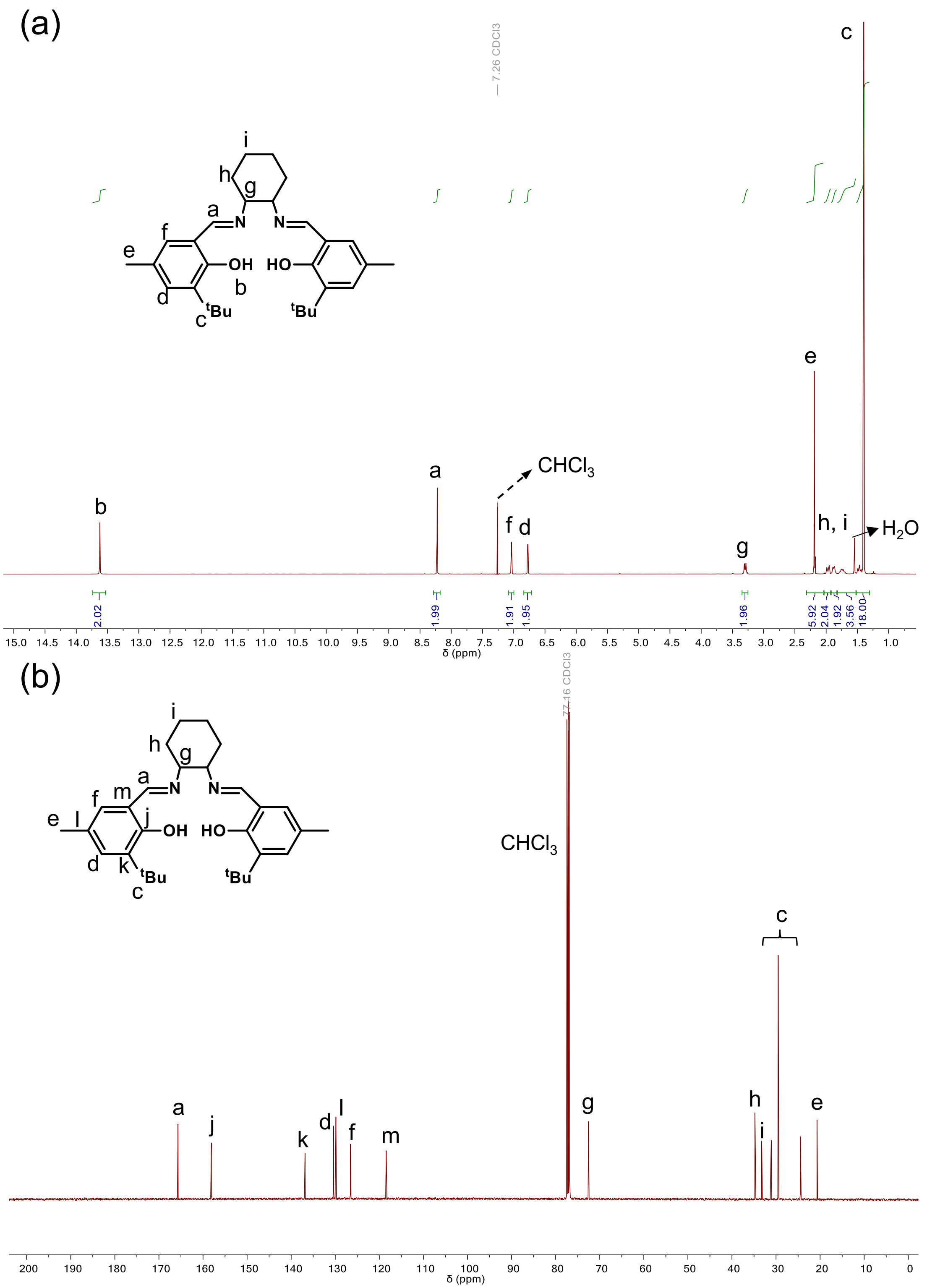

Supplementary Figure 36. (a) ^{1}H NMR (400 MHz, $CDCl_3$) and (b) ^{13}C NMR (125 MHz, $CDCl_3$) spectra of $A_{11}C_5B_1$.

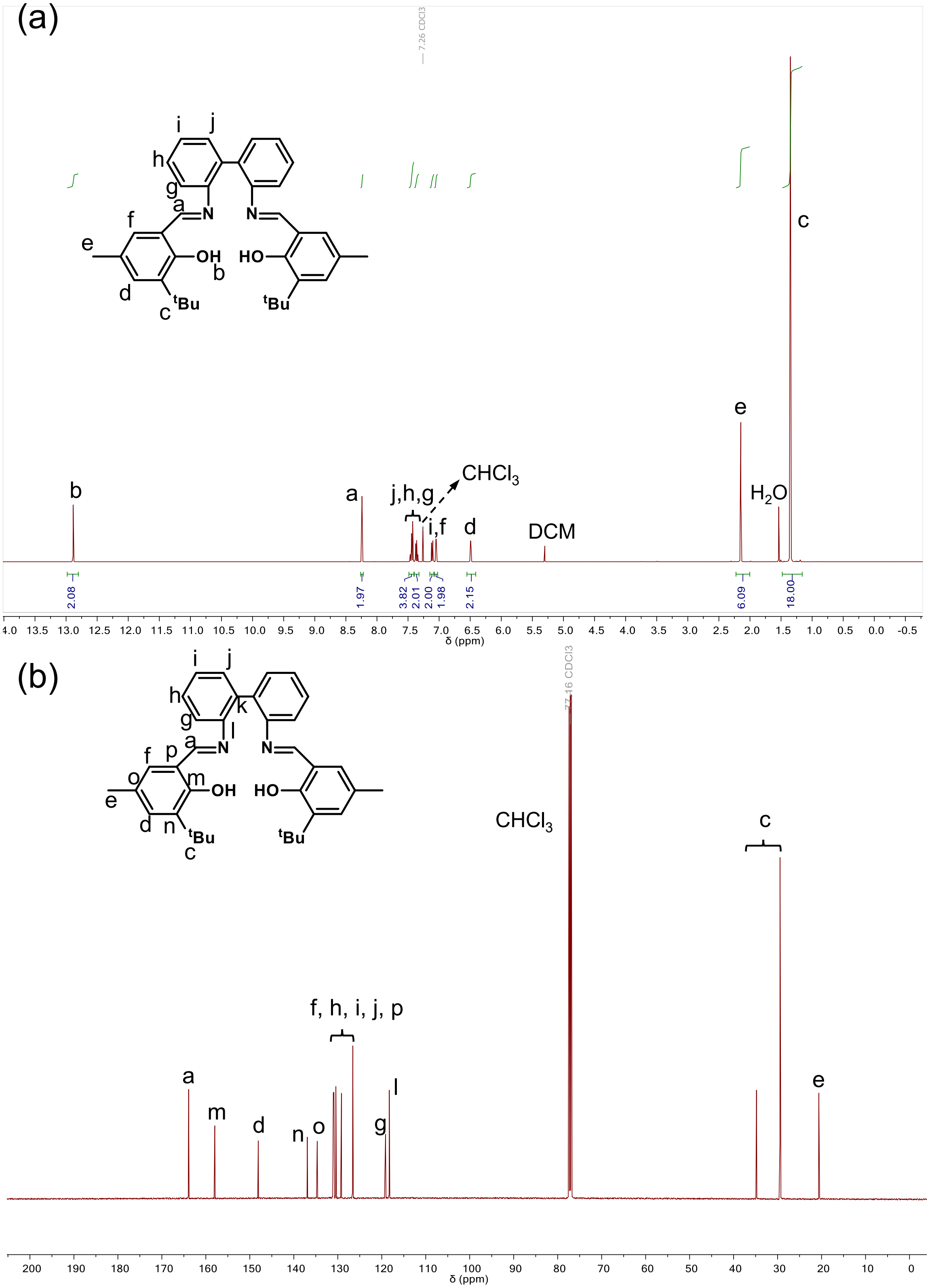

Supplementary Figure 37. (a) ^{1}H NMR (400 MHz, $CDCl_3$) and (b) ^{13}C NMR (125 MHz, $CDCl_3$) spectra of $A_{11}C_8B_1$.

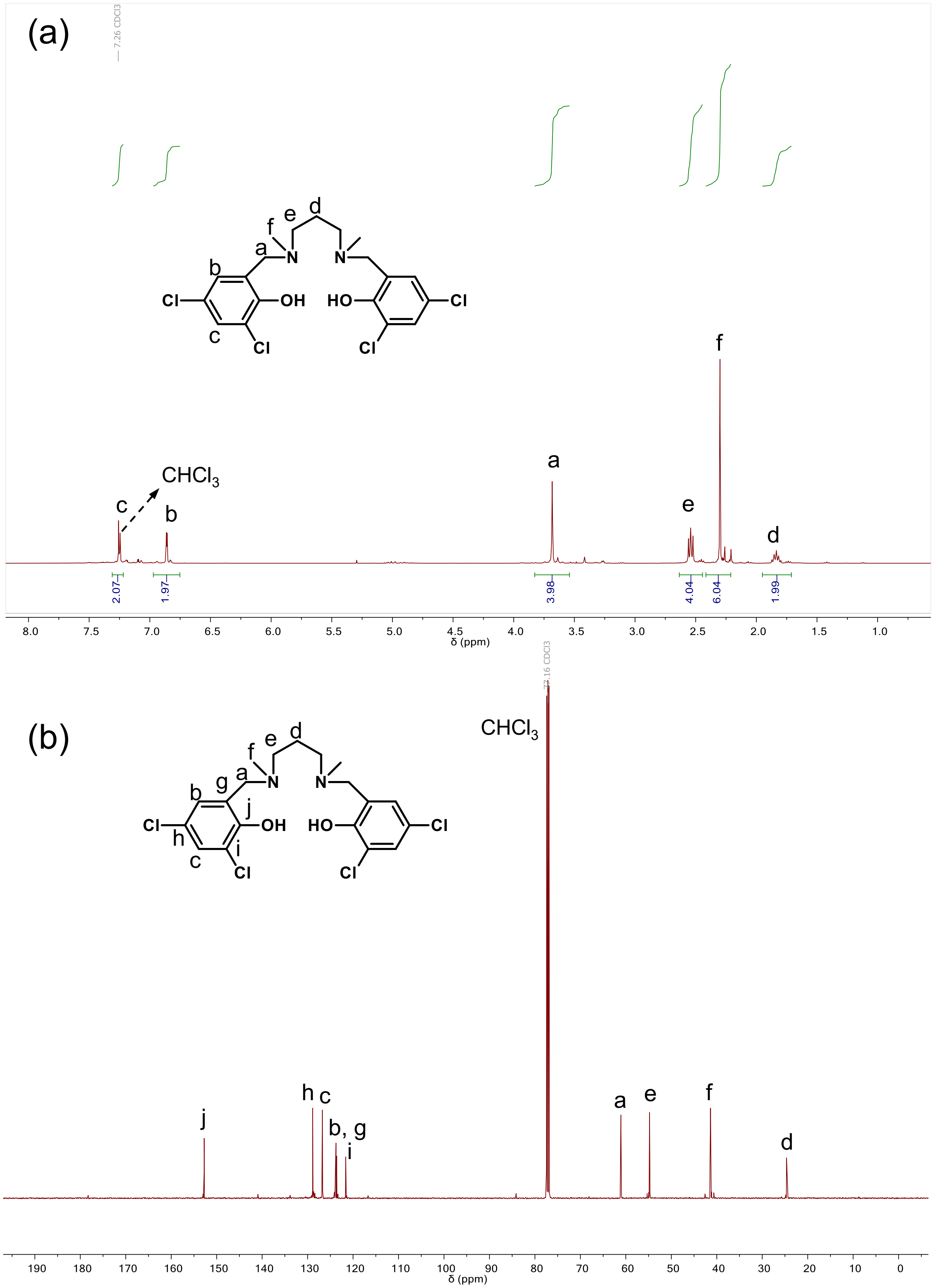

Supplementary Figure 38. (a) ^{1}H NMR (400 MHz, $CDCl_3$) and (b) ^{13}C NMR (125 MHz, $CDCl_3$) spectra of $A_6C_2B_2$.

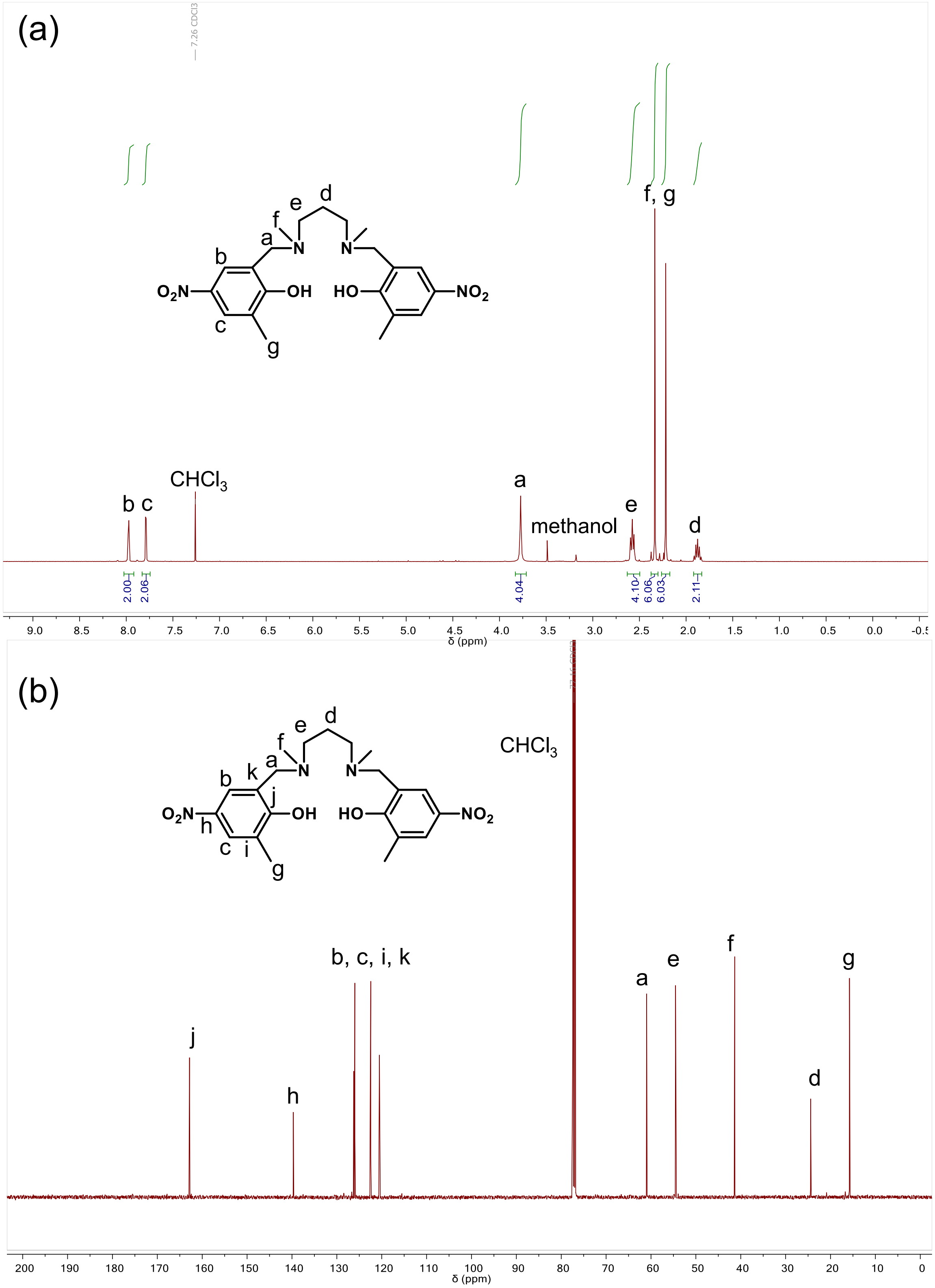

Supplementary Figure 39. (a) ^{1}H NMR (400 MHz, $CDCl_3$) and (b) ^{13}C NMR (125 MHz, $CDCl_3$) spectra of $A_5C_2B_2$.

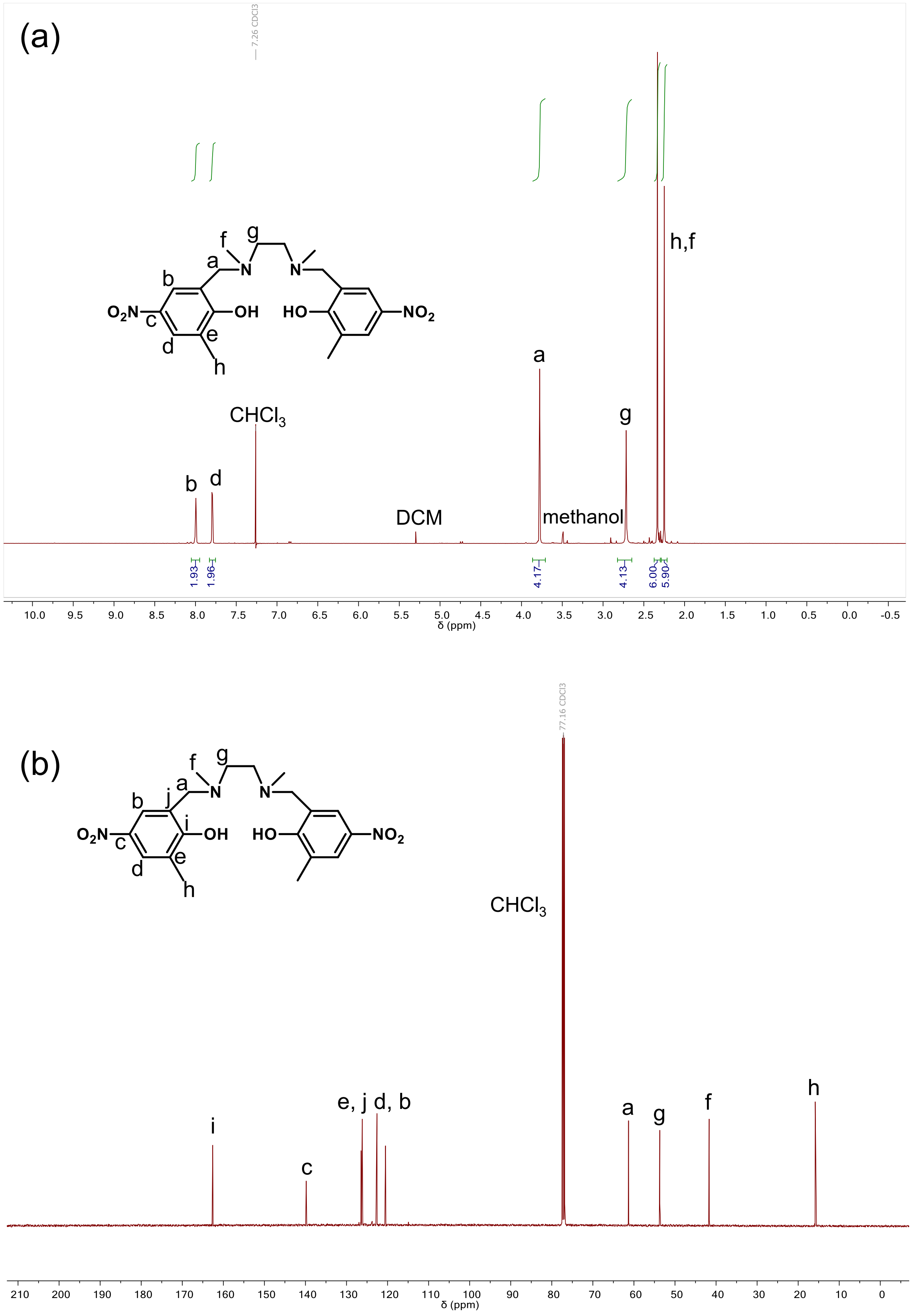

Supplementary Figure 40. (a) ^{1}H NMR (400 MHz, $CDCl_3$) and (b) ^{13}C NMR (125 MHz, $CDCl_3$) spectra of $A_5C_1B_2$.

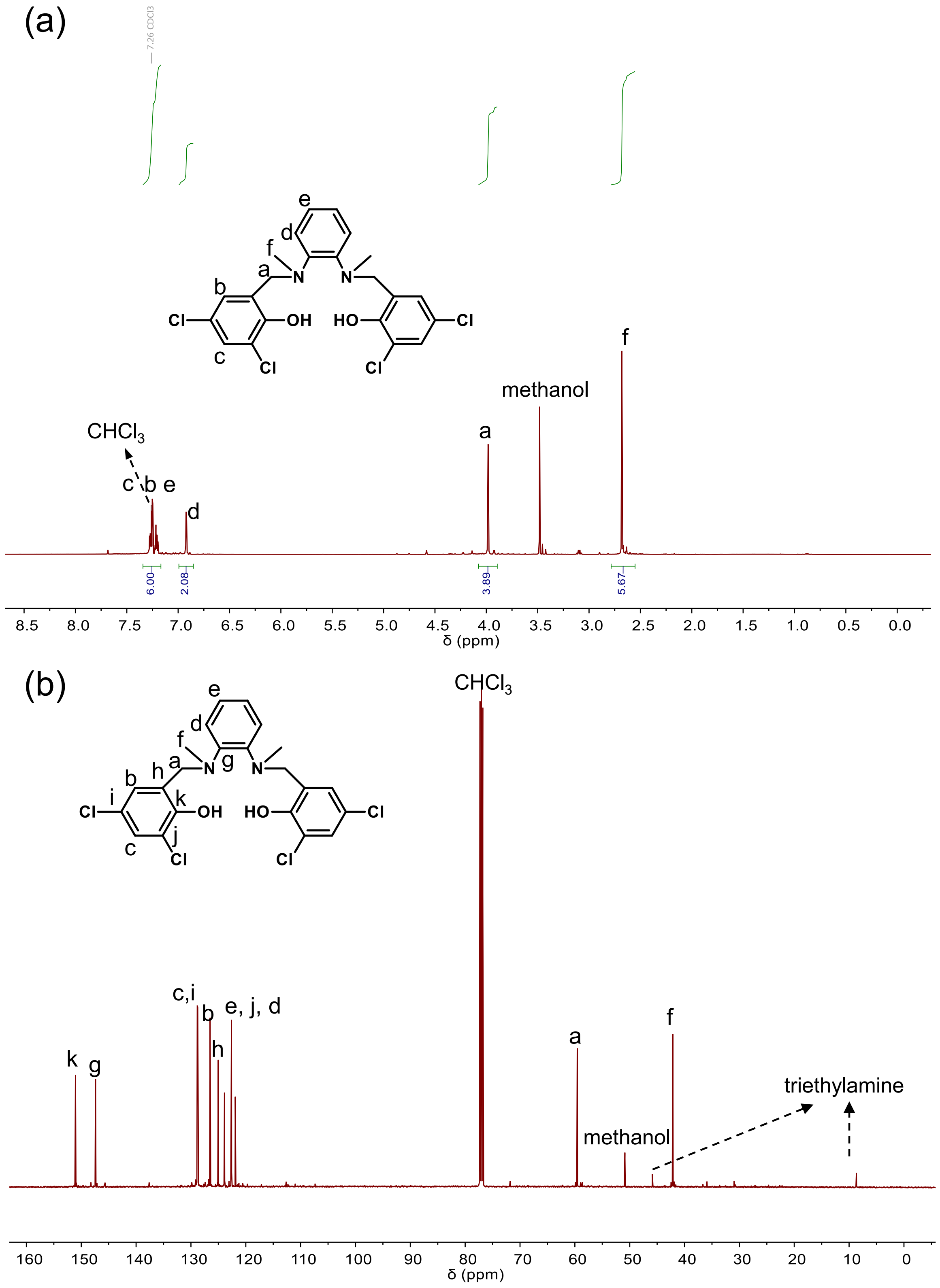

Supplementary Figure 41. (a) ^{1}H NMR (400 MHz, $CDCl_3$) and (b) ^{13}C NMR (125 MHz, $CDCl_3$) spectra of $A_6C_6B_2$.

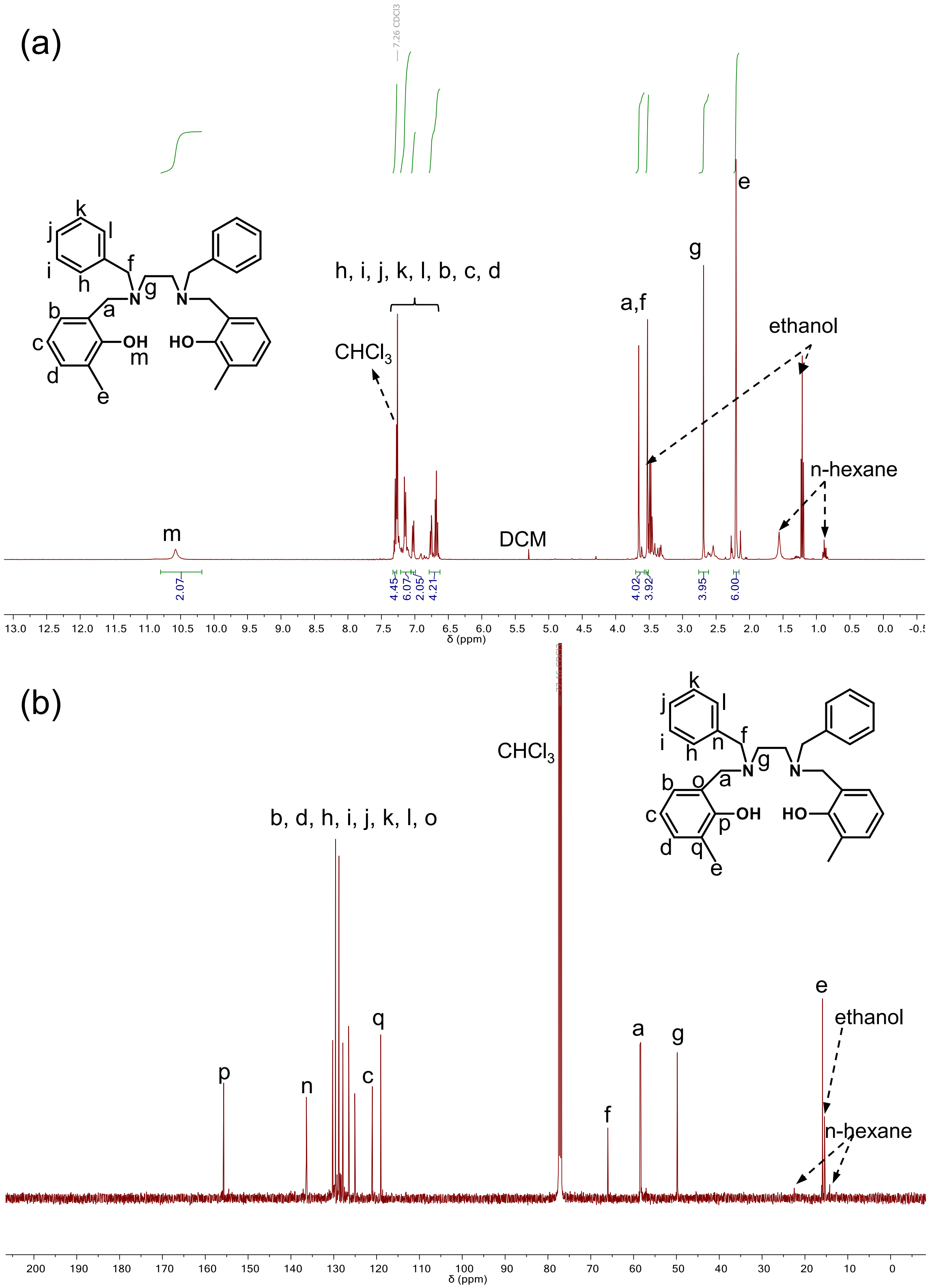

Supplementary Figure 42. (a) ^{1}H NMR (400 MHz, $CDCl_3$) and (b) ^{13}C NMR (125 MHz, $CDCl_3$) spectra of $A_2C_1B_3$.

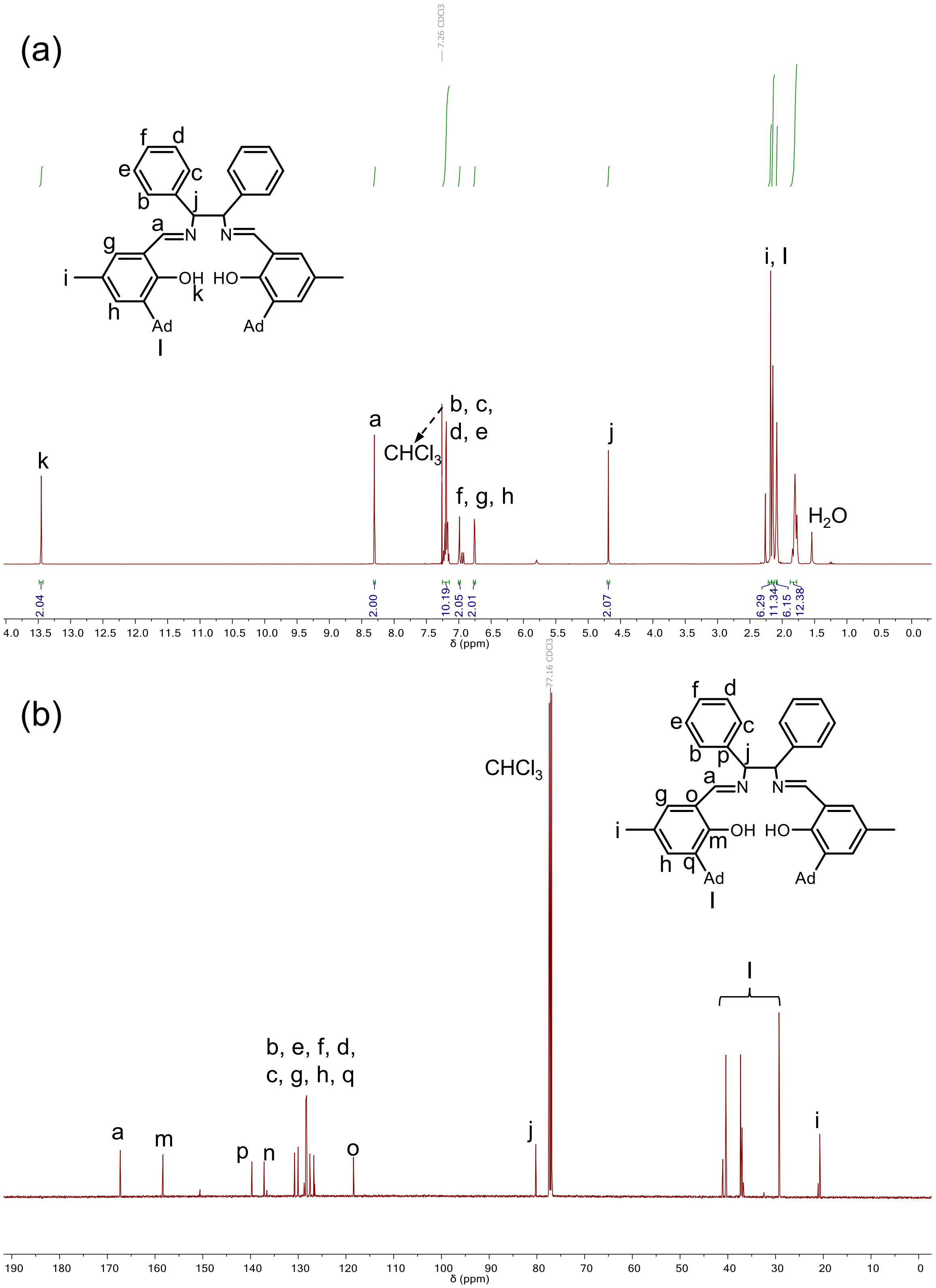

Supplementary Figure 43. (a) ^{1}H NMR (400 MHz, $CDCl_3$) and (b) ^{13}C NMR (125 MHz, $CDCl_3$) spectra of $A_8C_{11}B_1$.

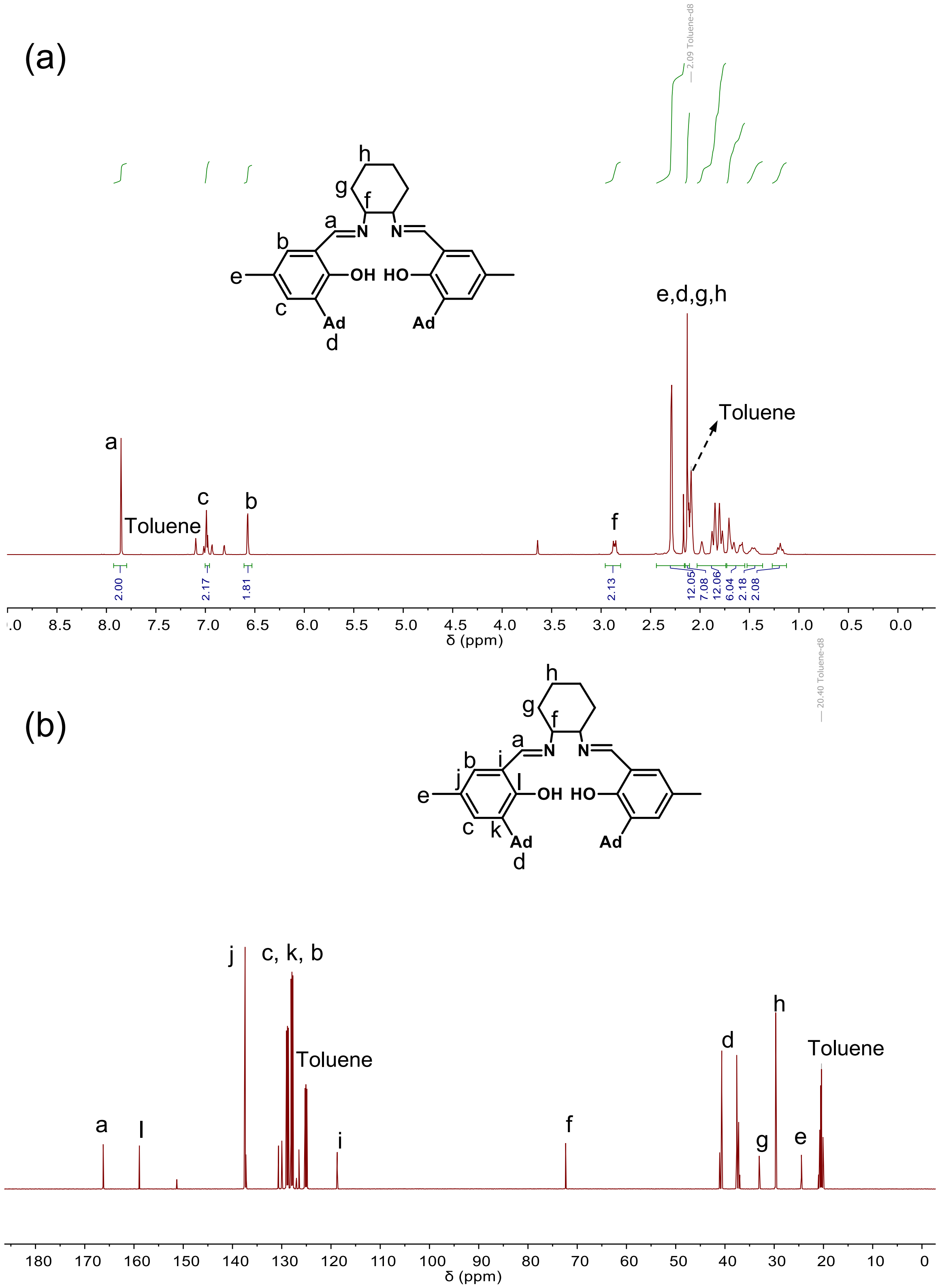

Supplementary Figure 44. (a) ^{1}H NMR (400 MHz, toluene-d_8) and (b) ^{13}C NMR (125 MHz, toluene-d_8) spectra of $A_8C_5B_1$.

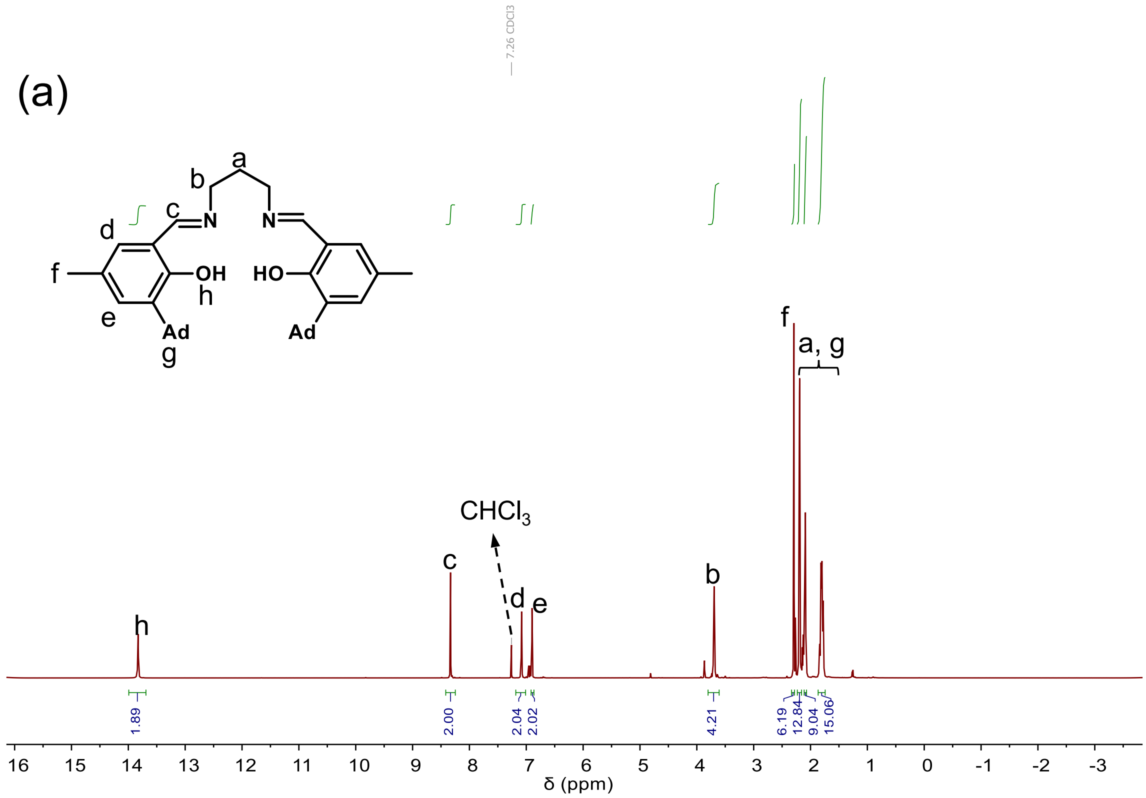

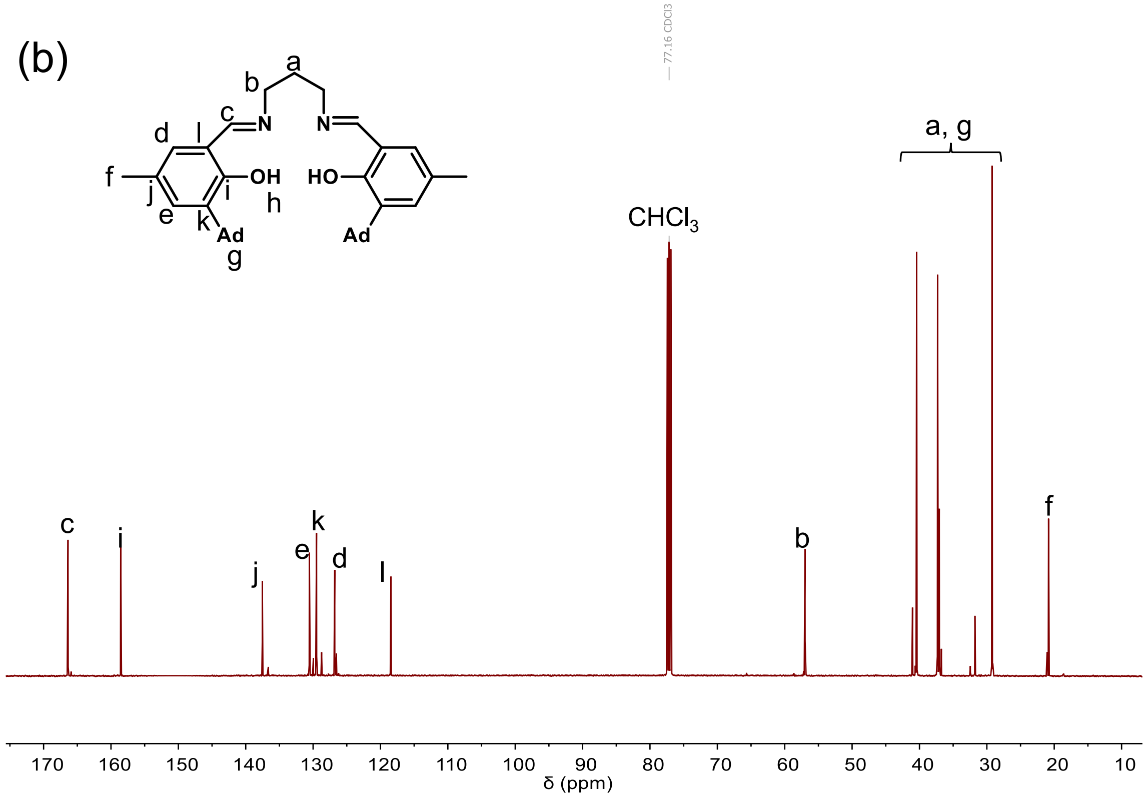

Supplementary Figure 45. (a) ^{1}H NMR (400 MHz, $CDCl_3$) and (b) ^{13}C NMR (125 MHz, $CDCl_3$) spectra of $A_8C_2B_1$.

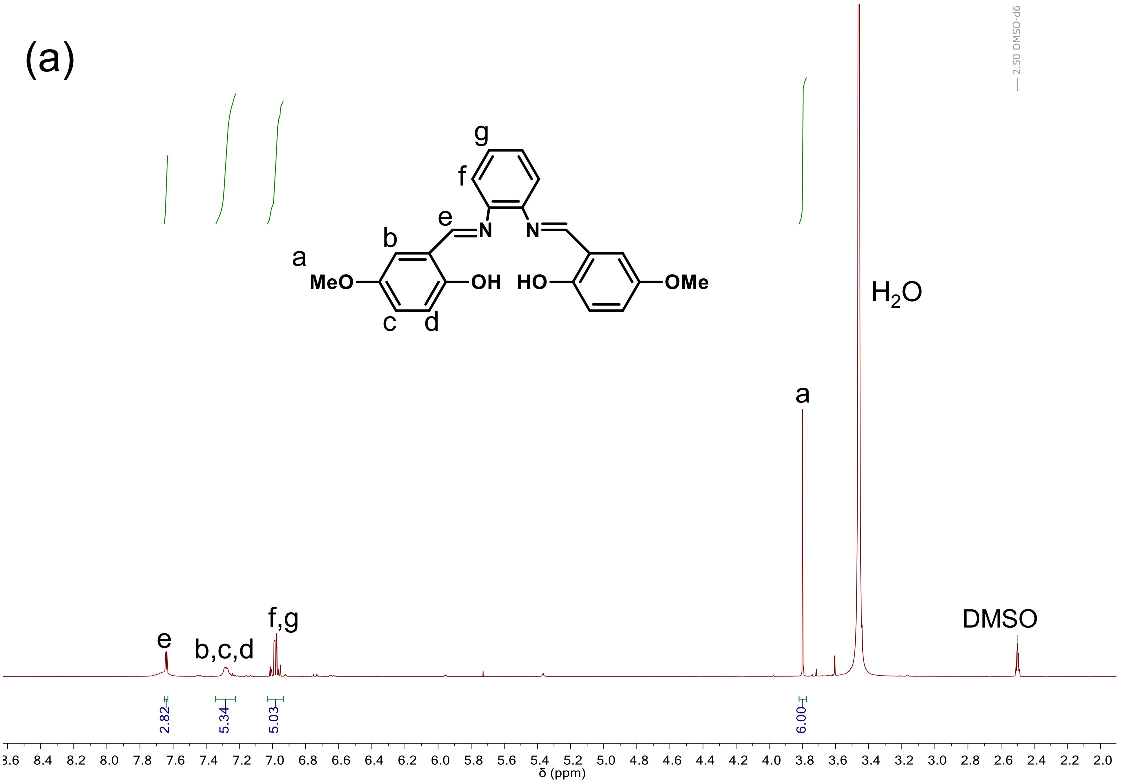

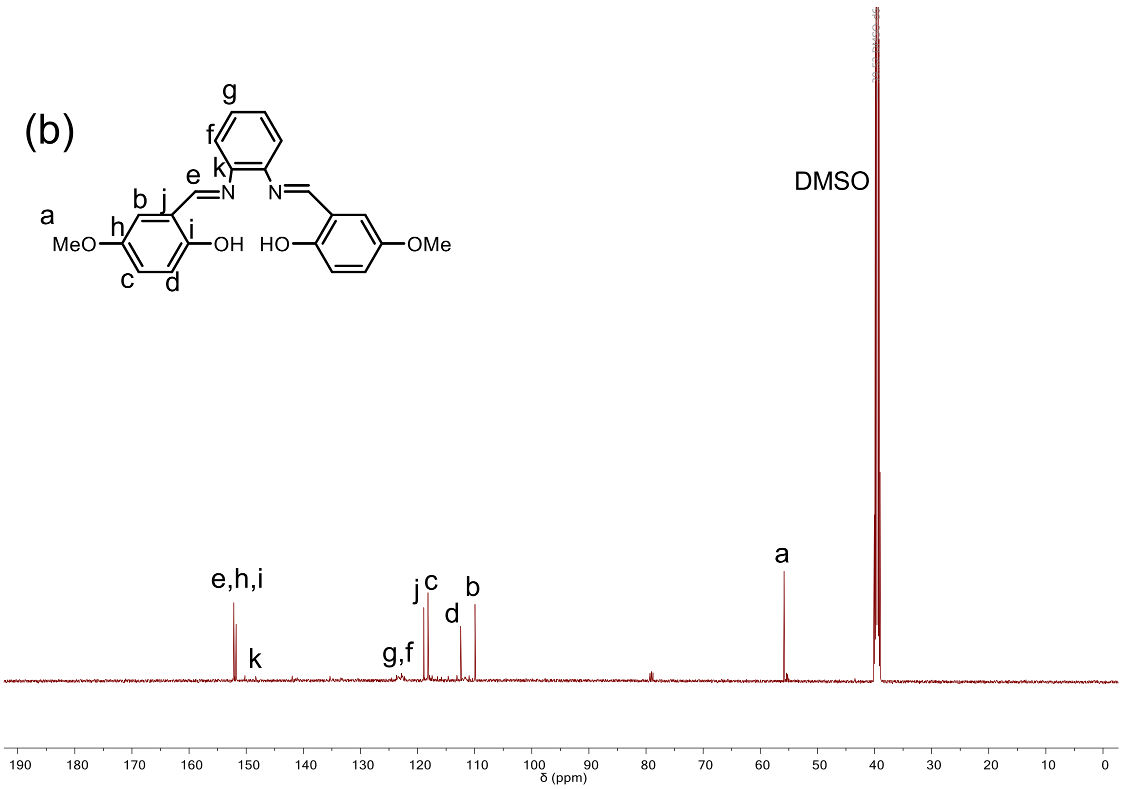

Supplementary Figure 46. (a) ^{1}H NMR (400 MHz, DMSO-d_6) and (b) ^{13}C NMR (125 MHz, DMSO-d_6) spectra of $A_4C_6B_1$.

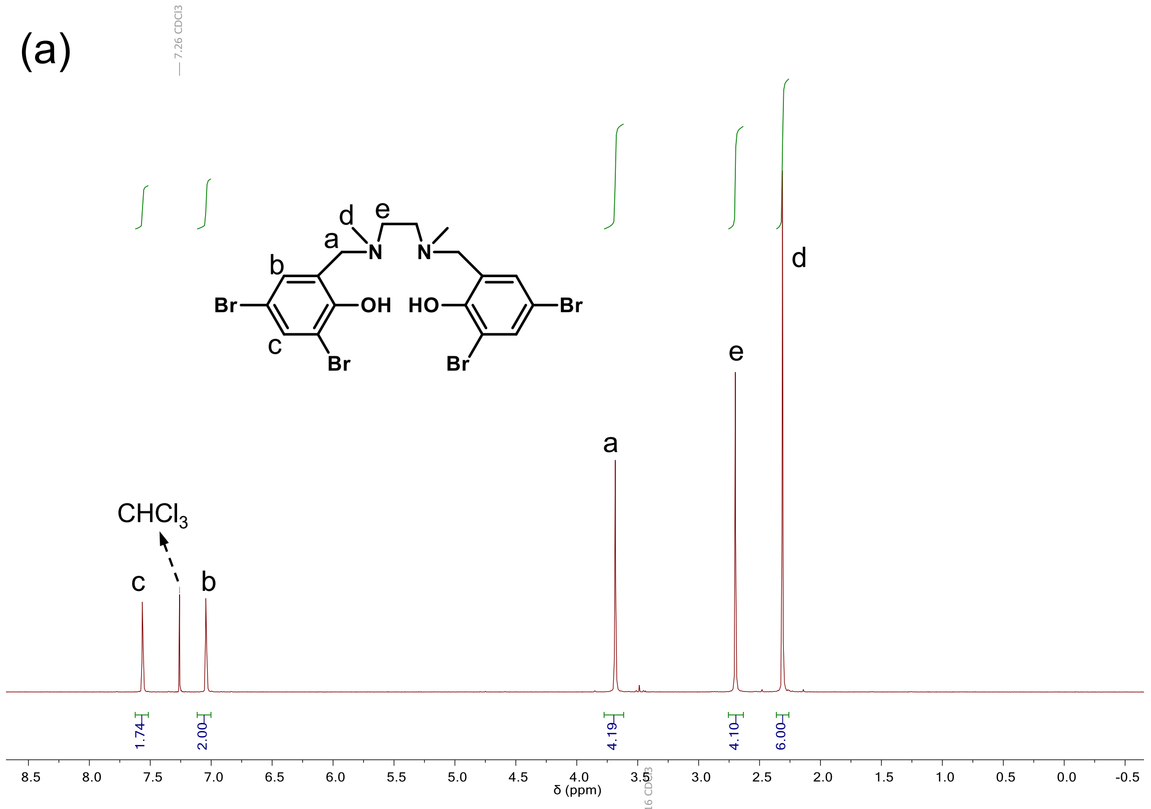

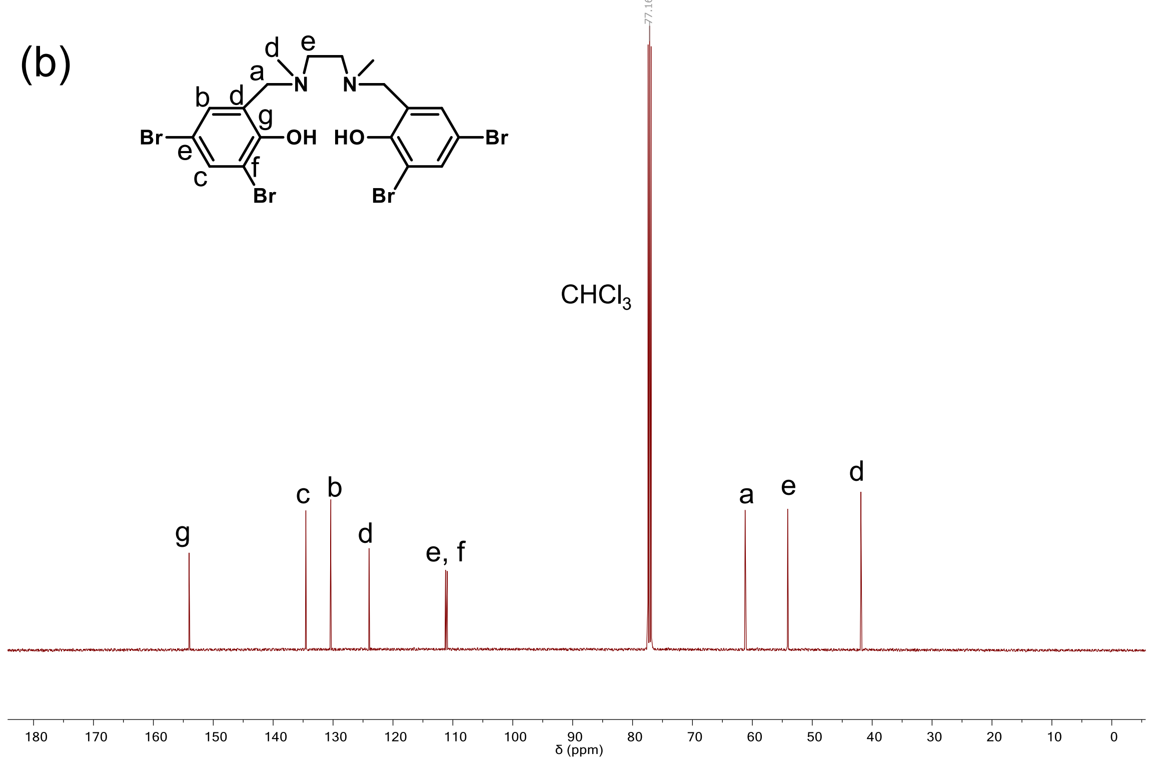

Supplementary Figure 47. (a) ^{1}H NMR (400 MHz, $CDCl_3$) and (b) ^{13}C NMR (125 MHz, $CDCl_3$) spectra of $A_4C_6B_1$.

(a)

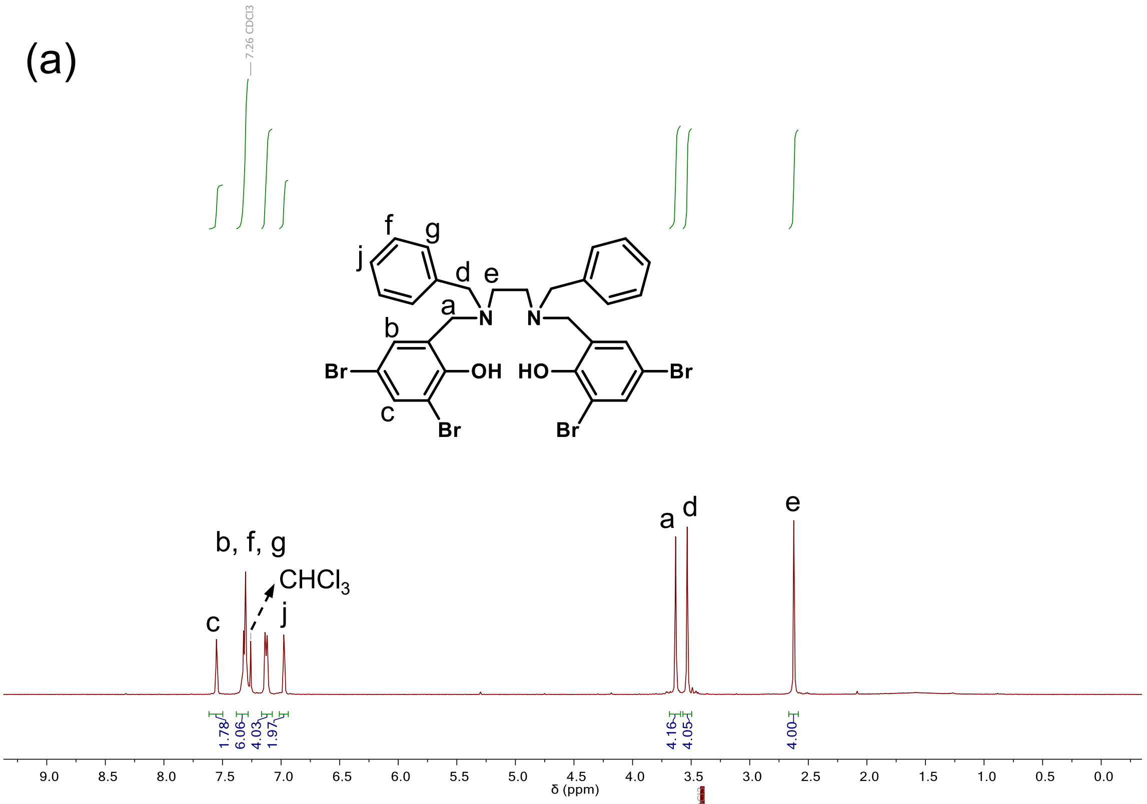

(b)

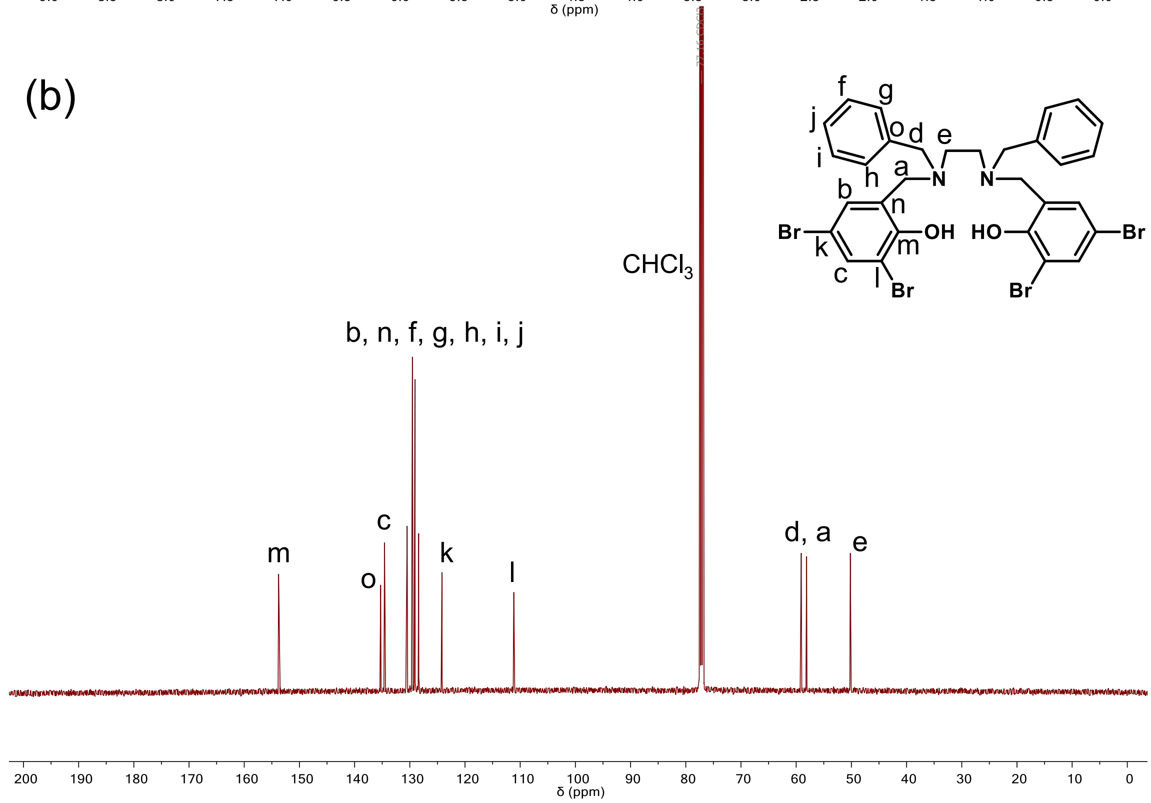

Supplementary Figure 48. (a) ^{1}H NMR (400 MHz, $CDCl_3$) and (b) ^{13}C NMR (125 MHz, $CDCl_3$) spectra of $A_{16}C_1B_3$.

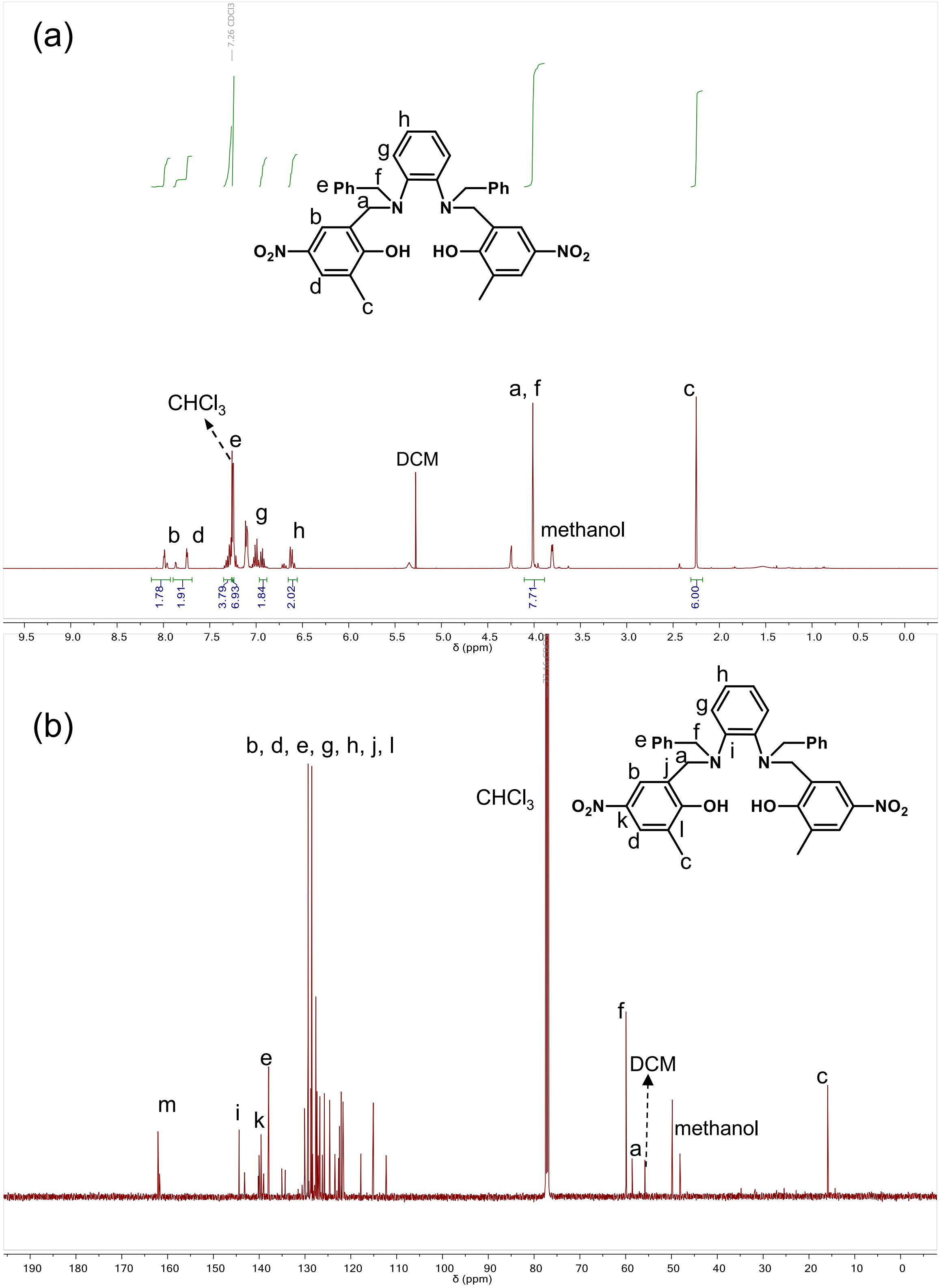

Supplementary Figure 49. (a) ^{1}H NMR (400 MHz, $CDCl_3$) and (b) ^{13}C NMR (125 MHz, $CDCl_3$) spectra of $A_5C_6B_3$.

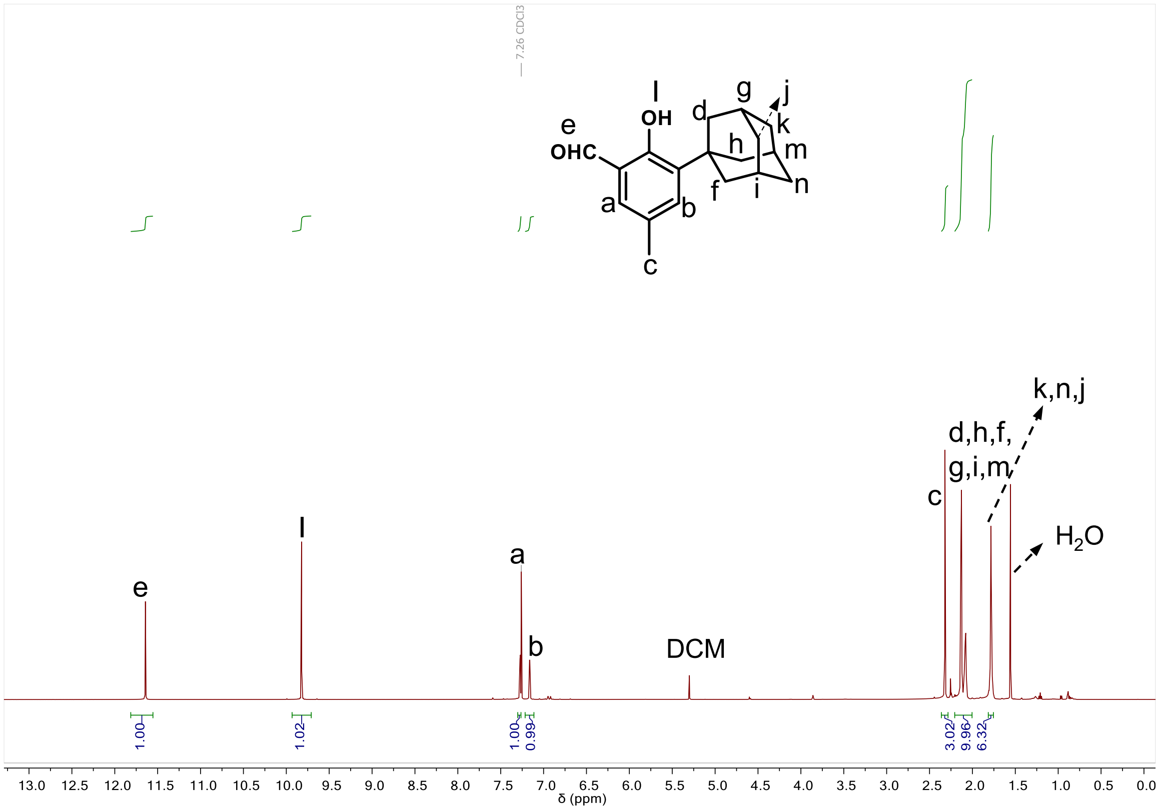

Supplementary Figure 50. ^{1}H NMR spectrum (400 MHz, $CDCl_3$) of A_8-CHO

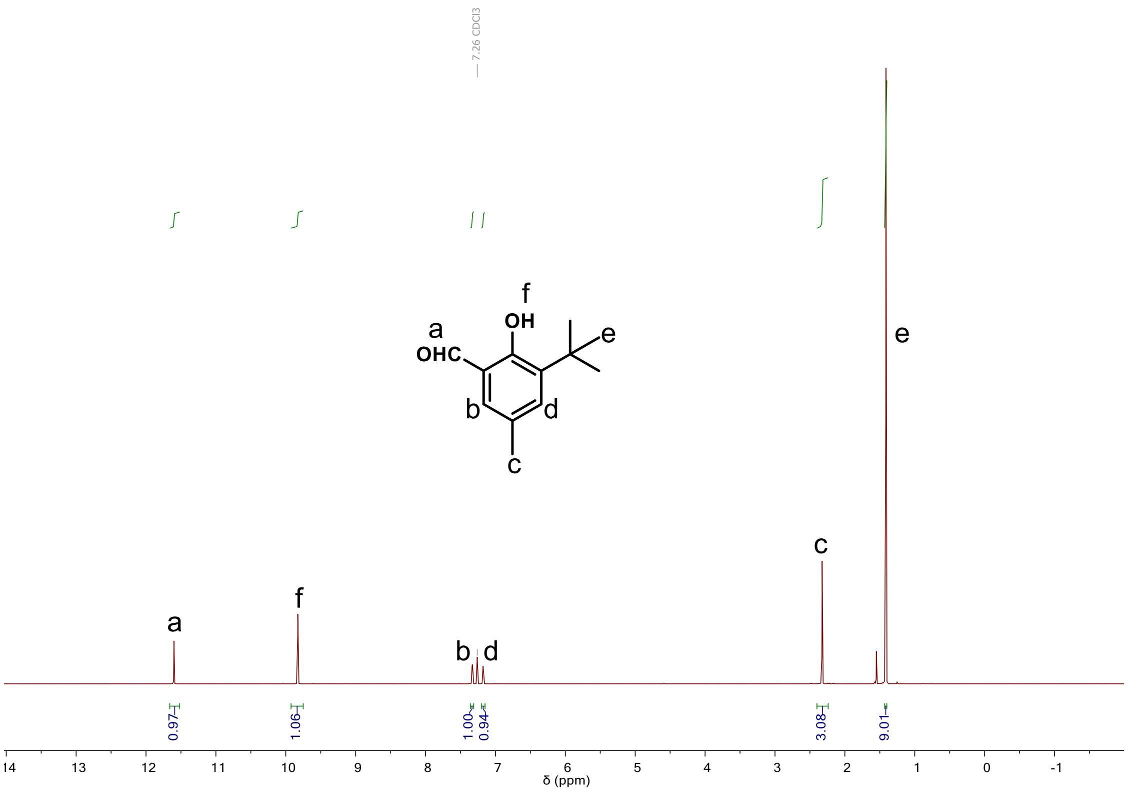

Supplementary Figure 51. ^{1}H NMR spectrum (400 MHz, $CDCl_3$) of A_{11}-CHO

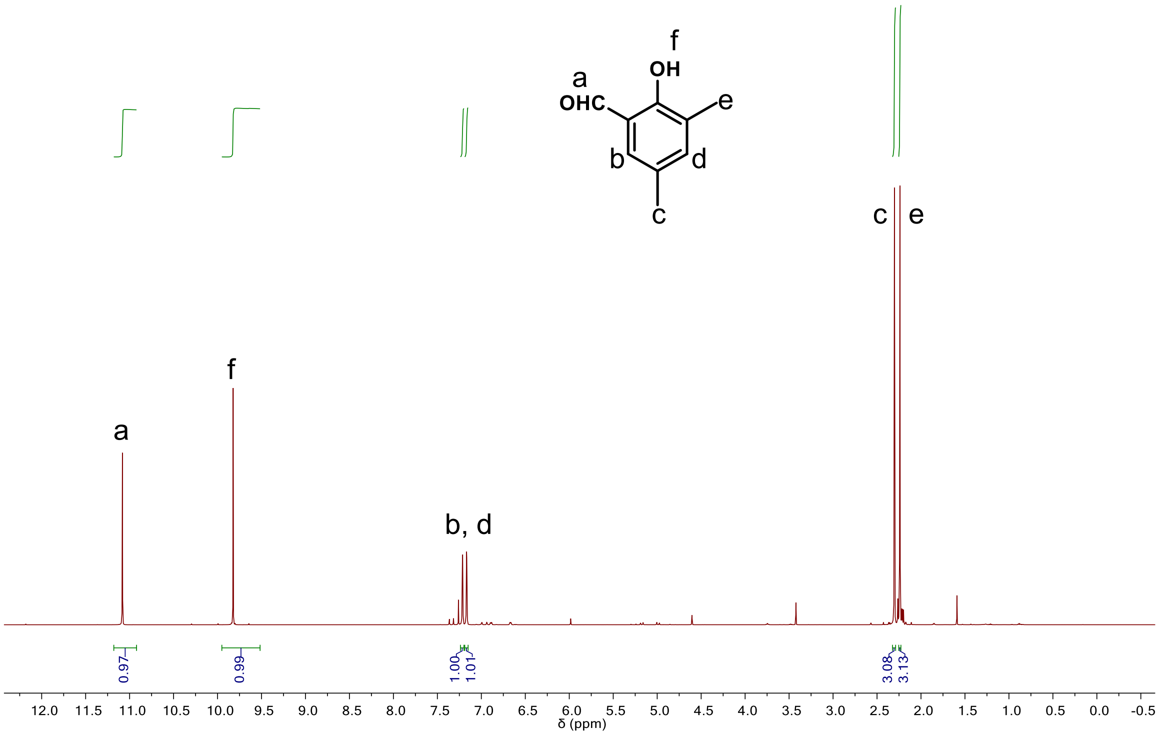

Supplementary Figure 52. ^{1}H NMR spectrum (400 MHz, $CDCl_3$) of A_{14}-CHO.

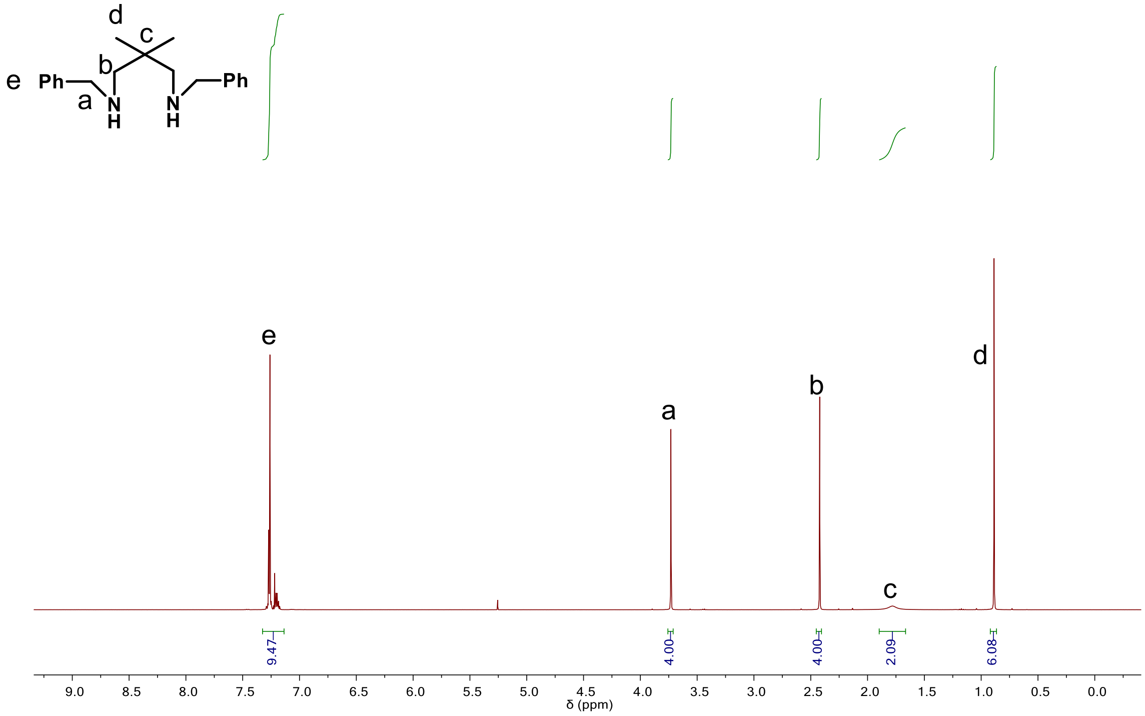

Supplementary Figure 53. ^{1}H NMR spectrum (400 MHz, $CDCl_3$) of C_3B_3.

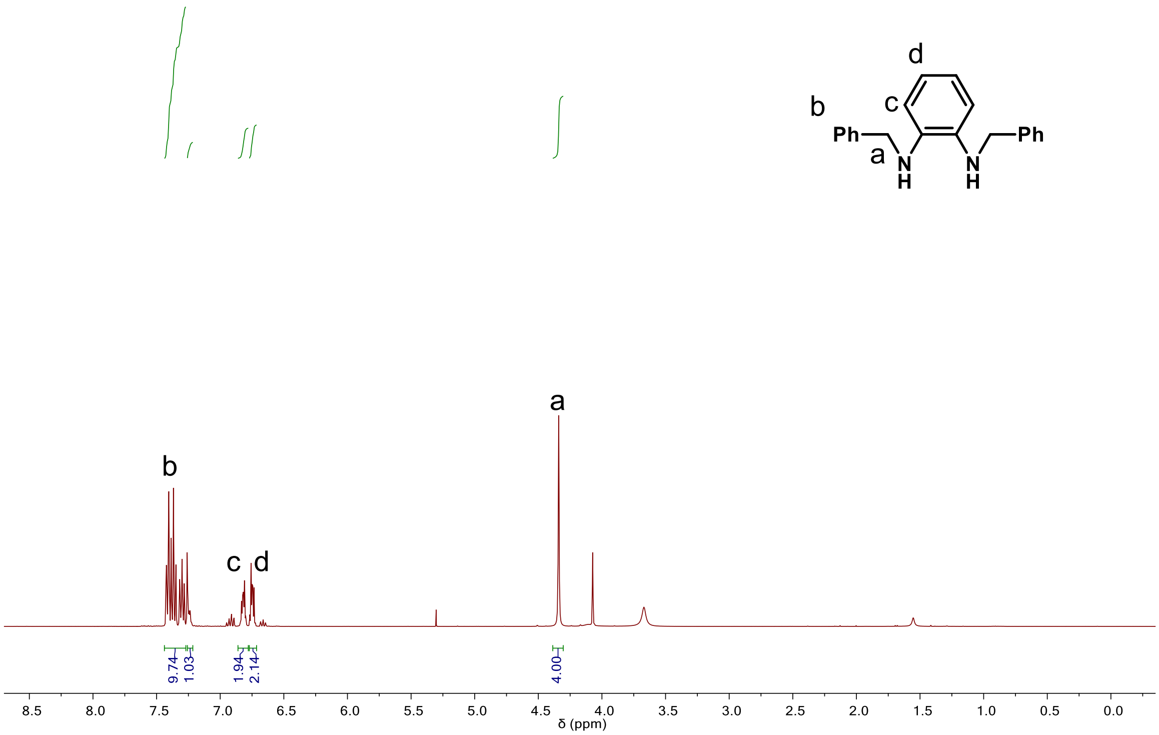

Supplementary Figure 54. ^{1}H NMR spectrum (400 MHz, $CDCl_3$) of C_6B_3.

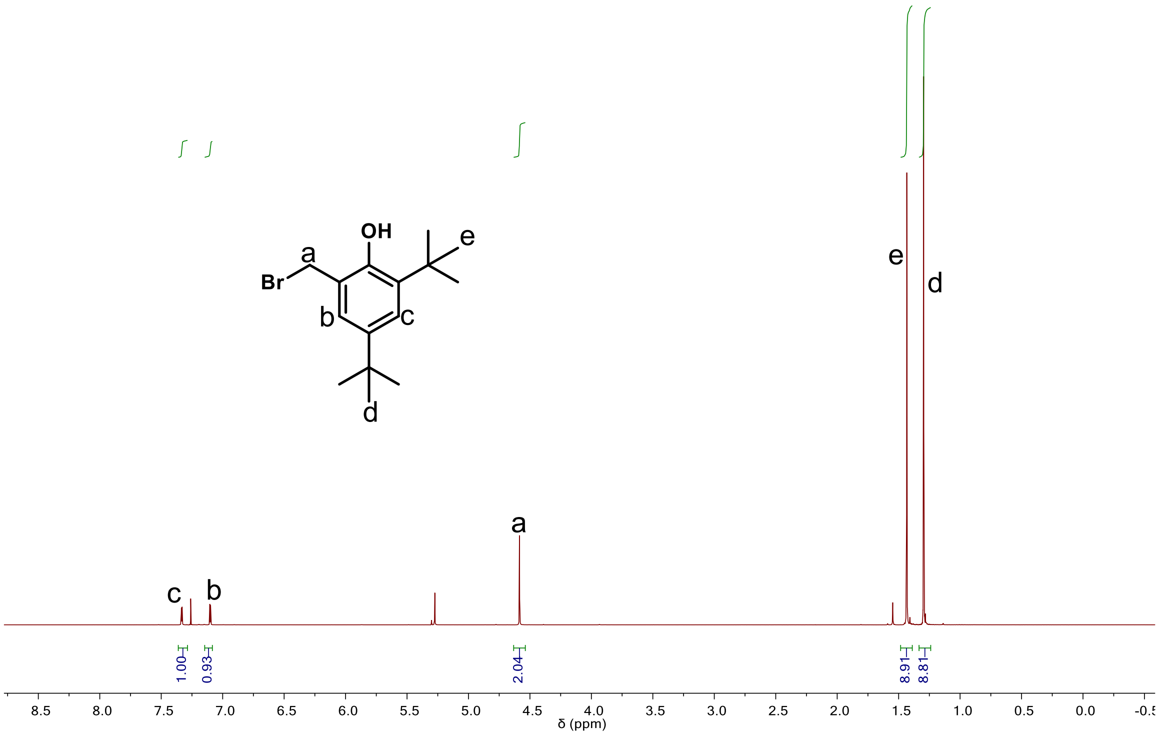

Supplementary Figure 55. ^{1}H NMR spectrum (400 MHz, $CDCl_3$) of A_3-CH_2Br.

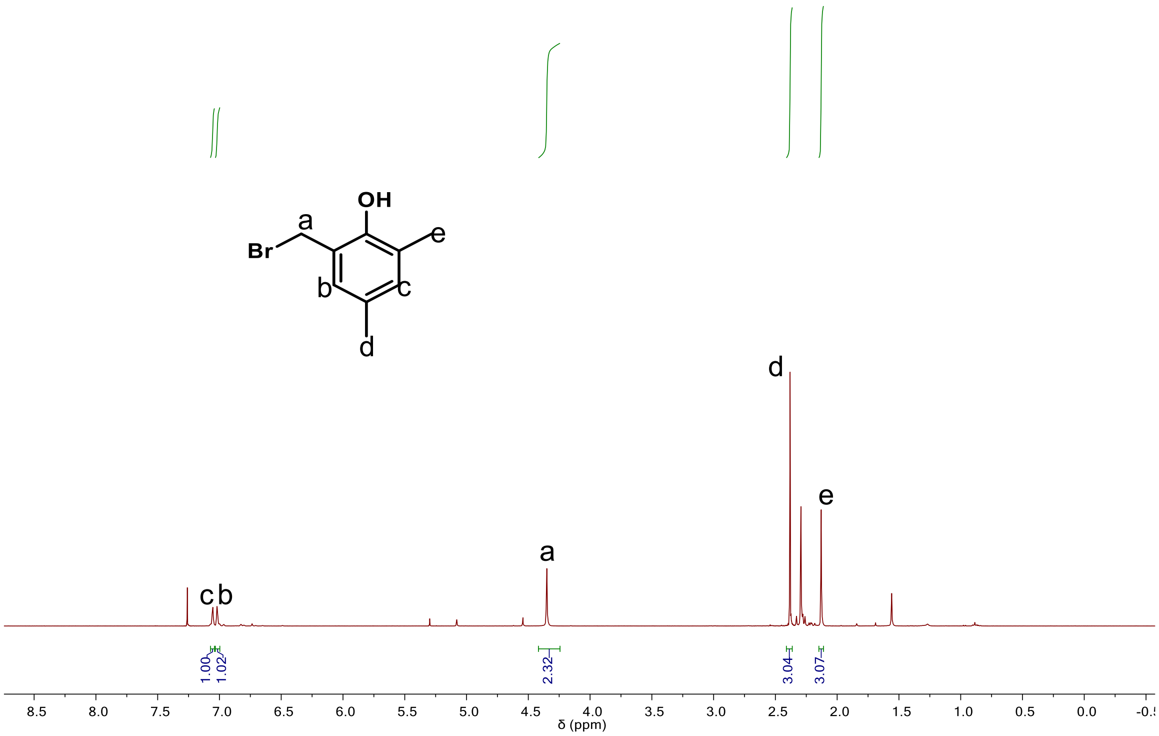

Supplementary Figure 56. ^{1}H NMR spectrum (400 MHz, $CDCl_3$) of A_{14}-CH_2Br.

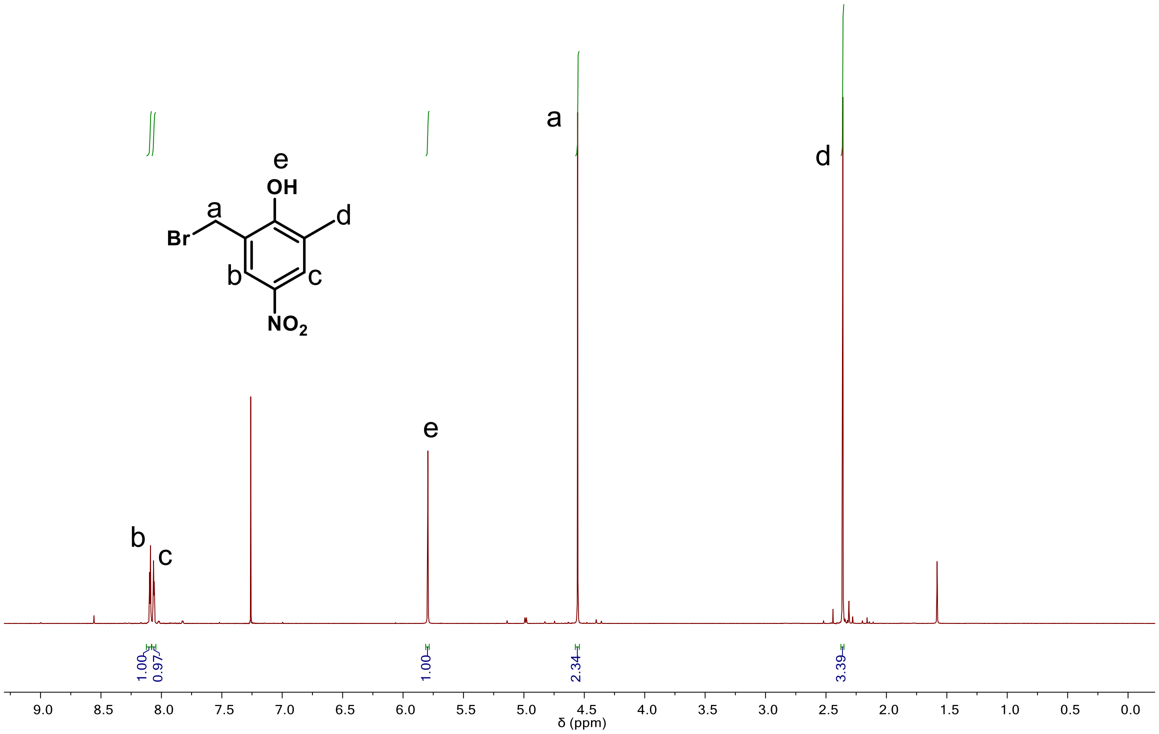

Supplementary Figure 57. ^{1}H NMR spectrum (400 MHz, $CDCl_3$) of A_5-CH_2Br.

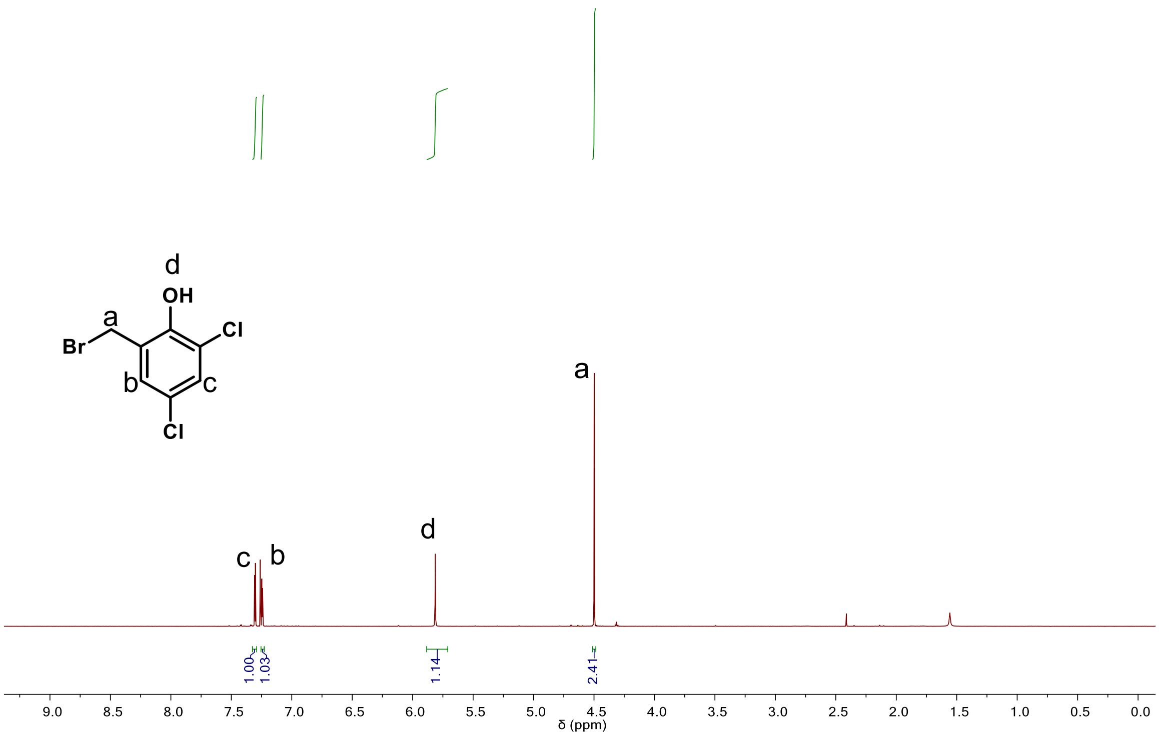

Supplementary Figure 58. ^{1}H NMR spectrum (400 MHz, $CDCl_3$) of A_6-CH_2Br.

S8. NMR Spectra of Al complexes

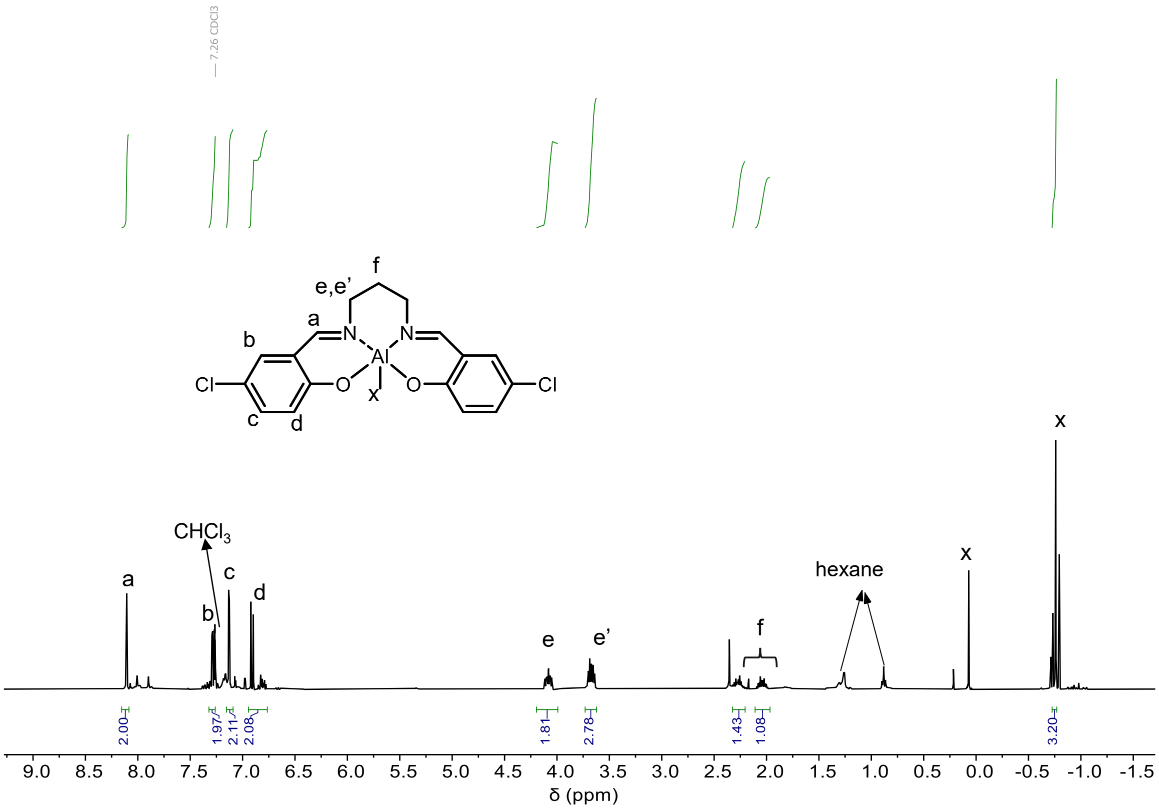

Supplementary Figure 59. ^{1}H NMR (400 MHz, $CDCl_3$) spectrum of ($A_7C_2B_1$)AlMe. We note that the Al complex had low solubility in $CDCl_3$. We also tested toluene-d_8 and THF-d_8, and none of them showed improved solution of the NMR spectrum. ^{1}H NMR showed the aggregation status of the complex in the solution. ESI-MS confirmed the complex.

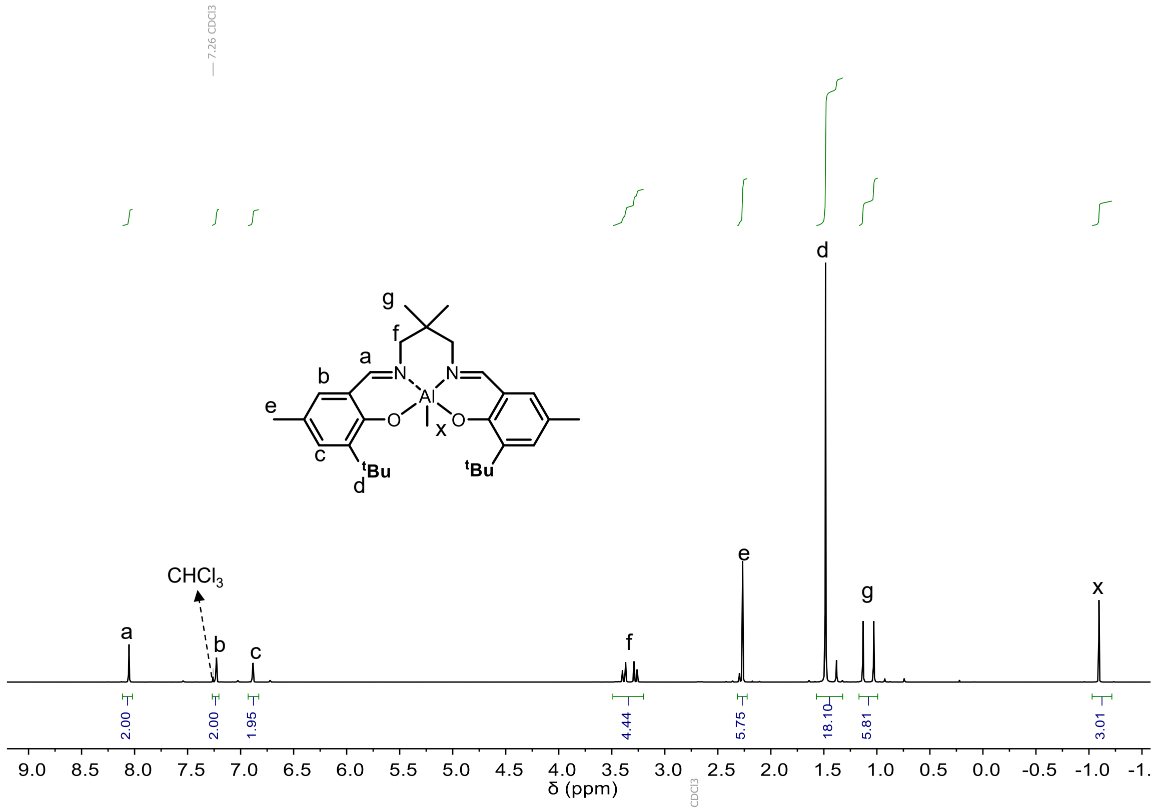

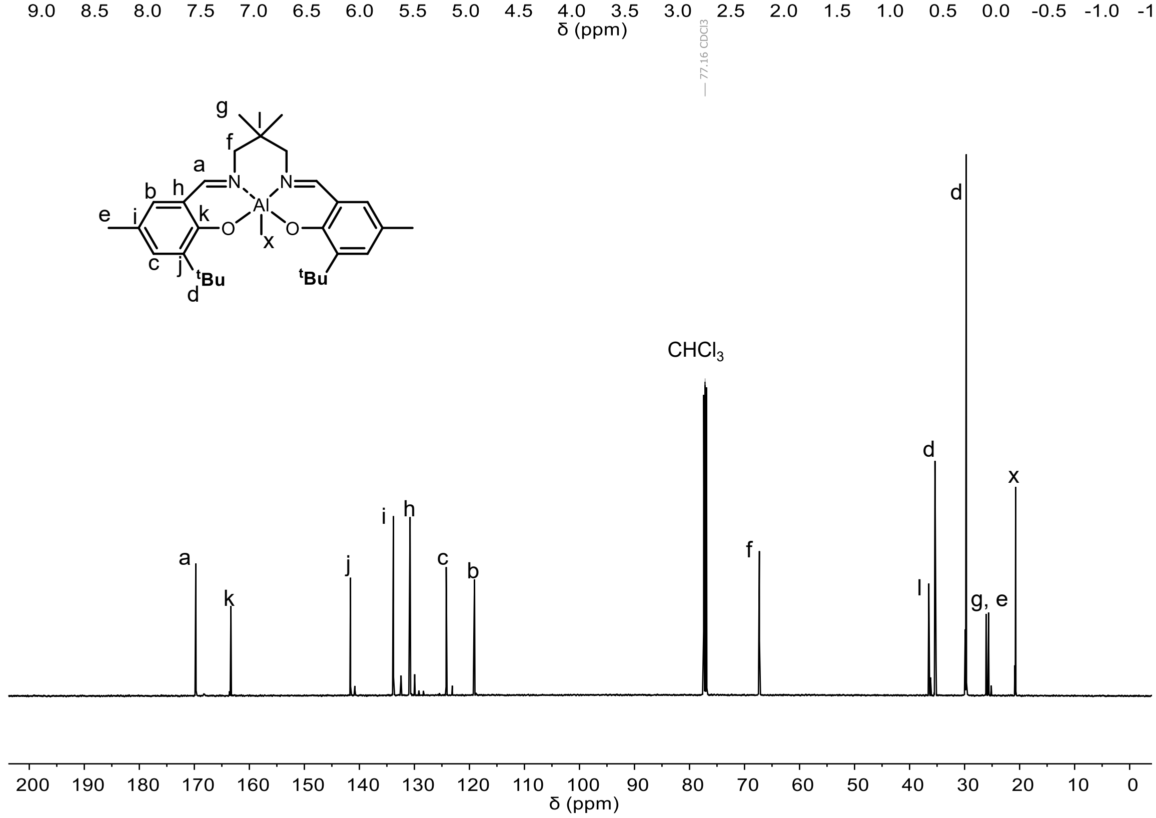

Supplementary Figure 60. ^{1}H NMR (400 MHz, $CDCl_3$) and ^{13}C NMR (125 MHz, $CDCl_3$) spectra of $(A_{11}C_3B_1)AlMe$.

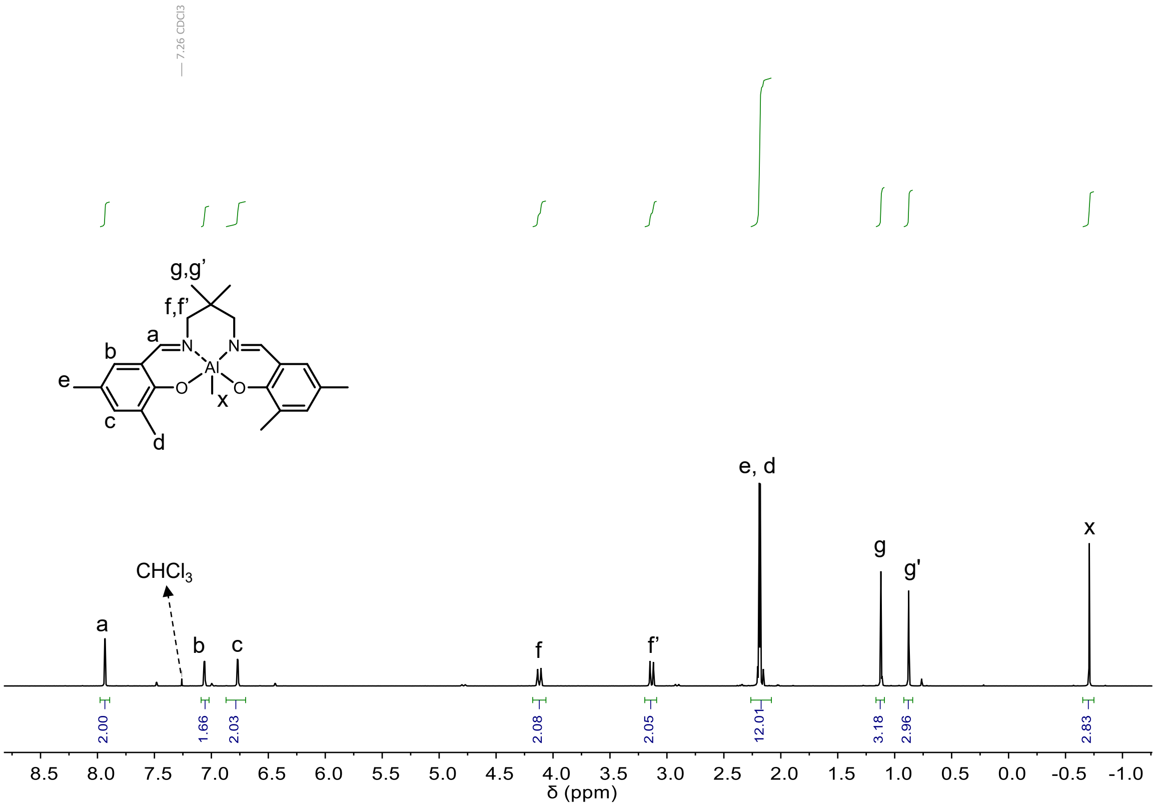

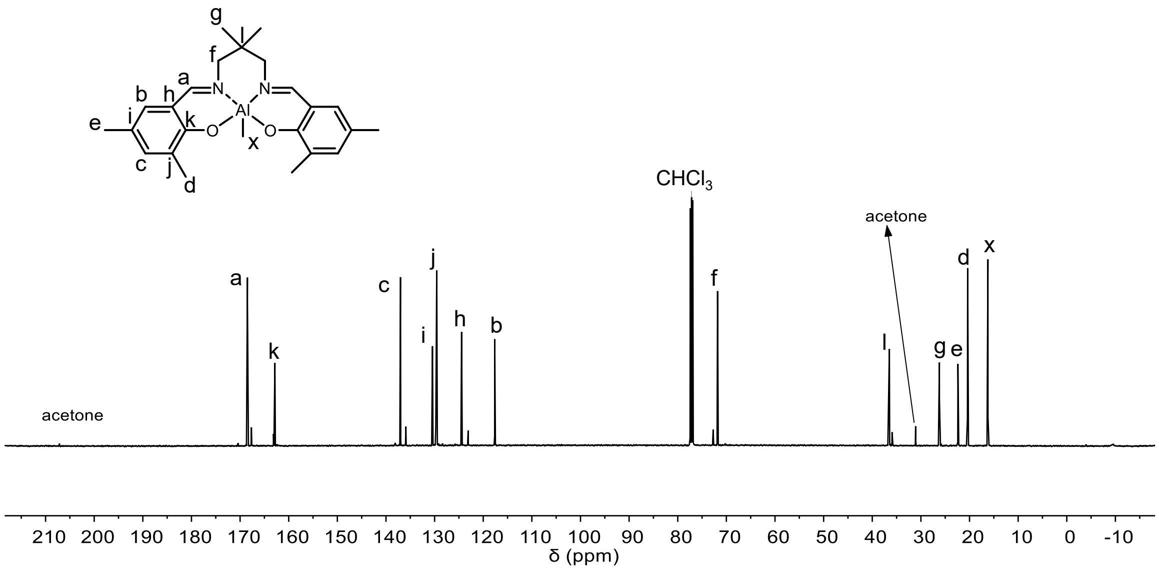

Supplementary Figure 61. ^{1}H NMR (400 MHz, $CDCl_3$) and ^{13}C NMR (125 MHz, $CDCl_3$) spectra of $(A_{14}C_3B_1)AlMe$.

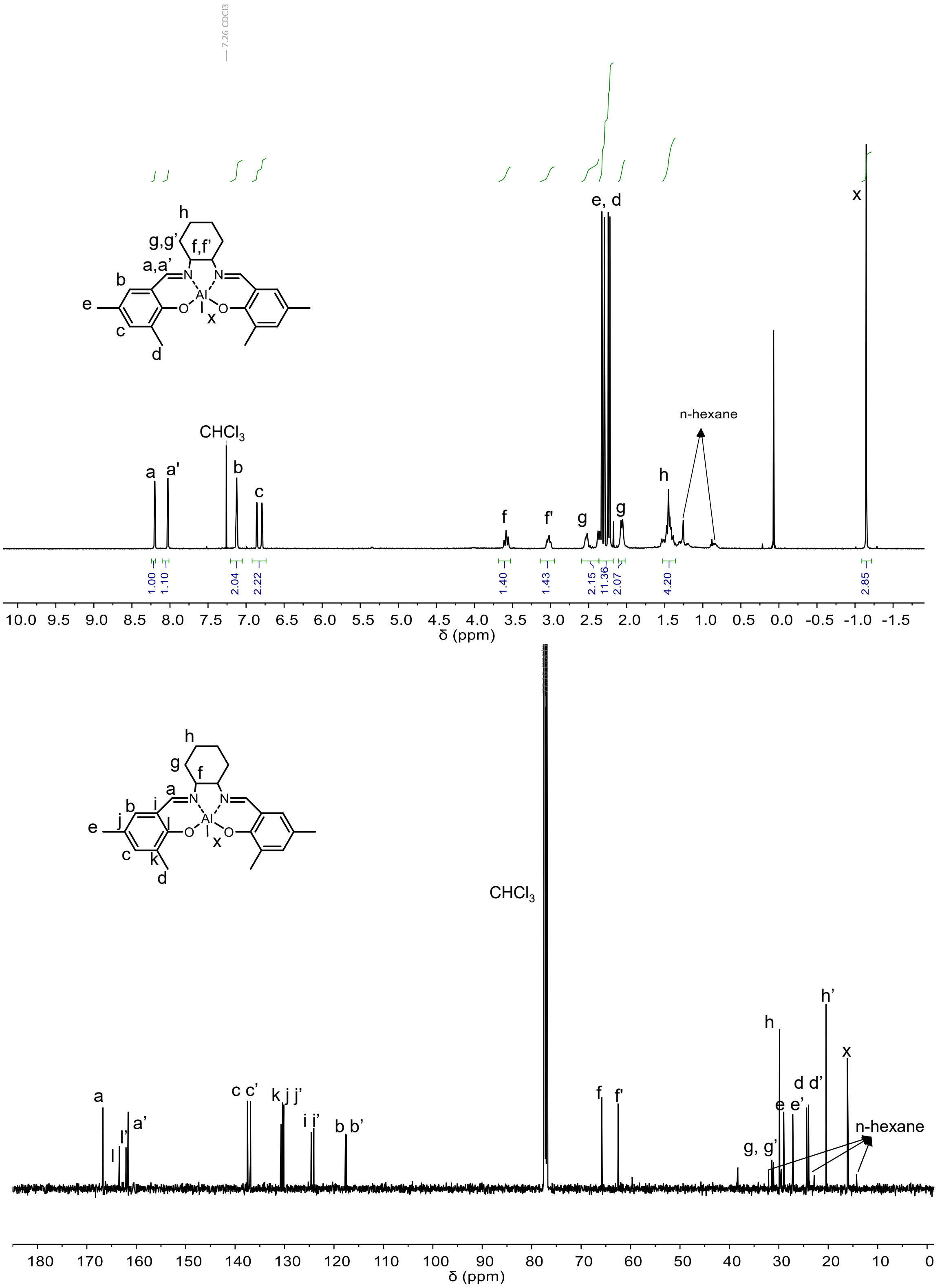

Supplementary Figure 62. ^{1}H NMR (400 MHz, $CDCl_3$) and ^{13}C NMR (125 MHz, $CDCl_3$) spectra of $(A_{14}C_5B_1)$AlMe (isomer structures as we use *rac*-C_5 in the ligand).

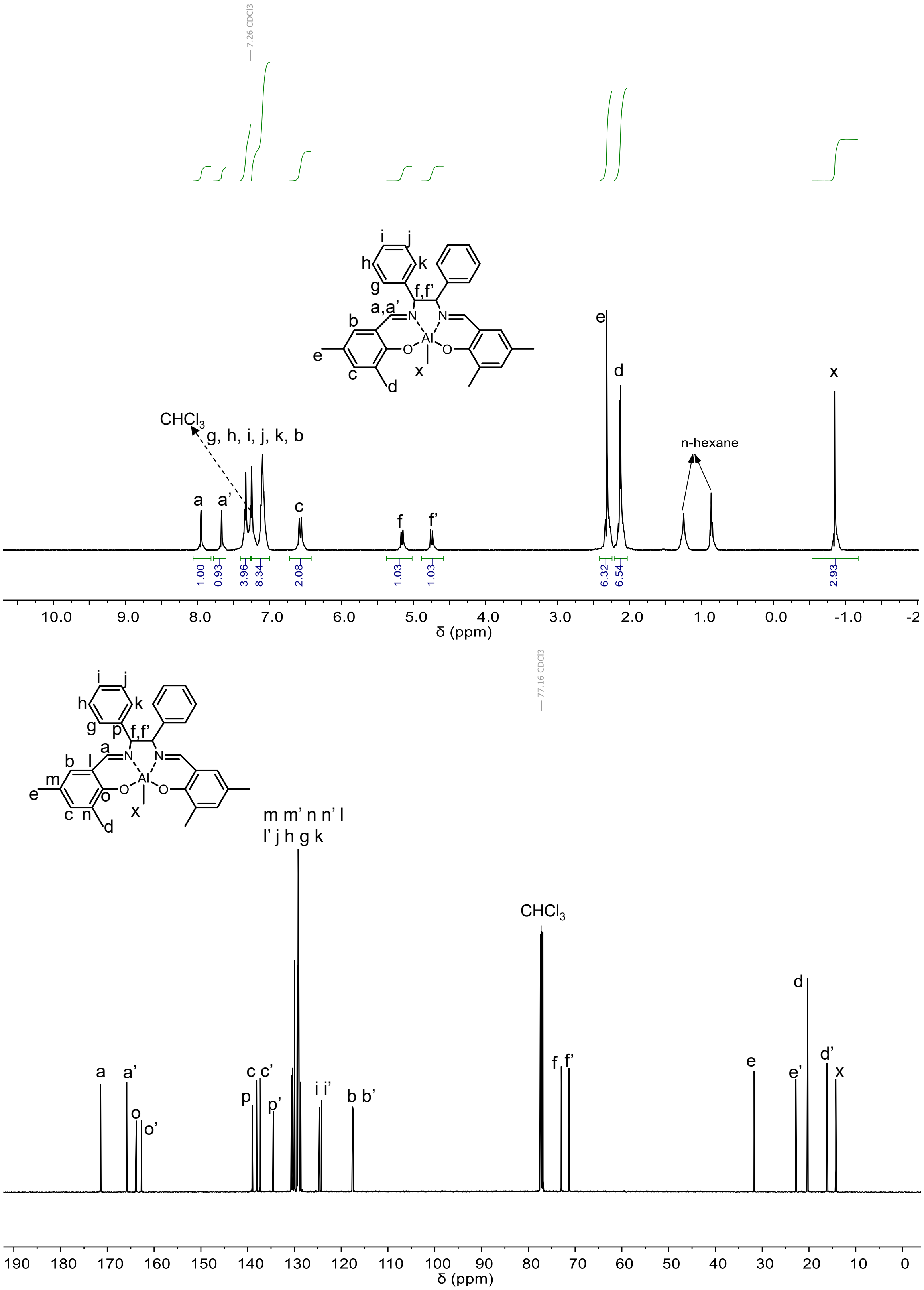

Supplementary Figure 63. ^{1}H NMR (400 MHz, $CDCl_3$) and ^{13}C NMR (125 MHz, $CDCl_3$) spectra of ($A_{14}C_{11}B_1$)AlMe (isomer structures as we used *rac*-C_{11} in the ligand).

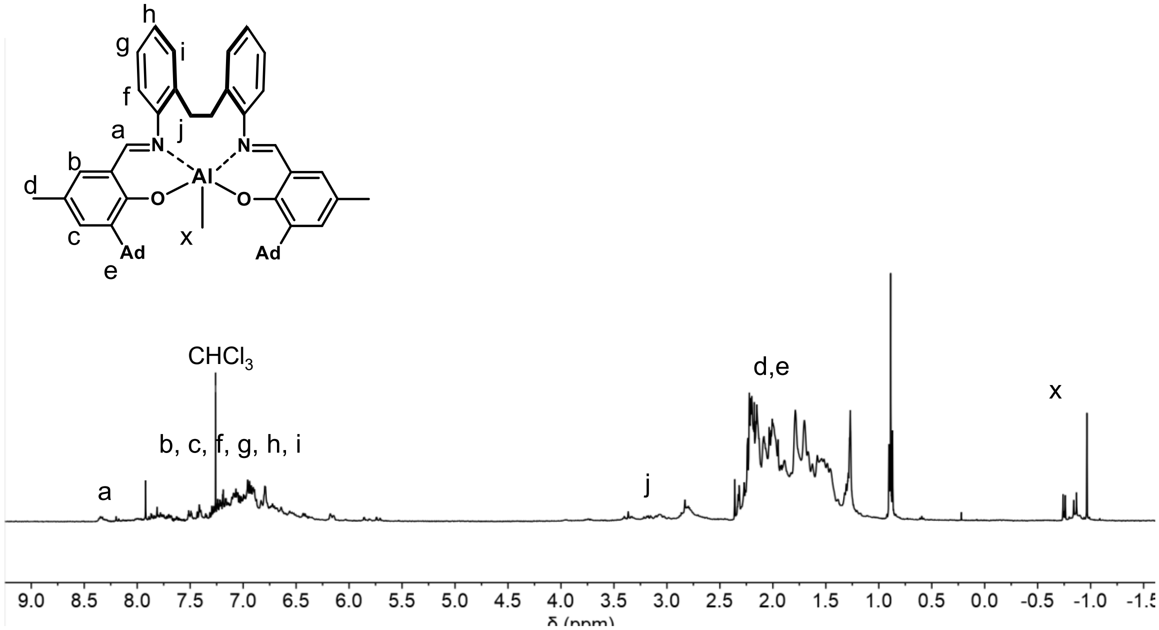

Supplementary Figure 64. ^{1}H NMR (400 MHz, $CDCl_3$) spectrum of ($A_8C_9B_1$)AlMe. We note that the Al complex had low solubility in $CDCl_3$. We also tested toluene-d_8 and THF-d_8, and none of them showed improved solution of the NMR spectrum. ^{1}H NMR showed the aggregation status of the complex in the solution. ESI-MS confirmed the complex. Note the *rac*-LA conversion for the ROP using this complex was only 35.3%

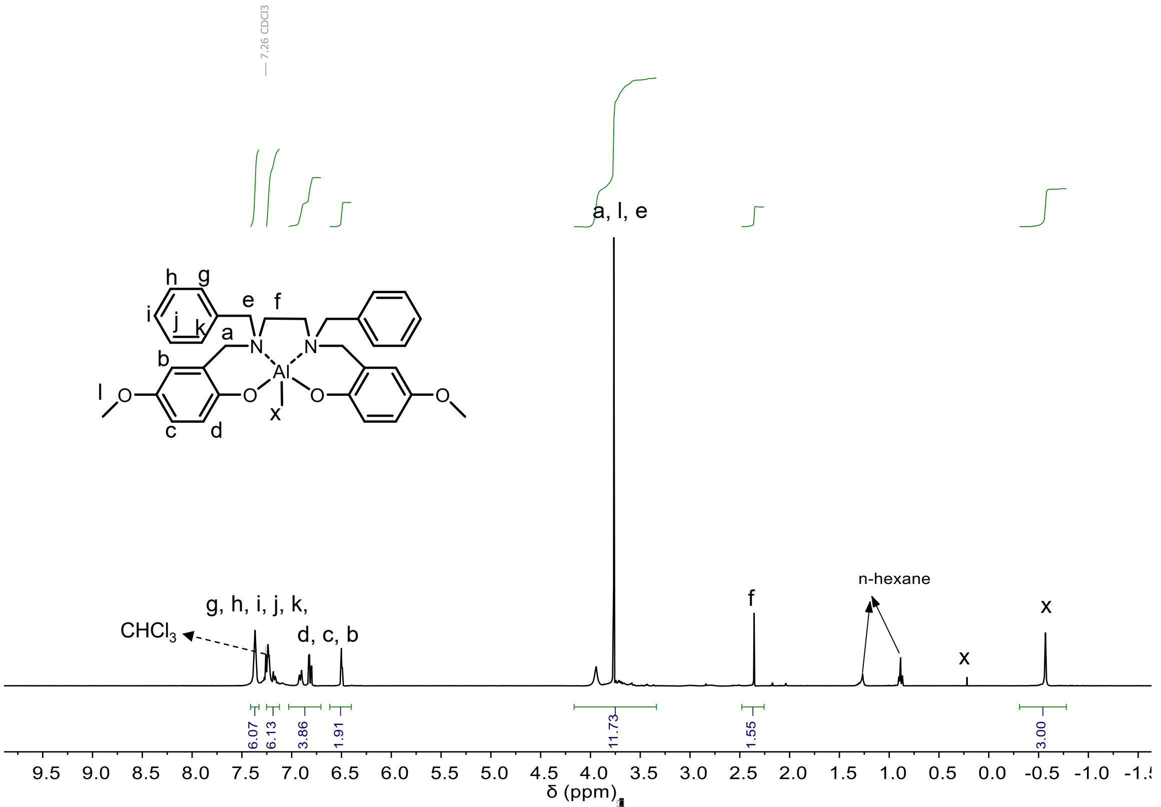

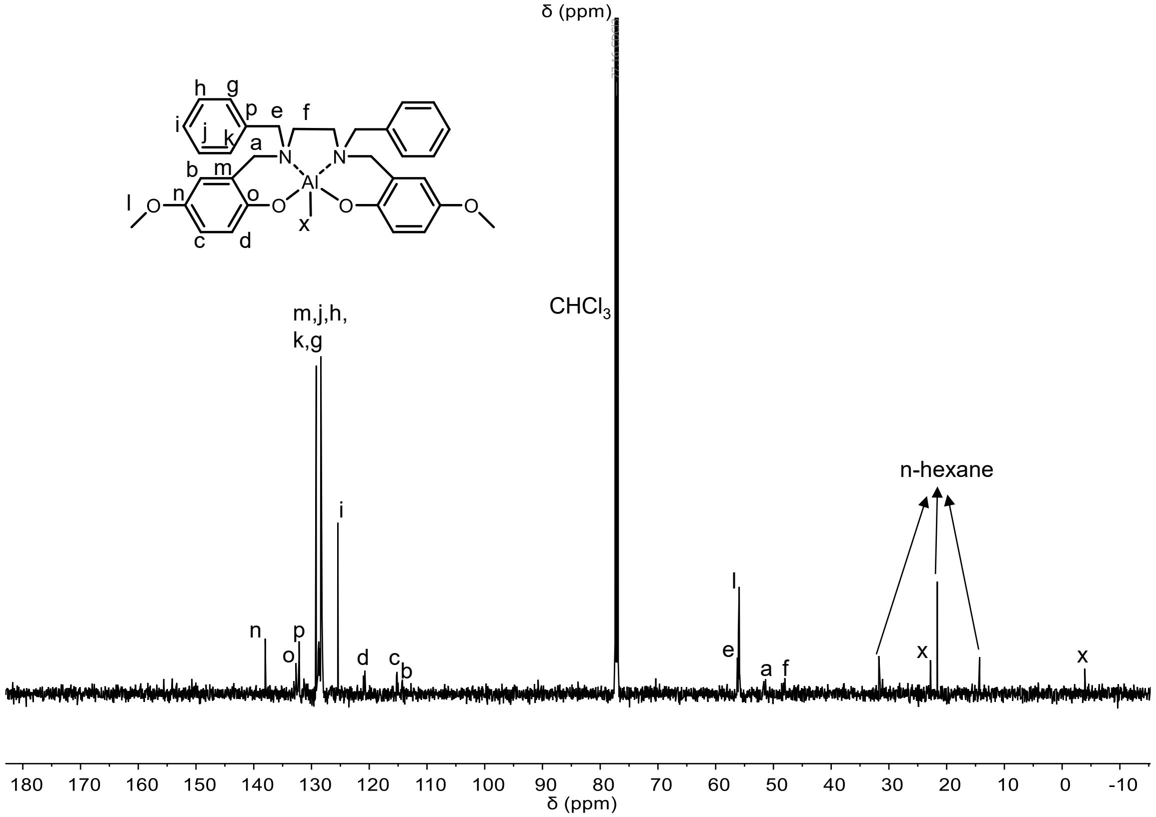

Supplementary Figure 65. ^{1}H NMR (400 MHz, $CDCl_3$) and ^{13}C NMR (400 MHz, $CDCl_3$) spectra of $(A_4C_1B_3)$AlMe.

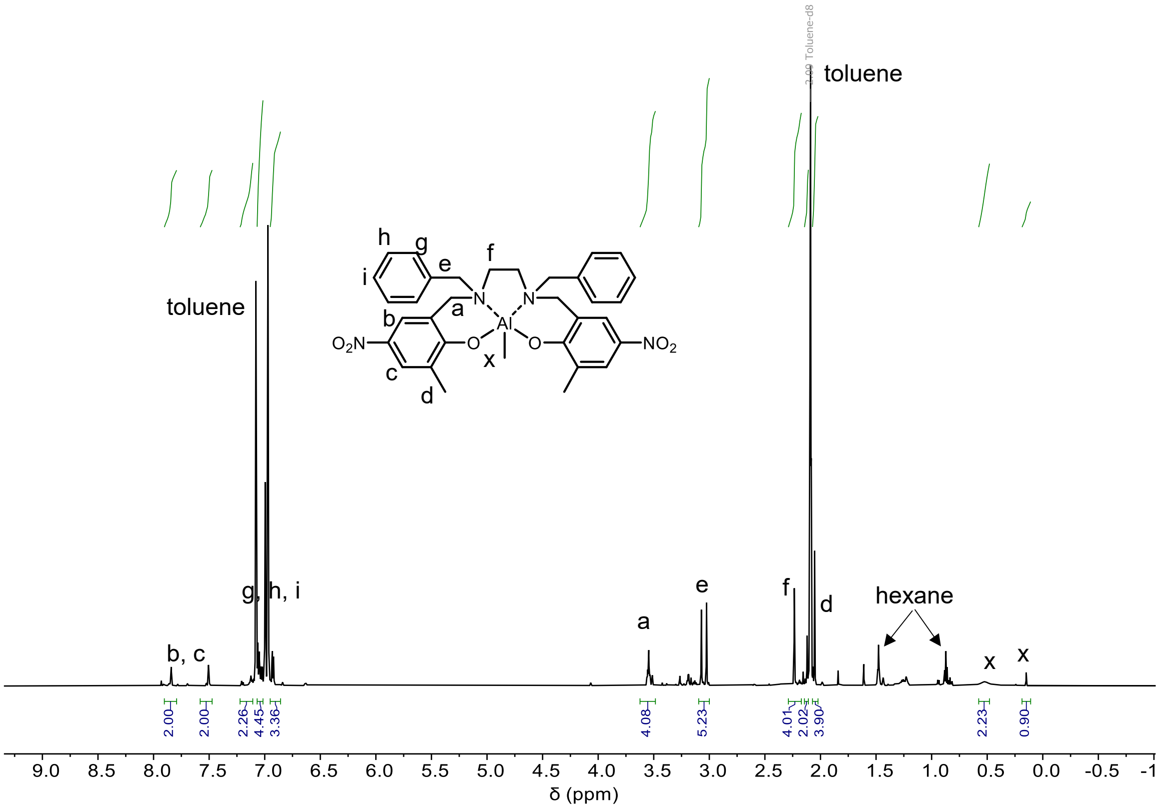

Supplementary Figure 66. ^{1}H NMR (600 MHz, toluene-d_8) spectrum of ($A_5C_1B_3$)AlMe at 85 ºC. We note that the Al complex had low solubility in all solvents, and the solubility improved at the elevated temperature. The complex may have still aggregation even at the elevated temperature. ESI-MS confirmed the complex.

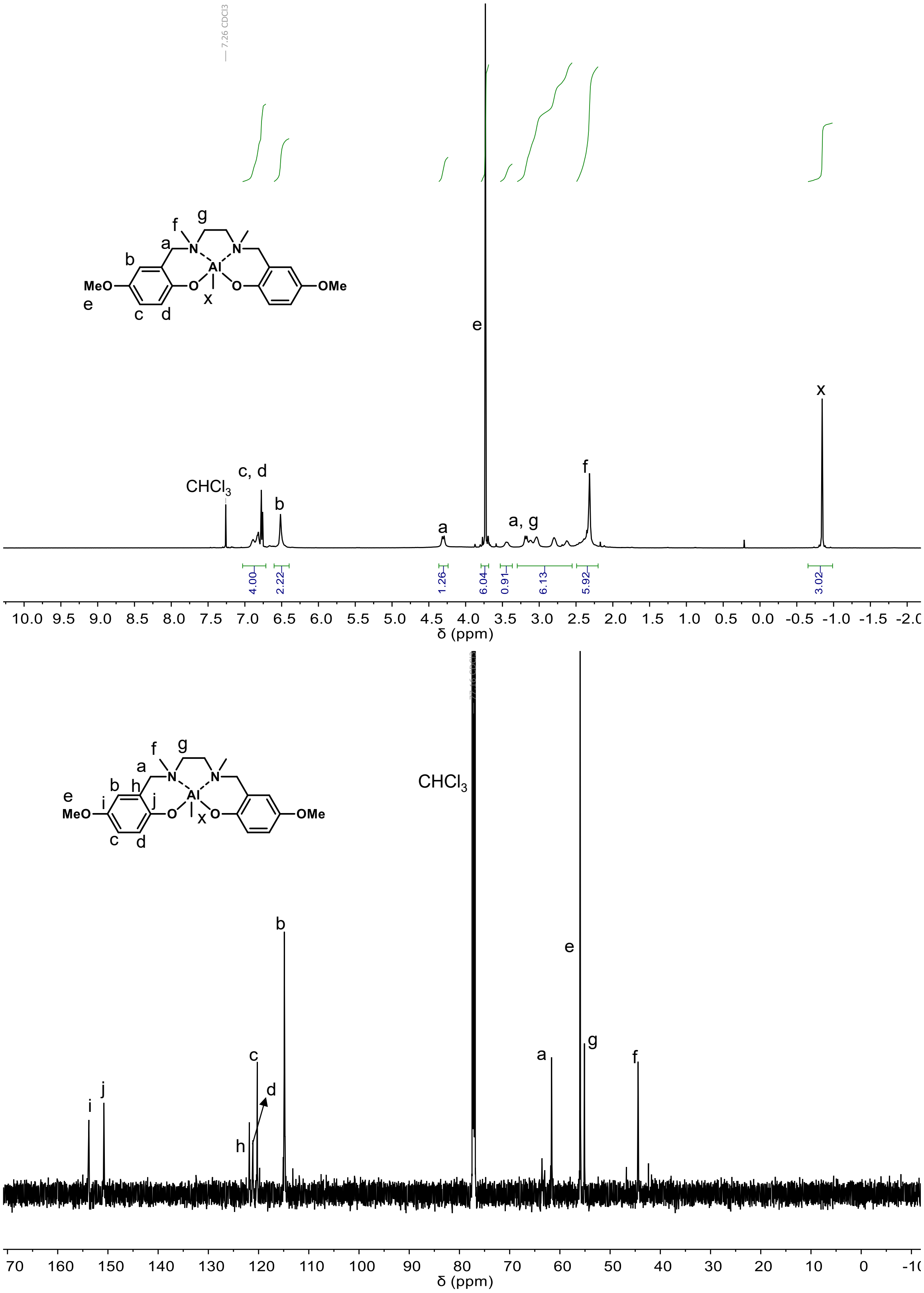

Supplementary Figure 67. ^{1}H NMR (400 MHz, $CDCl_3$) and ^{13}C NMR (125 MHz, $CDCl_3$) spectra of $(A_4C_1B_2)AlMe$.

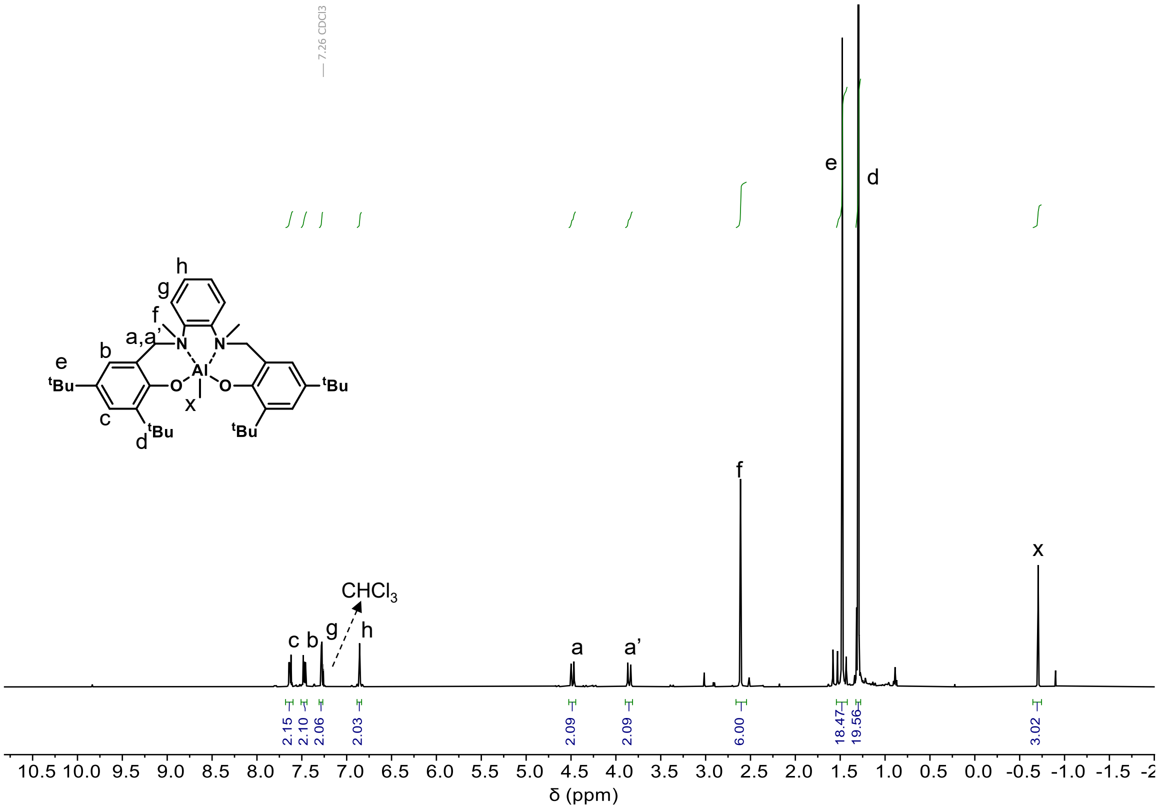

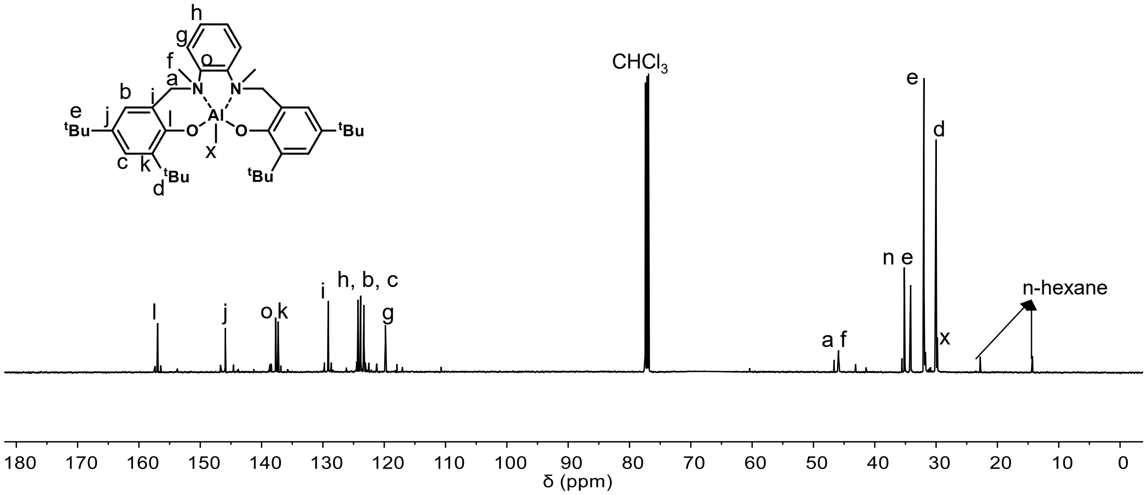

Supplementary Figure 68. ^{1}H NMR (400 MHz, $CDCl_3$) and ^{13}C NMR (125 MHz, $CDCl_3$) spectra of ($A_3C_6B_2$)AlMe.

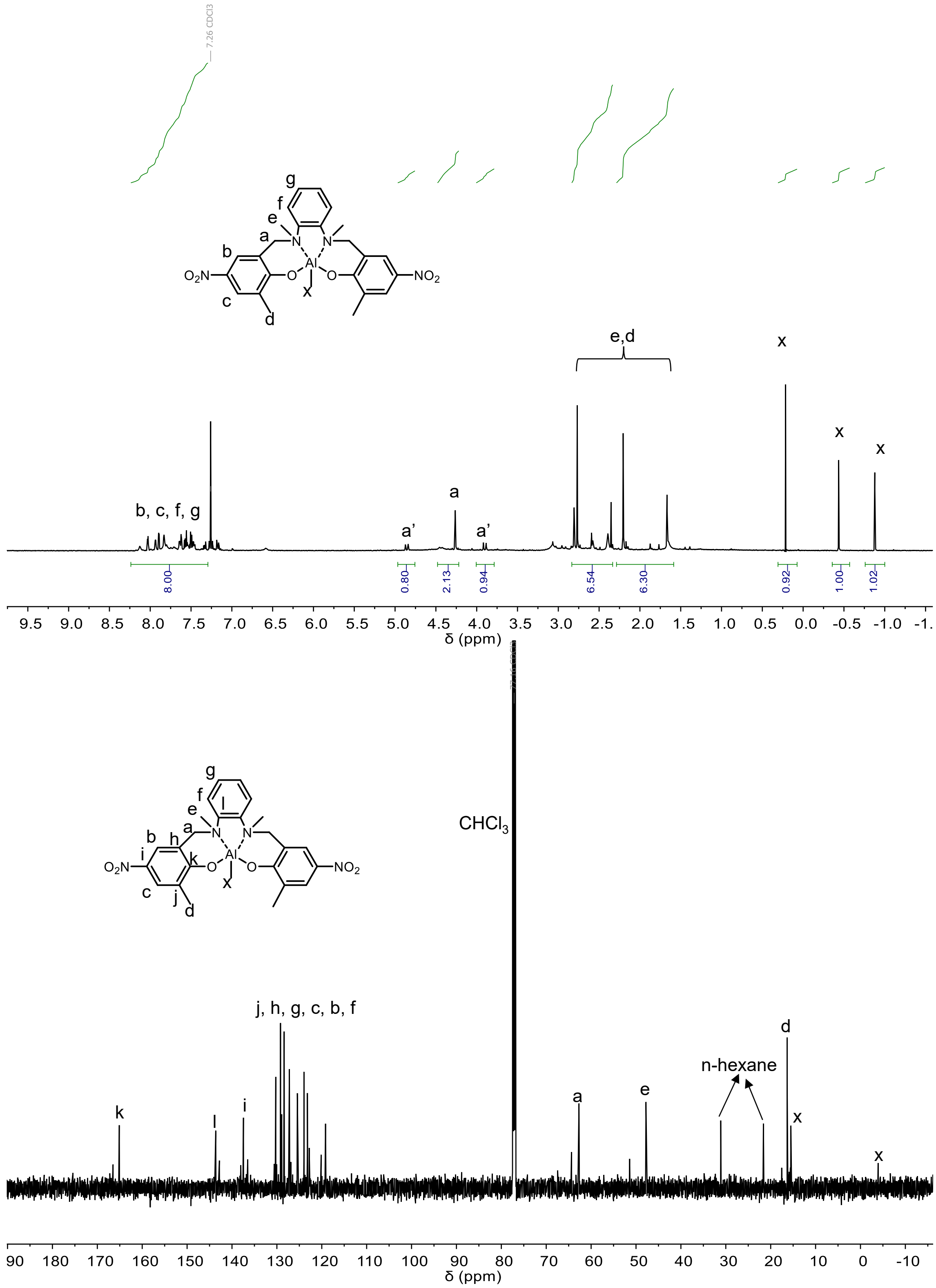

Supplementary Figure 69. ^{1}H (400 MHz, $CDCl_3$) and ^{13}C (400 MHz, $CDCl_3$) NMR spectra of $(A_5C_6B_2)AlMe$. ^{1}H NMR suggested three possible isomers in $CDCl_3$ solution. ESI-MS confirmed the complex.

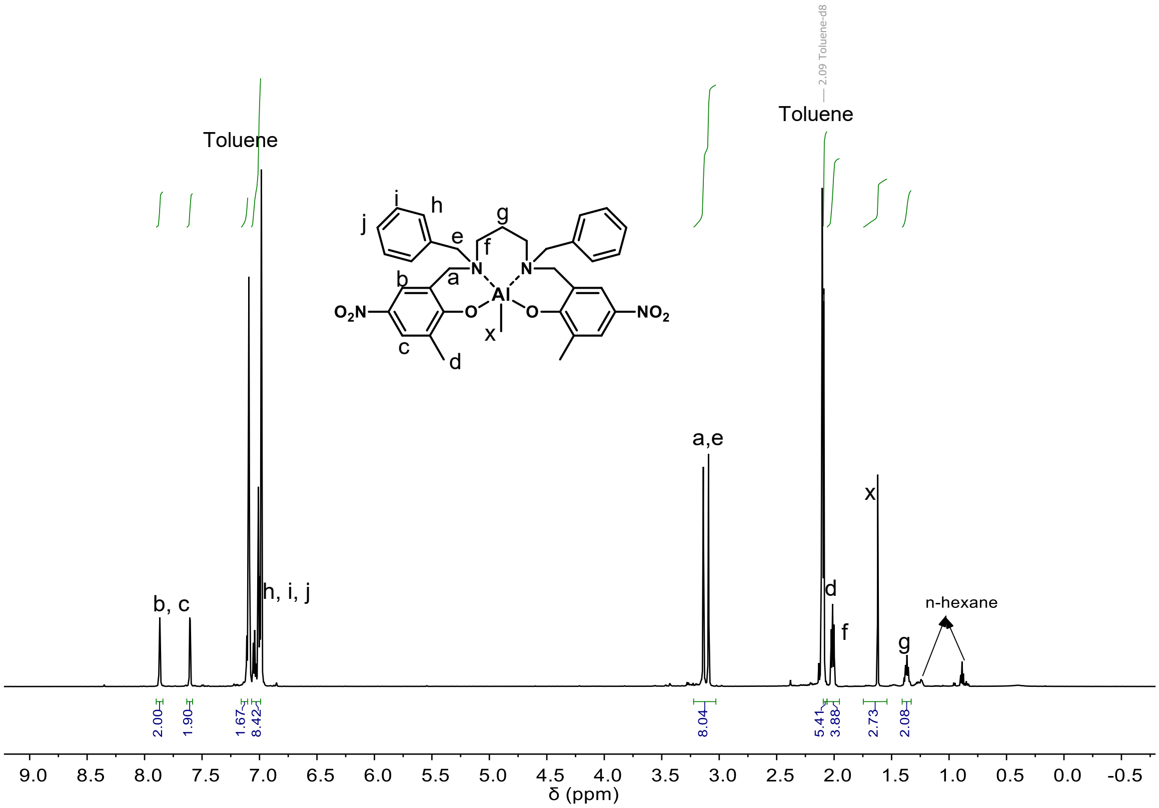

Supplementary Figure 70. ^{1}H NMR (600 MHz, toluene-d_8) spectrum of ($A_5C_2B_3$)AlMe at 85 ºC. We note that the Al complex had low solubility in all solvents. We also tested $CDCl_3$ and THF-d_8 at room temperature condition and none of them showed improved solution of the NMR spectrum. ESI-MS confirmed the complex.

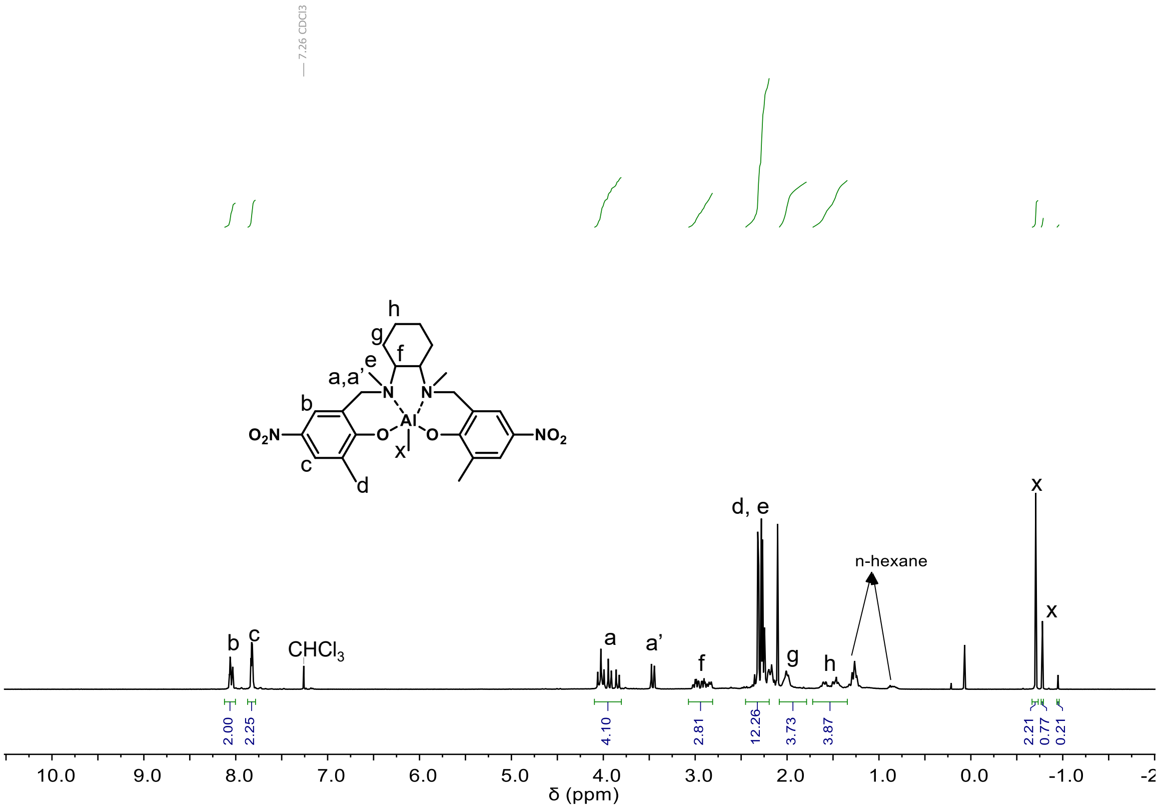

Supplementary Figure 71. ^{1}H NMR (400 MHz, $CDCl_3$) spectrum of $(A_5C_5B_2)AlMe$. We note that the Al complex had low solubility in all solvents. We also tested toluene-d_8 and THF-d_8 at room temperature condition and none of them showed improved solution of the NMR spectrum. ^{1}H NMR suggested the isomers in the $CDCl_3$ solution (because using *rac*-C_5 in the ligand). ESI-MS confirmed the existence of the complex.

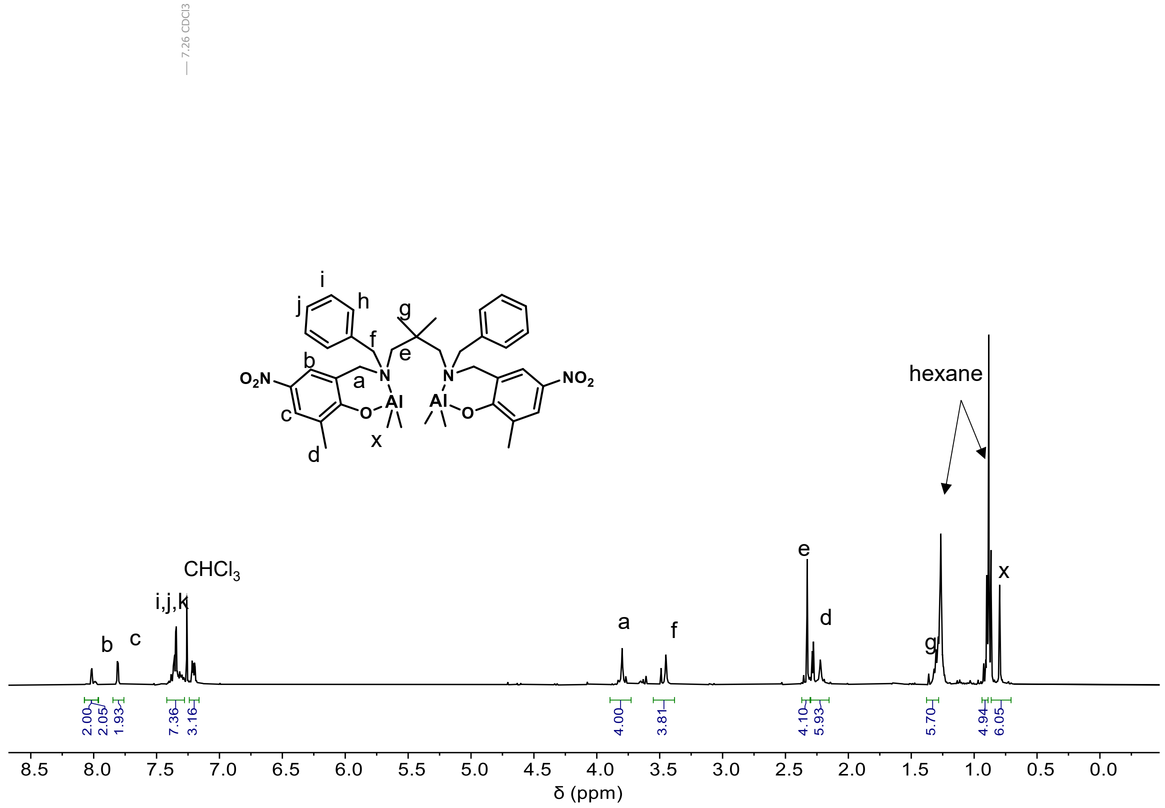

Supplementary Figure 72. ^{1}H NMR (400 MHz, $CDCl_3$) spectrum of $(A_5C_3B_3)Al_2C_4H_{12}$. We note that the Al complex had low solubility in all solvents. We also tested toluene-d_8 and THF-d_8 at room temperature condition and none of them showed improved solution of the NMR spectrum. ^{1}H NMR suggested aggregations in the $CDCl_3$ solution. ESI-MS confirmed the complex having two Al centers. Note the *rac*-LA conversion for the ROP using this complex was only 40.3%

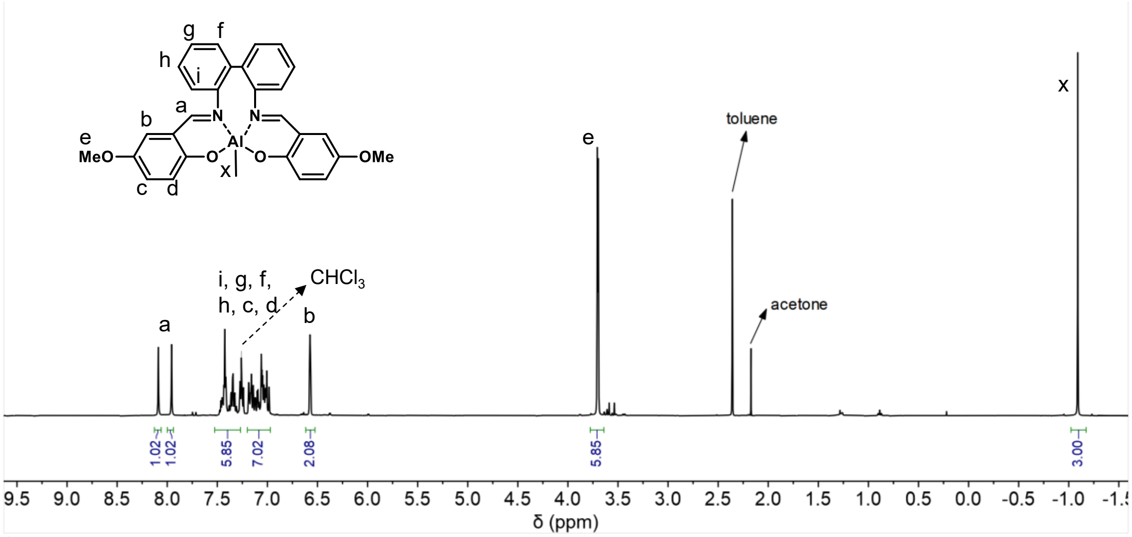

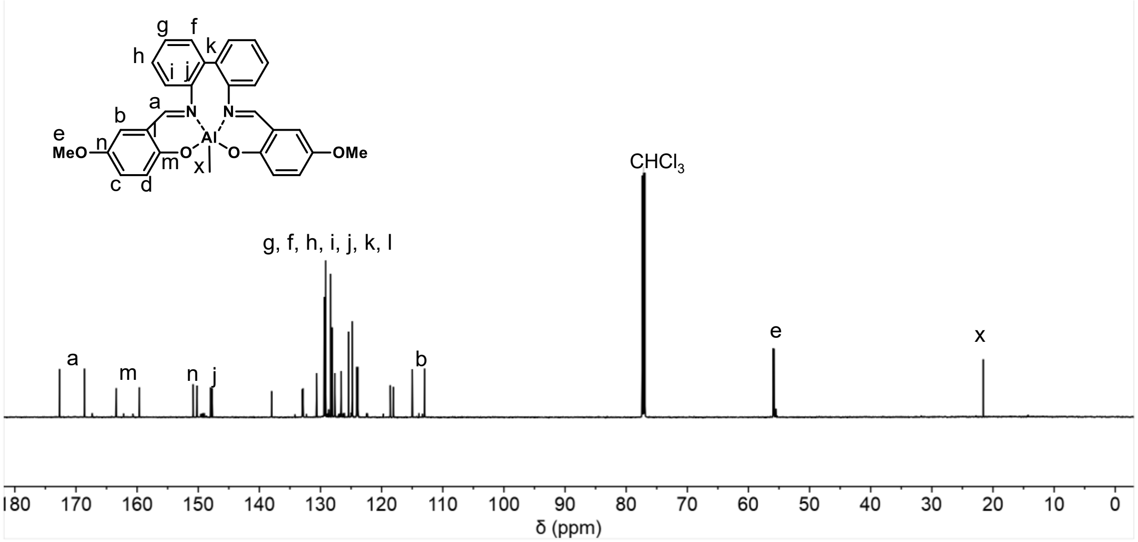

Supplementary Figure 73. ^{1}H NMR (400 MHz, $CDCl_3$) and ^{13}C NMR (125 MHz, $CDCl_3$) spectra of $(A_4C_8B_1)AlMe$. The ^{13}C NMR spectrum suggested possible *R*, *S* configurations (two sets of aromatic C peaks) in the complex (like the binaphthyl-Al complex).

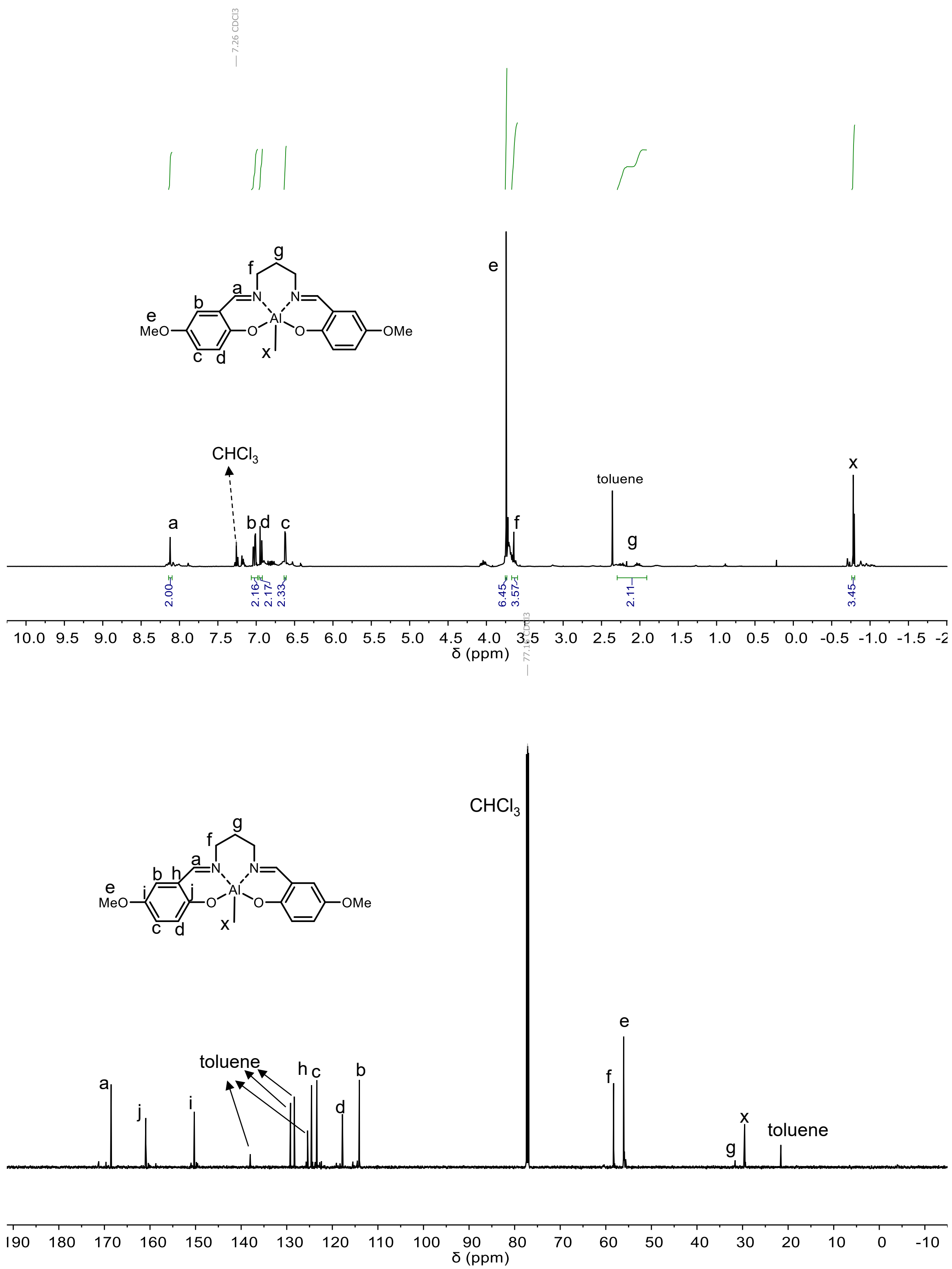

Supplementary Figure 74. ^{1}H NMR (400 MHz, $CDCl_3$) and ^{13}C NMR (125 MHz, $CDCl_3$) spectra of $(A_4C_2B_1)$AlMe.

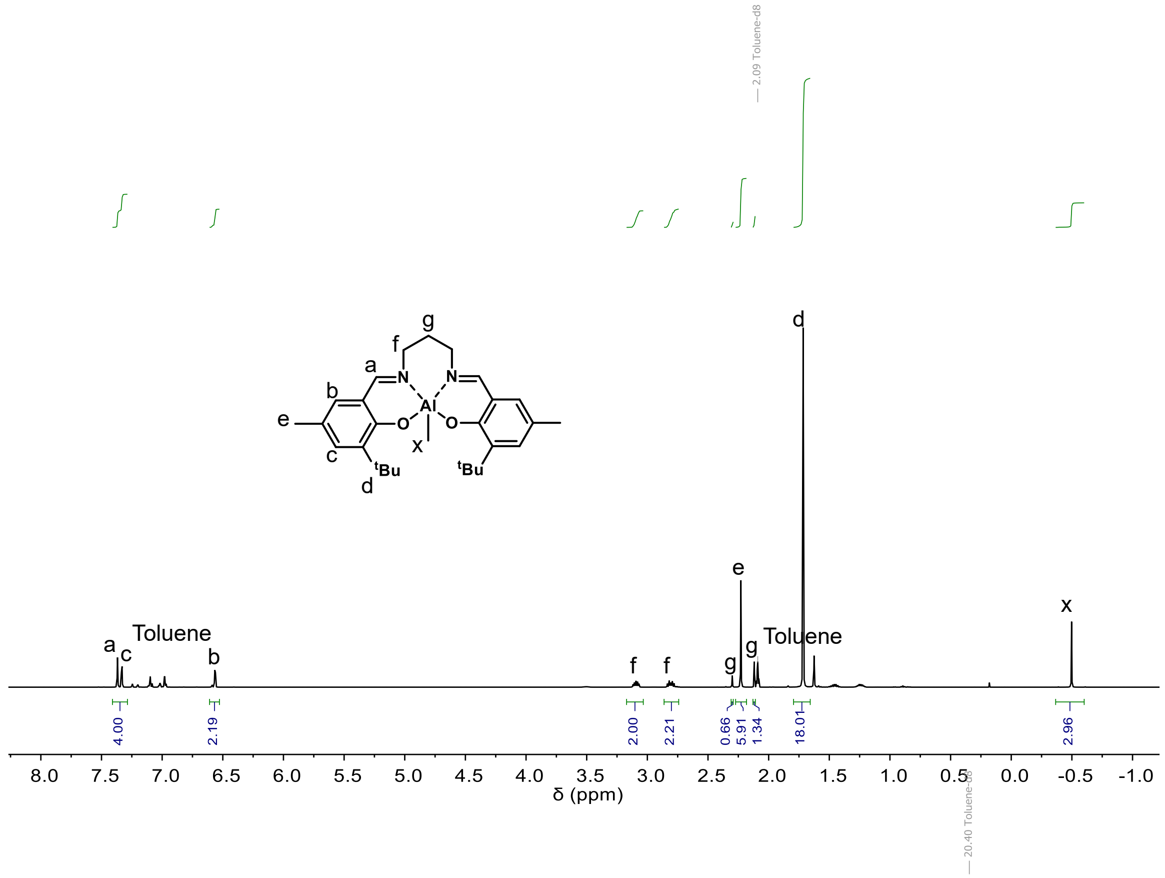

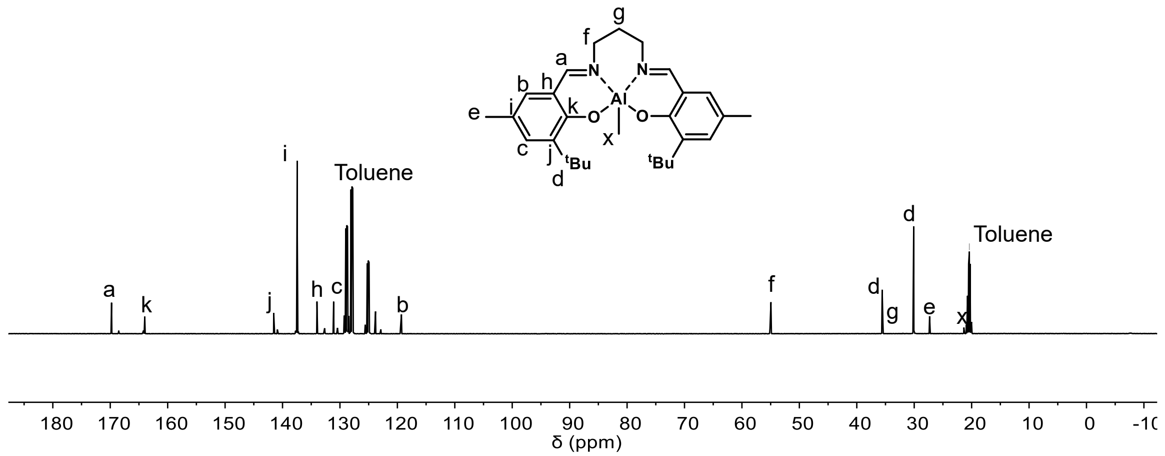

Supplementary Figure 75. ^{1}H NMR (400 MHz, toluene-d_8) and ^{13}C NMR (125 MHz, toluene-d_8) spectra of $(A_{11}C_2B_1)$AlMe.

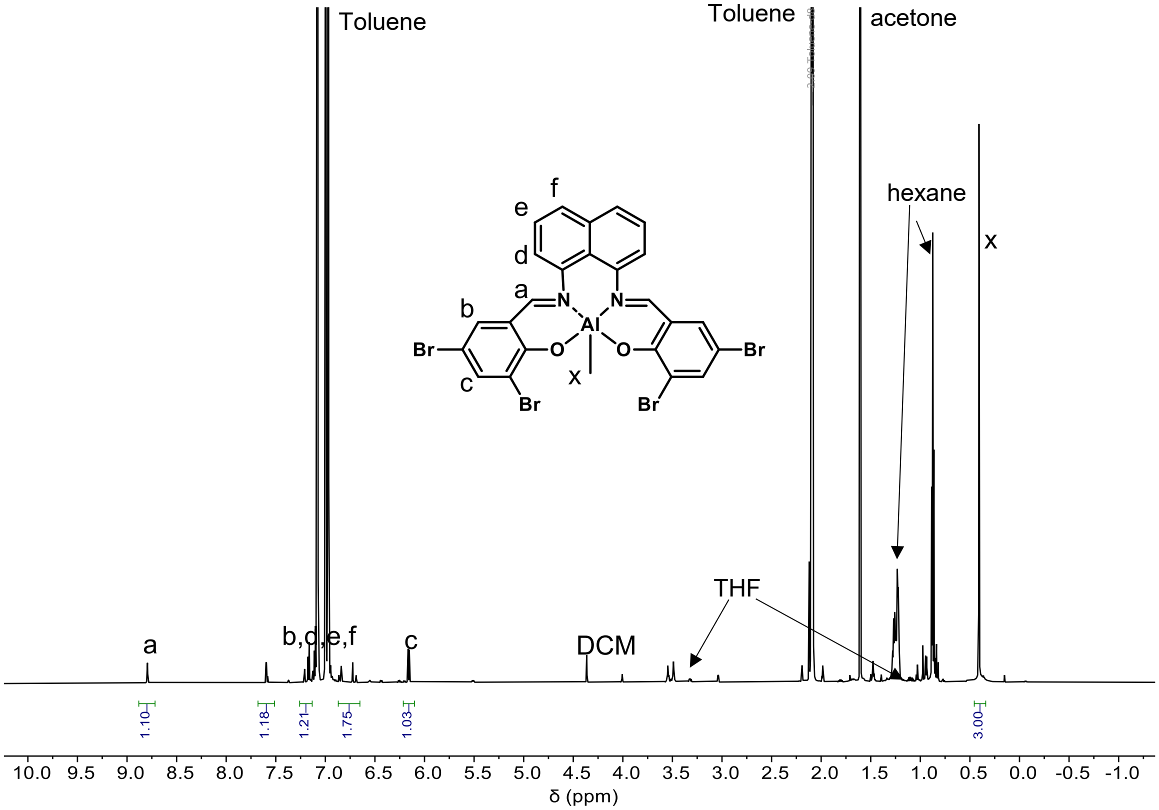

Supplementary Figure 76. ^{1}H NMR (600 MHz, toluene-d_8) spectrum of $(A_{16}C_7B_1)$AlMe at 85 °C. We note that the Al complex had low solubility in all solvents (THF, toluene, chloroform) at room temperature. ESI-MS confirmed the complex.

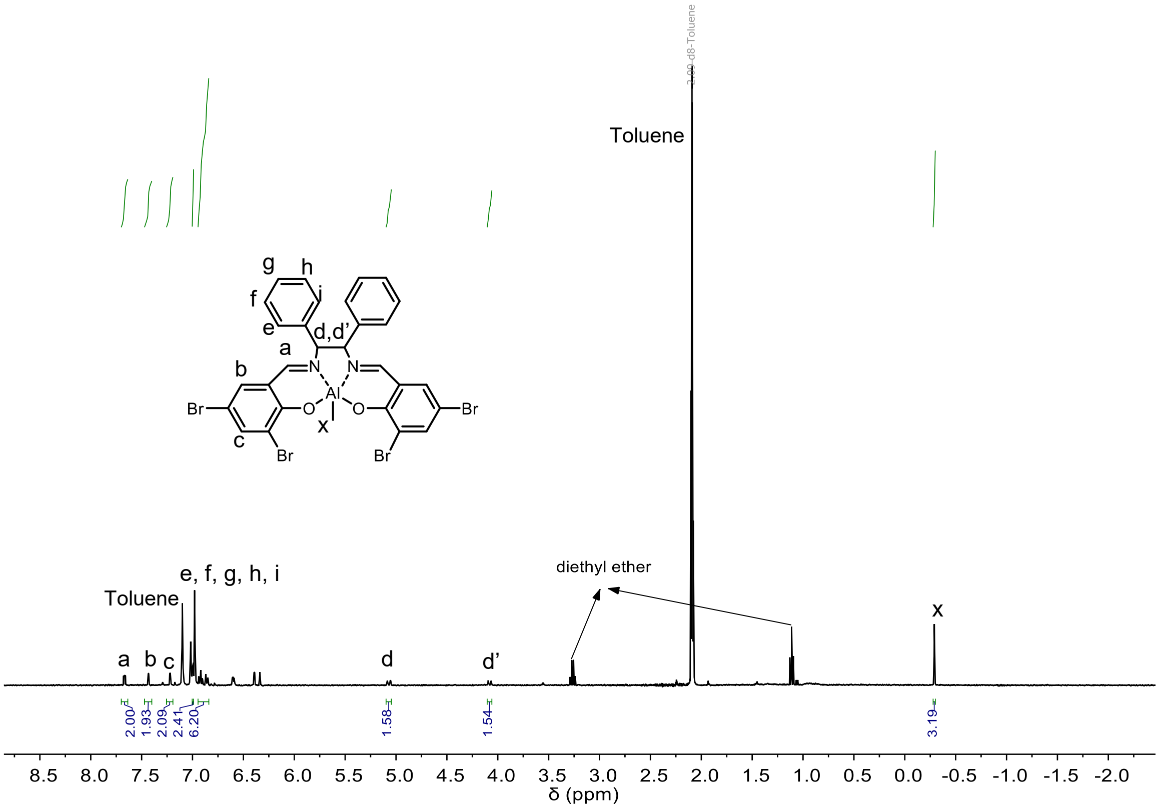

Supplementary Figure 77. ^{1}H NMR (400 MHz, toluene-d_8) spectrum of $(A_{16}C_{11}B_1)$AlMe. We note that the Al complex had low solubility in all solvents. We also tested $CDCl_3$ and THF-d_8 at room temperature condition and none of them showed improved solution of the NMR spectrum. ESI-MS confirmed the complex.

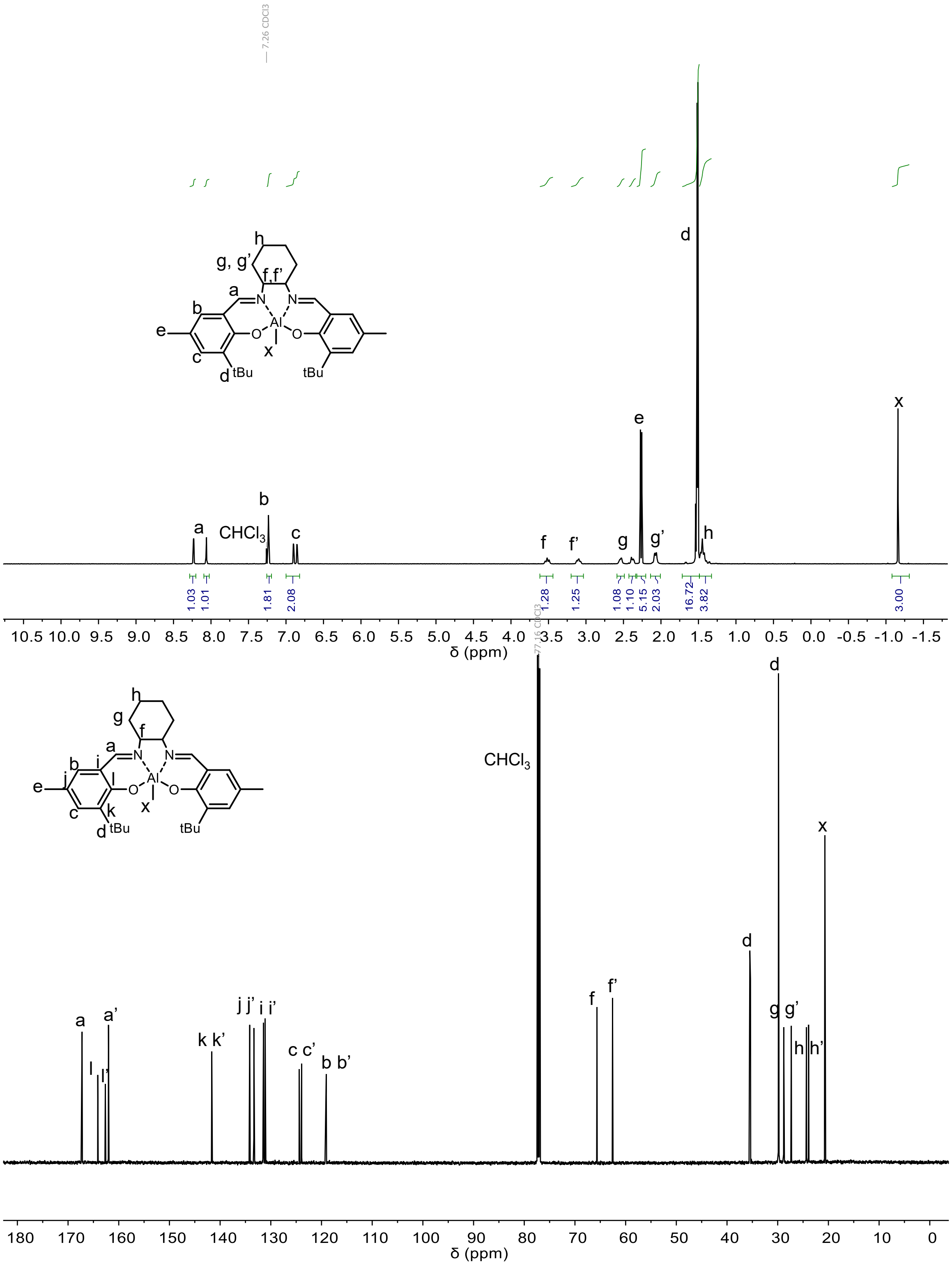

Supplementary Figure 78. ^{1}H NMR (400 MHz, $CDCl_3$) and ^{13}C NMR (125 MHz, $CDCl_3$) spectra of $(A_{11}C_5B_1)AlMe$. The ^{13}C NMR spectrum suggested possible two configurations (two sets of aromatic C peaks) in the complex (as we used the *rac*-C_5 ligand).

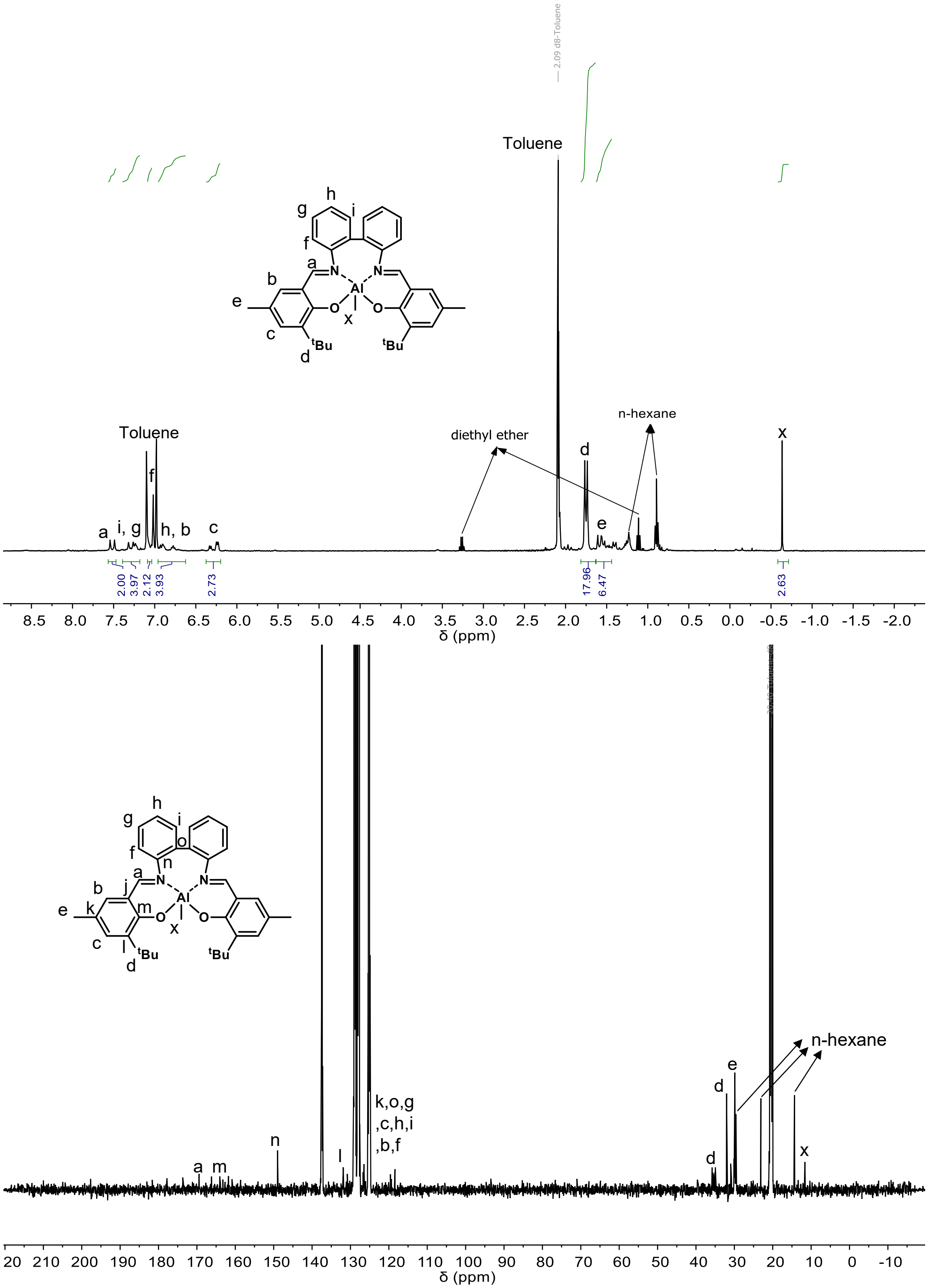

Supplementary Figure 79. ^{1}H NMR (400 MHz, toluene-d_8) spectrum of $(A_{11}C_8B_1)AlMe$. ESI-MS confirmed the complex.

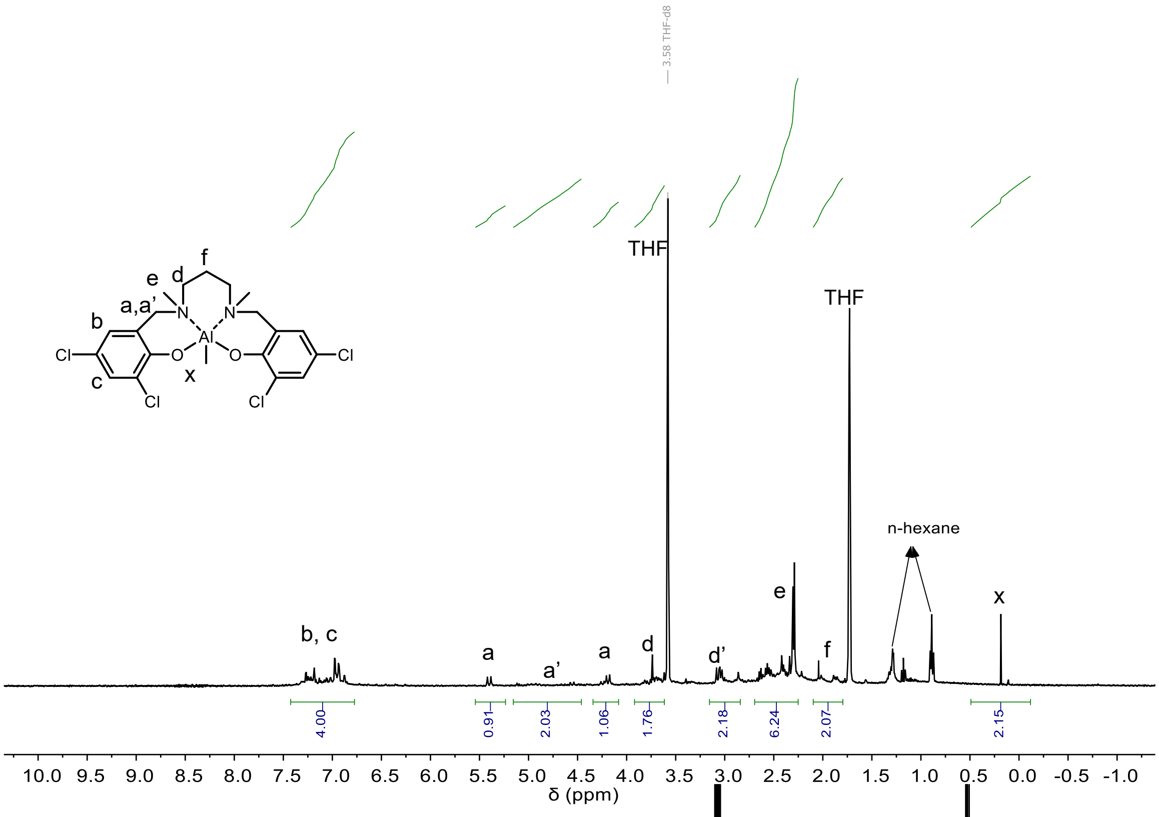

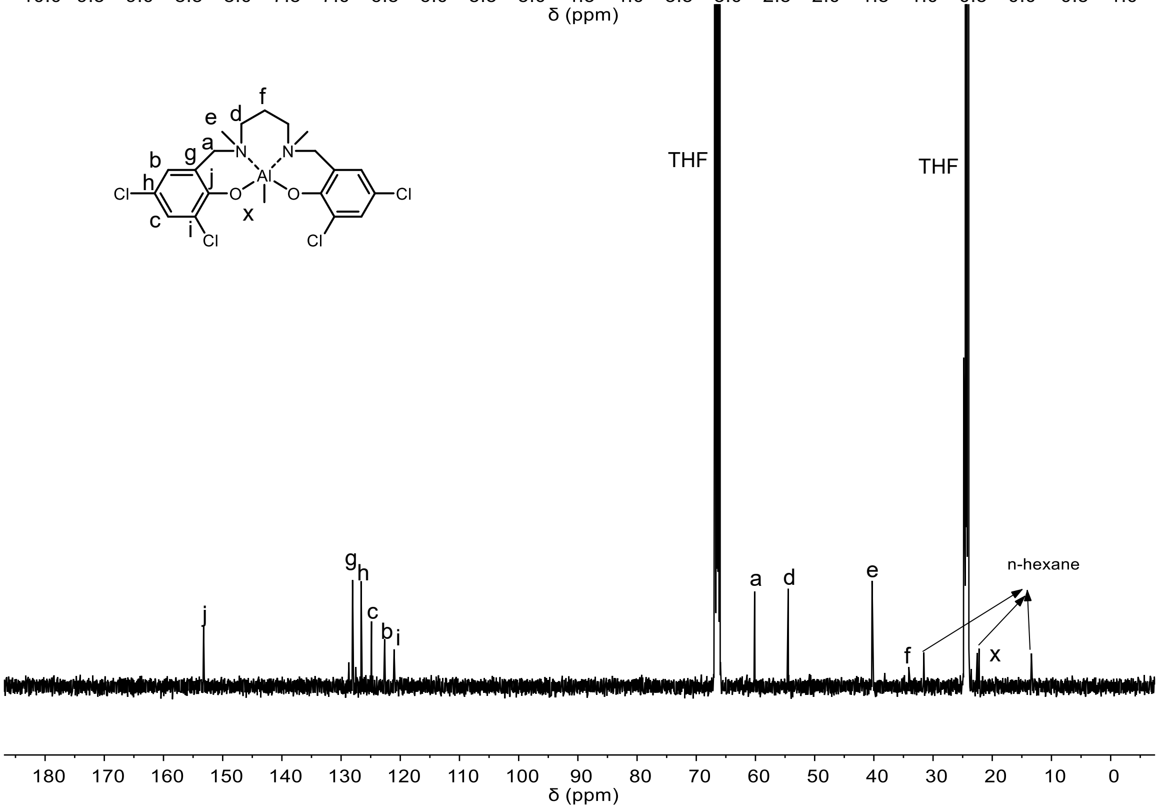

Supplementary Figure 80. ^{1}H NMR (400 MHz, THF-d_8) and ^{13}C NMR (125 MHz, THF-d_8) spectra of $(A_6C_2B_2)AlMe$. The ^{1}H NMR spectra suggested possible aggregations of the complex in the solution.

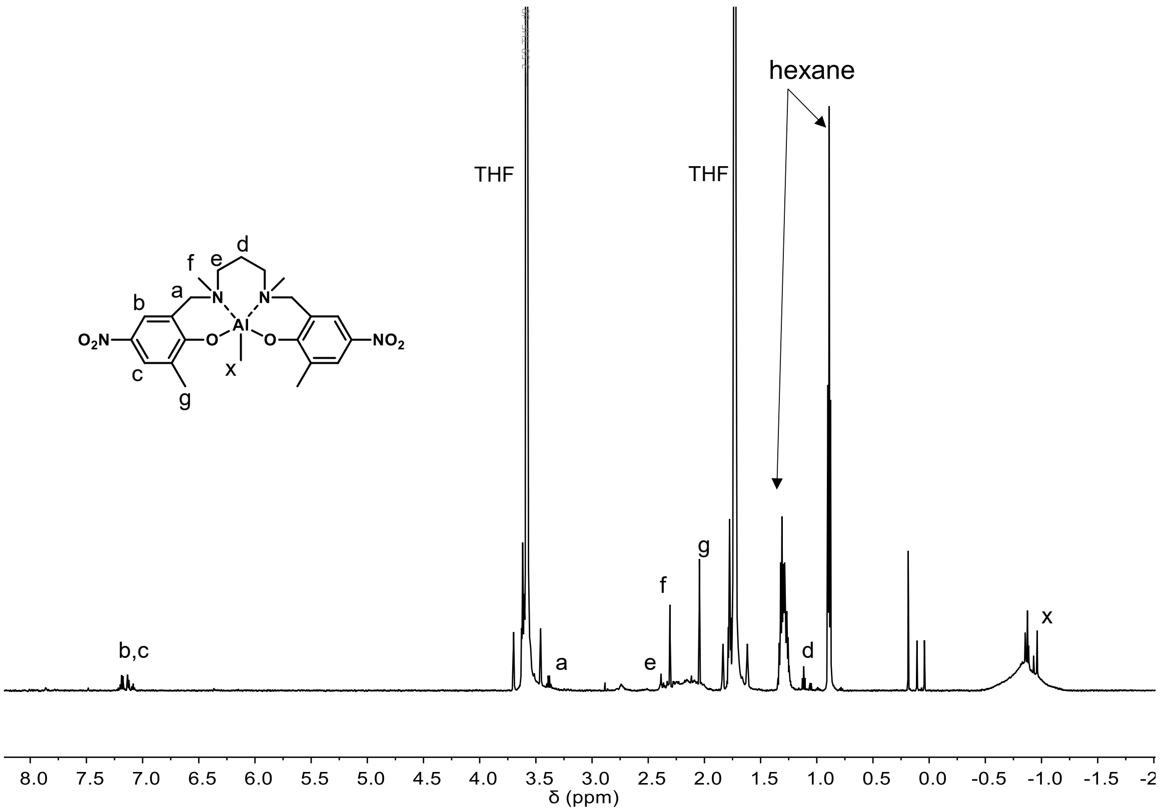

Supplementary Figure 81. ^{1}H NMR (400 MHz, THF-d_8) spectrum of ($A_5C_2B_2$)AlMe. We note that the Al complex had low solubility in all solvents. We also tested $CDCl_3$ and toluene-d_8 at room temperature condition and none of them showed improved solution of the NMR spectrum. ESI-MS confirmed the existence of the complex. Note that PLA produced by this Al complex only had a P_r of 0.31.

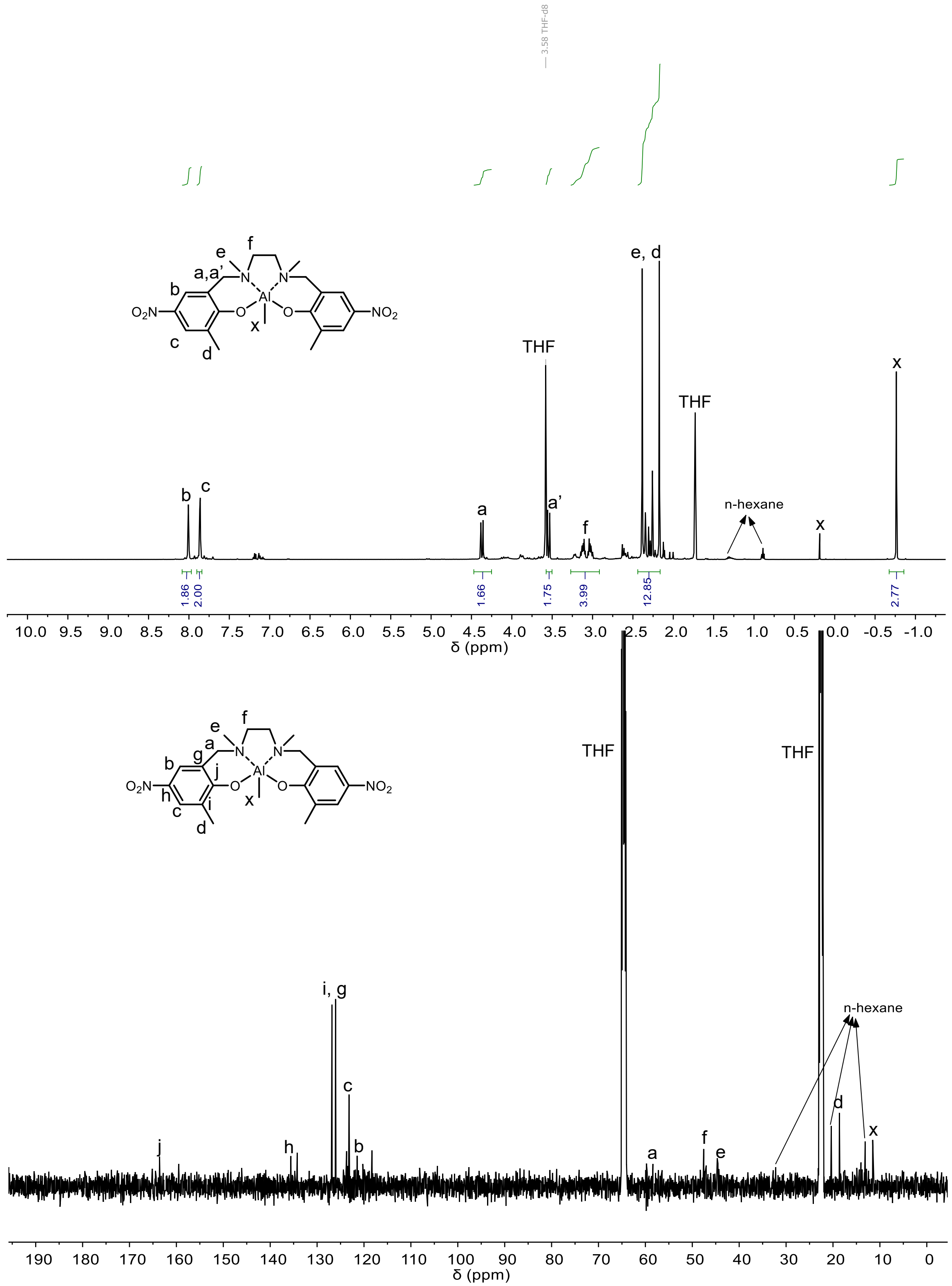

Supplementary Figure 82. ^{1}H NMR (400 MHz, THF-d_8) and ^{13}C NMR (125 MHz, THF-d_8) spectra of $(A_5C_1B_2)AlMe$.

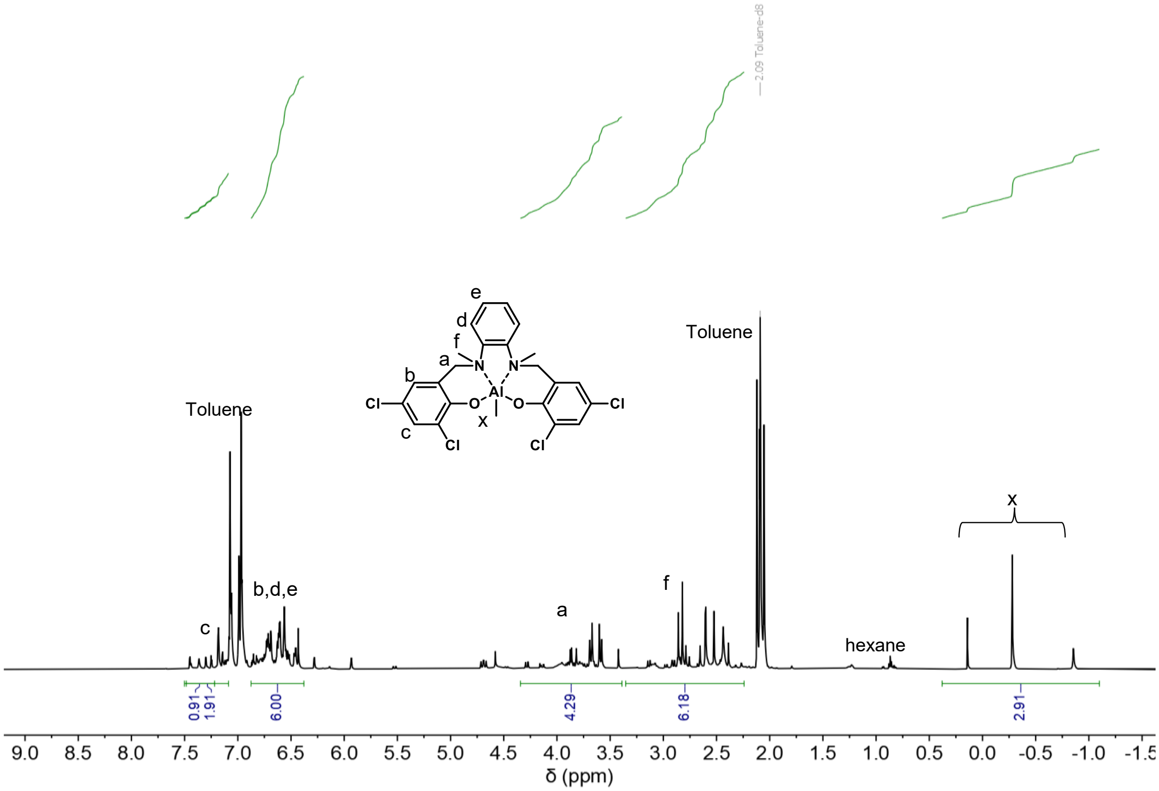

Supplementary Figure 83. ^{1}H NMR (600 MHz, toluene-d_8) spectrum of ($A_6C_6B_2$)AlMe at 85 ºC. We note that the Al complex had low solubility in all solvents. We also tested $CDCl_3$ and THF-d_8 at room temperature condition and none of them showed improved solution of the NMR spectrum. ^{1}H NMR suggested isomers in the toluene solution. ESI-MS confirmed the complex.

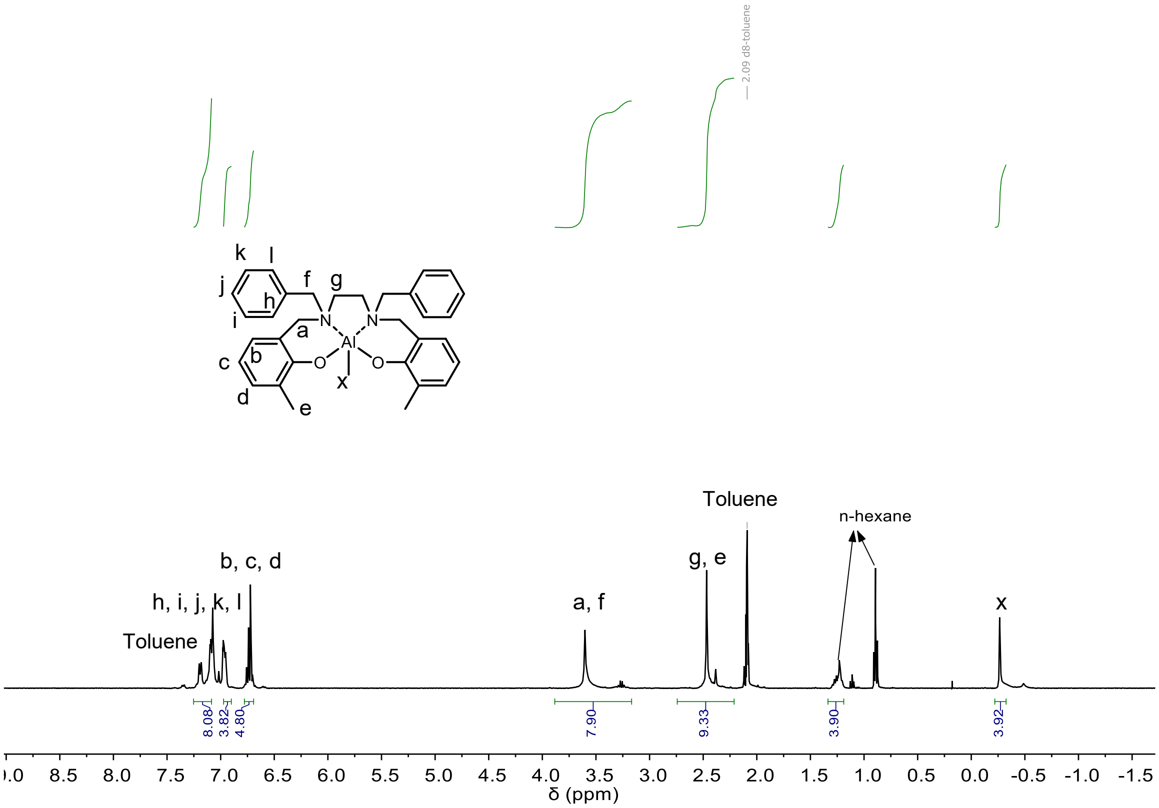

Supplementary Figure 84. ^{1}H NMR (400 MHz, toluene-d_8) spectrum of ($A_2C_1B_3$)AlMe. We note that the Al complex had low solubility in all solvents. We also tested $CDCl_3$ and THF-d_8 at room temperature condition and none of them showed improved solution of the NMR spectrum. ESI-MS confirmed the complex.

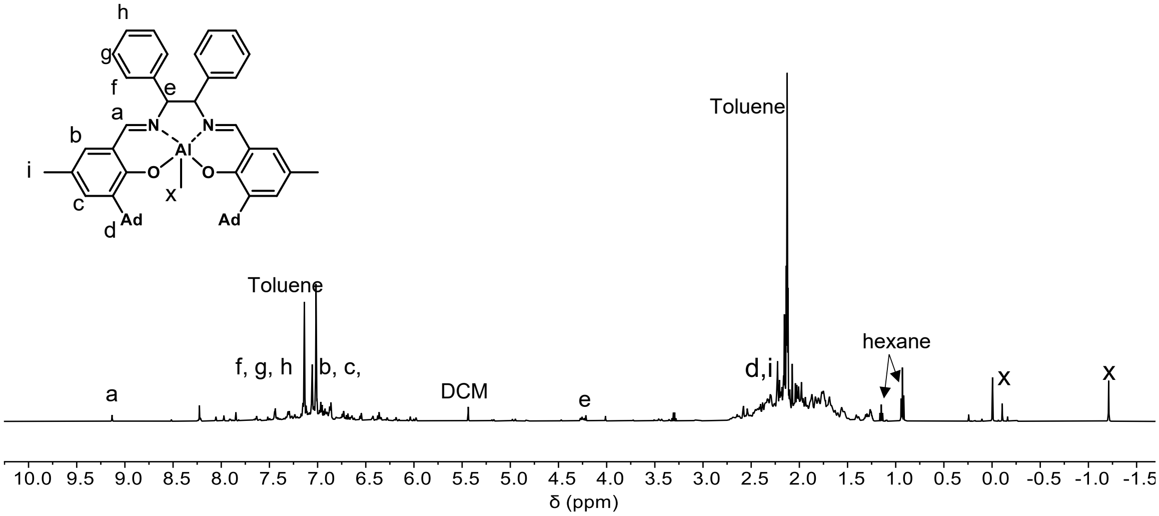

Supplementary Figure 85. ^{1}H NMR (400 MHz, toluene-d_8) spectrum of ($A_8C_{11}B_1$)AlMe. We note that the Al complex had low solubility in all solvents. The ^{1}H NMR spectrum suggested the complex had isomers as we used *rac*-C_{11}. ESI-MS confirmed the complex. Note the ROP of *rac*-LA mediated by this Al complex had a monomer conversion of 37.5%.

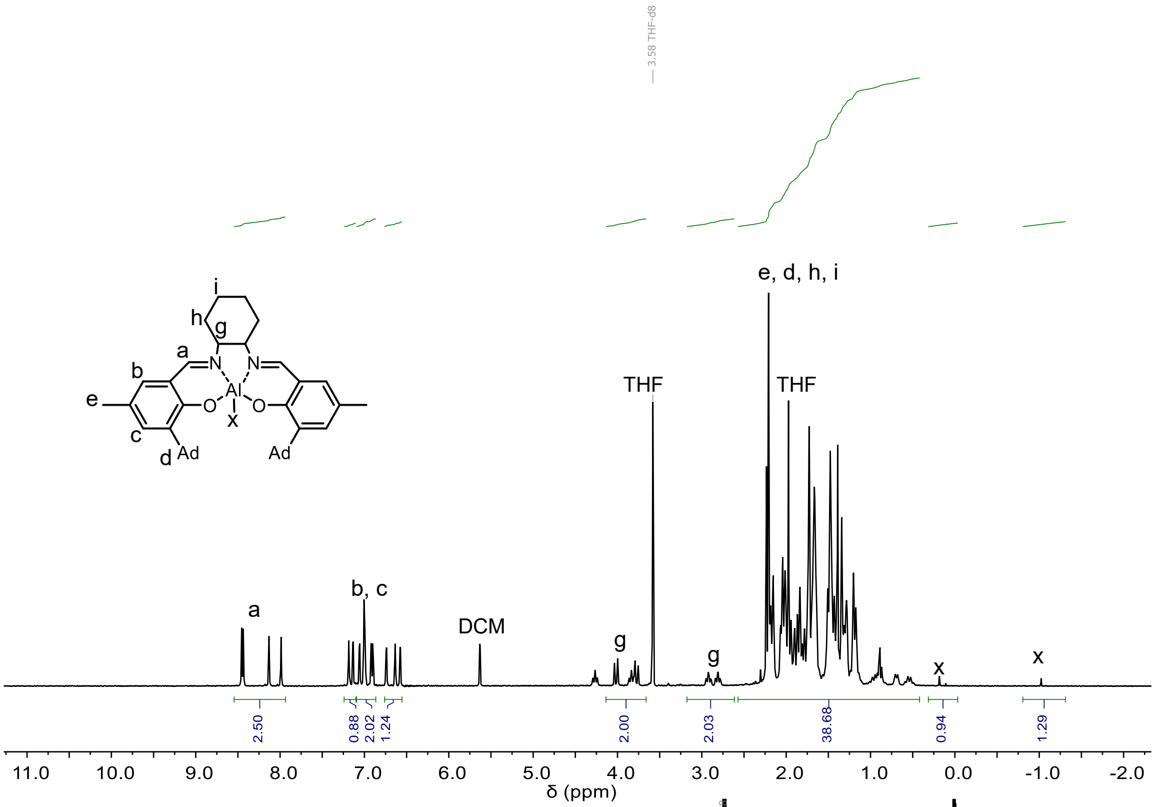

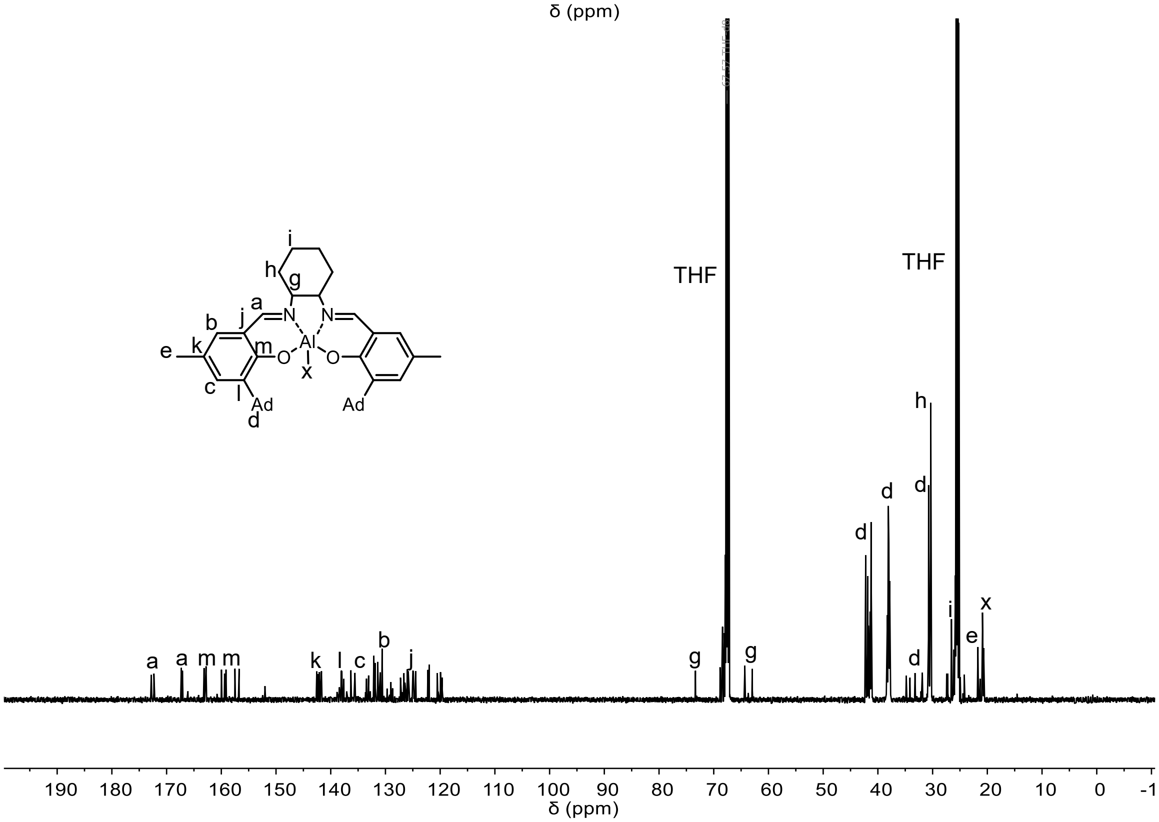

Supplementary Figure 86. ^{1}H NMR (400 MHz, THF-d_8) and ^{13}C NMR (125 MHz, THF-d_8) spectra of ($A_8C_5B_1$)AlMe. The ^{1}H NMR spectrum suggested the complex had isomers as we used *rac*-C_5, as well as the aggregation states in THF. ESI-MS confirmed the complex.

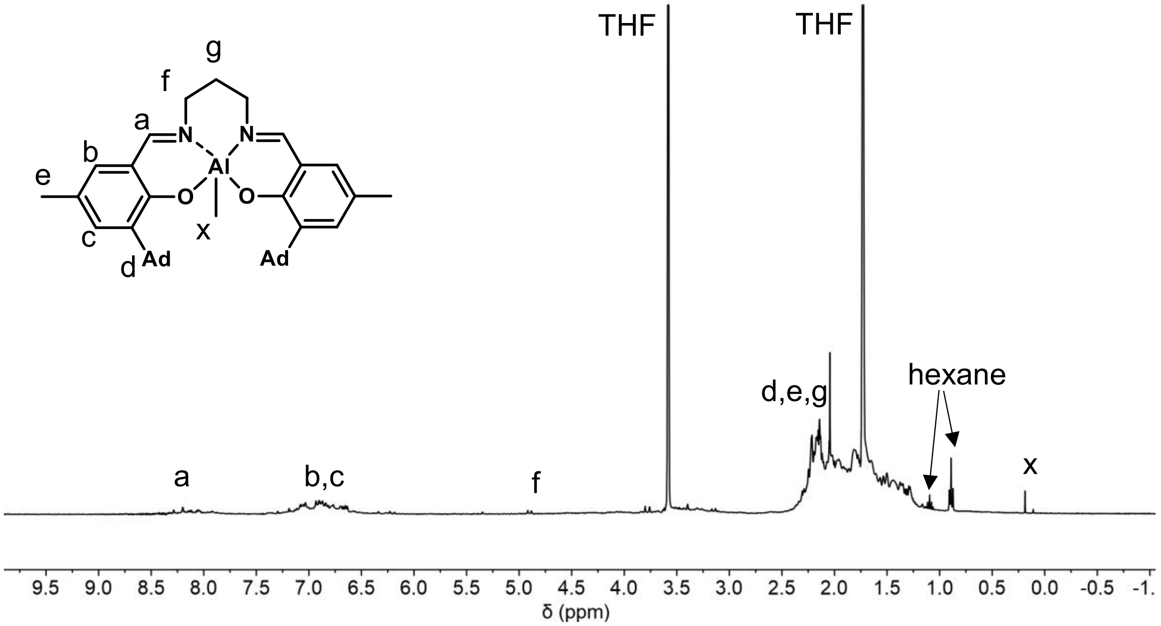

Supplementary Figure 87. ^{1}H NMR (400 MHz, THF-d_8) spectrum of $(A_8C_2B_1)$AlMe. We note that the Al complex had low solubility in all solvents. ESI-MS confirmed the complex. Note the conversion of *rac*-LA was 42.4% for the ROP using this Al complex.

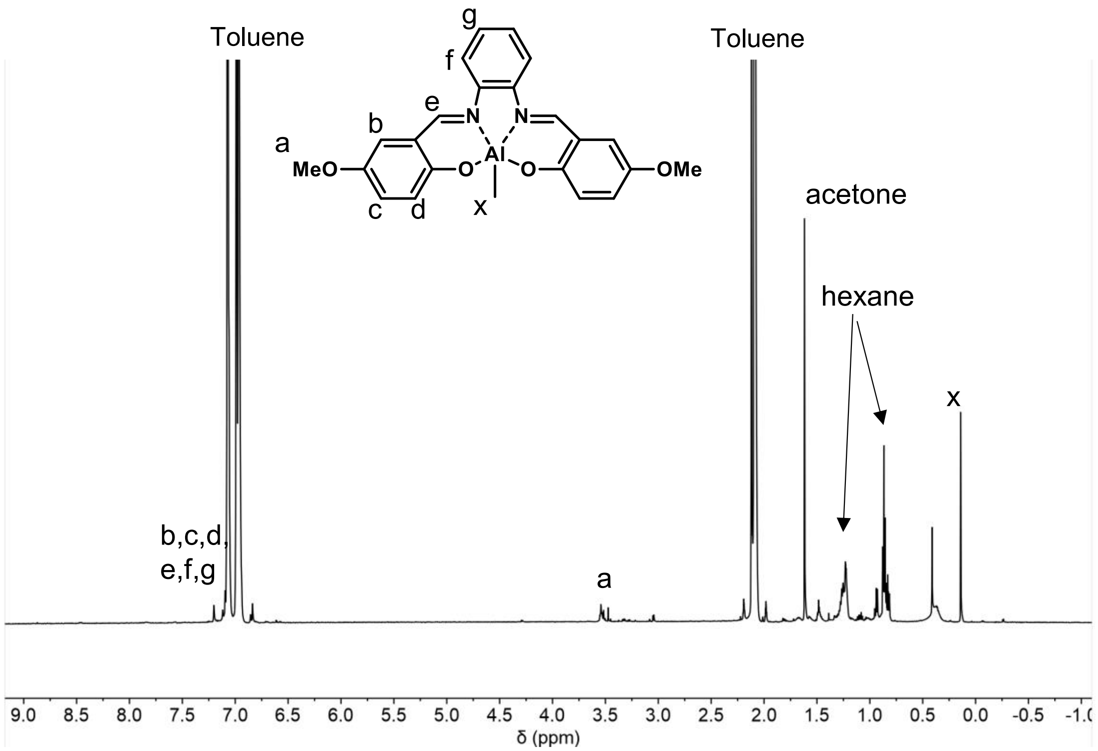

Supplementary Figure 88. ^{1}H NMR (600 MHz, toluene-d_8) spectrum of $(A_4C_1B_1)$AlMe at 85 °C. We note that the Al complex had low solubility in all solvents. ESI-MS confirmed the complex.

Note that ROP of *rac*-LA mediated by this Al complex had a monomer conversion of 50.4% and a P_m value of 0.51.

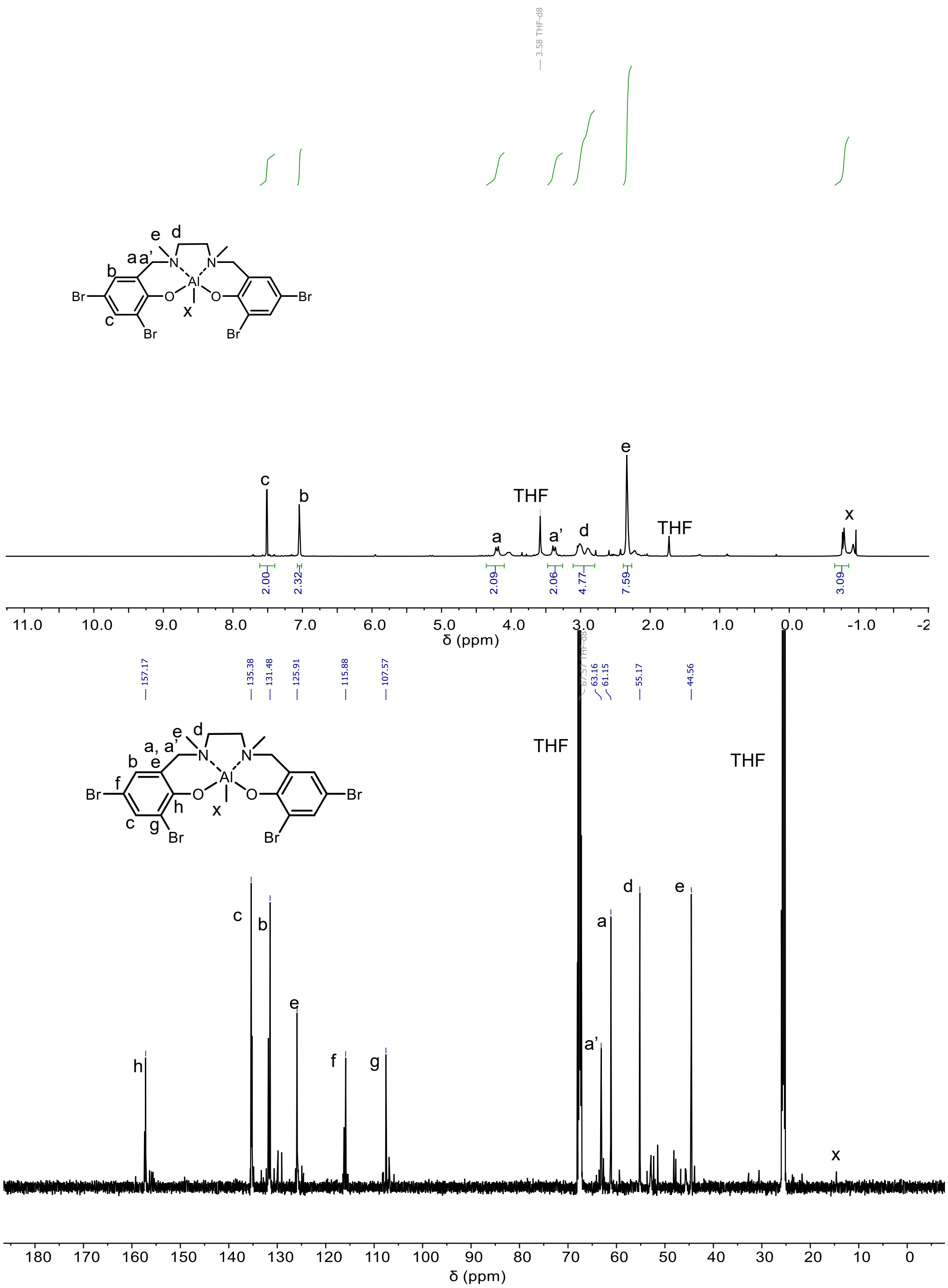

Supplementary Figure 89. ^{1}H NMR (400 MHz, THF-d_8) and ^{13}C NMR (125 MHz, THF-d_8) spectra of ($A_{16}C_1B_2$)AlMe. ESI-MS confirmed the complex.

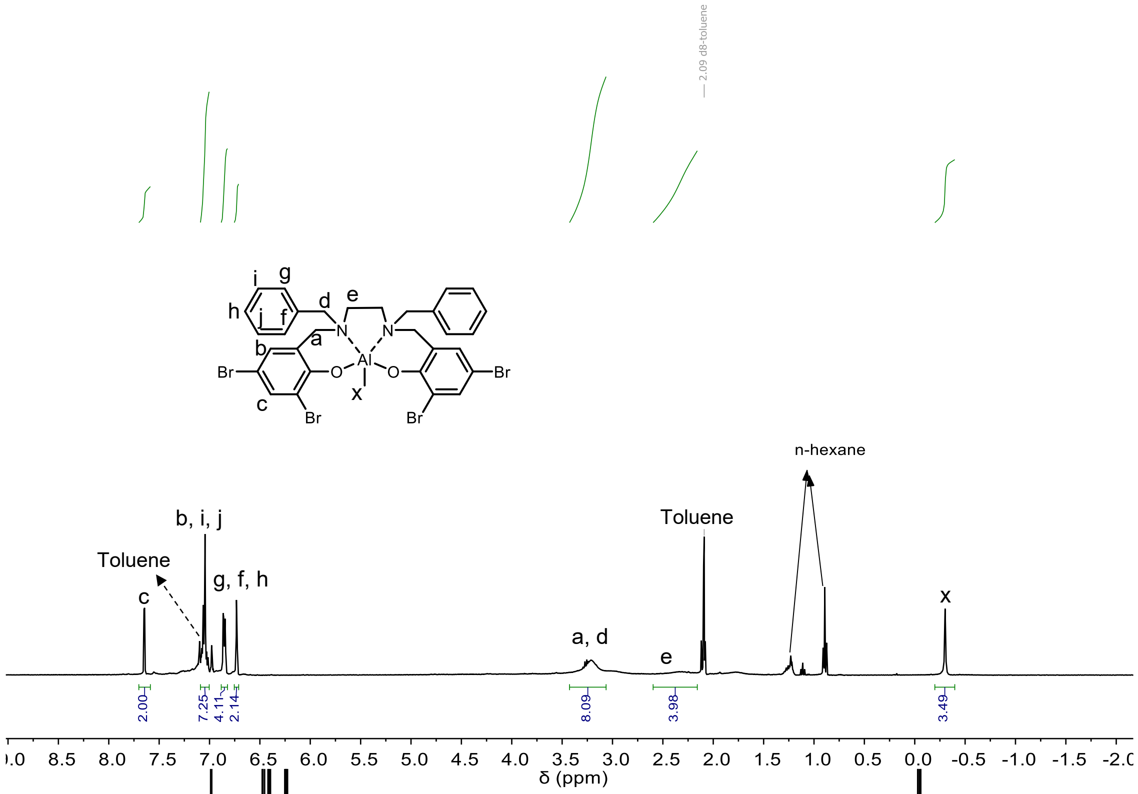

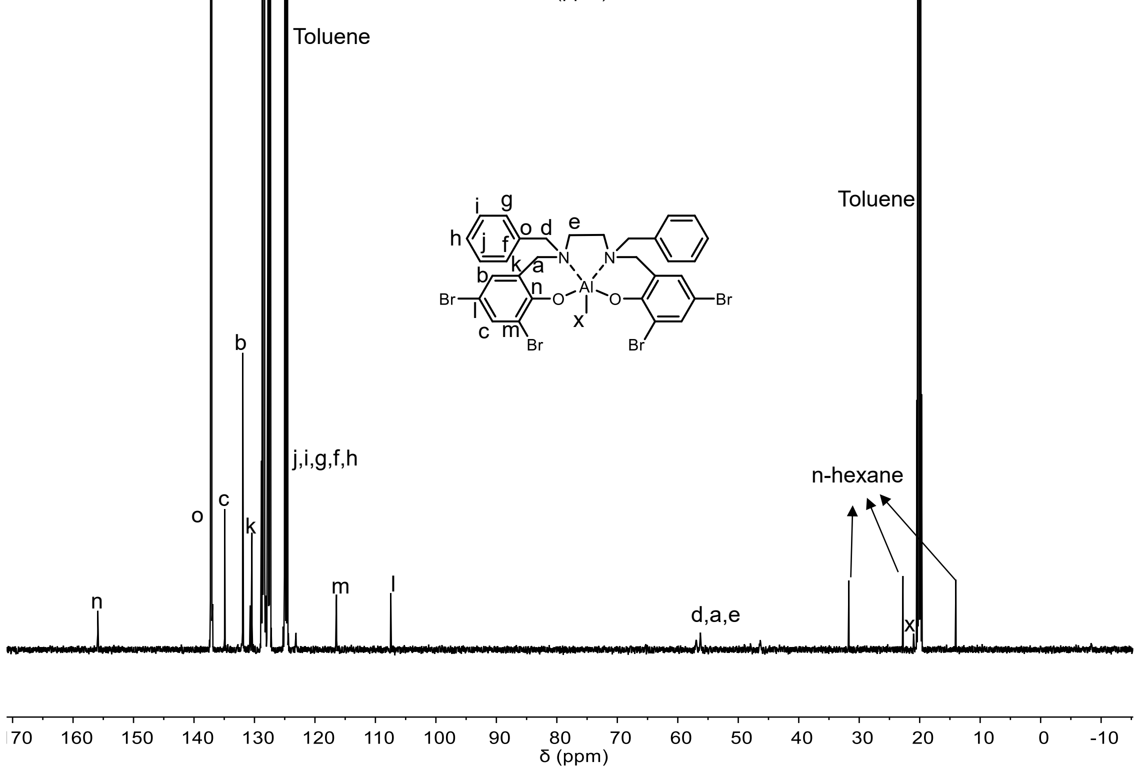

Supplementary Figure 90. ^{1}H NMR (400 MHz, toluene-d_8) and ^{13}C NMR (125 MHz, toluene-d_8) spectra of $(A_{16}C_1B_3)AlMe$.

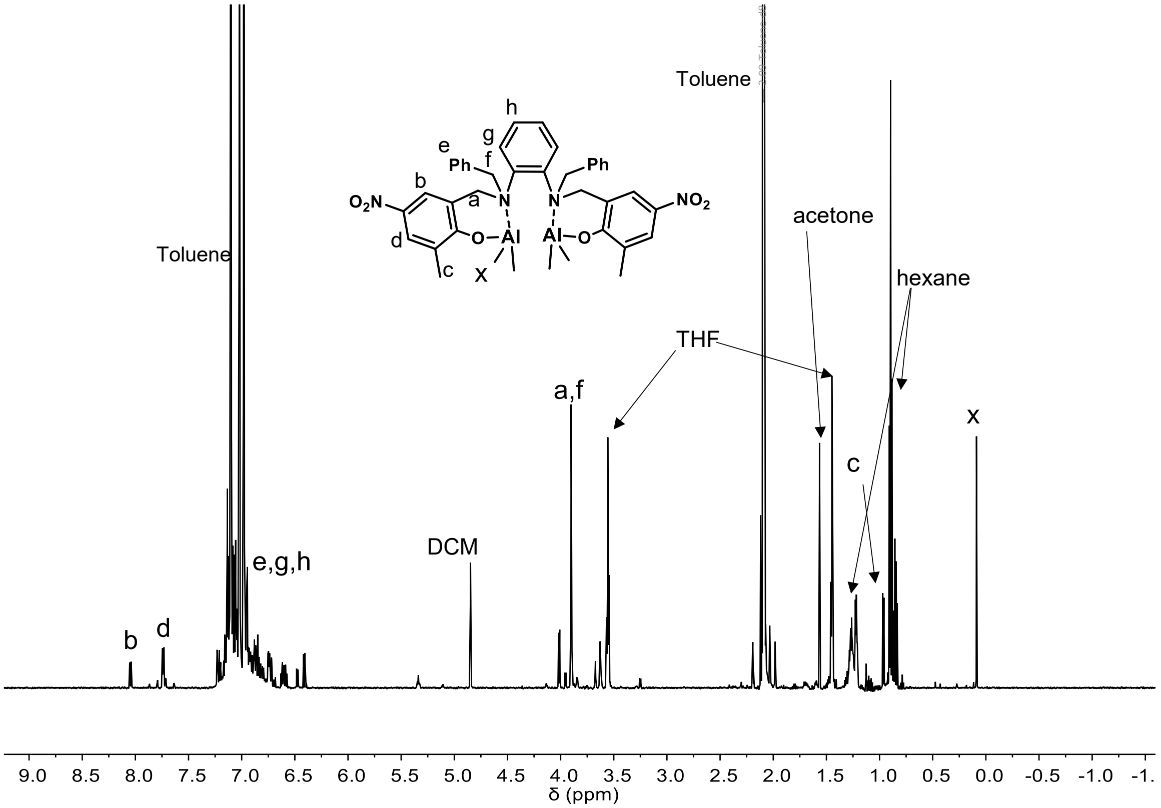

Supplementary Figure 91. ^{1}H NMR (600 MHz, toluene-d_8) spectrum of ($A_5C_6B_3$)AlMe at 85 °C. We note that the Al complex had low solubility in all solvents. We also tested THF-d_8 and $CDCl_3$ at room temperature condition and none of them showed improved solution of the NMR spectrum. ESI-MS confirmed the existence of the complex having two Al centers. Note that ROP of *rac*-LA mediated by this Al complex had a monomer conversion of 16.1%.

S9. Supplementary References

1. H. Chisholm M., S. Iyer S., E. Matison M. Concerning the stereochemistry of poly(lactide), PLA. Previous assignments are shown to be incorrect and a new assignment is proposed. *Chem. Commun.*, 1999-2000 (1997).
2. Zell M. T.*, et al.* Unambiguous Determination of the ^{13}C and ^{1}H NMR stereosequence assignments of polylactide using high-resolution solution NMR spectroscopy. *Macromolecules* **35**, 7700-7707 (2002).
3. Stanford M. J., Dove A. P. Stereocontrolled ring-opening polymerisation of lactide. *Chem. Soc. Rev.* **39**, 486-494 (2010).
4. Nomura N., Ishii R., Yamamoto Y., Kondo T. Stereoselective ring-opening polymerization of a racemic lactide by using achiral salen– and homosalen–aluminum complexes. *Chem. Eur. J.* **13**, 4433-4451 (2007).
5. Kan C., Ge J., Ma H. Aluminum methyl, alkoxide and α-alkoxy ester complexes supported by 6,6′-dimethylbiphenyl-bridged salen ligands: synthesis, characterization and catalysis for *rac*-lactide polymerization. *Dalton Trans.* **45**, 6682-6695 (2016).
6. Tshuva E. Y., Gendeziuk N., Kol M. Single-step synthesis of salans and substituted salans by Mannich condensation. *Tetrahedron Lett.* **42**, 6405-6407 (2001).
7. Hormnirun P., Marshall E. L., Gibson V. C., White A. J. P., Williams D. J. Remarkable stereocontrol in the polymerization of racemic lactide using aluminum initiators supported by tetradentate aminophenoxide ligands. *J. Am. Chem. Soc.* **126**, 2688-2689 (2004).
8. Knight P. D., Clarkson G., Hammond M. L., Kimberley B. S., Scott P. Radical and migratory insertion reaction mechanisms in Schiff base zirconium alkyls. *J. Organomet. Chem.* **690**, 5125-5144 (2005).
9. Price E. W.*, et al.* H4octapa: an acyclic chelator for ^{111}in radiopharmaceuticals. *J. Am. Chem. Soc.* **134**, 8670-8683 (2012).
10. Loro C.*, et al.* Direct synthesis of fluorescent oxazolo-phenoxazines by copper-catalyzed/hypervalent iodine(III)-mediated dimerization/cyclization of 2-benzylamino-phenols. *J. Org. Chem.* **87**, 1032-1042 (2022).
11. Wang Z., He J., Mu Y. Synthesis of chiral salan ligands with bulky substituents and their application in Cu-catalyzed asymmetric Henry reaction. *J. Organomet. Chem.* **928**, 121546 (2020).
12. Declercq J.-P., Delangle P., Dutasta J.-P., Van Oostenryck L., Simon P., Tinant B. Synthesis and molecular structure of new phosphorous-crown compounds containing the thiophosphoryl group. *J. Chem. Soc. Perkin Trans. 2*, 2471-2478 (1996).
13. Hormnirun P., Marshall Edward L., Gibson Vernon C., Pugh Robert I., White Andrew J. P. Study of ligand substituent effects on the rate and stereoselectivity of lactide polymerization using aluminum salen-type initiators. *Proc. Natl. Acad. Sci. U.S.A.* **103**, 15343-15348 (2006).
14. Ovitt T. M., Coates G. W. Stereochemistry of lactide polymerization with chiral catalysts: new opportunities for stereocontrol using polymer exchange mechanisms. *J. Am. Chem. Soc.* **124**, 1316-1326 (2002).
15. Frisch M.*, et al.* Gaussian 16. Gaussian, Inc. Wallingford, CT (2016).

16. Lee C., Yang W., Parr R. G. Development of the Colle-Salvetti correlation-energy formula into a functional of the electron density. *Phys. Rev. B* **37**, 785-789 (1988).
17. Becke A. D. Density-functional exchange-energy approximation with correct asymptotic behavior. *Phys. Rev. A Gen. Phys.* **38**, 3098-3100 (1988).
18. Miehlich B., Savin A., Stoll H., Preuss H. Results obtained with the correlation energy density functionals of Becke and Lee, Yang and Parr. *Chem. Phys. Lett.* **157**, 200-206 (1989).
19. Grimme S., Antony J., Ehrlich S., Krieg H. A consistent and accurate ab initio parametrization of density functional dispersion correction (DFT-D) for the 94 elements H-Pu. *J. Chem. Phys.* **132**, 154104 (2010).
20. Marenich A. V., Cramer C. J., Truhlar D. G. Universal solvation model based on solute electron density and on a continuum model of the solvent defined by the bulk dielectric constant and atomic surface tensions. *J. Phys. Chem. B* **113**, 6378-6396 (2009).
21. Neese F. The ORCA program system. *WIREs Comput Mol Sci.* **2**, 73-78 (2012).
22. Neese F. Software update: The ORCA program system—version 5.0. *WIREs Comput Mol Sci.* **12**, e1606 (2022).
23. Mardirossian N., Head-Gordon M. ωB97M-V: A combinatorially optimized, range-separated hybrid, meta-GGA density functional with VV10 nonlocal correlation. *J. Chem. Phys.* **144**, 214110 (2016).
24. Weigend F. Accurate Coulomb-fitting basis sets for H to Rn. *Phys. Chem. Chem. Phys.* **8**, 1057-1065 (2006).
25. Chan B., Gill P. M. W., Kimura M. Assessment of DFT methods for transition metals with the TMC151 compilation of data sets and comparison with accuracies for main-group chemistry. *J. Chem. Theor. Comput.* **15**, 3610-3622 (2019).
26. Lu T., Chen Q. Shermo: A general code for calculating molecular thermochemistry properties. *Comput. Theor. Chem.* **1200**, 113249 (2021).
27. Shields B. J*., et al.* Bayesian reaction optimization as a tool for chemical synthesis. *Nature* **590**, 89-96 (2021).
28. Brethomé A. V., Fletcher S. P., Paton R. S. Conformational effects on physical-organic descriptors: the case of sterimol steric parameters. *ACS Catal.* **9**, 2313-2323 (2019).
29. Moriwaki H., Tian Y.-S., Kawashita N., Takagi T. Mordred: a molecular descriptor calculator. *J. Cheminform.* **10**, 4 (2018).
30. Kier L. B., Hall L. H. An electrotopological-state index for atoms in molecules. *Pharm. Res.* **7**, 801-807 (1990).
31. Rupp M., Tkatchenko A., Müller K.-R., von Lilienfeld O. A. Fast and accurate modeling of molecular atomization energies with machine learning. *Phys. Rev. Lett.* **108**, 058301 (2012).
32. Rasmussen C. E., Williams C. K. I. *Gaussian Processes for Machine Learning* (The MIT Press, 2005).
33. Agatemor C., Arnold A. E., Cross E. D., Decken A., Shaver M. P. Aluminium salophen and salen initiators in the ring-opening polymerisation of *rac*-lactide and *rac*-β-butyrolactone: Electronic effects on stereoselectivity and polymerisation rates. *J. Organomet. Chem.* **745-746**, 335-340 (2013).

34. Du H., *et al.* Chiral salan aluminium ethyl complexes and their application in lactide polymerization. *Chem. Eur. J.* **15**, 9836-9845 (2009).
35. Luo W., Shi T., Liu S., Zuo W., Li Z. Well-designed unsymmetrical salphen-Al complexes: synthesis, characterization, and ring-opening polymerization catalysis. *Organometallics* **36**, 1736-1742 (2017).
36. Maudoux N., Roisnel T., Dorcet V., Carpentier J.-F., Sarazin Y. Chiral (1,2)-diphenylethylene-salen complexes of triel metals: coordination patterns and mechanistic considerations in the isoselective ROP of lactide. *Chem. Eur. J.* **20**, 6131-6147 (2014).
37. Zhong Z., Dijkstra P. J., Feijen J. [(Salen)Al]-mediated, controlled and stereoselective ring-opening polymerization of lactide in solution and without solvent: synthesis of highly isotactic polylactide stereocopolymers from racemic D,L-lactide. *Angew. Chem. Int. Ed.* **41**, 4510-4513 (2002).
38. Van der Maaten L., Hinton G. Visualizing data using t-SNE. *J. Mach. Learn. Res.* **9**, 2579-2605, (2008).
39. Breiman L. Random forests. *Machine Learning* **45**, 5-32 (2001).
40. Hutter F., Hoos H. H., Leyton-Brown K. Sequential model-based optimization for general algorithm configuration. In: *Learning and Intelligent Optimization* (eds Coello CAC). (Springer Berlin Heidelberg, 2011).
41. Gao B., Li D., Li Y., Duan Q., Duan R., Pang X. Ring-opening polymerization of lactide using chiral salen aluminum complexes as initiators: high productivity and stereoselectivity. *New J. Chem.* **39**, 4670-4675 (2015).
42. Spassky N., Wisniewski M., Pluta C., Le Borgne A. Highly stereoelective polymerization of *rac*-(D,L)-lactide with a chiral Schiff's base/aluminium alkoxide initiator. *Macromol. Chem. Phys.* **197**, 2627-2637 (1996).
43. Press K., Goldberg I., Kol M. Mechanistic insight into the stereochemical control of lactide polymerization by salan–aluminum catalysts. *Angew. Chem. Int. Ed.* **54**, 14858-14861 (2015).
44. Hador R., Botta A., Venditto V., Lipstman S., Goldberg I., Kol M. The dual-stereocontrol mechanism: heteroselective polymerization of *rac*-lactide and syndioselective polymerization of *meso*-lactide by chiral aluminum salan catalysts. *Angew. Chem. Int. Ed.* **58**, 14679-14685 (2019).
45. Jones M. D., *et al.* Metal influence on the iso- and hetero-selectivity of complexes of bipyrrolidine derived salan ligands for the polymerisation of *rac*-lactide. *Chem. Sci.* **6**, 5034-5039 (2015).
46. Peterson A., Hador R., Pink M., Popowski Y., Kol M., Tolman W. B. Defining stereochemistry in the polymerization of lactide by aluminum catalysts: insights into the dual-stereocontrol mechanism. *J. Am. Chem. Soc.* **144**, 20047-20055 (2022).
47. Das M., Sharma P., Sunoj R. B. Machine learning studies on asymmetric relay Heck reaction—potential avenues for reaction development. *J. Chem. Phys.* **156**, 114303 (2022).
48. Zahrt A. F., Henle J. J., Rose B. T., Wang Y., Darrow W. T., Denmark S. E. Prediction of higher-selectivity catalysts by computer-driven workflow and machine learning. *Science* **363**, eaau5631 (2019).

49. Hueffel J. A., Sperger T., Funes-Ardoiz I., Ward J. S., Rissanen K., Schoenebeck F. Accelerated dinuclear palladium catalyst identification through unsupervised machine learning. *Science* **374**, 1134-1140 (2021).
50. Hancock S. L., Mahon M. F., Jones M. D. Aluminium salalen complexes based on 1,2-diaminocyclohexane and their exploitation for the polymerisation of *rac*-lactide. *Dalton Trans.* **42**, 9279-9285 (2013).
51. Pilone A., Press K., Goldberg I., Kol M., Mazzeo M., Lamberti M. Gradient isotactic multiblock polylactides from aluminum complexes of chiral salalen ligands. *J. Am. Chem. Soc.* **136**, 2940-2943 (2014).
52. Britton L., *et al.* Salalens and salans derived from 3-aminopyrrolidine: aluminium complexation and lactide polymerisation. *Eur. J. Inorg. Chem.* **2019**, 2768-2773 (2019).
53. Whitelaw E. L., Loraine G., Mahon M. F., Jones M. D. Salalen aluminium complexes and their exploitation for the ring opening polymerisation of *rac*-lactide. *Dalton Trans.* **40**, 11469-11473 (2011).
54. Pang X., Du H., Chen X., Wang X., Jing X. Enolic schiff base aluminum complexes and their catalytic stereoselective polymerization of racemic lactide. *Chem. Eur. J.* **14**, 3126-3136 (2008).
55. Zhao W., Wang Q., Cui Y., He J., Zhang Y. Living/controlled ring-opening (co)polymerization of lactones by Al-based catalysts with different sidearms. *Dalton Trans.* **48**, 7167-7178 (2019).
56. Gesslbauer S., Savela R., Chen Y., White A. J. P., Romain C. Exploiting noncovalent interactions for room-temperature heteroselective *rac*-lactide polymerization using aluminum catalysts. *ACS Catal.* **9**, 7912-7920 (2019).
57. Williams W. L., Zeng L., Gensch T., Sigman M. S., Doyle A. G., Anslyn E. V. The evolution of data-driven modeling in organic chemistry. *ACS Cent. Sci.* **7**, 1622-1637 (2021).
58. Chamberlain B. M., Cheng M., Moore D. R., Ovitt T. M., Lobkovsky E. B., Coates G. W. Polymerization of lactide with zinc and magnesium β-diiminate complexes: stereocontrol and mechanism. *J. Am. Chem. Soc.* **123**, 3229-3238 (2001).
59. Marshall E. L., Gibson V. C., Rzepa H. S. A computational analysis of the ring-opening polymerization of *rac*-lactide initiated by single-site β-diketiminate metal complexes: defining the mechanistic pathway and the origin of stereocontrol. *J. Am. Chem. Soc.* **127**, 6048-6051 (2005).
