# Supplementary material for: Bayesian-optimization-assisted discovery of stereoselective aluminum complexes for ring-opening polymerization of racemic lactide
Source: Nat Commun. 2023 Jun 20;14:3647. doi: 10.1038/s41467-023-39405-5 (PMC10282063; doi:10.1038/s41467-023-39405-5)
Supplement: Supplementary file 3 — Description of Additional Supplementary Files [file 41467_2023_39405_MOESM3_ESM.pdf]

File name: Supplementary Data 1.

Description: DFT coordinates of all ligands and the fragments of Al complexes, and reaction intermediates as shown in Supplementary Figure 15.
