# Supplementary material for: Bayesian-optimization-assisted discovery of stereoselective aluminum complexes for ring-opening polymerization of racemic lactide
Source: Nat Commun. 2023 Jun 20;14:3647. doi: 10.1038/s41467-023-39405-5 (PMC10282063; doi:10.1038/s41467-023-39405-5)
Supplement: Supplementary file 4 — Supplementary Data 1 [file 41467_2023_39405_MOESM4_ESM.pdf]

Coordinates for fragments

A1

C	2.00267	-0.47925	-0.00000
C	0.89141	-1.31966	0.00009
C	-0.39557	-0.77387	0.00004
C	-0.59141	0.62041	0.00004
C	0.54388	1.43928	-0.00006
C	1.83380	0.90690	-0.00006
H	3.00042	-0.91042	0.00001
H	0.99895	-2.40041	0.00002
H	0.40507	2.51830	-0.00004
H	2.69617	1.56756	0.00011
O	-1.44144	-1.65677	-0.00010
H	-2.27796	-1.16547	0.00013
C	-1.98670	1.19940	0.00003
H	-2.56281	0.89685	-0.88679
H	-2.56334	0.89564	0.88604
H	-1.95356	2.29279	0.00084

A2

C	-1.20800	1.42849	0.01155
C	-1.20789	0.03355	-0.01372
C	-0.00009	-0.66379	-0.02558
C	1.20837	0.03385	-0.01337
C	1.20818	1.42845	0.01142
C	-0.00008	2.12584	0.02427
H	-2.16036	1.97809	0.02135
H	2.16042	1.97856	0.02104
H	0.00002	3.22527	0.04401
C	-2.54177	-0.73599	-0.02667
H	-2.85853	-0.91763	0.97910
H	-3.28337	-0.15576	-0.53488
H	-2.41021	-1.66926	-0.53323
C	2.54181	-0.73645	-0.02655
H	2.59023	-1.38029	0.82669
H	2.60647	-1.32230	-0.91958
H	3.35522	-0.04195	0.00407
O	0.00012	-2.09357	-0.05065
H	-0.00400	-2.42985	0.84852

A3

C	0.22420	-0.75219	-0.00002
C	-1.07327	-0.21468	-0.00003
C	-1.17490	1.19121	-0.00000
C	-0.03236	2.01282	0.00001
C	1.22723	1.41431	0.00001
C	1.38998	0.02390	0.00001
H	0.32406	-1.82811	-0.00001
H	2.09805	2.06529	0.00003
O	-2.42669	1.76247	-0.00002
H	-2.33339	2.72777	-0.00020
C	2.80507	-0.58771	-0.00000
C	3.57151	-0.12115	-1.26001
H	3.66641	0.96956	-1.30052
H	4.58495	-0.54234	-1.27258
H	3.05999	-0.44465	-2.17440
C	3.57139	-0.12155	1.26023
H	3.05978	-0.44537	2.17446
H	4.58484	-0.54273	1.27276
H	3.66625	0.96915	1.30110
C	2.78177	-2.12768	-0.00025
H	2.28055	-2.53106	0.88737
H	2.28049	-2.53078	-0.88797
H	3.80835	-2.51267	-0.00034
C	-2.32351	-1.12339	0.00000
C	-1.95450	-2.62071	-0.00010

H	-1.37968	-2.90714	-0.88858
H	-1.37908	-2.90711	0.88800
H	-2.87380	-3.21791	0.00022
C	-3.17372	-0.85990	-1.26682
H	-4.05689	-1.51171	-1.26994
H	-3.51600	0.17572	-1.31899
H	-2.59636	-1.07588	-2.17425
C	-3.17355	-0.86004	1.26696
H	-3.51570	0.17561	1.31938
H	-4.05680	-1.51175	1.27005
H	-2.59612	-1.07628	2.17429
C	-0.17751	3.51795	0.00003
H	-0.71363	3.88732	-0.88686
H	-0.71376	3.88727	0.88686
H	0.80400	4.00081	0.00008

A4

C	-0.90304	-1.14732	-0.00002
C	0.42090	-1.59424	-0.00001
C	1.47968	-0.68972	0.00001
C	1.22938	0.69741	-0.00001
C	-0.09659	1.12981	-0.00001
C	-1.16698	0.22546	-0.00001
H	-1.70631	-1.87519	-0.00005
H	0.64147	-2.65766	-0.00000
H	-0.32045	2.19313	-0.00001
O	2.75117	-1.20765	-0.00002
H	3.39251	-0.48028	0.00032
O	-2.41808	0.78351	-0.00003
C	2.37458	1.68251	0.00000
H	3.01591	1.57077	0.88637
H	3.01611	1.57054	-0.88619
H	2.00444	2.71163	-0.00018
C	-3.53480	-0.09224	0.00004
H	-3.55357	-0.72903	0.89514
H	-4.41999	0.54815	0.00007
H	-3.55365	-0.72903	-0.89507

A5

C	-0.41439	1.21565	0.00006
C	0.97746	1.22501	0.00005
C	1.65203	-0.01407	-0.00003
C	0.96583	-1.24659	0.00000
C	-0.42417	-1.22051	0.00001
C	-1.10018	0.00152	0.00001
H	-0.97179	2.14459	0.00011
H	-0.99113	-2.14371	-0.00002
N	-2.55679	0.00957	-0.00002
O	-3.14629	-1.07590	-0.00003
O	-3.13440	1.10180	-0.00003
O	3.00899	-0.08675	-0.00013
H	3.38832	0.80744	-0.00009
C	1.73944	-2.53911	0.00006
H	2.39036	-2.61417	-0.87934
H	2.38985	-2.61436	0.87985
H	1.06189	-3.39771	-0.00018
C	1.75754	2.51763	0.00003
H	2.40073	2.61102	0.88677
H	2.39897	2.61203	-0.88787
H	1.08261	3.37757	0.00122

A6

C	-0.52432	-1.15716	-0.00002
C	0.80777	-0.74982	-0.00002
C	1.17258	0.60244	-0.00002
C	0.16436	1.58684	-0.00001
C	-1.17076	1.18239	0.00000

C	-1.50463	-0.17229	-0.00002
H	-0.77902	-2.21018	-0.00002
H	-1.95513	1.93215	0.00000
C	0.55097	3.04314	-0.00001
H	1.15525	3.29395	0.88001
H	1.15580	3.29380	-0.87970
H	-0.33739	3.68120	-0.00035
O	2.46088	1.02792	0.00005
H	3.05434	0.25587	-0.00008
Cl	-3.20185	-0.64927	0.00001
Cl	2.08675	-1.96739	-0.00000

A7

C	0.77206	-1.39370	-0.00007
C	-0.59574	-1.65409	-0.00007
C	-1.51600	-0.60241	0.00000
C	-1.08106	0.73692	-0.00010
C	0.29576	0.98435	-0.00006
C	1.20570	-0.07002	0.00001
H	1.48725	-2.20943	-0.00005
H	-0.96605	-2.67454	-0.00013
H	0.65407	2.00913	-0.00009
O	-2.83931	-0.94069	0.00014
H	-3.38123	-0.13570	-0.00012
C	-2.07991	1.86963	0.00001
H	-2.73006	1.84706	-0.88642
H	-2.72920	1.84746	0.88703
H	-1.57180	2.83792	-0.00053
Cl	2.93804	0.28115	0.00005

A8

C	1.43273	1.27553	-0.28611
C	0.91561	-0.02835	-0.32543
C	1.83544	-1.08504	-0.16324
C	3.19887	-0.85500	0.09013
C	3.64594	0.46454	0.15352
C	2.78199	1.54783	-0.04356
H	0.76824	2.11358	-0.46228
H	4.70142	0.64865	0.34738
C	4.13812	-2.02164	0.26555
H	3.84169	-2.65569	1.11054
H	4.14523	-2.66881	-0.62028
H	5.16116	-1.67493	0.44313
O	1.47193	-2.40887	-0.25374
H	0.55566	-2.47081	-0.56238
C	3.29286	2.96895	0.02072
H	3.50859	3.27617	1.05344
H	4.22265	3.09097	-0.54844
H	2.56031	3.67647	-0.38276
C	-0.55310	-0.35641	-0.59984
H	-0.54666	-1.07210	-1.44228
C	-1.47090	0.80708	-1.07537
C	-1.28482	-1.07966	0.58265
H	-0.96685	1.36131	-1.87731
C	-1.85778	1.77648	0.06819
C	-2.77511	0.18318	-1.62768
H	-0.64556	-1.87059	0.99213
C	-1.62525	-0.08964	1.71514
C	-2.58771	-1.70396	0.03670
H	-2.51697	2.55692	-0.33732
H	-0.98272	2.28836	0.48043
C	-2.57462	1.00682	1.19309
H	-2.54768	-0.48537	-2.46962
H	-3.42658	0.97644	-2.01930
C	-3.50771	-0.59478	-0.51220
H	-0.70684	0.36246	2.11153

H	-2.09467	-0.63637	2.54453
H	-2.35763	-2.43280	-0.75396
H	-3.10071	-2.25576	0.83642
H	-2.82470	1.69750	2.00913
C	-3.86445	0.36904	0.63827
H	-4.42401	-1.04266	-0.91891
H	-4.39326	-0.17428	1.43394
H	-4.54620	1.15254	0.27898

A9

C	0.03845	-0.94161	-0.00162
C	-1.16784	-0.22943	-0.00365
C	-1.09708	1.17269	-0.00328
C	0.14097	1.84459	-0.00198
C	1.31189	1.08413	-0.00039
C	1.28872	-0.31666	0.00003
H	-0.00070	-2.02926	-0.00138
H	2.26448	1.60973	0.00061
O	-2.28128	1.87026	-0.00407
H	-2.08938	2.82062	-0.00549
C	-2.51116	-0.95237	-0.00422
C	-2.70605	-1.79034	1.27315
C	-2.69190	-1.81939	-1.26401
H	-3.29400	-0.18856	-0.01742
H	-2.61974	-1.16981	2.17258
H	-3.69841	-2.25860	1.27977
H	-1.96077	-2.59184	1.34814
H	-2.59411	-1.21998	-2.17651
H	-1.94768	-2.62385	-1.31209
H	-3.68493	-2.28613	-1.27134
C	0.18863	3.35531	-0.00213
H	-0.29750	3.78848	-0.88891
H	-0.29844	3.78864	0.88405
H	1.22313	3.71091	-0.00167
C	2.57370	-1.13678	0.00206
H	2.27816	-2.19489	0.00212
C	3.41558	-0.89544	-1.26448
H	4.30264	-1.54130	-1.27115
H	2.83746	-1.10426	-2.17200
H	3.76261	0.14366	-1.32249
C	3.41228	-0.89434	1.27058
H	3.75936	0.14474	1.32850
H	2.83171	-1.10216	2.17678
H	4.29919	-1.54036	1.28024

A10

C	-0.72549	1.61350	-0.00505
C	-0.44955	0.23659	0.00115
C	-1.54798	-0.64555	0.00352
C	-2.87799	-0.17749	0.00053
C	-3.09667	1.19991	-0.00521
C	-2.02878	2.09974	-0.00816
H	0.10455	2.31537	-0.00748
H	-4.11987	1.56893	-0.00753
H	-2.21336	3.17050	-0.01280
C	-4.01807	-1.16421	0.00334
H	-3.98287	-1.82369	-0.87280
H	-3.98426	-1.81717	0.88440
H	-4.98196	-0.64527	0.00064
O	-1.41790	-2.01114	0.00887
H	-0.47688	-2.24580	0.00949
C	0.99654	-0.24710	0.00390
C	1.76215	0.17606	-1.27238
C	1.76607	0.19934	1.26981
H	1.02194	-1.34926	0.01439
C	3.21151	-0.33313	-1.26667

H	1.76228	1.27259	-1.34242
H	1.23068	-0.19126	-2.15930
C	3.21535	-0.31012	1.26879
H	1.76697	1.29693	1.31994
H	1.23732	-0.15165	2.16492
C	3.96423	0.11076	-0.00401
H	3.73306	0.01961	-2.16577
H	3.21027	-1.43240	-1.31963
H	3.73970	0.05873	2.15975
H	3.21418	-1.40824	1.34166
H	4.98042	-0.30415	-0.00178
H	4.07315	1.20551	-0.01410

A11

C	-0.34653	1.37784	0.00002
C	0.43297	0.21098	-0.00003
C	-0.26919	-1.01313	-0.00007
C	-1.67652	-1.06388	-0.00005
C	-2.39244	0.13392	0.00003
C	-1.74507	1.37162	0.00004
H	0.15068	2.34127	0.00006
H	-3.48048	0.09361	0.00009
O	0.45470	-2.18377	0.00006
H	-0.15869	-2.93488	0.00049
C	-2.38937	-2.39756	-0.00006
H	-2.14690	-3.00163	-0.88704
H	-2.14703	-3.00167	0.88694
H	-3.47404	-2.25647	-0.00009
C	-2.53407	2.66096	0.00001
H	-3.18256	2.73975	-0.88250
H	-3.18480	2.73837	0.88098
H	-1.87225	3.53353	0.00150
C	1.97693	0.26806	-0.00001

C	2.53596	-0.42403	-1.26707
H	3.63248	-0.37505	-1.26983
H	2.24285	-1.47460	-1.31973
H	2.17705	0.07748	-2.17430
C	2.53596	-0.42415	1.26700
H	2.24288	-1.47473	1.31950
H	3.63248	-0.37511	1.26980
H	2.17701	0.07725	2.17426
C	2.50270	1.71764	0.00005
H	2.18340	2.27516	-0.88819
H	2.18348	2.27506	0.88839
H	3.59885	1.70318	0.00001

A12

C	-0.52651	0.44031	0.69276
C	0.85305	0.64284	0.58363
C	1.28772	1.72523	-0.21574
C	0.37183	2.57862	-0.84872
C	-0.99668	2.33637	-0.68970
C	-1.47470	1.26584	0.07023
H	-0.88748	-0.39608	1.28009
H	-1.68796	3.00707	-1.19029
C	0.87514	3.72601	-1.68855
H	1.45700	4.43986	-1.09188
H	1.54022	3.37635	-2.48754
H	0.04244	4.26848	-2.14781
O	2.61175	2.02756	-0.41082
H	3.15330	1.23921	-0.22937
C	1.87127	-0.23021	1.35339
C	2.49559	0.64197	2.47130
H	3.17463	0.05933	3.10406
H	3.04372	1.50203	2.07494
H	1.69717	1.02751	3.11347

C	1.20398	-1.44370	2.04473
H	0.67999	-2.08927	1.33296
H	1.97396	-2.04726	2.53738
H	0.49064	-1.12468	2.81263
C	2.92988	-0.80549	0.38240
C	4.30862	-0.76953	0.64683
C	2.50612	-1.44378	-0.79855
C	5.22837	-1.34944	-0.23446
H	4.68096	-0.28782	1.54490
C	3.41997	-2.02560	-1.67379
H	1.44572	-1.47649	-1.03329
C	4.78915	-1.98149	-1.39529
H	6.28985	-1.30326	-0.00491
H	3.06291	-2.51151	-2.57821
H	5.50321	-2.43105	-2.08006
C	-2.97749	0.98735	0.28910
C	-3.87510	1.87344	-0.60735
H	-3.65995	1.73561	-1.67199
H	-4.92712	1.61444	-0.44558
H	-3.75480	2.93617	-0.36743
C	-3.31556	1.33210	1.75884
H	-2.76580	0.70406	2.46765
H	-3.04419	2.37376	1.96120
H	-4.38726	1.22320	1.96313
C	-3.29017	-0.48512	-0.05505
C	-3.98437	-1.34148	0.80960
C	-2.90420	-0.99602	-1.30708
C	-4.28330	-2.65746	0.43996
H	-4.30096	-0.99261	1.78686
C	-3.20309	-2.30416	-1.68127
H	-2.35152	-0.35995	-1.99355
C	-3.89666	-3.14525	-0.80633
H	-4.82053	-3.29871	1.13461
H	-2.89080	-2.66944	-2.65668
H	-4.12851	-4.16748	-1.09395

A13

C	0.50625	-1.05542	0.00003
C	-0.60477	-0.20113	0.00004
C	-0.33878	1.18775	0.00004
C	0.97256	1.70084	-0.00007
C	2.05000	0.81201	-0.00008
C	1.80420	-0.55358	-0.00002
H	0.36730	-2.12816	0.00008
H	3.06675	1.19141	-0.00019
C	1.20801	3.19393	-0.00009
H	0.77964	3.68211	-0.88768
H	0.77960	3.68214	0.88744
H	2.27830	3.41747	-0.00005
C	-2.04378	-0.76320	-0.00002
C	-2.79737	-0.29278	-1.26790
H	-3.81514	-0.70282	-1.27074
H	-2.87013	0.79546	-1.31959
H	-2.29226	-0.64666	-2.17490
C	-2.05912	-2.30495	-0.00019
H	-1.57410	-2.72549	0.88843
H	-3.09799	-2.65376	-0.00026
H	-1.57405	-2.72528	-0.88888
C	-2.79751	-0.29307	1.26787
H	-3.81527	-0.70315	1.27053
H	-2.29248	-0.64709	2.17485
H	-2.87032	0.79516	1.31977
O	-1.40555	2.05081	0.00028
H	-1.07912	2.96431	0.00024
Cl	3.16797	-1.68144	0.00007

A14

C	-1.02374	1.18291	-0.00335
C	0.37076	1.23852	-0.00017
C	1.08082	0.02516	0.00173
C	0.41918	-1.21201	-0.00014
C	-0.98159	-1.21279	-0.00329
C	-1.72564	-0.03079	-0.00396
H	-1.57953	2.11930	-0.00591
H	-1.49972	-2.16996	-0.00554
O	2.45057	0.11888	0.00335
H	2.83135	-0.77322	0.00398
C	1.20700	-2.50145	-0.00085
H	1.85420	-2.59391	-0.88557
H	1.84992	-2.59738	0.88662
H	0.53729	-3.36646	-0.00412
C	1.11742	2.54828	-0.00076
H	1.76666	2.64193	0.87882
H	1.76883	2.64003	-0.87893
H	0.42266	3.39419	-0.00251
C	-3.23682	-0.05142	0.00450
H	-3.62312	-1.06902	-0.11727
H	-3.64264	0.34373	0.94561
H	-3.65482	0.56132	-0.80431

A15

C	-0.77982	1.67896	-0.00001
C	-0.66041	0.27590	-0.00001
C	-1.86356	-0.45834	-0.00001
C	-3.13692	0.14562	0.00002
C	-3.19502	1.54689	0.00003
C	-2.02765	2.31381	0.00001
H	0.11230	2.29331	-0.00008
H	-4.16693	2.03219	0.00004
H	-2.08739	3.39758	0.00004
O	-1.74140	-1.85383	0.00006
H	-2.61031	-2.29906	-0.00054
C	-4.39367	-0.69608	0.00009
H	-4.46239	-1.34246	-0.88751
H	-4.46212	-1.34264	0.88755
H	-5.28355	-0.06012	0.00021
Si	1.02996	-0.67101	0.00003
C	1.10672	-1.77584	-1.56829
H	2.01768	-2.38589	-1.58074
H	0.24202	-2.44604	-1.57945
H	1.08486	-1.17496	-2.48502
C	1.10650	-1.77619	1.56811
H	0.24085	-2.44518	1.57978
H	2.01660	-2.38754	1.57963
H	1.08628	-1.17549	2.48499
C	2.59293	0.52587	0.00006
C	2.63181	1.41187	-1.26886
H	1.77344	2.08892	-1.34040
H	3.54090	2.03297	-1.26710
H	2.65288	0.80589	-2.18322
C	3.86348	-0.36520	0.00108
H	4.76317	0.26891	0.00066
H	3.91641	-1.00620	0.88942
H	3.91685	-1.00790	-0.88602
C	2.63136	1.41350	1.26782
H	3.54105	2.03374	1.26604
H	1.77360	2.09147	1.33763
H	2.65101	0.80871	2.18299

A16

C	-0.25139	-0.78306	0.00001
C	1.00881	-0.19228	0.00002

C	1.18040	1.19430	0.00003
C	0.04929	2.03682	0.00001
C	-1.21985	1.44687	0.00001
C	-1.35635	0.05973	0.00001
H	-0.36121	-1.85900	-0.00000
H	-2.09922	2.07960	0.00002
C	0.23047	3.53213	-0.00002
H	0.79625	3.86298	0.87901
H	0.79590	3.86300	-0.87928
H	-0.73684	4.04221	0.00018
O	2.41688	1.80726	-0.00001
H	3.13886	1.14381	0.00001
Br	-3.14165	-0.72129	-0.00000
Br	2.60688	-1.31750	-0.00001
C1B1			
C	0.64458	-1.37133	-0.36813
H	0.40112	-1.61510	-1.41037
H	1.32107	-2.15463	0.01632
C	-0.66117	-1.39245	0.44220
H	-1.00559	-2.43382	0.49490
H	-0.45584	-1.05175	1.47187
N	-1.69600	-0.61035	-0.21493
N	1.25076	-0.04896	-0.34276
C	2.45756	0.04815	0.04305
H	3.04090	-0.83863	0.34801
C	-2.13275	0.42399	0.37957
H	-1.75561	0.72275	1.37253
C	3.17621	1.36174	0.10913
H	3.53241	1.55583	1.12991
H	4.06443	1.34734	-0.53696
H	2.51814	2.17674	-0.20420
C	-3.18296	1.30877	-0.21993

H	-3.49649	0.92795	-1.19573
H	-4.05768	1.37438	0.44127
H	-2.79897	2.33088	-0.33898
C1B2			
C	0.73903	-0.33776	-1.00887
H	0.83641	0.66907	-1.42355
H	1.04836	-1.04819	-1.80416
C	-0.75673	-0.55885	-0.72841
H	-1.23964	-0.62916	-1.71075
H	-0.91881	-1.53711	-0.23958
N	1.63411	-0.45103	0.14989
N	-1.39512	0.53934	-0.00012
C	2.93031	0.19830	-0.09045
H	3.61624	-0.15077	0.69041
H	3.36888	-0.12683	-1.05541
C	1.81891	-1.84335	0.54460
H	2.30769	-2.45529	-0.24014
H	2.43544	-1.89403	1.44873
H	0.85514	-2.30696	0.77530
C	2.87249	1.72462	-0.03229
H	2.48828	2.05830	0.93786
H	3.87818	2.14105	-0.16691
H	2.23391	2.15327	-0.81198
C	-2.79647	0.75837	-0.37123
H	-3.12133	1.68367	0.12326
H	-2.82542	0.95646	-1.45098
C	-3.79902	-0.36134	-0.04201
H	-4.80478	-0.07295	-0.37223
H	-3.54181	-1.29950	-0.54802
H	-3.85084	-0.56203	1.03410
C	-1.18646	0.48171	1.44230
H	-1.61042	1.37930	1.90921

H	-1.64651	-0.40112	1.92760
H	-0.11417	0.45444	1.64702
C1B3			
C	1.54525	1.76843	-0.72276
H	1.07176	2.75463	-0.72131
H	2.43435	1.86597	-1.37527
C	0.57680	0.76870	-1.39915
H	0.62866	0.96994	-2.47682
H	0.94295	-0.25165	-1.26305
N	1.96825	1.45699	0.65811
N	-0.83706	0.78118	-1.01810
C	2.17837	2.68355	1.45070
H	2.72582	2.39880	2.35598
H	2.82329	3.40619	0.91333
C	3.18514	0.62546	0.68602
H	3.97914	1.06135	0.04920
H	3.56437	0.64868	1.71444
C	0.88388	3.37136	1.88143
H	0.26230	2.68963	2.47221
H	1.11865	4.24588	2.50069
H	0.28785	3.72423	1.03309
C	-1.56405	2.02678	-1.26704
H	-2.59399	1.86149	-0.93237
H	-1.17970	2.86802	-0.65862
C	-1.59748	2.43887	-2.73935
H	-2.27104	3.29404	-2.87034
H	-0.61409	2.73912	-3.11815
H	-1.96456	1.61456	-3.36151
C	-1.13926	0.19724	0.28785
H	-1.01470	0.91387	1.11803
H	-0.40360	-0.59543	0.46514
C	2.99081	-0.82947	0.30001

C	3.65036	-1.37990	-0.80421
C	2.18075	-1.66662	1.08251
C	3.49931	-2.73116	-1.13009
H	4.28760	-0.74531	-1.41657
C	2.02603	-3.01439	0.76114
H	1.66820	-1.25020	1.94622
C	2.68527	-3.55141	-0.34923
H	4.01782	-3.13970	-1.99385
H	1.39462	-3.64859	1.37835
H	2.56558	-4.60223	-0.59983
C	-2.53429	-0.40835	0.36492
C	-3.30991	-0.26614	1.52180
C	-3.04989	-1.16491	-0.69695
C	-4.56528	-0.87089	1.62433
H	-2.92687	0.32531	2.35134
C	-4.30444	-1.76689	-0.60077
H	-2.45841	-1.26379	-1.60294
C	-5.06693	-1.62411	0.56209
H	-5.15274	-0.74655	2.53086
H	-4.68895	-2.34977	-1.43449
H	-6.04504	-2.09270	0.63670
C2B1			
C	-1.39857	-1.53563	-0.02372
H	-2.14972	-2.21826	0.39741
H	-1.47526	-1.60655	-1.12380
C	-0.01384	-2.01786	0.44042
H	0.12598	-1.72424	1.48833
H	-0.00168	-3.11548	0.41079
C	1.18246	-1.53424	-0.39601
H	2.09755	-2.03007	-0.02435
H	1.03580	-1.85985	-1.43584
N	-1.71985	-0.20433	0.47419

N	1.31376	-0.08482	-0.40749
C	-2.04698	0.69088	-0.36470
H	-2.07525	0.48691	-1.44870
C	2.35984	0.42449	0.10246
H	3.15618	-0.20568	0.53705
C	-2.40377	2.08560	0.05173
H	-2.36087	2.18501	1.13983
H	-1.71162	2.80764	-0.40261
H	-3.41146	2.35021	-0.29634
C	2.59203	1.90435	0.15251
H	2.71669	2.23898	1.19120
H	3.51683	2.16914	-0.37788
H	1.75256	2.44070	-0.29808

C2B2

C	-1.04756	-1.09879	-0.87145
H	-1.76530	-1.72375	-1.43703
H	-0.48076	-0.52515	-1.61131
C	-0.06739	-1.99311	-0.08401
H	-0.37131	-2.03271	0.96788
H	-0.12027	-3.02638	-0.45155
C	1.40336	-1.55930	-0.20192
H	2.02168	-2.22884	0.43225
H	1.72527	-1.73100	-1.23743
N	1.65945	-0.14885	0.09563
N	-1.76184	-0.14104	-0.01359
C	1.37586	0.17590	1.48903
H	1.99202	-0.40988	2.20173
H	1.56517	1.23696	1.67469
H	0.31943	-0.00452	1.70034
C	3.02056	0.22887	-0.29870
H	3.77988	-0.24124	0.36012
H	3.19042	-0.17667	-1.30359
C	3.25064	1.73982	-0.34524
H	3.22960	2.20150	0.64757
H	4.23365	1.95350	-0.78164
H	2.48642	2.22454	-0.96349
C	-2.88920	-0.78230	0.65252
H	-3.32356	-0.12012	1.40723
H	-3.69371	-1.07743	-0.05197
H	-2.55290	-1.68724	1.16883
C	-2.16048	1.06186	-0.75092
H	-2.91175	0.82947	-1.53430
H	-1.26605	1.42887	-1.26670
C	-2.69538	2.17955	0.14452
H	-2.83198	3.09383	-0.44498
H	-3.66433	1.93506	0.59306
H	-1.98731	2.39534	0.95295

C2B3

C	-0.90773	0.26233	0.17594
H	-1.63037	0.92695	0.68678
H	-0.11708	0.04861	0.90286
C	-0.26003	1.01883	-0.99158
H	0.29841	0.30392	-1.60906
H	-1.01993	1.46790	-1.64371
C	0.66402	2.14390	-0.50116
H	0.89078	2.83208	-1.33642
H	0.12071	2.73792	0.24313
N	1.89679	1.64533	0.13375
N	-1.49725	-1.02414	-0.20848
C	2.89640	1.25836	-0.87323
H	2.35575	1.00112	-1.79192
H	3.54170	2.11673	-1.13764
C	2.42237	2.60606	1.11500
H	2.79261	3.52284	0.61217

H	1.58044	2.91426	1.74535
C	3.52214	2.05526	2.02126
H	4.43771	1.81409	1.47375
H	3.77710	2.80648	2.77889
H	3.18715	1.14810	2.53557
C	-2.64449	-0.91871	-1.13112
H	-2.88621	-1.93043	-1.47259
H	-2.30438	-0.37002	-2.01539
C	-1.72668	-1.90028	0.94664
H	-2.58383	-1.57338	1.56364
H	-0.84065	-1.81373	1.58614
C	-1.89548	-3.37321	0.56901
H	-1.92288	-3.98836	1.47634
H	-2.82149	-3.56962	0.01763
H	-1.05595	-3.70892	-0.05034
C	3.77181	0.06639	-0.51566
C	3.23864	-1.06530	0.11528
C	5.12320	0.05160	-0.88381
C	4.03707	-2.18038	0.37226
H	2.19354	-1.06281	0.40947
C	5.92504	-1.06364	-0.63039
H	5.55337	0.92402	-1.37228
C	5.38385	-2.18487	0.00021
H	3.60621	-3.04911	0.86426
H	6.97266	-1.05280	-0.92113
H	6.00540	-3.05334	0.20275
C	-3.90951	-0.25992	-0.58969
C	-4.90143	-1.01844	0.05070
C	-4.11182	1.12367	-0.70625
C	-6.04917	-0.41591	0.56920
H	-4.78079	-2.09632	0.13145
C	-5.25750	1.73232	-0.19029
H	-3.36998	1.73013	-1.22088
C	-6.22952	0.96368	0.45289
H	-6.80577	-1.02524	1.05746
H	-5.39418	2.80566	-0.29716
H	-7.12380	1.43467	0.85277

C3B1

C	-3.04637	-0.19702	-0.35562
H	-3.57055	-0.96496	0.22566
H	-3.55984	0.75675	-0.18441
H	-3.15738	-0.44888	-1.41843
C	-1.56284	-0.11208	0.05630
C	-1.46396	0.19744	1.55824
H	-0.42296	0.26396	1.88394
H	-1.96415	1.14625	1.79273
H	-1.94707	-0.58957	2.15048
C	-0.91783	-1.48810	-0.26610
H	-0.89220	-1.62730	-1.36243
H	-1.58791	-2.25974	0.14038
C	-0.93339	1.03662	-0.77838
H	-1.02044	0.76956	-1.84161
H	-1.53664	1.94990	-0.62150
N	0.38188	-1.70854	0.34808
N	0.47533	1.27171	-0.50596
C	1.34732	-2.06228	-0.39631
H	1.22364	-2.18126	-1.48683
C	0.82322	2.42898	-0.11340
H	0.08360	3.23787	0.02442
C	2.71963	-2.33001	0.14289
H	3.04720	-3.34707	-0.11176
H	3.44647	-1.64030	-0.30761
H	2.73649	-2.20708	1.22926
C	2.24869	2.78333	0.18523

H	2.58229	3.61216	-0.45397
H	2.35082	3.12544	1.22391
H	2.90137	1.92085	0.02578

C3B2

C	-0.05428	2.64082	-1.29795
H	-0.95185	3.25506	-1.15142
H	0.81575	3.30412	-1.22286
H	-0.08626	2.24682	-2.32221
C	0.02267	1.50279	-0.25416
C	0.22681	2.15255	1.12706
H	0.40124	1.41606	1.91379
H	1.08695	2.83408	1.11288
H	-0.65229	2.74724	1.40661
C	-1.34195	0.75043	-0.36488
H	-1.57218	0.68066	-1.43565
H	-2.11674	1.41074	0.07371
C	1.22279	0.59311	-0.65409
H	0.90883	-0.00306	-1.51942
H	2.03413	1.24630	-1.01734
N	-1.44204	-0.61595	0.15052
N	1.74087	-0.30309	0.38867
C	-1.45417	-0.71891	1.60498
H	-1.66965	-1.75456	1.89183
H	-0.46880	-0.46658	2.00162
H	-2.20501	-0.06967	2.09376
C	2.97351	0.17456	0.99907
H	2.81735	1.16244	1.44381
H	3.27294	-0.50658	1.80488
H	3.82434	0.26233	0.29632
C	1.79429	-1.71895	0.01025
H	2.00294	-2.29415	0.92309
H	0.78638	-1.99683	-0.31332
C	2.81710	-2.11479	-1.06844
H	3.84782	-1.91572	-0.75253
H	2.74050	-3.18839	-1.28234
H	2.64491	-1.57826	-2.00953
C	-2.52822	-1.37783	-0.48095
H	-2.42589	-2.42034	-0.15206
H	-2.34782	-1.37364	-1.56387
C	-3.96921	-0.91132	-0.21218
H	-4.67772	-1.57002	-0.72976
H	-4.21396	-0.93852	0.85595
H	-4.14383	0.10896	-0.57255

C3B3

C	0.54425	3.57966	1.26087
H	-0.20983	4.15684	1.80961
H	1.42165	3.48042	1.91133
H	0.84547	4.17369	0.38781
C	0.00030	2.19224	0.84890
C	-0.33621	1.40759	2.12629
H	-0.69856	0.40021	1.90572
H	0.55282	1.31145	2.76326
H	-1.09879	1.92603	2.72180
C	-1.24739	2.48759	-0.03559
H	-0.87935	3.03761	-0.90955
H	-1.89231	3.20069	0.51167
C	1.15394	1.49899	0.05149
H	1.27853	2.05207	-0.88851
H	2.08237	1.67851	0.60995
N	-2.05118	1.35614	-0.53542
N	1.03381	0.07058	-0.23464
C	-3.12298	0.98012	0.40041
C	1.82018	-0.84478	0.59860
H	1.61289	-0.59443	1.64505

H	1.41562	-1.85193	0.43610
C	0.82603	-0.30130	-1.63359
H	0.67087	-1.38797	-1.66010
H	-0.12349	0.14731	-1.94751
C	1.91072	0.07284	-2.66059
H	2.87060	-0.40469	-2.44643
H	1.58970	-0.24314	-3.66206
H	2.07907	1.15546	-2.70196
C	-2.58336	1.65352	-1.87699
H	-1.73952	1.97454	-2.49810
H	-3.29014	2.50814	-1.83875
C	-3.26963	0.47897	-2.57232
H	-3.55318	0.77903	-3.58871
H	-4.18040	0.15903	-2.05777
H	-2.60247	-0.38640	-2.64340
H	-4.05469	1.52988	0.16980
H	-2.82838	1.31459	1.39948
C	-3.43291	-0.50695	0.49127
C	-4.73931	-0.93394	0.76371
C	-2.42206	-1.47058	0.37915
C	-5.03249	-2.28970	0.92417
H	-5.53655	-0.19748	0.84837
C	-2.71337	-2.82693	0.53734
H	-1.41021	-1.14174	0.16159
C	-4.01851	-3.24275	0.81081
H	-6.05362	-2.60068	1.13134
H	-1.91755	-3.56255	0.44544
H	-4.24398	-4.29940	0.93091
C	3.33737	-0.90300	0.41409
C	3.92083	-1.84330	-0.44847
C	4.18964	-0.04435	1.12443
C	5.30415	-1.90458	-0.62238

H	3.28198	-2.54184	-0.98528
C	5.57471	-0.09625	0.95108
H	3.76803	0.66148	1.83644
C	6.13655	-1.02426	0.07298
H	5.73277	-2.64434	-1.29410
H	6.21494	0.58032	1.51189
H	7.21445	-1.07072	-0.05890

C4B1

C	-0.34714	0.48178	2.45803
H	-0.26139	-0.56214	2.77122
H	-0.42844	1.10005	3.36229
H	-1.27709	0.57124	1.89280
C	0.87191	0.93593	1.64431
H	0.80170	2.02053	1.47304
H	1.76375	0.79798	2.27310
C	1.17068	0.27055	0.26855
C	2.65869	0.62833	-0.05332
H	2.79022	1.70226	0.13828
H	3.29121	0.11392	0.68351
C	3.19632	0.32321	-1.45840
H	4.25763	0.59440	-1.51353
H	3.12217	-0.73867	-1.71860
H	2.67790	0.89422	-2.23653
C	0.29830	0.87270	-0.86882
H	0.69745	1.87400	-1.11555
H	0.42741	0.24919	-1.76451
C	1.07828	-1.28015	0.31989
H	1.57979	-1.69502	-0.57140
H	1.65431	-1.61278	1.19567
N	-0.26492	-1.82058	0.45665
N	-1.13113	0.93448	-0.60197
C	-1.71185	2.05535	-0.74836

H	-1.15329	2.96108	-1.04615
C	-0.68580	-2.60878	-0.44524
H	-0.06260	-2.87378	-1.31868
C	-2.04970	-3.22795	-0.39800
H	-2.63612	-2.92801	-1.27707
H	-1.98058	-4.32400	-0.42123
H	-2.57986	-2.91824	0.50643
C	-3.18412	2.23298	-0.52981
H	-3.64809	1.28516	-0.24368
H	-3.36993	2.97737	0.25635
H	-3.66708	2.61035	-1.44147

C4B2

C	-1.41881	-2.10389	-2.00298
H	-0.56233	-2.12828	-2.68590
H	-2.10345	-2.90002	-2.32236
H	-1.92144	-1.14366	-2.13960
C	-0.99074	-2.33929	-0.54546
H	-1.87999	-2.54322	0.06686
H	-0.40424	-3.26962	-0.52069
C	-0.15238	-1.23992	0.18271
C	0.46799	-1.96229	1.42166
H	-0.31305	-2.61023	1.84632
H	1.24405	-2.64950	1.05692
C	1.03721	-1.11497	2.57095
H	1.53895	-1.76570	3.29853
H	1.75701	-0.37014	2.22444
H	0.24692	-0.58276	3.11189
C	-1.02030	-0.06197	0.71368
H	-1.61441	-0.42821	1.56834
H	-0.32391	0.67828	1.11998
C	0.91504	-0.72236	-0.82288
H	1.38451	-1.59170	-1.31494
H	0.37221	-0.18641	-1.60739
N	-1.89924	0.61555	-0.25547
N	1.95917	0.17672	-0.29085
C	3.21582	-0.53196	-0.05969
H	3.69985	-0.85537	-1.00305
H	3.92468	0.10119	0.48300
H	3.04433	-1.42283	0.54816
C	2.17101	1.35618	-1.14112
H	2.65915	1.08179	-2.09883
H	1.18563	1.75746	-1.39845
C	2.97865	2.46140	-0.46021
H	4.02021	2.17391	-0.28137
H	2.99311	3.35671	-1.09325
H	2.52848	2.72787	0.50318
C	-1.77518	2.07903	-0.25670
H	-2.32767	2.45369	-1.12917
H	-0.72053	2.32171	-0.42963
C	-2.25684	2.82734	0.99804
H	-2.07587	3.90342	0.88396
H	-3.33014	2.69133	1.17201
H	-1.72424	2.49575	1.89726
C	-3.28939	0.18223	-0.17182
H	-3.88298	0.69775	-0.93641
H	-3.36729	-0.89134	-0.36326
H	-3.75956	0.37492	0.81054

C4B3

C	0.88999	1.58041	-2.70077
H	-0.01890	0.98794	-2.85949
H	1.22328	1.91579	-3.69065
H	1.64775	0.91949	-2.28007
C	0.63858	2.79953	-1.80309
H	1.54715	3.41760	-1.78931

H	-0.12360	3.42458	-2.29247
C	0.17710	2.59200	-0.32467
C	-0.19080	4.03198	0.16423
H	0.66535	4.68850	-0.04532
H	-1.00913	4.40116	-0.46987
C	-0.59624	4.22354	1.63122
H	-0.81046	5.28147	1.82300
H	-1.50072	3.66133	1.88670
H	0.19502	3.92194	2.32591
C	1.31934	2.07505	0.60099
H	1.95472	2.94386	0.83669
H	0.87161	1.77685	1.55149
C	-1.12531	1.69330	-0.29888
H	-1.97355	2.32885	0.00290
H	-1.34436	1.39741	-1.32654
N	-1.10643	0.47133	0.52286
N	2.19009	0.97425	0.12060
C	3.51515	1.50845	-0.24257
H	4.04113	1.87613	0.66312
C	-1.73083	0.65071	1.83829
H	-2.81281	0.86396	1.74746
C	-1.53616	-0.52757	2.79392
H	-0.47582	-0.77632	2.91264
H	-1.93602	-0.26704	3.78040
H	-2.06303	-1.42466	2.45518
C	4.44288	0.55798	-1.00035
H	3.96359	0.16766	-1.90369
H	5.34424	1.10629	-1.30015
H	4.75769	-0.29495	-0.39467
C	2.26639	-0.12921	1.08727
H	2.97312	0.09869	1.91008
H	1.27238	-0.21051	1.53593

C	-1.49931	-0.75926	-0.17637
H	-1.03051	-1.60970	0.33193
H	-1.03217	-0.72120	-1.16746
H	3.34364	2.38723	-0.87254
H	-1.28098	1.53989	2.29167
C	2.60328	-1.49786	0.51606
C	3.42001	-2.37767	1.23680
C	2.04261	-1.94373	-0.68799
C	3.67260	-3.66992	0.77097
H	3.86614	-2.04585	2.17249
C	2.29560	-3.23206	-1.15910
H	1.41671	-1.26413	-1.25677
C	3.11122	-4.10193	-0.43103
H	4.31294	-4.33521	1.34487
H	1.85523	-3.55857	-2.09818
H	3.30938	-5.10511	-0.79939
C	-2.98936	-1.07066	-0.33165
C	-3.45709	-2.35461	-0.01979
C	-3.91695	-0.12992	-0.80536
C	-4.80095	-2.69716	-0.18004
H	-2.75476	-3.09942	0.34981
C	-5.26285	-0.46433	-0.95988
H	-3.58966	0.87351	-1.05897
C	-5.71086	-1.74998	-0.64965
H	-5.13591	-3.70148	0.06681
H	-5.96332	0.28168	-1.32699
H	-6.75912	-2.00928	-0.77210

C5B1

C	0.21332	-0.76896	0.22167
C	0.56938	0.73186	0.07328
C	2.02263	0.99117	0.51391
C	3.02402	0.11314	-0.24709

C	2.67246	-1.37380	-0.10594
C	1.22596	-1.64545	-0.53962
H	2.25222	2.05469	0.37095
H	0.47240	0.98415	-0.99338
H	0.25618	-1.01599	1.29758
H	3.02024	0.38808	-1.31208
H	4.04051	0.30276	0.12053
H	3.35986	-1.98919	-0.70020
H	2.80477	-1.67971	0.94211
H	1.11345	-1.45077	-1.61509
H	0.96697	-2.70046	-0.38431
H	2.09607	0.79866	1.59297
N	-1.11998	-1.00543	-0.32362
N	-0.31514	1.51829	0.92931
C	-2.00844	-1.48942	0.44617
H	-1.79129	-1.72303	1.50189
C	-1.20331	2.27853	0.42368
H	-1.81263	2.83583	1.14442
C	-3.40380	-1.77787	-0.01869
H	-3.64427	-2.84057	0.12052
H	-4.13201	-1.20992	0.57590
H	-3.52132	-1.51883	-1.07453
C	-1.53880	2.51421	-1.03351
H	-2.43643	3.13367	-1.12067
H	-0.72345	3.03284	-1.55475
H	-1.71591	1.56724	-1.55483

C5B2

C	-0.63671	0.58640	-0.23287
C	0.81621	0.16199	0.17545
C	1.78730	1.25527	-0.33216
C	1.44794	2.66478	0.16806
C	0.03328	3.06798	-0.24696
C	-0.96708	2.02198	0.24940
H	2.81133	1.01419	-0.03358
H	0.88418	0.15772	1.28138
H	-0.62292	0.59184	-1.33030
H	1.53154	2.69807	1.26439
H	2.18738	3.37570	-0.22297
H	-0.22276	4.05946	0.14922
H	-0.01895	3.14366	-1.34311
H	-0.97813	2.04424	1.34780
H	-1.98235	2.28161	-0.07610
H	1.77063	1.25971	-1.43142
N	-1.68751	-0.38813	0.13627
N	1.16022	-1.17964	-0.35813
C	-2.05772	-0.38071	1.54778
H	-2.71076	0.46415	1.83539
H	-2.58528	-1.30567	1.80382
H	-1.16056	-0.34011	2.17252
C	-2.85448	-0.34492	-0.74798
H	-2.48296	-0.25603	-1.77521
H	-3.48753	0.54620	-0.56161
C	-3.73256	-1.59509	-0.65933
H	-4.22514	-1.69426	0.31401
H	-4.52207	-1.54770	-1.41899
H	-3.13928	-2.49924	-0.83734
C	0.66724	-2.27757	0.46959
H	-0.41823	-2.22674	0.53392
H	0.93937	-3.23149	0.00109
H	1.08113	-2.28103	1.49682
C	2.54321	-1.43581	-0.78468
H	2.51311	-2.36136	-1.37613
H	2.85492	-0.65314	-1.48040
C	3.61540	-1.60716	0.30856

H	3.40937	-2.47196	0.94886
H	4.59684	-1.76932	-0.15524
H	3.69598	-0.72694	0.95696

C5B3

C	-1.79976	1.13405	-0.57587
C	-0.84485	1.13314	0.66083
C	-1.27026	2.23750	1.66417
C	-2.74214	2.15189	2.08414
C	-3.64747	2.21243	0.85103
C	-3.28231	1.09302	-0.13087
H	-0.61869	2.18652	2.54585
H	-0.97331	0.16709	1.16152
H	-1.66019	2.10333	-1.06897
H	-2.92236	1.21008	2.62309
H	-2.98219	2.96409	2.78294
H	-4.70370	2.12584	1.13804
H	-3.53469	3.19181	0.36241
H	-3.49515	0.13322	0.35571
H	-3.92405	1.14491	-1.02043
H	-1.10054	3.22158	1.20581
N	-1.44580	0.11336	-1.59757
N	0.57724	1.19534	0.28275
C	-2.11941	-1.18904	-1.49533
H	-3.21916	-1.09657	-1.51108
H	-1.86251	-1.73910	-2.40719
C	-1.50459	0.61716	-2.98022
H	-1.26856	1.68536	-2.95821
H	-2.52310	0.53369	-3.40764
C	-0.50614	-0.06103	-3.92169
H	-0.68454	-1.13725	-4.02561
H	-0.58092	0.37806	-4.92451
H	0.51825	0.07856	-3.55954
C	1.49293	0.55315	1.21565
H	0.98150	-0.33567	1.60634
H	1.73444	1.17330	2.09881
C	1.07676	2.43666	-0.32092
H	1.81607	2.17288	-1.08957
H	0.24532	2.90552	-0.85577
C	1.72828	3.48197	0.60335
H	2.01900	4.35719	0.00837
H	1.05720	3.82416	1.39745
H	2.63953	3.09606	1.07316
C	-1.71852	-2.06574	-0.32011
C	-2.69071	-2.61919	0.52206
C	-0.37577	-2.40689	-0.10239
C	-2.33580	-3.48410	1.56151
H	-3.73882	-2.37598	0.35993
C	-0.01637	-3.26937	0.93315
H	0.38851	-1.98806	-0.74913
C	-0.99620	-3.81033	1.77170
H	-3.10729	-3.90085	2.20432
H	1.03034	-3.52209	1.08361
H	-0.71574	-4.48212	2.57892
C	2.80274	0.09960	0.58349
C	2.84935	-0.35705	-0.74069
C	3.98262	0.08326	1.33808
C	4.04253	-0.82640	-1.29223
H	1.93650	-0.32580	-1.32862
C	5.17718	-0.39146	0.79192
H	3.96521	0.44388	2.36499
C	5.21120	-0.84825	-0.52694
H	4.06064	-1.17430	-2.32240
H	6.08209	-0.39737	1.39478
H	6.14099	-1.21267	-0.95640

C6B1

C	-0.88490	-0.47229	-0.43536
C	-0.04633	0.56922	0.03176
C	-0.62887	1.76801	0.47280
C	-2.01097	1.95001	0.44921
C	-2.83283	0.93105	-0.03563
C	-2.26936	-0.26144	-0.48753
H	0.01696	2.54453	0.87412
H	-2.44069	2.88047	0.81017
H	-3.91140	1.06124	-0.06244
H	-2.89569	-1.05584	-0.88400
N	-0.35869	-1.65983	-0.98915
N	1.34508	0.33430	0.09997
C	0.20188	-2.55887	-0.28056
H	0.58951	-3.42142	-0.83354
C	2.15868	1.23741	-0.28790
H	1.80312	2.18019	-0.73120
C	0.40856	-2.59953	1.20521
H	-0.20123	-1.86013	1.72993
H	0.18036	-3.60128	1.58918
H	1.46343	-2.39448	1.43002
C	3.64068	1.07217	-0.17367
H	4.11367	1.15942	-1.16107
H	4.06743	1.86980	0.44983
H	3.89054	0.10171	0.26326

C6B2

C	-1.11058	0.03237	0.04053
C	0.01472	0.91475	0.06594
C	-0.20972	2.28893	-0.10019
C	-1.49247	2.81609	-0.26742
C	-2.58919	1.96238	-0.25490
C	-2.39014	0.58801	-0.10611
H	0.63442	2.96950	-0.06939
H	-1.62374	3.88869	-0.38458
H	-3.59720	2.35059	-0.37638
H	-3.25206	-0.06999	-0.13179
N	-0.92528	-1.37175	0.19023
N	1.32901	0.39143	0.24813
C	1.56257	-0.14973	1.59101
H	2.46670	-0.76542	1.60009
H	1.68215	0.65112	2.34306
H	0.72231	-0.78324	1.87666
C	-2.08351	-2.12968	0.64309
H	-2.85579	-2.27100	-0.13553
H	-1.76169	-3.12238	0.97072
H	-2.54447	-1.63014	1.49990
C	2.43445	1.21861	-0.24289
H	2.64923	2.06405	0.43834
H	2.12043	1.65121	-1.19810
C	-0.21807	-2.02012	-0.93212
H	-0.92621	-2.21030	-1.75983
H	0.52968	-1.31415	-1.29389
C	3.72193	0.42320	-0.46794
H	4.46937	1.06558	-0.94831
H	4.16084	0.05774	0.46619
H	3.53902	-0.43754	-1.12101
C	0.48363	-3.32207	-0.54692
H	-0.21583	-4.10876	-0.24388
H	1.04584	-3.70329	-1.40790
H	1.18931	-3.15653	0.27456

C6B3

C	-0.73682	1.31159	0.84539
C	0.68044	1.19389	0.99945
C	1.20198	0.98998	2.28531

C	0.38195	0.87492	3.40879
C	-0.99763	0.94527	3.25347
C	-1.54028	1.16334	1.98558
H	2.27563	0.88824	2.40397
H	0.82467	0.70656	4.38701
H	-1.65908	0.84687	4.11036
H	-2.61636	1.24411	1.89353
N	-1.29831	1.56570	-0.44376
N	1.54039	1.31116	-0.13236
C	1.47648	0.17484	-1.08157
H	1.91733	0.50339	-2.02834
H	0.41784	-0.01301	-1.26916
C	-2.72059	1.25141	-0.63070
H	-3.36871	1.80714	0.06889
H	-2.98284	1.62198	-1.62697
C	2.88505	1.84590	0.11401
H	3.58123	1.07706	0.48971
H	2.78973	2.59742	0.90375
C	-0.95751	2.89620	-1.00149
H	-1.71731	3.63387	-0.68838
H	-0.01014	3.20462	-0.56114
C	3.49410	2.51658	-1.11966
H	4.44996	2.98100	-0.84935
H	3.69621	1.80941	-1.93108
H	2.83096	3.29752	-1.50832
C	-0.81715	2.90590	-2.52281
H	-1.73632	2.60054	-3.03593
H	-0.57673	3.92012	-2.86495
H	-0.01151	2.23752	-2.84312
C	-3.05855	-0.22812	-0.57595
C	-4.06082	-0.70603	0.27568
C	-2.41616	-1.13806	-1.42799
C	-4.40996	-2.05884	0.28651
H	-4.57624	-0.01287	0.93706
C	-2.75685	-2.49043	-1.41707
H	-1.64782	-0.77863	-2.10715
C	-3.75573	-2.95622	-0.55758
H	-5.18968	-2.40958	0.95791
H	-2.24633	-3.18079	-2.08380
H	-4.02206	-4.00996	-0.54892
C	2.16614	-1.10210	-0.62314
C	3.47256	-1.40197	-1.03596
C	1.51558	-2.00649	0.23098
C	4.11727	-2.56320	-0.60409
H	3.98767	-0.72412	-1.71362
C	2.15606	-3.16792	0.66521
H	0.49687	-1.80401	0.54932
C	3.46025	-3.44920	0.25114
H	5.12927	-2.77690	-0.93934
H	1.63270	-3.85759	1.32281
H	3.95757	-4.35558	0.58714

C7B1

C	0.88698	2.44747	0.10586
C	0.14895	1.27309	0.19112
C	0.81837	-0.00032	0.12995
C	2.24611	0.00029	-0.05086
C	2.95317	1.23054	-0.13450
C	2.28681	2.42899	-0.05302
H	0.36534	3.39513	0.20104
C	0.14992	-1.27427	0.19049
C	2.95358	-1.22932	-0.14040
H	4.03260	1.20136	-0.25895
H	2.83096	3.36821	-0.10932
C	2.28767	-2.42832	-0.06364

C	0.88824	-2.44794	0.09875
H	4.03287	-1.19920	-0.26589
H	2.83194	-3.36712	-0.12544
H	0.36723	-3.39612	0.19220
N	-1.23542	1.34889	0.42844
N	-1.23313	-1.35235	0.43534
C	-1.97518	2.04604	-0.33981
H	-1.56901	2.52876	-1.24288
C	-1.97679	-2.04382	-0.33428
H	-1.57527	-2.51917	-1.24332
C	-3.43284	2.24794	-0.06929
H	-3.65479	3.31755	0.04807
H	-4.03464	1.89453	-0.91748
H	-3.73495	1.71623	0.83669
C	-3.43303	-2.24809	-0.05803
H	-4.03905	-1.88485	-0.89906
H	-3.65508	-3.31878	0.04856
H	-3.73005	-1.72605	0.85523

C7B2

C	-1.80663	1.64271	-0.57538
C	-1.05833	0.58593	-0.05978
C	0.37748	0.76330	0.09566
C	0.88977	2.10824	-0.00722
C	0.06305	3.15863	-0.48757
C	-1.24453	2.90930	-0.82342
H	-2.87264	1.51770	-0.72265
C	1.34570	-0.29350	0.33892
C	2.23097	2.39728	0.36449
H	0.48967	4.15305	-0.59011
H	-1.87684	3.70297	-1.21394
C	3.06911	1.39263	0.77789
C	2.63678	0.05251	0.72672
H	2.57234	3.42828	0.31749
H	4.09014	1.61204	1.08042
H	3.35905	-0.73017	0.92883
N	-1.68732	-0.61926	0.30256
N	1.00083	-1.65421	0.16976
C	-2.95879	-1.00377	-0.30898
H	-2.96253	-2.10195	-0.37702
C	0.57109	-2.06986	-1.17492
H	-0.12929	-1.32519	-1.55294
C	-4.24987	-0.57173	0.40855
H	-4.31096	0.51270	0.54637
H	-5.11831	-0.88784	-0.18236
H	-4.34045	-1.03801	1.39547
C	1.70655	-2.26279	-2.18991
H	1.28880	-2.53641	-3.16677
H	2.28350	-1.33979	-2.31815
H	2.39894	-3.05919	-1.89417
C	-1.50320	-1.12140	1.65855
H	-1.57176	-2.21640	1.66731
H	-0.52409	-0.84099	2.04060
H	-2.26165	-0.72348	2.35258
C	1.80370	-2.65103	0.85943
H	1.91322	-2.37660	1.91340
H	1.27926	-3.61258	0.81115
H	2.81393	-2.80549	0.44346
H	0.00894	-3.00670	-1.06571
H	-2.96100	-0.63030	-1.33762

C7B3

C	-3.37297	-1.41815	-1.12014
C	-2.26711	-0.81056	-0.52602
C	-2.47949	0.42416	0.21870
C	-3.84577	0.73162	0.58072

C	-4.93099	0.02896	-0.00525
C	-4.69187	-0.98079	-0.90007
H	-3.24028	-2.30769	-1.71946
C	-1.46669	1.39398	0.62275
C	-4.13875	1.75237	1.52432
H	-5.94359	0.31980	0.26145
H	-5.51234	-1.50413	-1.38449
C	-3.12771	2.50562	2.05753
C	-1.81463	2.35609	1.57049
H	-5.17521	1.91570	1.80773
H	-3.33481	3.27655	2.79548
H	-1.08381	3.07942	1.90399
N	-0.98538	-1.41957	-0.62176
N	-0.17148	1.41665	0.02888
C	-0.80890	-2.47310	-1.63812
H	0.25176	-2.49328	-1.90487
C	-0.17445	1.50675	-1.45050
H	-0.71526	0.64565	-1.83836
C	-1.22318	-3.90682	-1.26136
H	-2.25522	-3.97966	-0.90597
H	-1.12248	-4.55370	-2.14174
H	-0.57237	-4.31570	-0.48214
C	-0.76888	2.79633	-2.03141
H	-0.70529	2.76736	-3.12623
H	-1.82429	2.90831	-1.76076
H	-0.23821	3.69435	-1.69551
C	-0.37377	-1.72312	0.69150
H	-0.35314	-0.79313	1.26340
H	-1.00713	-2.42873	1.25342
C	0.78805	2.34219	0.67012
H	0.75646	2.13497	1.74355
H	0.47936	3.39251	0.54067
C	1.03371	-2.29008	0.63654
C	1.38042	-3.34702	1.49022
C	2.02045	-1.77041	-0.21224
C	2.67429	-3.87184	1.49974
H	0.62632	-3.76459	2.15417
C	3.31071	-2.30368	-0.21792
H	1.77279	-0.94474	-0.87130
C	3.64378	-3.35521	0.63882
H	2.91945	-4.69064	2.17151
H	4.05802	-1.89004	-0.88996
H	4.64892	-3.76904	0.63354
C	2.23141	2.22112	0.21636
C	3.08675	1.28159	0.80678
C	2.76726	3.10587	-0.73059
C	4.43585	1.21898	0.45411
H	2.69361	0.59847	1.55436
C	4.11407	3.04323	-1.09255
H	2.12525	3.85881	-1.18181
C	4.95326	2.09812	-0.49960
H	5.08253	0.48626	0.92951
H	4.50848	3.73866	-1.82910
H	6.00464	2.05301	-0.77182
H	0.86254	1.39428	-1.77936
H	-1.33786	-2.14584	-2.53736

C8B1

C	0.77130	0.84353	-0.28370
C	1.52103	-0.33855	-0.08506
C	2.86737	-0.38355	-0.47910
C	3.48123	0.73197	-1.04663
C	2.75882	1.91280	-1.21796
C	1.41857	1.95903	-0.83212
C	-0.66345	0.94675	0.12219

C	-1.67882	0.25542	-0.57995
C	-3.01865	0.41718	-0.19196
C	-3.35935	1.24436	0.87775
C	-2.36337	1.92414	1.57823
C	-1.02957	1.76945	1.19502
H	3.42198	-1.30793	-0.34217
H	4.52426	0.67648	-1.34786
H	3.22917	2.78870	-1.65617
H	0.84640	2.87240	-0.97225
H	-3.79215	-0.09273	-0.75990
H	-4.40367	1.35773	1.15782
H	-2.61890	2.57272	2.41172
H	-0.24715	2.29465	1.73734
N	-1.34680	-0.46364	-1.74477
N	0.88903	-1.49311	0.42755
C	-1.54302	-1.71717	-1.86647
H	-1.28489	-2.14173	-2.84280
C	1.17459	-1.97703	1.57201
H	0.64589	-2.89764	1.84208
C	-2.05760	-2.70754	-0.86034
H	-3.00703	-3.13755	-1.20840
H	-1.34537	-3.53783	-0.77879
H	-2.20671	-2.26737	0.12708
C	2.11736	-1.45614	2.62039
H	2.55567	-0.49184	2.35553
H	1.58133	-1.35966	3.57357
H	2.92691	-2.17897	2.78985

C8B2

C	-0.42089	1.04660	-0.55683
C	-1.58073	0.28191	-0.23265
C	-2.73839	0.98285	0.16448
C	-2.78558	2.37494	0.19829
C	-1.67632	3.12189	-0.19231
C	-0.51389	2.44715	-0.56481
C	0.90746	0.43955	-0.88671
C	1.66166	-0.34532	0.03249
C	2.85965	-0.93011	-0.42061
C	3.33637	-0.72539	-1.71492
C	2.63351	0.09508	-2.59380
C	1.43354	0.66598	-2.16788
H	-3.63104	0.42582	0.42608
H	-3.70361	2.87002	0.50588
H	-1.70481	4.20799	-0.19166
H	0.37510	3.01583	-0.82715
H	3.44394	-1.53762	0.26242
H	4.26874	-1.19288	-2.02232
H	2.99912	0.27658	-3.60080
H	0.85452	1.27461	-2.85829
N	1.20776	-0.55809	1.35629
N	-1.56612	-1.12451	-0.26559
C	1.66312	-1.76097	2.03491
H	2.71342	-1.73757	2.37332
H	1.03727	-1.91771	2.92123
H	1.53999	-2.62626	1.37630
C	1.09297	0.61282	2.23952
H	0.45348	0.32086	3.08257
H	0.56174	1.40043	1.70295
C	2.42379	1.16640	2.76833
H	2.23852	2.04479	3.39903
H	2.96610	0.43283	3.37599
H	3.07802	1.47616	1.94513
C	-2.44166	-1.88663	0.62117
H	-1.88658	-2.78627	0.92690
H	-2.59540	-1.29543	1.52894

C	-3.79680	-2.33590	0.04925
H	-4.35899	-2.87927	0.81881
H	-4.41043	-1.49227	-0.28489
H	-3.67093	-3.01317	-0.80265
C	-1.19207	-1.82277	-1.48740
H	-0.80649	-2.81963	-1.23751
H	-2.04066	-1.95167	-2.18016
H	-0.40711	-1.28636	-2.01815

C8B3

C	-1.14710	1.55723	-0.40982
C	-1.59135	1.08020	0.84590
C	-2.60840	1.78506	1.51468
C	-3.17891	2.94087	0.98755
C	-2.73719	3.42003	-0.24452
C	-1.74208	2.72551	-0.92687
C	-0.19553	0.83689	-1.32855
C	1.22533	0.79996	-1.23420
C	1.93606	0.12549	-2.24732
C	1.29715	-0.50002	-3.31505
C	-0.08943	-0.45472	-3.41632
C	-0.80859	0.21978	-2.43125
H	-2.96596	1.41020	2.46825
H	-3.96223	3.45871	1.53527
H	-3.17094	4.31664	-0.67945
H	-1.41055	3.08396	-1.89786
H	3.01726	0.08508	-2.20335
H	1.89303	-1.01082	-4.06744
H	-0.60849	-0.93357	-4.24193
H	-1.89203	0.25902	-2.49589
N	1.92690	1.38328	-0.13966
N	-1.03069	-0.11659	1.42725
C	3.33033	1.01053	0.05633
H	3.97018	1.32114	-0.78760
H	3.67521	1.60567	0.91100
C	1.71404	2.82787	0.09585
H	2.10974	3.05273	1.09358
H	0.64263	3.01841	0.13723
C	2.35354	3.76133	-0.94001
H	2.11369	4.80396	-0.69799
H	3.44544	3.67093	-0.96425
H	1.97335	3.55449	-1.94694
C	-0.01039	0.14082	2.46841
H	0.47758	-0.82045	2.67051
H	0.74769	0.78506	2.01564
C	-0.47491	0.74481	3.80258
H	0.38445	0.81793	4.48103
H	-0.88350	1.75311	3.67943
H	-1.23102	0.12602	4.30032
C	-2.01599	-1.13190	1.83714
H	-1.43852	-1.92785	2.32535
H	-2.72918	-0.76675	2.59429
C	3.62745	-0.45073	0.36867
C	2.64411	-1.40381	0.65315
C	4.97308	-0.84695	0.40493
C	2.99958	-2.71765	0.97271
H	1.59800	-1.12509	0.61135
C	5.32993	-2.15510	0.72942
H	5.75095	-0.11949	0.17871
C	4.34048	-3.09900	1.01572
H	2.21984	-3.44538	1.18406
H	6.37937	-2.43803	0.75451
H	4.61344	-4.12137	1.26416
C	-2.79868	-1.73110	0.68370
C	-2.14141	-2.42525	-0.34198

C	-4.19475	-1.64282	0.64407
C	-2.86370	-3.01917	-1.37647
H	-1.05732	-2.49306	-0.32920
C	-4.92289	-2.23525	-0.39108
H	-4.71825	-1.10577	1.43204
C	-4.25841	-2.92663	-1.40416
H	-2.33748	-3.55515	-2.16240
H	-6.00697	-2.15426	-0.40458
H	-4.82112	-3.39063	-2.21030

C9B1

C	-4.25052	-0.82727	-0.45326
C	-3.05829	-1.55038	-0.49551
C	-1.90671	-1.12161	0.17530
C	-1.96899	0.09027	0.89898
C	-3.17541	0.80940	0.95310
C	-4.30882	0.35712	0.28185
H	-5.13001	-1.19526	-0.97476
H	-3.01723	-2.48236	-1.05634
H	-3.22184	1.71116	1.55764
H	-5.23537	0.92206	0.34338
C	-0.63503	-1.93714	0.09280
H	0.01272	-1.69440	0.93959
H	-0.89371	-3.00123	0.17133
C	0.16271	-1.75210	-1.22929
H	0.95758	-2.50567	-1.24382
H	-0.50565	-1.95719	-2.07469
C	0.78660	-0.38774	-1.40689
C	0.13191	0.62080	-2.12095
C	2.04504	-0.09377	-0.83345
C	0.69599	1.88760	-2.28642
H	-0.83913	0.40337	-2.55895
C	2.61984	1.17434	-1.01680
C	1.94888	2.16186	-1.73744
H	0.16861	2.64689	-2.85764
H	3.61485	1.36418	-0.62296
H	2.41240	3.13444	-1.88246
N	-0.83308	0.51540	1.62290
N	2.72870	-1.12456	-0.15358
C	-0.47482	1.73738	1.57068
C	3.31291	-0.88449	0.95335
H	3.25295	0.10535	1.43327
C	4.10779	-1.92974	1.67446
H	5.14873	-1.60268	1.80054
H	3.70472	-2.09324	2.68267
H	4.09122	-2.87124	1.12044
C	0.68018	2.26199	2.36684
H	1.10709	1.47005	2.98733
H	1.45298	2.65597	1.69407
H	0.36169	3.09338	3.00979
H	-0.99167	2.46152	0.92231

C9B2

C	-1.76134	-1.87558	-2.14338
C	-0.91456	-0.92930	-1.56847
C	-1.34960	-0.06658	-0.54931
C	-2.68855	-0.17538	-0.11219
C	-3.53588	-1.12877	-0.69665
C	-3.08224	-1.97711	-1.70520
H	-1.39233	-2.52564	-2.93265
H	0.11031	-0.84634	-1.92072
H	-4.56508	-1.20447	-0.35481
H	-3.75512	-2.70890	-2.14545
C	-0.39887	0.95455	0.03738
H	0.29426	1.29769	-0.73793
H	-0.97732	1.81858	0.37556

C	0.44472	0.46571	1.24986
H	0.92459	1.35011	1.67923
H	-0.23254	0.07449	2.01742
C	1.47986	-0.59078	0.92027
C	1.20082	-1.94019	1.17218
C	2.71878	-0.26282	0.31821
C	2.09291	-2.95686	0.82629
H	0.25143	-2.19837	1.63560
C	3.61130	-1.28879	-0.02593
C	3.30092	-2.62830	0.21538
H	1.83985	-3.99460	1.02721
H	4.56374	-1.03695	-0.48290
H	4.00811	-3.40584	-0.06282
N	-3.16955	0.69533	0.93850
N	3.03248	1.11964	0.07551
C	-4.36018	1.48592	0.59321
H	-5.24038	0.84599	0.39146
H	-4.61079	2.07471	1.48467
C	-3.34915	-0.01016	2.20778
H	-3.53206	0.71813	3.00715
H	-4.19302	-0.72544	2.20051
H	-2.44142	-0.56787	2.45580
C	-4.13505	2.43180	-0.58318
H	-3.31052	3.12338	-0.37706
H	-3.90193	1.88693	-1.50395
H	-5.04032	3.02298	-0.76524
C	3.77433	1.38870	-1.16352
H	4.85196	1.16148	-1.05605
H	3.38227	0.71474	-1.93207
C	3.61720	2.83268	-1.64324
H	4.11032	2.95609	-2.61484
H	2.55867	3.09061	-1.75868
H	4.07065	3.55372	-0.95460
C	3.63637	1.76905	1.24266
H	4.68141	1.44791	1.40948
H	3.62772	2.85678	1.12116
H	3.06640	1.52867	2.14352

C9B3

C	-2.92723	-2.61966	1.85945
C	-1.83718	-2.27654	1.06303
C	-1.94853	-1.37119	-0.00695
C	-3.22107	-0.82059	-0.27803
C	-4.31696	-1.18145	0.52256
C	-4.17895	-2.06534	1.59005
H	-2.80028	-3.32120	2.68009
H	-0.86911	-2.72284	1.27078
H	-5.29699	-0.76819	0.29849
H	-5.04246	-2.32764	2.19639
C	-0.72748	-1.04218	-0.84252
H	-0.19734	-1.97111	-1.08498
H	-1.06040	-0.60166	-1.78466
C	0.28689	-0.04396	-0.21257
H	1.00010	0.21637	-0.99930
H	-0.25059	0.87030	0.05581
C	1.05691	-0.53377	0.99417
C	0.60277	-0.21893	2.28565
C	2.24183	-1.29189	0.87250
C	1.27678	-0.64653	3.42836
H	-0.29797	0.38080	2.38743
C	2.91353	-1.72138	2.02730
C	2.43841	-1.40860	3.29992
H	0.89762	-0.38343	4.41267
H	3.82226	-2.30946	1.92547
H	2.97365	-1.75320	4.18138

N	-3.41142	0.07265	-1.40701
N	2.75220	-1.62804	-0.44245
C	-4.18758	-0.55175	-2.49922
H	-5.21054	-0.81363	-2.16854
H	-4.29905	0.20896	-3.28144
C	-3.99999	1.37882	-1.04222
H	-4.19152	1.90074	-1.98702
H	-4.97998	1.26995	-0.54563
C	-3.52453	-1.78616	-3.10392
H	-2.53683	-1.54575	-3.51172
H	-3.40746	-2.59028	-2.37056
H	-4.14581	-2.16994	-3.92198
C	2.63826	-3.07035	-0.74218
H	3.45224	-3.65026	-0.26817
H	1.70646	-3.42005	-0.28748
C	2.59657	-3.37232	-2.24057
H	3.52976	-3.10772	-2.75024
H	2.43281	-4.44507	-2.39970
H	1.78054	-2.82306	-2.72294
C	4.11291	-1.11248	-0.68721
H	4.82675	-1.45111	0.08379
H	4.45240	-1.54642	-1.63317
C	4.17581	0.39849	-0.80059
C	4.80073	1.17327	0.18250
C	3.62388	1.04731	-1.91504
C	4.87347	2.56345	0.06048
H	5.23161	0.68439	1.05306
C	3.69328	2.43417	-2.04135
H	3.13821	0.45532	-2.68737
C	4.31992	3.19751	-1.05189
H	5.36199	3.14856	0.83561
H	3.26374	2.92014	-2.91395
H	4.37705	4.27859	-1.15030
C	-3.10110	2.24773	-0.18623
C	-3.34814	2.42442	1.18014
C	-2.01404	2.92005	-0.76304
C	-2.52943	3.25036	1.95511
H	-4.18880	1.91208	1.64154
C	-1.19511	3.74776	0.00513
H	-1.81107	2.78997	-1.82364
C	-1.45083	3.91438	1.36913
H	-2.73745	3.37627	3.01469
H	-0.35757	4.26221	-0.45897
H	-0.81420	4.55949	1.96905

C10B1

C	-1.37120	1.05509	0.01093
C	-1.88205	2.36362	0.05151
C	-1.32194	3.31088	0.89566
C	-0.24160	2.96155	1.70930
C	0.29326	1.66904	1.69376
C	-0.27628	0.70049	0.83360
H	-2.72069	2.60796	-0.59233
H	-1.72010	4.32175	0.92477
H	0.19732	3.70502	2.37059
C	0.27626	-0.70064	0.83349
C	1.37121	-1.05507	0.01079
C	-0.29329	-1.66934	1.69346
C	1.88209	-2.36360	0.05116
C	0.24159	-2.96184	1.70879
C	1.32197	-3.31101	0.89513
H	2.72076	-2.60780	-0.59269
H	-0.19734	-3.70544	2.36993
H	1.72014	-4.32188	0.92408
C	1.46749	1.33323	2.58497

H	1.25660	0.47667	3.23580
H	2.35919	1.07162	2.00222
H	1.72561	2.18428	3.22300
C	-1.46754	-1.33370	2.58470
H	-1.25659	-0.47737	3.23582
H	-2.35918	-1.07182	2.00199
H	-1.72577	-2.18492	3.22246
C	-1.98773	0.06600	-0.89950
N	-2.99145	0.34738	-1.63555
C	-3.52830	-0.69908	-2.49133
H	-2.94861	-1.63549	-2.41811
H	-3.45379	-0.34751	-3.52981
C	-4.99954	-0.96062	-2.15949
H	-5.42436	-1.69813	-2.85028
H	-5.10626	-1.34539	-1.13860
H	-5.58295	-0.03658	-2.23608
C	1.98775	-0.06581	-0.89944
N	2.99145	-0.34708	-1.63556
C	3.52829	0.69950	-2.49120
H	2.94862	1.63592	-2.41780
H	3.45371	0.34810	-3.52973
C	4.99955	0.96095	-2.15938
H	5.42435	1.69858	-2.85006
H	5.10634	1.34552	-1.13843
H	5.58293	0.03691	-2.23618
H	-1.53158	-0.93313	-0.91224
H	1.53162	0.93333	-0.91199

C10B2

C	-1.80488	-0.39021	0.46441
C	-2.71061	-0.23319	1.52182
C	-2.44022	-0.77032	2.77659
C	-1.25948	-1.48097	2.98543
C	-0.33776	-1.66555	1.94887
C	-0.61013	-1.11388	0.67404
H	-3.62535	0.32171	1.33886
H	-3.14946	-0.63976	3.59036
H	-1.04640	-1.90389	3.96461
C	0.36158	-1.34224	-0.45499
C	1.41882	-0.43912	-0.70197
C	0.20647	-2.48372	-1.27744
C	2.29185	-0.67466	-1.77181
C	1.10366	-2.69495	-2.33050
C	2.13952	-1.79664	-2.58081
H	3.09296	0.03639	-1.94792
H	0.98394	-3.57372	-2.96016
H	2.82569	-1.97147	-3.40597
C	0.93310	-2.44177	2.21100
H	1.06028	-3.27335	1.50823
H	1.82422	-1.80954	2.11169
H	0.93280	-2.85547	3.22470
C	-0.90569	-3.48015	-1.03445
H	-0.84725	-3.92306	-0.03288
H	-1.89719	-3.01823	-1.11255
H	-0.86091	-4.29653	-1.76265
C	-2.16128	0.16146	-0.91219
H	-1.24145	0.37635	-1.48311
H	-2.67987	-0.62714	-1.47434
N	-3.05486	1.32402	-0.87555
C	-2.34167	2.53364	-0.44674
H	-1.63051	2.86990	-1.22894
H	-1.74214	2.26334	0.42868
C	-3.26585	3.68895	-0.06239
H	-2.67622	4.51404	0.35440
H	-3.82167	4.08709	-0.91813

H	-3.98989	3.36877	0.69534
C	1.58314	0.82579	0.13385
H	0.94280	1.60665	-0.29838
H	1.19809	0.65326	1.15418
N	2.95262	1.34774	0.15489
C	2.98389	2.76319	0.53844
H	2.68511	2.90295	1.59735
H	2.23147	3.27989	-0.06907
C	4.33959	3.42780	0.30022
H	4.25888	4.50768	0.47127
H	5.11788	3.05005	0.97172
H	4.66980	3.26676	-0.73244
C	-3.72504	1.50429	-2.15847
H	-4.44883	2.32206	-2.09791
H	-3.02650	1.72881	-2.98895
H	-4.27641	0.59385	-2.41728
C	3.81658	0.52606	0.99674
H	3.53984	0.57434	2.06893
H	3.75877	-0.51799	0.67603
H	4.85960	0.84116	0.90104

C10B3

C	0.41901	-1.56294	-1.66648
C	0.05199	-1.62973	-3.01586
C	-0.97878	-2.46666	-3.43377
C	-1.65075	-3.25506	-2.50210
C	-1.30057	-3.22325	-1.14714
C	-0.25517	-2.36971	-0.72253
H	0.59050	-1.01133	-3.72676
H	-1.25671	-2.50685	-4.48425
H	-2.45585	-3.91103	-2.82573
C	0.15404	-2.37879	0.72798
C	-0.48301	-1.54582	1.67473
C	1.16643	-3.27371	1.14845
C	-0.11095	-1.62530	3.02226
C	1.52196	-3.31848	2.50153
C	0.88763	-2.50221	3.43579
H	-0.61870	-0.98318	3.73483
H	2.30151	-4.00616	2.82180
H	1.16994	-2.55191	4.48469
C	-2.03660	-4.11069	-0.16757
H	-1.36108	-4.81145	0.33784
H	-2.53170	-3.53245	0.62161
H	-2.80482	-4.69918	-0.67968
C	1.85942	-4.19285	0.16664
H	1.15493	-4.88157	-0.31562
H	2.35357	-3.63922	-0.64037
H	2.62094	-4.79582	0.67187
C	1.58051	-0.66896	-1.23754
H	1.40629	-0.30564	-0.21249
H	2.48405	-1.29065	-1.17865
N	1.86266	0.42254	-2.17329
C	0.87503	1.50585	-2.08037
H	1.01919	2.11212	-1.16811
H	-0.10705	1.03215	-1.98379
C	0.84689	2.41613	-3.30848
H	0.02584	3.13679	-3.21588
H	1.77147	2.99085	-3.43265
H	0.68871	1.83236	-4.22242
C	-1.61286	-0.60830	1.25489
H	-1.44853	-0.27270	0.21898
H	-2.54470	-1.18925	1.23152
N	-1.82212	0.51194	2.17597
C	-0.78472	1.54324	2.04361
H	-0.91580	2.13826	1.12198

H	0.17085	1.01953	1.93904
C	-0.68800	2.47405	3.25269
H	0.16597	3.15072	3.13079
H	-1.58033	3.09668	3.38263
H	-0.54146	1.90022	4.17482
C	3.26850	0.86488	-2.14712
H	3.41639	1.54733	-2.99069
H	3.88373	-0.02017	-2.34809
C	-3.20546	1.02100	2.16969
H	-3.85725	0.17241	2.40931
H	-3.29949	1.73201	2.99715
C	-3.69724	1.68417	0.88631
C	-3.55960	3.06673	0.69191
C	-4.28856	0.92945	-0.13781
C	-3.98520	3.67509	-0.49062
H	-3.12535	3.67657	1.48107
C	-4.71633	1.53165	-1.32211
H	-4.42521	-0.14116	-0.00325
C	-4.56279	2.90768	-1.50368
H	-3.87081	4.74882	-0.61759
H	-5.17477	0.92682	-2.10049
H	-4.89802	3.37901	-2.42398
C	3.76137	1.54062	-0.87057
C	3.71132	2.93552	-0.73035
C	4.26588	0.78753	0.20023
C	4.13844	3.55882	0.44389
H	3.34477	3.54244	-1.55535
C	4.69386	1.40450	1.37680
H	4.33406	-0.29404	0.10930
C	4.62906	2.79371	1.50338
H	4.09330	4.64185	0.52817
H	5.08390	0.80033	2.19209
H	4.96532	3.27623	2.41749

C11B1

C	1.60343	1.90881	-1.10654
C	1.14622	1.25381	0.05067
C	2.08512	0.84028	1.00971
C	3.45287	1.06546	0.81497
C	3.90005	1.71209	-0.34285
C	2.97029	2.13421	-1.30178
H	0.88030	2.25850	-1.83455
H	1.74477	0.33597	1.90936
H	4.16495	0.73869	1.56674
H	4.96044	1.88980	-0.49450
H	3.30886	2.64390	-2.19906
C	-0.33821	0.98421	0.26298
H	-0.51008	0.75733	1.32304
C	-0.84867	-0.26391	-0.54732
H	-0.68467	-0.04156	-1.61361
C	-0.10880	-1.54253	-0.17968
C	0.90920	-2.03695	-1.01098
C	-0.42491	-2.24581	0.99632
C	1.60502	-3.20333	-0.67223
H	1.16006	-1.50500	-1.92392
C	0.26971	-3.41194	1.33513
H	-1.23037	-1.88661	1.62719
C	1.28838	-3.89387	0.50318
H	2.38964	-3.57143	-1.32632
H	0.01260	-3.94667	2.24477
H	1.82579	-4.79997	0.76604
N	-1.10970	2.15222	-0.21343
N	-2.28715	-0.42512	-0.24693
C	-1.93613	2.77259	0.55037
H	-2.44915	3.62283	0.09466

C	-3.13887	-0.35791	-1.20342
H	-2.82149	-0.18269	-2.24221
C	-2.29585	2.47438	1.98749
H	-3.06014	3.17222	2.34021
H	-2.68585	1.45444	2.09016
H	-1.42644	2.56436	2.65186
C	-4.61002	-0.51279	-0.97527
H	-5.14946	0.38523	-1.30566
H	-5.00612	-1.35407	-1.55996
H	-4.81866	-0.68573	0.08382

C11B2

C	2.72078	-0.30470	0.94610
C	1.77727	0.24724	0.06198
C	2.16217	0.44162	-1.27871
C	3.44546	0.09498	-1.71606
C	4.37217	-0.45564	-0.82240
C	4.00441	-0.65599	0.51252
H	2.44284	-0.46866	1.98389
H	1.45992	0.86166	-1.99128
H	3.72024	0.25234	-2.75494
H	5.36653	-0.72692	-1.16353
H	4.71252	-1.08615	1.21459
C	0.39547	0.67512	0.58975
H	0.33678	0.30743	1.62141
C	-0.74700	-0.01605	-0.22238
H	-0.54245	0.14614	-1.28724
C	-0.72188	-1.54083	-0.01170
C	-0.68702	-2.39498	-1.12845
C	-0.78874	-2.12798	1.26680
C	-0.71772	-3.78623	-0.98119
H	-0.62500	-1.96360	-2.12408
C	-0.82336	-3.51897	1.41866
H	-0.80759	-1.50299	2.15335
C	-0.78843	-4.35414	0.29570
H	-0.68297	-4.42310	-1.86015
H	-0.87418	-3.94958	2.41440
H	-0.81190	-5.43292	0.41528
N	0.22860	2.14439	0.71503
N	-2.08143	0.60073	-0.00404
C	1.11235	2.74673	1.72011
H	0.77219	3.75931	1.95573
H	1.07134	2.16150	2.64558
H	2.17067	2.80944	1.40509
C	-2.56899	0.66548	1.37759
H	-2.90995	-0.30878	1.77452
H	-1.78551	1.06446	2.02408
H	-3.41313	1.35912	1.43401
C	-3.10094	0.16649	-0.98126
H	-2.59621	0.05571	-1.94886
H	-3.51686	-0.82790	-0.72845
C	0.10255	2.93556	-0.52330
H	1.09351	3.18475	-0.94950
H	-0.42502	2.32347	-1.25771
C	-0.70305	4.22386	-0.31447
H	-0.84054	4.74169	-1.27215
H	-1.68851	3.98302	0.09735
H	-0.20499	4.92361	0.36617
C	-4.24903	1.17033	-1.13922
H	-3.85777	2.16689	-1.37250
H	-4.90943	0.85639	-1.95701
H	-4.86343	1.24642	-0.23552

C11B3

C	-2.59153	-1.49688	-0.93554
C	-1.72534	-1.42366	0.17075

C	-1.78592	-2.46018	1.12096	C	2.03819	1.14852	2.08807
C	-2.68214	-3.52495	0.97410	H	1.60889	0.57451	2.91618
C	-3.53967	-3.57908	-0.13036	H	3.13322	1.08529	2.19638
C	-3.48953	-2.55899	-1.08702	C	-2.10778	0.52828	2.33007
H	-2.56768	-0.71033	-1.68284	H	-3.12330	0.16928	2.08678
H	-1.12215	-2.45360	1.97910	H	-1.59375	-0.32031	2.79353
H	-2.70492	-4.31396	1.72003	C	-2.20535	1.64474	3.37725
H	-4.23337	-4.40628	-0.24564	H	-2.69648	1.25645	4.27792
H	-4.14621	-2.58863	-1.95144	H	-1.21129	2.00819	3.65942
C	-0.77465	-0.22272	0.31542	H	-2.79614	2.50072	3.03214
H	-0.63687	0.18679	-0.69104	C	1.62903	2.62437	2.23219
C	0.65334	-0.61718	0.85223	H	0.54458	2.73724	2.14987
H	0.53031	-0.87377	1.91007	H	1.94997	3.00663	3.21015
C	1.25769	-1.85255	0.16507	H	2.10408	3.25069	1.46812
C	2.05666	-2.72617	0.92733	C	-2.96353	2.06383	-0.50030
C	1.11201	-2.12121	-1.20728	C	-4.26200	1.98942	0.03844
C	2.69191	-3.82603	0.34294	C	-2.82645	2.11156	-1.89929
H	2.18195	-2.53725	1.99066	C	-5.38360	1.93982	-0.79643
C	1.75161	-3.21844	-1.79884	H	-4.39859	1.97816	1.11530
H	0.48287	-1.48849	-1.82424	C	-3.94664	2.06892	-2.73887
C	2.54389	-4.07479	-1.02781	H	-1.83275	2.19409	-2.33316
H	3.29809	-4.48790	0.95456	C	-5.22995	1.97659	-2.18834
H	1.62357	-3.40520	-2.86092	H	-6.37696	1.87948	-0.36178
H	3.03520	-4.92751	-1.48614	H	-3.81748	2.11193	-3.81636
N	-1.33680	0.89410	1.12763	H	-6.10138	1.94263	-2.83501
N	1.62467	0.49341	0.84065	C	3.51469	1.32430	-0.60020
C	-1.72567	2.11658	0.39367	C	3.99911	2.51158	-1.17304
H	-1.85364	2.91410	1.13364	C	4.43594	0.32560	-0.24079
H	-0.86837	2.40908	-0.22361	C	5.36966	2.69685	-1.39368
C	2.01157	1.11817	-0.42014	H	3.29785	3.29602	-1.44767
H	1.65952	0.47137	-1.23275	C	5.80591	0.50901	-0.45644
H	1.51505	2.09331	-0.57086	H	4.07013	-0.59122	0.20982

C	6.27823	1.69473	-1.03512
H	5.72562	3.62193	-1.83773
H	6.50506	-0.27278	-0.17401
H	7.34200	1.83644	-1.20067

C12B1

C	4.31081	0.07566	-0.17207
C	3.02635	-0.37790	-0.47233
C	2.12876	0.42651	-1.19019
C	2.56214	1.69733	-1.59487
C	3.84390	2.16021	-1.28788
C	4.72580	1.34773	-0.57554
H	4.99216	-0.56795	0.37961
H	2.69267	-1.35123	-0.12864
H	1.88900	2.33166	-2.16866
H	4.15395	3.14961	-1.61511
H	5.72784	1.69841	-0.34177
C	0.74188	-0.05591	-1.58332
H	0.43793	0.54454	-2.44914
H	0.81607	-1.08675	-1.94816
C	-0.43463	0.00245	-0.53288
C	-1.68565	0.49527	-1.35276
H	-1.45369	1.50560	-1.71501
H	-1.75533	-0.13973	-2.24539
C	-3.04203	0.52765	-0.67268
C	-3.49000	1.68085	-0.00868
C	-3.91243	-0.57209	-0.72748
C	-4.74252	1.72437	0.60511
H	-2.85424	2.56290	0.01480
C	-5.16591	-0.53521	-0.11585
H	-3.61092	-1.46560	-1.26841
C	-5.58436	0.61255	0.55905
H	-5.06282	2.63115	1.11172
H	-5.81950	-1.40179	-0.17496
H	-6.56160	0.64434	1.03329
C	-0.75438	-1.41001	0.04002
H	-1.27331	-1.28283	0.99689
H	-1.45131	-1.92595	-0.64183
C	-0.18916	1.02179	0.61302
H	-1.17055	1.27292	1.03400
H	0.23322	1.95259	0.19398
N	0.41490	-2.24383	0.28936
N	0.58889	0.50300	1.72485
C	0.33815	-3.47046	-0.03046
H	-0.56939	-3.88869	-0.50380
C	1.45342	-4.44403	0.21640
H	1.10884	-5.26608	0.85777
H	1.78884	-4.89785	-0.72562
H	2.29837	-3.94747	0.69958
C	1.57068	1.18775	2.14612
C	2.40892	0.74720	3.30934
H	3.45156	0.61474	2.99191
H	2.40962	1.50783	4.10154
H	2.03145	-0.19572	3.71239
H	1.86621	2.13687	1.66688

C12B2

C	5.02710	1.44357	0.11284
C	3.73655	1.54317	-0.41131
C	3.09557	0.43730	-0.99369
C	3.81360	-0.76836	-1.06154
C	5.10353	-0.87417	-0.54072
C	5.71466	0.23101	0.05547
H	5.49714	2.31779	0.55638
H	3.22505	2.50263	-0.38474
H	3.36033	-1.63380	-1.53782

H	5.63495	-1.82021	-0.60832
H	6.72070	0.15038	0.45875
C	1.70308	0.56687	-1.58545
H	1.56369	1.60803	-1.90332
H	1.65646	-0.03951	-2.49910
C	0.43427	0.18454	-0.71041
C	-0.73922	0.27808	-1.77050
H	-0.41784	1.01258	-2.51928
H	-0.76995	-0.68019	-2.30199
C	-2.15805	0.67149	-1.40009
C	-2.53216	2.02487	-1.38039
C	-3.15922	-0.28355	-1.17060
C	-3.84472	2.41294	-1.10484
H	-1.78723	2.78677	-1.59893
C	-4.47444	0.09638	-0.89905
H	-2.90479	-1.33740	-1.20751
C	-4.82239	1.44816	-0.85702
H	-4.10426	3.46875	-1.09549
H	-5.22995	-0.66625	-0.72599
H	-5.84682	1.74535	-0.64788
C	0.67282	-1.26863	-0.21022
H	1.07894	-1.83350	-1.06230
H	1.47694	-1.23352	0.53173
C	0.37218	1.24070	0.44401
H	-0.18315	2.12827	0.08647
H	1.39949	1.57628	0.59564
N	-0.08643	0.84415	1.78657
N	-0.46582	-2.02777	0.34910
C	-1.55063	0.80558	1.90494
H	-1.98970	1.78998	1.64995
H	-1.90916	0.09217	1.16233
C	-0.82349	-3.16429	-0.51198
H	-0.98865	-2.77747	-1.52369
H	0.01796	-3.88436	-0.58901
C	0.51822	1.72152	2.78744
H	1.60903	1.69210	2.69437
H	0.19665	2.77928	2.69858
H	0.26729	1.37990	3.79517
C	-0.18895	-2.46744	1.71701
H	0.59833	-3.24679	1.76648
H	0.13462	-1.60443	2.30053
H	-1.09277	-2.87391	2.18141
C	-2.06924	0.37838	3.27982
H	-1.91481	1.14123	4.05085
H	-3.14912	0.19976	3.21495
H	-1.59606	-0.54973	3.61874
C	-2.07676	-3.91765	-0.06514
H	-2.37573	-4.63242	-0.84103
H	-1.91460	-4.48681	0.85589
H	-2.91108	-3.22834	0.10710

C12B3

C	2.58770	3.98845	2.20903
C	1.87387	2.82397	2.50253
C	0.69405	2.49739	1.81504
C	0.24693	3.38371	0.82314
C	0.95499	4.54944	0.52720
C	2.13004	4.85758	1.21738
H	3.49641	4.21787	2.76033
H	2.23552	2.15839	3.28388
H	-0.65836	3.13457	0.27992
H	0.58566	5.22219	-0.24344
H	2.67978	5.76681	0.98775
C	-0.07427	1.24257	2.19383
H	0.28432	0.95610	3.18875

H	-1.12928	1.50593	2.32764	H	1.90352	0.10445	-2.90465
C	-0.02379	-0.05628	1.27290	C	-3.16012	1.51412	0.24480
C	-0.00270	-1.22569	2.33740	H	-3.15226	1.43989	1.33950
H	0.90703	-1.08372	2.93287	H	-3.22462	2.58812	0.02979
H	-0.84267	-1.04233	3.02122	C	0.76811	-2.29305	-2.66758
C	-0.04984	-2.68269	1.91585	H	1.46102	-1.96559	-3.45024
C	1.12496	-3.45126	1.88898	H	0.70464	-3.38601	-2.72824
C	-1.25975	-3.32969	1.61393	H	-0.22085	-1.88303	-2.90190
C	1.10098	-4.80297	1.54122	C	-1.91555	2.33141	-2.37806
H	2.07194	-2.98347	2.14780	H	-1.72654	2.22455	-3.45313
C	-1.28994	-4.68023	1.26296	H	-2.93358	2.72162	-2.26783
H	-2.19257	-2.77464	1.66831	H	-1.21632	3.07960	-1.98782
C	-0.10774	-5.42242	1.21985	C	-4.44629	0.86603	-0.27785
H	2.02687	-5.37258	1.52844	C	-5.57609	1.67226	-0.47899
H	-2.24058	-5.15580	1.03444	C	-4.56509	-0.50855	-0.53208
H	-0.13087	-6.47548	0.95184	C	-6.78946	1.12873	-0.90378
C	-1.35783	-0.17809	0.46346	H	-5.50370	2.74308	-0.29684
H	-1.26696	-1.01034	-0.24323	C	-5.77448	-1.05611	-0.96552
H	-2.11563	-0.48206	1.19410	H	-3.70788	-1.16056	-0.39820
C	1.29146	-0.07060	0.40503	C	-6.89289	-0.24128	-1.15018
H	2.04251	-0.69109	0.92364	H	-7.65041	1.77662	-1.04936
H	1.69585	0.94011	0.41649	H	-5.84117	-2.12375	-1.16042
N	1.19597	-0.44219	-1.01642	H	-7.83344	-0.66896	-1.48755
N	-1.86513	1.01984	-0.23150	C	3.63090	0.09271	-1.62642
C	1.21270	-1.88862	-1.26049	C	4.35915	0.91190	-0.75036
H	2.20344	-2.32834	-1.04349	C	4.31440	-0.94397	-2.28092
H	0.52315	-2.34301	-0.54756	C	5.71919	0.69431	-0.52259
C	-1.72615	0.97735	-1.69399	H	3.85922	1.73810	-0.25044
H	-0.71845	0.60652	-1.89190	C	5.67407	-1.16727	-2.05548
H	-2.42923	0.25378	-2.14695	H	3.78037	-1.57545	-2.98703
C	2.14009	0.32441	-1.85857	C	6.38107	-0.34975	-1.17175
H	1.91570	1.38543	-1.70312	H	6.26345	1.34602	0.15660

H	6.18233	-1.97520	-2.57621
H	7.44069	-0.51896	-0.99788

Al complexes, all ligands coordinating with AlMe

$A_1C_1B_1$

Al	-0.00987	0.17922	0.69363
N	1.37152	1.63374	0.17528
N	-1.16050	1.54974	-0.32990
O	1.23795	-1.05569	0.15865
O	-1.34835	-1.06633	0.37833
C	3.23326	-2.23811	-0.23979
C	4.59377	-2.24848	-0.49939
C	5.32272	-1.05050	-0.62643
C	4.65506	0.15222	-0.48973
C	3.26950	0.19589	-0.21336
C	2.52794	-1.02079	-0.08047
C	2.61794	1.47312	-0.12663
C	0.75842	2.95370	0.14759
C	-0.50229	2.80900	-0.70998
C	-2.42082	1.41214	-0.62135
C	-3.20534	0.23500	-0.41339
C	-4.58187	0.26523	-0.74605
C	-5.37576	-0.85574	-0.61155
C	-4.78971	-2.05170	-0.14554
C	-3.44692	-2.11790	0.17882
C	-2.61020	-0.97727	0.06611
C	-0.10956	0.50649	2.64815
H	5.10612	-3.20160	-0.60825
H	5.19276	1.09324	-0.59161
H	3.22879	2.35594	-0.34825
H	1.43087	3.72128	-0.25512
H	0.48118	3.23316	1.17196
H	-1.17320	3.66704	-0.58406
H	-0.21244	2.75219	-1.76761
H	-2.94025	2.25902	-1.08340
H	-5.00527	1.19808	-1.11407
H	-5.40564	-2.94186	-0.03965
H	-0.15269	-0.45045	3.18960
H	0.74879	1.05832	3.05868
H	-1.01295	1.06070	2.94592
H	-6.43102	-0.82244	-0.86413
H	-2.99685	-3.03966	0.53495
H	2.67068	-3.16205	-0.14552
H	6.38847	-1.07278	-0.83218

$A_1C_1B_2$

Al	-0.08727	-0.13095	0.72120
C	0.32808	0.12558	2.63319
O	-1.55659	-1.18867	0.71937
C	-2.79281	-1.07626	0.24955
C	-3.81112	-1.93251	0.71353
C	-5.11420	-1.80732	0.24059
C	-5.44277	-0.82411	-0.69908
C	-4.44085	0.02848	-1.16248
C	-3.12232	-0.09069	-0.71131
C	-2.02853	0.76866	-1.28087
N	-1.12060	1.41854	-0.25379
C	-0.24904	2.38047	-0.99582
C	0.96743	2.83392	-0.19847
N	1.79308	1.69300	0.21676
C	2.60314	1.17326	-0.91439
C	3.16722	-0.19735	-0.64934
C	4.54137	-0.45302	-0.67991
C	5.04358	-1.74048	-0.48151
C	4.15135	-2.78923	-0.24467
C	2.77778	-2.55376	-0.20544
C	2.26876	-1.25955	-0.40307

O	0.93981	-1.05232	-0.39893
C	-1.93178	2.15408	0.75592
C	2.67340	2.07040	1.33032
H	-0.26690	-0.57977	3.22849
H	0.10108	1.12898	3.01974
H	1.38247	-0.06909	2.86730
H	-5.88346	-2.48016	0.61284
H	-4.67957	0.79635	-1.89671
H	-2.46367	1.56560	-1.89843
H	-1.36265	0.17777	-1.92307
H	-0.84853	3.25297	-1.28942
H	0.06523	1.88207	-1.91584
H	1.53539	3.55746	-0.80957
H	0.65114	3.36942	0.70185
H	3.41369	1.88275	-1.15342
H	1.95292	1.12249	-1.79345
H	5.22567	0.37223	-0.86982
H	4.52583	-3.79817	-0.08823
H	-2.57494	2.89363	0.26176
H	-1.27113	2.66312	1.45872
H	-2.55867	1.45509	1.30993
H	3.35399	2.89425	1.05292
H	3.27821	1.21112	1.62725
H	2.07558	2.38438	2.18912
H	-6.46133	-0.72340	-1.06271
H	-3.54964	-2.68945	1.44767
H	2.07413	-3.36201	-0.02646
H	6.11444	-1.92164	-0.51086

$A_1C_1B_3$

Al	-0.44830	-1.38556	0.81354
C	0.29055	-1.05214	2.60766
O	-2.04387	-2.14650	1.10772
C	-3.24672	-2.01940	0.55441
C	-4.33464	-2.74971	1.07061
C	-5.60371	-2.62092	0.51763
C	-5.82726	-1.75642	-0.55960
C	-4.75727	-1.02394	-1.06986
C	-3.46963	-1.13945	-0.52962
C	-2.31400	-0.42246	-1.17800
N	-1.29872	0.21828	-0.24562
C	-0.34027	0.95958	-1.12580
C	0.98105	1.35449	-0.46866
N	1.76787	0.19487	-0.04998
C	2.36020	-0.54371	-1.19048
C	2.65935	-1.98589	-0.86542
C	3.94788	-2.51630	-0.98341
C	4.20667	-3.86406	-0.72875
C	3.15279	-4.69838	-0.35125
C	1.85890	-4.19252	-0.23585
C	1.59611	-2.83799	-0.49157
O	0.33125	-2.37278	-0.42812
C	-1.94518	1.12778	0.78342
C	2.76368	0.51350	1.00201
H	-0.30558	-1.61319	3.33805
H	0.27099	-0.00164	2.92779
H	1.32869	-1.39094	2.70363
H	-6.42763	-3.19817	0.93028
H	-4.91540	-0.35394	-1.91369
H	-2.69306	0.35107	-1.85732
H	-1.72332	-1.12736	-1.77693
H	-0.83460	1.85807	-1.51282
H	-0.14590	0.31069	-1.98152
H	1.51921	2.00916	-1.17699
H	0.78687	1.96399	0.42012

H	3.26524	-0.03741	-1.56015
H	1.63538	-0.51920	-2.00933
H	4.75861	-1.86132	-1.29761
H	3.33617	-5.75082	-0.14886
H	-1.15571	1.39411	1.49026
H	-2.66195	0.50576	1.32270
H	3.21079	-0.43777	1.30476
H	2.20542	0.89190	1.86378
H	-6.81748	-1.65620	-0.99407
H	-4.14733	-3.41739	1.90620
H	1.02696	-4.83327	0.04187
H	5.21499	-4.25625	-0.82651
C	-2.62225	2.38673	0.27457
C	-1.93022	3.60723	0.24711
C	-3.96709	2.37551	-0.12436
C	-2.55130	4.77759	-0.18992
H	-0.89762	3.64750	0.58657
C	-4.59120	3.54449	-0.56197
H	-4.53026	1.44837	-0.07960
C	-3.88473	4.74765	-0.60085
H	-1.99678	5.71206	-0.20021
H	-5.63490	3.51557	-0.86324
H	-4.37282	5.65799	-0.93812
C	3.86446	1.50013	0.63510
C	3.66864	2.88381	0.75857
C	5.11151	1.04850	0.17914
C	4.67395	3.78801	0.41425
H	2.72424	3.25945	1.14647
C	6.12167	1.94802	-0.16608
H	5.29647	-0.02060	0.11243
C	5.90356	3.32188	-0.05484
H	4.50130	4.85572	0.52276
H	7.08135	1.57469	-0.51395
H	6.68946	4.02412	-0.31967

$A_1C_2B_1$

Al	0.04985	0.00132	0.60796
C	0.31609	0.20310	2.57249
O	-1.16577	-1.15798	-0.09612
C	-2.47655	-1.16926	-0.21550
C	-3.11956	-2.36689	-0.60489
C	-4.49794	-2.42834	-0.73854
C	-5.30363	-1.30085	-0.49716
C	-4.69571	-0.11510	-0.12479
C	-3.29365	-0.02150	0.02012
C	-2.71769	1.25126	0.37093
N	-1.45436	1.47216	0.52625
C	-0.99177	2.82579	0.82432
C	-0.23156	3.41869	-0.38509
C	0.32111	2.34639	-1.33394
N	1.04448	1.26280	-0.64736
C	2.32434	1.16543	-0.88598
C	3.19837	0.11691	-0.46245
C	4.58730	0.23773	-0.71697
C	5.46133	-0.78509	-0.41121
C	4.94667	-1.97935	0.13914
C	3.59510	-2.13834	0.38070
C	2.67521	-1.09162	0.10427
O	1.40602	-1.25635	0.33099
H	-0.46183	0.75701	3.11596
H	1.27865	0.67616	2.82305
H	0.35989	-0.80061	3.02139
H	-4.96177	-3.36662	-1.03380
H	-5.29620	0.77342	0.06156
H	-3.42037	2.08314	0.50187

H	-0.31767	2.75265	1.68364
H	-1.82763	3.47573	1.11283
H	-0.50880	1.88325	-1.87824
H	0.97270	2.81859	-2.07880
H	2.78439	1.94446	-1.50308
H	4.95574	1.16114	-1.16018
H	5.62759	-2.79401	0.37527
H	-6.38247	-1.36057	-0.60340
H	-2.49634	-3.23698	-0.78836
H	3.20010	-3.05967	0.79821
H	6.52547	-0.68082	-0.59921
H	-0.90039	4.05730	-0.97492
H	0.58311	4.05547	-0.02127

$A_1C_3B_1$

Al	0.07834	-0.42889	0.62155
C	0.34902	-0.37273	2.59529
O	-1.12957	-1.54743	-0.15967
C	-2.44298	-1.56838	-0.24790
C	-3.08071	-2.74361	-0.70788
C	-4.46121	-2.81544	-0.81110
C	-5.27476	-1.72063	-0.46743
C	-4.67248	-0.55629	-0.02481
C	-3.26846	-0.45246	0.09067
C	-2.69965	0.80091	0.51587
N	-1.43447	1.02683	0.65070
C	-0.97156	2.35782	1.02711
C	-0.28432	3.08345	-0.16851
C	0.30097	2.04372	-1.15754
N	1.04907	0.93992	-0.53524
C	2.32880	0.87720	-0.78776
C	3.21944	-0.18628	-0.44236
C	4.60573	-0.02676	-0.68980
C	5.49532	-1.05687	-0.46356
C	4.99942	-2.29672	-0.00454
C	3.65079	-2.49338	0.22557
C	2.71519	-1.44224	0.03078
O	1.44858	-1.64333	0.24363
C	-1.31878	3.91789	-0.94536
C	0.82216	3.99986	0.37863
H	-0.43129	0.13403	3.17985
H	1.30876	0.08749	2.87838
H	0.40110	-1.40665	2.96841
H	-4.92044	-3.73643	-1.16261
H	-5.27916	0.30763	0.24060
H	-3.40759	1.61089	0.72780
H	-0.24711	2.22710	1.83805
H	-1.79556	2.97193	1.41502
H	-0.52103	1.59067	-1.72234
H	0.94081	2.56489	-1.88091
H	2.77341	1.70134	-1.35499
H	4.95988	0.93261	-1.06266
H	5.69269	-3.11686	0.16820
H	-2.17115	3.30546	-1.26441
H	-0.87517	4.36160	-1.84530
H	-1.70331	4.73714	-0.32663
H	1.62335	3.42204	0.85420
H	0.42103	4.68969	1.13146
H	1.26577	4.60534	-0.42122
H	-6.35517	-1.78834	-0.55031
H	-2.45146	-3.58890	-0.96978
H	3.27015	-3.44958	0.57187
H	6.55729	-0.92299	-0.64486

$A_1C_5B_2$

Al	0.01504	-0.55515	0.96577

C	0.45741	-0.11483	2.84103
O	-1.47290	-1.56882	1.17743
C	-2.57741	-1.76021	0.46767
C	-3.52793	-2.71527	0.87925
C	-4.71650	-2.88308	0.17694
C	-5.00044	-2.09905	-0.94677
C	-4.06791	-1.14875	-1.35819
C	-2.85853	-0.97588	-0.67611
C	-1.82309	-0.01944	-1.20265
N	-1.12458	0.84829	-0.16183
C	-0.33269	1.89362	-0.92724
C	0.98763	2.32450	-0.23569
N	1.75188	1.11843	0.18344
C	2.42588	0.52402	-1.01317
C	3.05793	-0.81345	-0.74895
C	4.41562	-1.05508	-0.97827
C	4.96327	-2.32346	-0.78449
C	4.13544	-3.36282	-0.35058
C	2.78123	-3.13923	-0.11123
C	2.22710	-1.86315	-0.30381
O	0.91725	-1.65679	-0.10238
C	-2.13106	1.42037	0.77821
C	2.78579	1.40561	1.19526
H	-0.17237	-0.73384	3.49098
H	0.27481	0.93110	3.12559
H	1.50048	-0.33250	3.10196
H	-5.43378	-3.62863	0.51237
H	-4.27398	-0.53261	-2.23249
H	-2.28922	0.63987	-1.94572
H	-1.01433	-0.56622	-1.70437
H	-0.04034	1.35916	-1.83377
H	1.56389	2.83690	-1.02995
H	3.17967	1.23325	-1.39544
H	1.66901	0.40562	-1.79442
H	5.04758	-0.23721	-1.32083
H	4.54833	-4.35672	-0.19537
H	-2.90866	1.97545	0.24405
H	-1.63426	2.07731	1.48991
H	-2.59954	0.60733	1.32852
H	3.50599	2.16299	0.84235
H	3.33330	0.48458	1.39908
H	2.33296	1.74355	2.12529
H	-5.93250	-2.22492	-1.48979
H	-3.30265	-3.31040	1.75925
H	2.12651	-3.93855	0.22292
H	6.01929	-2.49841	-0.96908
C	-1.12411	3.13616	-1.40865
H	-0.57921	3.51765	-2.28303
H	-2.10819	2.83560	-1.78669
C	-1.26166	4.28730	-0.39638
H	-1.65772	5.16803	-0.91695
H	-1.99370	4.04003	0.37878
C	0.77858	3.39279	0.85384
H	0.20866	2.99319	1.70035
H	1.74761	3.70378	1.25361
C	0.08114	4.62858	0.26028
H	0.74813	5.08074	-0.48825
H	-0.06139	5.38319	1.04282

$A_1C_8B_1$

Al	0.33796	-0.95566	-0.64948
C	0.64297	-0.81900	-2.61311
O	-0.69613	-2.22977	0.12668
C	-1.98738	-2.46583	0.19876
C	-2.43265	-3.71351	0.69222

C	-3.78470	-4.00645	0.77349
C	-4.75911	-3.07252	0.37411
C	-4.34671	-1.84168	-0.10142
C	-2.97445	-1.51270	-0.19540
C	-2.61694	-0.20273	-0.66155
N	-1.40682	0.26047	-0.76208
C	-1.25177	1.58881	-1.26475
C	-1.57738	1.89229	-2.59095
C	-1.40685	3.18747	-3.07930
C	-0.91355	4.18735	-2.24075
C	-0.60270	3.88791	-0.91479
C	-0.75939	2.59040	-0.40547
C	-0.53514	2.30967	1.03705
C	-1.19047	3.10442	1.99183
C	-1.03437	2.88841	3.35989
C	-0.20892	1.85670	3.80423
C	0.46179	1.06340	2.87507
C	0.31318	1.28302	1.49881
N	1.08860	0.46313	0.60600
C	2.39048	0.62782	0.71440
C	3.40775	-0.22864	0.20332
C	4.76248	0.18730	0.27898
C	5.78815	-0.66503	-0.06875
C	5.47540	-1.98390	-0.47080
C	4.16961	-2.43056	-0.53051
C	3.08832	-1.56242	-0.21973
O	1.86169	-1.98467	-0.27552
H	-0.27726	-0.81791	-3.21346
H	1.24517	-1.67164	-2.95579
H	1.19911	0.08547	-2.90231
H	-1.67988	-4.43448	0.99614
H	-4.09575	-4.97751	1.15149
H	-5.81518	-3.31509	0.44094
H	-5.07909	-1.09823	-0.40987
H	-3.44866	0.45355	-0.93595
H	-1.94987	1.10308	-3.23647
H	-1.65541	3.40979	-4.11331
H	-0.77198	5.19729	-2.61518
H	-0.22809	4.66705	-0.25650
H	-1.84665	3.89688	1.64286
H	-1.56369	3.51637	4.07103
H	-0.08388	1.66619	4.86646
H	1.10624	0.25426	3.20620
H	2.73545	1.48729	1.29447
H	4.97746	1.19888	0.61735
H	6.82202	-0.33727	-0.02228
H	6.28182	-2.66418	-0.73516
H	3.93177	-3.44582	-0.83338

$A_1C_9B_1$

Al	-0.68098	0.04317	-1.23442
C	-0.43817	-1.16904	-2.78856
O	-2.53757	0.14594	-1.51195
C	-3.58550	-0.22316	-0.84598
C	-4.86900	-0.21937	-1.45781
C	-5.98903	-0.61260	-0.75211
C	-5.90752	-1.01669	0.60052
C	-4.67861	-1.01185	1.22419
C	-3.50387	-0.62865	0.52554
C	-2.27060	-0.57785	1.23475
N	-1.07216	-0.32298	0.75766
C	-0.05731	-0.18386	1.78350
C	0.09570	1.06991	2.38270
C	1.05932	1.27054	3.37140
C	1.87518	0.20984	3.76140

C	1.70641	-1.04337	3.17179
C	0.73908	-1.27357	2.18384
C	0.54074	-2.67050	1.63561
C	0.65179	-2.84540	0.10544
C	1.98188	-2.48469	-0.51856
C	2.90694	-3.51951	-0.71943
C	4.13939	-3.31779	-1.33671
C	4.46170	-2.04469	-1.80164
C	3.56562	-0.99745	-1.60993
C	2.34098	-1.18164	-0.93763
N	1.49469	-0.03682	-0.74626
C	2.12733	1.05889	-0.41980
C	1.61411	2.40094	-0.40607
C	2.46190	3.42956	0.07079
C	2.07324	4.75595	0.04314
C	0.81200	5.08355	-0.48899
C	-0.03733	4.10131	-0.97278
C	0.33359	2.73760	-0.93451
O	-0.49834	1.83274	-1.39715
H	-1.05099	-2.07960	-2.72158
H	0.58425	-1.47972	-3.02963
H	-0.82424	-0.62478	-3.66449
H	-6.95711	-0.60805	-1.24792
H	-4.58985	-1.30520	2.26849
H	-2.34134	-0.75430	2.31115
H	-0.54058	1.88809	2.05895
H	1.16962	2.25103	3.82610
H	2.63427	0.35140	4.52600
H	2.33263	-1.87370	3.48952
H	-0.45391	-3.03622	1.92862
H	1.26341	-3.33696	2.12016
H	0.45756	-3.90295	-0.10835
H	-0.15376	-2.30081	-0.38790
H	2.63454	-4.51980	-0.38989
H	4.82210	-4.15078	-1.47969
H	5.39460	-1.86522	-2.32911
H	3.79644	-0.01967	-2.02114
H	3.18126	0.97807	-0.14172
H	3.43878	3.15379	0.46288
H	0.49710	6.12379	-0.52451
H	2.73300	5.53367	0.41546
H	-1.00940	4.34971	-1.38798
H	-6.80170	-1.31749	1.13750
H	-4.93425	0.09530	-2.49499

$A_1C_{11}B_1$

Al	-1.36693	-0.58827	0.89663
N	0.52827	-1.22593	0.33096
N	-0.64681	1.00100	-0.15181
O	-1.99799	-2.18057	0.24179
O	-3.06911	0.14314	0.75281
C	-2.27120	-4.48023	-0.18285
C	-1.72806	-5.73244	-0.42132
C	-0.33499	-5.92308	-0.48391
C	0.49511	-4.83104	-0.30749
C	-0.02832	-3.54287	-0.05457
C	-1.44273	-3.35080	0.01810
C	0.88728	-2.43936	0.06717
C	1.47875	-0.10520	0.40650
C	0.82077	1.05841	-0.39654
C	-1.37041	1.91592	-0.73719
C	-2.79077	2.04444	-0.67592
C	-3.40909	3.10685	-1.38212
C	-4.77679	3.28411	-1.35199
C	-5.56385	2.38490	-0.59994

C	-4.99369	1.33763	0.09956
C	-3.58945	1.12972	0.08396
C	-1.05942	-0.48537	2.85513
H	-2.39277	-6.58163	-0.56229
H	1.57566	-4.94870	-0.36191
H	1.94362	-2.66750	-0.09506
H	1.49123	0.20208	1.45990
H	0.98572	0.85867	-1.46196
H	-0.85455	2.67284	-1.33513
H	-2.77813	3.78664	-1.95170
H	-6.64291	2.51800	-0.56989
H	-1.93767	-0.88718	3.38261
H	-0.19054	-1.05254	3.21905
H	-0.94271	0.54598	3.22198
H	-5.24307	4.10031	-1.89505
H	-5.60029	0.64596	0.67634
H	-3.34585	-4.33190	-0.13476
H	0.07883	-6.90910	-0.67186
C	1.44099	2.40456	-0.05434
C	2.24331	3.07102	-0.98765
C	1.23556	2.99235	1.20333
C	2.83591	4.29648	-0.67331
H	2.41015	2.62623	-1.96572
C	1.82445	4.21624	1.51744
H	0.60248	2.49616	1.93450
C	2.62812	4.87157	0.58021
H	3.45639	4.79969	-1.41013
H	1.65415	4.66031	2.49465
H	3.08585	5.82596	0.82638
C	2.91004	-0.37996	-0.02063
C	3.93922	-0.32508	0.92770
C	3.23897	-0.69250	-1.34956
C	5.26508	-0.56397	0.56148
H	3.69991	-0.08631	1.96110
C	4.56245	-0.93554	-1.71751
H	2.45751	-0.75574	-2.10295
C	5.58019	-0.86908	-0.76297
H	6.04941	-0.51063	1.31173
H	4.79832	-1.17649	-2.75069
H	6.61134	-1.05571	-1.05073

$A_2C_1B_3$

Al	-0.39230	-1.07297	0.67004
C	0.25607	-0.86931	2.52269
O	-2.04102	-1.77260	0.89700
C	-3.22861	-1.57644	0.32878
C	-4.35147	-2.29362	0.81516
C	-5.59657	-2.07489	0.22568
C	-5.76437	-1.16515	-0.82382
C	-4.65935	-0.45824	-1.29027
C	-3.39107	-0.65307	-0.72660
C	-2.19230	0.03750	-1.32499
N	-1.16242	0.58708	-0.35374
C	-0.14935	1.31267	-1.18473
C	1.17564	1.57291	-0.47718
N	1.83765	0.32756	-0.06970
C	2.41778	-0.40022	-1.22689
C	2.66676	-1.86278	-0.95269
C	3.93722	-2.42908	-1.10509
C	4.14095	-3.79422	-0.90968
C	3.05732	-4.60161	-0.55840
C	1.77161	-4.07465	-0.40240
C	1.57750	-2.68897	-0.60352
O	0.33211	-2.18090	-0.51036
C	-1.77983	1.49112	0.70097

C	2.82896	0.54055	1.01929
H	-0.41372	-1.43262	3.18625
H	0.27167	0.16146	2.90260
H	1.26588	-1.27261	2.66834
H	-6.45586	-2.62916	0.59849
H	-4.77138	0.24865	-2.11054
H	-2.52389	0.85691	-1.97348
H	-1.62999	-0.66596	-1.95208
H	-0.57453	2.26254	-1.52557
H	0.01640	0.70493	-2.07600
H	1.79902	2.18731	-1.14777
H	1.00667	2.17269	0.42249
H	3.34360	0.08618	-1.56853
H	1.70360	-0.32138	-2.05150
H	4.76875	-1.79096	-1.39716
H	3.20776	-5.66828	-0.40364
H	-0.99232	1.68541	1.43238
H	-2.54309	0.89183	1.20029
H	3.21243	-0.44673	1.29004
H	2.27233	0.91654	1.88191
C	-2.37363	2.80696	0.23367
C	-3.71182	2.89475	-0.17813
C	-1.60634	3.98204	0.25974
C	-4.25806	4.11645	-0.57487
H	-4.33176	2.00335	-0.17642
C	-2.14935	5.20489	-0.13632
H	-0.57640	3.94202	0.60711
C	-3.47791	5.27402	-0.55947
H	-5.29790	4.16400	-0.88731
H	-1.53794	6.10289	-0.10618
H	-3.90497	6.22549	-0.86495
C	3.99516	1.47629	0.72662
C	5.22363	0.97741	0.26782
C	3.88153	2.85968	0.93476
C	6.29485	1.83248	0.00124
H	5.34641	-0.09437	0.13623
C	4.94843	3.71930	0.66930
H	2.95255	3.26879	1.32559
C	6.15852	3.20786	0.19591
H	7.23811	1.42230	-0.35037
H	4.83824	4.78687	0.84250
H	6.99197	3.87502	-0.00774
H	5.13082	-4.22575	-1.03130
H	-6.74385	-1.01197	-1.26813
C	-4.16514	-3.27158	1.94549
H	-3.43510	-4.04852	1.68680
H	-3.78043	-2.77667	2.84635
H	-5.11053	-3.76133	2.20214
C	0.59840	-4.94710	-0.03709
H	0.14772	-4.64264	0.91615
H	-0.19848	-4.88201	-0.78779
H	0.90189	-5.99570	0.05152

$A_2C_{11}B_1$

Al	1.16112	0.13227	0.84199
N	-0.47762	1.29122	0.32130
N	-0.00263	-1.17511	-0.19119
O	2.20880	1.48780	0.18479
O	2.58029	-1.05405	0.65048
C	3.14843	3.60854	-0.22512
C	2.95539	4.96831	-0.43657
C	1.67558	5.55320	-0.46416
C	0.56852	4.74872	-0.28035
C	0.71307	3.36066	-0.05349
C	2.01300	2.77359	-0.01610

C	-0.47609	2.56107	0.07747
C	-1.70968	0.49135	0.40999
C	-1.42833	-0.80620	-0.40821
C	0.41710	-2.25566	-0.79195
C	1.74017	-2.78996	-0.76232
C	2.00523	-3.98366	-1.48093
C	3.26504	-4.54037	-1.47523
C	4.29195	-3.90573	-0.74271
C	4.08641	-2.73765	-0.02514
C	2.78416	-2.14837	-0.02514
C	0.88285	0.09983	2.80790
H	3.82902	5.60001	-0.58551
H	-0.43394	5.17110	-0.30700
H	-1.42569	3.08415	-0.05903
H	-1.79312	0.19250	1.46252
H	-1.54710	-0.55838	-1.46966
H	-0.30637	-2.82724	-1.38055
H	1.19327	-4.45193	-2.03360
H	5.28619	-4.34863	-0.73955
H	1.85211	0.21007	3.31715
H	0.23404	0.89823	3.19629
H	0.46642	-0.85252	3.17069
H	3.47240	-5.45447	-2.02331
H	1.56560	6.62032	-0.63260
C	-2.40374	-1.91960	-0.05741
C	-3.38103	-2.32031	-0.97576
C	-2.35272	-2.55147	1.19465
C	-4.29490	-3.32620	-0.65203
H	-3.43184	-1.83894	-1.94935
C	-3.26255	-3.55693	1.51810
H	-1.58971	-2.26410	1.91342
C	-4.23794	-3.94652	0.59583
H	-5.04729	-3.62453	-1.37727
H	-3.20848	-4.03904	2.49059
H	-4.94560	-4.73146	0.84894
C	-3.00897	1.16914	0.01032
C	-3.99928	1.39159	0.97545
C	-3.25344	1.58445	-1.30884
C	-5.20766	2.00258	0.63544
H	-3.82483	1.07618	2.00133
C	-4.45827	2.19908	-1.65043
H	-2.49621	1.43756	-2.07514
C	-5.44042	2.40764	-0.67918
H	-5.96512	2.16048	1.39843
H	-4.62950	2.51501	-2.67610
H	-6.37970	2.88433	-0.94647
C	5.18547	-2.06630	0.75383
H	5.34898	-1.03853	0.40735
H	4.93320	-1.99491	1.81914
H	6.12726	-2.61688	0.65969
C	4.52114	2.99021	-0.20002
H	4.72051	2.49989	0.76063
H	4.62347	2.21361	-0.96802
H	5.29566	3.74561	-0.36907

$A_3C_1B_1$

Al	-0.00675	-1.12140	0.74678
N	-1.35104	-2.65848	0.44630
N	1.14291	-2.54007	-0.18066
O	-1.31362	-0.00371	0.09341
O	1.31432	0.11388	0.30057
C	-3.39916	1.11828	-0.25107
C	-4.78227	0.98544	-0.33264
C	-5.49483	-0.23246	-0.21768
C	-4.73508	-1.36832	-0.01631

C	-3.32700	-1.30727	0.09750
C	-2.62795	-0.06630	-0.00454
C	-2.62255	-2.55059	0.24382
C	-0.70335	-3.96158	0.46118
C	0.50077	-3.82820	-0.47558
C	2.41147	-2.41440	-0.43587
C	3.20034	-1.23290	-0.26536
C	4.58717	-1.33987	-0.51266
C	5.43445	-0.25334	-0.39355
C	4.83132	0.97039	-0.02020
C	3.47175	1.15527	0.22666
C	2.60677	0.01743	0.10450
C	0.14788	-1.22463	2.72605
H	-5.36542	1.88237	-0.50157
H	-5.20124	-2.34584	0.06479
H	-3.22207	-3.46438	0.15572
H	-1.37442	-4.76879	0.14268
H	-0.35662	-4.17537	1.48048
H	1.20046	-4.66402	-0.35648
H	0.14798	-3.81970	-1.51544
H	2.94524	-3.28965	-0.82227
H	4.97467	-2.31530	-0.79972
H	5.47685	1.83151	0.08183
H	-0.16514	-0.27488	3.18618
H	-0.46709	-2.00735	3.19419
H	1.18297	-1.39187	3.06021
C	6.94641	-0.39962	-0.65920
C	7.54619	-1.43629	0.31936
H	7.07866	-2.42103	0.20683
H	8.62218	-1.55838	0.14069
H	7.41017	-1.12067	1.36057
C	7.17308	-0.88421	-2.11028

H	6.76647	-0.16758	-2.83374
H	8.24498	-1.00051	-2.31531
H	6.69445	-1.85227	-2.29609
C	7.70822	0.92640	-0.47495
H	7.35587	1.70268	-1.16426
H	7.62119	1.31247	0.54741
H	8.77452	0.76970	-0.67583
C	2.91815	2.53898	0.62900
C	2.24822	2.45561	2.02149
H	1.43717	1.72613	2.03397
H	2.97933	2.16885	2.78790
H	1.83651	3.43370	2.30200
C	4.02104	3.61324	0.71565
H	4.78098	3.37267	1.46888
H	4.52681	3.77075	-0.24457
H	3.56820	4.56886	1.00474
C	1.89109	3.01535	-0.42449
H	2.36468	3.12026	-1.40903
H	1.06183	2.31356	-0.51470
H	1.48718	3.99623	-0.14243
C	-2.71949	2.49333	-0.42594
C	-1.92459	2.86364	0.84877
H	-1.13831	2.13763	1.05781
H	-1.45969	3.85046	0.72918
H	-2.59036	2.91284	1.71979
C	-1.77628	2.44909	-1.65224
H	-2.34478	2.24951	-2.56963
H	-1.27029	3.41482	-1.77702
H	-1.01578	1.67419	-1.54390
C	-3.73779	3.62503	-0.67262
H	-4.32696	3.46617	-1.58380
H	-4.43116	3.75286	0.16745

H	-3.19837	4.57135	-0.79575
C	-7.02986	-0.23865	-0.32931
C	-7.63584	0.65068	0.78171
H	-7.29217	1.68842	0.70926
H	-8.73103	0.65986	0.71210
H	-7.36329	0.27867	1.77648
C	-7.61443	-1.65538	-0.17957
H	-7.25034	-2.33340	-0.96063
H	-7.37400	-2.09608	0.79517
H	-8.70689	-1.61721	-0.26206
C	-7.45526	0.31370	-1.71013
H	-8.54891	0.32154	-1.80085
H	-7.10419	1.33956	-1.86724
H	-7.05312	-0.30360	-2.52233

$A_3C_1B_2$

Al	0.06228	0.81998	1.00572
C	0.28781	0.77553	2.97385
O	-1.36535	-0.27809	0.69661
C	-2.59250	-0.17933	0.18704
C	-3.59156	-1.17382	0.42108
C	-4.85256	-0.96652	-0.14583
C	-5.20732	0.15165	-0.92281
C	-4.21152	1.10452	-1.13030
C	-2.92738	0.94368	-0.59957
C	-1.86282	1.95664	-0.91293
N	-1.08229	2.45478	0.28387
C	-0.27455	3.62827	-0.16037
C	0.92692	3.85675	0.74437
N	1.73741	2.63203	0.85311
C	2.50639	2.40009	-0.40168
C	3.25028	1.09313	-0.41276
C	4.63384	1.06900	-0.61197
C	5.33526	-0.13365	-0.69282
C	4.57945	-1.31147	-0.56334
C	3.19533	-1.34820	-0.35722
C	2.51473	-0.09965	-0.26798
O	1.18056	-0.03521	-0.09918
C	-2.01118	2.88245	1.36717
C	2.66578	2.75036	1.99163
H	-0.39596	0.04512	3.42480
H	0.09566	1.73210	3.48028
H	1.30319	0.47248	3.26342
H	-5.61503	-1.71659	0.02704
H	-4.41151	1.99390	-1.72109
H	-2.31655	2.82648	-1.40613
H	-1.11208	1.54284	-1.59930
H	-0.90899	4.52396	-0.18610
H	0.04964	3.43689	-1.18579
H	1.51798	4.70431	0.35901
H	0.59553	4.13076	1.75069
H	3.20664	3.23633	-0.56161
H	1.79275	2.41164	-1.23176
H	5.14948	2.01966	-0.71166
H	5.10809	-2.25459	-0.62800
H	-2.73655	3.60923	0.97976
H	-1.44190	3.34106	2.17725
H	-2.54904	2.02087	1.76155
H	3.36142	3.59454	1.85345
H	3.24971	1.83422	2.08976
H	2.10178	2.90614	2.91301
C	-3.29416	-2.43472	1.26084
C	-2.88438	-2.03110	2.69673
H	-1.99250	-1.40288	2.69029
H	-3.69320	-1.47959	3.19311

H	-2.67236	-2.92510	3.29784
C	-4.51441	-3.37000	1.37818
H	-4.85253	-3.73824	0.40229
H	-4.24023	-4.24535	1.97901
H	-5.36416	-2.88679	1.87544
C	-2.15269	-3.24243	0.60022
H	-2.44712	-3.58427	-0.40006
H	-1.24883	-2.63982	0.50351
H	-1.91621	-4.13027	1.20172
C	-6.63267	0.27576	-1.49382
C	-6.82352	1.57017	-2.30659
H	-6.66038	2.46565	-1.69503
H	-6.14588	1.61693	-3.16726
H	-7.84847	1.61930	-2.69311
C	-7.66222	0.28095	-0.33948
H	-8.68395	0.36248	-0.73256
H	-7.61030	-0.63564	0.25813
H	-7.49170	1.12780	0.33625
C	-6.92712	-0.92179	-2.42763
H	-6.84805	-1.87892	-1.90067
H	-7.94292	-0.85208	-2.83811
H	-6.22350	-0.94712	-3.26830
C	2.44766	-2.69435	-0.23563
C	1.81077	-2.81756	1.16861
H	1.08570	-2.02384	1.35450
H	1.29172	-3.77936	1.26888
H	2.58027	-2.77053	1.94956
C	1.35388	-2.79299	-1.32578
H	0.82183	-3.74952	-1.24330
H	0.62671	-1.98485	-1.23577
H	1.80133	-2.74619	-2.32698
C	3.38367	-3.90690	-0.41804
H	3.86882	-3.91353	-1.40139
H	4.16485	-3.95207	0.35022
H	2.79608	-4.82906	-0.33793
C	6.85790	-0.21292	-0.91434
C	7.50213	1.18102	-1.03420
H	7.09992	1.74651	-1.88310
H	7.35663	1.77887	-0.12666
H	8.58266	1.07910	-1.19022
C	7.52171	-0.94532	0.27541
H	7.13756	-1.96444	0.39334
H	8.60740	-1.01517	0.12912
H	7.34128	-0.41162	1.21631
C	7.15683	-0.99070	-2.21750
H	8.23923	-1.05894	-2.38699
H	6.76248	-2.01211	-2.18339
H	6.70968	-0.49097	-3.08520

$A_3C_1B_3$

Al	-0.01461	-0.02350	0.85302
C	0.43103	0.37451	2.73822
O	-1.55833	-0.96264	0.98621
C	-2.74597	-0.98895	0.37908
C	-3.80677	-1.82281	0.84884
C	-5.00871	-1.79587	0.13905
C	-5.25135	-0.99546	-0.99244
C	-4.20970	-0.16699	-1.40119
C	-2.97934	-0.15169	-0.73083
C	-1.87086	0.71137	-1.27123
N	-1.04304	1.47015	-0.24966
C	-0.16045	2.40087	-1.01377
C	1.09217	2.80748	-0.24819
N	1.88897	1.63687	0.13965
C	2.55439	1.02098	-1.03909

C	3.11661	-0.35388	-0.78567
C	4.45979	-0.61899	-1.07424
C	4.99636	-1.89880	-0.94617
C	4.11819	-2.90611	-0.51578
C	2.76466	-2.70673	-0.22314
C	2.25341	-1.38474	-0.36328
O	0.94667	-1.11417	-0.16318
C	-1.88970	2.22029	0.76520
C	2.85174	1.95330	1.23341
H	-0.21723	-0.22471	3.38912
H	0.29776	1.42080	3.04634
H	1.46572	0.10679	2.98518
H	-5.81459	-2.43268	0.48307
H	-4.32854	0.48476	-2.26224
H	-2.28646	1.43530	-1.98310
H	-1.13770	0.10093	-1.81570
H	-0.72603	3.29272	-1.30394
H	0.11482	1.89203	-1.93927
H	1.66108	3.52239	-0.86522
H	0.81234	3.33999	0.66710
H	3.34202	1.68703	-1.42306
H	1.80025	0.93969	-1.82732
H	5.07418	0.20322	-1.42770
H	4.51772	-3.90696	-0.41001
H	-1.19960	2.55377	1.54393
H	-2.54721	1.47631	1.21596
H	3.35706	1.01638	1.48090
H	2.25269	2.23633	2.10247
C	-3.63589	-2.72227	2.09095
C	-3.28560	-1.85579	3.32505
H	-2.36161	-1.29781	3.16773
H	-4.09146	-1.14234	3.53821
H	-3.15963	-2.49299	4.20964
C	-4.91970	-3.50592	2.43279
H	-5.21773	-4.18600	1.62627
H	-4.73956	-4.11675	3.32479
H	-5.76423	-2.84256	2.65407
C	-2.51517	-3.75751	1.83860
H	-2.77687	-4.41252	0.99872
H	-1.56916	-3.26740	1.60914
H	-2.37508	-4.38747	2.72669
C	-6.61371	-1.05973	-1.70601
C	-6.68185	-0.11180	-2.91852
H	-6.54238	0.93578	-2.62619
H	-5.92533	-0.35940	-3.67204
H	-7.66422	-0.19164	-3.39800
C	-7.74002	-0.66057	-0.72303
H	-8.71888	-0.71196	-1.21645
H	-7.77229	-1.32188	0.14910
H	-7.59632	0.36336	-0.35844
C	-6.86769	-2.49994	-2.21193
H	-6.86947	-3.22423	-1.39072
H	-7.83978	-2.56547	-2.71699
H	-6.09239	-2.80603	-2.92357
C	1.87786	-3.88440	0.23706
C	1.39691	-3.63421	1.68590
H	0.79969	-2.72451	1.76185
H	0.77836	-4.47157	2.03170
H	2.25209	-3.54289	2.36648
C	0.66478	-4.05198	-0.70991
H	0.05047	-4.90086	-0.38435
H	0.04040	-3.15858	-0.72330
H	1.00272	-4.25570	-1.73357
C	2.63855	-5.22746	0.23894

H	3.01156	-5.49225	-0.75735
H	3.48517	-5.22702	0.93539
H	1.95599	-6.02377	0.55662
C	6.46610	-2.23695	-1.25592
C	7.26329	-1.00378	-1.72210
H	6.84651	-0.57118	-2.63894
H	7.28840	-0.22064	-0.95510
H	8.29999	-1.29011	-1.93356
C	7.15087	-2.79678	0.01362
H	6.65399	-3.70217	0.37741
H	8.19815	-3.05110	-0.19338
H	7.13354	-2.05926	0.82442
C	6.53135	-3.29871	-2.37967
H	7.57375	-3.55615	-2.60647
H	6.01437	-4.22170	-2.09731
H	6.06537	-2.92407	-3.29832
C	-2.69460	3.40499	0.26508
C	-3.97617	3.23877	-0.28042
C	-2.18958	4.70814	0.39360
C	-4.71737	4.34160	-0.70702
H	-4.39841	2.24171	-0.35870
C	-2.92825	5.81265	-0.03194
H	-1.21231	4.86232	0.84656
C	-4.19492	5.63065	-0.58883
H	-5.70998	4.19267	-1.12366
H	-2.51844	6.81286	0.07999
H	-4.77515	6.48836	-0.91809
C	3.88324	3.03815	0.95639
C	5.16146	2.71361	0.47958
C	3.59404	4.38870	1.20356
C	6.11080	3.70650	0.23144
H	5.41792	1.67032	0.31852
C	4.53827	5.38565	0.95599
H	2.62433	4.66368	1.61274
C	5.79975	5.04705	0.46307
H	7.09628	3.43088	-0.13487
H	4.29298	6.42494	1.15897
H	6.53853	5.82116	0.27366

$A_3C_2B_1$

Al	0.00904	1.01843	0.69915
C	0.39748	1.15850	2.65107
O	-1.30713	-0.07372	0.06265
C	-2.62668	-0.04371	0.00324
C	-3.36189	-1.21004	-0.38685
C	-4.75128	-1.11801	-0.41864
C	-5.49829	0.04099	-0.10192
C	-4.77021	1.16026	0.25619
C	-3.35813	1.14033	0.31033
C	-2.69590	2.37452	0.64380
N	-1.41800	2.54484	0.72979
C	-0.89041	3.88014	1.00138
C	-0.20053	4.45738	-0.25615
C	0.26997	3.36923	-1.23215
N	0.97943	2.25860	-0.57680
C	2.25617	2.14213	-0.82221
C	3.12226	1.07847	-0.42052
C	4.50911	1.23697	-0.66795
C	5.40896	0.22874	-0.39499
C	4.86487	-0.98276	0.10812
C	3.51810	-1.22175	0.34894
C	2.59476	-0.14489	0.10212
O	1.31085	-0.27984	0.30446
C	-2.64071	-2.52748	-0.74584
C	-1.68022	-2.30028	-1.93806

C	-1.85132	-3.04206	0.48144	H	-2.52708	-3.23237	1.32487
C	-3.62515	-3.64066	-1.15817	H	-4.21509	-3.36971	-2.04216
C	-7.03570	0.00924	-0.17185	H	-4.31802	-3.90589	-0.35067
C	-7.66026	1.36194	0.21752	H	-3.05866	-4.54482	-1.40951
C	-7.48565	-0.33088	-1.61208	H	-7.10767	-1.30544	-1.94018
C	-7.57933	-1.06680	0.79713	H	-7.34300	2.16830	-0.45435
C	6.92655	0.35212	-0.61858	H	-7.40366	1.65090	1.24358
C	7.31987	1.73825	-1.16185	H	-8.75300	1.29725	0.15842
C	7.66911	0.13304	0.72044	H	-7.12787	0.42177	-2.32483
C	7.39383	-0.71308	-1.63831	H	-8.58079	-0.36306	-1.67540
C	3.02637	-2.59146	0.86224	H	-7.20554	-2.06667	0.55077
C	4.18247	-3.58975	1.07277	H	-8.67529	-1.10407	0.75433
C	2.06140	-3.21302	-0.17531	H	-7.28847	-0.84843	1.83152
C	2.30607	-2.42882	2.22156	H	6.85463	1.94474	-2.13305
H	-0.22681	1.85957	3.22208	H	8.40601	1.78782	-1.30198
H	1.44627	1.42398	2.85481	H	7.04069	2.54263	-0.47105
H	0.25262	0.17087	3.11493	H	8.75427	0.20899	0.57541
H	-5.30863	-2.00122	-0.70621	H	7.46177	-0.85452	1.14708
H	-5.26750	2.09330	0.50444	H	7.37340	0.88447	1.46222
H	-3.35075	3.23591	0.82303	H	6.89497	-0.57790	-2.60539
H	-0.15883	3.78042	1.80952	H	7.18201	-1.73156	-1.29465
H	-1.68210	4.55432	1.35256	H	8.47638	-0.63915	-1.80272
H	-0.59956	2.94010	-1.74103	H	3.77355	-4.54180	1.43074
H	0.90703	3.81808	-2.00364	H	4.90204	-3.24192	1.82384
H	2.72529	2.92573	-1.42677	H	4.72821	-3.79879	0.14483
H	4.83993	2.18708	-1.07700	H	1.20335	-2.56505	-0.35992
H	5.56314	-1.78491	0.31481	H	1.69239	-4.18203	0.18489
H	-2.23455	-1.96888	-2.82557	H	2.57585	-3.38538	-1.12927
H	-1.17079	-3.23779	-2.19520	H	1.44575	-1.76372	2.14097
H	-0.92239	-1.55086	-1.70496	H	2.98759	-2.01830	2.97719
H	-1.09536	-2.32349	0.80106	H	1.95644	-3.40499	2.58181
H	-1.34790	-3.98624	0.23797	H	-0.89362	5.10800	-0.80327

H	0.64791	5.07880	0.05310

$A_3C_3B_1$

Al	0.01689	0.71700	0.73696
C	0.39371	0.79230	2.69505
O	-1.29621	-0.35973	0.06507
C	-2.61643	-0.33171	0.01860
C	-3.35220	-1.48775	-0.40038
C	-4.74206	-1.39906	-0.41660
C	-5.48943	-0.25283	-0.05705
C	-4.76124	0.85678	0.32974
C	-3.34863	0.83994	0.36943
C	-2.68663	2.06592	0.73272
N	-1.40746	2.23702	0.80379
C	-0.87306	3.56008	1.10519
C	-0.23300	4.21829	-0.15315
C	0.27090	3.12103	-1.12603
N	0.98872	2.00699	-0.48854
C	2.26470	1.89708	-0.74278
C	3.13276	0.82327	-0.37250
C	4.51942	0.99203	-0.61415
C	5.42112	-0.02255	-0.37237
C	4.87904	-1.25048	0.09127
C	3.53239	-1.49995	0.32193
C	2.60718	-0.41755	0.10893
O	1.32293	-0.56250	0.30317
C	-2.63106	-2.79175	-0.80567
C	-1.68009	-2.52606	-1.99756
C	-1.83143	-3.34234	0.39912
C	-3.61631	-3.89387	-1.24495
C	-7.02727	-0.28690	-0.11488
C	-7.65214	1.05181	0.31960
C	-7.48855	-0.58546	-1.56078
C	-7.55978	-1.39292	0.82611
C	-1.28342	5.05219	-0.90867
C	0.92532	5.12256	0.29840
C	6.93876	0.11100	-0.58968
C	7.32932	1.51283	-1.09314
C	7.67911	-0.14370	0.74425
C	7.41089	-0.92394	-1.63795
C	3.04283	-2.88688	0.78894
C	4.20077	-3.88906	0.96869
C	2.08137	-3.47584	-0.27067
C	2.31962	-2.77075	2.15144
H	-0.23313	1.47618	3.28394
H	1.44151	1.04839	2.91506
H	0.24296	-0.20957	3.12540
H	-5.29948	-2.27475	-0.72621
H	-5.25898	1.77997	0.61158
H	-3.34079	2.91812	0.95205
H	-0.10640	3.43056	1.87662
H	-1.64977	4.21835	1.51761
H	-0.58967	2.68843	-1.64750
H	0.90845	3.58742	-1.88787
H	2.73055	2.69462	-1.33077
H	4.84870	1.95522	-0.99278
H	5.57868	-2.05740	0.27320
H	-2.24195	-2.16986	-2.87061
H	-1.17002	-3.45396	-2.28640
H	-0.92263	-1.78168	-1.74783
H	-1.07531	-2.63188	0.73600
H	-1.32696	-4.27676	0.12235
H	-2.50051	-3.56134	1.24089
H	-4.21385	-3.59662	-2.11526
H	-4.30217	-4.18618	-0.44082

H	-3.04985	-4.78829	-1.52908
H	-7.11057	-1.54865	-1.92095
H	-7.34236	1.87868	-0.33047
H	-7.38808	1.31085	1.35174
H	-8.74515	0.98581	0.26733
H	-7.13912	0.18925	-2.25378
H	-8.58410	-0.61921	-1.61567
H	-7.18605	-2.38390	0.54601
H	-8.65600	-1.43158	0.79211
H	-7.25992	-1.20524	1.86393
H	-2.16961	4.45440	-1.15477
H	-0.87681	5.44307	-1.84974
H	-1.60901	5.90895	-0.30701
H	1.73494	4.54066	0.75434
H	0.58375	5.85178	1.04343
H	1.34184	5.68405	-0.54683
H	6.86453	1.74550	-2.05862
H	8.41549	1.56900	-1.23060
H	7.04748	2.29675	-0.38026
H	8.76445	-0.06212	0.60371
H	7.47222	-1.14299	1.14293
H	7.38059	0.58645	1.50588
H	6.91320	-0.76334	-2.60174
H	7.20169	-1.95225	-1.32324
H	8.49346	-0.84216	-1.79838
H	3.79334	-4.85322	1.29449
H	4.91832	-3.56455	1.73203
H	4.74857	-4.06646	0.03539
H	1.22241	-2.82380	-0.43562
H	1.71364	-4.45698	0.05647
H	2.59828	-3.61547	-1.22864
H	1.45884	-2.10405	2.09146
H	2.99922	-2.38521	2.92182
H	1.97016	-3.75876	2.47798

$A_3C_4B_1$

Al	-0.02614	0.47197	0.76481
C	-0.38548	0.57173	2.72519
O	-1.33612	-0.80561	0.35229
C	-2.61800	-0.67660	0.12735
C	-3.54387	-1.75096	0.35026
C	-4.88943	-1.51751	0.07340
C	-5.42022	-0.31124	-0.44266
C	-4.51043	0.70062	-0.68856
C	-3.13489	0.54868	-0.40243
C	-2.26715	1.62965	-0.75677
N	-1.00836	1.78134	-0.45078
C	-0.30469	2.90580	-1.09811
C	0.33631	3.97879	-0.16884
C	0.92494	3.29908	1.09725
N	1.42260	1.96011	0.79992
C	2.69858	1.76359	0.72483
C	3.33882	0.52671	0.36320
C	4.75103	0.52168	0.30830
C	5.45691	-0.60060	-0.08292
C	4.68705	-1.73598	-0.42972
C	3.29641	-1.80320	-0.39618
C	2.58365	-0.63381	0.02658
O	1.26455	-0.63800	0.09114
C	-0.66121	5.09035	0.25502
C	1.47542	4.61381	-1.02202
H	-0.22099	-0.42246	3.16833
H	-1.43405	0.82042	2.94989
H	0.23921	1.26970	3.29974
H	-5.58371	-2.32451	0.26213

H	-4.83855	1.65105	-1.10453
H	-2.72940	2.40640	-1.37431
H	-0.98209	3.40597	-1.80206
H	0.49626	2.45699	-1.69660
H	1.71588	3.92493	1.52952
H	0.14442	3.19200	1.85624
H	3.36923	2.60401	0.93889
H	5.26623	1.43814	0.58069
H	5.22694	-2.62129	-0.74304
H	-0.87396	5.70947	-0.62813
H	-0.14236	5.75064	0.96210
H	1.05979	4.86380	-2.00873
H	2.23320	3.84062	-1.20775
C	-6.92121	-0.10990	-0.73397
C	-7.45912	1.06580	0.11492
C	-7.12090	0.21552	-2.23272
C	-7.75897	-1.35927	-0.40239
H	-7.34147	0.86461	1.18635
H	-6.93388	2.00164	-0.10668
H	-8.52616	1.22941	-0.08344
H	-6.75869	-0.60326	-2.86574
H	-8.18434	0.37038	-2.45570
H	-6.58611	1.12532	-2.52776
H	-8.81467	-1.16496	-0.62530
H	-7.45376	-2.22903	-0.99594
H	-7.69287	-1.62954	0.65802
C	-3.06518	-3.12102	0.87511
C	-2.08445	-3.74562	-0.14536
C	-2.36746	-2.95758	2.24604
C	-4.22600	-4.11740	1.06863
H	-2.58462	-3.92136	-1.10632
H	-1.22500	-3.09671	-0.31924

H	-1.71941	-4.71305	0.22315
H	-3.06164	-2.54671	2.98992
H	-2.02360	-3.93338	2.61295
H	-1.50630	-2.29206	2.17945
H	-3.82442	-5.06790	1.43902
H	-4.95951	-3.76538	1.80411
H	-4.75447	-4.33129	0.13187
C	2.55115	-3.09910	-0.78187
C	1.77281	-3.63295	0.44409
C	1.57761	-2.82708	-1.95390
C	3.51272	-4.21532	-1.23768
H	2.45933	-3.85911	1.26980
H	1.03691	-2.90889	0.79670
H	1.24678	-4.56019	0.18412
H	2.12452	-2.47844	-2.83947
H	1.05307	-3.75082	-2.22972
H	0.83279	-2.07493	-1.69015
H	2.92884	-5.10280	-1.50799
H	4.09663	-3.92791	-2.12052
H	4.21062	-4.51508	-0.44677
C	6.99318	-0.65961	-0.15927
C	7.51947	-1.77511	0.77400
C	7.43179	-0.96400	-1.61100
C	7.64470	0.66833	0.26900
H	7.23521	-1.58409	1.81560
H	7.12697	-2.75979	0.49739
H	8.61447	-1.83092	0.72667
H	7.08648	-0.18295	-2.29892
H	8.52588	-1.01561	-1.67957
H	7.03351	-1.92045	-1.96721
H	8.73585	0.58451	0.20471
H	7.34143	1.50068	-0.37713

H	7.39605	0.93087	1.30408
C	-1.99552	4.67080	0.88528
H	-2.65842	4.17780	0.16807
H	-1.87053	3.99574	1.73885
H	-2.52343	5.55994	1.25036
C	2.16677	5.86203	-0.46130
H	2.99551	6.15172	-1.11804
H	1.48884	6.71939	-0.39756
H	2.58953	5.69734	0.53702

$A_3C_5B_1$

O	-1.22752	-0.53017	0.36779
O	1.38317	-0.45886	0.87960
N	-1.47972	2.13064	0.60187
N	1.03281	2.11282	0.07369
C	-0.88518	3.45555	0.72259
C	-1.73663	4.68952	0.41273
C	-0.90015	5.96291	0.63229
C	0.40668	5.93802	-0.17635
C	1.23019	4.66434	0.08585
C	0.37265	3.41479	-0.17120
C	-2.70504	1.93076	0.24654
C	-3.29260	0.63921	0.02202
C	-4.66760	0.60481	-0.30512
C	-5.31635	-0.58086	-0.58618
C	-4.52335	-1.75243	-0.54327
C	-3.16653	-1.79411	-0.23950
C	-2.51221	-0.55504	0.07030
C	-6.81100	-0.67638	-0.93633
C	-7.49455	0.70397	-0.93519
C	-7.53213	-1.56975	0.10102
C	-6.97859	-1.29353	-2.34531
C	-2.39526	-3.12973	-0.22570
C	-3.28843	-4.32866	-0.60612
C	-1.23877	-3.07788	-1.25368
C	-1.84788	-3.40015	1.19670
C	2.18815	1.89889	-0.49295
C	3.00429	0.73496	-0.38401
C	4.27363	0.77054	-1.01586
C	5.16230	-0.27585	-0.90548
C	4.74397	-1.37537	-0.10952
C	3.51733	-1.48763	0.53006
C	2.57982	-0.40789	0.36372
C	6.54865	-0.29644	-1.57067
C	7.64727	-0.40961	-0.48679
C	6.81535	0.98051	-2.38960
C	6.64803	-1.50903	-2.52672
C	3.17524	-2.71533	1.39837
C	2.84282	-2.26266	2.84156
C	1.96904	-3.46748	0.79131
C	4.34728	-3.71368	1.48931
H	-0.53606	3.53433	1.76393
H	-2.08979	4.65094	-0.62780
H	-2.62630	4.71159	1.05411
H	-1.49131	6.84729	0.36653
H	-0.66385	6.05527	1.70161
H	1.01060	6.82292	0.05725
H	0.17012	5.99711	-1.24859
H	2.12188	4.66950	-0.55107
H	1.58304	4.64383	1.12611
H	0.02968	3.43035	-1.21839
H	-3.36591	2.78800	0.08483
H	-5.20079	1.55102	-0.32694
H	-5.01720	-2.69017	-0.76707
H	-8.55542	0.59327	-1.18691

H	-7.04887	1.37950	-1.67470
H	-7.43700	1.18564	0.04782
H	-8.59891	-1.65504	-0.14099
H	-7.44395	-1.14787	1.10872
H	-7.11577	-2.58235	0.12660
H	-8.04152	-1.37600	-2.60453
H	-6.54389	-2.29689	-2.40589
H	-6.48999	-0.67242	-3.10485
H	-4.11793	-4.46969	0.09694
H	-2.68520	-5.24325	-0.58604
H	-3.70413	-4.23286	-1.61630
H	-0.54403	-2.26682	-1.03400
H	-1.63365	-2.93364	-2.26713
H	-0.68280	-4.02357	-1.24180
H	-2.67282	-3.48639	1.91455
H	-1.18425	-2.60156	1.52960
H	-1.28827	-4.34360	1.21250
H	2.61109	2.69642	-1.11201
H	4.52647	1.66115	-1.58413
H	5.44742	-2.19094	0.00765
H	7.61083	0.44577	0.19755
H	8.64184	-0.43405	-0.94942
H	7.53836	-1.32002	0.11213
H	6.79476	1.87870	-1.76141
H	6.08212	1.10780	-3.19452
H	7.80727	0.92479	-2.85217
H	6.50047	-2.45759	-1.99958
H	7.63640	-1.54341	-3.00181
H	5.89176	-1.44635	-3.31752
H	1.99389	-1.57806	2.86146
H	3.70436	-1.75994	3.29795
H	2.59792	-3.13582	3.45931

H	1.09366	-2.82218	0.72613
H	1.71519	-4.33558	1.41303
H	2.20660	-3.83235	-0.21555
H	5.24326	-3.26294	1.93269
H	4.61722	-4.12868	0.51089
H	4.05227	-4.55335	2.12853
Al	-0.06296	0.69985	1.08506
C	-0.09369	0.93502	3.05484
H	-0.08740	-0.04565	3.55175
H	0.79354	1.46575	3.43030
H	-0.97387	1.47218	3.43504

$A_3C_6B_1$

Al	0.05657	0.77465	0.92624
O	-1.34506	-0.42179	0.67047
O	1.26303	-0.44260	0.23997
N	-1.18336	2.22785	0.17286
N	1.37087	2.25507	0.26731
C	-0.61786	3.52066	0.21241
C	-1.33116	4.72503	0.24148
C	-0.64577	5.93626	0.31111
C	0.75084	5.95369	0.37764
C	1.46987	4.76028	0.37220
C	0.79276	3.53837	0.27859
C	-2.38791	2.04623	-0.30885
C	-3.12871	0.83309	-0.28988
C	-2.59020	-0.36818	0.27375
C	-3.46461	-1.50502	0.38817
C	-4.74733	-1.38376	-0.13102
C	-5.28006	-0.22291	-0.75223
C	-4.45078	0.87526	-0.80611
C	-3.00359	-2.80727	1.07574
C	-2.57441	-2.50784	2.53238

C	-1.82093	-3.42186	0.29498	H	-1.48868	-4.34866	0.78013
C	-4.12118	-3.86770	1.13681	H	-0.97827	-2.73296	0.25232
C	-6.71796	-0.23858	-1.29980	H	-2.11802	-3.66951	-0.73199
C	-7.10800	1.10835	-1.93585	H	-3.74039	-4.75919	1.64847
C	-6.84912	-1.33667	-2.38132	H	-4.45284	-4.18316	0.14024
C	-7.71202	-0.53405	-0.15207	H	-4.99732	-3.51961	1.69711
C	2.62076	2.06837	-0.05084	H	-8.13289	1.05405	-2.32084
C	3.27658	0.80283	-0.12292	H	-6.45447	1.36800	-2.77702
C	2.55816	-0.42763	0.00136	H	-7.07402	1.92938	-1.20984
C	3.29055	-1.64962	-0.17934	H	-7.87268	-1.36631	-2.77576
C	4.65699	-1.55610	-0.42136	H	-6.61951	-2.33218	-1.98560
C	5.38993	-0.34840	-0.53483	H	-6.16876	-1.14690	-3.22000
C	4.66777	0.81783	-0.39567	H	-8.74151	-0.55172	-0.53148
C	6.90372	-0.39175	-0.80697	H	-7.65235	0.23397	0.62825
C	7.51206	1.01881	-0.91233	H	-7.51883	-1.50367	0.32006
C	7.61641	-1.13848	0.34505	H	3.23234	2.93482	-0.31380
C	7.17468	-1.13284	-2.13727	H	5.21100	-2.47914	-0.53876
C	2.58936	-3.02249	-0.11677	H	5.14820	1.78746	-0.48580
C	3.56973	-4.19566	-0.32058	H	7.06914	1.59542	-1.73304
C	1.53543	-3.10674	-1.24692	H	8.58840	0.94542	-1.10659
C	1.92689	-3.22018	1.26725	H	7.38429	1.58974	0.01492
C	0.21967	1.00745	2.88617	H	7.45685	-0.62921	1.30286
H	-2.41663	4.71930	0.24197	H	8.69741	-1.18442	0.16190
H	-1.20425	6.86733	0.34145	H	7.25576	-2.16760	0.45162
H	1.28044	6.89852	0.45852	H	6.69281	-0.62096	-2.97879
H	2.55097	4.78436	0.46600	H	6.80237	-2.16298	-2.11706
H	-2.89476	2.90472	-0.75559	H	8.25233	-1.17571	-2.33957
H	-5.40291	-2.24265	-0.05545	H	4.06551	-4.16205	-1.29808
H	-4.79030	1.81308	-1.23535	H	3.01303	-5.13868	-0.27166
H	-2.23835	-3.43042	3.02266	H	4.34253	-4.23365	0.45667
H	-3.41581	-2.10871	3.11262	H	0.80091	-2.30351	-1.17318
H	-1.75821	-1.78445	2.57145	H	1.00451	-4.06589	-1.20213

H	2.02051	-3.03969	-2.22908
H	1.18297	-2.45054	1.47453
H	2.68221	-3.19067	2.06277
H	1.43345	-4.19907	1.31342
H	0.03714	0.06327	3.41964
H	-0.50384	1.73188	3.28958
H	1.21531	1.35464	3.19856

$A_3C_6B_2$

Al	-0.05407	0.49316	-1.23910
C	-0.31211	0.55636	-3.20287
O	1.39954	-0.58047	-0.96874
C	2.53981	-0.55058	-0.28124
C	3.50891	-1.59043	-0.38759
C	4.68003	-1.47034	0.37401
C	4.97340	-0.38406	1.21199
C	4.02204	0.63846	1.26025
C	2.82903	0.56418	0.54109
C	1.83333	1.68746	0.65798
N	1.23094	2.12921	-0.66760
N	-1.56543	2.17898	-0.70976
C	-2.18850	1.81887	0.61991
C	-3.02088	0.57266	0.56670
C	-4.35691	0.59569	0.97912
C	-5.13287	-0.56257	0.98008
C	-4.50117	-1.74216	0.54701
C	-3.17143	-1.82203	0.11925
C	-2.41311	-0.61461	0.11689
O	-1.12773	-0.58261	-0.26416
C	2.27875	2.27203	-1.72783
C	-2.60952	2.26819	-1.76416
H	0.43489	-0.07823	-3.69848
H	-0.21466	1.55631	-3.64901
H	-1.29427	0.17459	-3.51280
H	5.40885	-2.26558	0.30242
H	4.19848	1.52003	1.87385
H	2.31565	2.55285	1.12591
H	0.98106	1.41298	1.29414
H	-2.79971	2.66237	0.96668
H	-1.35470	1.70746	1.32369
H	-4.77336	1.54334	1.30805
H	-5.08868	-2.65222	0.54670
H	3.12757	2.86659	-1.37524
H	1.82826	2.75986	-2.59324
H	2.63715	1.28779	-2.02046
H	-3.44647	2.90812	-1.45746
H	-3.00169	1.27170	-1.96611
H	-2.16473	2.66750	-2.67596
C	0.54172	3.40537	-0.49334
C	-0.85399	3.43478	-0.55597
C	-1.52297	4.66013	-0.42503
C	-0.81189	5.84053	-0.23308
C	0.58205	5.80726	-0.15776
C	1.25295	4.59511	-0.28633
H	-2.60654	4.69084	-0.47086
H	-1.34466	6.78214	-0.13684
H	1.14623	6.72220	-0.00230
H	2.33644	4.57662	-0.23301
C	6.27061	-0.27964	2.03765
C	7.05703	0.98489	1.61870
C	5.92207	-0.18083	3.54150
C	7.19262	-1.49793	1.84289
H	7.33037	0.94459	0.55741
H	6.47456	1.89947	1.77763
H	7.98207	1.07716	2.20254

H	5.37391	-1.06917	3.87704
H	6.83442	-0.09753	4.14641
H	5.29960	0.69441	3.75904
H	8.09661	-1.37962	2.45222
H	6.70857	-2.43175	2.15154
H	7.51227	-1.60872	0.80010
C	3.29355	-2.79962	-1.32461
C	2.01777	-3.56959	-0.91516
C	3.15629	-2.31032	-2.78655
C	4.46879	-3.79715	-1.28355
H	2.10851	-3.95757	0.10709
H	1.13970	-2.92530	-0.96005
H	1.85721	-4.42489	-1.58518
H	4.06479	-1.78633	-3.11029
H	3.00535	-3.16350	-3.46101
H	2.30783	-1.63249	-2.89921
H	4.25758	-4.63013	-1.96469
H	5.41375	-3.34395	-1.60659
H	4.61761	-4.22358	-0.28436
C	-2.55050	-3.17150	-0.29909
C	-2.04176	-3.10350	-1.75822
C	-1.38349	-3.51054	0.65853
C	-3.55990	-4.33526	-0.22500
H	-2.86822	-2.88894	-2.44793
H	-1.28133	-2.33215	-1.88522
H	-1.60385	-4.06597	-2.05266
H	-1.75049	-3.62457	1.68655
H	-0.91113	-4.45692	0.36541
H	-0.62228	-2.72879	0.65093
H	-3.06130	-5.26310	-0.52942
H	-3.94376	-4.49080	0.79016
H	-4.41471	-4.18877	-0.89636

C	-6.60715	-0.59529	1.42607
C	-6.77124	-1.56479	2.62017
C	-7.11259	0.79124	1.86688
C	-7.49648	-1.07890	0.25652
H	-6.47049	-2.58595	2.36177
H	-6.16122	-1.24490	3.47340
H	-7.81845	-1.60074	2.94732
H	-7.05663	1.52530	1.05419
H	-8.16248	0.72357	2.17573
H	-6.54440	1.18204	2.71928
H	-8.55045	-1.11389	0.56128
H	-7.41602	-0.40430	-0.60441
H	-7.21645	-2.08263	-0.08135

$A_3C_7B_1$

Al	0.03279	0.38903	0.59496
C	0.36062	0.50159	2.54868
O	-1.25280	-0.73606	-0.07144
C	-2.56571	-0.78643	-0.06096
C	-3.26906	-1.97419	-0.45188
C	-4.65770	-1.94569	-0.39202
C	-5.44052	-0.83147	0.00226
C	-4.74847	0.31331	0.33538
C	-3.33214	0.35422	0.31195
C	-2.71580	1.61461	0.56863
N	-1.43682	1.88727	0.60970
N	1.14045	1.75358	-0.46793
C	2.45304	1.57565	-0.48431
C	3.20946	0.41314	-0.19185
C	4.62517	0.54244	-0.22271
C	5.44739	-0.54023	-0.01630
C	4.80350	-1.78790	0.21310
C	3.43280	-1.99509	0.25108

C	2.58373	-0.84750	0.06024	H	-2.28211	-4.02129	1.12850
O	1.28448	-0.92600	0.09054	H	-4.11864	-4.09017	-2.15523
C	-2.51539	-3.24091	-0.90205	H	-4.10815	-4.71036	-0.48841
C	-1.62589	-2.91821	-2.12745	H	-2.88069	-5.24090	-1.64053
C	-1.65116	-3.76614	0.26824	H	-7.08863	-2.22965	-1.75832
C	-3.47224	-4.37715	-1.31718	H	-7.40602	1.20108	-0.19878
C	-6.97429	-0.94165	0.02698	H	-7.33094	0.65760	1.49078
C	-7.64021	0.37173	0.47873	H	-8.72940	0.25148	0.48804
C	-7.49615	-1.28248	-1.38923	H	-7.22227	-0.49977	-2.10590
C	-7.39843	-2.05850	1.01037	H	-8.58957	-1.37179	-1.38337
C	6.98309	-0.46439	-0.02744	H	-6.98523	-3.03198	0.72559
C	7.48802	0.96532	-0.29809	H	-8.49103	-2.15517	1.03246
C	7.53536	-0.91716	1.34532	H	-7.05556	-1.83543	2.02716
C	7.54354	-1.38940	-1.13409	H	7.15517	1.33661	-1.27438
C	2.83968	-3.39503	0.49790	H	8.58378	0.97840	-0.29667
C	3.92841	-4.47156	0.68280	H	7.14845	1.66906	0.47062
C	1.98360	-3.80806	-0.72273	H	8.63168	-0.87670	1.34943
C	1.98024	-3.38376	1.78517	H	7.24104	-1.94432	1.58580
H	0.32058	1.52908	2.93661	H	7.16736	-0.26822	2.14815
H	1.33013	0.08181	2.84669	H	7.18079	-1.08217	-2.12168
H	-0.40439	-0.06346	3.10253	H	7.25104	-2.43379	-0.98175
H	-5.19045	-2.84628	-0.67200	H	8.63985	-1.35088	-1.14657
H	-5.27221	1.21943	0.62494	H	3.44650	-5.44153	0.84900
H	-3.41069	2.44964	0.68993	H	4.56763	-4.27161	1.55103
H	3.05445	2.44697	-0.74897	H	4.56826	-4.57194	-0.20204
H	5.03566	1.52873	-0.41730	H	1.17899	-3.09536	-0.90443
H	5.44456	-2.64653	0.37277	H	1.53935	-4.79695	-0.55399
H	-2.24045	-2.57862	-2.97044	H	2.60518	-3.86856	-1.62467
H	-1.08750	-3.81911	-2.44673	H	1.17776	-2.64826	1.72582
H	-0.89457	-2.14265	-1.89695	H	2.59981	-3.14680	2.65868
H	-0.91730	-3.02411	0.58322	H	1.53371	-4.37261	1.94791
H	-1.11462	-4.67301	-0.03640	C	-1.11828	3.26420	0.76831

C	-0.18125	3.83111	-0.14733
C	-1.80239	4.08137	1.65404
C	0.74325	3.05277	-0.91599
C	-0.19008	5.25384	-0.32553
C	-1.68291	5.48556	1.57725
H	-2.46365	3.63357	2.38950
C	1.37623	3.64096	-2.00221
C	0.53869	5.82550	-1.40292
C	-0.94017	6.06056	0.57232
H	-2.22523	6.10609	2.28492
C	1.25176	5.02302	-2.26166
H	2.00880	3.03255	-2.64112
H	0.50069	6.90181	-1.54770
H	-0.90929	7.14023	0.45274
H	1.76626	5.45105	-3.11727

$A_3C_8B_1$

Al	0.10225	0.22761	-0.69386
C	0.60237	0.39112	-2.61667
O	-1.21997	-0.86690	-0.09330
C	-2.53856	-0.87691	-0.08814
C	-3.25526	-2.04327	0.33316
C	-4.64610	-1.99018	0.30024
C	-5.41101	-0.87250	-0.11283
C	-4.70073	0.24615	-0.50305
C	-3.28587	0.26604	-0.49322
C	-2.64963	1.49457	-0.87355
N	-1.37105	1.73277	-0.88391
C	-0.95560	3.03313	-1.30685
C	-1.11900	3.43335	-2.63747
C	-0.69975	4.69764	-3.05029
C	-0.11685	5.57076	-2.13128
C	0.03407	5.17683	-0.80243
C	-0.37526	3.90719	-0.36717
C	-0.30797	3.54382	1.07279
C	-0.87435	4.41337	2.01958
C	-0.84849	4.13173	3.38427
C	-0.24702	2.95715	3.83377
C	0.33156	2.08479	2.91358
C	0.31237	2.36748	1.54102
N	0.98239	1.44732	0.66263
C	2.28747	1.38569	0.82675
C	3.17482	0.38367	0.33841
C	4.56912	0.61490	0.47253
C	5.48989	-0.36265	0.16676
C	4.96681	-1.62273	-0.23505
C	3.61993	-1.93436	-0.35654
C	2.66975	-0.88257	-0.10278
O	1.38359	-1.07094	-0.21359
H	-0.27368	0.32584	-3.27962
H	1.29348	-0.40147	-2.93050
H	1.09247	1.34542	-2.85765
H	-5.19134	-2.87241	0.61301
H	-5.21231	1.14833	-0.82446
H	-3.32364	2.30705	-1.16200
H	-1.56191	2.73929	-3.34503
H	-0.82515	4.99434	-4.08794
H	0.21914	6.55494	-2.44567
H	0.48024	5.85800	-0.08295
H	-1.35638	5.32060	1.66614
H	-1.30428	4.82230	4.08828
H	-0.22535	2.71684	4.89304
H	0.80289	1.16528	3.24824
H	2.74516	2.16630	1.43967
H	4.88430	1.59377	0.82112

H	5.68548	-2.40269	-0.45594
C	-6.94833	-0.94820	-0.10565
C	-7.44988	-1.22963	1.33025
H	-8.54538	-1.29126	1.34906
H	-7.05998	-2.17504	1.72338
H	-7.14426	-0.43189	2.01766
C	-7.59371	0.36359	-0.58921
H	-7.32801	1.21183	0.05270
H	-7.30244	0.60841	-1.61750
H	-8.68564	0.26856	-0.57156
C	-7.41764	-2.08783	-1.04019
H	-7.09038	-1.91152	-2.07174
H	-7.02442	-3.06177	-0.72851
H	-8.51274	-2.15839	-1.03987
C	-2.51300	-3.31665	0.79315
C	-1.61430	-2.99773	2.01223
H	-0.86577	-2.24138	1.77135
H	-2.21629	-2.63395	2.85470
H	-1.09319	-3.90544	2.34257
C	-1.65242	-3.86860	-0.36831
H	-1.13426	-4.78275	-0.05207
H	-2.28133	-4.12333	-1.23074
H	-0.90268	-3.14430	-0.68986
C	-3.48321	-4.43666	1.21991
H	-4.11982	-4.13868	2.06178
H	-4.13117	-4.76383	0.39792
H	-2.90353	-5.30910	1.54286
C	3.15327	-3.35269	-0.74625
C	2.33322	-3.31297	-2.05733
H	1.44898	-2.68256	-1.95776
H	2.94062	-2.92672	-2.88548
H	2.00650	-4.32552	-2.32727
C	2.28978	-3.93848	0.39682
H	1.41781	-3.31413	0.59850
H	1.94038	-4.94387	0.12879
H	2.87517	-4.02404	1.32112
C	4.33534	-4.31642	-0.97263
H	4.98418	-3.99398	-1.79596
H	4.95334	-4.43697	-0.07477
H	3.94576	-5.30695	-1.23477
C	7.01279	-0.16323	0.26049
C	7.61068	-1.16362	1.27768
H	7.41973	-2.20411	0.99260
H	8.69827	-1.03554	1.34803
H	7.18683	-1.00947	2.27718
C	7.38118	1.26038	0.71704
H	7.00815	2.02179	0.02199
H	6.98639	1.48559	1.71489
H	8.47138	1.36474	0.76495
C	7.65375	-0.40442	-1.12644
H	8.74197	-0.27256	-1.07482
H	7.46163	-1.41771	-1.49626
H	7.26227	0.30076	-1.86915

$A_3C_9B_1$

Al	0.09257	0.70373	-1.13561
C	0.39423	1.61443	-2.87956
O	1.79192	-0.11664	-1.21031
C	2.86389	-0.16486	-0.47586
C	4.08770	-0.75680	-0.95312
C	5.17022	-0.76799	-0.08516
C	5.17304	-0.25036	1.24002
C	3.99542	0.31011	1.68232
C	2.84679	0.36988	0.84912
C	1.66465	0.93328	1.40523

N	0.50535	1.16367	0.82638	C	-1.08677	-3.75954	-0.80835
C	-0.51375	1.62459	1.74847	C	-0.77120	-3.65183	-2.31971
C	-1.20669	0.66440	2.49198	C	-1.55079	-5.20545	-0.53944
C	-2.20165	1.04527	3.39274	C	0.19989	-3.52848	0.01991
C	-2.50640	2.39625	3.54949	H	1.30222	2.23372	-2.89829
C	-1.79964	3.35153	2.81833	H	-0.42693	2.23163	-3.26098
C	-0.78840	2.99535	1.91544	H	0.56540	0.82121	-3.62373
C	0.00544	4.07603	1.21302	H	6.09426	-1.20873	-0.43969
C	0.03514	4.03324	-0.33020	H	3.91194	0.72418	2.68284
C	-1.30207	4.14091	-1.02945	H	1.73749	1.20009	2.46260
C	-1.71319	5.41360	-1.45126	H	-0.96573	-0.38458	2.34859
C	-2.89880	5.62765	-2.15064	H	-2.73393	0.28723	3.96060
C	-3.69966	4.53398	-2.47228	H	-3.28295	2.70910	4.24225
C	-3.31874	3.26006	-2.06160	H	-2.02574	4.40638	2.95537
C	-2.14918	3.04175	-1.30665	H	1.04947	4.03896	1.55562
N	-1.84866	1.70078	-0.88947	H	-0.38422	5.05007	1.53042
C	-2.88226	1.00554	-0.49232	H	0.65528	4.87473	-0.66060
C	-2.97119	-0.40725	-0.24851	H	0.56469	3.14023	-0.66387
C	-4.19734	-0.88067	0.27931	H	-1.06544	6.25926	-1.23038
C	-4.42957	-2.22696	0.48266	H	-3.17446	6.63029	-2.46563
C	-3.38526	-3.10907	0.11307	H	-4.60656	4.66225	-3.05697
C	-2.16040	-2.71931	-0.42140	H	-3.91759	2.40557	-2.36095
C	-1.93178	-1.31581	-0.59436	H	-3.82165	1.54130	-0.33459
O	-0.78896	-0.87603	-1.08074	H	-4.95509	-0.14173	0.52056
C	4.19176	-1.33794	-2.37914	H	-3.55966	-4.16847	0.25660
C	3.93619	-0.22168	-3.41964	H	2.94410	0.21677	-3.30181
C	3.15897	-2.47393	-2.56917	H	4.01251	-0.62921	-4.43604
C	5.58592	-1.92930	-2.66900	H	4.68085	0.57869	-3.32440
C	6.45100	-0.34159	2.09247	H	2.13944	-2.11553	-2.42168
C	7.60138	0.41883	1.39171	H	3.34421	-3.29159	-1.86151
C	6.85525	-1.82430	2.26845	H	3.23655	-2.88599	-3.58365
C	6.25648	0.27001	3.49209	H	6.38078	-1.17649	-2.60431

H	5.60071	-2.32984	-3.68925
H	5.83770	-2.75416	-1.99147
H	7.35214	1.47889	1.26328
H	7.82259	0.00685	0.40091
H	8.52119	0.35723	1.98701
H	6.06659	-2.38927	2.77947
H	7.77130	-1.90418	2.86737
H	7.04608	-2.31474	1.30752
H	5.47224	-0.24573	4.05875
H	5.99794	1.33435	3.44145
H	7.18610	0.18526	4.06681
H	-0.41564	-2.65539	-2.58684
H	-1.66398	-3.87069	-2.91902
H	0.00309	-4.37874	-2.59622
H	-2.44786	-5.46930	-1.11226
H	-1.75504	-5.38756	0.52263
H	-0.75691	-5.89847	-0.84097
H	0.62889	-2.54345	-0.16792
H	0.95265	-4.28483	-0.23608
H	-0.00869	-3.61888	1.09385
C	-5.73893	-2.78873	1.06522
C	-5.43663	-3.56353	2.36938
C	-6.75035	-1.67509	1.39388
C	-6.39555	-3.74820	0.04512
H	-4.74773	-4.39856	2.20034
H	-4.98484	-2.90637	3.12213
H	-6.36015	-3.97789	2.79322
H	-7.03567	-1.10497	0.50192
H	-7.66501	-2.11481	1.80831
H	-6.35784	-0.97155	2.13761
H	-7.32824	-4.16020	0.45079
H	-6.63470	-3.22617	-0.88899
H	-5.74306	-4.59244	-0.20344

$A_3C_{12}B_1$

Al	0.03151	-0.63499	-0.84933
C	-0.22943	-0.56712	-2.82319
O	1.24942	-1.75748	-0.07864
C	2.56052	-1.79744	0.05690
C	3.20616	-2.98315	0.53793
C	4.59307	-2.96240	0.64914
C	5.42212	-1.86140	0.32697
C	4.78109	-0.72480	-0.12534
C	3.37484	-0.67097	-0.25980
C	2.80219	0.57368	-0.69280
N	1.54011	0.81509	-0.84303
C	1.10795	2.14882	-1.24570
C	0.36831	2.89513	-0.08865
C	-0.22490	1.85744	0.90359
N	-0.94696	0.73436	0.28671
C	-2.22585	0.64666	0.54677
C	-3.11696	-0.41134	0.19904
C	-4.49897	-0.20384	0.44217
C	-5.42673	-1.19390	0.20517
C	-4.91791	-2.43745	-0.25656
C	-3.58032	-2.72549	-0.48751
C	-2.62544	-1.66847	-0.27537
O	-1.34766	-1.84882	-0.46478
C	2.39341	-4.24367	0.89996
C	1.37002	-3.91670	2.01482
C	1.66195	-4.76786	-0.35959
C	3.28832	-5.38678	1.42152
C	6.94752	-1.97461	0.48997
C	7.67006	-0.67423	0.09036
C	7.29282	-2.28104	1.96677

C	7.48347	-3.11592	-0.40695	H	2.65850	-6.24831	1.66991
C	1.39488	3.73183	0.75065	H	6.83765	-3.21740	2.30644
C	-0.75405	3.76329	-0.74567	H	7.35740	0.17284	0.71216
C	-6.93900	-1.02056	0.42395	H	7.48967	-0.41448	-0.95921
C	-7.29136	0.39114	0.92945	H	8.75157	-0.79590	0.21900
C	-7.68767	-1.25317	-0.91011	H	6.93823	-1.48005	2.62566
C	-7.43640	-2.04320	1.47338	H	8.37841	-2.37336	2.09616
C	-3.12869	-4.12576	-0.94874	H	7.03492	-4.08141	-0.15010
C	-4.31555	-5.09228	-1.13809	H	8.57047	-3.21421	-0.29481
C	-2.19649	-4.73970	0.12325	H	7.26629	-2.91810	-1.46282
C	-2.38945	-4.03374	-2.30531	H	2.06818	3.01451	1.23634
H	0.48764	0.04220	-3.38903	H	0.85171	4.21881	1.56845
H	-1.23670	-0.22208	-3.10040	H	-1.26438	3.12437	-1.47447
H	-0.15163	-1.58872	-3.22264	H	-0.26483	4.54603	-1.33896
H	5.08243	-3.85985	1.00758	H	-6.81559	0.60920	1.89262
H	5.34449	0.16583	-0.38855	H	-8.37496	0.47589	1.06983
H	3.51551	1.38032	-0.90214	H	-6.98942	1.16557	0.21448
H	0.42245	2.01547	-2.08835	H	-8.76992	-1.14208	-0.76782
H	1.95230	2.74863	-1.60593	H	-7.50729	-2.25672	-1.30983
H	0.59717	1.42419	1.48288	H	-7.36774	-0.53008	-1.66915
H	-0.87891	2.37703	1.61349	H	-6.93407	-1.89138	2.43560
H	-2.67701	1.46799	1.11108	H	-7.24853	-3.07529	1.15898
H	-4.80148	0.76970	0.81703	H	-8.51680	-1.93622	1.63189
H	-5.63926	-3.22582	-0.43521	H	-3.93436	-6.06716	-1.46221
H	1.88362	-3.57231	2.92117	H	-5.01640	-4.74324	-1.90593
H	0.80023	-4.81757	2.27475	H	-4.87380	-5.25301	-0.20814
H	0.66793	-3.14465	1.69812	H	-1.32345	-4.11001	0.29731
H	0.97362	-4.02269	-0.75994	H	-1.85076	-5.72952	-0.20101
H	1.08758	-5.66938	-0.11278	H	-2.73061	-4.86446	1.07329
H	2.38368	-5.03257	-1.14205	H	-1.50851	-3.39540	-2.23733
H	3.83275	-5.10583	2.33107	H	-3.05102	-3.62747	-3.08014
H	4.01624	-5.72056	0.67254	H	-2.07089	-5.03369	-2.62585

C	2.26252	4.74861	0.02598
C	1.77961	5.99029	-0.41910
C	3.62240	4.46927	-0.18532
C	2.61636	6.90192	-1.06526
H	0.74220	6.26306	-0.25546
C	4.46405	5.37679	-0.82969
H	4.03212	3.52913	0.17746
C	3.96150	6.59847	-1.27769
H	2.21377	7.85523	-1.39746
H	5.51252	5.13013	-0.97496
H	4.61208	7.30964	-1.77916
C	-1.83750	4.37789	0.13121
C	-3.17050	3.98146	-0.07104
C	-1.59939	5.35957	1.10790
C	-4.21848	4.52451	0.67362
H	-3.39097	3.23902	-0.83459
C	-2.64346	5.90343	1.85929
H	-0.59347	5.72092	1.28964
C	-3.95799	5.48729	1.64910
H	-5.23797	4.19739	0.48605
H	-2.42466	6.66072	2.60763
H	-4.76941	5.91349	2.23255

$A_4C_1B_2$

Al	-0.04830	0.08602	0.75424
C	0.45538	0.41621	2.63201
O	-1.43444	-1.06163	0.80242
C	-2.70695	-0.99046	0.40850
C	-3.67057	-1.86789	0.92760
C	-5.00894	-1.79687	0.53413
C	-5.41759	-0.82953	-0.39094
C	-4.46641	0.05311	-0.91494
C	-3.12614	-0.02466	-0.53640
C	-2.09828	0.86861	-1.17395
N	-1.17240	1.57717	-0.20305
C	-0.37269	2.54711	-1.01615
C	0.81976	3.15004	-0.28149
N	1.75922	2.12137	0.17231
C	2.59429	1.61212	-0.94057
C	3.22220	0.27737	-0.63220
C	4.60488	0.10396	-0.62533
C	5.17866	-1.15077	-0.37983
C	4.34393	-2.24488	-0.13483
C	2.95557	-2.07402	-0.13649
C	2.37400	-0.82523	-0.37997
O	1.03009	-0.68882	-0.41967
C	-1.97077	2.31016	0.81824
C	2.60952	2.62422	1.25534
H	-0.02904	-0.32962	3.27680
H	0.15540	1.40113	3.01592
H	1.53649	0.33008	2.79841
H	-5.72024	-2.49660	0.95956
H	-4.79286	0.79782	-1.63686
H	-2.59519	1.63294	-1.78531
H	-1.44030	0.28960	-1.83422
H	-1.03914	3.35167	-1.35694
H	-0.03564	2.00965	-1.90556
H	1.29695	3.88627	-0.95371
H	0.48050	3.71103	0.59462
H	3.37038	2.35143	-1.20405
H	1.95095	1.49818	-1.81851
H	5.26491	0.94623	-0.81853
H	4.75452	-3.22997	0.05820
H	-2.64153	3.03075	0.33258
H	-1.30425	2.83921	1.49988

H	-2.56970	1.60484	1.39528
H	3.21156	3.49574	0.94067
H	3.29353	1.83680	1.57989
H	1.99326	2.91648	2.10977
H	-3.35261	-2.61489	1.64942
H	2.30217	-2.92255	0.04694
O	6.54986	-1.19630	-0.40199
O	-6.70427	-0.66209	-0.84100
C	-7.70037	-1.53328	-0.33196
H	-7.80926	-1.43823	0.75748
H	-8.63642	-1.23411	-0.81007
H	-7.49205	-2.58311	-0.58084
C	7.17379	-2.44599	-0.15661
H	8.24952	-2.26385	-0.21881
H	6.93413	-2.83321	0.84338
H	6.89446	-3.19704	-0.90843

$A_4C_1B_3$

Al	-0.23171	-1.02369	0.89104
C	0.48520	-0.60442	2.67865
O	-1.73409	-1.94685	1.22643
C	-2.96525	-1.92642	0.71049
C	-3.97652	-2.72670	1.26432
C	-5.27628	-2.72099	0.75597
C	-5.59740	-1.89640	-0.32843
C	-4.60036	-1.08707	-0.88341
C	-3.29655	-1.09177	-0.38152
C	-2.22953	-0.28892	-1.08173
N	-1.24184	0.45133	-0.19749
C	-0.36570	1.23220	-1.13064
C	0.90596	1.79832	-0.50544
N	1.80170	0.74618	-0.01630
C	2.48711	0.02953	-1.11753
C	2.92768	-1.36375	-0.73773
C	4.26477	-1.74875	-0.82846
C	4.67122	-3.05707	-0.53295
C	3.71266	-3.99433	-0.13855
C	2.36926	-3.61538	-0.04969
C	1.95479	-2.31232	-0.34495
O	0.64237	-1.98829	-0.30792
C	-1.93008	1.34697	0.81761
C	2.74374	1.22195	1.02739
H	-0.02182	-1.24285	3.41500
H	0.32950	0.43159	3.00945
H	1.56002	-0.80484	2.76484
H	-6.02466	-3.35896	1.21360
H	-4.86041	-0.45428	-1.72847
H	-2.69613	0.43614	-1.75835
H	-1.60765	-0.95402	-1.69375
H	-0.95014	2.04982	-1.56637
H	-0.10955	0.55706	-1.94883
H	1.38074	2.44870	-1.25996
H	0.65107	2.44812	0.33844
H	3.34230	0.61025	-1.49408
H	1.77990	-0.05189	-1.94769
H	5.01926	-1.03463	-1.14856
H	3.99044	-5.01551	0.09877
H	-1.14493	1.71023	1.48426
H	-2.57518	0.69317	1.40763
H	3.29147	0.34307	1.37891
H	2.13575	1.57629	1.86518
H	-3.72233	-3.36496	2.10580
H	1.61701	-4.34284	0.24279
C	-2.73057	2.51914	0.27996
C	-2.13283	3.77641	0.09991

C	-4.09921	2.38844	0.00088
C	-2.87288	4.86342	-0.36783
H	-1.08441	3.92259	0.34564
C	-4.84205	3.47375	-0.46655
H	-4.58789	1.43212	0.15963
C	-4.23015	4.71361	-0.65736
H	-2.39007	5.82855	-0.49663
H	-5.90180	3.35006	-0.67321
H	-4.80855	5.55994	-1.01816
C	3.73442	2.30809	0.62650
C	3.38660	3.66614	0.69290
C	5.02918	1.97873	0.19781
C	4.29173	4.66124	0.32091
H	2.40133	3.95041	1.05584
C	5.93913	2.96998	-0.17518
H	5.33220	0.93504	0.17370
C	5.57090	4.31517	-0.11975
H	4.00189	5.70706	0.38515
H	6.93783	2.69047	-0.50100
H	6.27855	5.08877	-0.40609
O	6.01336	-3.30996	-0.66291
O	-6.83758	-1.80476	-0.91028
C	6.46750	-4.62589	-0.39111
H	7.54678	-4.61411	-0.56190
H	6.27366	-4.91849	0.65011
H	6.00722	-5.36359	-1.06282
C	-7.87600	-2.61431	-0.38364
H	-8.08971	-2.37378	0.66715
H	-8.76235	-2.39708	-0.98481
H	-7.64185	-3.68466	-0.46597

$A_4C_2B_1$

Al	0.04728	0.26179	0.67436
C	0.34188	0.57209	2.62196
O	-1.22193	-0.87455	0.04581
C	-2.53982	-0.83753	-0.04019
C	-3.24497	-2.01849	-0.34638
C	-4.63117	-2.04151	-0.44764
C	-5.38380	-0.86904	-0.24908
C	-4.71319	0.31124	0.04374
C	-3.30858	0.34940	0.14980
C	-2.67721	1.61871	0.41629
N	-1.40358	1.79470	0.53190
C	-0.88174	3.14333	0.73996
C	-0.13170	3.63311	-0.52031
C	0.36932	2.48404	-1.40628
N	1.06841	1.42438	-0.66036
C	2.34275	1.27919	-0.89923
C	3.18967	0.23327	-0.41203
C	4.57799	0.31130	-0.67043
C	5.43295	-0.71480	-0.30639
C	4.88964	-1.86569	0.30665
C	3.52920	-1.96863	0.54979
C	2.63179	-0.92456	0.21821
O	1.35363	-1.04883	0.44798
H	-0.41254	1.18009	3.14025
H	1.32123	1.02872	2.83552
H	0.36042	-0.40528	3.12740
H	-5.12292	-2.97968	-0.68151
H	-5.29077	1.22067	0.19013
H	-3.34408	2.48378	0.51430
H	-0.18841	3.09214	1.58549
H	-1.68400	3.84202	1.00955
H	-0.48451	2.01412	-1.90571
H	1.02261	2.88492	-2.19044

H	2.82000	2.00395	-1.56712
H	4.98870	1.19039	-1.16086
H	5.53531	-2.68919	0.59328
H	-0.79476	4.25143	-1.13761
H	0.70864	4.26734	-0.21523
H	-2.66972	-2.92679	-0.49832
H	3.11993	-2.85989	1.01581
O	-6.75054	-0.79360	-0.32655
O	6.76462	-0.53600	-0.58419
C	-7.46824	-1.98214	-0.62117
H	-8.52376	-1.70022	-0.63150
H	-7.19845	-2.38975	-1.60511
H	-7.31275	-2.75461	0.14437
C	7.66622	-1.57432	-0.23408
H	8.65603	-1.22716	-0.54006
H	7.67432	-1.76289	0.84824
H	7.43888	-2.51084	-0.76182

$A_4C_6B_1$

Al	0.00344	0.04813	1.04592
O	-1.30708	-1.22998	0.86864
O	1.26636	-1.25467	0.76422
N	-1.28003	1.35691	0.05523
N	1.28991	1.36319	0.05093
C	-0.70344	2.63144	-0.12283
C	-1.40235	3.83791	-0.25020
C	-0.70327	5.03353	-0.39968
C	0.69524	5.03766	-0.40007
C	1.40140	3.84619	-0.25127
C	0.70972	2.63560	-0.12326
C	-2.49573	1.10492	-0.35326
C	-3.17710	-0.13732	-0.18655
C	-2.53705	-1.26223	0.42494
C	-3.28962	-2.45480	0.52644
C	-4.59276	-2.53733	0.06143
C	-5.21878	-1.42457	-0.54307
C	-4.50529	-0.24469	-0.66379
C	2.50933	1.11408	-0.34360
C	3.17883	-0.13787	-0.19093
C	2.51139	-1.27869	0.35784
C	3.24872	-2.48194	0.43193
C	4.56541	-2.55979	0.00445
C	5.21928	-1.43075	-0.53553
C	4.51913	-0.24022	-0.63255
C	0.05809	0.69988	2.91269
H	-2.48665	3.84888	-0.20200
H	-1.25063	5.96737	-0.48901
H	1.23700	5.97470	-0.49006
H	2.48571	3.86360	-0.20539
H	-3.04580	1.88882	-0.87924
H	-5.12681	-3.47552	0.16829
H	-4.98165	0.61378	-1.13044
H	3.07242	1.90294	-0.84821
H	5.08720	-3.50692	0.09052
H	5.01642	0.63057	-1.05218
H	-0.08782	-0.13531	3.61367
H	-0.72423	1.43755	3.14408
H	1.01791	1.16541	3.18041
H	2.75140	-3.35556	0.84200
H	-2.81406	-3.31572	0.98613
O	-6.50000	-1.42276	-1.02928
O	6.51492	-1.42172	-0.98160
C	7.25903	-2.62846	-0.90379
H	8.24776	-2.39642	-1.30642
H	7.36880	-2.97589	0.13255

H	6.80700	-3.42753	-1.50708
C	-7.25841	-2.61855	-0.92523
H	-8.23180	-2.39450	-1.36783
H	-6.79644	-3.44597	-1.48085
H	-7.40382	-2.92166	0.12055

$A_4C_8B_1$

Al	0.16007	-0.71414	-0.65759
C	0.56027	-0.60496	-2.60667
O	-1.12790	-1.76747	0.05223
C	-2.44731	-1.76014	0.04457
C	-3.14825	-2.90304	0.47774
C	-4.53680	-2.95229	0.48161
C	-5.29619	-1.84657	0.05234
C	-4.63002	-0.70417	-0.36839
C	-3.22072	-0.63891	-0.37732
C	-2.60407	0.59242	-0.79194
N	-1.32608	0.82213	-0.82015
C	-0.90154	2.10700	-1.27781
C	-1.09648	2.49132	-2.60872
C	-0.66466	3.74146	-3.05124
C	-0.03960	4.61615	-2.16223
C	0.14201	4.23842	-0.83219
C	-0.27717	2.98295	-0.36830
C	-0.17852	2.63482	1.07368
C	-0.72446	3.51232	2.02461
C	-0.67343	3.23883	3.39041
C	-0.06731	2.06512	3.83587
C	0.49318	1.18583	2.91098
C	0.44917	1.46144	1.53780
N	1.10347	0.53738	0.65016
C	2.40885	0.47350	0.80064
C	3.27656	-0.53857	0.29124
C	4.67690	-0.35590	0.39690
C	5.55560	-1.36734	0.05361
C	5.03097	-2.61102	-0.36968
C	3.66449	-2.81767	-0.45562
C	2.73778	-1.78897	-0.15589
O	1.45379	-1.99798	-0.23778
H	-0.31894	-0.42047	-3.23939
H	1.00290	-1.55175	-2.94606
H	1.28908	0.18081	-2.85641
H	-5.02697	-3.85906	0.81954
H	-5.21263	0.15473	-0.69149
H	-3.28446	1.39617	-1.08982
H	-1.57490	1.79795	-3.29329
H	-0.81439	4.02598	-4.08906
H	0.30525	5.58923	-2.50053
H	0.61981	4.92129	-0.13496
H	-1.21085	4.41833	1.67404
H	-1.11405	3.93489	4.09865
H	-0.02748	1.83086	4.89595
H	0.96769	0.26680	3.24239
H	2.87857	1.24584	1.41451
H	5.07473	0.59205	0.74979
H	5.69936	-3.42469	-0.63090
H	3.27153	-3.77751	-0.77684
H	-2.56783	-3.75972	0.80636
O	-6.66550	-1.80495	0.01876
O	6.89325	-1.08808	0.16637
C	-7.37895	-2.95725	0.44121
H	-7.17570	-3.20133	1.49288
H	-8.43767	-2.71051	0.33263
H	-7.15039	-3.83154	-0.18339
C	7.82248	-2.11046	-0.15954

H	7.73621	-2.42386	-1.20890
H	8.81233	-1.67633	-0.00022
H	7.71037	-2.98906	0.49053

$A_4C_{11}B_1$

Al	-0.06709	-0.99160	1.15504
N	1.35197	0.35068	0.42104
N	-1.18458	0.31021	0.05167
O	1.01003	-2.32808	0.52668
O	-1.51892	-2.14238	1.18693
C	2.91176	-3.65839	0.11014
C	4.26397	-3.78971	-0.18123
C	5.07378	-2.65142	-0.35714
C	4.49314	-1.39613	-0.24029
C	3.12407	-1.24686	0.06213
C	2.29884	-2.39631	0.25170
C	2.57871	0.08660	0.11518
C	0.80097	1.71477	0.42664
C	-0.56856	1.61592	-0.31249
C	-2.34817	0.05096	-0.47860
C	-3.11735	-1.13983	-0.29540
C	-4.36203	-1.24497	-0.95985
C	-5.16043	-2.36600	-0.81244
C	-4.71084	-3.41479	0.02037
C	-3.49416	-3.33074	0.67869
C	-2.65418	-2.19954	0.54625
C	0.09806	-0.51714	3.07693
H	4.68591	-4.78506	-0.27124
H	5.11334	-0.51540	-0.38661
H	3.26181	0.90079	-0.13881
H	0.58157	1.93937	1.47802
H	-0.36053	1.59449	-1.38869
H	-2.79940	0.80932	-1.12486
H	-4.70530	-0.43302	-1.59583
H	-5.31714	-4.30436	0.15518
H	0.01689	-1.43092	3.68462
H	1.04847	-0.03939	3.35566
H	-0.70645	0.14492	3.43280
H	-3.15627	-4.14186	1.31648
H	2.29202	-4.53988	0.24431
C	-1.46279	2.81058	-0.01610
C	-1.71768	3.76590	-1.00688
C	-2.03309	2.98496	1.25425
C	-2.51887	4.87795	-0.73651
H	-1.28216	3.64285	-1.99561
C	-2.83445	4.09310	1.52464
H	-1.85896	2.24396	2.03021
C	-3.07857	5.04438	0.53007
H	-2.70580	5.60998	-1.51772
H	-3.27112	4.21300	2.51261
H	-3.70431	5.90717	0.74232
C	1.68501	2.82348	-0.11707
C	2.14990	3.82458	0.74499
C	2.05741	2.87764	-1.47000
C	2.95641	4.86101	0.27133
H	1.87164	3.79505	1.79568
C	2.86644	3.91004	-1.94522
H	1.72282	2.10532	-2.15819
C	3.31620	4.90667	-1.07589
H	3.30206	5.63089	0.95593
H	3.14555	3.93533	-2.99525
H	3.94445	5.71188	-1.44720
O	-6.34872	-2.37178	-1.49764
O	6.41379	-2.68262	-0.64697
C	-7.19417	-3.50345	-1.36117

H	-7.51688	-3.65125	-0.32131
H	-8.07184	-3.29738	-1.97843
H	-6.71194	-4.42177	-1.72328
C	7.03972	-3.95049	-0.76779
H	6.60838	-4.54390	-1.58565
H	8.08926	-3.74660	-0.99299
H	6.98062	-4.52669	0.16577

$A_5C_1B_2$

Al	0.01248	0.52077	0.94376
C	0.20883	0.82320	2.88711
O	-1.39961	-0.66481	0.89427
C	-2.61330	-0.69136	0.40009
C	-3.53917	-1.67369	0.86189
C	-4.82714	-1.67386	0.35000
C	-5.21800	-0.72283	-0.60353
C	-4.32050	0.23973	-1.06859
C	-3.02085	0.25182	-0.58188
C	-2.00424	1.21237	-1.13322
N	-1.17951	1.93042	-0.08945
C	-0.36517	2.96843	-0.79112
C	0.84701	3.36949	0.03562
N	1.64726	2.17915	0.39648
C	2.44338	1.72293	-0.78203
C	3.15332	0.41743	-0.55056
C	4.52031	0.28738	-0.75872
C	5.11943	-0.96271	-0.60046
C	4.36944	-2.08568	-0.23350
C	3.00224	-1.97792	-0.01252
C	2.38379	-0.70796	-0.16729
O	1.07385	-0.60162	0.01864
C	-2.07316	2.59332	0.90442
C	2.56732	2.51802	1.50378
H	-0.50109	0.18126	3.42419
H	0.02005	1.85093	3.22639
H	1.20977	0.55624	3.25228
H	-5.55142	-2.40688	0.68631
H	-4.64807	0.95819	-1.81149
H	-2.50459	1.96734	-1.75281
H	-1.28355	0.68491	-1.77130
H	-0.98832	3.84545	-1.00720
H	-0.05445	2.55180	-1.75226
H	1.44753	4.10460	-0.52174
H	0.52927	3.85427	0.96333
H	3.16600	2.50501	-1.05923
H	1.75247	1.60506	-1.62261
H	5.12554	1.13810	-1.05039
H	4.87083	-3.03984	-0.12015
H	-2.78917	3.24746	0.39156
H	-1.47441	3.18687	1.59647
H	-2.62190	1.84401	1.47474
H	3.26186	3.31844	1.20686
H	3.14911	1.63872	1.78414
H	1.99486	2.84792	2.37140
N	-6.57202	-0.73386	-1.11286
O	-6.89435	0.12482	-1.94622
O	-7.35146	-1.59923	-0.69381
N	6.54809	-1.09708	-0.82051
O	7.05985	-2.21442	-0.68602
O	7.19021	-0.08616	-1.13250
C	-3.09050	-2.67591	1.89118
H	-2.23004	-3.25409	1.53337
H	-2.76845	-2.18100	2.81576
H	-3.89675	-3.37361	2.13759
C	2.16743	-3.16560	0.38514

H	1.68408	-3.00596	1.35672
H	1.36131	-3.34390	-0.33630
H	2.77966	-4.07019	0.45017

$A_5C_1B_3$

Al	-0.08711	-0.40546	1.01951
C	0.32940	0.11234	2.88123
O	-1.59941	-1.41360	1.31893
C	-2.76064	-1.57604	0.73178
C	-3.69979	-2.49323	1.29245
C	-4.93422	-2.64978	0.68349
C	-5.26118	-1.91202	-0.46301
C	-4.35487	-1.00756	-1.01757
C	-3.10687	-0.83639	-0.43072
C	-2.08937	0.05525	-1.09509
N	-1.23175	0.90030	-0.17766
C	-0.37428	1.76180	-1.05058
C	0.90669	2.19347	-0.35274
N	1.67972	1.02279	0.11563
C	2.32774	0.33304	-1.03908
C	2.88928	-1.02389	-0.70262
C	4.19912	-1.36044	-1.02306
C	4.65769	-2.65447	-0.77462
C	3.82161	-3.62466	-0.21221
C	2.50685	-3.31518	0.11222
C	2.03237	-1.99856	-0.13526
O	0.76324	-1.71493	0.13516
C	-2.05661	1.73904	0.79217
C	2.68995	1.41596	1.15574
H	-0.38080	-0.39811	3.54398
H	0.25783	1.18342	3.11360
H	1.33375	-0.20458	3.19223
H	-5.66366	-3.34153	1.08883
H	-4.63271	-0.45491	-1.90802
H	-2.59084	0.71568	-1.81130
H	-1.37681	-0.55795	-1.66162
H	-0.93698	2.64018	-1.37993
H	-0.14449	1.18467	-1.94831
H	1.48941	2.82508	-1.03793
H	0.66895	2.80612	0.52228
H	3.10975	0.97163	-1.46972
H	1.56315	0.20418	-1.81040
H	4.86565	-0.63604	-1.47575
H	4.21273	-4.61942	-0.03286
H	-1.34316	2.17160	1.49691
H	-2.67257	1.03429	1.35224
H	3.16117	0.49145	1.49727
H	2.12200	1.81847	1.99608
C	3.76001	2.41411	0.74090
C	5.03172	1.97913	0.33925
C	3.51856	3.79597	0.79326
C	6.02277	2.89242	-0.02561
H	5.25458	0.91582	0.32861
C	4.50503	4.71268	0.42763
H	2.55463	4.16090	1.13983
C	5.75970	4.26251	0.01143
H	7.00141	2.53157	-0.33062
H	4.29749	5.77839	0.47886
H	6.53056	4.97512	-0.26930
C	-2.91663	2.84178	0.20601
C	-2.43397	4.15877	0.14732
C	-4.22584	2.59194	-0.23213
C	-3.22350	5.19181	-0.35926
H	-1.43547	4.38222	0.51656
C	-5.01797	3.62278	-0.73965

H	-4.63377	1.58845	-0.16100
C	-4.51754	4.92421	-0.80932
H	-2.83152	6.20475	-0.39310
H	-6.03036	3.40868	-1.07107
H	-5.13628	5.72706	-1.20082
C	-3.31785	-3.26586	2.52619
H	-2.41802	-3.86877	2.35436
H	-3.08561	-2.59343	3.36123
H	-4.12694	-3.93394	2.83693
C	1.57825	-4.33744	0.71142
H	1.20784	-4.01419	1.69190
H	0.69424	-4.48914	0.08071
H	2.08340	-5.30022	0.83508
N	-6.55870	-2.08391	-1.08018
O	-6.82770	-1.40774	-2.08314
O	-7.34505	-2.89763	-0.57903
N	6.02916	-2.99625	-1.10699
O	6.41274	-4.15265	-0.89781
O	6.75385	-2.11324	-1.58348

$A_5C_2B_2$

Al	-0.07833	0.53693	0.86034
C	-0.28820	1.04500	2.76370
O	-1.36789	-0.79201	0.81996
C	-2.60921	-0.82622	0.40479
C	-3.54305	-1.72663	0.99769
C	-4.85263	-1.72282	0.54300
C	-5.25411	-0.85340	-0.48223
C	-4.34483	0.02015	-1.08238
C	-3.02624	0.02707	-0.65146
C	-1.98480	0.86147	-1.33911
N	-1.18028	1.77964	-0.43099
C	-0.34196	2.60966	-1.35650
C	1.27835	3.47126	0.54824
N	1.82620	2.10524	0.69343
C	2.75205	1.77449	-0.42301
C	3.27844	0.36288	-0.35832
C	4.63435	0.10906	-0.52669
C	5.09534	-1.20716	-0.52966
C	4.21700	-2.28185	-0.35717
C	2.85892	-2.05484	-0.17675
C	2.37823	-0.71601	-0.17323
O	1.07486	-0.51867	-0.02126
C	-2.11212	2.65086	0.33864
C	2.59377	2.05121	1.95922
H	-1.29662	0.76039	3.09032
H	-0.15931	2.10422	3.01680
H	0.40474	0.48229	3.40525
H	-5.58657	-2.39095	0.97906
H	-4.67764	0.66871	-1.88481
H	-2.45947	1.48486	-2.10598
H	-1.24944	0.21893	-1.83812
H	-1.01223	3.06006	-2.10144
H	0.29364	1.90395	-1.89584
H	2.08796	4.21628	0.62262
H	0.63058	3.63044	1.41529
H	3.59596	2.48262	-0.42544
H	2.22014	1.91716	-1.36661
H	5.33936	0.92124	-0.66208
H	4.61024	-3.29178	-0.36183
H	-2.73418	3.23931	-0.34788
H	-1.54734	3.32422	0.98252
H	-2.76148	2.03573	0.96184
H	3.48211	2.69949	1.90268
H	2.92346	1.03002	2.15888

H	1.97132	2.38580	2.78793
N	-6.62920	-0.86131	-0.92997
O	-6.95899	-0.07787	-1.83200
O	-7.41931	-1.64842	-0.39272
N	6.51319	-1.46249	-0.70895
O	6.90202	-2.63612	-0.70983
O	7.26963	-0.49369	-0.85281
C	-3.08145	-2.64866	2.09407
H	-2.25900	-3.28921	1.75302
H	-2.70040	-2.08688	2.95569
H	-3.89837	-3.29099	2.43713
C	1.88765	-3.18893	0.01527
H	1.35808	-3.10289	0.97162
H	1.11669	-3.18896	-0.76438
H	2.40320	-4.15386	-0.00858
C	0.49397	3.74172	-0.74527
H	1.17945	4.05553	-1.54135
H	-0.14519	4.61091	-0.55288

$A_5C_2B_3$

Al	-0.19830	-0.37783	0.83932
C	0.15424	0.37717	2.62119
O	-1.52166	-1.57014	1.18536
C	-2.79267	-1.61395	0.84169
C	-3.69070	-2.43077	1.58619
C	-5.02539	-2.46604	1.21253
C	-5.47907	-1.71029	0.12280
C	-4.60556	-0.91059	-0.61486
C	-3.26221	-0.85890	-0.26230
C	-2.27968	-0.11298	-1.12814
N	-1.31442	0.83513	-0.42765
C	-0.46937	1.40868	-1.53854
C	1.52436	2.50967	-0.10596
N	2.15181	1.20865	0.14986
C	2.79554	0.60609	-1.03164
C	3.07856	-0.87072	-0.85485
C	4.34670	-1.37258	-1.12917
C	4.59845	-2.73943	-1.01349
C	3.59489	-3.62823	-0.62093
C	2.31708	-3.15795	-0.34369
C	2.05028	-1.76682	-0.46308
O	0.80431	-1.34711	-0.25256
C	-2.04674	1.88908	0.38416
C	3.06778	1.26854	1.32316
H	-0.70995	0.16491	3.26451
H	0.32582	1.45856	2.66194
H	1.01873	-0.10119	3.09999
H	-5.73389	-3.07646	1.76048
H	-4.98433	-0.34307	-1.45658
H	-2.82501	0.46050	-1.88367
H	-1.63924	-0.82878	-1.65729
H	-1.14696	1.75403	-2.32861
H	0.07466	0.55400	-1.94378
H	2.26703	3.29715	-0.31600
H	1.03253	2.79753	0.83094
H	3.72872	1.12547	-1.29944
H	2.12405	0.73432	-1.88436
H	5.14514	-0.71015	-1.44224
H	3.82344	-4.68431	-0.53712
H	-1.28215	2.58556	0.73483
H	-2.43433	1.37579	1.26756
H	3.38792	0.24294	1.52897
H	2.46530	1.59506	2.17361
N	-6.87972	-1.75680	-0.24563
O	-7.25823	-1.07198	-1.20534

O	-7.63735	-2.47694	0.41644
N	5.92992	-3.24440	-1.30263
O	6.12983	-4.46016	-1.20283
O	6.80412	-2.43462	-1.63525
C	-3.16674	-3.23206	2.74769
H	-2.37590	-3.92217	2.42955
H	-2.72385	-2.58343	3.51347
H	-3.96620	-3.81651	3.21306
C	1.21087	-4.09326	0.06776
H	0.79441	-3.81935	1.04437
H	0.37695	-4.06085	-0.64367
H	1.57327	-5.12411	0.12555
C	0.50389	2.56343	-1.25616
H	1.03627	2.69096	-2.20724
H	-0.05006	3.49853	-1.11827
C	4.29231	2.16826	1.21253
C	5.52614	1.66300	0.77620
C	4.22381	3.52265	1.57495
C	6.64907	2.48741	0.68052
H	5.61449	0.60850	0.52840
C	5.34272	4.35165	1.48044
H	3.28814	3.92881	1.95277
C	6.55892	3.83656	1.02719
H	7.59543	2.07208	0.34399
H	5.26727	5.39649	1.77091
H	7.43271	4.47902	0.95719
C	-3.19294	2.63738	-0.27898
C	-4.50479	2.39231	0.15495
C	-2.99663	3.62227	-1.26049
C	-5.58756	3.08568	-0.38714
H	-4.67975	1.65191	0.92966
C	-4.07759	4.31326	-1.81000
H	-1.99571	3.87233	-1.59561
C	-5.37741	4.04474	-1.37841
H	-6.59302	2.87345	-0.03461
H	-3.90025	5.06968	-2.56984
H	-6.21822	4.58449	-1.80528

$A_5C_5B_2$

Al	0.06649	0.25212	1.11851
C	0.29613	0.76673	3.01553
O	-1.37234	-0.90794	1.29181
C	-2.48310	-1.14690	0.64140
C	-3.37669	-2.15132	1.12151
C	-4.57151	-2.36265	0.45267
C	-4.90501	-1.59760	-0.67456
C	-4.03989	-0.61327	-1.15545
C	-2.82918	-0.39374	-0.51354
C	-1.84143	0.58968	-1.08208
N	-1.15888	1.49613	-0.07300
C	-0.37870	2.53201	-0.86879
C	0.93237	2.96074	-0.16600
N	1.65630	1.73062	0.30750
C	2.34495	1.11190	-0.87760
C	3.05573	-0.17767	-0.57412
C	4.38724	-0.36946	-0.91876
C	4.98034	-1.61095	-0.68594
C	4.25962	-2.66277	-0.10788
C	2.92976	-2.49082	0.25317
C	2.31857	-1.22826	0.02239
O	1.04607	-1.05720	0.34601
C	-2.18008	2.08680	0.84209
C	2.69229	2.03883	1.32323
H	-0.45370	0.24086	3.61998
H	0.18479	1.83738	3.23525

H	1.27723	0.47097	3.41222
H	-5.26781	-3.11844	0.79759
H	-4.32154	-0.03837	-2.03057
H	-2.34721	1.21545	-1.82608
H	-1.03349	0.05535	-1.59806
H	-0.08584	1.98263	-1.76597
H	1.54587	3.42206	-0.95769
H	3.05657	1.83863	-1.29503
H	1.58523	0.92555	-1.64254
H	4.96898	0.42471	-1.37260
H	4.75595	-3.61193	0.05794
H	-2.96422	2.60415	0.28155
H	-1.70151	2.78138	1.52875
H	-2.63972	1.29214	1.42717
H	3.43670	2.74666	0.93046
H	3.20658	1.11636	1.59516
H	2.23877	2.45099	2.22112
C	-1.17240	3.76226	-1.37132
H	-0.63341	4.11884	-2.25918
H	-2.15892	3.45127	-1.73401
C	-1.29805	4.93791	-0.38753
H	-1.69303	5.80580	-0.92943
H	-2.02465	4.71478	0.40022
C	0.73426	4.06693	0.88668
H	0.15387	3.70796	1.74391
H	1.70662	4.37983	1.27666
C	0.05237	5.28796	0.24654
H	0.72124	5.70294	-0.52138
H	-0.07520	6.07121	1.00277
C	-2.99515	-2.94609	2.34127
H	-2.04475	-3.47232	2.19111
H	-2.85416	-2.29491	3.21283

H	-3.76448	-3.68532	2.58488
C	2.12591	-3.59872	0.87912
H	1.76117	-3.31336	1.87342
H	1.23844	-3.83298	0.27928
H	2.72453	-4.50906	0.98046
N	-6.16428	-1.82493	-1.34753
O	-6.43935	-1.12738	-2.33456
O	-6.91580	-2.70578	-0.90922
N	6.37088	-1.81116	-1.04893
O	6.87862	-2.91896	-0.83953
O	6.98761	-0.86305	-1.55190

$A_5C_3B_3$

Al	-0.05601	0.30706	1.31648
C	0.19284	1.35654	2.98546
O	-1.58138	-0.54925	1.95478
C	-2.51760	-1.18758	1.29515
C	-3.32623	-2.14300	1.98407
C	-4.33309	-2.79974	1.29717
C	-4.56565	-2.51985	-0.05690
C	-3.79607	-1.57617	-0.73726
C	-2.77320	-0.90616	-0.07436
C	-1.90438	0.03996	-0.87675
N	-1.27424	1.22309	-0.15327
C	-0.61304	2.02814	-1.24899
C	1.65001	2.56143	0.04613
N	1.86066	1.11470	0.32907
C	2.24736	0.35395	-0.89645
C	2.47860	-1.12147	-0.67183
C	3.51381	-1.76871	-1.33796
C	3.68052	-3.14500	-1.18781
C	2.82587	-3.89173	-0.36900
C	1.78512	-3.27113	0.30678

C	1.59794	-1.86772	0.14984	H	7.35167	0.69921	-0.19942
O	0.58257	-1.30095	0.77999	H	6.10490	4.44211	1.51662
C	-2.31802	2.04878	0.60026	H	7.86041	3.05334	0.42842
C	2.93780	0.98361	1.39196	C	-3.42927	2.71375	-0.19820
H	-0.79886	1.51811	3.42638	C	-3.41978	4.10225	-0.40332
H	0.68587	2.33516	2.94394	C	-4.53860	1.98850	-0.66349
H	0.74706	0.75984	3.72547	C	-4.45915	4.74102	-1.08200
H	-4.95435	-3.53381	1.79724	H	-2.60114	4.69561	-0.01061
H	-4.00693	-1.37880	-1.78263	C	-5.57585	2.62208	-1.34862
H	-2.48517	0.42739	-1.72147	H	-4.60568	0.92349	-0.47081
H	-1.06111	-0.51759	-1.29977	C	-5.53718	4.00031	-1.56698
H	-1.40080	2.58883	-1.76457	H	-4.42788	5.81815	-1.22339
H	-0.26108	1.28921	-1.96914	H	-6.42196	2.03698	-1.69888
H	2.59714	3.01351	-0.27334	H	-6.34795	4.49399	-2.09590
H	1.40639	2.99989	1.01662	C	0.08863	4.39484	-0.48570
H	3.14093	0.80253	-1.34947	H	-0.60367	4.84955	-1.20138
H	1.43701	0.46936	-1.61788	H	-0.40109	4.34996	0.49161
H	4.19291	-1.21751	-1.97833	H	0.94786	5.06910	-0.38546
H	2.98836	-4.95858	-0.26898	C	1.19934	3.24517	-2.37301
H	-1.75527	2.80870	1.14440	H	1.97771	4.01343	-2.31062
H	-2.74380	1.37743	1.34427	H	1.65518	2.34141	-2.78909
H	3.03077	-0.08250	1.60854	H	0.44635	3.59631	-3.08866
H	2.53942	1.46413	2.28400	C	-3.05150	-2.41108	3.43946
C	0.56400	3.00944	-0.97756	H	-2.02311	-2.76020	3.59168
C	4.30941	1.56571	1.08786	H	-3.16077	-1.49923	4.03939
C	5.31747	0.78923	0.49720	H	-3.73597	-3.16785	3.83514
C	4.62019	2.88424	1.45799	C	0.84654	-4.04524	1.19311
C	6.58576	1.32088	0.25660	H	0.84994	-3.65089	2.21645
H	5.11486	-0.24593	0.23911	H	-0.18803	-3.97424	0.83692
C	5.88617	3.41997	1.21888	H	1.12592	-5.10257	1.22956
H	3.86699	3.49320	1.95257	N	-5.62666	-3.20778	-0.75953
C	6.87218	2.64001	0.61144	O	-6.29681	-4.04386	-0.13970

O	-5.82101	-2.93029	-1.95166
N	4.76495	-3.80867	-1.88702
O	4.89618	-5.02944	-1.74149
O	5.51127	-3.12282	-2.59775

$A_5C_6B_2$

Al	0.00115	0.22701	1.36648
C	0.14595	0.71403	3.27425
O	-1.38465	-0.96920	1.21130
C	-2.48736	-1.10322	0.51334
C	-3.32670	-2.23198	0.75094
C	-4.50609	-2.35486	0.03359
C	-4.87469	-1.38073	-0.90473
C	-4.06435	-0.27067	-1.14439
C	-2.86908	-0.13264	-0.45120
C	-1.95333	1.02339	-0.75616
N	-1.33706	1.67177	0.46914
N	1.45908	1.66731	0.46459
C	2.04687	1.05077	-0.78782
C	2.88070	-0.17051	-0.52371
C	4.14552	-0.30853	-1.07991
C	4.86220	-1.48561	-0.86183
C	4.33064	-2.52453	-0.08646
C	3.07437	-2.40406	0.48989
C	2.33498	-1.20664	0.27418
O	1.13800	-1.08225	0.80948
C	-2.38666	1.99922	1.48559
C	2.53517	1.93896	1.46201
H	-0.68973	0.27890	3.83876
H	0.14115	1.79196	3.48598
H	1.06236	0.31208	3.72780
H	-5.16123	-3.20334	0.19431
H	-4.37646	0.46755	-1.87442
H	-2.50299	1.78647	-1.31857
H	-1.11165	0.70498	-1.38519
H	2.64817	1.80656	-1.30637
H	1.19605	0.80951	-1.43651
H	4.58055	0.47773	-1.68645
H	4.91805	-3.42361	0.06046
H	-3.22142	2.54405	1.03390
H	-1.93520	2.60854	2.26877
H	-2.76698	1.07912	1.92603
H	3.37720	2.47424	1.00870
H	2.90506	0.99486	1.86194
H	2.12254	2.53236	2.27751
N	-6.11848	-1.52004	-1.63194
O	-6.42510	-0.63891	-2.44715
O	-6.82294	-2.51226	-1.40724
N	6.18131	-1.63045	-1.44488
O	6.79997	-2.68242	-1.24385
O	6.63164	-0.69351	-2.11770
C	-2.90560	-3.25368	1.77259
H	-1.92340	-3.67619	1.52937
H	-2.81135	-2.80535	2.76944
H	-3.62994	-4.07188	1.83083
C	2.47539	-3.49935	1.33048
H	2.27309	-3.15356	2.35174
H	1.51467	-3.83312	0.92064
H	3.14627	-4.36201	1.38651
C	-0.63215	2.88733	0.06629
C	0.76519	2.89274	0.08988
C	1.45815	4.05369	-0.27640
C	0.76608	5.19796	-0.66130
C	-0.62968	5.18868	-0.69404
C	-1.32402	4.03778	-0.33349

H	2.54301	4.06466	-0.26086
H	1.31591	6.09237	-0.93870
H	-1.17800	6.07542	-0.99760
H	-2.40897	4.03952	-0.35886

$A_5C_6B_3$

Al	0.00199	-0.73089	0.83532
C	0.50041	-0.44564	2.72959
O	-1.47533	-1.80127	1.06902
C	-2.65063	-1.94826	0.51007
C	-3.50928	-2.99880	0.95270
C	-4.76030	-3.13157	0.37307
C	-5.18053	-2.24088	-0.62536
C	-4.35257	-1.20555	-1.05994
C	-3.08787	-1.05676	-0.50180
C	-2.15870	-0.00733	-1.05637
N	-1.27600	0.73766	-0.06014
N	1.59032	0.76696	-0.14818
C	2.42710	0.02789	-1.16286
C	3.03340	-1.25764	-0.67177
C	4.39088	-1.52226	-0.81055
C	4.88299	-2.77752	-0.45217
C	4.03483	-3.77924	0.03333
C	2.67346	-3.54112	0.17385
C	2.16730	-2.26130	-0.17911
O	0.86531	-2.02312	-0.07866
C	-2.03049	1.29476	1.15642
C	2.41241	1.30371	1.01035
H	-0.15522	-1.09318	3.32643
H	0.39276	0.56659	3.13902
H	1.52989	-0.75950	2.94817
H	-5.43130	-3.92254	0.68772
H	-4.70375	-0.53308	-1.83474
H	-2.75260	0.71428	-1.61989
H	-1.45191	-0.45764	-1.76532
H	3.21440	0.68263	-1.55048
H	1.74844	-0.19310	-1.99387
H	5.06923	-0.77922	-1.21262
H	4.45524	-4.74225	0.29923
H	-1.24683	1.65324	1.82624
H	-2.49305	0.42804	1.62999
H	1.68903	1.69324	1.72639
H	2.87380	0.42443	1.46345
C	-0.56419	1.79198	-0.80054
C	0.83825	1.80199	-0.84392
C	1.48853	2.77051	-1.62625
C	0.77090	3.73123	-2.33008
C	-0.62139	3.72905	-2.27322
C	-1.27938	2.76104	-1.52216
H	2.56850	2.76735	-1.69050
H	1.29956	4.47011	-2.92518
H	-1.19981	4.46807	-2.81962
H	-2.36102	2.76152	-1.50571
N	-6.49536	-2.39032	-1.21161
O	-6.84691	-1.58218	-2.08254
O	-7.21291	-3.31794	-0.81666
N	6.30262	-3.04779	-0.59281
O	6.71798	-4.17274	-0.29309
O	7.03384	-2.13824	-1.00504
C	-3.02671	-3.93169	2.03036
H	-2.10106	-4.43814	1.73144
H	-2.79813	-3.38747	2.95506
H	-3.77952	-4.69306	2.25633
C	1.73035	-4.59752	0.68418
H	1.22842	-4.27346	1.60393

H	0.93839	-4.80650	-0.04474
H	2.26258	-5.53050	0.89310
C	-3.05695	2.39193	0.96785
C	-2.70891	3.72537	1.23173
C	-4.38089	2.10492	0.60372
C	-3.64913	4.74866	1.11131
H	-1.69284	3.96279	1.53709
C	-5.32188	3.12783	0.47485
H	-4.68092	1.07580	0.43195
C	-4.95779	4.45250	0.72489
H	-3.36099	5.77479	1.32329
H	-6.34273	2.88634	0.19144
H	-5.69266	5.24748	0.63079
C	3.47464	2.35845	0.76182
C	4.78948	2.00363	0.42562
C	3.17922	3.71806	0.94753
C	5.77139	2.97956	0.24394
H	5.05445	0.95492	0.32899
C	4.15828	4.69608	0.77250
H	2.17315	4.01187	1.23699
C	5.45677	4.32913	0.41196
H	6.78429	2.68265	-0.01445
H	3.90946	5.74303	0.92490
H	6.22188	5.08906	0.27762

$A_5C_{11}B_1$

Al	-0.04057	-0.72982	1.09743
N	1.37073	0.62425	0.43692
N	-1.17035	0.60555	0.05732
O	1.08150	-2.06024	0.48366
O	-1.45342	-1.94535	0.97464
C	2.95377	-3.40732	0.02996
C	4.30069	-3.48915	-0.26883
C	5.08188	-2.32727	-0.41652
C	4.52440	-1.06917	-0.27168
C	3.16216	-0.94876	0.03909
C	2.35392	-2.11945	0.20059
C	2.60030	0.37796	0.13269
C	0.81957	1.99265	0.48481
C	-0.54507	1.92322	-0.26279
C	-2.35892	0.39095	-0.42511
C	-3.13149	-0.80881	-0.28174
C	-4.40931	-0.84738	-0.86480
C	-5.17644	-1.98960	-0.74236
C	-4.69696	-3.11830	-0.04269
C	-3.44675	-3.11873	0.54024
C	-2.62588	-1.94699	0.42944
C	0.03135	-0.36924	3.04142
H	4.77619	-4.45521	-0.39376
H	5.14131	-0.18688	-0.40074
H	3.27850	1.20429	-0.08966
H	0.60047	2.18161	1.54294
H	-0.33173	1.92445	-1.33765
H	-2.83777	1.19050	-0.99607
H	-4.79619	0.01018	-1.40384
H	-5.33453	-3.99225	0.02795
H	0.05035	-1.32168	3.59184
H	0.91253	0.19851	3.37204
H	-0.85331	0.16956	3.41253
C	-1.43668	3.11249	0.05766
C	-1.72851	4.06044	-0.92995
C	-1.97223	3.28588	1.34340
C	-2.53283	5.16554	-0.64114
H	-1.31956	3.93818	-1.93005
C	-2.77689	4.38695	1.63157

H	-1.76801	2.55224	2.11937
C	-3.05821	5.33113	0.64005
H	-2.74879	5.89246	-1.41930
H	-3.18655	4.50731	2.63081
H	-3.68619	6.18846	0.86651
C	1.71198	3.11165	-0.02051
C	2.19038	4.07346	0.87800
C	2.07926	3.21175	-1.37217
C	3.00687	5.11772	0.44040
H	1.91528	4.00743	1.92784
C	2.89839	4.25221	-1.81077
H	1.73290	2.47108	-2.08887
C	3.36234	5.20961	-0.90557
H	3.36401	5.85713	1.15189
H	3.17414	4.31463	-2.85998
H	3.99865	6.02083	-1.24856
C	-2.91143	-4.30736	1.29038
H	-1.97509	-4.66569	0.84646
H	-2.68241	-4.04770	2.33095
H	-3.63339	-5.12936	1.28977
C	2.09888	-4.63472	0.19119
H	1.65821	-4.68136	1.19416
H	1.26134	-4.62959	-0.51651
H	2.68518	-5.54379	0.02773
N	-6.49723	-2.02894	-1.34322
O	-6.89228	-1.02965	-1.95548
O	-7.16470	-3.06114	-1.21411
N	6.49367	-2.44949	-0.73517
O	7.15649	-1.41358	-0.86376
O	6.96525	-3.58447	-0.86379

A$_5$C$_1$B$_1$

Al	0.00879	0.80859	0.81513
N	1.38488	2.28629	0.35581
N	-1.14315	2.19985	-0.17854
O	1.26903	-0.39405	0.22504
O	-1.32050	-0.43449	0.43296
Cl	2.44250	-3.07328	-0.13117
Cl	7.08964	-0.33960	-0.77117
Cl	-2.74673	-3.00763	0.71276
Cl	-7.04200	-0.14248	-0.97227
C	3.29380	-1.54465	-0.18718
C	4.65938	-1.54309	-0.41873
C	5.35313	-0.32585	-0.47261
C	4.68731	0.87028	-0.30065
C	3.29904	0.87336	-0.05359
C	2.55380	-0.34486	0.01474
C	2.63458	2.14678	0.06973
C	0.76079	3.60299	0.36538
C	-0.48783	3.47562	-0.51183
C	-2.39769	2.06712	-0.48289
C	-3.18101	0.87594	-0.31795
C	-4.55125	0.93149	-0.65823
C	-5.32966	-0.20082	-0.55987
C	-4.77120	-1.41847	-0.13574
C	-3.42873	-1.48296	0.18943
C	-2.57299	-0.34233	0.12323
C	-0.10854	1.05385	2.77386
H	5.18566	-2.48067	-0.55787
H	5.22329	1.81326	-0.35183
H	3.24517	3.03787	-0.11027
H	1.43102	4.38817	-0.00427
H	0.47120	3.84322	1.39583
H	-1.16665	4.32396	-0.36882
H	-0.18635	3.44878	-1.56680

H	-2.92048	2.92349	-0.92065
H	-4.97885	1.87046	-0.99661
H	-5.38896	-2.30657	-0.06306
H	-0.15072	0.07386	3.27129
H	0.74304	1.59241	3.21394
H	-1.01754	1.58979	3.08641

$A_6C_1B_2$

Al	0.03966	0.53200	0.86906
C	0.34165	0.63694	2.81484
O	-1.41214	-0.56940	0.75190
C	-2.60798	-0.51392	0.21435
C	-3.61150	-1.45734	0.54297
C	-4.88948	-1.40243	-0.00369
C	-5.19805	-0.37894	-0.89749
C	-4.24724	0.57521	-1.24575
C	-2.96245	0.50408	-0.70490
C	-1.89989	1.47371	-1.14716
N	-1.07923	2.08005	-0.03017
C	-0.23996	3.15876	-0.63621
C	0.96163	3.50530	0.23094
N	1.77985	2.30861	0.49876
C	2.58345	1.94599	-0.69983
C	3.23055	0.59075	-0.58708
C	4.60886	0.43318	-0.73596
C	5.16846	-0.84081	-0.68870
C	4.37422	-1.96573	-0.48728
C	2.99968	-1.79834	-0.33169
C	2.39004	-0.52624	-0.37620
O	1.07190	-0.38978	-0.26698
C	-1.97246	2.67214	1.00682
C	2.68082	2.55934	1.63895
H	-0.30787	-0.09574	3.31034
H	0.11915	1.61294	3.26807
H	1.37284	0.38883	3.09789
H	-5.63115	-2.14385	0.27053
H	-4.50044	1.36712	-1.94426
H	-2.35970	2.29235	-1.71539
H	-1.17889	0.97521	-1.80733
H	-0.85714	4.05068	-0.80427
H	0.08727	2.80506	-1.61669
H	1.54621	4.29886	-0.26270
H	0.63108	3.90761	1.19315
H	3.34705	2.71822	-0.88345
H	1.91299	1.94178	-1.56477
H	5.24081	1.30124	-0.89701
H	4.81044	-2.95706	-0.44704
H	-2.66470	3.38894	0.54787
H	-1.37025	3.18410	1.75864
H	-2.54731	1.88762	1.49899
H	3.36817	3.39496	1.42896
H	3.27354	1.66663	1.84584
H	2.09481	2.79893	2.52773
Cl	-3.22926	-2.73782	1.68179
Cl	-6.82094	-0.29069	-1.58889
Cl	1.99998	-3.21666	-0.07472
Cl	6.91216	-1.03586	-0.88313

$A_6C_1B_3$

Al	-0.13669	-0.56539	0.77731
C	0.45541	-0.27892	2.63300
O	-1.70371	-1.44889	1.01214
C	-2.88894	-1.41681	0.44347
C	-3.94502	-2.23566	0.91109
C	-5.21224	-2.21316	0.33910
C	-5.45339	-1.34764	-0.72584

C	-4.44927	-0.51677	-1.21189
C	-3.17590	-0.54780	-0.63633
C	-2.06904	0.27307	-1.24979
N	-1.11116	0.95254	-0.29184
C	-0.18738	1.77086	-1.14197
C	1.09696	2.18280	-0.43491
N	1.87664	1.01423	0.00248
C	2.54571	0.35047	-1.14454
C	2.96129	-1.07237	-0.85821
C	4.28510	-1.48145	-1.02940
C	4.63473	-2.81433	-0.82873
C	3.68197	-3.75728	-0.45765
C	2.36148	-3.34375	-0.29237
C	1.96384	-2.00430	-0.48788
O	0.68535	-1.64440	-0.37928
C	-1.82701	1.80592	0.74738
C	2.83400	1.35183	1.09620
H	-0.16478	-0.90950	3.28320
H	0.35615	0.74855	3.00844
H	1.49823	-0.57550	2.79995
H	-5.99524	-2.85732	0.72225
H	-4.65113	0.14917	-2.04544
H	-2.49952	1.03576	-1.90850
H	-1.43488	-0.37304	-1.86922
H	-0.71462	2.66157	-1.49836
H	0.04476	1.16817	-2.02221
H	1.66433	2.83916	-1.11356
H	0.86149	2.78071	0.45029
H	3.41142	0.93636	-1.48407
H	1.83354	0.33331	-1.97458
H	5.03832	-0.76223	-1.33484
H	3.95346	-4.79440	-0.29839

H	-1.06605	2.09430	1.47583
H	-2.52232	1.13839	1.25914
H	3.31408	0.41552	1.39245
H	2.23095	1.68450	1.94455
Cl	-3.63988	-3.32127	2.25609
Cl	-7.05855	-1.30208	-1.46044
Cl	1.15678	-4.53474	0.16499
Cl	6.31237	-3.31879	-1.04773
C	3.89747	2.39680	0.78985
C	5.17653	2.01737	0.35652
C	3.63707	3.76502	0.96295
C	6.15645	2.97262	0.07939
H	5.41329	0.96137	0.25682
C	4.61219	4.72427	0.68653
H	2.66615	4.08348	1.33573
C	5.87462	4.33035	0.23793
H	7.14191	2.65457	-0.25093
H	4.39010	5.77827	0.83265
H	6.63690	5.07558	0.02701
C	-2.55282	3.04293	0.25459
C	-1.90985	4.29094	0.25760
C	-3.89229	2.98435	-0.15790
C	-2.57494	5.44280	-0.16373
H	-0.88293	4.36439	0.60840
C	-4.56038	4.13485	-0.58014
H	-4.42083	2.03622	-0.13237
C	-3.90240	5.36599	-0.58917
H	-2.05959	6.39941	-0.15070
H	-5.59926	4.06927	-0.89200
H	-4.42444	6.26194	-0.91401

$A_6C_2B_2$

Al	-0.12006	0.32597	0.60830

C	-0.03106	0.54029	2.55872
O	-1.37907	-0.93116	0.28869
C	-2.66447	-0.75807	0.04388
C	-3.65179	-1.63858	0.53799
C	-5.00472	-1.45283	0.26686
C	-5.39541	-0.36487	-0.51107
C	-4.45530	0.52643	-1.02091
C	-3.10035	0.32589	-0.75371
C	-2.04755	1.20663	-1.36861
N	-1.10859	1.87962	-0.37859
C	-0.16822	2.70751	-1.21918
C	1.48028	3.58777	0.69932
N	2.13240	2.27978	0.78934
C	3.03784	1.99723	-0.33642
C	3.45212	0.54296	-0.38459
C	4.80465	0.19710	-0.37634
C	5.18815	-1.13933	-0.44749
C	4.23941	-2.15317	-0.51729
C	2.89049	-1.80363	-0.51862
C	2.45704	-0.46200	-0.45564
O	1.15948	-0.16157	-0.51779
C	-1.90947	2.73160	0.54192
C	2.86026	2.18477	2.05843
H	-0.91727	0.08083	3.01652
H	0.01914	1.57568	2.91521
H	0.83971	0.01820	2.97702
H	-5.73896	-2.14489	0.66297
H	-4.77269	1.36387	-1.63485
H	-2.52443	1.99544	-1.96231
H	-1.40804	0.62088	-2.03910
H	-0.76719	3.17917	-2.00957
H	0.49677	1.99085	-1.70340

H	2.21561	4.41298	0.77060
H	0.84901	3.67390	1.59074
H	3.93975	2.63421	-0.29117
H	2.52406	2.25175	-1.26731
H	5.55869	0.97587	-0.31614
H	4.53440	-3.19480	-0.56729
H	-2.45634	3.49682	-0.02373
H	-1.25784	3.21455	1.26837
H	-2.62852	2.11066	1.07839
H	3.67974	2.92471	2.12035
H	3.29119	1.18740	2.17204
H	2.17761	2.35950	2.89325
C	0.63319	3.84110	-0.56031
H	1.27974	4.20928	-1.36693
H	-0.03670	4.67768	-0.32878
Cl	-7.10985	-0.11588	-0.85070
Cl	-3.15826	-3.00420	1.52296
Cl	1.69212	-3.08246	-0.60422
Cl	6.90322	-1.55785	-0.44022

$A_6C_2B_1$

Al	0.01059	0.66158	0.70354
C	0.31850	0.86112	2.65544
O	-1.24030	-0.47240	0.00951
C	-2.54384	-0.46553	-0.07358
C	-3.24016	-1.64499	-0.45553
C	-4.62233	-1.69178	-0.55082
C	-5.37334	-0.54269	-0.27243
C	-4.74939	0.63483	0.08964
C	-3.34479	0.68637	0.18879
C	-2.73672	1.95463	0.52290
N	-1.46836	2.15395	0.63930
C	-0.97386	3.50221	0.91710

C	-0.22411	4.06652	-0.31255
C	0.28116	2.97804	-1.26894
N	1.00165	1.88315	-0.59354
C	2.26659	1.75272	-0.87421
C	3.13455	0.68778	-0.46062
C	4.51205	0.79507	-0.75976
C	5.36192	-0.24532	-0.45674
C	4.87023	-1.42812	0.12344
C	3.52179	-1.54882	0.39975
C	2.59318	-0.49497	0.13797
O	1.33668	-0.62806	0.39974
H	-0.44780	1.41698	3.21247
H	1.28615	1.33330	2.88614
H	0.36976	-0.14292	3.10134
H	-5.11526	-2.61377	-0.83781
H	-5.33281	1.52682	0.29668
H	-3.42412	2.79488	0.67126
H	-0.28716	3.42194	1.76542
H	-1.79222	4.16964	1.21323
H	-0.57022	2.52507	-1.78771
H	0.92250	3.43153	-2.03323
H	2.72468	2.50865	-1.51906
H	4.89002	1.69996	-1.22604
H	5.54564	-2.24493	0.35211
Cl	-7.12978	-0.61247	-0.39549
Cl	-2.31332	-3.08765	-0.80998
Cl	2.92013	-3.03082	1.11195
Cl	7.08510	-0.11779	-0.80485
H	0.61342	4.68449	0.03012
H	-0.88896	4.71792	-0.89208

$A_6C_3B_1$

Al	0.02351	0.29699	0.73733
C	0.32545	0.39998	2.69796
O	-1.22508	-0.80558	-0.01194
C	-2.53026	-0.80046	-0.07169
C	-3.22892	-1.96316	-0.49768
C	-4.61242	-2.01240	-0.56988
C	-5.36297	-0.88222	-0.22201
C	-4.73727	0.27955	0.18531
C	-3.33128	0.33371	0.26021
C	-2.72192	1.58834	0.64098
N	-1.45147	1.78611	0.73880
C	-0.94744	3.11734	1.06265
C	-0.26154	3.78038	-0.16955
C	0.27698	2.69431	-1.13512
N	1.00953	1.58955	-0.49175
C	2.27542	1.47811	-0.77773
C	3.14900	0.40042	-0.41040
C	4.52674	0.53043	-0.69935
C	5.38239	-0.51805	-0.44427
C	4.89659	-1.73059	0.07629
C	3.54789	-1.87310	0.34103
C	2.61313	-0.81415	0.12624
O	1.35591	-0.96925	0.37328
C	-1.28735	4.61573	-0.95719
C	0.87869	4.68339	0.32803
H	-0.43477	0.94008	3.27835
H	1.29887	0.84679	2.95341
H	0.36109	-0.62452	3.09657
H	-5.10622	-2.92209	-0.89235
H	-5.32050	1.15765	0.44555
H	-3.40780	2.41736	0.84617
H	-0.21463	2.99351	1.86678
H	-1.74931	3.76508	1.44024

H	-0.56559	2.24210	-1.66910
H	0.91594	3.17409	-1.88641
H	2.72913	2.26218	-1.39078
H	4.90035	1.45943	-1.11941
H	5.57654	-2.55314	0.26809
H	-2.16299	4.01735	-1.23762
H	-0.84905	5.01367	-1.88054
H	-1.63535	5.46727	-0.36128
H	1.67227	4.10101	0.81081
H	0.50845	5.40864	1.06282
H	1.32410	5.24920	-0.49906
Cl	-7.12125	-0.95491	-0.31587
Cl	-2.30319	-3.38273	-0.93802
Cl	2.95361	-3.39131	0.97965
Cl	7.10585	-0.36241	-0.77991

$A_6C_5B_1$

O	-1.10223	-1.00115	0.39344
O	1.43631	-0.82104	1.00549
N	-1.47635	1.65962	0.42656
N	1.06808	1.68592	0.00511
C	-0.92170	3.00974	0.50206
C	-1.78848	4.19803	0.08224
C	-0.99709	5.50360	0.28025
C	0.35323	5.47638	-0.45245
C	1.19390	4.24059	-0.08450
C	0.38009	2.96048	-0.32605
C	-2.67316	1.40461	0.02449
C	-3.19694	0.06691	-0.13759
C	-4.54310	-0.07394	-0.53029
C	-5.07425	-1.33116	-0.73795
C	-4.28358	-2.47627	-0.57346
C	-2.95700	-2.34247	-0.19687
C	-2.35709	-1.07462	0.04520
C	2.22496	1.46294	-0.54401
C	3.02906	0.28373	-0.39357
C	4.27981	0.24652	-1.05142
C	5.08664	-0.86310	-0.93099
C	4.67905	-1.96576	-0.16037
C	3.45551	-1.93987	0.48211
C	2.57363	-0.82005	0.39433
H	-0.63113	3.15004	1.55384
H	-2.08242	4.09492	-0.97186
H	-2.71037	4.22613	0.67558
H	-1.59580	6.35404	-0.06614
H	-0.82570	5.65786	1.35454
H	0.92160	6.38606	-0.22601
H	0.17775	5.47931	-1.53772
H	2.11589	4.23580	-0.67634
H	1.49083	4.27884	0.97230
H	0.09416	2.91334	-1.38746
H	-3.35343	2.22234	-0.22679
H	-5.15526	0.81234	-0.66734
H	-4.70337	-3.46211	-0.73881
H	2.64975	2.23177	-1.19492
H	4.59713	1.09843	-1.64504
H	5.32025	-2.83546	-0.06999
Al	-0.02908	0.32349	1.08103
C	-0.14638	0.68719	3.02105
H	-0.02129	-0.25180	3.57938
H	0.65001	1.35697	3.37921
H	-1.10050	1.12444	3.34809
Cl	6.64987	-0.91864	-1.74333
Cl	2.95924	-3.31883	1.43933
Cl	-1.97795	-3.78174	-0.00294

Cl	-6.75925	-1.51259	-1.22214

$A_6C_5B_2$

Al	0.08509	0.19231	1.00536
C	0.47420	0.54386	2.90669
O	-1.41624	-0.82798	1.13779
C	-2.49973	-0.99176	0.41165
C	-3.47946	-1.95317	0.75840
C	-4.65608	-2.10143	0.03192
C	-4.88838	-1.27209	-1.06443
C	-3.95708	-0.30808	-1.43440
C	-2.76928	-0.17350	-0.71144
C	-1.72015	0.80051	-1.18109
N	-1.03965	1.62379	-0.09659
C	-0.22705	2.69268	-0.80846
C	1.08333	3.07807	-0.07418
N	1.82436	1.84165	0.30875
C	2.51537	1.29871	-0.90158
C	3.13589	-0.05556	-0.69132
C	4.49149	-0.28163	-0.93521
C	5.01227	-1.56529	-0.79763
C	4.19849	-2.62863	-0.41407
C	2.84756	-2.39464	-0.16520
C	2.28336	-1.10772	-0.29477
O	0.98675	-0.89507	-0.09032
C	-2.06288	2.17005	0.84274
C	2.84471	2.07491	1.35121
H	-0.18583	-0.09570	3.50386
H	0.29806	1.57829	3.23352
H	1.50406	0.29589	3.19140
H	-5.38349	-2.84952	0.32462
H	-4.15185	0.33077	-2.29085
H	-2.17177	1.48831	-1.90621
H	-0.90917	0.26499	-1.69047
H	0.07490	2.19320	-1.73179
H	1.68321	3.61357	-0.83373
H	3.28118	2.01695	-1.23705
H	1.77454	1.21874	-1.70244
H	5.13687	0.53680	-1.23947
H	4.60497	-3.62718	-0.30478
H	-2.82754	2.74470	0.31113
H	-1.57753	2.80272	1.58319
H	-2.54411	1.34366	1.36139
H	3.58026	2.83423	1.03941
H	3.37547	1.13946	1.53247
H	2.37802	2.38385	2.28395
C	-0.99789	3.96227	-1.25025
H	-0.43393	4.37287	-2.09880
H	-1.97801	3.68839	-1.65776
C	-1.14049	5.07215	-0.19338
H	-1.51572	5.97795	-0.68492
H	-1.89036	4.80387	0.55736
C	0.86663	4.10196	1.05543
H	0.27696	3.67514	1.87456
H	1.83161	4.38468	1.48460
C	0.19413	5.36934	0.50033
H	0.87994	5.84401	-0.21643
H	0.04648	6.09215	1.31105
Cl	6.72165	-1.85098	-1.10873
Cl	1.82720	-3.72909	0.31856
Cl	-3.20193	-2.97847	2.14934
Cl	-6.38306	-1.44219	-1.98141

$A_6C_6B_2$

Al	0.01214	0.25746	1.24889
C	0.28714	0.58034	3.17557

O	-1.47883	-0.79893	1.14263
C	-2.51151	-0.87621	0.33563
C	-3.43109	-1.95076	0.42768
C	-4.55411	-2.04387	-0.38649
C	-4.79489	-1.03642	-1.31843
C	-3.93160	0.04718	-1.43497
C	-2.79611	0.12702	-0.62495
C	-1.83941	1.27762	-0.82691
N	-1.22400	1.82540	0.44605
N	1.58323	1.75787	0.45706
C	2.15152	1.24229	-0.84362
C	2.89286	-0.05608	-0.69526
C	4.18786	-0.20599	-1.19326
C	4.83001	-1.43571	-1.08287
C	4.20357	-2.51867	-0.47044
C	2.91546	-2.35636	0.03269
C	2.22335	-1.12808	-0.06463
O	0.99041	-0.99428	0.38929
C	-2.27910	2.12095	1.46957
C	2.66555	1.90485	1.46827
H	-0.51506	0.08652	3.73970
H	0.28554	1.63606	3.48023
H	1.22926	0.15067	3.54177
H	-5.23037	-2.88528	-0.28796
H	-4.13669	0.82879	-2.16065
H	-2.35816	2.09373	-1.34248
H	-0.99859	0.97898	-1.46566
H	2.81456	2.00376	-1.27315
H	1.30164	1.12688	-1.52613
H	4.68793	0.63032	-1.67220
H	4.70660	-3.47452	-0.37993
H	-3.09418	2.71157	1.03985
H	-1.82046	2.67493	2.28922
H	-2.68227	1.18663	1.85426
H	3.52360	2.45836	1.06761
H	3.01054	0.91659	1.77347
H	2.27263	2.42661	2.34053
C	-0.47886	3.04786	0.14008
C	0.91862	3.02375	0.19010
C	1.63680	4.20062	-0.06128
C	0.97364	5.38626	-0.36284
C	-0.42063	5.40496	-0.42807
C	-1.14086	4.24082	-0.17786
H	2.72108	4.18977	-0.02235
H	1.54434	6.29076	-0.55151
H	-0.94793	6.32338	-0.66828
H	-2.22474	4.26488	-0.22023
Cl	6.46532	-1.62786	-1.71970
Cl	2.12932	-3.71805	0.80874
Cl	-3.14231	-3.20790	1.61818
Cl	-6.22313	-1.13337	-2.35281

$A_6C_8B_1$

Al	0.14259	-0.25541	-0.70706
C	0.59240	-0.11919	-2.63612
O	-1.10471	-1.39422	-0.02971
C	-2.40847	-1.44087	-0.01496
C	-3.07589	-2.61891	0.41877
C	-4.45772	-2.71818	0.44461
C	-5.23831	-1.62779	0.03707
C	-4.64353	-0.45654	-0.38582
C	-3.23717	-0.35014	-0.41264
C	-2.66954	0.90875	-0.82601
N	-1.40404	1.18777	-0.86097
C	-1.02991	2.49483	-1.30997

C	-1.22586	2.87286	-2.64168
C	-0.84027	4.14148	-3.07419
C	-0.26218	5.03698	-2.17424
C	-0.08143	4.66320	-0.84303
C	-0.45420	3.38988	-0.38816
C	-0.36468	3.04051	1.05432
C	-0.96224	3.89175	1.99805
C	-0.92846	3.61302	3.36335
C	-0.28878	2.46049	3.81674
C	0.32444	1.60798	2.90034
C	0.29818	1.89107	1.52851
N	1.00902	0.99531	0.65083
C	2.30701	0.96991	0.84389
C	3.22918	-0.00617	0.35017
C	4.61438	0.23199	0.51520
C	5.51941	-0.74945	0.18240
C	5.08269	-2.00408	-0.28421
C	3.73167	-2.25449	-0.42217
C	2.74269	-1.26215	-0.13934
O	1.48331	-1.50371	-0.27504
H	-0.26589	0.08134	-3.29100
H	1.03767	-1.06466	-2.97416
H	1.33364	0.66502	-2.85148
H	-4.92858	-3.63640	0.77716
H	-5.24883	0.39044	-0.69377
H	-3.38399	1.68465	-1.11482
H	-1.66736	2.16311	-3.33410
H	-0.98927	4.42393	-4.11246
H	0.04611	6.02468	-2.50504
H	0.35805	5.36327	-0.13793
H	-1.47549	4.78054	1.64207
H	-1.40919	4.28846	4.06519
H	-0.26309	2.22194	4.87608
H	0.82504	0.70536	3.23827
H	2.73324	1.74511	1.48416
H	4.95145	1.18859	0.90241
H	5.80502	-2.77387	-0.53221
Cl	3.19774	-3.82288	-0.98749
Cl	7.24901	-0.46050	0.35510
Cl	-2.11253	-3.98881	0.92675
Cl	-6.99400	-1.76720	0.07370

$A_6C_9B_1$

Al	-0.27497	-0.32162	-1.13206
C	-0.44536	-1.36794	-2.80431
O	-2.04169	0.32201	-1.28114
C	-3.11059	0.25011	-0.56986
C	-4.37578	0.68244	-1.07774
C	-5.53090	0.60865	-0.32549
C	-5.48164	0.10709	0.98953
C	-4.29113	-0.30935	1.53893
C	-3.10380	-0.25040	0.76987
C	-1.87828	-0.63700	1.39536
N	-0.68828	-0.73075	0.85295
C	0.36735	-0.98858	1.81584
C	0.89371	0.10616	2.50780
C	1.91906	-0.07070	3.43709
C	2.41929	-1.35022	3.67253
C	1.87538	-2.44037	2.99273
C	0.83770	-2.29195	2.06205
C	0.22217	-3.51620	1.41944
C	0.20616	-3.56618	-0.12342
C	1.55318	-3.53944	-0.81113
C	2.12499	-4.76960	-1.16687
C	3.33321	-4.86658	-1.85316

C	3.99370	-3.69916	-2.22975
C	3.45414	-2.46444	-1.88328
C	2.26088	-2.36044	-1.14215
N	1.79476	-1.04503	-0.79191
C	2.73106	-0.21645	-0.42364
C	2.62681	1.21235	-0.25305
C	3.76136	1.88652	0.24859
C	3.75605	3.26196	0.36196
C	2.63842	4.00846	-0.03556
C	1.52534	3.35324	-0.53722
C	1.46862	1.93767	-0.65426
O	0.39882	1.35946	-1.12391
H	-1.26969	-2.09445	-2.77466
H	0.44634	-1.89831	-3.15497
H	-0.72370	-0.64661	-3.58743
H	-6.47384	0.93930	-0.74685
H	-4.25175	-0.68385	2.55739
H	-1.95226	-0.86215	2.46178
H	0.49830	1.09698	2.30463
H	2.32114	0.78809	3.96700
H	3.22153	-1.50380	4.38901
H	2.25399	-3.43960	3.19316
H	-0.82023	-3.61402	1.75501
H	0.74553	-4.40274	1.79486
H	-0.29532	-4.49874	-0.40642
H	-0.43157	-2.77182	-0.51361
H	1.58729	-5.67811	-0.90529
H	3.73455	-5.84092	-2.11715
H	4.91280	-3.74174	-2.80739
H	3.94226	-1.55762	-2.22621
H	3.73045	-0.61646	-0.23929
H	4.63550	1.31321	0.54134
H	2.64117	5.08956	0.04556
Cl	0.13461	4.29204	-1.03677
Cl	5.16659	4.10382	0.99949
Cl	-4.44664	1.31203	-2.70914
Cl	-6.97144	0.02891	1.92727

$A_6C_{11}B_1$

Al	-0.08268	-0.59091	1.02571
N	1.45956	0.64896	0.40129
N	-1.06281	0.85539	-0.01882
O	0.89227	-1.98474	0.32849
O	-1.62487	-1.62965	0.92899
C	2.67929	-3.45910	-0.15245
C	4.01878	-3.68848	-0.42245
C	4.90584	-2.60592	-0.49272
C	4.45585	-1.31501	-0.30243
C	3.09534	-1.07740	-0.01971
C	2.16072	-2.15332	0.07374
C	2.66457	0.29555	0.10827
C	1.02958	2.05659	0.47622
C	-0.32224	2.12082	-0.29703
C	-2.23818	0.73833	-0.56572
C	-3.11826	-0.38820	-0.46322
C	-4.36128	-0.32113	-1.13401
C	-5.24429	-1.37419	-1.04849
C	-4.92556	-2.51848	-0.29635
C	-3.71225	-2.59285	0.36125
C	-2.75111	-1.53830	0.30441
C	0.05436	-0.27295	2.97378
H	4.37449	-4.70083	-0.57734
H	5.14088	-0.47539	-0.37006
H	3.42498	1.05669	-0.07892
H	0.80499	2.23621	1.53496

H	-0.08941	2.13106	-1.36797
H	-2.61567	1.57374	-1.16113
H	-4.61124	0.56484	-1.70995
H	-5.62838	-3.34160	-0.23062
H	-0.05560	-1.23049	3.50395
H	1.00667	0.16289	3.30758
H	-0.74669	0.37349	3.36299
C	-1.11579	3.37340	0.04007
C	-1.28333	4.38013	-0.91777
C	-1.68043	3.54768	1.31305
C	-1.99461	5.54298	-0.61218
H	-0.84992	4.25702	-1.90738
C	-2.39240	4.70663	1.61795
H	-1.57263	2.76952	2.06459
C	-2.55032	5.70859	0.65624
H	-2.11474	6.31528	-1.36713
H	-2.82627	4.82700	2.60695
H	-3.10635	6.61099	0.89561
C	2.02251	3.10963	0.01829
C	2.54882	4.01397	0.94919
C	2.43416	3.20710	-1.32079
C	3.45594	4.99938	0.55580
H	2.23873	3.95026	1.98935
C	3.34395	4.18829	-1.71533
H	2.05179	2.50863	-2.06128
C	3.85530	5.08902	-0.77802
H	3.84838	5.69562	1.29192
H	3.65300	4.24904	-2.75535
H	4.56160	5.85475	-1.08686
Cl	-6.79652	-1.30393	-1.87991
Cl	-3.33050	-4.02010	1.29939
Cl	1.58235	-4.82209	-0.07595
Cl	6.60702	-2.91028	-0.83841

$A_7C_1B_1$

Al	-0.00999	0.38641	0.81025
N	1.35247	1.82045	0.19215
N	-1.18153	1.67211	-0.30154
O	1.25602	-0.86571	0.36024
O	-1.32750	-0.89819	0.56469
Cl	7.06161	-0.89330	-0.70858
Cl	-7.07836	-0.77751	-0.73201
C	3.27013	-2.04222	0.05800
C	4.63131	-2.05964	-0.18946
C	5.32513	-0.85425	-0.39042
C	4.65800	0.35231	-0.34643
C	3.27050	0.38650	-0.08444
C	2.54505	-0.82705	0.12778
C	2.60169	1.66035	-0.09245
C	0.72122	3.12768	0.07364
C	-0.53633	2.90779	-0.77184
C	-2.44234	1.50713	-0.56763
C	-3.21056	0.33545	-0.26907
C	-4.59160	0.34074	-0.57792
C	-5.35315	-0.78523	-0.35586
C	-4.76528	-1.95398	0.16491
C	-3.41677	-1.97930	0.46376
C	-2.59369	-0.83849	0.27016
C	-0.12138	0.84096	2.73581
H	5.16698	-3.00297	-0.22987
H	5.19146	1.28440	-0.51029
H	3.20097	2.53382	-0.37288
H	1.38443	3.87363	-0.38031
H	0.43984	3.47165	1.07683
H	-1.21629	3.76527	-0.70893

H	-0.24550	2.77518	-1.82213
H	-2.97605	2.31520	-1.07916
H	-5.04116	1.23930	-0.99082
H	-5.37763	-2.83513	0.32998
H	-0.15804	-0.07798	3.33966
H	0.73020	1.42671	3.11144
H	-1.03067	1.40568	2.99232
H	2.72418	-2.96759	0.21208
H	-2.95443	-2.87603	0.86397

$A_7C_2B_1$

Al	0.04346	0.19985	0.71262
C	0.31222	0.49238	2.66251
O	-1.20637	-0.95599	0.06269
C	-2.51452	-0.93100	-0.06046
C	-3.19550	-2.12640	-0.39000
C	-4.57303	-2.16054	-0.52793
C	-5.32413	-0.98883	-0.34524
C	-4.69894	0.20181	-0.03222
C	-3.29594	0.25069	0.11233
C	-2.68348	1.52486	0.40053
N	-1.41495	1.71123	0.54699
C	-0.90978	3.06394	0.77679
C	-0.12618	3.56690	-0.45809
C	0.39305	2.43076	-1.34950
N	1.08118	1.36077	-0.60580
C	2.35526	1.21012	-0.83829
C	3.19296	0.15442	-0.35531
C	4.58333	0.22507	-0.61356
C	5.40553	-0.81674	-0.24715
C	4.87127	-1.97118	0.35963
C	3.51506	-2.06566	0.60023
C	2.62896	-1.00453	0.26908
O	1.35688	-1.11685	0.50063
H	-0.44922	1.09718	3.17363
H	1.28829	0.94763	2.89227
H	0.32336	-0.48808	3.16177
H	-5.07464	-3.09087	-0.77538
H	-5.28019	1.10963	0.10209
H	-3.36071	2.38249	0.48707
H	-0.24260	3.01320	1.64290
H	-1.72605	3.75331	1.02579
H	-0.44941	1.96531	-1.87186
H	1.06056	2.84300	-2.11518
H	2.84095	1.93687	-1.49732
H	4.99138	1.10631	-1.10053
H	5.53274	-2.78704	0.63436
H	-2.60408	-3.02628	-0.52673
H	3.09438	-2.95331	1.06215
H	-0.77134	4.19590	-1.08313
H	0.70806	4.19388	-0.12350
Cl	-7.08004	-1.04335	-0.52261
Cl	7.14292	-0.72458	-0.54959

$A_8C_1B_1$

Al	-0.28034	2.31698	-0.66764
N	-1.84569	3.67479	-0.62625
N	0.42579	3.78048	0.58444
O	-1.53886	1.03584	-0.28592
O	1.08414	1.22369	-0.03705
C	-3.47418	-0.33509	-0.36120
C	-4.82730	-0.41891	-0.66393
C	-5.64077	0.69335	-0.98294
C	-5.04072	1.93511	-0.99119
C	-3.65500	2.08399	-0.75040
C	-2.84054	0.94633	-0.46654

C	-3.10655	3.40916	-0.72546	C	2.89916	-0.90936	-0.10640
C	-1.36247	5.03432	-0.46660	H	1.88206	-1.18254	0.20857
C	-0.40185	4.98655	0.72814	C	3.81036	-2.13193	0.19555
C	1.55435	3.73819	1.23718	C	2.82483	-0.74132	-1.65879
C	2.48006	2.65572	1.26842	H	3.91855	-2.25155	1.28238
C	3.67324	2.81573	2.01951	C	5.20992	-2.02113	-0.45947
C	4.59719	1.80080	2.11874	C	3.11966	-3.38907	-0.38571
C	4.32502	0.59752	1.41745	H	2.22680	0.14226	-1.89338
C	3.19724	0.38352	0.64102	C	4.23235	-0.60008	-2.27311
C	2.20756	1.42545	0.59178	C	2.13614	-1.99307	-2.24374
C	0.24816	2.57410	-2.56301	H	5.78695	-2.92692	-0.22395
H	-5.30197	-1.39427	-0.66451	H	5.78176	-1.17561	-0.06272
H	-5.63084	2.82626	-1.20095	C	5.06732	-1.86457	-1.98535
H	-3.82307	4.23941	-0.77066	H	2.13563	-3.52864	0.08205
H	-2.17200	5.75802	-0.30262	H	3.71449	-4.28255	-0.14864
H	-0.81223	5.32442	-1.37125	C	2.96612	-3.25080	-1.91671
H	0.21383	5.89327	0.78284	H	4.73855	0.28557	-1.86560
H	-0.98777	4.91571	1.65490	H	4.14404	-0.44319	-3.35727
H	1.83488	4.61793	1.82880	H	1.12065	-2.08510	-1.83482
H	3.84409	3.76319	2.52900	H	2.03137	-1.88747	-3.33281
H	5.05363	-0.20119	1.51009	H	6.06320	-1.76341	-2.43800
H	0.29084	1.60169	-3.07507	C	4.35993	-3.10770	-2.56211
H	-0.43261	3.20576	-3.15042	H	2.46359	-4.14260	-2.31541
H	1.25375	3.00859	-2.66099	H	4.26160	-3.01700	-3.65307
C	5.86316	1.93794	2.93097	H	4.96314	-4.00635	-2.36929
H	5.92123	2.91926	3.41326	C	-2.60367	-1.53271	-0.00191
H	5.92146	1.17690	3.71999	H	-1.73625	-1.46203	-0.67348
H	6.75969	1.82117	2.30831	C	-3.21821	-2.94655	-0.20374
C	-7.10826	0.50565	-1.28809	C	-2.01902	-1.46992	1.44645
H	-7.63665	0.01853	-0.45855	H	-3.67808	-3.00977	-1.19953
H	-7.60061	1.46537	-1.47660	C	-4.26364	-3.31611	0.87863
H	-7.25892	-0.12273	-2.17559	C	-2.06675	-3.97656	-0.11140

H	-1.62061	-0.46784	1.62408
C	-3.09582	-1.80616	2.49812
C	-0.87208	-2.49737	1.54618
H	-4.63085	-4.33452	0.68657
H	-5.13849	-2.65860	0.84545
C	-3.62464	-3.23866	2.27796
H	-1.31667	-3.77351	-0.88798
H	-2.45586	-4.98704	-0.30050
C	-1.41243	-3.91771	1.28692
H	-3.92415	-1.08702	2.43948
H	-2.66570	-1.71292	3.50538
H	-0.08785	-2.24615	0.82020
H	-0.41089	-2.44883	2.54288
H	-4.37987	-3.47677	3.03945
C	-2.46479	-4.25132	2.36420
H	-0.58947	-4.64395	1.33587
H	-2.00453	-4.21897	3.36184
H	-2.84679	-5.27223	2.22148

$A_8C_2B_1$

Al	0.26265	2.16028	-0.56063
C	-0.53743	2.49449	-2.35860
O	1.52535	0.87363	-0.28248
C	2.79428	0.72660	-0.61857
C	3.40836	-0.55818	-0.47086
C	4.71204	-0.71307	-0.93018
C	5.49072	0.33607	-1.46886
C	4.90936	1.58736	-1.53925
C	3.57440	1.80345	-1.12803
C	3.05141	3.14281	-1.20623
N	1.84639	3.49214	-0.89724
C	1.46567	4.90094	-0.95519
C	1.19028	5.44916	0.46409
C	0.83732	4.34973	1.47549
N	-0.16967	3.39304	0.98661
C	-1.32342	3.38352	1.59969
C	-2.39416	2.45660	1.41648
C	-3.60256	2.68428	2.12421
C	-4.63102	1.76872	2.09628
C	-4.43308	0.58420	1.34015
C	-3.28601	0.30337	0.61383
C	-2.21241	1.26105	0.65377
O	-1.08293	1.00693	0.05423
H	0.00794	3.18207	-3.01957
H	-1.57391	2.86036	-2.30024
H	-0.59552	1.53545	-2.89651
H	5.16844	-1.69627	-0.88763
H	5.47651	2.43286	-1.92568
H	3.75294	3.91479	-1.54526
H	0.55265	4.96355	-1.55635
H	2.23554	5.49953	-1.45886
H	1.73921	3.77459	1.71103
H	0.49390	4.80922	2.41015
H	-1.48983	4.14644	2.36745
H	-3.70075	3.60192	2.70246
H	-5.23581	-0.14576	1.35356
H	2.07752	5.96787	0.84735
H	0.38216	6.18808	0.41341
C	2.56304	-1.68910	0.10405
H	1.61634	-1.63958	-0.45165
C	2.17431	-1.48932	1.60520
C	3.10156	-3.13922	-0.05624
H	1.83797	-0.45931	1.74965
C	3.36492	-1.79124	2.53796
C	1.01432	-2.45396	1.93179

H	3.42850	-3.29818	-1.09272
C	4.26421	-3.46843	0.91319
C	1.93735	-4.10765	0.26381
H	3.07055	-1.60203	3.58012
H	4.20348	-1.11611	2.31869
C	3.81285	-3.25784	2.37077
H	0.15123	-2.22384	1.29303
H	0.68824	-2.30962	2.97176
C	1.46903	-3.91246	1.72321
H	5.14803	-2.85260	0.71662
H	4.57133	-4.51297	0.75971
H	1.10150	-3.93291	-0.42738
H	2.26504	-5.14590	0.11190
H	4.65080	-3.46971	3.04879
C	2.63946	-4.20855	2.68351
H	0.63518	-4.59581	1.93396
H	2.96496	-5.25328	2.57747
H	2.31271	-4.08008	3.72523
C	-3.05197	-0.98918	-0.15874
H	-2.06708	-1.33776	0.18421
C	-2.90766	-0.79928	-1.70318
C	-4.04864	-2.15808	0.08401
H	-2.25038	0.05150	-1.89517
C	-4.27788	-0.56375	-2.37011
C	-2.27111	-2.08014	-2.28478
H	-4.20926	-2.28738	1.16291
C	-5.41077	-1.95268	-0.62451
C	-3.41016	-3.44464	-0.49310
H	-4.13594	-0.39382	-3.44672
H	-4.74749	0.34370	-1.96660
C	-5.19765	-1.78025	-2.14030
H	-1.28034	-2.23988	-1.83769

H	-2.11638	-1.96337	-3.36688
C	-3.18656	-3.29125	-2.01418
H	-5.94707	-1.08130	-0.23432
H	-6.04972	-2.82587	-0.42881
H	-2.45544	-3.64989	0.01022
H	-4.06548	-4.30510	-0.29654
H	-6.16628	-1.61188	-2.63033
C	-4.54193	-3.05395	-2.71163
H	-2.72184	-4.20480	-2.40931
H	-5.20485	-3.91783	-2.55993
H	-4.39388	-2.95169	-3.79605
C	-5.92632	1.98608	2.84116
H	-5.90371	2.91819	3.41589
H	-6.78418	2.04037	2.15746
H	-6.13164	1.16778	3.54395
C	6.90321	0.07762	-1.93702
H	7.53598	-0.30877	-1.12713
H	6.93173	-0.66538	-2.74508
H	7.37148	0.99299	-2.31440

$A_8C_3B_1$

Al	-0.44819	1.80086	0.65954
C	0.29627	2.16370	2.47614
O	-1.52945	0.36781	0.32952
C	-2.76283	0.04156	0.67223
C	-3.19959	-1.30976	0.49100
C	-4.46448	-1.65292	0.95670
C	-5.37047	-0.73488	1.53429
C	-4.96234	0.58092	1.63871
C	-3.67433	0.98786	1.22218
C	-3.33347	2.38211	1.34112
N	-2.18834	2.89637	1.03347
C	-1.98557	4.33622	1.13718

C	-1.84857	4.99857	-0.26651	C	-2.21493	-2.29920	-0.12230
C	-1.32428	3.96385	-1.29418	H	-1.27861	-2.13876	0.43017
N	-0.19165	3.14255	-0.83736	C	-1.86892	-2.00634	-1.61862
C	0.94139	3.29305	-1.47170	C	-2.55248	-3.81250	-0.00247
C	2.12414	2.50453	-1.33536	H	-1.67487	-0.93691	-1.73445
C	3.27696	2.90080	-2.06136	C	-3.01598	-2.43853	-2.55467
C	4.41317	2.12274	-2.08009	C	-0.59299	-2.79705	-1.97859
C	4.38581	0.90605	-1.35024	H	-2.84624	-4.04362	1.03037
C	3.30183	0.46503	-0.60683	C	-3.66907	-4.26633	-0.97571
C	2.11349	1.27689	-0.60292	C	-1.27181	-4.60649	-0.35605
O	1.03885	0.86431	0.00873	H	-2.75809	-2.18208	-3.59213
C	-3.22579	5.47559	-0.76268	H	-3.93550	-1.88833	-2.31221
C	-0.88918	6.19495	-0.15853	C	-3.26218	-3.95608	-2.42857
H	-0.34408	2.73361	3.16341	H	0.23699	-2.47203	-1.33723
H	1.26878	2.67822	2.44344	H	-0.29797	-2.58045	-3.01529
H	0.49199	1.19888	2.96934	C	-0.84639	-4.30875	-1.81127
H	-4.78455	-2.68729	0.88879	H	-4.62504	-3.77906	-0.75732
H	-5.63349	1.32931	2.05769	H	-3.83345	-5.34635	-0.85131
H	-4.12441	3.04360	1.71459	H	-0.46105	-4.34056	0.33604
H	-1.05926	4.48982	1.70157	H	-1.45598	-5.68322	-0.23298
H	-2.79881	4.81368	1.70067	H	-4.06996	-4.25899	-3.10865
H	-2.13717	3.27353	-1.54471	C	-1.97482	-4.73164	-2.77435
H	-1.05264	4.49032	-2.21798	H	0.06964	-4.86773	-2.04604
H	0.99424	4.08892	-2.22183	H	-2.15662	-5.81316	-2.69738
H	3.24499	3.83660	-2.61735	H	-1.67716	-4.53114	-3.81341
H	5.27425	0.28506	-1.39918	C	3.25551	-0.86133	0.14231
H	-3.95952	4.66013	-0.75631	H	2.31878	-1.33186	-0.18984
H	-3.16667	5.85957	-1.78870	C	3.11945	-0.71905	1.69267
H	-3.61114	6.28247	-0.12833	C	4.39405	-1.88191	-0.14152
H	0.12638	5.87286	0.10003	H	2.35798	0.03186	1.91389
H	-1.22198	6.89372	0.61899	C	4.45950	-0.31150	2.33796
H	-0.84143	6.75031	-1.10328	C	2.67385	-2.08351	2.26145

H	4.54731	-1.97107	-1.22557
C	5.73130	-1.50592	0.54448
C	3.94736	-3.25192	0.42316
H	4.31911	-0.18040	3.42020
H	4.79360	0.65780	1.94349
C	5.52971	-1.38844	2.06708
H	1.70461	-2.36868	1.82976
H	2.52739	-2.00625	3.34822
C	3.73846	-3.15512	1.95096
H	6.13710	-0.56386	0.16121
H	6.47756	-2.28166	0.32015
H	3.01800	-3.57564	-0.06523
H	4.70789	-4.01282	0.19711
H	6.47724	-1.09859	2.54134
C	5.06445	-2.74795	2.62661
H	3.40988	-4.12942	2.33750
H	5.83435	-3.51169	2.44571
H	4.92748	-2.68438	3.71543
C	-6.72857	-1.19586	2.00782
H	-7.31263	-1.64474	1.19360
H	-6.64550	-1.95579	2.79617
H	-7.31262	-0.36288	2.41348
C	5.65119	2.51972	-2.84810
H	6.51448	2.65923	-2.18362
H	5.93564	1.75391	-3.58184
H	5.49874	3.45842	-3.39151

$A_8C_5B_1$

O	1.47560	0.38271	0.36952
O	-0.64299	-1.23103	0.31899
N	3.19306	-1.65300	0.72916
N	1.32943	-2.90107	-0.52887
C	3.47193	-3.08008	0.61502
C	4.92407	-3.55346	0.52427
C	4.95963	-5.08957	0.42853
C	4.09716	-5.61666	-0.72901
C	2.65416	-5.08438	-0.67229
C	2.66083	-3.54925	-0.61249
C	4.10381	-0.74225	0.80105
C	3.82847	0.67221	0.79519
C	4.90787	1.56035	1.00465
C	4.73071	2.92985	0.97416
C	3.43716	3.41029	0.66827
C	2.34615	2.59134	0.39957
C	2.52199	1.17442	0.52253
C	0.44752	-3.19776	-1.44390
C	-0.88087	-2.68564	-1.55431
C	-1.69347	-3.15586	-2.61787
C	-2.99212	-2.72282	-2.77175
C	-3.49988	-1.81690	-1.80439
C	-2.77216	-1.34033	-0.72456
C	-1.39463	-1.73786	-0.61753
H	3.01327	-3.54799	1.49997
H	5.40756	-3.11126	-0.35840
H	5.48977	-3.22138	1.40336
H	5.99552	-5.42957	0.31108
H	4.59631	-5.51427	1.37480
H	4.08546	-6.71321	-0.71920
H	4.55111	-5.31780	-1.68481
H	2.09722	-5.44020	-1.54642
H	2.13706	-5.47220	0.21609
H	3.16776	-3.16071	-1.50909
H	5.15839	-1.02811	0.85300
H	5.89255	1.14137	1.20683
H	3.30021	4.48614	0.65181

H	0.72905	-3.91997	-2.21579
H	-1.26624	-3.86939	-3.32096
H	-4.52268	-1.47879	-1.93683
Al	1.14768	-1.37083	0.80683
C	0.91562	-1.83773	2.72524
H	0.20868	-1.13967	3.19807
H	0.48291	-2.84006	2.86763
H	1.83708	-1.80281	3.32425
C	-3.86822	-3.18730	-3.91058
H	-4.76997	-3.69844	-3.54792
H	-4.20719	-2.34591	-4.52961
H	-3.33396	-3.88428	-4.56515
C	5.86135	3.89435	1.24325
H	6.80121	3.36398	1.43056
H	6.02694	4.57386	0.39685
H	5.65672	4.52287	2.12027
C	0.95424	3.10562	0.05163
H	0.27645	2.54964	0.71366
C	0.50064	2.75050	-1.40207
C	0.66376	4.61635	0.28168
H	0.74231	1.70312	-1.60263
C	1.18882	3.65850	-2.44180
C	-1.02770	2.94643	-1.48892
H	1.01600	4.91087	1.27952
C	1.30466	5.53462	-0.78904
C	-0.87013	4.81146	0.20419
H	0.88112	3.35550	-3.45277
H	2.27956	3.53675	-2.39352
C	0.81246	5.13383	-2.19237
H	-1.52699	2.28007	-0.77344
H	-1.38594	2.66516	-2.48951
C	-1.38655	4.41741	-1.19801

H	2.39836	5.49299	-0.76458
H	1.02798	6.57712	-0.57534
H	-1.36624	4.20367	0.97325
H	-1.12274	5.86047	0.41431
H	1.29332	5.77191	-2.94630
C	-0.71792	5.30976	-2.26377
H	-2.47653	4.54867	-1.23586
H	-0.98203	6.36419	-2.09913
H	-1.08618	5.04074	-3.26402
C	-3.30774	-0.36375	0.31539
H	-2.56304	0.44413	0.34829
C	-3.35311	-0.94346	1.76703
C	-4.68244	0.30300	0.02816
H	-2.41187	-1.45912	1.97292
C	-4.53561	-1.91716	1.94498
C	-3.51295	0.23784	2.74851
H	-4.69968	0.68241	-1.00250
C	-5.87793	-0.65646	0.25072
C	-4.84365	1.48964	1.00857
H	-4.51546	-2.33480	2.96159
H	-4.43952	-2.76508	1.25317
C	-5.86989	-1.18408	1.69808
H	-2.65969	0.92260	2.64938
H	-3.50546	-0.13221	3.78364
C	-4.83517	0.97987	2.46676
H	-5.85478	-1.50411	-0.44232
H	-6.81326	-0.11412	0.05074
H	-4.03321	2.21590	0.85818
H	-5.78596	2.01672	0.80197
H	-6.70798	-1.88000	1.83962
C	-6.00920	0.00103	2.67521
H	-4.93716	1.83178	3.15264

H	-6.96704	0.51437	2.50861
H	-6.01735	-0.36101	3.71311

$A_8C_6B_1$

Al	0.97167	1.63167	0.72287
O	-0.76505	1.18719	0.23610
O	1.57142	-0.04381	0.25849
N	0.76025	3.32388	-0.39714
N	2.94124	2.27437	0.43529
C	1.82811	4.23617	-0.24419
C	1.75843	5.61480	-0.47587
C	2.88061	6.41250	-0.25958
C	4.06978	5.84532	0.20910
C	4.14439	4.47727	0.46288
C	3.02978	3.66273	0.23138
C	-0.22046	3.58033	-1.23092
C	-1.40656	2.81465	-1.38838
C	-1.66429	1.66226	-0.57922
C	-2.94711	1.02198	-0.68322
C	-3.81665	1.47724	-1.66216
C	-3.55420	2.58112	-2.51653
C	-2.36115	3.24565	-2.34859
C	3.98841	1.50773	0.54559
C	3.94148	0.08278	0.65151
C	2.73195	-0.64435	0.42374
C	2.79806	-2.07478	0.35162
C	4.00839	-2.68522	0.65533
C	5.20575	-1.98265	0.93366
C	5.15707	-0.60574	0.89101
C	0.85905	1.98933	2.66702
H	0.82420	6.06919	-0.79118
H	2.81912	7.48298	-0.43288
H	4.93530	6.47380	0.39793
H	5.06178	4.05207	0.85766
H	-0.13634	4.46848	-1.86134
H	-4.76276	0.96419	-1.80048
H	-2.12808	4.11847	-2.95642
H	4.98614	1.95347	0.51586
H	4.05373	-3.76848	0.68795
H	6.06005	-0.02080	1.05812
H	0.00954	1.46684	3.12889
H	0.72456	3.05834	2.89244
H	1.75984	1.67086	3.21178
C	6.47703	-2.73587	1.24403
H	6.37598	-3.33911	2.15605
H	6.74829	-3.42576	0.43428
H	7.31895	-2.05152	1.39302
C	-4.57307	2.99798	-3.54957
H	-4.76785	2.19376	-4.27150
H	-5.53687	3.25275	-3.08942
H	-4.23322	3.87311	-4.11341
C	1.51943	-2.83346	0.01664
H	0.75214	-2.40156	0.67403
C	1.51172	-4.36919	0.26344
C	1.01178	-2.58435	-1.44160
H	1.90722	-4.58311	1.26558
C	2.31846	-5.16143	-0.79596
C	0.04181	-4.84777	0.18422
H	1.05412	-1.51256	-1.65356
C	1.86070	-3.35856	-2.47063
C	-0.45254	-3.06355	-1.52905
H	2.24243	-6.23500	-0.57117
H	3.38444	-4.91345	-0.76994
C	1.76595	-4.87525	-2.20492
H	-0.56249	-4.33531	0.94529

H	-0.01096	-5.92303	0.40610
C	-0.53226	-4.57253	-1.22359
H	2.90918	-3.03426	-2.42370
H	1.50414	-3.12909	-3.48477
H	-1.07016	-2.49573	-0.82093
H	-0.85298	-2.86322	-2.53302
H	2.36116	-5.42028	-2.95009
C	0.29592	-5.33533	-2.27776
H	-1.57839	-4.90528	-1.26300
H	-0.11197	-5.15073	-3.28170
H	0.23300	-6.41866	-2.10162
C	-3.23221	-0.15652	0.23994
H	-2.36170	-0.81814	0.12868
C	-4.49323	-1.01208	-0.06887
C	-3.27505	0.22414	1.75614
H	-4.51636	-1.26195	-1.13824
C	-5.81339	-0.30719	0.33124
C	-4.39092	-2.31851	0.75439
H	-2.42066	0.86559	1.98591
C	-4.58741	0.95104	2.11388
C	-3.17167	-1.07734	2.58026
H	-6.65712	-0.97478	0.10480
H	-5.97759	0.60922	-0.24544
C	-5.79584	0.03469	1.83335
H	-3.48396	-2.87109	0.47375
H	-5.24519	-2.97034	0.52284
C	-4.36954	-1.99567	2.26476
H	-4.67886	1.88105	1.53644
H	-4.56830	1.23849	3.17459
H	-2.22597	-1.58834	2.35337
H	-3.15509	-0.83951	3.65330
H	-6.72584	0.55375	2.10219
C	-5.67225	-1.26590	2.65258
H	-4.28353	-2.92920	2.83713
H	-5.67048	-1.03900	3.72812
H	-6.54122	-1.91245	2.46412

$A_8C_9B_1$

Al	0.91615	-0.93653	-0.56594
C	0.57873	-2.05393	-2.17551
O	-0.86447	-1.13060	0.02914
C	-1.45214	-1.63831	1.06954
C	-2.88426	-1.79108	1.09770
C	-3.45754	-2.26566	2.26598
C	-2.72485	-2.64973	3.42186
C	-1.35601	-2.51948	3.37980
C	-0.70021	-2.01723	2.22345
C	0.70522	-1.81260	2.28920
N	1.52147	-1.39752	1.34288
C	2.86744	-1.14060	1.81646
C	3.12470	0.10043	2.40718
C	4.39973	0.41253	2.87939
C	5.42531	-0.52353	2.75867
C	5.15934	-1.76690	2.18445
C	3.88457	-2.10851	1.71195
C	3.63115	-3.50080	1.17516
C	3.06034	-3.60594	-0.25551
C	3.90559	-3.01282	-1.36122
C	4.77083	-3.87535	-2.04983
C	5.55663	-3.45564	-3.12082
C	5.46325	-2.13325	-3.54901
C	4.61680	-1.25371	-2.88069
C	3.85984	-1.65626	-1.76237
N	3.04226	-0.67410	-1.10692
C	3.59688	0.50383	-0.98346

C	2.97777	1.74527	-0.61144
C	3.82512	2.86786	-0.44964
C	3.31627	4.12100	-0.17349
C	1.91150	4.24897	-0.07866
C	1.01742	3.19274	-0.23574
C	1.56784	1.89441	-0.49615
O	0.76431	0.86221	-0.64480
H	0.17326	-3.04759	-1.93726
H	1.41932	-2.20262	-2.86231
H	-0.21181	-1.54394	-2.74753
H	-4.53835	-2.34228	2.32443
H	-0.75145	-2.79122	4.24367
H	1.15535	-2.00969	3.26555
H	2.31751	0.82286	2.48235
H	4.58474	1.38241	3.33269
H	6.42562	-0.29400	3.11584
H	5.95635	-2.50266	2.10707
H	2.92860	-4.02302	1.84035
H	4.57005	-4.06443	1.22217
H	2.93044	-4.67340	-0.46897
H	2.05544	-3.18410	-0.28283
H	4.81128	-4.91611	-1.73581
H	6.20707	-4.16062	-3.63112
H	6.02752	-1.78704	-4.41071
H	4.50625	-0.23969	-3.25199
H	4.66378	0.58769	-1.20547
H	4.89976	2.72512	-0.54882
H	1.52266	5.23975	0.12648
C	4.20650	5.32703	0.01164
H	3.97278	6.11819	-0.71279
H	4.09082	5.76657	1.01128
H	5.26248	5.06515	-0.11368
C	-3.45036	-3.16941	4.63972
H	-4.00484	-4.09149	4.41956
H	-2.75257	-3.39027	5.45445
H	-4.18105	-2.44157	5.01668
C	-3.66913	-1.33775	-0.12703
H	-3.26334	-0.34272	-0.36019
C	-5.20658	-1.16749	0.03388
C	-3.41590	-2.19605	-1.40846
H	-5.42092	-0.60734	0.95414
C	-5.96773	-2.51658	0.05579
C	-5.71823	-0.36080	-1.18378
H	-2.34267	-2.37000	-1.51349
C	-4.16173	-3.54396	-1.33770
C	-3.92425	-1.39487	-2.62661
H	-7.04502	-2.31656	0.14700
H	-5.69074	-3.12838	0.92080
C	-5.68210	-3.30597	-1.23575
H	-5.23004	0.62285	-1.21610
H	-6.79728	-0.17771	-1.08121
C	-5.43839	-1.13593	-2.49058
H	-3.81469	-4.12779	-0.47413
H	-3.93009	-4.13702	-2.23366
H	-3.37753	-0.44495	-2.70235
H	-3.72221	-1.95271	-3.55196
H	-6.20772	-4.26990	-1.20032
C	-6.17223	-2.49233	-2.45075
H	-5.79362	-0.54780	-3.34756
H	-5.98587	-3.04806	-3.38063
H	-7.25842	-2.33599	-2.38427
C	-0.49966	3.31653	-0.15817
H	-0.85310	2.28792	-0.26551
C	-1.17218	4.11537	-1.31918

C	-1.10086	3.82722	1.18899
H	-0.72215	3.81088	-2.27257
C	-1.03354	5.64229	-1.14145
C	-2.67608	3.75728	-1.32101
H	-0.60231	3.31674	2.02329
C	-0.97738	5.35633	1.36538
C	-2.60531	3.46622	1.18848
H	-1.53426	6.15074	-1.97772
H	0.01844	5.94849	-1.18310
C	-1.67393	6.08049	0.19358
H	-2.80364	2.67833	-1.48205
H	-3.18157	4.26712	-2.15355
C	-3.31789	4.17931	0.01820
H	0.07083	5.66584	1.44216
H	-1.45039	5.64763	2.31423
H	-2.73102	2.37859	1.10448
H	-3.06192	3.76809	2.14191
H	-1.56625	7.16685	0.31526
C	-3.17086	5.70587	0.18778
H	-4.38234	3.90839	0.01740
H	-3.64438	6.03059	1.12519
H	-3.68831	6.22832	-0.62930

$A_8C_{11}B_1$

Al	-0.89488	-0.17272	0.79218
N	-2.67241	0.87343	0.67807
N	-1.99511	-1.31410	-0.46567
O	-0.05851	1.39185	0.31381
O	0.60026	-1.17740	0.32019
C	0.64883	3.65899	0.37189
C	0.31770	4.97209	0.68705
C	-0.98234	5.39736	1.04475
C	-1.97726	4.44020	1.07515
C	-1.69695	3.07835	0.81737
C	-0.37106	2.66093	0.50093
C	-2.79889	2.15264	0.81186
C	-3.81315	-0.04973	0.60079
C	-3.45743	-1.04669	-0.54487
C	-1.50828	-2.16688	-1.32858
C	-0.15132	-2.58871	-1.44632
C	0.17120	-3.53436	-2.45429
C	1.45667	-4.00413	-2.60779
C	2.43840	-3.53873	-1.69421
C	2.18614	-2.64409	-0.66538
C	0.85970	-2.09923	-0.56357
C	-0.98562	-0.61389	2.72966
H	1.09664	5.72696	0.66858
H	-3.00157	4.72277	1.31319
H	-3.79619	2.59021	0.90289
H	-3.80254	-0.62183	1.53767
H	-3.65004	-0.53453	-1.49532
H	-2.20407	-2.63183	-2.03263
H	-0.62256	-3.88284	-3.11325
H	3.44867	-3.91258	-1.82622
H	-0.01897	-0.38724	3.20478
H	-1.74765	-0.06454	3.30106
H	-1.16001	-1.68498	2.91514
C	-4.31072	-2.30503	-0.48944
C	-5.28692	-2.54133	-1.46437
C	-4.14781	-3.24067	0.54394
C	-6.09053	-3.68276	-1.40841
H	-5.42395	-1.82498	-2.27089
C	-4.94759	-4.38109	0.59998
H	-3.38383	-3.08190	1.30073
C	-5.92293	-4.60516	-0.37581

H	-6.84379	-3.84936	-2.17392	H	4.09079	5.98930	-0.72865
H	-4.80752	-5.09758	1.40510	H	2.33708	5.96673	-0.85220
H	-6.54469	-5.49539	-0.33173	C	3.31963	4.71524	-2.31867
C	-5.19171	0.56704	0.44023	H	4.58825	2.64300	0.80133
C	-6.14086	0.42215	1.45963	H	5.39040	4.10134	0.21303
C	-5.54889	1.28754	-0.71101	C	4.65404	2.80069	-1.37378
C	-7.41850	0.97083	1.33303	H	1.17561	4.35292	-2.44198
H	-5.87827	-0.13119	2.35801	H	2.13025	3.35169	-3.53595
C	-6.82335	1.83998	-0.83862	H	3.47076	1.02682	-0.90376
H	-4.82586	1.42670	-1.51095	H	3.53049	1.41695	-2.62332
C	-7.76328	1.68073	0.18260	H	3.30309	5.51462	-3.07196
H	-8.14209	0.84222	2.13355	C	4.63302	3.91592	-2.43905
H	-7.08217	2.39509	-1.73644	H	5.59321	2.23569	-1.44749
H	-8.75690	2.10903	0.08132	H	4.72173	3.47794	-3.44341
C	-1.25087	6.84849	1.36574	H	5.49373	4.58689	-2.30609
H	-0.97595	7.50753	0.53172	C	3.23677	-2.14151	0.31744
H	-2.31025	7.01787	1.58624	H	3.16042	-1.04564	0.27258
H	-0.67455	7.18283	2.23875	C	4.71947	-2.50261	0.02064
C	1.83294	-4.98735	-3.69026	C	2.94383	-2.51651	1.80703
H	2.24534	-5.91498	-3.27168	H	4.94652	-2.29103	-1.03302
H	0.96505	-5.25746	-4.30129	C	5.05592	-3.98046	0.34013
H	2.59652	-4.57533	-4.36354	C	5.61083	-1.61276	0.91982
C	2.04752	3.20177	-0.02626	H	1.89236	-2.31335	2.02409
H	2.26157	2.34653	0.62967	C	3.26024	-4.00031	2.08367
C	3.21331	4.21403	0.16116	C	3.83103	-1.62964	2.70721
C	2.13464	2.64201	-1.48369	H	6.11979	-4.15877	0.12726
H	3.15434	4.66224	1.16222	H	4.49092	-4.67296	-0.29281
C	3.22777	5.33273	-0.91056	C	4.75336	-4.28345	1.82012
C	4.54042	3.42766	0.03383	H	5.43002	-0.55199	0.69849
H	1.29475	1.96301	-1.65291	H	6.67039	-1.80758	0.70117
C	2.10959	3.78038	-2.52386	C	5.31957	-1.90096	2.40921
C	3.45793	1.85934	-1.61950	H	2.63786	-4.64802	1.45132

H	3.00783	-4.23971	3.12639
H	3.59267	-0.57067	2.53820
H	3.61752	-1.83895	3.76506
H	4.97442	-5.33859	2.03111
C	5.62682	-3.38137	2.71554
H	5.94844	-1.25481	3.03659
H	5.43086	-3.59447	3.77599
H	6.69088	-3.59379	2.53843

$A_9C_1B_1$

Al	0.00867	1.15457	0.81042
N	1.38116	2.64577	0.41321
N	-1.13606	2.56358	-0.14936
O	1.28858	-0.03153	0.24031
O	-1.30520	-0.08615	0.38161
C	3.30477	-1.19364	-0.18893
C	4.67487	-1.12909	-0.40720
C	5.41055	0.07922	-0.43706
C	4.70464	1.25166	-0.24083
C	3.31022	1.25016	-0.00341
C	2.58678	0.02124	0.03466
C	2.63760	2.51077	0.13711
C	0.75641	3.96056	0.43512
C	-0.48570	3.84625	-0.45425
C	-2.39460	2.42998	-0.45451
C	-3.17143	1.23750	-0.32472
C	-4.54812	1.28958	-0.65901
C	-5.34836	0.16589	-0.61487
C	-4.73148	-1.05349	-0.23566
C	-3.39028	-1.17111	0.09584
C	-2.57075	0.00298	0.07067
C	-0.14992	1.35257	2.78026
H	5.21337	-2.05926	-0.56945
H	5.22361	2.20913	-0.26651
H	3.24376	3.41074	-0.01924
H	1.42946	4.75205	0.08253
H	0.45510	4.19016	1.46522
H	-1.16843	4.69056	-0.30040
H	-0.17401	3.84511	-1.50730
H	-2.91915	3.29907	-0.86697
H	-4.97083	2.24801	-0.95835
H	-5.34788	-1.94843	-0.20781
H	-0.06553	0.37057	3.27013
H	0.62016	1.99147	3.23723
H	-1.12396	1.76013	3.09133
C	-6.82954	0.22735	-0.96484
C	-7.72145	-0.14946	0.23283
C	-7.17093	-0.63516	-2.19409
H	-7.05272	1.27146	-1.22422
H	-7.51535	0.49018	1.09872
H	-8.78283	-0.04241	-0.02377
H	-7.55964	-1.18959	0.54171
H	-6.57100	-0.34329	-3.06378
H	-6.98362	-1.69891	-2.00249
H	-8.22990	-0.52936	-2.46097
C	6.91377	0.08758	-0.68356
C	7.27656	-0.47782	-2.06932
C	7.68543	-0.64924	0.42696
H	7.23425	1.13840	-0.66375
H	6.76356	0.06899	-2.86892
H	8.35676	-0.40593	-2.24770
H	6.99730	-1.53504	-2.15695
H	7.46579	-0.22416	1.41306
H	7.42367	-1.71412	0.45782
H	8.76766	-0.57975	0.26024

C	-2.75483	-2.48754	0.52313
C	-3.43057	-3.73050	-0.07318
C	-2.69247	-2.58904	2.06111
H	-1.71977	-2.46145	0.16321
H	-3.51001	-3.66638	-1.16490
H	-2.84544	-4.62587	0.16838
H	-4.43865	-3.88884	0.33029
H	-2.16294	-1.73385	2.49244
H	-3.70115	-2.61922	2.49309
H	-2.16640	-3.50187	2.36837
C	2.53989	-2.51037	-0.14267
C	2.39843	-3.00878	1.31019
C	3.13456	-3.60915	-1.03567
H	1.52901	-2.29265	-0.50499
H	1.91892	-2.25493	1.94276
H	1.78747	-3.91963	1.34979
H	3.37997	-3.24208	1.74277
H	3.27179	-3.26436	-2.06744
H	4.10460	-3.96563	-0.66702
H	2.46327	-4.47599	-1.05838

$A_9C_2B_1$

Al	0.01321	1.02666	0.70823
C	0.35279	1.21430	2.66463
O	-1.26024	-0.10113	0.05916
C	-2.57807	-0.09491	-0.00767
C	-3.26624	-1.29086	-0.36405
C	-4.65591	-1.27255	-0.42340
C	-5.43787	-0.12766	-0.15776
C	-4.75876	1.03287	0.17421
C	-3.34906	1.07544	0.25337
C	-2.72375	2.33371	0.56856
N	-1.45036	2.52923	0.67415
C	-0.94981	3.87557	0.94229
C	-0.20986	4.43945	-0.29375
C	0.27822	3.34710	-1.25488
N	0.99236	2.24428	-0.58981
C	2.25752	2.09980	-0.88399
C	3.11644	1.02493	-0.50157
C	4.49364	1.10764	-0.83159
C	5.36146	0.06391	-0.58494
C	4.81405	-1.11356	-0.01159
C	3.47636	-1.26340	0.31654
C	2.58611	-0.16231	0.09182
O	1.32250	-0.27304	0.38778
H	-0.31349	1.88987	3.21868
H	1.38439	1.52789	2.88758
H	0.24146	0.22222	3.12896
H	-5.16662	-2.19619	-0.68911
H	-5.31571	1.94602	0.38116
H	-3.40027	3.18385	0.71879
H	-0.25387	3.79825	1.78372
H	-1.76347	4.54650	1.24617
H	-0.58381	2.90822	-1.76879
H	0.91760	3.79694	-2.02398
H	2.71446	2.86270	-1.52322
H	4.86204	2.02416	-1.29099
H	5.48540	-1.94834	0.17391
H	-0.87650	5.09696	-0.86507
H	0.63585	5.05137	0.04069
C	-6.95898	-0.16502	-0.23264
C	-7.56423	-1.12244	0.81075
C	-7.46140	-0.50841	-1.64716
H	-7.31519	0.84682	0.00535
H	-7.24613	-0.85710	1.82550

H	-8.66049	-1.08980	0.77883
H	-7.25884	-2.15992	0.62711
H	-7.06865	0.19477	-2.39070
H	-7.15365	-1.51739	-1.94829
H	-8.55710	-0.47095	-1.69002
C	-2.48005	-2.56263	-0.65956
C	-2.72807	-3.07375	-2.09043
C	-2.76974	-3.66396	0.37711
H	-1.42133	-2.30175	-0.57815
H	-2.48713	-2.30585	-2.83481
H	-2.10367	-3.95160	-2.29888
H	-3.77392	-3.36965	-2.24259
H	-2.55180	-3.31879	1.39434
H	-3.82040	-3.98036	0.34930
H	-2.15171	-4.54955	0.18246
C	2.91309	-2.53120	0.94420
C	2.85027	-2.39847	2.47962
C	3.65889	-3.81149	0.54154
H	1.87981	-2.61784	0.58865
H	2.27473	-1.51615	2.77672
H	2.37264	-3.27996	2.92625
H	3.85744	-2.30791	2.90675
H	3.73596	-3.91123	-0.54766
H	4.67399	-3.84880	0.95653
H	3.12533	-4.69068	0.92217
C	6.84246	0.16086	-0.92733
C	7.25879	-0.86885	-1.99418
C	7.73381	0.04389	0.32309
H	7.00590	1.16003	-1.35429
H	6.65873	-0.76206	-2.90522
H	8.31432	-0.74223	-2.26575
H	7.13232	-1.89630	-1.63126
H	7.47255	0.80247	1.06990
H	7.63129	-0.93967	0.79809
H	8.79110	0.17526	0.06087

$A_{10}C_1B_1$

Al	-0.49825	-1.63603	0.65340
N	-2.23409	-2.73152	0.40783
N	0.16947	-3.32734	-0.30153
O	-1.45213	-0.19551	0.03036
O	1.09471	-0.82369	0.14332
C	-3.11969	1.45230	-0.36502
C	-4.48059	1.73570	-0.50491
C	-5.48059	0.75706	-0.40626
C	-5.10890	-0.55098	-0.16805
C	-3.74935	-0.89503	-0.01003
C	-2.72427	0.10038	-0.10452
C	-3.42342	-2.28200	0.18155
C	-1.97165	-4.16196	0.46303
C	-0.81373	-4.40141	-0.51045
C	1.39310	-3.54213	-0.68555
C	2.47792	-2.61172	-0.62487
C	3.75500	-3.05861	-1.03702
C	4.83626	-2.20376	-1.02058
C	4.64698	-0.88038	-0.58563
C	3.41042	-0.37978	-0.17891
C	2.27492	-1.25853	-0.19533
C	-0.30534	-1.76990	2.62414
H	-4.77145	2.76013	-0.72119
H	-5.85788	-1.33757	-0.10191
H	-4.25384	-2.99410	0.10906
H	-2.85025	-4.76199	0.19657
H	-1.66179	-4.42593	1.48201
H	-0.36484	-5.39110	-0.36638

H	-1.19178	-4.34139	-1.53957
H	1.63554	-4.52504	-1.10417
H	3.86730	-4.09116	-1.36146
H	5.50757	-0.21794	-0.54220
H	-0.27807	-0.76268	3.06736
H	-1.12062	-2.30512	3.13252
H	0.63279	-2.25846	2.92796
H	5.82216	-2.54275	-1.32330
H	-6.52454	1.02728	-0.53212
C	3.30837	1.03532	0.26965
C	2.57831	1.40177	1.41413
C	4.00862	2.04291	-0.41802
C	2.56660	2.72225	1.86060
H	2.02650	0.64531	1.95958
C	3.99567	3.36444	0.02866
H	4.55796	1.78912	-1.32096
C	3.27623	3.70967	1.17379
H	1.99830	2.98000	2.75056
H	4.54425	4.12415	-0.52294
H	3.26237	4.73859	1.52395
C	-2.13269	2.56106	-0.47539
C	-2.42297	3.81438	0.09388
C	-0.92530	2.42097	-1.18198
C	-1.55034	4.89251	-0.05018
H	-3.33447	3.93932	0.67247
C	-0.05495	3.49993	-1.32823
H	-0.67028	1.46317	-1.61852
C	-0.36222	4.74066	-0.76631
H	-1.79696	5.84848	0.40547
H	0.87329	3.36571	-1.87671
H	0.32318	5.57701	-0.87683

$A_{10}C_2B_1$

Al	0.66528	-1.39631	0.56851
C	0.24993	-1.79744	2.47930
O	1.40516	0.18695	0.04985
C	2.58678	0.75211	0.18061
C	2.75194	2.13595	-0.14091
C	4.01199	2.71653	0.03164
C	5.12767	1.99708	0.48116
C	4.98503	0.65004	0.75420
C	3.73518	0.01338	0.60485
C	3.67610	-1.40975	0.82897
N	2.61479	-2.13753	0.72641
C	2.70890	-3.58509	0.89773
C	2.44825	-4.30572	-0.44598
C	1.65980	-3.44916	-1.44632
N	0.46290	-2.81313	-0.86914
C	-0.69224	-3.19003	-1.34511
C	-1.97575	-2.62939	-1.05591
C	-3.11761	-3.26557	-1.59835
C	-4.37295	-2.72513	-1.42027
C	-4.49925	-1.52004	-0.70457
C	-3.41075	-0.84020	-0.16294
C	-2.09797	-1.40054	-0.32661
O	-1.04936	-0.78737	0.13288
H	1.07175	-2.19260	3.09188
H	-0.59706	-2.49144	2.59261
H	-0.07054	-0.86196	2.96441
H	4.12108	3.77714	-0.17862
H	5.83555	0.05897	1.08783
H	4.62499	-1.89513	1.08713
H	1.94338	-3.87355	1.62537
H	3.68392	-3.87352	1.31042
H	2.30260	-2.64411	-1.81792

H	1.37761	-4.06241	-2.31027
H	-0.69406	-4.00964	-2.07123
H	-2.98613	-4.19215	-2.15361
H	-5.49240	-1.10557	-0.55051
H	6.08420	2.49443	0.60949
H	-5.25475	-3.21666	-1.81947
H	3.39996	-4.56541	-0.92516
H	1.91739	-5.24521	-0.25390
C	1.63635	2.96547	-0.67447
C	0.33734	2.91836	-0.13929
C	1.88391	3.86226	-1.73013
C	-0.66883	3.74756	-0.63432
H	0.11404	2.23263	0.66842
C	0.87707	4.68966	-2.22646
H	2.87291	3.89592	-2.17976
C	-0.40536	4.63697	-1.67813
H	-1.66379	3.69306	-0.20125
H	1.09462	5.36881	-3.04731
H	-1.19382	5.27799	-2.06456
C	-3.62865	0.43702	0.56801
C	-4.51070	1.40209	0.05159
C	-3.01268	0.69791	1.80503
C	-4.77624	2.58240	0.74713
H	-4.97888	1.23229	-0.91448
C	-3.27885	1.87663	2.50073
H	-2.33168	-0.03318	2.22530
C	-4.16131	2.82503	1.97682
H	-5.45991	3.31418	0.32378
H	-2.79690	2.05298	3.45919
H	-4.36567	3.74365	2.52082

$A_{10}C_3B_1$

Al	0.99320	-0.61927	0.65253
C	0.82094	-1.15235	2.56874
O	0.74582	1.09994	0.09727
C	1.41077	2.22688	0.23965
C	0.79046	3.46589	-0.11474
C	1.50775	4.65077	0.07466
C	2.81795	4.67672	0.57203
C	3.43917	3.48016	0.87518
C	2.76123	2.25423	0.70950
C	3.49524	1.03905	0.96378
N	3.01847	-0.15562	0.84808
C	3.88467	-1.31075	1.05150
C	4.16155	-2.05884	-0.28802
C	3.01064	-1.80001	-1.29310
N	1.65293	-1.93663	-0.74025
C	0.90948	-2.89112	-1.23067
C	-0.47798	-3.13009	-0.97890
C	-1.06184	-4.29687	-1.52727
C	-2.41175	-4.53779	-1.38862
C	-3.20427	-3.59506	-0.70795
C	-2.68936	-2.42083	-0.16333
C	-1.28074	-2.16520	-0.28507
O	-0.75946	-1.07003	0.17938
C	5.45454	-1.52628	-0.93202
C	4.30392	-3.56168	0.00303
H	1.70766	-1.01369	3.20233
H	0.50203	-2.19920	2.68873
H	0.02117	-0.54833	3.02528
H	1.01665	5.59130	-0.16048
H	4.46215	3.46613	1.24557
H	4.54406	1.16148	1.25953
H	3.36496	-1.98358	1.74230
H	4.83294	-1.01987	1.52330

H	3.09663	-0.77665	-1.67398
H	3.13227	-2.47471	-2.14996
H	1.37934	-3.57841	-1.94117
H	-0.42396	-5.00296	-2.05510
H	-4.26544	-3.79656	-0.58474
H	5.42103	-0.43742	-1.06117
H	5.61745	-1.97167	-1.92123
H	6.32637	-1.76677	-0.31241
H	3.36500	-3.99006	0.37297
H	5.07172	-3.74048	0.76589
H	4.59907	-4.11271	-0.89828
H	3.33117	5.62307	0.71284
H	-2.86296	-5.43893	-1.79220
C	-0.57755	3.52830	-0.69975
C	-1.64935	2.76978	-0.19746
C	-0.83188	4.40147	-1.77335
C	-2.92748	2.89316	-0.74162
H	-1.48340	2.08379	0.62362
C	-2.10953	4.52277	-2.31892
H	-0.01293	4.97643	-2.19783
C	-3.16495	3.76928	-1.80280
H	-3.73914	2.29767	-0.33291
H	-2.27653	5.20079	-3.15244
H	-4.16177	3.85893	-2.22731
C	-3.59921	-1.46800	0.52723
C	-4.85486	-1.16601	-0.02829
C	-3.26548	-0.88601	1.76321
C	-5.75041	-0.32126	0.62863
H	-5.12356	-1.58403	-0.99489
C	-4.16079	-0.04287	2.42038
H	-2.30428	-1.10610	2.21296
C	-5.40751	0.24401	1.85827
H	-6.71377	-0.10126	0.17511
H	-3.88392	0.38902	3.37892
H	-6.10256	0.90295	2.37231

$A_{10}C_6B_1$

Al	-1.14487	-0.07496	0.66031
O	0.19973	-1.27616	0.22709
O	-0.13059	1.31663	0.01253
N	-2.47540	-1.46125	-0.08762
N	-2.81668	1.06176	0.11913
C	-3.82166	-1.07235	0.08261
C	-4.91773	-1.93737	0.17645
C	-6.19501	-1.41629	0.37521
C	-6.38272	-0.03664	0.50454
C	-5.29505	0.83179	0.43319
C	-4.01127	0.32104	0.21036
C	-2.17680	-2.58986	-0.67649
C	-0.87029	-3.14018	-0.81410
C	0.28586	-2.46697	-0.29359
C	1.55156	-3.14031	-0.37310
C	1.60359	-4.38505	-0.99965
C	0.47648	-5.01738	-1.55434
C	-0.74989	-4.39992	-1.45028
C	-2.81500	2.31733	-0.22836
C	-1.64968	3.13005	-0.37688
C	-0.33002	2.58085	-0.26457
C	0.78462	3.45623	-0.47689
C	0.53436	4.79626	-0.77673
C	-0.76013	5.32979	-0.88707
C	-1.84017	4.49548	-0.69588
C	-1.28536	-0.07806	2.63196
H	-4.77357	-3.01236	0.12846
H	-7.04224	-2.09091	0.45707

H	-7.37636	0.36258	0.68591
H	-5.44476	1.89721	0.57779
H	-2.99159	-3.18239	-1.09911
H	2.55932	-4.90069	-1.03751
H	-1.64678	-4.87580	-1.84072
H	-3.76737	2.80350	-0.45496
H	1.38495	5.44894	-0.95348
H	-2.85621	4.87326	-0.78733
H	-0.28965	0.02293	3.08820
H	-1.71431	-1.00933	3.03067
H	-1.89492	0.74506	3.03152
C	2.79580	-2.56705	0.20784
C	2.80697	-1.94580	1.46931
C	4.02090	-2.70986	-0.46885
C	4.00097	-1.50456	2.03749
H	1.87666	-1.81990	2.01054
C	5.21548	-2.26735	0.10003
H	4.03564	-3.16260	-1.45686
C	5.21104	-1.66494	1.35925
H	3.98415	-1.03282	3.01655
H	6.14850	-2.39078	-0.44451
H	6.13972	-1.31851	1.80526
C	2.19406	2.99037	-0.37250
C	3.15034	3.80001	0.26689
C	2.62678	1.78227	-0.94552
C	4.49292	3.42678	0.31849
H	2.83352	4.72344	0.74454
C	3.96978	1.41203	-0.89748
H	1.90835	1.13405	-1.43261
C	4.90938	2.23103	-0.26810
H	5.21055	4.06814	0.82436
H	4.28031	0.47243	-1.34515
H	5.95415	1.93396	-0.22689
H	0.57561	-5.98826	-2.02966
H	-0.89968	6.37788	-1.13312

$A_{11}C_2B_1$

Al	-0.02517	1.05827	0.59984
C	0.42652	1.23297	2.53512
O	-1.31686	-0.09353	0.02207
C	-2.63801	-0.11834	0.00442
C	-3.33344	-1.32222	-0.34747
C	-4.72600	-1.28954	-0.33884
C	-5.49952	-0.15399	-0.01467
C	-4.81775	1.00214	0.30768
C	-3.40576	1.04191	0.32000
C	-2.78346	2.30495	0.61985
N	-1.51131	2.52581	0.66583
C	-1.02812	3.88388	0.90613
C	-0.41309	4.47596	-0.38274
C	0.07224	3.39721	-1.36217
N	0.84956	2.32644	-0.71785
C	2.12282	2.26555	-0.99768
C	3.04551	1.24388	-0.61333
C	4.41728	1.45680	-0.89759
C	5.35606	0.48264	-0.64302
C	4.88747	-0.74985	-0.12417
C	3.55798	-1.04096	0.15341
C	2.58645	-0.00038	-0.07164
O	1.31490	-0.18771	0.16628
C	-2.56752	-2.61266	-0.70990
C	-1.65462	-2.36347	-1.93459
C	-1.72009	-3.07444	0.49950
C	-3.51687	-3.77183	-1.07518
C	3.13470	-2.42709	0.68212

C	4.33557	-3.37790	0.85911
C	2.16494	-3.08848	-0.32587
C	2.44923	-2.28938	2.06202
H	-0.20614	1.91258	3.12261
H	1.46970	1.54424	2.69903
H	0.34026	0.24457	3.01179
H	-5.26931	-2.19292	-0.59605
H	-5.36554	1.90999	0.55622
H	-3.46730	3.14113	0.80948
H	-0.26158	3.82095	1.68499
H	-1.83169	4.52914	1.28344
H	-0.79377	2.92442	-1.83701
H	0.66392	3.86269	-2.15951
H	2.54000	3.06424	-1.62029
H	4.71947	2.41406	-1.31988
H	5.63590	-1.51441	0.05810
H	-2.24948	-2.06836	-2.80855
H	-1.11488	-3.28290	-2.19503
H	-0.92196	-1.58005	-1.73532
H	-0.98582	-2.31997	0.78512
H	-1.18495	-4.00040	0.25380
H	-2.36114	-3.27986	1.36615
H	-4.14429	-3.53903	-1.94403
H	-4.17347	-4.05189	-0.24281
H	-2.92131	-4.65591	-1.33074
H	3.97600	-4.34518	1.22902
H	5.06219	-3.00007	1.58850
H	4.86200	-3.56558	-0.08437
H	1.27643	-2.47584	-0.48502
H	1.84651	-4.07097	0.04578
H	2.65727	-3.24168	-1.29466
H	1.56246	-1.65722	2.00672
H	3.13688	-1.85242	2.79696
H	2.14743	-3.27747	2.43284
C	6.82834	0.68709	-0.90806
H	7.41981	0.61904	0.01489
H	7.22581	-0.07001	-1.59684
H	7.02025	1.67058	-1.35043
C	-7.00797	-0.22427	-0.02940
H	-7.39099	-0.51609	-1.01618
H	-7.38623	-0.96274	0.68987
H	-7.45397	0.74298	0.22584
H	0.41578	5.14044	-0.11224
H	-1.15626	5.08567	-0.91107

$A_{11}C_3B_1$

Al	-0.01429	0.70309	0.65751
C	0.42758	0.81087	2.60025
O	-1.27827	-0.45822	0.03582
C	-2.59863	-0.51745	0.03678
C	-3.26803	-1.72489	-0.35173
C	-4.66064	-1.73063	-0.31885
C	-5.45827	-0.63033	0.06320
C	-4.80204	0.53056	0.41964
C	-3.39161	0.60875	0.40957
C	-2.79784	1.87675	0.74533
N	-1.53058	2.12904	0.77272
C	-1.06947	3.48458	1.05094
C	-0.52409	4.17669	-0.23360
C	0.01573	3.10907	-1.22022
N	0.82721	2.04928	-0.60346
C	2.09974	2.02707	-0.89410
C	3.04899	1.01543	-0.54904
C	4.41395	1.27270	-0.82943
C	5.37754	0.31326	-0.61451

C	4.94160	-0.94912	-0.14117
C	3.62066	-1.28349	0.12843
C	2.62291	-0.25976	-0.05477
O	1.35691	-0.48877	0.17688
C	-2.47490	-2.97937	-0.77693
C	-1.58800	-2.65825	-2.00400
C	-1.59697	-3.46673	0.40022
C	-3.39974	-4.14771	-1.17453
C	-1.65684	4.93225	-0.95115
C	0.58653	5.16026	0.16928
C	3.23347	-2.69844	0.60597
C	4.45759	-3.62615	0.74141
C	2.27396	-3.34450	-0.42156
C	2.55250	-2.62917	1.99326
H	-0.21967	1.45594	3.21042
H	1.46416	1.13447	2.78106
H	0.35725	-0.19656	3.03823
H	-5.18419	-2.63756	-0.60301
H	-5.36989	1.41264	0.71197
H	-3.49855	2.68573	0.98326
H	-0.26531	3.40595	1.79031
H	-1.86813	4.09447	1.49439
H	-0.83262	2.61723	-1.70798
H	0.59312	3.61086	-2.00721
H	2.49291	2.85320	-1.49553
H	4.69077	2.25244	-1.21612
H	5.70936	-1.70114	0.01026
H	-2.20400	-2.34697	-2.85746
H	-1.02716	-3.55162	-2.30709
H	-0.87409	-1.86274	-1.78537
H	-0.87579	-2.70645	0.70326
H	-1.04450	-4.36942	0.11027
H	-2.21842	-3.72062	1.26827
H	-4.04758	-3.89656	-2.02304
H	-4.03467	-4.47898	-0.34413
H	-2.78571	-5.00442	-1.47552
H	-2.50961	4.27419	-1.15840
H	-1.31469	5.34175	-1.90975
H	-2.01564	5.76999	-0.34169
H	1.45108	4.63774	0.59535
H	0.22501	5.86935	0.92431
H	0.93049	5.74323	-0.69385
H	4.12316	-4.61506	1.07605
H	5.17895	-3.25885	1.48131
H	4.98322	-3.76534	-0.21089
H	1.36950	-2.74852	-0.55135
H	1.98269	-4.34836	-0.08654
H	2.76349	-3.44756	-1.39836
H	1.65079	-2.01651	1.96625
H	3.23374	-2.20420	2.74108
H	2.27582	-3.63737	2.32775
C	6.84330	0.56427	-0.87577
H	7.43898	0.48212	0.04333
H	7.25820	-0.15988	-1.58921
H	7.00842	1.56606	-1.28675
C	-6.96439	-0.74100	0.07060
H	-7.35711	-1.00083	-0.92130
H	-7.31002	-1.51949	0.76358
H	-7.43128	0.20200	0.37463

$A_{11}C_5B_1$

O	-1.16905	-0.75803	0.13812
O	1.44155	-0.40698	0.55586
N	-1.69955	1.85525	0.46699
N	0.76730	2.11883	-0.19315

C	-1.24581	3.23782	0.58222
C	-2.24489	4.37265	0.34536
C	-1.54014	5.72753	0.53503
C	-0.29283	5.85454	-0.35258
C	0.68168	4.67857	-0.16261
C	-0.04723	3.34398	-0.38398
C	-2.91229	1.53125	0.16880
C	-3.36644	0.18485	-0.06044
C	-4.74400	0.00416	-0.31587
C	-5.26494	-1.24226	-0.59613
C	-4.35851	-2.32385	-0.63122
C	-2.98936	-2.21835	-0.39960
C	-2.46070	-0.91919	-0.09016
C	1.91115	2.04534	-0.81328
C	2.85674	0.97586	-0.76185
C	4.08270	1.15963	-1.44853
C	5.08006	0.21248	-1.39194
C	4.83528	-0.93817	-0.60289
C	3.65767	-1.19162	0.08962
C	2.60102	-0.21778	-0.01609
H	-0.84637	3.34031	1.60278
H	-2.65487	4.30537	-0.67234
H	-3.08888	4.28935	1.04089
H	-2.24082	6.54295	0.31923
H	-1.24924	5.83521	1.58930
H	0.22509	6.79773	-0.14108
H	-0.60113	5.89729	-1.40712
H	1.52197	4.78868	-0.85688
H	1.10133	4.68964	0.85269
H	-0.44943	3.32354	-1.40861
H	-3.66976	2.31271	0.06263
H	-5.39541	0.87641	-0.28714
H	-4.77348	-3.30058	-0.85720
H	2.21515	2.89268	-1.43364
H	4.22700	2.07690	-2.01764
H	5.63984	-1.66406	-0.54244
Al	-0.11632	0.58289	0.83331
C	-0.08827	0.74590	2.81423
H	-0.00338	-0.24970	3.27545
H	0.77569	1.31936	3.18348
H	-0.98627	1.21003	3.24757
C	6.39517	0.37580	-2.11535
H	7.24592	0.35316	-1.42147
H	6.55886	-0.42809	-2.84541
H	6.43549	1.32676	-2.65732
C	-6.73508	-1.46362	-0.86073
H	-7.29281	-0.52170	-0.82215
H	-6.90682	-1.91048	-1.84886
H	-7.18211	-2.14271	-0.12255
C	3.49394	-2.46044	0.95201
C	3.18995	-2.06505	2.41732
C	2.34472	-3.33010	0.39442
C	4.76848	-3.32796	0.96585
H	4.01425	-1.47817	2.84186
H	2.27490	-1.47526	2.49317
H	3.07032	-2.96605	3.03269
H	2.56396	-3.65400	-0.63078
H	2.21836	-4.22982	1.01056
H	1.40342	-2.78118	0.38789
H	4.59845	-4.20473	1.60153
H	5.03285	-3.69718	-0.03230
H	5.63369	-2.79174	1.37406
C	-2.07771	-3.46196	-0.45964
C	-0.98482	-3.26456	-1.53734

C	-1.43605	-3.70276	0.92752
C	-2.85430	-4.74208	-0.83035
H	-1.43992	-3.13860	-2.52821
H	-0.36722	-2.39005	-1.32937
H	-0.33338	-4.14659	-1.58052
H	-2.21020	-3.89345	1.68156
H	-0.77902	-4.58099	0.89469
H	-0.84624	-2.84495	1.25344
H	-2.15643	-5.58683	-0.86441
H	-3.62811	-4.98983	-0.09400
H	-3.32860	-4.67112	-1.81659

$A_{11}C_8B_1$

Al	0.08184	0.17382	-0.66746
C	0.57488	0.48901	-2.57314
O	-0.90551	-1.24656	-0.10798
C	-2.16915	-1.62234	-0.14940
C	-2.54366	-2.94977	0.24184
C	-3.89272	-3.28586	0.16328
C	-4.91474	-2.41194	-0.27024
C	-4.54104	-1.13467	-0.63267
C	-3.18939	-0.72234	-0.57566
C	-2.90502	0.63830	-0.93150
N	-1.74178	1.21886	-0.90043
C	-1.68692	2.58687	-1.31020
C	-1.91562	2.93838	-2.64496
C	-1.84781	4.27273	-3.04434
C	-1.55367	5.26426	-2.10784
C	-1.33862	4.91536	-0.77522
C	-1.39593	3.57839	-0.35318
C	-1.27332	3.23517	1.08797
C	-2.08356	3.90849	2.01711
C	-2.02080	3.63377	3.38201
C	-1.13323	2.66571	3.84952
C	-0.31109	1.99299	2.94724
C	-0.36791	2.27024	1.57465
N	0.55374	1.57629	0.71643
C	1.82029	1.87428	0.91808
C	2.96213	1.15993	0.45353
C	4.23604	1.76131	0.62430
C	5.38947	1.06884	0.33894
C	5.25358	-0.28109	-0.07728
C	4.04597	-0.94680	-0.23480
C	2.83862	-0.19373	-0.00407
O	1.65650	-0.72658	-0.14949
H	-0.23995	0.22703	-3.26463
H	1.44676	-0.10519	-2.87519
H	0.82530	1.53848	-2.78592
H	-4.19675	-4.28727	0.44993
H	-5.28926	-0.41787	-0.96667
H	-3.76820	1.23638	-1.23983
H	-2.13097	2.15571	-3.36575
H	-2.02002	4.53265	-4.08514
H	-1.49196	6.30551	-2.41158
H	-1.11764	5.68649	-0.04226
H	-2.78528	4.65169	1.64887
H	-2.66906	4.16677	4.07191
H	-1.07693	2.43202	4.90899
H	0.38454	1.23533	3.29610
H	2.02909	2.74521	1.54424
H	4.28407	2.78816	0.98271
H	6.17433	-0.82077	-0.27467
C	6.76022	1.68687	0.47360
H	6.69939	2.70944	0.86105
H	7.40012	1.11020	1.15434

H	7.28092	1.73003	-0.49243
C	-6.34959	-2.87922	-0.32461
H	-6.70558	-3.20992	0.65994
H	-7.01555	-2.07957	-0.66588
H	-6.47322	-3.72826	-1.00981
C	-1.48980	-3.97238	0.71780
C	-0.47516	-4.24500	-0.41820
C	-0.75293	-3.43520	1.96828
C	-2.12309	-5.32431	1.10415
H	-0.98249	-4.64838	-1.30368
H	0.05499	-3.33666	-0.70798
H	0.26532	-4.98617	-0.09188
H	-1.45740	-3.26911	2.79328
H	-0.00826	-4.16622	2.30834
H	-0.24000	-2.49541	1.75835
H	-1.33387	-6.00688	1.43990
H	-2.84243	-5.22802	1.92635
H	-2.63010	-5.80468	0.25878
C	3.99294	-2.43504	-0.63778
C	3.29539	-3.24356	0.48241
C	3.22515	-2.61019	-1.96930
C	5.39891	-3.03623	-0.83529
H	3.85905	-3.17249	1.42130
H	2.28092	-2.88380	0.66200
H	3.24199	-4.30410	0.20480
H	3.72258	-2.06433	-2.78078
H	3.19653	-3.67106	-2.24961
H	2.19934	-2.24859	-1.89114
H	5.30236	-4.09333	-1.10888
H	5.95414	-2.54082	-1.64088
H	6.00437	-2.99019	0.07783

$A_{13}C_2B_1$

Al	-0.00685	1.04638	0.64034
C	0.41837	1.20404	2.57959
O	-1.31350	-0.07545	0.03413
C	-2.63140	-0.07619	-0.00575
C	-3.33932	-1.26762	-0.37801
C	-4.73182	-1.21986	-0.39634
C	-5.44843	-0.05939	-0.07302
C	-4.79031	1.09682	0.27350
C	-3.38039	1.09919	0.30636
C	-2.73867	2.35151	0.62471
N	-1.46466	2.54453	0.68779
C	-0.95668	3.89167	0.94408
C	-0.30521	4.47470	-0.33094
C	0.16661	3.39195	-1.31194
N	0.91337	2.29982	-0.66464
C	2.18464	2.21029	-0.93435
C	3.08002	1.16422	-0.53850
C	4.45552	1.35569	-0.80724
C	5.33272	0.33959	-0.52737
C	4.87136	-0.88789	-0.01774
C	3.52878	-1.13773	0.23775
C	2.58769	-0.07161	-0.00336
O	1.31140	-0.22780	0.21666
H	-0.21891	1.88415	3.16102
H	1.46062	1.50823	2.76090
H	0.31898	0.21343	3.04848
H	-5.29834	-2.10172	-0.66607
H	-5.33741	2.00299	0.51632
H	-3.40910	3.19849	0.81091
H	-0.20708	3.80751	1.73702
H	-1.75356	4.55154	1.30896
H	-0.70330	2.93944	-1.79908

H	0.77781	3.84856	-2.09908
H	2.62719	2.99667	-1.55412
H	4.79948	2.29739	-1.22469
H	5.60836	-1.65788	0.17137
H	-1.02300	5.10752	-0.86655
H	0.53646	5.11470	-0.04257
C	-2.59249	-2.57040	-0.73459
C	-1.65353	-2.33038	-1.94125
C	-1.77859	-3.05423	0.48916
C	-3.55976	-3.70638	-1.12390
H	-2.22666	-2.01776	-2.82335
H	-0.90732	-1.56453	-1.72476
H	-1.12964	-3.25966	-2.19768
H	-2.44049	-3.25203	1.34152
H	-1.25822	-3.98916	0.24724
H	-1.03425	-2.31729	0.79396
H	-2.97826	-4.60081	-1.37437
H	-4.23620	-3.97808	-0.30484
H	-4.16591	-3.45548	-2.00271
C	3.06724	-2.51356	0.76015
C	2.36037	-2.35883	2.12733
C	2.10306	-3.15332	-0.26692
C	4.24633	-3.48675	0.95860
H	3.04245	-1.93231	2.87323
H	1.48483	-1.71287	2.05597
H	2.03633	-3.34114	2.49369
H	2.61041	-3.31815	-1.22574
H	1.75754	-4.12807	0.09999
H	1.23032	-2.52292	-0.44313
H	3.86127	-4.44576	1.32341
H	4.78459	-3.68692	0.02449
H	4.96708	-3.12222	1.70041
Cl	-7.21521	-0.09407	-0.12114
Cl	7.06299	0.54898	-0.82433

A$_{13}$C$_5$B$_1$

O	-1.06088	-1.36498	0.33995
O	1.47832	-1.15049	0.95583
N	-1.45457	1.29932	0.35703
N	1.08969	1.35239	-0.05065
C	-0.91564	2.65446	0.42450
C	-1.79233	3.83144	-0.00881
C	-1.01774	5.14752	0.18552
C	0.33896	5.13115	-0.53605
C	1.18945	3.90657	-0.15411
C	0.39251	2.61527	-0.39429
C	-2.65073	1.02680	-0.04699
C	-3.16317	-0.30939	-0.20134
C	-4.51166	-0.47456	-0.59686
C	-5.06240	-1.72637	-0.80454
C	-4.21680	-2.84388	-0.61823
C	-2.88574	-2.73897	-0.24012
C	-2.32716	-1.44743	-0.01348
C	2.25588	1.13607	-0.59356
C	3.06885	-0.02766	-0.43599
C	4.33149	-0.06619	-1.08387
C	5.16995	-1.15625	-0.97233
C	4.71948	-2.24634	-0.18429
C	3.49704	-2.26772	0.46495
C	2.63001	-1.13695	0.34696
H	-0.63129	2.80974	1.47641
H	-2.07850	3.71823	-1.06408
H	-2.71916	3.85392	0.57731
H	-1.62312	5.98965	-0.17056
H	-0.85579	5.31080	1.26012

H	0.89460	6.04957	-0.31167
H	0.17139	5.12512	-1.62273
H	2.11727	3.90869	-0.73703
H	1.47700	3.95453	0.90508
H	0.11237	2.56393	-1.45777
H	-3.33566	1.83985	-0.30419
H	-5.12195	0.41668	-0.73634
H	-4.62932	-3.83932	-0.77950
H	2.67342	1.90928	-1.24542
H	4.63490	0.79503	-1.67743
H	5.37020	-3.11476	-0.08589
Al	0.00046	-0.02453	1.00901
C	-0.13129	0.34241	2.95595
H	0.00170	-0.59179	3.52166
H	0.65453	1.02375	3.31696
H	-1.09251	0.76958	3.27703
C	-6.50368	-1.91270	-1.21514
H	-7.01072	-0.94967	-1.33811
H	-6.58795	-2.45557	-2.16585
H	-7.06631	-2.48935	-0.46891
C	-2.01244	-3.95145	-0.05460
H	-1.13877	-3.91865	-0.71705
H	-1.61959	-4.00721	0.96801
H	-2.56800	-4.87207	-0.26153
C	3.04559	-3.44162	1.29174
H	2.85433	-3.14689	2.33102
H	2.10381	-3.85620	0.91211
H	3.79843	-4.23675	1.29225
C	6.51695	-1.21048	-1.65228
H	7.33251	-1.33321	-0.92720
H	6.58432	-2.05423	-2.35191
H	6.71418	-0.29443	-2.21934

$A_{14}C_1B_1$

Al	0.00087	0.55452	0.76299
N	1.37818	2.01498	0.26974
N	-1.14937	1.92785	-0.24717
O	1.25789	-0.67102	0.22332
O	-1.33248	-0.69337	0.42435
C	3.25251	-1.85808	-0.18421
C	4.61496	-1.82740	-0.44300
C	5.35938	-0.63017	-0.56118
C	4.67246	0.55986	-0.40936
C	3.28422	0.58756	-0.13265
C	2.55041	-0.62876	-0.01388
C	2.62600	1.85929	-0.03342
C	0.76241	3.33373	0.25232
C	-0.49624	3.19467	-0.60963
C	-2.40756	1.78546	-0.55129
C	-3.18889	0.60381	-0.36715
C	-4.56383	0.63677	-0.71645
C	-5.36412	-0.48286	-0.61399
C	-4.75425	-1.67846	-0.15652
C	-3.41665	-1.77194	0.18974
C	-2.59331	-0.60718	0.09878
C	-0.11141	0.84888	2.72330
H	5.13731	-2.77597	-0.56304
H	5.20071	1.50799	-0.49965
H	3.23344	2.74671	-0.24605
H	1.43415	4.10648	-0.14192
H	0.48190	3.60428	1.27834
H	-1.17111	4.04823	-0.47352
H	-0.20388	3.15431	-1.66747
H	-2.92646	2.63601	-1.00723
H	-4.98374	1.57598	-1.07380

H	-5.37101	-2.57291	-0.07412
H	-0.15135	-0.11650	3.25006
H	0.74135	1.39955	3.14700
H	-1.01972	1.39287	3.02509
C	-6.83022	-0.46178	-0.97407
H	-7.06284	-1.18267	-1.76904
H	-7.46246	-0.72147	-0.11454
H	-7.13997	0.52855	-1.32454
C	6.84185	-0.67274	-0.84506
H	7.39225	-1.19039	-0.04820
H	7.06048	-1.20516	-1.78010
H	7.25972	0.33580	-0.93305
C	-2.79910	-3.05890	0.66748
H	-1.96272	-3.36109	0.02545
H	-2.38647	-2.95182	1.67827
H	-3.53564	-3.86902	0.68008
C	2.49035	-3.15162	-0.07109
H	2.02519	-3.25528	0.91690
H	1.67272	-3.19710	-0.80069
H	3.14803	-4.01168	-0.23447

$A_{14}C_1B_2$

Al	-0.01707	0.18407	0.73941
C	0.40530	0.32196	2.66324
O	-1.46997	-0.89357	0.68234
C	-2.70517	-0.78421	0.19801
C	-3.70291	-1.70506	0.60632
C	-4.99553	-1.56168	0.10492
C	-5.35774	-0.53694	-0.78555
C	-4.36144	0.35956	-1.17290
C	-3.04653	0.24261	-0.70447
C	-1.96916	1.16192	-1.20861
N	-1.08335	1.76895	-0.13706
C	-0.23369	2.79753	-0.81185
C	0.97059	3.22649	0.01686
N	1.82187	2.08025	0.35777
C	2.64107	1.65024	-0.80414
C	3.23537	0.27759	-0.62898
C	4.61766	0.06373	-0.67636
C	5.16581	-1.21643	-0.56929
C	4.27804	-2.29068	-0.40878
C	2.89326	-2.12083	-0.35058
C	2.36418	-0.81432	-0.45804
O	1.02737	-0.63591	-0.44121
C	-3.33893	-2.80676	1.56697
C	-6.77430	-0.41963	-1.29912
C	-1.91807	2.41784	0.91160
C	2.69619	2.40510	1.49200
C	6.65903	-1.44482	-0.62143
C	1.95965	-3.29141	-0.17968
H	-0.16494	-0.43885	3.21289
H	0.15209	1.28976	3.11830
H	1.46675	0.14381	2.87779
H	-5.75589	-2.27405	0.42391
H	-4.60446	1.16525	-1.86486
H	-2.41913	1.98489	-1.77961
H	-1.28400	0.62898	-1.88048
H	-0.85356	3.67251	-1.05063
H	0.09460	2.36739	-1.76089
H	1.52209	4.00225	-0.54347
H	0.64047	3.69382	0.94979
H	3.43614	2.39087	-0.99655
H	1.99154	1.64344	-1.68515
H	5.27763	0.92030	-0.80818
H	4.68029	-3.29969	-0.32422

H	-4.20028	-3.45076	1.77422
H	-2.97540	-2.40425	2.52119
H	-2.52973	-3.43240	1.17025
H	-7.49807	-0.31011	-0.48039
H	-7.07547	-1.30769	-1.87105
H	-6.88935	0.44821	-1.95793
H	-2.57768	3.17063	0.46099
H	-1.27462	2.89760	1.65018
H	-2.53004	1.66874	1.41439
H	3.35914	3.25888	1.26638
H	3.31959	1.54168	1.73328
H	2.09410	2.65208	2.36957
H	6.93818	-2.12573	-1.43638
H	7.03537	-1.89218	0.30835
H	7.20128	-0.50569	-0.77697
H	2.51402	-4.23549	-0.14822
H	1.23221	-3.34626	-0.99859
H	1.37404	-3.21019	0.74494

$A_{14}C_1B_3$

Al	-0.28807	-0.97105	0.79271
C	0.40726	-0.64339	2.60742
O	-1.87512	-1.76374	1.06412
C	-3.07305	-1.66908	0.48606
C	-4.14597	-2.44490	0.99159
C	-5.39850	-2.33107	0.39491
C	-5.64776	-1.47218	-0.68843
C	-4.58116	-0.71072	-1.16294
C	-3.30154	-0.79509	-0.59366
C	-2.15646	-0.03986	-1.21668
N	-1.17286	0.61891	-0.26308
C	-0.22702	1.40316	-1.11803
C	1.07991	1.80355	-0.43754
N	1.87140	0.64393	-0.02510
C	2.48698	-0.06732	-1.17206
C	2.83238	-1.50280	-0.86332
C	4.13923	-1.98495	-0.99937
C	4.45672	-3.32621	-0.77518
C	3.41237	-4.18725	-0.41230
C	2.09345	-3.75158	-0.27271
C	1.80071	-2.38924	-0.50129
O	0.51956	-1.96397	-0.42640
C	-3.89506	-3.37175	2.15235
C	-7.02135	-1.38823	-1.31382
C	-1.85926	1.49327	0.77052
C	2.85426	0.96210	1.04047
C	5.87427	-3.83469	-0.90764
C	0.98235	-4.70031	0.09879
H	-0.19287	-1.22564	3.31776
H	0.35979	0.40195	2.94151
H	1.44994	-0.96228	2.72197
H	-6.21627	-2.93368	0.78849
H	-4.73771	-0.04001	-2.00734
H	-2.54638	0.73031	-1.89369
H	-1.53706	-0.72099	-1.81438
H	-0.73641	2.30196	-1.48405
H	-0.01358	0.78297	-1.99041
H	1.62283	2.47345	-1.12736
H	0.86590	2.39704	0.45762
H	3.37515	0.47181	-1.53602
H	1.76066	-0.05763	-1.98990
H	4.92348	-1.29546	-1.30893
H	3.63171	-5.23966	-0.23628
H	-4.80499	-3.91507	2.42694
H	-3.54143	-2.82079	3.03243

H	-3.11288	-4.10225	1.91380
H	-7.79016	-1.12962	-0.57429
H	-7.32165	-2.34358	-1.76402
H	-7.05385	-0.62979	-2.10345
H	-1.08856	1.76808	1.49475
H	-2.56835	0.84324	1.28605
H	3.31501	0.01352	1.33029
H	2.28272	1.31690	1.90335
H	5.92147	-4.74799	-1.51333
H	6.31291	-4.07714	0.06985
H	6.52265	-3.08917	-1.38058
H	1.35786	-5.72357	0.20166
H	0.18787	-4.69557	-0.65646
H	0.50652	-4.41476	1.04526
C	-2.55917	2.74367	0.27197
C	-1.90314	3.98390	0.29623
C	-3.89004	2.70323	-0.16976
C	-2.54503	5.14670	-0.13092
H	-0.88277	4.04463	0.66831
C	-4.53466	3.86458	-0.59788
H	-4.42579	1.75882	-0.16633
C	-3.86367	5.08848	-0.58430
H	-2.01849	6.09681	-0.10085
H	-5.56709	3.81315	-0.93317
H	-4.36810	5.99258	-0.91431
C	3.94058	1.97383	0.69974
C	3.72180	3.35112	0.85273
C	5.19753	1.55245	0.24197
C	4.71434	4.27885	0.53490
H	2.76917	3.70284	1.24294
C	6.19497	2.47564	-0.07669
H	5.39962	0.48830	0.15231
C	5.95411	3.84306	0.06347
H	4.52369	5.34097	0.66589
H	7.16265	2.12557	-0.42661
H	6.73015	4.56352	-0.18075

$A_{14}C_2B_1$

Al	0.02680	0.38152	0.65145
C	0.32980	0.58866	2.61160
O	-1.21740	-0.76969	-0.01793
C	-2.53234	-0.76361	-0.11777
C	-3.18986	-1.97093	-0.48867
C	-4.57430	-1.98133	-0.59209
C	-5.37996	-0.84527	-0.35256
C	-4.73369	0.32735	-0.00359
C	-3.32566	0.39405	0.11740
C	-2.72450	1.65979	0.44639
N	-1.45557	1.86665	0.58216
C	-0.97339	3.21748	0.86111
C	-0.22052	3.79106	-0.36240
C	0.30407	2.70395	-1.31052
N	1.02009	1.61471	-0.62582
C	2.29661	1.49681	-0.88043
C	3.16280	0.44088	-0.46407
C	4.55115	0.54587	-0.74206
C	5.42615	-0.48022	-0.45122
C	4.88025	-1.66254	0.11292
C	3.53402	-1.82943	0.38646
C	2.63219	-0.75254	0.11412
O	1.36257	-0.89816	0.36211
H	-0.43079	1.15689	3.16493
H	1.30316	1.04902	2.84366
H	0.36617	-0.41311	3.06582
H	-5.06267	-2.91456	-0.87046

H	-5.31224	1.23076	0.18401
H	-3.41289	2.50323	0.57938
H	-0.29017	3.14538	1.71344
H	-1.79848	3.87963	1.15317
H	-0.54006	2.25061	-1.84120
H	0.95285	3.16148	-2.06710
H	2.75803	2.26681	-1.50782
H	4.92121	1.46541	-1.19328
H	5.55783	-2.48527	0.33956
H	-0.88769	4.43556	-0.94779
H	0.60821	4.41824	-0.01389
C	6.90806	-0.37717	-0.72134
H	7.25473	-1.17477	-1.39173
H	7.16225	0.58088	-1.18729
H	7.49546	-0.46142	0.20284
C	2.98388	-3.10115	0.97443
H	2.49426	-2.91539	1.93837
H	2.22015	-3.54257	0.32235
H	3.77684	-3.84065	1.12723
C	-2.36190	-3.20129	-0.74978
H	-1.63935	-3.03206	-1.55747
H	-1.77395	-3.48082	0.13285
H	-2.99559	-4.05051	-1.02592
C	-6.88260	-0.92761	-0.47849
H	-7.35325	0.03986	-0.27355
H	-7.18879	-1.23850	-1.48610
H	-7.30832	-1.65759	0.22265

$A_{14}C_3B_1$

Al	0.04344	-0.01093	0.68569
C	0.34149	0.08371	2.65544
O	-1.19515	-1.12979	-0.04839
C	-2.51191	-1.12887	-0.12424
C	-3.16749	-2.31859	-0.55124
C	-4.55355	-2.33502	-0.62905
C	-5.36306	-1.22170	-0.30934
C	-4.71908	-0.06529	0.09383
C	-3.30949	0.00700	0.19087
C	-2.71120	1.25819	0.57654
N	-1.44035	1.46661	0.69435
C	-0.95338	2.79913	1.03236
C	-0.26163	3.48325	-0.18504
C	0.29679	2.40978	-1.15347
N	1.02787	1.30586	-0.51193
C	2.30455	1.20942	-0.77670
C	3.17861	0.13825	-0.41878
C	4.56625	0.26889	-0.68977
C	5.44844	-0.76644	-0.45963
C	4.91076	-1.98405	0.03267
C	3.56558	-2.17635	0.29441
C	2.65637	-1.09141	0.08649
O	1.38741	-1.26155	0.32308
C	-1.28623	4.31872	-0.97387
C	0.86565	4.39138	0.33250
H	-0.41925	0.62160	3.23816
H	1.31508	0.52834	2.91545
H	0.37494	-0.94217	3.05257
H	-5.04011	-3.25503	-0.95125
H	-5.30072	0.82092	0.34358
H	-3.40160	2.08681	0.77406
H	-0.22622	2.67866	1.84261
H	-1.76517	3.43647	1.40865
H	-0.53840	1.96146	-1.70265
H	0.94114	2.90222	-1.89293
H	2.75833	2.01220	-1.36689

H	4.93000	1.21605	-1.08556
H	5.59397	-2.81411	0.21033
H	-2.15320	3.71550	-1.27032
H	-0.84198	4.73118	-1.88831
H	-1.64937	5.16053	-0.37268
H	1.65995	3.80954	0.81465
H	0.48325	5.10508	1.07279
H	1.31391	4.97062	-0.48406
C	-6.86724	-1.30980	-0.41101
H	-7.34124	-0.36069	-0.13894
H	-7.19093	-1.56210	-1.42945
H	-7.27332	-2.08445	0.25285
C	-2.33576	-3.52519	-0.89747
H	-1.63184	-3.30538	-1.70945
H	-1.72702	-3.84791	-0.04419
H	-2.96866	-4.36338	-1.20704
C	3.02385	-3.48491	0.80409
H	2.53188	-3.36086	1.77668
H	2.26395	-3.89157	0.12546
H	3.82185	-4.22670	0.91333
C	6.92972	-0.63720	-0.72193
H	7.17767	0.34877	-1.12930
H	7.51722	-0.77280	0.19604
H	7.28221	-1.39071	-1.43865

$A_{14}C_5B_1$

O	-1.06053	-1.36495	0.34006
O	1.47863	-1.14996	0.95659
N	-1.45476	1.29930	0.35725
N	1.08944	1.35248	-0.05060
C	-0.91594	2.65449	0.42458
C	-1.79271	3.83139	-0.00875
C	-1.01815	5.14752	0.18546
C	0.33851	5.13116	-0.53620
C	1.18907	3.90664	-0.15426
C	0.39215	2.61530	-0.39431
C	-2.65079	1.02660	-0.04698
C	-3.16298	-0.30971	-0.20137
C	-4.51140	-0.47511	-0.59702
C	-5.06190	-1.72701	-0.80479
C	-4.21612	-2.84437	-0.61845
C	-2.88511	-2.73922	-0.24021
C	-2.32679	-1.44760	-0.01346
C	2.25543	1.13606	-0.59390
C	3.06855	-0.02756	-0.43625
C	4.33097	-0.06620	-1.08456
C	5.16959	-1.15612	-0.97284
C	4.71954	-2.24592	-0.18416
C	3.49732	-2.26717	0.46552
C	2.63011	-1.13657	0.34733
H	-0.63151	2.80986	1.47646
H	-2.07893	3.71812	-1.06400
H	-2.71950	3.85385	0.57742
H	-1.62359	5.98960	-0.17064
H	-0.85614	5.31086	1.26004
H	0.89412	6.04962	-0.31189
H	0.17086	5.12507	-1.62287
H	2.11685	3.90873	-0.73726
H	1.47670	3.95466	0.90491
H	0.11194	2.56387	-1.45776
H	-3.33581	1.83952	-0.30435
H	-5.12184	0.41603	-0.73653
H	-4.62844	-3.83988	-0.77977
H	2.67265	1.90909	-1.24619
H	4.63407	0.79480	-1.67857

H	5.37040	-3.11421	-0.08559
Al	0.00049	-0.02437	1.00933
C	-0.13194	0.34253	2.95623
H	0.00148	-0.59150	3.52211
H	0.65325	1.02448	3.31748
H	-1.09359	0.76908	3.27688
C	6.51631	-1.21053	-1.65333
H	7.33210	-1.33378	-0.92861
H	6.58314	-2.05405	-2.35330
H	6.71359	-0.29434	-2.22015
C	3.04635	-3.44076	1.29301
H	2.85563	-3.14562	2.33228
H	2.10439	-3.85552	0.91404
H	3.79924	-4.23584	1.29347
C	-2.01162	-3.95156	-0.05463
H	-1.13783	-3.91854	-0.71690
H	-1.61897	-4.00735	0.96806
H	-2.56698	-4.87226	-0.26174
C	-6.50310	-1.91355	-1.21560
H	-7.01029	-0.95059	-1.33854
H	-6.58714	-2.45631	-2.16639
H	-7.06574	-2.49039	-0.46953

$A_{14}C_5B_2$

Al	0.04784	-0.14277	0.97404
C	0.46443	0.25850	2.86581
O	-1.45150	-1.14985	1.15018
C	-2.55129	-1.32043	0.42137
C	-3.50764	-2.28900	0.81713
C	-4.68143	-2.41685	0.07911
C	-4.96733	-1.61728	-1.04013
C	-4.01714	-0.66689	-1.40946
C	-2.81539	-0.51657	-0.70513
C	-1.77078	0.44609	-1.20120
N	-1.07697	1.28784	-0.13635
C	-0.27155	2.34389	-0.87110
C	1.04630	2.74417	-0.15764
N	1.79146	1.51888	0.24116
C	2.46749	0.94235	-0.96438
C	3.09663	-0.40092	-0.72347
C	4.45560	-0.63468	-0.96247
C	5.01564	-1.90239	-0.79978
C	4.16683	-2.93918	-0.38240
C	2.80907	-2.74603	-0.12721
C	2.26746	-1.45188	-0.29724
O	0.95538	-1.24702	-0.08769
C	-2.08948	1.84616	0.80505
C	2.82273	1.77317	1.26491
H	-0.17915	-0.36844	3.49440
H	0.28439	1.29983	3.16867
H	1.50212	0.02925	3.13850
H	-5.40939	-3.16532	0.39077
H	-4.20575	-0.03020	-2.27367
H	-2.22707	1.12423	-1.93345
H	-0.96013	-0.09294	-1.70853
H	0.02343	1.82921	-1.78809
H	1.63740	3.26736	-0.93359
H	3.22374	1.65694	-1.33103
H	1.71201	0.84074	-1.74942
H	5.08418	0.19235	-1.29088
H	4.58121	-3.93786	-0.24891
H	-2.85742	2.41823	0.27471
H	-1.59624	2.48395	1.53642
H	-2.56938	1.02393	1.33114
H	3.55282	2.52971	0.93138

H	3.35903	0.84201	1.45138
H	2.36814	2.09687	2.19912
C	-1.04773	3.60468	-1.32885
H	-0.49226	4.00192	-2.18948
H	-2.03131	3.32198	-1.72166
C	-1.18280	4.73278	-0.29055
H	-1.56652	5.62939	-0.79298
H	-1.92316	4.47402	0.47292
C	0.83804	3.78908	0.95456
H	0.25627	3.37574	1.78625
H	1.80640	4.08085	1.37017
C	0.15806	5.04524	0.38409
H	0.83552	5.50757	-0.34873
H	0.01716	5.78324	1.18266
C	6.48228	-2.15838	-1.06262
H	6.99555	-2.53710	-0.16915
H	6.62890	-2.90477	-1.85419
H	6.99627	-1.24287	-1.37452
C	1.91261	-3.87275	0.31733
H	1.46816	-3.66699	1.29879
H	1.07341	-4.00797	-0.37494
H	2.46655	-4.81503	0.38089
C	-3.22456	-3.14437	2.02449
H	-2.28769	-3.70123	1.90437
H	-3.10244	-2.53192	2.92649
H	-4.03435	-3.86022	2.19994
C	-6.25633	-1.79008	-1.81058
H	-6.31599	-2.77665	-2.28937
H	-7.13562	-1.69909	-1.15974
H	-6.35153	-1.03721	-2.60063

$A_{14}C_{11}B_1$

Al	-0.26515	-0.95388	0.96470
N	1.41312	0.10412	0.36208
N	-1.06373	0.60124	-0.07028
O	0.55782	-2.45416	0.30244
O	-1.91301	-1.80993	0.85641
C	2.14932	-4.13708	-0.13987
C	3.46997	-4.47848	-0.39484
C	4.51610	-3.53100	-0.47990
C	4.18160	-2.20074	-0.30263
C	2.85216	-1.79659	-0.03232
C	1.81397	-2.76669	0.06041
C	2.57574	-0.39001	0.08339
C	1.15019	1.54991	0.43213
C	-0.18707	1.77274	-0.33992
C	-2.24175	0.61385	-0.63543
C	-3.23934	-0.40157	-0.55012
C	-4.46772	-0.20530	-1.23518
C	-5.47989	-1.14112	-1.18641
C	-5.24697	-2.31410	-0.42252
C	-4.07035	-2.56065	0.26370
C	-3.02194	-1.59018	0.20932
C	-0.11286	-0.65313	2.92234
H	3.71125	-5.53152	-0.53634
H	4.95117	-1.43319	-0.36968
H	3.41737	0.28286	-0.09788
H	0.94788	1.76447	1.48934
H	0.04706	1.77224	-1.41125
H	-2.51146	1.48785	-1.23544
H	-4.60024	0.71287	-1.80540
H	-6.03773	-3.06214	-0.37371
H	-0.34625	-1.58746	3.45511
H	0.88307	-0.33872	3.26671
H	-0.83010	0.08996	3.30371

C	-0.82860	3.10623	0.01408
C	-0.84460	4.15280	-0.91519
C	-1.39743	3.31703	1.27949
C	-1.40969	5.38817	-0.58928
H	-0.40663	4.00295	-1.89903
C	-1.96370	4.54855	1.60528
H	-1.40781	2.50986	2.00741
C	-1.97022	5.58901	0.67197
H	-1.41294	6.18974	-1.32323
H	-2.40289	4.69548	2.58850
H	-2.41317	6.54807	0.92700
C	2.25662	2.48150	-0.03174
C	2.86950	3.34164	0.88787
C	2.68845	2.51133	-1.36770
C	3.87948	4.21836	0.48730
H	2.54663	3.32893	1.92595
C	3.70048	3.38312	-1.77002
H	2.24017	1.84365	-2.09950
C	4.29728	4.24166	-0.84362
H	4.33755	4.88185	1.21588
H	4.02284	3.39140	-2.80787
H	5.08372	4.92238	-1.15830
C	5.93297	-3.97353	-0.75775
H	6.01004	-4.51331	-1.71087
H	6.61521	-3.11813	-0.80574
H	6.30615	-4.65063	0.02207
C	1.05975	-5.17324	-0.05840
H	0.57530	-5.16636	0.92573
H	0.26771	-4.97780	-0.79164
H	1.45734	-6.17729	-0.23966
C	-3.85400	-3.81592	1.06551
H	-2.98325	-4.37503	0.70190
H	-3.65296	-3.58568	2.11910
H	-4.73032	-4.47067	1.01732
C	-6.79259	-0.94763	-1.90673
H	-7.64114	-0.94676	-1.20968
H	-6.81140	0.00227	-2.45150
H	-6.97862	-1.75011	-2.63289

$A_{15}C_3B_1$

Al	-0.52712	1.55467	0.61580
C	0.16758	1.98827	2.43522
O	-1.49832	0.03936	0.28818
C	-2.73845	-0.35675	0.52942
C	-3.10256	-1.71355	0.26382
C	-4.40996	-2.10581	0.56894
C	-5.37549	-1.23783	1.10389
C	-5.02926	0.07971	1.32607
C	-3.72766	0.54177	1.04037
C	-3.46919	1.94802	1.21312
N	-2.34577	2.53162	0.96237
C	-2.25142	3.98371	1.05454
C	-2.19667	4.63210	-0.36069
C	-1.55595	3.63953	-1.36658
N	-0.36574	2.93853	-0.86198
C	0.78470	3.27151	-1.37619
C	2.04803	2.64784	-1.12993
C	3.20291	3.27911	-1.64710
C	4.44150	2.69441	-1.50738
C	4.52850	1.44063	-0.87082
C	3.42985	0.74667	-0.36218
C	2.13992	1.37067	-0.47845
O	1.06100	0.77372	-0.03926
C	-3.61855	4.94027	-0.86246
C	-1.37996	5.93181	-0.27714

H	-0.52590	2.51458	3.10525
H	1.09463	2.58076	2.40201
H	0.43978	1.05369	2.94808
H	-4.70894	-3.13266	0.38897
H	-5.75870	0.78611	1.71807
H	-4.31369	2.55513	1.56039
H	-1.32965	4.21657	1.59804
H	-3.08956	4.40302	1.62729
H	-2.29206	2.87227	-1.62755
H	-1.30763	4.17752	-2.29002
H	0.79913	4.09986	-2.09211
H	3.09372	4.23775	-2.15105
H	5.51564	0.99948	-0.78494
H	-4.25544	4.04756	-0.84044
H	-3.60157	5.30814	-1.89585
H	-4.09328	5.71092	-0.24387
H	-0.33422	5.73448	-0.01351
H	-1.79095	6.60211	0.48791
H	-1.39602	6.47130	-1.23197
H	-6.37572	-1.59777	1.32599
H	5.33582	3.17840	-1.88825
Si	-1.79905	-2.91918	-0.44498
C	-1.05030	-2.15875	-2.01183
H	-1.81027	-2.01311	-2.79040
H	-0.61908	-1.18057	-1.78111
H	-0.25868	-2.78715	-2.43615
C	-0.48358	-3.17824	0.89412
H	-0.00591	-2.22497	1.13937
H	-0.93071	-3.56887	1.81674
H	0.30139	-3.87700	0.58234
C	-2.53531	-4.65576	-0.92052
C	-1.40140	-5.47653	-1.58178

H	-1.76324	-6.48545	-1.83145
H	-1.04822	-5.02134	-2.51414
H	-0.53470	-5.60065	-0.92112
C	-3.01513	-5.44885	0.31482
H	-3.82879	-4.95454	0.85704
H	-3.38575	-6.43999	0.01145
H	-2.20141	-5.61490	1.03110
C	-3.68330	-4.54317	-1.94681
H	-4.02248	-5.54466	-2.25314
H	-4.55805	-4.01285	-1.55526
H	-3.36520	-4.01932	-2.85674
Si	3.59572	-0.99481	0.40827
C	2.73946	-1.04314	2.09671
H	3.27222	-0.44156	2.84385
H	1.72807	-0.63966	2.01076
H	2.66515	-2.06661	2.48315
C	2.76509	-2.20538	-0.79084
H	2.78648	-3.23866	-0.42391
H	1.71878	-1.92104	-0.93654
H	3.24961	-2.18980	-1.77497
C	5.44014	-1.55695	0.66796
C	6.23549	-0.56755	1.54677
H	7.24351	-0.96091	1.74918
H	6.36127	0.41506	1.07951
H	5.75216	-0.40632	2.51827
C	6.18475	-1.78504	-0.66551
H	6.27484	-0.87672	-1.27160
H	7.20661	-2.14702	-0.47447
H	5.68609	-2.54071	-1.28441
C	5.40430	-2.91380	1.41300
H	4.95937	-2.82772	2.41087
H	4.84421	-3.67920	0.86168

H	6.42718	-3.29718	1.54677

$A_{16}C_{1}B_{2}$

Al	0.06655	0.60307	0.56495
C	0.54310	0.31555	2.44560
O	-1.28821	-0.49845	0.13508
C	-2.57591	-0.28667	-0.05542
C	-3.54243	-1.26617	0.25526
C	-4.90096	-1.05123	0.04736
C	-5.31937	0.17133	-0.47398
C	-4.39972	1.16863	-0.78479
C	-3.03730	0.94098	-0.58510
C	-2.01120	1.96573	-0.98438
N	-1.01699	2.33136	0.10230
C	-0.19510	3.45655	-0.45697
C	0.96484	3.90836	0.42493
N	1.93594	2.83860	0.65937
C	2.83119	2.64109	-0.50214
C	3.44382	1.26252	-0.50863
C	4.82256	1.06417	-0.43063
C	5.33934	-0.22965	-0.40707
C	4.49997	-1.33892	-0.43038
C	3.12479	-1.13070	-0.50709
C	2.56595	0.15795	-0.57816
O	1.25129	0.34374	-0.72874
C	-1.74675	2.78460	1.32017
C	2.71206	3.07056	1.87976
H	0.15610	-0.66378	2.75770
H	0.13444	1.05304	3.14911
H	1.62905	0.29685	2.59992
H	-5.61918	-1.82343	0.29585
H	-4.73842	2.11693	-1.19034
H	-2.51087	2.88672	-1.30873
H	-1.41163	1.59622	-1.82489
H	-0.86253	4.31096	-0.63723
H	0.17355	3.11966	-1.42895
H	1.42153	4.79394	-0.05357
H	0.58905	4.24820	1.39432
H	3.62070	3.41164	-0.51985
H	2.23968	2.76720	-1.41373
H	5.48962	1.91935	-0.38454
H	4.89985	-2.34459	-0.38216
H	-2.37246	3.65560	1.08675
H	-1.03599	3.04676	2.10338
H	-2.38437	1.98006	1.68932
H	3.28881	4.01239	1.84057
H	3.41470	2.24711	2.02982
H	2.04413	3.10729	2.74519
Br	-7.19012	0.48411	-0.75601
Br	-2.95321	-2.92161	0.99485
Br	1.93758	-2.62041	-0.45570
Br	7.23770	-0.48947	-0.31128

$A_{16}C_{1}B_{3}$

Al	-0.02387	-0.20490	0.61580
C	0.67014	-0.09546	2.44667
O	-1.54204	-1.15385	0.74607
C	-2.75787	-1.02500	0.25559
C	-3.80630	-1.86841	0.68557
C	-5.09794	-1.75765	0.18477
C	-5.36756	-0.77560	-0.76605
C	-4.36799	0.08640	-1.20474
C	-3.06958	-0.03051	-0.70025
C	-1.96941	0.83412	-1.26504
N	-0.97932	1.42198	-0.27518
C	-0.06528	2.28890	-1.09528

C	1.20186	2.76886	-0.39129
N	2.09944	1.67130	-0.02588
C	2.80873	1.09851	-1.19164
C	3.21762	-0.33636	-0.95616
C	4.55329	-0.73717	-1.01379
C	4.89598	-2.06474	-0.76577
C	3.92756	-3.00606	-0.43261
C	2.59630	-2.59929	-0.38344
C	2.20453	-1.27944	-0.67151
O	0.91580	-0.92723	-0.70445
C	-1.66153	2.20167	0.83969
C	3.00985	2.00611	1.09742
H	0.19087	-0.89286	3.03049
H	0.46167	0.84495	2.97399
H	1.75273	-0.25950	2.50314
H	-5.87787	-2.42373	0.53374
H	-4.59230	0.84646	-1.94652
H	-2.40860	1.65532	-1.84204
H	-1.35722	0.24062	-1.95485
H	-0.63081	3.15891	-1.44628
H	0.19811	1.70265	-1.97764
H	1.67831	3.51112	-1.05430
H	0.93927	3.30373	0.52575
H	3.68561	1.70351	-1.46512
H	2.12896	1.13066	-2.04739
H	5.32351	-0.01156	-1.25497
H	4.19669	-4.03154	-0.21008
H	-0.87372	2.45966	1.55065
H	-2.32430	1.49305	1.34109
H	3.54283	1.08680	1.35668
H	2.37526	2.25678	1.95329
C	-2.43365	3.45068	0.45509
C	-1.81236	4.70978	0.46419
C	-3.80115	3.38721	0.14842
C	-2.52654	5.86594	0.14792
H	-0.76342	4.79161	0.73806
C	-4.51875	4.54181	-0.16777
H	-4.31127	2.42931	0.17197
C	-3.88230	5.78402	-0.17452
H	-2.02601	6.83033	0.16376
H	-5.57833	4.46989	-0.39804
H	-4.44150	6.68364	-0.41691
C	4.01707	3.12513	0.86475
C	3.66893	4.46892	1.07096
C	5.32698	2.83778	0.45271
C	4.59023	5.49365	0.85049
H	2.67054	4.71618	1.42481
C	6.25333	3.85887	0.23107
H	5.62929	1.80115	0.32413
C	5.88536	5.19127	0.42440
H	4.30046	6.52728	1.02190
H	7.26400	3.61208	-0.08375
H	6.60550	5.98770	0.25659
Br	-7.14314	-0.60687	-1.47033
Br	-3.42191	-3.19800	1.99736
Br	1.24595	-3.83781	0.14019
Br	6.73578	-2.60042	-0.85700

$A_{16}C_{2}B_{1}$

Al	-0.00372	1.07656	0.75358
C	0.32347	1.20539	2.70702
O	-1.27669	-0.02074	0.04992
C	-2.58046	-0.00330	-0.01320
C	-3.29104	-1.17613	-0.38287
C	-4.67331	-1.20736	-0.46859

C	-5.41026	-0.04990	-0.18618
C	-4.77019	1.11885	0.17550
C	-3.36434	1.15634	0.26493
C	-2.73824	2.40947	0.62118
N	-1.46694	2.58969	0.73979
C	-0.95746	3.92295	1.06095
C	-0.20128	4.52154	-0.14786
C	0.29784	3.46115	-1.13820
N	1.00368	2.33645	-0.49808
C	2.27011	2.20547	-0.77171
C	3.12033	1.11220	-0.39621
C	4.50297	1.20874	-0.67374
C	5.33287	0.14165	-0.41106
C	4.81462	-1.05866	0.10722
C	3.46200	-1.16884	0.36275
C	2.55590	-0.08952	0.13964
O	1.29460	-0.21408	0.37954
H	-0.42158	1.76427	3.28932
H	1.30694	1.63864	2.94666
H	0.34543	0.18471	3.11637
H	-5.17870	-2.12391	-0.74966
H	-5.34121	2.01645	0.39209
H	-3.41432	3.25436	0.79367
H	-0.27158	3.80619	1.90578
H	-1.76863	4.58879	1.37987
H	-0.55588	3.03411	-1.67504
H	0.94688	3.93359	-1.88432
H	2.74327	2.98368	-1.37840
H	4.89957	2.12850	-1.09252
H	5.47420	-1.89583	0.30451
H	-0.86006	5.19648	-0.70704
H	0.63996	5.12159	0.21675
Br	7.21144	0.26908	-0.76163
Br	2.75764	-2.79669	1.05431
Br	-2.29294	-2.75422	-0.75485
Br	-7.32064	-0.10364	-0.30582

$A_{16}C_3B_1$

Al	0.00685	0.77043	0.76476
C	0.33460	0.84076	2.72125
O	-1.27104	-0.30692	0.03676
C	-2.57589	-0.28649	-0.00433
C	-3.29426	-1.44836	-0.39307
C	-4.67783	-1.47649	-0.45664
C	-5.40878	-0.32671	-0.13094
C	-4.76143	0.83122	0.25200
C	-3.35427	0.86583	0.31805
C	-2.72085	2.10948	0.69437
N	-1.44648	2.28433	0.78920
C	-0.92058	3.60383	1.12651
C	-0.23192	4.27455	-0.09935
C	0.29737	3.19409	-1.07602
N	1.01300	2.07315	-0.44301
C	2.28099	1.95349	-0.71547
C	3.13379	0.85495	-0.36001
C	4.51887	0.96718	-0.61852
C	5.35173	-0.10329	-0.37973
C	4.83412	-1.32214	0.09354
C	3.47903	-1.44756	0.32860
C	2.56953	-0.36649	0.12964
O	1.30543	-0.50602	0.34821
C	-1.25191	5.12616	-0.87696
C	0.91578	5.16343	0.40659
H	-0.39959	1.39890	3.31791
H	1.32627	1.24784	2.97246

H	0.33585	-0.19063	3.10364
H	-5.18858	-2.38501	-0.75357
H	-5.32776	1.72288	0.50264
H	-3.39119	2.94948	0.90663
H	-0.18509	3.45805	1.92455
H	-1.71053	4.25826	1.51754
H	-0.54877	2.75772	-1.61765
H	0.94461	3.67464	-1.81969
H	2.75233	2.74417	-1.30668
H	4.91524	1.90184	-1.00313
H	5.49591	-2.16175	0.27220
H	-2.13247	4.53768	-1.16287
H	-0.81130	5.53013	-1.79660
H	-1.59261	5.97423	-0.27191
H	1.70541	4.56981	0.88210
H	0.55201	5.88347	1.14969
H	1.36445	5.73475	-0.41493
Br	-7.32095	-0.37513	-0.22207
Br	-2.30481	-3.01712	-0.82312
Br	2.77662	-3.10073	0.95948
Br	7.23363	0.04540	-0.70299

$A_{16}C_7B_1$

Al	0.02336	0.51534	0.66254
C	0.34971	0.62808	2.60903
O	-1.21447	-0.67958	0.03536
C	-2.50814	-0.74393	-0.01947
C	-3.16396	-1.97491	-0.29527
C	-4.54196	-2.06514	-0.37322
C	-5.32812	-0.91599	-0.18675
C	-4.74128	0.30365	0.07458
C	-3.33453	0.40006	0.16807
C	-2.75304	1.68866	0.40458
N	-1.48420	1.96359	0.55452
N	1.19095	1.88684	-0.36909
C	2.49592	1.68612	-0.39534
C	3.21711	0.47894	-0.15454
C	4.63213	0.51824	-0.22546
C	5.35545	-0.64351	-0.08757
C	4.70665	-1.87771	0.11499
C	3.32942	-1.92697	0.18562
C	2.52921	-0.75207	0.07109
O	1.24639	-0.80053	0.15127
H	-0.09588	1.49900	3.10850
H	1.42156	0.63514	2.85502
H	-0.06446	-0.26455	3.10229
H	-5.01222	-3.01987	-0.57757
H	-5.34852	1.19276	0.21307
H	-3.46030	2.51971	0.43290
H	3.12428	2.54361	-0.63619
H	5.13246	1.46650	-0.39555
H	5.28893	-2.78587	0.21974
C	-1.18657	3.34473	0.75350
C	-0.18400	3.93849	-0.07150
C	-1.95289	4.13152	1.59821
C	0.80436	3.19815	-0.79884
C	-0.19998	5.36709	-0.21226
C	-1.84092	5.53754	1.57262
H	-2.66826	3.65842	2.26389
C	1.48871	3.82437	-1.83207
C	0.59004	5.97918	-1.22176
C	-1.02507	6.14330	0.64580
H	-2.44487	6.13352	2.25041
C	1.36219	5.21077	-2.06032
H	2.15776	3.24391	-2.45911

H	0.54652	7.05862	-1.33682
H	-0.99530	7.22562	0.55553
H	1.91875	5.66773	-2.87325
Br	-7.23337	-1.06480	-0.30466
Br	-2.09233	-3.52654	-0.54648
Br	2.44084	-3.58486	0.46866
Br	7.26745	-0.60830	-0.18063

$A_{16}C_{11}B_{1}$

Al	0.02188	0.07112	1.10974
N	1.48523	1.39138	0.45285
N	-1.05331	1.46656	0.09177
O	1.04733	-1.26057	0.37836
O	-1.46359	-1.04128	1.04260
C	2.89292	-2.65226	-0.10812
C	4.23487	-2.82039	-0.40435
C	5.06602	-1.69676	-0.51111
C	4.55735	-0.42676	-0.32765
C	3.19360	-0.24943	-0.01698
C	2.31765	-1.37055	0.10568
C	2.69723	1.10069	0.12154
C	0.98455	2.77392	0.54839
C	-0.38906	2.77276	-0.18928
C	-2.22501	1.28310	-0.44530
C	-3.03243	0.10289	-0.34587
C	-4.27899	0.08791	-1.01364
C	-5.08444	-1.02646	-0.94176
C	-4.68048	-2.15641	-0.20848
C	-3.46489	-2.15186	0.44717
C	-2.58816	-1.02778	0.40995
C	0.19072	0.37771	3.05620
H	4.63762	-3.81577	-0.55088
H	5.19895	0.44404	-0.41999
H	3.41116	1.90039	-0.08806
H	0.77849	2.93527	1.61382
H	-0.18447	2.80747	-1.26538
H	-2.65678	2.09769	-1.03293
H	-4.59114	0.96101	-1.57814
H	-5.32059	-3.02959	-0.15755
H	0.15926	-0.59415	3.57083
H	1.12076	0.87054	3.37296
H	-0.64094	0.96208	3.47765
C	-1.24123	3.97536	0.18456
C	-1.48456	4.98642	-0.75227
C	-1.78578	4.09845	1.47213
C	-2.25098	6.10360	-0.41160
H	-1.06760	4.90350	-1.75307
C	-2.55271	5.21171	1.81197
H	-1.61902	3.31530	2.20749
C	-2.78633	6.21863	0.87108
H	-2.42952	6.88008	-1.15055
H	-2.97017	5.29255	2.81200
H	-3.38502	7.08546	1.13748
C	1.90963	3.87952	0.07300
C	2.41926	4.79941	0.99797
C	2.27622	4.00967	-1.27634
C	3.26534	5.83166	0.58902
H	2.14467	4.71015	2.04621
C	3.12506	5.03796	-1.68660
H	1.90556	3.30137	-2.01340
C	3.61984	5.95355	-0.75488
H	3.64555	6.53896	1.32100
H	3.39924	5.12384	-2.73463
H	4.27851	6.75597	-1.07577
Br	-6.77397	-1.05771	-1.84307

Br	-2.91619	-3.68285	1.43584
Br	1.76276	-4.17796	0.03846
Br	6.92101	-1.94202	-0.92017

Reaction pathways for ROP of LA by various Al complexes

L-LA

O	-0.25870900	1.35202500	-0.11136100
O	0.25869800	-1.35203600	-0.11128900
O	-1.91782400	-1.72047000	-0.38241900
O	1.91779400	1.72044200	-0.38267600
C	1.03440800	0.96628000	-0.05709600
C	1.26440400	-0.46001700	0.44127400
C	-1.03441300	-0.96628100	-0.05696900
C	-1.26436800	0.46004200	0.44134500
C	-2.62084000	1.00636100	0.04578900
C	2.62084300	-1.00635700	0.04563100
H	1.15507700	-0.45667100	1.53589500
H	-1.15494100	0.45676000	1.53595600
H	-2.72625800	1.01120900	-1.04266700
H	-2.73266800	2.02699300	0.42181500
H	-3.40877000	0.37868000	0.46874900
H	2.73270100	-2.02697100	0.42169600
H	3.40880800	-0.37865700	0.46849600
H	2.72617100	-1.01125500	-1.04283300

D-LA

O	0.25867800	1.35211900	-0.11029000
O	-0.25864500	-1.35214700	-0.11032300
O	1.91787900	-1.72058500	-0.38112700
O	-1.91790400	1.72114300	-0.37982900
C	-1.03440100	0.96629300	-0.05618800
C	-1.26453000	-0.46020500	0.44168300
C	1.03446500	-0.96652800	-0.05540700
C	1.26451900	0.46019200	0.44190100
C	-2.62082800	-1.00628400	0.04508100
H	-3.40896700	-0.37853300	0.46753400
H	-2.72543000	-1.01094500	-1.04345500
H	-2.73306600	-2.02697100	0.42083000
C	2.62082400	1.00619200	0.04521000
H	2.72541600	1.01069600	-1.04332900
H	2.73310200	2.02693100	0.42080500
H	3.40894600	0.37847600	0.46774200
H	1.15590100	0.45717200	1.53659400
H	-1.15587800	-0.45699700	1.53637700

(A6C1B3)Al-(L-LA)$_2$

Al	0.24686000	0.24224700	-0.16876000
O	1.42140100	-1.14696700	-0.42858700
C	2.56728200	-1.40723100	0.15541500
C	3.21650300	-2.64769200	-0.07059500
C	4.44354900	-2.97851700	0.49048600
C	5.07346200	-2.04201700	1.30607200
C	4.48885600	-0.80371200	1.54841700
C	3.25094500	-0.47882600	0.98815800
C	2.63762200	0.85372500	1.36123700
N	1.83630100	1.52026000	0.27772000
C	1.26750900	2.79443700	0.81027300
C	-0.03438500	3.15903600	0.10212500
N	-1.04381500	2.07957600	0.26297900
C	-1.60367300	2.09780400	1.65391300
C	-2.23052100	0.78569500	2.03526800
C	-3.55902200	0.66958700	2.43804200
C	-4.05940100	-0.58296900	2.79067400
C	-3.25538400	-1.71994000	2.74614200
C	-1.92777000	-1.58843900	2.34160900
C	-1.37748400	-0.34048800	1.98389700
O	-0.12281700	-0.19848300	1.58197300
C	2.66444000	1.76064400	-0.97270400
C	-2.14651600	2.18794000	-0.75307600
H	4.89818000	-3.94157900	0.28969900
H	4.99877400	-0.08140800	2.17878000
H	3.42549300	1.53941800	1.69375900
H	1.94379300	0.70906000	2.19364200

H	1.98910100	3.61097300	0.71416900
H	1.09499300	2.65527400	1.87866900
H	-0.40641100	4.11679600	0.48856200
H	0.14472100	3.27936600	-0.96887500
H	-2.31149800	2.92715400	1.75940400
H	-0.77483000	2.29008800	2.33792700
H	-4.19666600	1.54652100	2.48453300
H	-3.65299300	-2.69294900	3.01116700
H	1.97950900	2.15963800	-1.71934300
H	2.96792900	0.77630700	-1.32912300
H	-2.77844100	1.30844800	-0.60537500
H	-1.65885000	2.09196200	-1.72285200
Cl	2.43076800	-3.82517300	-1.12022500
Cl	6.64101900	-2.42812000	2.02450200
Cl	-0.92326800	-3.02519400	2.22246600
Cl	-5.74698900	-0.73749400	3.29126000
C	-2.99317300	3.44269800	-0.70403600
C	-4.23488000	3.44173100	-0.05277200
C	-2.56698100	4.62542700	-1.32633500
C	-5.02010500	4.59485100	-0.00191200
H	-4.59261300	2.52393500	0.40643300
C	-3.34653800	5.78160200	-1.27557200
H	-1.62350600	4.63661500	-1.86601600
C	-4.57391300	5.77054000	-0.60843300
H	-5.98065800	4.57257300	0.50589900
H	-3.00111600	6.68797500	-1.76594100
H	-5.18329400	6.66954200	-0.57218000
C	3.86359400	2.66835100	-0.80986800
C	3.75717000	4.03950200	-1.08816800
C	5.11033700	2.16274600	-0.41330600
C	4.85568400	4.88896600	-0.94657600
H	2.80734400	4.44201500	-1.43378100
C	6.21121100	3.00830900	-0.27045400
H	5.21869500	1.09901800	-0.22496900
C	6.08555800	4.37481800	-0.53105000
H	4.75315000	5.94789300	-1.16881700
H	7.16920500	2.59723000	0.03673100
H	6.94352300	5.03290900	-0.42247700
O	-2.09256200	-4.77838200	-2.41471100
C	-0.81217600	-4.34870700	-1.90215800
H	-0.32412200	-3.68073800	-2.61424800
H	-0.88840400	-3.86792800	-0.92334000
H	-0.22744000	-5.26318000	-1.79864000
O	0.14520800	0.74473900	-1.92814000
O	-2.21133600	-1.85572300	-2.53234200
O	-1.34142400	-0.90857500	-0.69875500
O	-4.24808200	-4.46732600	-2.77847700
C	-3.20538000	-4.03144200	-2.34672600
C	-3.12157500	-2.64985400	-1.68470900
C	-1.39773800	-1.01576800	-1.93038400
C	-0.49451200	-0.13627800	-2.78595200
C	0.47896500	-0.99930100	-3.60708500
C	-4.45869900	-1.94415200	-1.60661200
H	-2.67499400	-2.73651400	-0.69176200
H	-1.16568600	0.39705100	-3.48373700
H	1.11882300	-1.57445700	-2.93257700
H	1.11095300	-0.33141300	-4.20019200
H	-0.05367300	-1.67419900	-4.28637500
H	-4.33137000	-0.96748300	-1.12932400
H	-5.14969100	-2.53743900	-1.00152700
H	-4.89079500	-1.81757300	-2.60212500

LLA-IM1

Al	0.84953700	-0.05332600	0.35683200
O	2.11871300	-0.72636100	-0.78683600
C	3.41767600	-0.82068800	-0.62566100
C	4.18698800	-1.61048300	-1.51745000
C	5.56850600	-1.72728100	-1.43353500
C	6.23184100	-1.02684700	-0.42925500

C	5.52745600	-0.22915800	0.46605000
C	4.13668100	-0.12090900	0.38306400
C	3.43193600	0.69495800	1.44806400
N	2.19401100	1.41505200	0.99407500
C	1.58118100	2.12832400	2.15543100
C	0.06404000	2.22397600	2.00886900
N	-0.53992900	0.87015700	1.88371000
C	-0.52945400	0.16808700	3.20785100
C	-0.69527700	-1.31947600	3.06591600
C	-1.71398400	-2.03096800	3.69537200
C	-1.78168600	-3.41419100	3.53556300
C	-0.84949100	-4.09730700	2.75764800
C	0.16541800	-3.37281900	2.13421000
C	0.27509100	-1.97358800	2.27208600
O	1.23056800	-1.27084500	1.68393000
C	2.48313300	2.37247000	-0.14997900
C	-1.93707300	0.93314400	1.33409100
H	6.11205600	-2.34553200	-2.13838100
H	6.06339900	0.31463800	1.23837000
H	4.13186300	1.42209800	1.87582500
H	3.10829200	0.02714500	2.25104700
H	2.00982900	3.12820500	2.26656000
H	1.84503100	1.57749900	3.05963700
H	-0.35445800	2.76982200	2.86431200
H	-0.19160300	2.77603000	1.10158200
H	-1.29702400	0.59729500	3.86120200
H	0.43879800	0.35889700	3.67464900
H	-2.44242500	-1.51597300	4.31321200
H	-0.91193600	-5.17202100	2.63092800
H	1.51998200	2.77491200	-0.45876100
H	2.83927900	1.75736400	-0.97625700
H	-2.26285000	-0.10496500	1.22782100
H	-1.84035100	1.36497500	0.34119300
Cl	3.34638800	-2.48308700	-2.79763000
Cl	7.99092900	-1.14312400	-0.30411300
Cl	1.32298300	-4.21927100	1.11918300
Cl	-3.07774200	-4.31946100	4.32536400
C	-2.95602100	1.71318200	2.13757400
C	-3.82520900	1.05892900	3.02183300
C	-3.08228200	3.10188100	1.97733800
C	-4.78039600	1.77217200	3.74947200
H	-3.76176800	-0.02089900	3.12809100
C	-4.03224000	3.81808000	2.70573200
H	-2.46439900	3.61443300	1.24633700
C	-4.88077000	3.15624700	3.59778700
H	-5.44697800	1.24649800	4.42814500
H	-4.12037600	4.89232600	2.56554200
H	-5.62412700	3.71464500	4.16067700
C	3.45712700	3.49079500	0.14874700
C	2.98598400	4.75419800	0.53738300
C	4.84101500	3.30799200	0.01029800
C	3.87162500	5.79829700	0.80991400
H	1.91401100	4.92505200	0.61060500
C	5.72965700	4.34897000	0.28205300
H	5.22136600	2.34681800	-0.32185800
C	5.24770200	5.59550400	0.68841400
H	3.48663400	6.77006000	1.10769200
H	6.79840900	4.18776700	0.16789500
H	5.93973900	6.40697400	0.89740000
O	-1.16344200	-4.17622000	-3.44349300
C	0.10222800	-3.62842600	-3.01516800
H	0.23781100	-2.62486100	-3.42277600
H	0.21105200	-3.61383100	-1.92750600
H	0.85626000	-4.29629900	-3.43291300
O	0.05716700	1.05042700	-0.87146100
O	-1.84807100	-1.64439700	-2.16570800
O	-0.57493300	-1.35920800	-0.33548600
O	-3.34749200	-4.42959400	-3.24417000

C	-2.33546200	-3.89719100	-2.85084700
C	-2.34590100	-2.93362100	-1.65617800
C	-1.02044000	-0.95113200	-1.41569700
C	-0.62202700	0.43365100	-1.90448200
C	0.17982300	0.34658000	-3.21382300
C	-3.73116800	-2.71282900	-1.08422900
H	-1.65636400	-3.28825300	-0.88666100
H	-1.57050000	0.94909400	-2.12174000
H	1.09778200	-0.22357000	-3.04985900
H	0.45322900	1.36349200	-3.51241800
H	-0.41188800	-0.10965000	-4.01503300
H	-3.66386200	-2.05467200	-0.21102800
H	-4.15346900	-3.66886800	-0.76413600
H	-4.38948000	-2.26584900	-1.83246700
O	-3.49230000	2.38814700	-1.02265200
O	-3.51318700	1.59940600	-3.69933600
O	-4.79624500	-0.14599600	-3.21655600
O	-2.23063000	4.14003600	-1.51298200
C	-2.91040600	3.22012900	-1.90343800
C	-3.13267300	2.96269500	-3.38707200
C	-4.28122200	0.88439300	-2.85497400
C	-4.45652100	1.38925500	-1.42846700
C	-4.14203000	3.94003100	-3.98562000
H	-4.22512800	3.76098700	-5.06119000
H	-5.13126300	3.81611600	-3.53339800
H	-3.80336300	4.96683300	-3.81923400
C	-5.86925400	1.89983100	-1.16019200
H	-6.09571400	2.77642500	-1.77521600
H	-6.59366100	1.11199700	-1.38460300
H	-5.95485100	2.17843400	-0.10624300
H	-4.24832900	0.53431400	-0.78058200
H	-2.16058900	3.09058500	-3.86759700

LLA-TS1

Al	-0.69682	0.24556	-0.03392
O	4.23963	-2.75525	0.45769
C	4.67417	-4.12267	0.56040
H	5.41326	-4.34743	-0.21410
H	5.10959	-4.31182	1.54551
H	3.77535	-4.72394	0.42026
O	0.66225	0.18132	1.35773
O	3.21470	-0.33387	0.77199
O	3.57312	-1.12773	2.86577
O	6.35254	-2.05329	0.86373
C	5.18257	-1.82263	0.65073
C	4.62270	-0.41122	0.45513
C	2.81677	-0.76599	1.98829
C	1.29694	-0.83968	2.09784
C	0.87952	-2.26287	1.72124
C	5.44063	0.65738	1.16414
H	4.63255	-0.23668	-0.62571
H	1.05940	-0.68865	3.15301
H	1.29725	-2.96711	2.44870
H	1.25660	-2.52407	0.73569
H	-0.20822	-2.33518	1.72437
H	5.42573	0.50428	2.24474
H	5.03671	1.64735	0.92957
H	6.47660	0.61047	0.81845
O	-1.06906	0.80366	3.11691
O	-0.00103	3.20103	3.96220
O	-0.76483	2.88824	6.02464
O	-0.95793	1.78830	1.10970
C	-0.37215	1.51650	2.21925
C	0.59278	2.57612	2.79048
C	-0.56626	2.44174	4.92070
C	-1.00473	1.03467	4.54565
C	2.01954	2.15782	3.10489
H	2.06713	1.33110	3.81641
H	2.53297	1.86236	2.18952

H	2.54182	3.01440	3.54117
C	-0.21659	-0.03659	5.29368
H	0.86034	0.03301	5.12080
H	-0.39793	0.08461	6.36573
H	-0.56577	-1.02590	4.98587
H	-2.05205	0.96430	4.85248
H	0.60378	3.38893	2.06538
C	-2.90421	-1.52904	0.32064
C	-3.49601	-2.55617	1.10028
C	-4.55543	-3.33486	0.65186
C	-5.07155	-3.08542	-0.61735
C	-4.54653	-2.07267	-1.41154
C	-3.47697	-1.29573	-0.95817
C	-2.92564	-0.24762	-1.90096
N	-2.34222	0.96651	-1.23883
C	-1.81625	1.89047	-2.28553
C	-0.56495	2.61757	-1.80181
N	0.47063	1.64227	-1.35920
C	1.04857	0.96835	-2.56533
C	1.75749	-0.33143	-2.30947
C	3.03092	-0.57925	-2.81521
C	3.54283	-1.87570	-2.77568
C	2.77121	-2.93608	-2.31297
C	1.49508	-2.67586	-1.81727
C	0.98715	-1.36197	-1.72025
O	-0.18714	-1.09964	-1.17588
C	-3.36089	1.67740	-0.36429
C	1.56837	2.30632	-0.57513
H	-4.96997	-4.11344	1.28142
H	-4.97439	-1.87902	-2.39068
H	-3.71317	0.06620	-2.59633
H	-2.11451	-0.68664	-2.48755
H	-2.57585	2.61730	-2.58516
H	-1.59679	1.30161	-3.17721

H	-0.18409	3.26270	-2.60379
H	-0.80551	3.25034	-0.94436
H	1.70057	1.67060	-3.09484
H	0.21905	0.74155	-3.23745
H	3.60731	0.22417	-3.26308
H	3.15232	-3.95021	-2.32980
H	-2.84109	2.52924	0.07185
H	-3.59054	0.99815	0.45584
H	2.16843	1.49749	-0.16088
H	1.07116	2.81131	0.24771
Cl	-2.86221	-2.85870	2.71613
Cl	-6.42492	-4.05469	-1.21149
Cl	0.48323	-4.03144	-1.33385
Cl	5.19234	-2.18161	-3.32996
C	2.44717	3.28964	-1.31760
C	3.71645	2.89926	-1.76804
C	2.03633	4.61173	-1.54033
C	4.54732	3.79677	-2.44060
H	4.05403	1.88349	-1.58188
C	2.86147	5.51215	-2.21586
H	1.06875	4.94185	-1.17055
C	4.11801	5.10537	-2.67118
H	5.52874	3.47560	-2.77933
H	2.52799	6.53373	-2.37807
H	4.76257	5.80743	-3.19304
C	-4.62731	2.14623	-1.05002
C	-4.73122	3.46279	-1.52507
C	-5.73706	1.29951	-1.18939
C	-5.89478	3.91239	-2.15186
H	-3.89881	4.14945	-1.38521
C	-6.90191	1.74437	-1.81550
H	-5.68902	0.28860	-0.79757
C	-6.98205	3.05025	-2.30478
H	-5.95470	4.93651	-2.51074

H	-7.75003	1.07181	-1.91293
H	-7.89033	3.39737	-2.79010
O	-1.91598	-0.81771	0.81358

LLA-IM2

O	7.07376500	0.74145100	3.25214100
C	7.93535200	0.42898400	2.14287000
H	7.67536800	-0.54152800	1.70924200
H	7.91126400	1.21092700	1.37598000
H	8.94016700	0.37821600	2.56565400
O	1.50773000	-0.66366000	1.29910500
O	3.84239000	0.35013600	1.67547100
O	4.78370100	-1.63812400	2.18436100
O	5.03695500	0.94202600	4.07935400
C	5.74093700	0.83510200	3.10118800
C	5.16007300	0.92764700	1.67943800
C	3.79411100	-0.96522400	1.98102600
C	2.36524500	-1.49870800	2.07303300
C	2.27646800	-2.93075900	1.56016300
C	5.00216200	2.39192100	1.28788600
H	5.76871200	0.38718900	0.95333300
H	2.08631900	-1.47168800	3.13100200
H	2.53993000	-2.96922800	0.49905900
H	1.25022800	-3.28503800	1.67718100
H	2.95689700	-3.58140000	2.11609500
H	4.55378800	2.46963800	0.29463200
H	5.97787200	2.88910200	1.27127300
H	4.36329900	2.90363800	2.01399500
O	-0.28136100	-0.98512300	2.67274800
O	-0.59173300	1.79176500	3.10305800
O	-2.76029400	1.51888600	3.45022900
O	-0.38118900	0.39103000	0.81063200
C	0.37637400	-0.03814500	1.85403100
C	0.69194500	1.19784000	2.73484500
C	-1.68006300	1.00940300	3.23896500
C	-1.55237800	-0.51345200	3.14099100
C	1.49651300	0.97466700	4.00366000
H	1.44023500	1.88104700	4.61544400
H	1.08775700	0.14337000	4.58577500
H	2.55090000	0.79175300	3.78867200
C	-1.82786800	-1.16125400	4.49304500
H	-1.10009100	-0.83530900	5.24387000
H	-2.83306500	-0.89785600	4.83269300
H	-1.76211500	-2.24805300	4.38892000
H	-2.31481700	-0.82251000	2.42269800
H	1.17055300	1.95351100	2.10798700
Al	-1.05016400	-0.38854500	-0.61751000
O	-2.37100900	-1.39587800	0.10842800
C	-3.68005900	-1.44282800	0.00576700
C	-4.44597500	-2.30189300	0.83102400
C	-5.83354900	-2.35828800	0.75970500
C	-6.49396700	-1.54057900	-0.15502600
C	-5.78334400	-0.68302000	-0.98823100
C	-4.39005900	-0.63380300	-0.91323200
C	-3.62323400	0.22260200	-1.88661300
N	-2.44285700	0.97123300	-1.31256200
C	-1.86908500	1.80144400	-2.41476000
C	-0.43169500	2.19249200	-2.11464200
N	0.38442300	0.98228400	-1.85589600
C	0.72811800	0.31795300	-3.14955900
C	1.39421600	-1.01984600	-2.97253200
C	2.59923200	-1.32108800	-3.60687200
C	3.15375600	-2.59052600	-3.46675100
C	2.51639500	-3.57640700	-2.71749800
C	1.30642700	-3.27024200	-2.09737600
C	0.72593700	-1.98803300	-2.19463400
C	-2.83740900	1.82507800	-0.10536100
C	1.62338100	1.30839400	-1.06933500
H	-6.38646900	-3.02666600	1.40941700

H	-6.31152000	-0.05257300	-1.69694200
H	-4.29650600	0.94841300	-2.35599400
H	-3.21233000	-0.40830400	-2.68548800
H	-2.48431300	2.68988300	-2.58229000
H	-1.91754800	1.20414200	-3.32803700
H	-0.03075700	2.78359300	-2.94852700
H	-0.39217200	2.82193500	-1.22224900
H	1.35843100	0.98268300	-3.75301300
H	-0.20722900	0.16870700	-3.69803800
H	3.10184800	-0.56779300	-4.20425500
H	2.94603100	-4.56614200	-2.61685100
H	-1.90921100	2.21044000	0.31529500
H	-3.25011000	1.14024900	0.63515400
H	2.05123000	0.36283900	-0.74223700
H	1.27786900	1.81207800	-0.16803200
Cl	-3.61334000	-3.34281400	1.97465500
Cl	-8.25604000	-1.59110100	-0.25078600
Cl	0.47447600	-4.51584700	-1.18783900
Cl	4.69255600	-2.95828200	-4.25055300
C	2.67842100	2.14160400	-1.76900300
C	3.85317600	1.53504900	-2.23770600
C	2.54736300	3.53171900	-1.90675700
C	4.85664400	2.28743400	-2.85101100
H	3.98398300	0.46500200	-2.10704500
C	3.54623600	4.28816200	-2.52197500
H	1.66723400	4.03131300	-1.51102600
C	4.70224000	3.66709000	-3.00086600
H	5.75889000	1.79605000	-3.20575300
H	3.42732500	5.36434600	-2.61603200
H	5.48164700	4.25648200	-3.47623500
C	-3.80177300	2.95286100	-0.38539700
C	-3.32172600	4.23213700	-0.70396400
C	-5.18716300	2.75780800	-0.30113900
C	-4.20198500	5.28340600	-0.96199900
H	-2.24859400	4.40756500	-0.73498900
C	-6.07067100	3.80692900	-0.55808500
H	-5.57242900	1.78422600	-0.01584700
C	-5.58062900	5.07033700	-0.89547300
H	-3.81237400	6.26866100	-1.20365600
H	-7.14161700	3.63863900	-0.48342800
H	-6.26883800	5.88802800	-1.09189100
O	-0.43684700	-1.72377900	-1.61087800

LLA-IM3

Al	-1.04955000	-0.38319400	-0.62257700
O	7.07304300	0.70505100	3.26320900
C	7.93544500	0.40253900	2.15179200
H	7.67502300	-0.56344900	1.70847500
H	7.91286200	1.19197600	1.39254900
H	8.93978100	0.34668000	2.57507500
O	1.50731900	-0.67628100	1.29291600
O	3.84267000	0.33121600	1.67995300
O	4.78203600	-1.66301900	2.16866100
O	5.03550900	0.89751300	4.09051800
C	5.74036500	0.80032800	3.11196300
C	5.16082700	0.90751900	1.69067000
C	3.79311800	-0.98713400	1.97201200
C	2.36376600	-1.52015600	2.05842200
C	2.27374000	-2.94682300	1.53105400
C	5.00440900	2.37570900	1.31345400
H	5.76963300	0.37381400	0.95973000
H	2.08476100	-1.50352900	3.11658900
H	2.53802400	-2.97488000	0.46983700
H	1.24699500	-3.30107000	1.64364400
H	2.95298300	-3.60383500	2.08091300
H	4.55665000	2.46353300	0.32074700
H	5.98056000	2.87218200	1.30227000
H	4.36551900	2.88084100	2.04413700
O	-0.28238800	-1.01262400	2.66251200

O	-0.59407300	1.75960100	3.12068600
O	-2.76269200	1.48247800	3.46400900
O	-0.38229800	0.38187500	0.81427500
C	0.37523000	-0.05724800	1.85353500
C	0.68991700	1.17003400	2.74668100
C	-1.68221300	0.97553800	3.24792900
C	-1.55402700	-0.54619300	3.13434700
C	1.49455300	0.93451600	4.01321400
H	1.43818200	1.83479100	4.63393500
H	1.08592900	0.09744900	4.58710400
H	2.54895600	0.75381000	3.79636800
C	-1.83067600	-1.20810400	4.47928600
H	-1.10379200	-0.88970500	5.23419900
H	-2.83631400	-0.94861100	4.82062200
H	-1.76442400	-2.29373500	4.36392800
H	-2.31565500	-0.84793200	2.41210300
H	1.16819200	1.93214700	2.12740700
O	-2.37016500	-1.39956700	0.09115500
C	-3.67932900	-1.44483800	-0.01072800
C	-4.44498800	-2.31214600	0.80608400
C	-5.83264100	-2.36731400	0.73504100
C	-6.49336200	-1.54000400	-0.17080400
C	-5.78298300	-0.67405700	-0.99550800
C	-4.38965600	-0.62605200	-0.92076300
C	-3.62296000	0.24038300	-1.88525400
N	-2.44286800	0.98339900	-1.30335800
C	-1.86958700	1.82535700	-2.39690100
C	-0.43251600	2.21420000	-2.09269200
N	0.38430500	1.00201400	-1.84587000
C	0.72854200	0.35126000	-3.14626900
C	1.39620100	-0.98752800	-2.98327300
C	2.60127300	-1.28075300	-3.62124300
C	3.15731900	-2.55093000	-3.49465000
C	2.52140000	-3.54531000	-2.75545800
C	1.31133600	-3.24705600	-2.13166500
C	0.72928400	-1.96454700	-2.21530700
C	-2.83778900	1.82430600	-0.08719400
C	1.62281400	1.32125300	-1.05583300
H	-6.38541400	-3.04217000	1.37814800
H	-6.31135800	-0.03612200	-1.69732600
H	-4.29634500	0.97077000	-2.34729600
H	-3.21157100	-0.38218900	-2.69040900
H	-2.48543500	2.71503200	-2.55530100
H	-1.91758100	1.23747300	-3.31630800
H	-0.03174200	2.81388700	-2.92050600
H	-0.39364500	2.83481600	-1.19422600
H	1.35796000	1.02288500	-3.74300500
H	-0.20681200	0.20661000	-3.69601000
H	3.10269200	-0.52065800	-4.21098200
H	2.95220500	-4.53555400	-2.66532800
H	-1.90977900	2.20567500	0.33750000
H	-3.25010600	1.13143300	0.64604400
H	2.05206300	0.37279600	-0.73906600
H	1.27651500	1.81455900	-0.14912600
Cl	-3.61196600	-3.36513900	1.93833600
Cl	-8.25551300	-1.58903600	-0.26602800
Cl	0.48137400	-4.50315700	-1.23483600
Cl	4.69621300	-2.90866200	-4.28292700
C	2.67652900	2.16386700	-1.74622000
C	3.85178300	1.56449800	-2.22286100
C	2.54359100	3.55535700	-1.86728700
C	4.85384000	2.32549200	-2.82778900
H	3.98417000	0.49316600	-2.10500500
C	3.54104900	4.32043000	-2.47407100
H	1.66304900	4.04904000	-1.46509400
C	4.69753700	3.70663900	-2.96108700
H	5.75652100	1.83959800	-3.18892100
H	3.42064500	5.39749900	-2.55518500

H	5.47585900	4.30267800	-3.42989900
C	-3.80278300	2.95447000	-0.35512400
C	-3.32344800	4.23710400	-0.66099100
C	-5.18802300	2.75809300	-0.27162800
C	-4.20427900	5.29067400	-0.90742000
H	-2.25039600	4.41321100	-0.69118100
C	-6.07211200	3.80950800	-0.51696000
H	-5.57273900	1.78154900	0.00408300
C	-5.58279700	5.07647900	-0.84180300
H	-3.81520500	6.27850200	-1.13924000
H	-7.14293100	3.64011300	-0.44299300
H	-6.27146400	5.89591600	-1.02913800
O	-0.43358200	-1.70769500	-1.62854800

LLA-TS2

Al	0.92987400	0.31617200	-0.04973000
O	-7.83027100	-2.53474600	-1.10887200
C	-7.16749800	-2.14476900	-2.32270300
H	-6.70932100	-1.15906900	-2.20477400
H	-6.41377000	-2.86706900	-2.64573600
H	-7.95401200	-2.08788500	-3.07799700
O	-2.13993000	-0.56331900	-1.78923000
O	-5.02723500	-2.08848700	-0.30677500
O	-3.86029800	-2.75661100	-2.14247300
O	-7.84294800	-3.42355700	0.90634300
C	-7.19686900	-3.11107600	-0.06659900
C	-5.68779400	-3.38174500	-0.16286200
C	-4.08977700	-1.95788900	-1.26347000
C	-3.41508800	-0.59701000	-1.11823200
C	-4.26410600	0.49255200	-1.76709000
C	-5.14315400	-4.05980400	1.07928800
H	-5.47600400	-3.97655300	-1.05589900
H	-3.26001600	-0.37326300	-0.06373300
H	-4.40477700	0.28465700	-2.83248100
H	-3.77187700	1.46258900	-1.65849200
H	-5.24031900	0.54226200	-1.27624300
H	-5.36270100	-3.46627300	1.96913800
H	-4.06020000	-4.17080600	0.99216000
H	-5.60514900	-5.04459100	1.19531400
O	-0.82789700	-0.05457200	0.54269100
O	-1.94588300	-2.61768100	0.37288700
O	-2.44982200	-2.55609900	2.52246000
O	0.05139400	-0.47719600	-1.68671900
C	-1.00307700	-1.01998300	-1.24584500
C	-0.90832700	-2.39702500	-0.60783700
C	-1.87105400	-2.02440000	1.60571900
C	-1.15423000	-0.66912200	1.77464900
C	-0.98244000	-3.51203200	-1.66641600
H	-0.99479800	-4.48283700	-1.16869500
H	-1.88785500	-3.44603100	-2.27508500
H	-0.11156400	-3.45949900	-2.32522300
C	-2.07806400	0.24205900	2.59345200
H	-1.61194700	1.22096800	2.70753600
H	-3.04143000	0.37425400	2.08704900
H	-2.26547900	-0.18517100	3.58118800
H	-0.24140200	-0.87818500	2.34837400
H	0.05383800	-2.42272300	-0.10309000
C	2.84850300	-1.59926300	0.86445800
C	3.07088300	-2.76739600	1.63188000
C	4.33651600	-3.17424000	2.03480700
C	5.43105700	-2.40043900	1.65439500
C	5.26377600	-1.25159900	0.88860500
C	3.98724300	-0.84301300	0.49200600
C	3.86460700	0.45624200	-0.27598700
N	2.71688800	0.52581300	-1.24288600
C	2.69434000	1.87310600	-1.88430800
C	1.26209500	2.31620900	-2.16894300
N	0.44293600	2.27924800	-0.92256600
C	0.85959400	3.41956900	-0.03426000

C	0.45120900	3.28264200	1.40521700
C	-0.23931000	4.28696300	2.07981100
C	-0.44687100	4.17116600	3.45368900
C	0.03686800	3.07628500	4.16609300
C	0.72291400	2.07627000	3.47839000
C	0.93299900	2.14144700	2.08551700
C	2.80668700	-0.56586300	-2.29533900
C	-1.03325000	2.37042800	-1.22410000
H	4.46416800	-4.07345600	2.62592400
H	6.13153200	-0.66903300	0.59389000
H	4.79898400	0.65293200	-0.81429900
H	3.70759900	1.27225600	0.43398800
H	3.27621500	1.87744200	-2.80973400
H	3.18887900	2.57519100	-1.21165300
H	1.26706100	3.32246900	-2.60503400
H	0.79221300	1.63906100	-2.88539400
H	0.49145000	4.35746800	-0.46119200
H	1.94973100	3.46291000	-0.05525000
H	-0.59237400	5.16211900	1.54418300
H	-0.12611800	2.99124300	5.23418500
H	1.93577100	-0.44147100	-2.93628500
H	2.67752400	-1.51094000	-1.76888000
H	-1.54150400	2.18360600	-0.27725600
H	-1.25748200	1.53558100	-1.88315700
Cl	1.67374800	-3.73633400	2.09029500
Cl	7.05498500	-2.89912100	2.13893300
Cl	1.30770700	0.67522200	4.35908300
Cl	-1.32991900	5.43858900	4.30978000
C	-1.52446800	3.66185300	-1.84185000
C	-2.14173000	4.63943400	-1.04794100
C	-1.41319400	3.89218000	-3.22128700
C	-2.61171200	5.82749200	-1.61027800
H	-2.25979000	4.46036200	0.01734100
C	-1.87805100	5.08001900	-3.78713700
H	-0.97172200	3.12908900	-3.85722200
C	-2.47449700	6.05311700	-2.98145000
H	-3.08869700	6.57244200	-0.97900400
H	-1.78476800	5.24167300	-4.85781600
H	-2.84029700	6.97659100	-3.42211000
C	4.06367700	-0.58670700	-3.13894600
C	4.08922400	0.05224400	-4.38811300
C	5.21176800	-1.27447200	-2.71856200
C	5.23791800	0.03513000	-5.18119500
H	3.19399400	0.55246000	-4.75151800
C	6.36204200	-1.29396000	-3.50799900
H	5.20007200	-1.80321200	-1.77059400
C	6.38066900	-0.63372200	-4.73884000
H	5.23631200	0.53467400	-6.14634700
H	7.24109200	-1.83272400	-3.16437700
H	7.27590900	-0.65125100	-5.35461300
O	1.62405100	-1.29011300	0.48823200
O	1.57819900	1.19422600	1.42442200

LLA-IM4

Al	-2.27122800	0.08149600	-0.21695300
O	9.40058900	-1.17365300	-1.75283600
C	10.38057800	-0.27829900	-1.19535400
H	10.07513800	0.76448600	-1.32486000
H	10.57425500	-0.49429100	-0.13925600
H	11.29173300	-0.46081600	-1.76736500
O	3.96331900	0.71555100	0.21664000
O	6.41721300	-0.07592200	-0.01319500
O	6.93655000	1.37979700	-1.66230300
O	7.31236800	-1.87454600	-1.88525900
C	8.13564800	-1.20680500	-1.30217200
C	7.80032900	-0.48247700	0.01250400
C	6.12305700	0.87757600	-0.91542500
C	4.65348700	1.27813900	-0.91728100
C	4.49327900	2.79226300	-0.82448600

C	7.95232100	-1.44018100	1.18738700
H	8.41032800	0.41159200	0.14637400
H	4.20647000	0.92850200	-1.84526900
H	4.87235400	3.16185300	0.13348000
H	3.43335900	3.04482400	-0.90604500
H	5.04416100	3.27143100	-1.63782300
H	7.66908100	-0.93827700	2.11714300
H	8.99027600	-1.77832800	1.27252600
H	7.31096100	-2.31458100	1.04291700
O	-0.93299300	0.44677500	-1.26835700
O	1.56557000	-1.39171500	-1.38555500
O	1.85701500	0.74233200	-2.05311800
O	2.17099900	-0.19210200	1.07338500
C	2.98932000	-0.20129000	0.17093300
C	2.91196300	-1.37618800	-0.83624800
C	1.14574800	-0.24145600	-1.95479900
C	-0.34080800	-0.27954400	-2.31375100
C	3.94425100	-1.59571500	-1.92960500
H	3.76570600	-2.58309800	-2.36721000
H	3.86494800	-0.86039400	-2.73192600
H	4.96017100	-1.59348000	-1.52802700
C	-0.61069800	0.37675900	-3.66020600
H	-0.24714800	1.40870600	-3.64985600
H	-0.11730300	-0.16683100	-4.47542500
H	-1.69008100	0.37763300	-3.84078800
H	-0.67781800	-1.32304500	-2.32795100
H	2.91237600	-2.24794200	-0.17564700
O	-2.92278400	-1.35417300	-1.12936700
C	-3.46633700	-2.49516900	-0.77419600
C	-3.91412700	-3.41945800	-1.74951300
C	-4.47931800	-4.64395400	-1.41241300
C	-4.59798900	-4.97891600	-0.06465000
C	-4.15340900	-4.11255300	0.92857600
C	-3.59204100	-2.88146900	0.58264400
C	-3.17198100	-1.92923100	1.67515800
N	-1.91637500	-1.13679700	1.40207800
C	-1.61567700	-0.31912600	2.61581100
C	-0.74203300	0.87355300	2.25964400
N	-1.37915600	1.67902700	1.18629900
C	-2.46981200	2.51822100	1.77547000
C	-3.34416600	3.16759700	0.73747100
C	-3.58259800	4.54153600	0.73524700
C	-4.42890300	5.09374500	-0.22330000
C	-5.03938200	4.29820100	-1.18879500
C	-4.80040900	2.92524700	-1.17430200
C	-3.96295000	2.32608000	-0.21188600
O	-3.78942000	1.00625600	-0.17688700
C	-0.73332000	-2.01849400	1.01198600
C	-0.35506200	2.53562600	0.47890100
H	-4.81429500	-5.32385900	-2.18711700
H	-4.23926200	-4.39490600	1.97350700
H	-3.03946500	-2.47730400	2.61468700
H	-3.95966800	-1.18247200	1.83802800
H	-1.13186800	-0.93466100	3.37975700
H	-2.57223400	0.00778500	3.02985300
H	-0.55670900	1.47600700	3.15814600
H	0.22603300	0.53423000	1.89288200
H	-2.03614800	3.27107800	2.44456100
H	-3.09201000	1.86005700	2.38857100
H	-3.12126100	5.17561600	1.48485400
H	-5.68743900	4.73238500	-1.94103600
H	0.07247300	-1.34580600	0.72008700
H	-1.03917900	-2.56023900	0.11741100
H	-0.89461700	3.07187800	-0.30432600
H	0.31976400	1.84665400	-0.02343300
Cl	-3.73845700	-2.99905600	-3.44627900
Cl	-5.30475200	-6.53432600	0.38399900
Cl	-5.56080400	1.91364500	-2.38921300

Cl	-4.72678000	6.83408900	-0.22205600
C	0.42489700	3.50329100	1.34596800
C	0.00321000	4.83148700	1.50233800
C	1.61483100	3.10030900	1.97341300
C	0.72916700	5.72855500	2.28807000
H	-0.88941000	5.16912700	0.98388100
C	2.34254200	3.99572300	2.75927100
H	1.98441600	2.08906400	1.82866500
C	1.89897600	5.30995200	2.92528600
H	0.38633500	6.75470500	2.39327800
H	3.26372600	3.66717900	3.23423100
H	2.46820300	6.00677800	3.53513800
C	-0.24479200	-2.97900500	2.07277700
C	0.81626400	-2.61771300	2.91802900
C	-0.81378400	-4.25297200	2.21328400
C	1.27683700	-3.49891900	3.89791900
H	1.29589300	-1.65272400	2.78685700
C	-0.35441900	-5.13431200	3.19283600
H	-1.61135200	-4.55940500	1.54396600
C	0.68840000	-4.75709700	4.04205500
H	2.10105700	-3.20498000	4.54264200
H	-0.80580200	-6.11854200	3.28607200
H	1.04806100	-5.44451000	4.80317100

DLA-IM1

Al	0.90414000	-1.01090800	-0.38671300
O	-0.44541900	-2.20095500	-0.12156800
C	-1.40822500	-2.71002700	-0.85177100
C	-2.40798000	-3.52508500	-0.26762400
C	-3.45240500	-4.06541400	-1.00934100
C	-3.51583200	-3.79405500	-2.37556900
C	-2.55629200	-2.99724000	-2.99197500
C	-1.51019600	-2.45844000	-2.24062200
C	-0.43552900	-1.65199100	-2.92376700
N	0.02675000	-0.42739800	-2.17053200
C	1.01806600	0.29567600	-3.02045700
C	1.86832200	1.22487300	-2.16738600
N	2.50305400	0.45695700	-1.06374500
C	3.68688300	-0.28744600	-1.61199200
C	4.30372500	-1.24200800	-0.62810100
C	5.66899700	-1.22166500	-0.34453100
C	6.20033400	-2.14818700	0.54914000
C	5.39033700	-3.09479300	1.17193000
C	4.02915100	-3.11165100	0.87511900
C	3.45658700	-2.20127600	-0.03670700
O	2.16738100	-2.26513700	-0.35245700
C	-1.13276700	0.47585400	-1.77728300
C	2.91469800	1.36328200	0.07118500
H	-4.20152900	-4.68395300	-0.52883700
H	-2.62326700	-2.78985300	-4.05545100
H	-0.77779900	-1.33862900	-3.91628400
H	0.46143600	-2.26878500	-3.06288000
H	0.50999700	0.85263100	-3.81285100
H	1.63896400	-0.45917700	-3.50814800
H	2.61796400	1.72351300	-2.79374000
H	1.25166300	2.00951200	-1.72582100
H	4.43167400	0.43247200	-1.97009100
H	3.33773200	-0.85663400	-2.47859800
H	6.31263800	-0.48921500	-0.81983200
H	5.80498100	-3.80638300	1.87631200
H	-0.69587800	1.25966300	-1.16066900
H	-1.76712400	-0.10839500	-1.11465600
H	3.28072600	0.71169200	0.86632900
H	1.99407700	1.81332000	0.43700900
Cl	-2.31015800	-3.86248600	1.45191100
Cl	-4.84232500	-4.46082900	-3.33152800
Cl	2.99615300	-4.29484200	1.65575300
Cl	7.92738200	-2.11476700	0.91164400
C	3.94566700	2.42556800	-0.25396100

C	5.29470400	2.22866200	0.07408500
C	3.57092200	3.64129800	-0.84802600
C	6.25264500	3.20258300	-0.21248300
H	5.59225800	1.31064600	0.57171700
C	4.52769300	4.61632600	-1.13647900
H	2.52529500	3.83793800	-1.06346500
C	5.87189000	4.39713000	-0.82725400
H	7.29276600	3.03065600	0.05188500
H	4.21888600	5.55291500	-1.59345300
H	6.61507500	5.15819000	-1.04977100
C	-1.94136000	1.07524100	-2.90514800
C	-1.58827300	2.31380100	-3.46372200
C	-3.09826500	0.43228200	-3.36950400
C	-2.35505900	2.87760400	-4.48456600
H	-0.73528600	2.86145000	-3.07404500
C	-3.86610200	0.99511100	-4.38958400
H	-3.40350700	-0.50722200	-2.92082600
C	-3.49196200	2.21666100	-4.95469100
H	-2.07118300	3.84042100	-4.90039700
H	-4.76024400	0.48333100	-4.73509300
H	-4.09087700	2.65786200	-5.74685500
O	-3.25932500	0.96560700	5.56094600
C	-2.55155100	2.04826700	4.92236600
H	-1.66830700	1.68807900	4.39184300
H	-3.19931800	2.60471800	4.24117400
H	-2.23827900	2.70105800	5.73939600
O	0.58363900	0.03781200	0.99013000
O	-2.18394200	-0.42159000	3.23070300
O	-2.11236600	0.53630400	1.19087500
O	-4.56152600	-0.81130500	5.43532700
C	-3.85706700	-0.01502800	4.85771900
C	-3.60303100	-0.09330900	3.34484900
C	-1.55369600	-0.03939300	2.10630600
C	-0.06541600	-0.39831900	2.15347100

C	0.60742500	0.22897500	3.38104400
C	-4.45074000	-1.15309800	2.66971200
H	-3.76923900	0.88254700	2.88104300
H	-0.03709400	-1.49227800	2.25882000
H	0.56969700	1.32290700	3.32243400
H	1.65899300	-0.07302300	3.39659800
H	0.13312300	-0.09747300	4.31060600
H	-4.19969900	-1.19937500	1.60722300
H	-5.50973200	-0.89734600	2.77402900
H	-4.28285200	-2.13039600	3.12539200
O	-2.02729300	3.88903600	-0.80050800
O	-1.77594300	3.49034900	1.90862200
O	-3.97407300	3.27806700	2.10411000
O	0.15928700	4.10923600	-1.04819700
C	-0.77553700	3.80600800	-0.33409200
C	-0.58851500	3.30692300	1.09140500
C	-3.00068200	3.30767300	1.38441300
C	-3.08965500	3.15575100	-0.12691200
C	0.54134100	4.03361000	1.80345600
H	1.47649700	3.91168700	1.25396400
H	0.31813000	5.10199300	1.88232900
H	0.66352600	3.62308600	2.80929100
C	-4.40227700	3.65730200	-0.69505200
H	-4.53413300	4.72231900	-0.48177400
H	-4.41312500	3.50224600	-1.77698900
H	-5.22871100	3.10398000	-0.24362900
H	-2.95482800	2.09377900	-0.35081900
H	-0.38361800	2.23048100	1.03307700

DLA-TS1

Al	-0.59105400	-0.04779700	0.18005400
O	6.05904100	-1.54272700	-1.79603100
C	6.57352700	-1.23473200	-3.10596200
H	5.76794800	-1.23723100	-3.84531200
H	7.10784600	-0.27874300	-3.11448500

H	7.27869400	-2.03677800	-3.33054700
O	0.69028800	-0.53743300	-1.20063000
O	3.24896100	0.66884500	-1.82089100
O	3.10197400	-1.07619600	-3.24271200
O	4.53336200	-1.23649500	-0.23375700
C	5.06426300	-0.83153500	-1.24564500
C	4.68385100	0.53458900	-1.83977600
C	2.56481100	-0.20511200	-2.58030200
C	1.05640900	0.00573700	-2.46080900
C	0.66771700	1.45950200	-2.67369600
C	5.23312600	1.65557200	-0.96854300
H	5.01615000	0.63914200	-2.87270000
H	0.59258100	-0.60201900	-3.24479200
H	0.90105400	1.75375800	-3.70445800
H	1.22295500	2.10643400	-1.99839700
H	-0.40061500	1.58228500	-2.50300200
H	4.92478700	2.62448600	-1.37041300
H	6.32732900	1.61605100	-0.94135800
H	4.84828900	1.56031800	0.04972800
O	-1.10319200	-2.21230600	-2.04210300
O	0.37137200	-4.45656200	-1.96175100
O	-0.98882600	-5.37234100	-3.47027800
O	-0.48046100	-2.02811800	0.08529200
C	-0.11850600	-2.25048200	-1.12162900
C	1.01252000	-3.21032200	-1.53924500
C	-0.54939900	-4.37858200	-2.94547500
C	-0.97392500	-2.95309800	-3.28393000
C	2.03942800	-3.61272800	-0.50852200
H	1.56275700	-3.95239400	0.41487600
H	2.62354400	-4.44069500	-0.92123200
H	2.72730000	-2.79305200	-0.30194300
C	-2.31715100	-2.88651500	-3.97944300
H	-3.08874200	-3.35154600	-3.35948600
H	-2.58647200	-1.84349500	-4.16002600
H	-2.26430200	-3.42257900	-4.93038200
H	-0.19370300	-2.49626600	-3.90644400
H	1.51808400	-2.77638200	-2.40707100
C	-3.02375500	0.94916800	-0.93999500
C	-3.69713800	1.37625900	-2.11343800
C	-4.87290700	2.11599600	-2.08851300
C	-5.42040000	2.45058800	-0.85269700
C	-4.80307800	2.05483100	0.32825400
C	-3.61697700	1.31698200	0.29660000
C	-2.95589200	0.99760700	1.61877300
N	-2.16071300	-0.27372400	1.65426000
C	-1.55007700	-0.42246400	3.00806100
C	-0.16003400	-1.03979000	2.91760600
N	0.70297300	-0.27071400	1.97568600
C	1.07218500	1.03266200	2.61099400
C	1.60038900	2.07645800	1.66918100
C	2.78888700	2.75781500	1.91714600
C	3.10251100	3.88347600	1.15689000
C	2.21308700	4.37763100	0.20699700
C	1.02204800	3.68950500	-0.02550900
C	0.72282600	2.47622100	0.63381300
O	-0.36036100	1.77275100	0.36220500
C	-3.01222600	-1.48451300	1.31290600
C	1.94901700	-1.03867000	1.62537000
H	-5.35008300	2.42045500	-3.01261000
H	-5.24697500	2.31923800	1.28351600
H	-3.71491700	0.96783200	2.40964300
H	-2.25091500	1.79742100	1.86209500
H	-2.18325900	-1.03043800	3.65948700
H	-1.50448400	0.56451500	3.47089300
H	0.28824000	-1.07769600	3.91794800
H	-0.22607300	-2.06141000	2.53770900
H	1.77795000	0.84855600	3.42742400
H	0.16642500	1.44638000	3.05880400

H	3.44821300	2.43295400	2.71523000
H	2.43230000	5.28647300	-0.34139700
H	-2.34459600	-2.34342100	1.36060600
H	-3.30192400	-1.37220500	0.26865900
H	2.44484600	-0.49687100	0.82037600
H	1.58837900	-1.97359200	1.21759500
Cl	-3.00815800	0.96231600	-3.68198300
Cl	-6.92421600	3.37697800	-0.78977800
Cl	-0.15491200	4.38242300	-1.13302800
Cl	4.63579400	4.71910400	1.42854600
C	2.92243700	-1.30772600	2.75293900
C	4.07355500	-0.52098400	2.89805600
C	2.72073600	-2.36537100	3.65151800
C	4.98045100	-0.75928700	3.93106300
H	4.26574000	0.26669200	2.17694400
C	3.62397300	-2.60762600	4.68801300
H	1.85789200	-3.01545000	3.53089500
C	4.75339200	-1.79961800	4.83489400
H	5.86870800	-0.13974300	4.02386500
H	3.45113600	-3.43317400	5.37363300
H	5.45947500	-1.98901400	5.63909100
C	-4.22390900	-1.72468600	2.18824500
C	-4.14915700	-2.61132100	3.27334200
C	-5.45439100	-1.10838800	1.91600700
C	-5.25971600	-2.85179800	4.08436400
H	-3.21616200	-3.13418400	3.47313400
C	-6.56675400	-1.34500700	2.72436000
H	-5.54141200	-0.44601500	1.06069400
C	-6.47120800	-2.21267500	3.81493400
H	-5.18076800	-3.54454700	4.91807700
H	-7.51084700	-0.85745100	2.49587200
H	-7.33873900	-2.39976900	4.44216600
O	-1.93811700	0.21636500	-1.03188000

DLA-IM2

O	6.72024900	-2.04807900	2.10323700
C	7.34550200	-2.41575000	0.86015500
H	6.82094900	-3.25505700	0.39304100
H	7.40548100	-1.56914700	0.16788600
H	8.35669000	-2.72633000	1.12862600
O	0.82472900	-1.65840400	1.03820500
O	3.33122800	-1.45444700	0.98887200
O	3.70573900	-3.67503700	1.16848700
O	4.90100300	-1.46976500	3.21569500
C	5.44206100	-1.63019600	2.14556600
C	4.74241100	-1.25866900	0.82784900
C	2.94344600	-2.73125000	1.18446700
C	1.45335400	-2.84564100	1.50494100
C	0.83204700	-4.09696000	0.89598300
C	4.94054800	0.22430600	0.53675200
H	5.08161300	-1.87574000	-0.00487500
H	1.41015700	-2.90848200	2.60128000
H	0.81579000	-4.00849900	-0.19278300
H	-0.19602900	-4.19173800	1.24783000
H	1.40906100	-4.98158200	1.18002300
H	4.35777900	0.49955500	-0.34379400
H	5.99496000	0.44446300	0.33941100
H	4.61089100	0.82312300	1.39065400
O	-1.29265900	-1.97551300	1.82794500
O	-0.95131600	0.15654800	3.69665100
O	-3.15950500	0.40264900	3.63644400
O	-0.55422200	0.06056400	1.02704300
C	-0.21179000	-1.04467900	1.74716800
C	0.15437700	-0.64843600	3.20334300
C	-2.19251700	-0.28577300	3.40062700
C	-2.26274300	-1.71720000	2.85411200
C	1.42602700	0.14662400	3.40075900
H	2.29413100	-0.41673300	3.04973900
H	1.38512800	1.09559700	2.86612200

H	1.55834500	0.35884000	4.46666000	H	-1.69396300	2.60111000	-2.50493600
C	-3.63981200	-2.08215200	2.33218500	H	-0.35854400	4.12521800	-1.02139900
H	-3.91786700	-1.44796500	1.48767900	H	-0.94506300	3.25359600	0.39052100
H	-3.63346900	-3.12064100	1.99127900	H	1.50749000	3.30910300	-2.13542800
H	-4.38795300	-1.96390200	3.12060300	H	0.31346400	2.24278100	-2.88317100
H	-2.02378200	-2.35896900	3.72010400	H	3.66699000	2.67823600	-2.89496200
H	0.20194500	-1.56298200	3.80703900	H	4.78394600	-1.47368900	-3.08982100
Al	-0.71954200	0.08007000	-0.74140400	H	-2.27768700	1.74407500	1.12217000
O	-1.73407500	-1.33776700	-1.20441100	H	-3.35576400	0.45526700	0.67220300
C	-3.02287300	-1.56161300	-1.31027600	H	2.00649500	1.35712700	0.28055800
C	-3.57267100	-2.85203900	-1.11851200	H	0.70139500	1.99832400	1.25185200
C	-4.93547000	-3.10083600	-1.25151500	Cl	-2.50756400	-4.18336900	-0.71358600
C	-5.78503100	-2.05090800	-1.59470400	Cl	-7.51755900	-2.35334500	-1.75872300
C	-5.28628700	-0.77015300	-1.81043600	Cl	2.37398000	-2.75604100	-2.28432800
C	-3.91965200	-0.52714400	-1.66850900	Cl	5.98397400	1.10455500	-3.54375000
C	-3.34223800	0.82250100	-1.99302800	C	2.06906900	3.50097400	0.54426000
N	-2.39038400	1.37065000	-0.95866800	C	3.36631900	3.65727300	0.03640900
C	-1.92738300	2.70302300	-1.44175600	C	1.49519500	4.57227000	1.24551700
C	-0.70014000	3.14476500	-0.66711600	C	4.05966900	4.85837800	0.19521000
N	0.36495100	2.11917400	-0.78682500	H	3.83995200	2.82657400	-0.47542900
C	1.07207100	2.30302600	-2.09480700	C	2.18274400	5.77598000	1.40448600
C	2.13019700	1.26732400	-2.35744900	H	0.50905500	4.45678100	1.68796800
C	3.40956700	1.63048400	-2.77939000	C	3.46594400	5.92470900	0.87315700
C	4.35438000	0.64310700	-3.04543300	H	5.06580700	4.95691800	-0.20387900
C	4.04468700	-0.70640400	-2.89094600	H	1.72153600	6.59259300	1.95365600
C	2.76330800	-1.05895000	-2.47390600	H	4.00427000	6.86009200	1.00010700
C	1.76861600	-0.08944000	-2.21268600	C	-4.23052900	2.41323900	0.51801100
C	-3.05509500	1.46868300	0.41276400	C	-4.03507200	3.76862300	0.82469800
C	1.31558100	2.19707000	0.37713700	C	-5.54252300	1.94739600	0.35809300
H	-5.32431800	-4.09935000	-1.08876600	C	-5.11736600	4.64264800	0.93049300
H	-5.95598100	0.03547300	-2.09375300	H	-3.02791800	4.13948900	0.99937100
H	-4.14476900	1.55139900	-2.15168500	C	-6.62828400	2.81746400	0.46565000
H	-2.76320200	0.75796200	-2.92296600	H	-5.71355400	0.89325200	0.16727600
H	-2.72781100	3.44431400	-1.35968700	C	-6.41798100	4.16951800	0.74337300

H	-4.94591400	5.68864000	1.17031000
H	-7.63818200	2.43542300	0.34282900
H	-7.26269000	4.84774700	0.82902400
O	0.53822000	-0.44246000	-1.89459200

DLA-IM3

Al	1.25828900	-0.32380700	0.59770000
O	-6.34130200	1.16204900	-4.07254900
C	-7.63663900	0.74715300	-3.60324500
H	-7.63824500	-0.31799900	-3.35188500
H	-7.97112800	1.34348100	-2.74744700
H	-8.31385200	0.92033200	-4.44143700
O	-1.86619900	-0.84934300	-0.91820700
O	-4.26411700	0.09640200	-1.30589200
O	-4.85540000	-1.67590300	-2.57791600
O	-4.15680400	1.27898100	-3.77926600
C	-5.24510000	1.06161300	-3.29836200
C	-5.42633800	0.78777700	-1.79604200
C	-4.08425400	-1.14138900	-1.80622300
C	-2.84589300	-1.82754600	-1.24074300
C	-3.20744300	-2.62360200	0.01510300
C	-5.51142000	2.10813700	-1.04033400
H	-6.29978300	0.16304300	-1.60392400
H	-2.50049700	-2.51674500	-2.01559000
H	-3.54621500	-1.94957300	0.80792100
H	-2.31570600	-3.14929800	0.36229100
H	-3.99845100	-3.35129700	-0.19628800
H	-5.59247100	1.92496200	0.03390900
H	-6.38735900	2.67742800	-1.36943500
H	-4.61501100	2.70501600	-1.22562600
O	-0.11259000	-2.30402300	-1.22509200
O	0.70851900	-1.02986900	-3.54081000
O	2.04112400	-2.75946900	-3.97157400
O	0.21557200	-0.08697300	-0.78777800
C	-0.55867100	-0.97448900	-1.45845600
C	-0.57075900	-0.65571500	-2.96786200
C	1.10905100	-2.30917300	-3.34999500
C	0.26145600	-3.10571100	-2.35572500
C	-0.80774800	0.80794000	-3.26953800
H	-1.79281600	1.10318300	-2.89937800
H	-0.03405400	1.42042300	-2.80010500
H	-0.78672100	0.96995300	-4.35131000
C	0.96153500	-4.33974000	-1.82197300
H	1.81914200	-4.04589700	-1.21502900
H	0.26770500	-4.91306600	-1.20070700
H	1.30538800	-4.96303400	-2.65130800
H	-0.64593100	-3.41068700	-2.90549300
H	-1.34899000	-1.25691700	-3.45381800
O	2.71456700	-1.16326900	-0.03426900
C	4.01003000	-0.90466200	0.00238600
C	4.93867800	-1.69653900	-0.71477000
C	6.30556500	-1.43577500	-0.69595800
C	6.77875800	-0.35742400	0.04747000
C	5.90284400	0.45196500	0.76083800
C	4.53273100	0.18123700	0.74027100
C	3.61255900	1.00958300	1.59313500
N	2.29001100	1.40529700	0.96961800
C	1.64310400	2.32505900	1.96250100
C	0.16496100	2.54498300	1.69996100
N	-0.56928000	1.27609900	1.73215600
C	-0.76874300	0.79165400	3.11659600
C	-1.15402200	-0.66564000	3.16034400
C	-2.33778300	-1.08563300	3.76634900
C	-2.65019300	-2.44244700	3.81712600
C	-1.79521000	-3.39806700	3.27805200
C	-0.61128500	-2.97178200	2.67761600
C	-0.26428800	-1.60851500	2.59968000
C	2.46322100	2.07107200	-0.39400700
C	-1.84816000	1.36029800	0.97575000

H	6.98523200	-2.06333000	-1.26051700
H	6.28105500	1.29436200	1.33130800
H	4.12660700	1.92361200	1.90783700
H	3.34872900	0.45054500	2.49976300
H	2.17519400	3.28094600	1.96753500
H	1.78737400	1.87539700	2.94713900
H	-0.21676500	3.27792700	2.42810400
H	0.02296300	2.98562100	0.71049400
H	-1.51418200	1.40223000	3.64645800
H	0.17777700	0.90907300	3.65226700
H	-3.00675800	-0.35713100	4.21203700
H	-2.03959800	-4.45305200	3.31641400
H	1.45653400	2.20395900	-0.79300900
H	2.95916600	1.33677200	-1.03059100
H	-2.30522500	0.37452300	1.01562100
H	-1.57934000	1.51826000	-0.06848000
Cl	4.35573400	-3.03955500	-1.66933400
Cl	8.50899700	-0.00828900	0.07063800
Cl	0.47161300	-4.16971700	1.99592100
Cl	-4.15999600	-2.96012100	4.57230600
C	-2.83674900	2.41368800	1.43802400
C	-3.85513400	2.09066100	2.34649300
C	-2.78733800	3.72291800	0.93729400
C	-4.78237400	3.04895400	2.76135000
H	-3.93575700	1.06980400	2.70946200
C	-3.71191400	4.68559200	1.34581000
H	-2.03047600	3.98479400	0.20169500
C	-4.71079900	4.35191700	2.26369300
H	-5.56539200	2.77552800	3.46404000
H	-3.65960600	5.69251400	0.93963000
H	-5.43360700	5.09897100	2.58064200
C	3.22050700	3.37933900	-0.40754000
C	2.54478000	4.59846500	-0.24502400
C	4.60622300	3.40313600	-0.61788400
C	3.23902500	5.80854000	-0.26302200
H	1.46540300	4.60135900	-0.11480000
C	5.30336100	4.61192400	-0.63720600
H	5.13829800	2.47126700	-0.77861600
C	4.62255700	5.81712800	-0.45381900
H	2.69922800	6.74323900	-0.13743300
H	6.37689200	4.61068200	-0.80531100
H	5.16477600	6.75863400	-0.47162400
O	0.89657400	-1.22868500	2.06102500

DLA-TS2

Al	0.70085900	0.02823400	0.16762000
O	-6.11555600	0.02372300	0.01586200
C	-6.91836700	-1.16647600	-0.09902400
H	-7.67088600	-1.20875500	0.69631400
H	-7.41354400	-1.07472100	-1.06556200
H	-6.29286400	-2.06119000	-0.09223100
O	-1.38132400	-2.17069100	2.52940800
O	-3.77308000	-1.36580000	2.18724100
O	-4.40480400	-3.13680100	0.91247900
O	-4.37496400	1.08203400	0.86792200
C	-5.16110300	0.16646100	0.94722100
C	-5.11086700	-0.79462800	2.15318200
C	-3.56896600	-2.51778700	1.53889600
C	-2.13038200	-3.02030100	1.65332600
C	-2.08834100	-4.44453700	2.20138600
C	-5.30390400	-0.01541500	3.46628200
H	-5.81742400	-1.61767400	2.06286200
H	-1.69213800	-2.99377500	0.65352800
H	-2.45147400	-4.46619500	3.23327000
H	-1.05878000	-4.81267800	2.18290900
H	-2.71770200	-5.09336800	1.58748700
H	-4.58233000	0.80483400	3.50195000
H	-5.13800000	-0.66047200	4.33323700
H	-6.30232500	0.42187800	3.53237600

O	-0.65228800	0.33743800	1.41221700
O	0.93599500	0.19243300	3.60478400
O	0.47211700	2.31171600	4.04498600
O	0.26424400	-1.82032000	1.00895900
C	-0.25165600	-1.56240700	2.12924300
C	0.47727100	-1.14039400	3.43716900
C	0.16838400	1.31213400	3.44572200
C	-0.99729600	1.21011500	2.45930600
C	1.71568700	-2.02327600	3.57806800
H	1.45059700	-3.08436100	3.51498000
H	2.41599600	-1.76662200	2.78154600
H	2.18518700	-1.82631800	4.54472500
C	-1.45187200	2.58572600	1.99188000
H	-1.80805900	3.16890300	2.84563500
H	-0.62121600	3.12695200	1.54072600
H	-2.26370600	2.47167400	1.27043900
H	-1.83544800	0.73154000	2.99586900
H	-0.24124900	-1.35333600	4.23578300
C	3.43537600	0.52195800	0.65692200
C	4.36170700	1.12765300	1.54353700
C	5.64076300	1.50124800	1.14656100
C	6.03371600	1.26761600	-0.16898200
C	5.16172300	0.67312900	-1.07147500
C	3.87242100	0.30295500	-0.67385700
C	2.95992600	-0.26027200	-1.74170600
N	1.91386500	-1.21489500	-1.25094100
C	1.07443200	-1.67412600	-2.39304300
C	-0.40513000	-1.68907500	-2.01744500
N	-0.81927500	-0.38341700	-1.44484800
C	-0.78560000	0.65848000	-2.52802400
C	-0.89185900	2.07489500	-2.04163900
C	-1.81940900	2.97115300	-2.57031300
C	-1.75654400	4.31469400	-2.20840200
C	-0.76132900	4.77952100	-1.35087300
C	0.16515400	3.87695100	-0.83300500
C	0.10371000	2.50023000	-1.13602000
C	2.53122800	-2.40469000	-0.54342200
C	-2.20072500	-0.42697100	-0.85371000
H	6.31591100	1.96541400	1.85543600
H	5.48514600	0.49392600	-2.09276900
H	3.56348100	-0.75187700	-2.51559400
H	2.41906700	0.56611600	-2.21133000
H	1.37550100	-2.67055000	-2.72683800
H	1.24898700	-1.01596400	-3.24592400
H	-1.00715400	-1.94718300	-2.89854600
H	-0.58139500	-2.45138000	-1.25470500
H	-1.56825800	0.43308700	-3.26210600
H	0.17373800	0.56422300	-3.03846700
H	-2.56891800	2.63363600	-3.27866400
H	-0.70474400	5.82819600	-1.08422700
H	1.69671400	-3.02114800	-0.21128300
H	3.01022400	-2.01405100	0.35245400
H	-2.39363400	0.56380700	-0.44790500
H	-2.13972600	-1.08585600	0.00380700
Cl	3.87427100	1.42931700	3.19479400
Cl	7.65828900	1.71803400	-0.68673800
Cl	1.43440100	4.45395700	0.22425000
Cl	-2.93833300	5.44777100	-2.86093400
C	-3.32072300	-0.84320700	-1.78371000
C	-4.13263700	0.12920300	-2.38307800
C	-3.60335500	-2.19424300	-2.03165200
C	-5.17466700	-0.23469400	-3.23614800
H	-3.96869400	1.17400100	-2.14053000
C	-4.64655600	-2.56449700	-2.88096600
H	-3.02143100	-2.96667700	-1.53704400
C	-5.42925900	-1.58327000	-3.49418400
H	-5.79536300	0.53509500	-3.68633400
H	-4.85305500	-3.61695100	-3.05549600

H	-6.24092800	-1.86937500	-4.15797800
C	3.49915500	-3.24323400	-1.35200800
C	3.06389900	-4.41897600	-1.98175000
C	4.85396200	-2.89453800	-1.45799800
C	3.94365800	-5.20709500	-2.72558700
H	2.02733600	-4.73244500	-1.87121000
C	5.73656000	-3.67861200	-2.20103400
H	5.21771800	-2.00716400	-0.94964400
C	5.28320500	-4.83353600	-2.84259600
H	3.58532800	-6.11481000	-3.20384300
H	6.78187800	-3.39021500	-2.27119000
H	5.97230300	-5.44490900	-3.41887500
O	2.25011400	0.13367200	1.10104900
O	0.97167200	1.63713700	-0.63633400

DLA-IM4

Al	-1.70562300	0.49143000	-0.21477400
O	8.50908200	-2.27005200	-1.70388600
C	9.49547900	-1.43048500	-1.07377900
H	9.30663200	-0.37522400	-1.29407300
H	9.53960500	-1.59526700	0.00755000
H	10.44732800	-1.72653800	-1.51730400
O	3.01366800	-0.02504800	-0.38943800
O	5.43778000	-0.90615200	-0.34336500
O	6.15597400	0.44283700	-2.01145800
O	6.39208300	-2.78418400	-2.05973600
C	7.20481300	-2.17418700	-1.40285700
C	6.79285000	-1.36477900	-0.16108600
C	5.26222800	-0.01197500	-1.33169000
C	3.79210600	0.35287900	-1.53631400
C	3.60597200	1.85122900	-1.73087900
C	6.78891000	-2.25176300	1.07792400
H	7.42889600	-0.48903000	-0.02370300
H	3.46486600	-0.17911900	-2.43553400
H	3.87704300	2.38882700	-0.81765300
H	2.56044000	2.06347900	-1.96884100
H	4.23956900	2.19631400	-2.55116000
H	6.44035600	-1.67981500	1.94273900
H	7.79741600	-2.62281600	1.28636800
H	6.12546800	-3.10864900	0.92866700
O	-0.20086000	0.05140200	-1.01567800
O	0.96094900	-2.75406000	-1.33490300
O	-1.04062600	-2.73081200	-2.25046000
O	2.04454600	-1.45876500	0.95712200
C	2.45010400	-1.23035300	-0.16223400
C	2.31600300	-2.27313800	-1.28386000
C	-0.04504900	-2.09698100	-1.99949100
C	0.09215500	-0.58063200	-2.24073200
C	3.18726200	-3.48063200	-0.95912600
H	4.24471700	-3.20712900	-0.99597600
H	2.93137800	-3.86233100	0.03335600
H	3.00233900	-4.26154500	-1.70214000
C	-0.77354900	-0.12432800	-3.40779900
H	-1.81750300	-0.38756300	-3.23913400
H	-0.68805600	0.96307600	-3.51016900
H	-0.43640900	-0.59120700	-4.34132200
H	1.13718300	-0.34456100	-2.48435400
H	2.59469500	-1.87502800	-2.26025200
O	-2.96171700	-0.45700700	-1.11595400
C	-3.92220000	-1.29531900	-0.80690500
C	-4.71536500	-1.90381200	-1.81106600
C	-5.73119300	-2.80492800	-1.51199900
C	-5.98136000	-3.12133100	-0.17834600
C	-5.23291100	-2.54597100	0.84228800
C	-4.21563600	-1.64004900	0.53515400
C	-3.47468700	-0.95655400	1.65455300
N	-1.99644600	-0.76685400	1.43205000
C	-1.41869300	-0.16151500	2.66839600
C	-0.17503300	0.64156900	2.33101100

N	-0.48748700	1.64318200	1.27914800
C	-1.30641100	2.75325800	1.86431800
C	-1.73103000	3.77700500	0.84721000
C	-1.56822600	5.14145800	1.08596600
C	-1.99179800	6.06558300	0.13462800
C	-2.56918900	5.64998200	-1.06247900
C	-2.73179500	4.28558200	-1.28978700
C	-2.33414500	3.31576000	-0.34337900
O	-2.55173300	2.02910400	-0.56122400
C	-1.29123400	-2.07381100	1.07494100
C	0.77846000	2.17622300	0.65266300
H	-6.31406000	-3.25254800	-2.30846500
H	-5.44057700	-2.79802600	1.87775300
H	-3.61905400	-1.51014400	2.58929600
H	-3.88329100	0.05153300	1.80636800
H	-1.18728800	-0.93593800	3.40418200
H	-2.18469500	0.47719200	3.11337800
H	0.21460000	1.12127600	3.23741600
H	0.60477100	-0.01454200	1.94153700
H	-0.74620200	3.23137300	2.67730300
H	-2.20106900	2.30423400	2.30648300
H	-1.11475500	5.48109400	2.01155100
H	-2.88757000	6.36983900	-1.80741200
H	-0.27560800	-1.80428600	0.79286200
H	-1.78475900	-2.45725800	0.18261100
H	0.46953600	2.89790800	-0.10665000
H	1.22534200	1.33384100	0.13576900
Cl	-4.42506500	-1.49324200	-3.49214700
Cl	-7.26211600	-4.27261900	0.21676400
Cl	-3.45607500	3.75274000	-2.79593800
Cl	-1.78075000	7.79149700	0.44171200
C	1.77859600	2.80425300	1.60210800
C	1.79140000	4.18745600	1.82987700
C	2.74255200	2.00939100	2.24428100
C	2.72106000	4.76282900	2.69844800
H	1.07727000	4.81860200	1.30949500
C	3.67131600	2.58232500	3.11415000
H	2.77532200	0.94321700	2.04148300
C	3.65970200	3.95966000	3.34895000
H	2.71575600	5.83764300	2.86023200
H	4.41107000	1.95292100	3.60259200
H	4.38496800	4.40510500	4.02489800
C	-1.25373500	-3.12597800	2.15880800
C	-0.12075000	-3.24281500	2.97903600
C	-2.31596500	-4.02139100	2.34639100
C	-0.06540500	-4.20960800	3.98412800
H	0.72762100	-2.58649200	2.80580800
C	-2.26222100	-4.98877500	3.35081900
H	-3.17850700	-3.97223300	1.68964500
C	-1.13998200	-5.08051400	4.17730800
H	0.82054700	-4.28886200	4.60886600
H	-3.09285000	-5.67773100	3.47983600
H	-1.09737000	-5.83583900	4.95769800

(A3C3B1)Al-(L-LA)$_2$

Al	-0.59567400	-0.29505100	0.29483300
O	-2.00474900	0.79035400	0.70975400
C	-3.25746100	0.56550500	0.37113100
C	-4.12480000	1.65657300	0.04664400
C	-5.44783400	1.36296800	-0.26283600
C	-6.01003400	0.06326500	-0.26636900
C	-5.16305700	-0.97818500	0.06266300
C	-3.80033000	-0.75674800	0.36715300
C	-3.01416800	-1.88881200	0.78310700
N	-1.72621900	-1.89663200	0.92717700
C	-1.08677200	-2.99779500	1.63161500
C	-0.77785300	-2.59252400	3.10941600
C	-0.63835200	-1.05036000	3.23557000
N	0.18571000	-0.43705300	2.19226700

C	1.36806200	-0.01737100	2.51946000
C	2.29780600	0.65950700	1.65784200
C	3.61028500	0.86825900	2.14091000
C	4.56239000	1.52761300	1.38685300
C	4.15341400	1.97881600	0.10685600
C	2.87822400	1.82154200	-0.42487600
C	1.89360500	1.15521700	0.38100900
O	0.65936600	1.02898200	-0.03294500
C	-3.58363500	3.09972800	0.01507700
C	-3.06368400	3.50094700	1.41600600
C	-2.43612000	3.20381300	-1.01798000
C	-4.66728900	4.11890900	-0.38702200
C	-7.49358900	-0.12192100	-0.62402000
C	-7.92315700	-1.59903400	-0.57113500
C	-8.37122000	0.66963500	0.37265800
C	-7.75026400	0.40226300	-2.05570700
C	-1.94283400	-3.01752300	4.01780000
C	0.52073700	-3.28445600	3.54777400
C	5.99954700	1.79138100	1.86451900
C	6.24773200	1.24856600	3.28310100
C	7.00361000	1.10794500	0.90795700
C	6.26775800	3.31380500	1.87734800
C	2.52215900	2.32412000	-1.83652700
C	3.74050600	2.91605100	-2.57056700
C	1.44263700	3.42684000	-1.73996000
C	1.98681900	1.15048900	-2.69147800
H	-6.10397900	2.18445700	-0.52291100
H	-5.52257600	-2.00254400	0.09054600
H	-3.57096100	-2.80066900	1.03191700
H	-0.15255800	-3.23012600	1.11609500
H	-1.71227500	-3.90117600	1.61963500
H	-1.62907600	-0.59157500	3.16657900
H	-0.23478100	-0.81743600	4.22972800
H	1.70520000	-0.17899600	3.54963400
H	3.84671600	0.48929600	3.13094000
H	4.89642900	2.48553900	-0.49696900
H	-3.87093800	3.45328100	2.15814600
H	-2.68562400	4.53141300	1.39653100
H	-2.25488100	2.84418300	1.74020900
H	-1.61585400	2.53067700	-0.76945300
H	-2.04706100	4.23003900	-1.04697000
H	-2.79464900	2.94988100	-2.02305100
H	-5.50689300	4.13249400	0.31884500
H	-5.06442600	3.92526700	-1.39074900
H	-4.23208600	5.12501200	-0.39668600
H	-8.14540900	1.74146400	0.35446200
H	-7.79317100	-2.02525500	0.43078800
H	-7.35691100	-2.21292400	-1.28143200
H	-8.98433900	-1.68787700	-0.83154200
H	-8.21652900	0.31374400	1.39826300
H	-9.43453900	0.55134800	0.12794200
H	-7.50589100	1.46586100	-2.15132300
H	-8.80654800	0.27923700	-2.32725100
H	-7.14409700	-0.14557300	-2.78669600
H	-2.89551100	-2.61257900	3.65433900
H	-1.79803300	-2.65627000	5.04333700
H	-2.03075300	-4.10967400	4.05640000
H	1.37806900	-2.91767700	2.97077100
H	0.45574200	-4.36909400	3.39595600
H	0.72380700	-3.10859800	4.61118200
H	5.58527100	1.71838500	4.01931000
H	7.27994700	1.45776400	3.58688800
H	6.10271000	0.16282100	3.33520200
H	8.03524600	1.30703200	1.22426900
H	6.89893100	1.46977200	-0.12068100
H	6.85759600	0.02099800	0.90253600
H	5.57383600	3.82637100	2.55366800
H	6.15143600	3.75711900	0.88239500

H	7.29063700	3.52210800	2.21570000
H	3.43514500	3.24608100	-3.56996100
H	4.54339300	2.17942700	-2.70146900
H	4.15536800	3.78866100	-2.05214600
H	0.54260400	3.05499400	-1.24934800
H	1.16778600	3.77732900	-2.74311000
H	1.81808000	4.28742000	-1.17230600
H	1.06565200	0.74760700	-2.27072000
H	2.73165900	0.34611900	-2.76618600
H	1.77491500	1.49445900	-3.71157700
O	5.23254100	-1.84266500	-2.63289100
C	5.55121500	-1.09709100	-1.44100400
H	4.87275100	-0.25035000	-1.30468100
H	5.54164800	-1.73471300	-0.55095700
H	6.56376200	-0.72627500	-1.60259500
O	-1.06912000	-0.60585200	-1.44400300
O	1.77287200	-2.50952600	-2.16113800
O	0.98029200	-1.64808300	-0.24457000
O	3.80811800	-3.03703100	-3.82976000
C	4.05774500	-2.48321200	-2.78684900
C	3.10910500	-2.54685400	-1.58372500
C	0.81737200	-1.96967000	-1.42920900
C	-0.53609700	-1.70650900	-2.08575000
C	-0.45833600	-1.53546600	-3.60252800
C	3.31305300	-3.84144000	-0.80485400
H	3.20486800	-1.68241100	-0.92810800
H	-1.11955000	-2.63266300	-1.87881900
H	0.16066000	-0.67305400	-3.86398400
H	-1.46965200	-1.35930500	-3.98104900
H	-0.04629200	-2.42616600	-4.08816600
H	2.61529500	-3.88169000	0.03599100
H	4.33425300	-3.89245700	-0.41158200
H	3.14781900	-4.70123600	-1.46067400

LLA-IM1

Al	-0.81537000	-0.39192600	0.91784600
O	2.36300800	5.43403800	0.34539700
C	3.44977800	5.45867500	-0.60161600
H	3.59532700	4.48324400	-1.07194500
H	3.29150700	6.22260000	-1.37073800
H	4.33190300	5.72451900	-0.01596200
O	-0.66510900	0.44806900	-0.62750000
O	-0.19745900	3.84777400	-1.63718400
O	1.21834900	2.43182900	-0.61418900
O	0.24360900	5.02796900	0.81250900
C	1.11767100	5.08688100	-0.02415100
C	0.82273600	4.86137700	-1.51790500
C	0.13361900	2.64769400	-1.12291800
C	-0.98931800	1.61488200	-1.28344400
C	-2.35308800	2.19144400	-0.87114500
C	0.25334100	6.13141300	-2.13563100
H	1.70191600	4.51261400	-2.05950900
H	-1.02922900	1.42627200	-2.37219900
H	-2.35460100	2.46359100	0.19001000
H	-3.11822000	1.42702500	-1.03049500
H	-2.60640400	3.08060400	-1.45474900
H	0.00054000	5.95459200	-3.18521200
H	0.98676900	6.94359800	-2.08579200
H	-0.64748600	6.43913800	-1.59625400
O	2.16737000	1.02173100	-3.10622900
O	4.14458700	-0.36238600	-1.81468900
O	3.26586400	-2.24721100	-2.59907500
O	3.37950400	2.86714200	-2.85039000
C	3.12846900	1.73446300	-2.49814300
C	3.86237400	0.99582000	-1.38418900
C	3.11830800	-1.08028300	-2.32650700
C	1.83178900	-0.28759000	-2.54266900
C	5.18195400	1.63485400	-1.01057600
H	5.67863300	1.02527100	-0.25192300

H	5.83427200	1.71955900	-1.88416400
H	5.01010900	2.63448400	-0.60349000
C	0.87715000	-0.95429100	-3.50838900
H	1.35969100	-1.13273300	-4.47374500
H	0.54738700	-1.90853400	-3.09573100
H	0.00070100	-0.31776100	-3.65337900
H	1.33267400	-0.11905500	-1.57948800
H	3.19025200	0.95146900	-0.52067100
O	0.28973500	-1.79949200	1.03871000
C	1.60439800	-1.91761200	1.10112500
C	2.22452200	-3.15018600	0.73624900
C	3.61105100	-3.21741900	0.81898900
C	4.44882100	-2.16539500	1.25792800
C	3.82778300	-0.98333100	1.61505000
C	2.42271000	-0.83685900	1.53726900
C	1.85516900	0.43386600	1.90947600
N	0.60372100	0.75048300	1.83782000
C	0.17782900	2.08699400	2.24281300
C	-0.64233400	2.05586100	3.55754200
C	-1.41234000	0.71727800	3.66463900
N	-2.00669500	0.26118200	2.39896300
C	-3.30743000	0.28778300	2.28726000
C	-4.07025500	-0.25757800	1.20731600
C	-5.44855700	0.05883300	1.12384200
C	-6.24503200	-0.47170900	0.12971100
C	-5.62116200	-1.36502700	-0.78105800
C	-4.28617500	-1.74299600	-0.74020900
C	-3.46647100	-1.16747300	0.28541900
O	-2.20441500	-1.49076300	0.40151900
C	1.38025200	-4.34108000	0.23981500
C	0.35947000	-4.76161600	1.32353400
C	0.63725900	-3.94542800	-1.05843400
C	2.25136000	-5.57061000	-0.08273900
C	5.97108100	-2.37230800	1.28002600

C	6.71401200	-1.16186600	1.87277000
C	6.32226500	-3.60832200	2.13959200
C	6.46779700	-2.59370600	-0.16746800
C	0.29901400	2.15386800	4.76796900
C	-1.61990600	3.24052300	3.54181600
C	-7.73780500	-0.14503500	-0.03399300
C	-8.23867200	0.83073000	1.04568300
C	-7.97456700	0.50399400	-1.41723000
C	-8.56876400	-1.44468700	0.06853800
C	-3.67536300	-2.71485500	-1.76616300
C	-4.71579200	-3.21352000	-2.78717900
C	-3.10721400	-3.95561800	-1.03631600
C	-2.55174700	-1.99858000	-2.55532700
H	4.09329900	-4.13811100	0.51777900
H	4.40509700	-0.13031000	1.95920600
H	2.56117900	1.19191800	2.26571000
H	-0.44522700	2.49027100	1.44357700
H	1.02910400	2.76977300	2.33897500
H	-0.72272500	-0.06933700	3.98973100
H	-2.18896700	0.80586500	4.43446300
H	-3.87794200	0.75006900	3.09909600
H	-5.85320700	0.74509400	1.86147400
H	-6.24208300	-1.77969400	-1.56574600
H	0.87248800	-5.06181400	2.24623500
H	-0.22706900	-5.61977900	0.97131400
H	-0.32997300	-3.94949100	1.55850200
H	-0.03085200	-3.09958700	-0.89148200
H	0.03320100	-4.78828400	-1.41706000
H	1.35764000	-3.68187900	-1.83930100
H	2.80085700	-5.93070000	0.79580100
H	2.97233400	-5.36598600	-0.88270100
H	1.60760900	-6.38925900	-0.42466700
H	5.86519800	-4.52422200	1.75044500
H	6.39769800	-0.95476800	2.90222700

H	6.56065300	-0.25351000	1.27921300
H	7.79199100	-1.35926600	1.89031200
H	5.98125900	-3.47764700	3.17369000
H	7.40806600	-3.76510500	2.15674100
H	6.01105500	-3.48087900	-0.61952100
H	7.55661300	-2.73173600	-0.18389900
H	6.21240500	-1.73973500	-0.80328900
H	1.08020500	1.38453400	4.72469600
H	-0.24859600	2.02193200	5.70947600
H	0.78815600	3.13421500	4.79894100
H	-2.38378300	3.11196300	2.76501100
H	-1.09150300	4.17634000	3.32789500
H	-2.12904900	3.34562200	4.50763000
H	-8.12301700	0.41612400	2.05411000
H	-9.30447300	1.03739600	0.89471700
H	-7.70838600	1.78954600	1.00558500
H	-9.03715500	0.74152100	-1.55366200
H	-7.67643500	-0.15946400	-2.23649400
H	-7.40239600	1.43413600	-1.51579100
H	-8.42595100	-1.92430700	1.04408000
H	-8.29148600	-2.17069500	-0.70357200
H	-9.63762900	-1.22716900	-0.05098500
H	-4.23092900	-3.89836100	-3.49248600
H	-5.14742100	-2.39328300	-3.37357300
H	-5.53547300	-3.76267600	-2.30806200
H	-2.33424200	-3.67986900	-0.31884600
H	-2.67077100	-4.65202200	-1.76334000
H	-3.90531700	-4.48557200	-0.50109200
H	-1.78774100	-1.58921500	-1.89448800
H	-2.96714000	-1.17202900	-3.14673900
H	-2.07460400	-2.70077700	-3.25077300

LLA-TS1

O	-2.30273500	0.45003500	0.54218800
C	-3.32088000	-0.37109400	0.44429300
C	-4.58215900	0.08855400	-0.05911000
C	-5.61835500	-0.83242100	-0.14797800
C	-5.53756600	-2.18810500	0.25752600
C	-4.33357300	-2.60251500	0.79377800
C	-3.22688700	-1.72758300	0.89001600
C	-2.05432300	-2.19022800	1.58258200
N	-0.91187300	-1.57844600	1.60675400
C	0.09962000	-1.95971900	2.58525900
C	0.06977800	-0.97641300	3.79974600
C	-0.55883600	0.38374200	3.40967700
N	-0.09589100	0.99959000	2.15284600
C	0.16339800	2.27376500	2.20360400
C	0.41311600	3.16907400	1.11010600
C	0.73734400	4.51040600	1.42969400
C	0.99876100	5.44411200	0.44764600
C	0.95140900	4.98548000	-0.89350500
C	0.64140200	3.68933300	-1.28294000
C	0.32241300	2.74474200	-0.25027900
O	-0.01084100	1.52011100	-0.55239200
C	-4.76210100	1.55828200	-0.48638500
C	-4.51770500	2.48534900	0.72885200
C	-3.76272300	1.91262400	-1.61125100
C	-6.18254800	1.84213300	-1.01157800
C	-6.76421700	-3.10128100	0.10254200
C	-6.48809500	-4.53212600	0.59782600
C	-7.94635900	-2.53006000	0.91912900
C	-7.16599600	-3.17776300	-1.38840500
C	-0.82335700	-1.56326100	4.90794800
C	1.49870400	-0.78877700	4.32857400
C	1.34362300	6.91402100	0.73521400
C	2.74808800	7.23814900	0.17577700
C	0.30059000	7.83123400	0.05630200
C	1.34559200	7.22433400	2.24272500
C	0.69327900	3.25257500	-2.76119600

C	1.05372900	4.42193600	-3.69769800
C	-0.66865600	2.68409400	-3.22120800
C	1.77935800	2.16224900	-2.92657300
H	-6.56479000	-0.49555800	-0.55263200
H	-4.20411700	-3.61736900	1.15852300
H	-2.16345800	-3.10976100	2.17064700
H	1.07292200	-1.92476000	2.09553400
H	-0.06131200	-2.98679300	2.94180400
H	-1.64081300	0.24942300	3.29785800
H	-0.40640000	1.08264200	4.24276900
H	0.17723700	2.74507700	3.19273200
H	0.77463700	4.78022700	2.48071100
H	1.18167200	5.70742200	-1.66702900
H	-5.23128400	2.26867100	1.53401900
H	-4.64997300	3.53481700	0.43517500
H	-3.50547900	2.36453800	1.11934400
H	-2.73423000	1.80402000	-1.27114500
H	-3.91408300	2.95060800	-1.93514100
H	-3.90793100	1.26282000	-2.48320500
H	-6.95213800	1.65072700	-0.25375400
H	-6.42000900	1.24627300	-1.90124700
H	-6.25933300	2.89809800	-1.29573200
H	-8.22555400	-1.52427400	0.58645200
H	-6.23121100	-4.55159400	1.66359100
H	-5.67168700	-5.00526400	0.03943000
H	-7.38223600	-5.15205000	0.46373800
H	-7.69285800	-2.47035100	1.98426400
H	-8.83159400	-3.17041600	0.81514600
H	-7.41736800	-2.19186500	-1.79454100
H	-8.04378800	-3.82359000	-1.51854400
H	-6.34839700	-3.58812000	-1.99284000
H	-1.82267700	-1.80612400	4.52496700
H	-0.94772200	-0.85595400	5.73750400
H	-0.38368500	-2.48144000	5.31452100

H	2.13133600	-0.27584000	3.59781100
H	1.96581600	-1.75724500	4.54382700
H	1.50113600	-0.19998600	5.25432600
H	3.51107600	6.60525700	0.64435500
H	3.00783200	8.28629200	0.37146500
H	2.80386000	7.08105100	-0.90706000
H	0.53452800	8.88660900	0.24565900
H	-0.70595500	7.63074500	0.44198300
H	0.27574400	7.68753100	-1.02950200
H	2.09281500	6.62855500	2.78027000
H	0.36559600	7.03771300	2.69756200
H	1.58863600	8.28086200	2.40511000
H	1.08221000	4.06135200	-4.73242200
H	2.03990700	4.84618700	-3.47339000
H	0.31277700	5.22937600	-3.65279100
H	-0.92936200	1.78341800	-2.66645900
H	-0.62552400	2.43118100	-4.28880200
H	-1.46734900	3.42241400	-3.08314000
H	1.52936500	1.27738700	-2.34215800
H	2.76154500	2.53643600	-2.61096800
H	1.85499200	1.86187400	-3.98023300
Al	-0.50135000	0.02391300	0.40489300
O	6.63241000	-3.67679000	1.38750000
C	7.43104600	-3.48277500	2.57197300
H	8.25462700	-2.79115100	2.37253100
H	6.81601200	-3.08748400	3.38536200
H	7.81613800	-4.46997300	2.83047300
O	1.13592400	-0.84786600	-0.00366200
O	4.36332300	-1.85796900	-0.67675800
O	6.18883700	-1.45722900	1.35014800
C	6.06674200	-2.56816900	0.88380300
C	5.30813300	-2.90580900	-0.39873800
C	3.35153700	-1.72044500	0.20790800
C	2.49359400	-0.51110900	-0.15989000

C	2.95656000	0.66202400	0.70698000
C	6.25266400	-2.97571800	-1.59193200
H	4.77031000	-3.84732100	-0.26499700
H	2.68651600	-0.25649700	-1.20773300
H	4.04122000	0.77896600	0.60762000
H	2.72436200	0.47738800	1.75705800
H	2.47541400	1.58652200	0.39034700
H	6.77978600	-2.02413800	-1.71711800
H	5.67534700	-3.19160500	-2.49407600
H	6.98728200	-3.77200400	-1.43896900
O	0.89808100	-1.14243000	-2.54027000
O	1.69314800	-3.58919300	-1.39888200
O	3.29601700	-3.83034300	-2.91343200
O	-0.86954600	-1.01939300	-1.18648800
C	0.24496700	-1.58428800	-1.44360700
C	0.36114700	-3.07287500	-1.14196400
C	2.33476200	-3.21176900	-2.51624200
C	1.84602300	-1.97126000	-3.25378100
C	-0.70485800	-3.87457700	-1.91869000
H	-0.63177300	-4.93686100	-1.66823300
H	-1.70912100	-3.53318500	-1.65407700
H	-0.59061300	-3.75565900	-2.99903300
C	1.25218900	-2.33350800	-4.61341100
H	0.36387000	-2.96206400	-4.49925700
H	0.96606800	-1.41681900	-5.13669700
H	1.99522200	-2.87088300	-5.20934800
H	2.73266600	-1.34662000	-3.39272300
H	0.23782600	-3.19054800	-0.06993200
O	3.18978100	-2.43988200	1.16968800

LLA-IM2

Al	-0.13098500	-1.54934700	-0.21533100
O	1.31659600	-1.09815000	-1.17605100
C	2.61648000	-1.31577100	-1.07005900
C	3.53435600	-0.50856500	-1.80776700
C	4.89056200	-0.78329200	-1.66075000
C	5.42576100	-1.80134000	-0.83716100
C	4.51862100	-2.56769100	-0.12867400
C	3.12722100	-2.34137800	-0.22734400
C	2.25402600	-3.16917600	0.56407000
N	0.96654200	-3.06256000	0.62686000
C	0.20498100	-3.97323800	1.47955400
C	-0.61951600	-4.99278500	0.64472200
C	-0.96592700	-4.38885600	-0.73706800
N	-1.41232800	-2.98805400	-0.70119200
C	-2.66578300	-2.74333000	-0.97339700
C	-3.25653000	-1.46280400	-1.20864200
C	-4.66204400	-1.40012800	-1.37335900
C	-5.28796500	-0.22656500	-1.73787800
C	-4.44811900	0.88478800	-2.00652600
C	-3.06407800	0.88974400	-1.89162600
C	-2.43939400	-0.30838400	-1.40898700
O	-1.14936300	-0.35485500	-1.18083500
C	3.02888300	0.62672900	-2.72045500
C	2.14462300	0.03397700	-3.84277700
C	2.22196000	1.65474300	-1.89051600
C	4.18718000	1.38831100	-3.39370000
C	6.94849900	-1.99946700	-0.76677800
C	7.34088600	-3.14327000	0.18534000
C	7.49045100	-2.33716200	-2.17478900
C	7.61665200	-0.70057300	-0.26031700
C	0.21245600	-6.26071900	0.39246700
C	-1.90115800	-5.33442000	1.41906000
C	-6.81025300	-0.07863100	-1.88390900
C	-7.55245000	-1.38447500	-1.54893200
C	-7.31131600	1.02176900	-0.92032600
C	-7.15915800	0.31687000	-3.33717200
C	-2.22396800	2.12448900	-2.27105400
C	-3.09126400	3.26984600	-2.82807900

C	-1.20964200	1.72818500	-3.36940100
C	-1.47270000	2.66618900	-1.03545200
H	5.59509800	-0.17262100	-2.21107700
H	4.85301600	-3.36329900	0.53000700
H	2.74085900	-3.94223500	1.16896400
H	-0.47629700	-3.35800100	2.07047000
H	0.86353900	-4.50681600	2.17732200
H	-0.07556300	-4.41782300	-1.37482000
H	-1.72875100	-5.01201800	-1.21947500
H	-3.33234300	-3.60303000	-1.08901700
H	-5.22998700	-2.30751300	-1.19362200
H	-4.93208000	1.79690700	-2.33241500
H	2.72523600	-0.65725400	-4.46678900
H	1.76664500	0.83511600	-4.49019900
H	1.29003500	-0.50614400	-3.43276200
H	1.33886900	1.20243200	-1.43972100
H	1.88887600	2.47662500	-2.53732800
H	2.83773200	2.08240700	-1.09216300
H	4.79656000	0.73849500	-4.03335800
H	4.84648900	1.86786500	-2.66020200
H	3.77599000	2.17938000	-4.03148300
H	7.27842500	-1.54060700	-2.89639200
H	6.92057800	-4.10365300	-0.13611000
H	7.00976000	-2.94952400	1.21241500
H	8.43133600	-3.25206800	0.20606900
H	7.04029500	-3.26138800	-2.55621400
H	8.57852500	-2.47571100	-2.14449300
H	7.41496100	0.14899700	-0.92184200
H	8.70536300	-0.82563600	-0.20514600
H	7.25267600	-0.43749300	0.73977700
H	1.17978700	-6.01643600	-0.06470100
H	-0.31022500	-6.95119500	-0.28100800
H	0.40483800	-6.79240200	1.33148700
H	-2.53929000	-4.45383900	1.54995300
H	-1.66070000	-5.70767300	2.42182000
H	-2.47554700	-6.11273700	0.90160900
H	-7.26632100	-2.20019900	-2.22345600
H	-8.63323600	-1.23524600	-1.65394000
H	-7.36166300	-1.70730400	-0.51881200
H	-8.39799000	1.14476500	-1.00966700
H	-6.85017000	1.99254700	-1.13347000
H	-7.08199500	0.76385400	0.12029700
H	-6.81990100	-0.45011300	-4.04347900
H	-6.69468600	1.26592300	-3.62699200
H	-8.24420400	0.43097500	-3.45372300
H	-2.44336900	4.10990400	-3.10465400
H	-3.80988400	3.64339600	-2.08843200
H	-3.64464700	2.97303400	-3.72716800
H	-0.53775000	0.94377400	-3.02271000
H	-0.60437700	2.59786700	-3.65499400
H	-1.72898200	1.37081500	-4.26747100
H	-0.82128700	1.91908800	-0.58806500
H	-2.18043300	2.99680000	-0.26585400
H	-0.86025500	3.53127900	-1.32173500
O	2.64722800	6.59228100	1.01104000
C	2.91779700	6.33318200	-0.37794100
H	3.11440900	5.27042400	-0.54194900
H	2.10058400	6.67935000	-1.02087800
H	3.81518900	6.91103200	-0.60768100
O	-0.17876000	1.34270300	1.64766400
O	0.37985000	3.87823100	1.53104700
O	2.53071200	3.34875600	1.12212400
O	1.62580700	6.00882200	2.88232300
C	1.68932900	5.92102800	1.67809000
C	0.61882900	5.14702600	0.88688900
C	1.43278600	3.04404600	1.54782600
C	1.09628200	1.70002500	2.19027900
C	2.19900900	0.67957800	1.94223000

H	0.98791900	1.88221600	3.26811900
H	2.31872300	0.49453100	0.87371200
H	1.94858800	-0.25849700	2.43870800
H	3.14665000	1.05840600	2.33621700
O	-0.55576700	0.03338300	3.48447400
O	-2.98045500	-0.83656300	2.37552300
O	-2.87521400	-2.71743800	3.54032300
O	-0.35339400	-0.95175500	1.44636100
C	-0.79350800	0.16257900	2.08377600
C	-2.31148600	0.37979100	1.92249200
C	-2.38984500	-1.63478300	3.28112000
C	-1.12301700	-1.16432600	4.00847200
C	-2.85939900	1.58457700	2.66460600
H	-3.94471500	1.64236300	2.53295700
H	-2.63129800	1.52112100	3.73229300
H	-2.40114000	2.49495200	2.26910700
C	-1.42223100	-0.93434100	5.48737000
H	-2.17485100	-0.14923500	5.61455000
H	-1.79064600	-1.85739200	5.94308800
H	-0.50637500	-0.62193000	5.99882000
H	-0.40443300	-1.98606900	3.90199100
H	-2.55581800	0.44398700	0.86456400
H	0.92558700	4.94394900	-0.13921400
C	-0.69687200	5.91003100	0.92398700
H	-1.47245800	5.34334600	0.40167100
H	-1.00206600	6.07133600	1.96158100
H	-0.58204900	6.88410900	0.43568600

LLA-IM3

Al	0.82626000	-0.15892900	-0.48800800
O	2.44123800	0.55171600	-0.84096900
C	3.69139200	0.12773200	-0.79832800
C	4.76496100	1.07016000	-0.81104000
C	6.06191800	0.56789900	-0.79072800
C	6.39786000	-0.80619000	-0.75229900
C	5.34456900	-1.70089900	-0.72483500
C	4.00182700	-1.25943700	-0.74380800
C	2.96683600	-2.25554400	-0.67682800
N	1.69507200	-2.01963400	-0.66342700
C	0.75960300	-3.13219900	-0.54502700
C	-0.07423900	-3.34191900	-1.83996700
C	-0.15337300	-2.02039500	-2.63721500
N	-0.47028100	-0.83364500	-1.82893900
C	-1.63316000	-0.26775700	-2.03012500
C	-2.07002400	0.99430700	-1.52862800
C	-3.40804000	1.37796300	-1.79053500
C	-3.87358800	2.62701900	-1.43692600
C	-2.93464400	3.51915900	-0.85507400
C	-1.61078300	3.21010600	-0.57193100
C	-1.16318300	1.88246000	-0.87727900
O	0.05868300	1.50283100	-0.60139800
C	4.48090000	2.58436900	-0.83185100
C	3.71991700	2.95382000	-2.12719200
C	3.64729900	2.97691900	0.41251300
C	5.77557600	3.41968500	-0.80085900
C	7.87618600	-1.22664700	-0.72912200
C	8.04325200	-2.75618300	-0.69214600
C	8.58565400	-0.69476900	-1.99547800
C	8.56111600	-0.63671800	0.52509600
C	0.59764800	-4.38812100	-2.74292300
C	-1.48174500	-3.80226600	-1.43250300
C	-5.34044400	3.05507400	-1.59172600
C	-6.18638600	1.96974400	-2.28181400
C	-5.93578600	3.30334300	-0.18583700
C	-5.43357000	4.34978800	-2.42911800
C	-0.65168200	4.23379700	0.06362500
C	-1.32731100	5.59709600	0.30401300
C	0.54535800	4.46581600	-0.88711300
C	-0.14881100	3.71597500	1.43332800

H	6.88110500	1.27601500	-0.79908400
H	5.52103000	-2.77135700	-0.68131400
H	3.30315800	-3.29660000	-0.61371900
H	0.08231700	-2.87858800	0.27386000
H	1.27736300	-4.06134100	-0.27274100
H	0.81554600	-1.83398000	-3.11353500
H	-0.89137800	-2.13294600	-3.44074000
H	-2.33966300	-0.78755500	-2.68038100
H	-4.05785100	0.63971200	-2.24413600
H	-3.29195700	4.50785700	-0.59421800
H	4.33126300	2.72558400	-3.00957400
H	3.49510300	4.02754100	-2.14225500
H	2.78016600	2.40598000	-2.20809000
H	2.68110400	2.47265600	0.42396700
H	3.46826100	4.05899500	0.41962400
H	4.18609700	2.71852000	1.33274100
H	6.41505200	3.22661700	-1.67067800
H	6.36335400	3.23718800	0.10681500
H	5.51806100	4.48500500	-0.81579400
H	8.54016700	0.39780800	-2.06124400
H	7.59818000	-3.23586000	-1.57207200
H	7.58888200	-3.19364400	0.20455400
H	9.10869000	-3.01366100	-0.68020900
H	8.12530900	-1.10314900	-2.90306300
H	9.64466800	-0.98222200	-1.99187300
H	8.51598500	0.45775800	0.53852600
H	9.61947000	-0.92511800	0.55629300
H	8.08105800	-1.00131100	1.44071600
H	1.63746200	-4.11433300	-2.96084700
H	0.06983300	-4.48458700	-3.69978700
H	0.60146100	-5.37346500	-2.26217100
H	-1.97275600	-3.03054000	-0.83160000
H	-1.42675800	-4.71815900	-0.82972900
H	-2.10308300	-4.00965500	-2.31150600

H	-5.82038000	1.75036900	-3.29204800
H	-7.22443500	2.31079100	-2.37290500
H	-6.19290400	1.03569100	-1.70918000
H	-6.98400700	3.62042400	-0.26149800
H	-5.38988500	4.08518700	0.35453600
H	-5.89676500	2.38831900	0.41594800
H	-5.01858400	4.19784700	-3.43278500
H	-4.89022000	5.18070400	-1.96573300
H	-6.48001000	4.66165400	-2.53779600
H	-0.60193800	6.28586100	0.75275300
H	-2.17653500	5.52207400	0.99386000
H	-1.68028900	6.05406300	-0.62844000
H	1.09103800	3.54079500	-1.06652200
H	1.23839600	5.19766600	-0.45330400
H	0.20156600	4.85881000	-1.85230200
H	0.28820000	2.72105600	1.35169600
H	-0.97662500	3.66706600	2.15011700
H	0.60762400	4.40002500	1.83942900
O	-4.97655300	-3.79343800	-1.91194800
C	-4.94740400	-5.19555800	-1.59028500
H	-3.94207600	-5.51122600	-1.29354000
H	-5.67028900	-5.45846200	-0.81351500
H	-5.22212700	-5.70545500	-2.51516000
O	-1.68541800	-0.60036500	1.40173600
O	-3.65810800	-2.29436000	1.12905000
O	-5.15879600	-0.58238800	1.22865300
O	-4.40210900	-1.71852100	-1.44088700
C	-4.61829600	-2.83435800	-1.03110000
C	-4.53938600	-3.21135500	0.45777600
C	-4.04480800	-1.02052900	1.39661600
C	-2.90878200	-0.21895700	2.04289500
C	-3.19728700	1.27312900	1.95572800
C	-5.92579400	-3.35518400	1.10146500
H	-4.00360300	-4.15874100	0.55550300

H	-2.89276000	-0.51228500	3.09640800
H	-3.33825500	1.57978100	0.92196800
H	-2.36405500	1.82303600	2.38984900
H	-4.11293100	1.50255100	2.50916200
H	-5.80396100	-3.44844400	2.18519600
H	-6.41887900	-4.26147700	0.73801000
H	-6.55814500	-2.49414100	0.89221100
O	-0.46869200	0.51153900	3.00370300
O	0.98425300	-1.74422800	3.72288800
O	2.56163300	-0.50358200	4.64890100
O	0.55030000	-0.55558700	1.22710500
C	-0.46153000	-0.60079400	2.11125200
C	-0.26933300	-1.86950000	2.98659300
C	1.51776000	-0.54657100	4.03445600
C	0.81307900	0.73710700	3.58652000
C	-1.35265100	-2.22649000	3.99079900
H	-1.00103600	-3.05861400	4.60983000
H	-1.57351000	-1.38084900	4.64966700
H	-2.26525700	-2.54542800	3.47879900
C	0.62463300	1.69524700	4.75505200
H	-0.04014300	1.25928300	5.50869300
H	1.59019200	1.91417700	5.21693400
H	0.17913000	2.62653800	4.39249300
H	1.47908600	1.18854900	2.83951400
H	-0.10052900	-2.71250300	2.31288600

LLA-TS2

O	4.18101000	7.16707200	-1.35117900
C	3.42429900	7.32558900	-2.56418500
H	2.46279800	7.81882900	-2.38531600
H	4.03648400	7.96470100	-3.20312000
H	3.27132900	6.36077700	-3.05797300
O	2.35590100	1.19869700	-0.25388700
O	2.15955300	4.69591100	0.34763900
O	3.31477600	3.69615200	-1.32325100
O	4.46247900	6.14929900	0.59047800
C	3.73230700	6.39661400	-0.34033400
C	2.26414400	5.94517600	-0.35724200
C	2.69517000	3.63896300	-0.28274400
C	2.46689500	2.38452700	0.55014400
C	3.67100300	2.16870800	1.46112200
C	1.38497800	6.94143000	0.38681000
H	1.89550700	5.78121800	-1.37018600
H	1.55249100	2.49019800	1.13450300
H	4.57388600	2.02141700	0.86082600
H	3.50894600	1.28472300	2.08315900
H	3.81470900	3.04228600	2.10448700
H	1.74545500	7.06769900	1.41224900
H	0.35964500	6.56309900	0.40429000
H	1.40398000	7.91578000	-0.11304700
O	-0.25727400	1.03068900	0.59050000
O	0.26283100	3.08726900	-1.24379100
O	-0.97607000	4.38667300	0.03681400
O	1.09123400	-0.38609100	-1.10773900
C	1.24840000	0.82617300	-0.92708500
C	0.47641000	1.78593000	-1.82343500
C	-0.67029500	3.25561300	-0.26573500
C	-1.26300500	1.99720900	0.39895000
C	1.19973800	1.98491000	-3.14985200
H	0.59698000	2.63218200	-3.79447000
H	2.17894400	2.44192200	-2.99396000
H	1.32501700	1.01582500	-3.64224800
C	-1.93326900	2.40740700	1.70995200
H	-2.38884400	1.53400700	2.17541700
H	-1.19504300	2.83435600	2.39624000
H	-2.70971700	3.15611400	1.53188800
H	-2.04072200	1.62924300	-0.29196000
H	-0.48560200	1.30730800	-1.99626600
Al	-0.35971600	-0.85182200	0.60631800

O	0.15157900	-2.55259400	0.16976000
C	1.35350500	-3.07850300	0.06933900
C	1.60273400	-4.15208800	-0.84340600
C	2.87337000	-4.71571100	-0.84538700
C	3.94749000	-4.30224300	-0.01944600
C	3.69900900	-3.24531800	0.83435100
C	2.43143300	-2.62068700	0.88429600
C	2.23640600	-1.56684800	1.83691900
N	1.18289000	-0.81457500	1.92094800
C	0.99402100	0.01899900	3.10316700
C	0.07202500	-0.69549000	4.14247800
C	-0.88680100	-1.69120700	3.43754900
N	-1.51761000	-1.17800900	2.21622300
C	-2.80710000	-1.01934600	2.20819800
C	-3.62178600	-0.67557400	1.07758200
C	-4.98045900	-0.36477900	1.32761100
C	-5.86192300	-0.10539300	0.29839500
C	-5.35574100	-0.25224500	-1.01746000
C	-4.04721600	-0.58999200	-1.33841100
C	-3.11303700	-0.73272300	-0.25728300
O	-1.84133400	-0.94981500	-0.49191900
C	0.52061800	-4.59322000	-1.84758500
C	-0.76763600	-5.04394300	-1.11945500
C	0.21110700	-3.39910100	-2.78236000
C	0.98635300	-5.76931800	-2.72795800
C	5.30354600	-5.01862200	-0.12049900
C	6.34129100	-4.43440800	0.85452600
C	5.12331200	-6.51810400	0.20981700
C	5.85888300	-4.87610200	-1.55637000
C	0.92666800	-1.51411700	5.12419100
C	-0.72933000	0.37137100	4.90232700
C	-7.33233000	0.28809400	0.50803900
C	-7.69471600	0.39697200	1.99982000
C	-7.59550000	1.66053200	-0.15333300
C	-8.25362100	-0.77327000	-0.13618900
C	-3.63118800	-0.89423200	-2.79118200
C	-4.76734200	-0.61643700	-3.79462100
C	-3.27940700	-2.39886300	-2.87260100
C	-2.40942600	-0.05787700	-3.23371200
H	3.06775800	-5.52736300	-1.53545900
H	4.47439700	-2.86055000	1.48990500
H	3.03859700	-1.41487000	2.56821800
H	0.52788400	0.94727300	2.77242500
H	1.95416300	0.26462100	3.57605000
H	-0.32441800	-2.58427300	3.14556300
H	-1.65378100	-2.00895000	4.15560100
H	-3.34748400	-1.18161100	3.14679500
H	-5.30305800	-0.32899000	2.36375700
H	-6.05498800	-0.10445600	-1.83076500
H	-0.56019300	-5.89228600	-0.45444300
H	-1.51770200	-5.36858700	-1.85213100
H	-1.19239000	-4.23516700	-0.52465400
H	-0.14560900	-2.53555200	-2.22030700
H	-0.55515900	-3.68023500	-3.51526000
H	1.11192500	-3.09955200	-3.33192800
H	1.22593700	-6.66031100	-2.13441500
H	1.86352200	-5.51320300	-3.33390700
H	0.18190700	-6.04275200	-3.42064600
H	4.42258800	-7.00651700	-0.47618000
H	6.01655400	-4.52713000	1.89776900
H	6.54134500	-3.37611400	0.64987100
H	7.29033600	-4.97404000	0.75557400
H	4.73976000	-6.65100600	1.22848300
H	6.08220600	-7.04661700	0.13542300
H	5.18548400	-5.31547000	-2.30048000
H	6.82791400	-5.38340900	-1.64625100
H	6.00083100	-3.82078400	-1.81712000
H	1.58104000	-2.21652700	4.59325900

H	0.29624700	-2.09893700	5.80536900
H	1.55753700	-0.85671600	5.73378100
H	-1.42702200	0.89149600	4.23732900
H	-0.06123900	1.12436100	5.33829600
H	-1.30521900	-0.07699400	5.72109300
H	-7.56591200	-0.55886700	2.52121800
H	-8.74526200	0.69062400	2.10710000
H	-7.08669300	1.15243700	2.51147500
H	-8.64285100	1.95822000	-0.01723200
H	-7.39488500	1.64026700	-1.23026600
H	-6.96070600	2.43687000	0.29005700
H	-8.09167200	-1.75843500	0.31692500
H	-8.07773800	-0.86867000	-1.21334200
H	-9.30779300	-0.50281300	0.00394400
H	-4.42367000	-0.86172600	-4.80599000
H	-5.06735800	0.43883000	-3.79493700
H	-5.65596100	-1.22732900	-3.59831400
H	-2.48826400	-2.65615400	-2.16683900
H	-2.93498700	-2.65685200	-3.88207200
H	-4.15879400	-3.01429200	-2.64666900
H	-1.52218700	-0.32967600	-2.66381700
H	-2.60261400	1.01530900	-3.10586000
H	-2.20061200	-0.23325500	-4.29652400

LLS-IM4

Al	-2.14579500	0.10691800	0.49920600
O	4.98086600	-2.85905200	-0.59225700
O	-0.77369700	-0.52366600	-0.38564700
O	2.19457500	-1.25237000	-2.12917000
O	1.46450300	-2.14202500	-0.18481000
O	4.30153500	-0.70586100	-0.40143800
C	4.21891800	-1.79521600	-0.92184800
C	3.30805700	-2.15342000	-2.09197800
C	1.35545800	-1.31170800	-1.06226200
C	0.34606900	-0.16224500	-1.10373100
C	1.08391500	1.07738500	-0.55944200
C	4.06507700	-1.99366800	-3.40593600
H	2.95273600	-3.17893400	-1.95897900
H	0.10749600	0.02013000	-2.16369900
H	1.31643900	0.93125800	0.50149500
H	0.45684300	1.96399200	-0.66445500
H	2.02248100	1.24323400	-1.09785400
H	3.40968900	-2.24808700	-4.24426600
H	4.93670600	-2.65398300	-3.41767800
H	4.40646300	-0.96048400	-3.52228800
O	-3.82130800	-0.26083200	-0.04863200
C	-4.61368800	-1.31613700	-0.04642000
C	-5.83171900	-1.29339700	-0.79341100
C	-6.64265400	-2.42228300	-0.73673500
C	-6.34629400	-3.59461700	-0.00201800
C	-5.15499300	-3.60141900	0.70045000
C	-4.28668300	-2.48680600	0.69155800
C	-3.05994400	-2.58343600	1.44059500
N	-2.16190600	-1.65960500	1.54183100
C	-0.95423400	-1.91272200	2.32641600
C	-0.89939600	-1.02061300	3.60792400
C	-1.89327500	0.15969600	3.49866900
N	-1.75857000	0.95515300	2.26948900
C	-1.35893300	2.19177700	2.40270000
C	-1.24658600	3.18167800	1.37893900
C	-0.66084700	4.42652500	1.71954400
C	-0.54061100	5.44001500	0.79196800
C	-1.05578600	5.18088600	-0.50550800
C	-1.65942900	3.99641800	-0.90387100
C	-1.75184100	2.94225700	0.06522100
O	-2.29266600	1.79387600	-0.23937600
C	-6.20682500	-0.06481000	-1.64487600
C	-6.33605100	1.18709700	-0.74466800
C	-5.12142200	0.16402600	-2.72439000

C	-7.55200000	-0.25087700	-2.37347300
C	-7.33056100	-4.77536700	-0.02463900
C	-6.83372500	-5.96096300	0.82192200
C	-8.69659600	-4.32044100	0.53847200
C	-7.51713800	-5.26766800	-1.47829800
C	-1.32075600	-1.84837400	4.83494900
C	0.53144500	-0.49354300	3.79100800
C	0.11029400	6.79977500	1.09060300
C	0.60312000	6.89853900	2.54532100
C	1.32456000	7.00611600	0.15586300
C	-0.91550000	7.93136900	0.85146800
C	-2.21040200	3.79965600	-2.32837200
C	-1.97629100	5.03464600	-3.21900000
C	-3.73509600	3.55310300	-2.25240100
C	-1.51821200	2.59527400	-3.01148200
H	-7.56865600	-2.41209400	-1.29812700
H	-4.85231600	-4.46843100	1.27962300
H	-2.88549700	-3.53516000	1.95547500
H	-0.10888900	-1.70073900	1.66727500
H	-0.89204100	-2.97182500	2.60881800
H	-2.91741700	-0.22808000	3.52481100
H	-1.76788100	0.80761200	4.37556400
H	-1.08861500	2.52380600	3.40990200
H	-0.29883600	4.55005500	2.73560700
H	-0.96710000	5.97522900	-1.23624000
H	-7.12903100	1.04488800	0.00064800
H	-6.59869200	2.06274800	-1.35168400
H	-5.40346100	1.39999000	-0.22089400
H	-4.14363700	0.34739500	-2.27737600
H	-5.38260100	1.02997200	-3.34500800
H	-5.04363600	-0.71080100	-3.38200100
H	-8.38507200	-0.39407700	-1.67457500
H	-7.53369400	-1.10075000	-3.06630500
H	-7.77071500	0.64684500	-2.96324300
H	-9.12721000	-3.50051600	-0.04669900
H	-6.70919600	-5.68515400	1.87580700
H	-5.87753400	-6.35126100	0.45398700
H	-7.56182200	-6.77933300	0.77969100
H	-8.59710200	-3.97427600	1.57414100
H	-9.41442300	-5.15040800	0.52423800
H	-7.91331900	-4.48072000	-2.12933500
H	-8.21999000	-6.10978800	-1.51167100
H	-6.56350500	-5.60288600	-1.90283200
H	-2.31456900	-2.29158600	4.69276800
H	-1.35496000	-1.23168700	5.74183500
H	-0.61060900	-2.66408100	5.01324200
H	0.82156900	0.16032400	2.96070700
H	1.24861500	-1.32231000	3.82276100
H	0.63015000	0.07236400	4.72605600
H	-0.21919600	6.78805600	3.26215700
H	1.06016000	7.87988200	2.71731700
H	1.36032100	6.13838600	2.77097600
H	1.80188200	7.97414600	0.35412200
H	1.03513000	6.99093900	-0.90066000
H	2.07311000	6.21960900	0.30793300
H	-1.78644300	7.81511500	1.50730900
H	-1.27637200	7.94508500	-0.18280000
H	-0.46278200	8.90971400	1.05647400
H	-2.38815200	4.84420900	-4.21675700
H	-0.90978000	5.25926100	-3.34197500
H	-2.47406500	5.92932900	-2.82610700
H	-3.96201600	2.67378200	-1.65054900
H	-4.14438700	3.39747000	-3.25860300
H	-4.24576700	4.41800300	-1.81055800
H	-1.69004500	1.67056900	-2.46006400
H	-0.43621100	2.76045700	-3.08647500
H	-1.90925300	2.46657600	-4.02875800
C	6.01767400	-2.60628400	0.36956400

H 5.58813000 -2.13384800 1.25698300
C 7.05282300 -1.66611200 -0.24045600
O 7.23236600 -1.49372500 -1.42394900
O 7.78253000 -1.09723700 0.74055600
C 6.65800600 -3.94782400 0.70596300
H 5.90856100 -4.61781900 1.13758400
H 7.07081700 -4.40782800 -0.19690100
H 7.46854800 -3.80217000 1.42487100
C 8.93373500 -0.35126100 0.31225600
H 8.64320200 0.35267500 -0.47224600
C 9.96788800 -1.32350800 -0.25916200
O 9.95710200 -2.52393500 -0.10236700
O 10.91407300 -0.64373200 -0.92382400
C 11.98143300 -1.44177300 -1.47746200
H 11.58359300 -2.16197400 -2.19759400
H 12.64800700 -0.73481700 -1.97259800
H 12.51136100 -1.97662600 -0.68414200
C 9.47583200 0.37733800 1.53572700
H 8.70974500 1.04708600 1.93737000
H 9.76051300 -0.33909300 2.31315100
H 10.35297700 0.96861900 1.25812200

DLA-IM1

Al -2.14579500 0.10691800 0.49920600
O 4.98086600 -2.85905200 -0.59225700
O -0.77369700 -0.52366600 -0.38564700
O 2.19457500 -1.25237000 -2.12917000
O 1.46450300 -2.14202500 -0.18481000
O 4.30153500 -0.70586100 -0.40143800
C 4.21891800 -1.79521600 -0.92184800
C 3.30805700 -2.15342000 -2.09197800
C 1.35545800 -1.31170800 -1.06226200
C 0.34606900 -0.16224500 -1.10373100
C 1.08391500 1.07738500 -0.55944200
C 4.06507700 -1.99366800 -3.40593600
H 2.95273600 -3.17893400 -1.95897900
H 0.10749600 0.02013000 -2.16369900
H 1.31643900 0.93125800 0.50149500
H 0.45684300 1.96399200 -0.66445500
H 2.02248100 1.24323400 -1.09785400
H 3.40968900 -2.24808700 -4.24426600
H 4.93670600 -2.65398300 -3.41767800
H 4.40646300 -0.96048400 -3.52228800
O -3.82130800 -0.26083200 -0.04863200
C -4.61368800 -1.31613700 -0.04642000
C -5.83171900 -1.29339700 -0.79341100
C -6.64265400 -2.42228300 -0.73673500
C -6.34629400 -3.59461700 -0.00201800
C -5.15499300 -3.60141900 0.70045000
C -4.28668300 -2.48680600 0.69155800
C -3.05994400 -2.58343600 1.44059500
N -2.16190600 -1.65960500 1.54183100
C -0.95423400 -1.91272200 2.32641600
C -0.89939600 -1.02061300 3.60792400
C -1.89327500 0.15969600 3.49866900
N -1.75857000 0.95515300 2.26948900
C -1.35893300 2.19177700 2.40270000
C -1.24658600 3.18167800 1.37893900
C -0.66084700 4.42652500 1.71954400
C -0.54061100 5.44001500 0.79196800
C -1.05578600 5.18088600 -0.50550800
C -1.65942900 3.99641800 -0.90387100
C -1.75184100 2.94225700 0.06522100
O -2.29266600 1.79387600 -0.23937600
C -6.20682500 -0.06481000 -1.64487600
C -6.33605100 1.18709700 -0.74466800
C -5.12142200 0.16402600 -2.72439000
C -7.55200000 -0.25087700 -2.37347300
C -7.33056100 -4.77536700 -0.02463900

C	-6.83372500	-5.96096300	0.82192200
C	-8.69659600	-4.32044100	0.53847200
C	-7.51713800	-5.26766800	-1.47829800
C	-1.32075600	-1.84837400	4.83494900
C	0.53144500	-0.49354300	3.79100800
C	0.11029400	6.79977500	1.09060300
C	0.60312000	6.89853900	2.54532100
C	1.32456000	7.00611600	0.15586300
C	-0.91550000	7.93136900	0.85146800
C	-2.21040200	3.79965600	-2.32837200
C	-1.97629100	5.03464600	-3.21900000
C	-3.73509600	3.55310300	-2.25240100
C	-1.51821200	2.59527400	-3.01148200
H	-7.56865600	-2.41209400	-1.29812700
H	-4.85231600	-4.46843100	1.27962300
H	-2.88549700	-3.53516000	1.95547500
H	-0.10888900	-1.70073900	1.66727500
H	-0.89204100	-2.97182500	2.60881800
H	-2.91741700	-0.22808000	3.52481100
H	-1.76788100	0.80761200	4.37556400
H	-1.08861500	2.52380600	3.40990200
H	-0.29883600	4.55005500	2.73560700
H	-0.96710000	5.97522900	-1.23624000
H	-7.12903100	1.04488800	0.00064800
H	-6.59869200	2.06274800	-1.35168400
H	-5.40346100	1.39999000	-0.22089400
H	-4.14363700	0.34739500	-2.27737600
H	-5.38260100	1.02997200	-3.34500800
H	-5.04363600	-0.71080100	-3.38200100
H	-8.38507200	-0.39407700	-1.67457500
H	-7.53369400	-1.10075000	-3.06630500
H	-7.77071500	0.64684500	-2.96324300
H	-9.12721000	-3.50051600	-0.04669900
H	-6.70919600	-5.68515400	1.87580700
H	-5.87753400	-6.35126100	0.45398700
H	-7.56182200	-6.77933300	0.77969100
H	-8.59710200	-3.97427600	1.57414100
H	-9.41442300	-5.15040800	0.52423800
H	-7.91331900	-4.48072000	-2.12933500
H	-8.21999000	-6.10978800	-1.51167100
H	-6.56350500	-5.60288600	-1.90283200
H	-2.31456900	-2.29158600	4.69276800
H	-1.35496000	-1.23168700	5.74183500
H	-0.61060900	-2.66408100	5.01324200
H	0.82156900	0.16032400	2.96070700
H	1.24861500	-1.32231000	3.82276100
H	0.63015000	0.07236400	4.72605600
H	-0.21919600	6.78805600	3.26215700
H	1.06016000	7.87988200	2.71731700
H	1.36032100	6.13838600	2.77097600
H	1.80188200	7.97414600	0.35412200
H	1.03513000	6.99093900	-0.90066000
H	2.07311000	6.21960900	0.30793300
H	-1.78644300	7.81511500	1.50730900
H	-1.27637200	7.94508500	-0.18280000
H	-0.46278200	8.90971400	1.05647400
H	-2.38815200	4.84420900	-4.21675700
H	-0.90978000	5.25926100	-3.34197500
H	-2.47406500	5.92932900	-2.82610700
H	-3.96201600	2.67378200	-1.65054900
H	-4.14438700	3.39747000	-3.25860300
H	-4.24576700	4.41800300	-1.81055800
H	-1.69004500	1.67056900	-2.46006400
H	-0.43621100	2.76045700	-3.08647500
H	-1.90925300	2.46657600	-4.02875800
C	6.01767400	-2.60628400	0.36956400
H	5.58813000	-2.13384800	1.25698300
C	7.05282300	-1.66611200	-0.24045600

O	7.23236600	-1.49372500	-1.42394900
O	7.78253000	-1.09723700	0.74055600
C	6.65800600	-3.94782400	0.70596300
H	5.90856100	-4.61781900	1.13758400
H	7.07081700	-4.40782800	-0.19690100
H	7.46854800	-3.80217000	1.42487100
C	8.93373500	-0.35126100	0.31225600
H	8.64320200	0.35267500	-0.47224600
C	9.96788800	-1.32350800	-0.25916200
O	9.95710200	-2.52393500	-0.10236700
O	10.91407300	-0.64373200	-0.92382400
C	11.98143300	-1.44177300	-1.47746200
H	11.58359300	-2.16197400	-2.19759400
H	12.64800700	-0.73481700	-1.97259800
H	12.51136100	-1.97662600	-0.68414200
C	9.47583200	0.37733800	1.53572700
H	8.70974500	1.04708600	1.93737000
H	9.76051300	-0.33909300	2.31315100
H	10.35297700	0.96861900	1.25812200

DLA-TS1

O	-2.30273500	0.45003500	0.54218800
C	-3.32088000	-0.37109400	0.44429300
C	-4.58215900	0.08855400	-0.05911000
C	-5.61835500	-0.83242100	-0.14797800
C	-5.53756600	-2.18810500	0.25752600
C	-4.33357300	-2.60251500	0.79377800
C	-3.22688700	-1.72758300	0.89001600
C	-2.05432300	-2.19022800	1.58258200
N	-0.91187300	-1.57844600	1.60675400
C	0.09962000	-1.95971900	2.58525900
C	0.06977800	-0.97641300	3.79974600
C	-0.55883600	0.38374200	3.40967700
N	-0.09589100	0.99959000	2.15284600
C	0.16339800	2.27376500	2.20360400
C	0.41311600	3.16907400	1.11010600
C	0.73734400	4.51040600	1.42969400
C	0.99876100	5.44411200	0.44764600
C	0.95140900	4.98548000	-0.89350500
C	0.64140200	3.68933300	-1.28294000
C	0.32241300	2.74474200	-0.25027900
O	-0.01084100	1.52011100	-0.55239200
C	-4.76210100	1.55828200	-0.48638500
C	-4.51770500	2.48534900	0.72885200
C	-3.76272300	1.91262400	-1.61125100
C	-6.18254800	1.84213300	-1.01157800
C	-6.76421700	-3.10128100	0.10254200
C	-6.48809500	-4.53212600	0.59782600
C	-7.94635900	-2.53006000	0.91912900
C	-7.16599600	-3.17776300	-1.38840500
C	-0.82335700	-1.56326100	4.90794800
C	1.49870400	-0.78877700	4.32857400
C	1.34362300	6.91402100	0.73521400
C	2.74808800	7.23814900	0.17577700
C	0.30059000	7.83123400	0.05630200
C	1.34559200	7.22433400	2.24272500
C	0.69327900	3.25257500	-2.76119600
C	1.05372900	4.42193600	-3.69769800
C	-0.66865600	2.68409400	-3.22120800
C	1.77935800	2.16224900	-2.92657300
H	-6.56479000	-0.49555800	-0.55263200
H	-4.20411700	-3.61736900	1.15852300
H	-2.16345800	-3.10976100	2.17064700
H	1.07292200	-1.92476000	2.09553400
H	-0.06131200	-2.98679300	2.94180400
H	-1.64081300	0.24942300	3.29785800
H	-0.40640000	1.08264200	4.24276900
H	0.17723700	2.74507700	3.19273200
H	0.77463700	4.78022700	2.48071100

H	1.18167200	5.70742200	-1.66702900
H	-5.23128400	2.26867100	1.53401900
H	-4.64997300	3.53481700	0.43517500
H	-3.50547900	2.36453800	1.11934400
H	-2.73423000	1.80402000	-1.27114500
H	-3.91408300	2.95060800	-1.93514100
H	-3.90793100	1.26282000	-2.48320500
H	-6.95213800	1.65072700	-0.25375400
H	-6.42000900	1.24627300	-1.90124700
H	-6.25933300	2.89809800	-1.29573200
H	-8.22555400	-1.52427400	0.58645200
H	-6.23121100	-4.55159400	1.66359100
H	-5.67168700	-5.00526400	0.03943000
H	-7.38223600	-5.15205000	0.46373800
H	-7.69285800	-2.47035100	1.98426400
H	-8.83159400	-3.17041600	0.81514600
H	-7.41736800	-2.19186500	-1.79454100
H	-8.04378800	-3.82359000	-1.51854400
H	-6.34839700	-3.58812000	-1.99284000
H	-1.82267700	-1.80612400	4.52496700
H	-0.94772200	-0.85595400	5.73750400
H	-0.38368500	-2.48144000	5.31452100
H	2.13133600	-0.27584000	3.59781100
H	1.96581600	-1.75724500	4.54382700
H	1.50113600	-0.19998600	5.25432600
H	3.51107600	6.60525700	0.64435500
H	3.00783200	8.28629200	0.37146500
H	2.80386000	7.08105100	-0.90706000
H	0.53452800	8.88660900	0.24565900
H	-0.70595500	7.63074500	0.44198300
H	0.27574400	7.68753100	-1.02950200
H	2.09281500	6.62855500	2.78027000
H	0.36559600	7.03771300	2.69756200
H	1.58863600	8.28086200	2.40511000
H	1.08221000	4.06135200	-4.73242200
H	2.03990700	4.84618700	-3.47339000
H	0.31277700	5.22937600	-3.65279100
H	-0.92936200	1.78341800	-2.66645900
H	-0.62552400	2.43118100	-4.28880200
H	-1.46734900	3.42241400	-3.08314000
H	1.52936500	1.27738700	-2.34215800
H	2.76154500	2.53643600	-2.61096800
H	1.85499200	1.86187400	-3.98023300
Al	-0.50135000	0.02391300	0.40489300
O	6.63241000	-3.67679000	1.38750000
C	7.43104600	-3.48277500	2.57197300
H	8.25462700	-2.79115100	2.37253100
H	6.81601200	-3.08748400	3.38536200
H	7.81613800	-4.46997300	2.83047300
O	1.13592400	-0.84786600	-0.00366200
O	4.36332300	-1.85796900	-0.67675800
O	6.18883700	-1.45722900	1.35014800
C	6.06674200	-2.56816900	0.88380300
C	5.30813300	-2.90580900	-0.39873800
C	3.35153700	-1.72044500	0.20790800
C	2.49359400	-0.51110900	-0.15989000
C	2.95656000	0.66202400	0.70698000
C	6.25266400	-2.97571800	-1.59193200
H	4.77031000	-3.84732100	-0.26499700
H	2.68651600	-0.25649700	-1.20773300
H	4.04122000	0.77896600	0.60762000
H	2.72436200	0.47738800	1.75705800
H	2.47541400	1.58652200	0.39034700
H	6.77978600	-2.02413800	-1.71711800
H	5.67534700	-3.19160500	-2.49407600
H	6.98728200	-3.77200400	-1.43896900
O	0.89808100	-1.14243000	-2.54027000
O	1.69314800	-3.58919300	-1.39888200

O	3.29601700	-3.83034300	-2.91343200
O	-0.86954600	-1.01939300	-1.18648800
C	0.24496700	-1.58428800	-1.44360700
C	0.36114700	-3.07287500	-1.14196400
C	2.33476200	-3.21176900	-2.51624200
C	1.84602300	-1.97126000	-3.25378100
C	-0.70485800	-3.87457700	-1.91869000
H	-0.63177300	-4.93686100	-1.66823300
H	-1.70912100	-3.53318500	-1.65407700
H	-0.59061300	-3.75565900	-2.99903300
C	1.25218900	-2.33350800	-4.61341100
H	0.36387000	-2.96206400	-4.49925700
H	0.96606800	-1.41681900	-5.13669700
H	1.99522200	-2.87088300	-5.20934800
H	2.73266600	-1.34662000	-3.39272300
H	0.23782600	-3.19054800	-0.06993200
O	3.18978100	-2.43988200	1.16968800

DLA-IM2

Al	-0.40659700	-1.49243600	-0.35070800
O	0.97830700	-1.09509500	-1.42423100
C	2.28070600	-1.30406200	-1.39982100
C	3.14325200	-0.54030000	-2.24606200
C	4.50547400	-0.81912800	-2.19917100
C	5.10465500	-1.79181700	-1.36454400
C	4.25697700	-2.49938500	-0.53278400
C	2.86264500	-2.27090000	-0.53345500
C	2.06816200	-2.98735100	0.42723900
N	0.79457900	-2.84800200	0.60093500
C	0.11782300	-3.63758900	1.62707700
C	-0.72253400	-4.78887100	1.00431900
C	-1.17955100	-4.40462300	-0.42566800
N	-1.66106500	-3.02394900	-0.55010100
C	-2.94261200	-2.82815600	-0.69594200
C	-3.59188500	-1.58010000	-0.95588600
C	-5.00522500	-1.56054800	-0.91558200
C	-5.71441500	-0.42294100	-1.24535800
C	-4.95011200	0.69325300	-1.65744600
C	-3.56084100	0.74693300	-1.73662100
C	-2.83595900	-0.42707300	-1.35358800
O	-1.53052900	-0.49055500	-1.40776100
C	2.58198800	0.60373100	-3.11071100
C	1.48194500	0.08057100	-4.06428500
C	2.01211700	1.69107700	-2.16628400
C	3.66768700	1.26570800	-3.98124600
C	6.62886500	-1.98524900	-1.39546500
C	7.09280600	-3.08705800	-0.42621600
C	7.07428100	-2.37930300	-2.82222400
C	7.32139500	-0.66353400	-0.98995100
C	0.13214800	-6.06107500	0.88558800
C	-1.93961200	-5.04356100	1.90521500
C	-7.24672800	-0.32271800	-1.19404800
C	-7.89579100	-1.63599600	-0.72223300
C	-7.65530900	0.79781500	-0.21039300
C	-7.79425700	0.00774900	-2.60141900
C	-2.92691800	2.05901800	-2.26303700
C	-3.32816200	2.22774000	-3.74826400
C	-1.38889200	2.12039600	-2.18755200
C	-3.46997900	3.26226800	-1.45310500
H	5.16360800	-0.24221100	-2.83678100
H	4.64316000	-3.24455700	0.15600700
H	2.60767200	-3.68591300	1.07617700
H	-0.53177100	-2.94763200	2.16884800
H	0.83813600	-4.04886300	2.34585200
H	-0.33365700	-4.51038200	-1.11321000
H	-1.95582700	-5.10626600	-0.75556400
H	-3.59621200	-3.70450500	-0.64381100
H	-5.51417200	-2.46979600	-0.61046600
H	-5.49861500	1.58456500	-1.94383800

H	1.88633200	-0.69214500	-4.73065200
H	1.11044100	0.90092800	-4.69173700
H	0.64110000	-0.34185800	-3.51462500
H	1.25883100	1.28414100	-1.49197500
H	1.55062300	2.49729300	-2.75030500
H	2.81662900	2.13088600	-1.56412300
H	4.12435600	0.55622900	-4.68222000
H	4.46506800	1.71811400	-3.37986400
H	3.21394900	2.06811700	-4.57456200
H	6.80784300	-1.61470700	-3.56008700
H	6.64828400	-4.05876000	-0.67205400
H	6.84131100	-2.84706000	0.61359300
H	8.18172200	-3.19843400	-0.48351300
H	6.60522300	-3.32048000	-3.13282000
H	8.16277800	-2.51214200	-2.86176800
H	7.07056000	0.15571300	-1.67251300
H	8.41229600	-0.78170100	-1.00059800
H	7.02078900	-0.35908000	0.01955800
H	1.05942300	-5.86495400	0.33254800
H	-0.40903300	-6.85627200	0.35803400
H	0.40271100	-6.44225200	1.87715900
H	-2.61005600	-4.17627700	1.91967700
H	-1.62589200	-5.23396600	2.93887300
H	-2.51005100	-5.91570800	1.56298300
H	-7.66922100	-2.46840600	-1.39910100
H	-8.98567000	-1.52293500	-0.69334600
H	-7.56608300	-1.91309300	0.28592900
H	-8.74786600	0.88890900	-0.16352100
H	-7.25437100	1.77187900	-0.51185700
H	-7.28713200	0.58446800	0.79990700
H	-7.52395500	-0.77491400	-3.32017100
H	-7.40283100	0.95815100	-2.98073100
H	-8.88850800	0.08657900	-2.57906200
H	-2.91095200	3.15845100	-4.15444100

H	-4.41584900	2.26420500	-3.87873900
H	-2.94600800	1.39523200	-4.35073200
H	-1.01794500	1.98845700	-1.16982400
H	-1.06252400	3.10435700	-2.55080800
H	-0.91257800	1.35943100	-2.80463700
H	-3.21854600	3.16497700	-0.39166000
H	-4.55672000	3.37163200	-1.52949600
H	-3.02701800	4.19463100	-1.82485800
O	2.05128800	6.69587600	1.60051800
C	2.53208100	7.09166000	0.30364500
H	3.27402300	6.37983000	-0.07158300
H	1.71557200	7.20728900	-0.41721600
H	3.00840500	8.06153400	0.45764200
O	0.84190800	0.72720700	1.11678800
O	0.90590200	3.33819500	0.89156700
O	3.15796800	3.36846800	1.03421800
O	1.18398700	5.13875800	2.90786600
C	1.43028600	5.51556900	1.78593900
C	0.95313400	4.73274000	0.55037200
C	2.10324900	2.77888500	1.15826700
C	1.97348500	1.36118000	1.70671300
C	3.24637900	0.55507200	1.48383900
H	1.81771200	1.49119300	2.78621800
H	3.44521900	0.43305600	0.41676100
H	3.11835400	-0.43039800	1.93473300
H	4.10099700	1.06013300	1.94292500
O	0.66910600	-0.70426400	2.91258400
O	-1.78837800	0.39772100	3.69821300
O	-1.79309500	-1.09906900	5.34062100
O	-0.85635900	-0.72027600	1.20349500
C	-0.09734500	0.06942200	1.97038900
C	-0.97288200	1.10236500	2.71944200
C	-1.16933600	-0.48031600	4.50771300
C	0.34170200	-0.59353000	4.30147000

C	-1.92805000	1.83515500	1.80322500
H	-1.35319000	2.38302200	1.05682300
H	-2.58570600	1.12332300	1.30054700
H	-2.53078900	2.54283000	2.38177400
C	0.93054300	-1.80003700	5.00847500
H	0.45663800	-2.71836500	4.65042700
H	2.00529400	-1.85154200	4.80856900
H	0.76996800	-1.72553600	6.08709900
H	0.78558000	0.32880800	4.71522800
H	-0.33467900	1.81736300	3.25247400
H	1.63030900	4.85552800	-0.29568300
C	-0.46666500	5.15021200	0.18738700
H	-0.82545200	4.54986200	-0.65157000
H	-1.13263300	5.00247900	1.04233700
H	-0.49344600	6.20732600	-0.09795100

DLA-IM3

Al	0.79750100	-0.21836100	-0.31552500
O	2.40475100	0.54924700	-0.61725500
C	3.66317200	0.14672500	-0.66529600
C	4.71924900	1.10363900	-0.78163400
C	6.02478500	0.62276700	-0.79871800
C	6.38808100	-0.74224900	-0.72509400
C	5.35205400	-1.65308300	-0.64204800
C	4.00247900	-1.23441000	-0.61619900
C	2.99042800	-2.25482300	-0.56452000
N	1.71441400	-2.04919600	-0.55554100
C	0.80306700	-3.18795200	-0.51195300
C	0.00801400	-3.36069300	-1.83599600
C	-0.08416900	-2.00605400	-2.57200200
N	-0.45080400	-0.86905900	-1.71430000
C	-1.62769100	-0.33106800	-1.91371100
C	-2.10462500	0.90613000	-1.38933600
C	-3.44850100	1.25971500	-1.66203100
C	-3.93867300	2.50502000	-1.33435200
C	-3.00797800	3.43332600	-0.80363300
C	-1.67796600	3.15495600	-0.51385800
C	-1.21808400	1.81243200	-0.73433300
O	-0.01006500	1.43337900	-0.39539800
C	4.41495400	2.60950900	-0.90217200
C	3.57970300	2.86039500	-2.18040100
C	3.64424700	3.09783300	0.34620400
C	5.69728300	3.45715900	-1.01385100
C	7.87334300	-1.13720300	-0.74409000
C	8.06848600	-2.66206800	-0.66898600
C	8.52888900	-0.63164600	-2.04973600
C	8.59073500	-0.49876200	0.46736900
C	0.72634400	-4.34970000	-2.76685600
C	-1.39873100	-3.87097400	-1.48610900
C	-5.41618600	2.89542300	-1.47989700
C	-6.25078800	1.75541500	-2.09118800
C	-5.98558900	3.20097200	-0.07441900
C	-5.55549200	4.14333200	-2.37888100
C	-0.73730700	4.23661800	0.04362000
C	-1.40948000	5.61992300	0.13096100
C	0.48606800	4.37294000	-0.89090500
C	-0.28322600	3.84064400	1.46473600
H	6.82941100	1.34305600	-0.87524100
H	5.54779500	-2.71998600	-0.59466600
H	3.35050400	-3.28943200	-0.53607900
H	0.10229500	-2.98240700	0.29657500
H	1.33663900	-4.11601000	-0.26715700
H	0.89099000	-1.77268100	-3.01365200
H	-0.79906900	-2.09658300	-3.39891000
H	-2.30694500	-0.84729200	-2.59412700
H	-4.08176700	0.50363000	-2.10561100
H	-3.37335300	4.43087100	-0.59261400
H	4.14795900	2.57026800	-3.07329900
H	3.33228400	3.92541600	-2.26973200

H	2.64750700	2.29380800	-2.16585500
H	2.68355700	2.59567700	0.44327600
H	3.46290500	4.17748500	0.27869700
H	4.22789200	2.91048600	1.25592000
H	6.29328400	3.19641500	-1.89673100
H	6.33465800	3.36114400	-0.12653600
H	5.42287900	4.51430900	-1.10647700
H	8.46449500	0.45776200	-2.14515700
H	7.60456700	-3.17439200	-1.52030500
H	7.65023800	-3.08098700	0.25375100
H	9.13813300	-2.90127200	-0.68400000
H	8.04299200	-1.07285200	-2.92817000
H	9.59167100	-0.90354000	-2.07604800
H	8.52245400	0.59452800	0.45217600
H	9.65541400	-0.76474300	0.46772900
H	8.15186100	-0.84698100	1.40967300
H	1.76467600	-4.04279500	-2.94454100
H	0.22545000	-4.41460000	-3.74069500
H	0.74134400	-5.35545800	-2.33050900
H	-1.93192500	-3.12696100	-0.88493200
H	-1.33833300	-4.79886100	-0.90270900
H	-1.98837200	-4.07349300	-2.38752400
H	-5.91128500	1.49943300	-3.10212500
H	-7.30119800	2.06203400	-2.16359100
H	-6.20597900	0.85121400	-1.47443400
H	-7.04340300	3.48743500	-0.14098300
H	-5.44893600	4.02417300	0.41140700
H	-5.90555100	2.31722600	0.56767600
H	-5.16045500	3.94890600	-3.38333800
H	-5.01839600	5.00688800	-1.97074600
H	-6.61033800	4.42939900	-2.47820200
H	-0.68479000	6.34750200	0.51527700
H	-2.26663300	5.62372500	0.81493000
H	-1.74874900	5.97855600	-0.84856200
H	1.02727700	3.43197200	-0.96978100
H	1.17419400	5.13668400	-0.50747700
H	0.17164100	4.67747300	-1.89733000
H	0.12043400	2.83084300	1.48029200
H	-1.12889800	3.87362100	2.16236500
H	0.48243900	4.53888000	1.82863600
O	-4.85913800	-3.92757100	-1.84171300
C	-4.86808800	-5.26452900	-1.30980800
H	-3.89555600	-5.52530700	-0.88004000
H	-5.66141900	-5.40860100	-0.57054600
H	-5.06322600	-5.91066900	-2.16720900
O	-1.71084400	-0.34391200	1.49257700
O	-3.72297600	-2.06225400	1.05531200
O	-5.09081700	-0.24188000	0.94235700
O	-4.38895800	-1.78547800	-1.62909900
C	-4.59437300	-2.84261600	-1.08270500
C	-4.62223300	-3.00437500	0.44264400
C	-4.05384400	-0.76394200	1.27550300
C	-2.96641100	-0.08036600	2.12343600
C	-3.24678200	1.40353000	2.29571700
C	-6.05758600	-2.97106200	0.98251500
H	-4.16592800	-3.95896700	0.71295200
H	-3.01797400	-0.58065800	3.09977200
H	-3.27942700	1.90175700	1.32747700
H	-2.45364400	1.84625300	2.89930700
H	-4.21048500	1.54795600	2.79420900
H	-6.03084900	-2.96352300	2.07670300
H	-6.59221500	-3.87066900	0.66187300
H	-6.59729600	-2.09152200	0.63470800
O	-0.29217600	0.73894700	2.93894300
O	0.59717800	-1.68148000	4.07177100
O	2.38727000	-0.70129000	4.91018600
O	0.47357500	-0.77700600	1.36615400
C	-0.51110000	-0.48829100	2.23582300

C	-0.64231200	-1.58258600	3.32552000
C	1.35787400	-0.59323800	4.28023200
C	0.89364600	0.76597500	3.74686700
C	-0.94086800	-2.97860800	2.82217500
H	-1.82791900	-2.96580600	2.18521300
H	-0.08888300	-3.35423900	2.25266600
H	-1.11306600	-3.65288800	3.66787300
C	2.06065100	1.46730900	3.04871500
H	2.33625100	0.93462000	2.13761500
H	1.77732000	2.48743200	2.78724600
H	2.92390900	1.49808600	3.71876300
H	0.61701000	1.34063500	4.64064700
H	-1.41005300	-1.26085300	4.03635400

DLA-TS2

O	-8.73187400	-1.86090100	-1.39088900
C	-9.94936600	-1.09738300	-1.51564500
H	-9.73279100	-0.09269200	-1.88919200
H	-10.56604000	-1.64487000	-2.22942300
H	-10.45702600	-1.02315100	-0.54962500
O	-2.81999400	0.66552700	-0.24247200
O	-5.54625200	-1.60815200	0.20819900
O	-5.16794600	-0.27434200	-1.59086600
O	-7.94505900	-0.33373200	0.08380000
C	-7.81452400	-1.35614000	-0.55104200
C	-6.59973000	-2.28192800	-0.50013200
C	-4.91754000	-0.62442700	-0.45927600
C	-3.89236800	0.02456900	0.46381100
C	-4.58620100	1.12188900	1.26632200
H	-3.47444900	-0.73332000	1.12858200
H	-4.97207300	1.89041400	0.58988400
H	-3.87773800	1.58226700	1.95944200
H	-5.42251900	0.69908800	1.83009900
O	-0.89672900	-1.15249600	0.57939100
O	-2.89288500	-2.26578000	-0.98309800
O	-2.88836500	-4.06920000	0.28104500
O	-0.82953300	0.65090800	-1.17517900
C	-1.86289000	0.01899200	-0.93919100
C	-2.21292400	-1.23668100	-1.74183600
C	-2.35888700	-3.01052900	0.02810700
C	-1.21797800	-2.46364000	0.90357700
C	-1.01533600	-1.78969900	-2.48425800
H	-0.67963900	-1.07668500	-3.24091300
H	-0.19120700	-1.95934200	-1.79574700
H	-1.29492500	-2.72540100	-2.97758000
C	-0.04399200	-3.45131500	0.90999400
H	-0.39576900	-4.43899200	1.22181400
H	0.41607300	-3.53078500	-0.07541800
H	0.72149000	-3.10725600	1.61039800
H	-1.66934000	-2.49755800	1.91246000
H	-2.98639000	-0.92458300	-2.45073100
Al	0.55339200	-0.00595500	0.53251000
O	1.49930400	1.44553200	-0.06594700
C	1.24674400	2.73413500	-0.11656100
C	2.01582600	3.59454300	-0.96716100
C	1.75645900	4.95893700	-0.91363600
C	0.77290200	5.56969100	-0.09857800
C	0.01608500	4.72453000	0.68807200
C	0.23089000	3.32626200	0.69147400
C	-0.55250600	2.54365400	1.60269000
N	-0.52697100	1.25440900	1.72219800
C	-1.20999200	0.65060500	2.86145100
C	-0.18909800	0.20160500	3.96831100
C	1.26418500	0.34903400	3.47257700
N	1.58699200	-0.38293500	2.23443700
C	2.71679800	-1.02776600	2.24003300
C	3.37398600	-1.67747600	1.14703500
C	4.63505000	-2.25333900	1.43967400
C	5.39041600	-2.86972200	0.46635600

C	4.82790200	-2.90046600	-0.83121400
C	3.59707200	-2.36670900	-1.19531300
C	2.81996300	-1.71542500	-0.17174000
O	1.63805400	-1.19677600	-0.41088000
C	3.01333500	3.01270400	-1.98720300
C	4.05538100	2.08676000	-1.31617400
C	2.19039000	2.22384500	-3.03212600
C	3.79551100	4.11116700	-2.73461300
C	0.59110600	7.09522300	-0.13757900
C	-0.51840200	7.57088700	0.81726400
C	1.91116900	7.78507400	0.27656900
C	0.21384900	7.53570000	-1.57080900
C	-0.34095700	1.12039500	5.19443900
C	-0.45733800	-1.25523600	4.37395000
C	6.76794700	-3.50161100	0.71711900
C	7.20674800	-3.36597700	2.18601600
C	6.71974200	-5.00619000	0.36505300
C	7.82542100	-2.80443700	-0.16928000
C	3.08869400	-2.54140800	-2.64168800
C	4.19484800	-3.02459600	-3.60432700
C	2.56081600	-1.20858300	-3.20942200
C	1.98022700	-3.61704800	-2.64175000
H	2.34164100	5.61148000	-1.54943400
H	-0.76588100	5.11293700	1.33378800
H	-1.20446100	3.10909400	2.27824100
H	-1.76235000	-0.21345000	2.49624700
H	-1.93083600	1.35696000	3.29235700
H	1.47652200	1.40851700	3.28848200
H	1.93510800	0.02152500	4.27716500
H	3.26668000	-1.06313900	3.18651000
H	4.99268100	-2.18264000	2.46227600
H	5.41274200	-3.38571600	-1.60156300
H	4.61564500	2.62983100	-0.54399300
H	4.77680600	1.73405000	-2.06445400
H	3.58660400	1.21960900	-0.85337300
H	1.61139400	1.43042800	-2.55767700
H	2.85037300	1.77322300	-3.78333200
H	1.49312400	2.89209000	-3.55230500
H	4.38628900	4.73363300	-2.05106400
H	3.14062700	4.76747900	-3.31949900
H	4.49227800	3.64184500	-3.43877300
H	2.73807000	7.52003000	-0.39133700
H	-0.29745900	7.31068400	1.85932500
H	-1.49241000	7.14132000	0.55518400
H	-0.61415200	8.66158500	0.76314700
H	2.19996200	7.49801600	1.29473300
H	1.80029900	8.87655300	0.24909600
H	0.98625300	7.26383800	-2.29855200
H	0.08330500	8.62441400	-1.61726200
H	-0.72403300	7.06585700	-1.88942900
H	-0.19055300	2.17343300	4.92480700
H	0.38534300	0.86717500	5.97644700
H	-1.34341300	1.02398500	5.62758400
H	-0.25699700	-1.93961000	3.54395000
H	-1.50088300	-1.39185100	4.68226200
H	0.18071300	-1.55196500	5.21567600
H	7.29017700	-2.31594300	2.49037300
H	8.19075000	-3.82849100	2.32508900
H	6.50819800	-3.86653700	2.86700200
H	7.69885500	-5.47214900	0.53360800
H	6.45126700	-5.17206600	-0.68410300
H	5.98223200	-5.52972300	0.98491800
H	7.88637000	-1.73466600	0.06288100
H	7.59291000	-2.90113400	-1.23551200
H	8.81643600	-3.24623700	-0.00513700
H	3.78709700	-3.07338000	-4.62064900
H	4.56271100	-4.02772600	-3.36014100
H	5.04952500	-2.33770800	-3.62279000

H	1.75238600	-0.80258200	-2.60754900
H	2.20007200	-1.35071100	-4.23626300
H	3.36589400	-0.46693500	-3.23552800
H	1.17677700	-3.36046400	-1.95329200
H	2.38587100	-4.58938500	-2.33684700
H	1.54573900	-3.72783700	-3.64365800
H	-6.27004300	-2.46312300	-1.52594700
C	-6.89431600	-3.60188700	0.23562100
H	-6.00159600	-4.23043000	0.27753900
H	-7.20065100	-3.37965100	1.26086300
H	-7.69237900	-4.17254900	-0.24769400

DLA-IM4

Al	1.84910400	-0.09583700	0.19398800
O	-4.28990600	-1.84756300	2.33286300
O	1.53165300	-0.08426100	1.93406800
O	-1.75719900	0.58331700	3.08696700
O	-0.75730600	-1.42774400	2.82144200
O	-2.92614700	-0.71759400	0.94266600
C	-3.36479400	-0.91083000	2.05625500
C	-3.02833600	-0.06086200	3.28139400
C	-0.68720100	-0.21573600	2.84574100
C	0.58061300	0.62320700	2.65246500
C	1.12719100	1.00351300	4.03692800
C	-3.12571100	-0.77643400	4.62459000
H	-3.74200900	0.77123000	3.25638400
H	0.25975900	1.54390500	2.14733300
H	1.42622800	0.09964000	4.57916600
H	0.38510400	1.55081000	4.62913100
H	2.01093000	1.63546400	3.90357500
H	-4.15522300	-1.09785700	4.80282800
H	-2.83283100	-0.08312700	5.41905500
H	-2.47068200	-1.64866600	4.65123500
O	3.48447500	0.31414300	-0.43609700
C	4.66592900	-0.27252700	-0.44931100
C	5.82651400	0.48021700	-0.80605300
C	7.04513500	-0.18950100	-0.83040600
C	7.21956800	-1.56054500	-0.52530200
C	6.08501100	-2.26704700	-0.16982500
C	4.81491100	-1.64904600	-0.12464500
C	3.68403200	-2.45395400	0.26009400
N	2.46335400	-2.03886800	0.35898000
C	1.40812800	-2.97009200	0.75783900
C	0.46998300	-3.32241900	-0.43493500
C	0.46097700	-2.17563500	-1.47531800
N	0.35579200	-0.82757300	-0.90222000
C	-0.76043100	-0.17345400	-1.07552900
C	-1.00941800	1.19420300	-0.73721500
C	-2.33518600	1.67296600	-0.85338800
C	-2.64791300	2.98947400	-0.59032000
C	-1.57053100	3.84422700	-0.23892100
C	-0.24370300	3.44918700	-0.12860700
C	0.05366900	2.06430800	-0.35412500
O	1.27647600	1.60883100	-0.23092400
C	5.71037100	1.97892200	-1.14446300
C	4.78834600	2.16539600	-2.37219600
C	5.13576500	2.74969800	0.06874200
C	7.07597500	2.60451700	-1.48788700
C	8.62359900	-2.18239300	-0.60016700
C	8.61522200	-3.67910600	-0.24154200
C	9.17761400	-2.03427300	-2.03592100
C	9.56622800	-1.45599100	0.38656800
C	0.98363400	-4.58156100	-1.15258300
C	-0.94910300	-3.55393100	0.10769500
C	-4.07931000	3.54544800	-0.63953900
C	-5.09810400	2.47938700	-1.08293900
C	-4.48028500	4.03797000	0.76989400
C	-4.15320100	4.72606000	-1.63419200
C	0.87811600	4.44048100	0.23807200

C	0.34638900	5.87403900	0.42721800	H	-1.36131800	-2.63542000	0.53429600
C	1.92805600	4.47328900	-0.89766400	H	-0.94209800	-4.31394200	0.89887300
C	1.55986100	4.01764100	1.56165600	H	-1.61912600	-3.90301900	-0.68881400
H	7.92996400	0.37309100	-1.10146300	H	-4.87056300	2.10193400	-2.08792400
H	6.13959100	-3.32097700	0.08531800	H	-6.10339800	2.91549100	-1.11693300
H	3.89645300	-3.50390700	0.49214700	H	-5.13474400	1.62945500	-0.39337900
H	0.83808400	-2.47585800	1.54759300	H	-5.49951800	4.44492600	0.76269500
H	1.83646100	-3.89200100	1.17346400	H	-3.80919500	4.82503700	1.13180400
H	1.39474700	-2.20373800	-2.04786300	H	-4.44774300	3.21350100	1.49198700
H	-0.35897500	-2.34529300	-2.18470000	H	-3.87729100	4.40309300	-2.64518500
H	-1.59112900	-0.70667700	-1.54565000	H	-3.48276900	5.54521300	-1.35199500
H	-3.10202300	0.95516500	-1.12003200	H	-5.17186100	5.13235800	-1.67302500
H	-1.81078800	4.88135100	-0.03876100	H	1.18113900	6.54084800	0.67253500
H	5.20876300	1.65906300	-3.25058800	H	-0.37808900	5.94191700	1.24789100
H	4.69250200	3.23176600	-2.61359100	H	-0.12687300	6.26239000	-0.48274900
H	3.79114700	1.76546600	-2.18640600	H	2.37732800	3.49140500	-1.04666000
H	4.13661500	2.39921800	0.32760400	H	2.72707500	5.18479600	-0.65292800
H	5.07379400	3.82062300	-0.16119400	H	1.46884300	4.79451900	-1.84106500
H	5.78539500	2.63034700	0.94492700	H	2.00260400	3.02429000	1.48255800
H	7.53898800	2.13439500	-2.36401200	H	0.83705500	4.01335500	2.38696800
H	7.78148400	2.54222900	-0.65050800	H	2.35672500	4.72785000	1.81722600
H	6.93855900	3.66658400	-1.72170400	C	-4.73958800	-2.59823300	1.18346000
H	9.24922200	-0.98414800	-2.33960900	H	-3.88352500	-3.10599200	0.73316100
H	7.98302300	-4.25798500	-0.92526700	C	-5.34979000	-1.64010800	0.16430300
H	8.26086700	-3.85057900	0.78172500	O	-6.01418500	-0.66865300	0.44789600
H	9.63162600	-4.08387400	-0.31028700	O	-5.10050900	-2.06451200	-1.08914900
H	8.53265900	-2.54733700	-2.75918300	C	-5.78950300	-3.58458000	1.67212100
H	10.18272400	-2.46896400	-2.10744100	H	-5.34924300	-4.26822400	2.40403900
H	9.65203200	-0.38786200	0.15877600	H	-6.62281000	-3.04890100	2.13626100
H	10.57488300	-1.88605700	0.34204800	H	-6.17350400	-4.16334800	0.82669400
H	9.20128700	-1.54899100	1.41622000	C	-5.78203300	-1.35131600	-2.13550600
H	2.03221000	-4.46610800	-1.45535100	H	-5.64509000	-0.27604400	-1.99514200
H	0.39680800	-4.79374500	-2.05528800	C	-7.27688400	-1.66611000	-2.05636400
H	0.91512900	-5.45736100	-0.49673800	O	-7.76487100	-2.57772600	-1.42670600

O	-7.96356800	-0.80393300	-2.82034400
C	-9.39151300	-1.00944900	-2.86395400
H	-9.81913700	-0.91778600	-1.86182800
H	-9.77911900	-0.22735900	-3.51761700
H	-9.62475000	-1.99799200	-3.26967700
C	-5.18085900	-1.81417300	-3.45627100
H	-4.10944600	-1.59204800	-3.47317400
H	-5.31920900	-2.89254900	-3.58449100
H	-5.66207400	-1.29151300	-4.28758100

LLA-TS1’

O	-0.82255900	-0.29557200	-0.98739100
C	-2.09452100	-0.09659600	-1.22484800
C	-2.96300600	-1.19809300	-1.50126700
C	-4.32934200	-0.93777900	-1.56547500
C	-4.91222600	0.34623000	-1.45917100
C	-4.03677300	1.41383700	-1.38825800
C	-2.64218400	1.22249700	-1.28670800
C	-1.78119100	2.35917300	-1.43965000
N	-0.48880600	2.36496200	-1.31114400
C	0.25619900	3.45999200	-1.92304400
C	0.76250100	3.04949200	-3.34721300
C	0.84638000	1.50406300	-3.48245300
N	1.51026200	0.83163100	-2.36796900
C	2.69036400	0.32296800	-2.57038500
C	3.45168800	-0.47159600	-1.65661400
C	4.76685000	-0.82983700	-2.04271200
C	5.53789900	-1.67507200	-1.27439600
C	4.92960500	-2.19827000	-0.10439800
C	3.64291400	-1.91079300	0.32612200
C	2.87238900	-0.98058800	-0.45209400
O	1.66180100	-0.65112800	-0.09212300
C	-2.37583400	-2.59476200	-1.78815100
C	-1.35073300	-2.47701900	-2.94514800
C	-1.68090400	-3.18276300	-0.53991400
C	-3.46017100	-3.59544300	-2.23311100
C	-6.43118500	0.58684700	-1.47179100
C	-6.82355900	1.34637100	-2.75915700
C	-7.23140700	-0.72709800	-1.41285400
C	-6.82063100	1.42646500	-0.23210100
C	-0.22983900	3.54296600	-4.41362900
C	2.14773500	3.67460300	-3.57038500
C	6.97709700	-2.07909300	-1.62630200
C	7.92747800	-1.64821300	-0.48472300
C	7.05801500	-3.61293700	-1.80746200
C	7.46275200	-1.41725500	-2.92874100
C	3.05030300	-2.56337100	1.58874200
C	4.03667000	-3.54174300	2.25618600
C	1.77979800	-3.36096000	1.21092300
C	2.69358700	-1.47428300	2.62568600
H	-4.99352000	-1.77645400	-1.72004600
H	-4.41032600	2.43550400	-1.40442900
H	-2.26887900	3.28307700	-1.77585300
H	1.11303300	3.67865100	-1.28818900
H	-0.36124500	4.36642800	-2.00041700
H	-0.16642900	1.09217700	-3.54973700
H	1.35343900	1.26999400	-4.42892900
H	3.14958900	0.48123800	-3.55364900
H	5.14943000	-0.41206200	-2.96944100
H	5.52146200	-2.88207700	0.49139200
H	-1.82355100	-2.04916900	-3.83798100
H	-0.96934300	-3.47155300	-3.20730800
H	-0.50389000	-1.85017300	-2.66174000
H	-0.98603900	-2.47222100	-0.09313600
H	-1.12002200	-4.08587400	-0.81105300
H	-2.42897000	-3.47308900	0.20441700
H	-3.98367800	-3.26266300	-3.13729500
H	-4.20293300	-3.76944700	-1.44603300
H	-2.99163800	-4.56093000	-2.45448700

H	-6.98874000	-1.28279200	-0.50232000
H	-6.56844600	0.76177500	-3.65097000
H	-6.30570500	2.30941200	-2.83149500
H	-7.90265100	1.54477000	-2.77698800
H	-7.04212600	-1.36640700	-2.28342400
H	-8.30465400	-0.50507900	-1.39916500
H	-6.51514000	0.91301400	0.68434800
H	-7.90624500	1.58470300	-0.20464500
H	-6.34485800	2.41318200	-0.24436600
H	-1.24242400	3.17132200	-4.21290900
H	0.05919000	3.19762400	-5.41359700
H	-0.26800600	4.63821700	-4.43516600
H	2.87833700	3.25938600	-2.86714100
H	2.11441200	4.76077300	-3.42046700
H	2.50508700	3.48991500	-4.59068800
H	7.89614200	-0.56177100	-0.34363300
H	8.96119600	-1.93524800	-0.71514000
H	7.65644400	-2.11620900	0.46757800
H	8.08295700	-3.91785200	-2.05350700
H	6.39794000	-3.94280100	-2.61778900
H	6.76180000	-4.14634900	-0.89811100
H	7.45051800	-0.32359500	-2.85510300
H	6.84599100	-1.71111300	-3.78603600
H	8.49306100	-1.72452200	-3.14127000
H	3.56073200	-3.98392200	3.13857400
H	4.95254600	-3.04087100	2.59344300
H	4.31866700	-4.36430300	1.58786500
H	1.02432200	-2.70848600	0.77831400
H	1.34979700	-3.82752500	2.10549600
H	2.02349700	-4.15368800	0.49253100
H	1.94302400	-0.79524200	2.22784000
H	3.59020900	-0.90325700	2.89822600
H	2.28902300	-1.94131900	3.53237600
Al	0.60002800	0.77386700	-0.55506200
O	0.13686600	1.24897100	1.26987300
O	2.57414100	1.72389700	2.16003900
O	0.73137000	3.70264700	3.03331500
O	0.91042800	2.98294500	5.12265300
O	1.99354400	2.08232600	0.05203500
C	1.76084400	2.30639500	1.27247000
C	1.20627900	3.66162900	1.67138900
C	1.26293800	2.89259700	3.97179200
C	2.28349500	1.82903200	3.56845000
C	2.27001400	4.74089200	1.47195300
H	3.15547200	4.52428100	2.07853300
H	2.57196200	4.78244900	0.42181700
H	1.86597800	5.71151800	1.77249400
C	3.60538500	2.03963700	4.29913500
H	3.42873800	2.04696900	5.37696900
H	4.29059000	1.22685000	4.04517200
H	4.06359200	2.98966100	4.00489800
H	1.84752600	0.86860300	3.85990000
H	0.32932000	3.85237600	1.05465800
C	-0.89059700	1.06808900	2.21649100
C	-1.31412900	-0.40816400	2.26681300
O	-0.53748300	-1.32453600	2.41290500
O	-2.66052000	-0.55783200	2.22415000
C	-3.14614200	-1.88356800	2.49253700
H	-2.44056600	-2.61155200	2.09438200
C	-4.48858400	-2.03367600	1.79296300
O	-5.27409900	-1.15169200	1.53830800
O	-4.71344200	-3.34742100	1.56627000
C	-3.30643400	-2.07953600	4.00181500
H	-2.32741800	-1.98937300	4.48011300
H	-3.98160100	-1.32170900	4.41106300
H	-3.71042000	-3.07444500	4.21257600
C	-6.00267400	-3.68051300	1.02366900
H	-6.06349900	-4.76850200	1.05984100

H	-6.79971000	-3.22544100	1.61776500
H	-6.08124900	-3.33053600	-0.00836100
C	-2.04781900	2.05372500	2.06458000
H	-1.65203500	3.07267300	2.07554800
H	-2.59790400	1.88011100	1.14018900
H	-2.74837100	1.94753300	2.89798500
H	-0.45944600	1.21729300	3.21766900
